# Efficacy and safety of commercial Chinese polyherbal preparation combined with oxaliplatin-based chemotherapy for gastric cancer: a systematic review and network meta-analysis

**DOI:** 10.3389/fphar.2025.1645079

**Published:** 2025-09-09

**Authors:** Bei Pan, Honghao Lai, Ning Ma, Xin He, Xiaowei Liu, Xiyuan Deng, Jinhui Tian, Long Ge, Kehu Yang

**Affiliations:** ^1^ Evidence-Based Medicine Center, School of Basic Medical Sciences, Lanzhou University, Lanzhou, China; ^2^ Centre for Evidence-Based Social Science/Center for Health Technology Assessment, School of Public Health, Lanzhou University, Lanzhou, China; ^3^ Gansu Key Laboratory of Evidence-Based Medicine, Lanzhou University, Lanzhou, China; ^4^ The First Clinical Medical College of Lanzhou University, Lanzhou, China; ^5^ Gansu Provincial Maternity and Child-care Hospital, Lanzhou, China

**Keywords:** network meta-analysis, gastric cancer, commercial Chinese polyherbal preparations, oxaliplatin-based chemotherapy, systematic review

## Abstract

**Objective:**

To evaluate the efficacy, safety, and quality of evidence for commercial Chinese polyherbal preparations (CCPPs) in combination with oxaliplatin-based chemotherapy (OX) for gastric cancer (GC).

**Methods:**

We searched six databases for randomized controlled trials (RCTs) comparing CCPPs plus OX to other active treatments for GC. The risk of bias was assessed using a modified version of Cochrane risk of bias tool. A frequentist network meta-analysis using a random-effects model was performed to estimate the relative effectiveness between treatments. We used GRADE to assess the certainty of evidence, to categories the interventions, and to present the findings.

**Results:**

One-hundred-and-eighty-nine RCTs involving 15,320 participants that reported 27 CCPPs were identified. Moderate to high certainty evidence showed that Compound Mylabris preparations (Disease Control Rate (DCR): 1.51, 1.13–2.03) is among the most effective CCPPs for improving DCR, while Kangai Injection (objective response rate (ORR): 1.40, 1.28 to 1.77; quality of life (QoL): 1.46, 1.11–1.94), Huachansu preparations (ORR: 1.28, 1.15–1.43), Ya Dan Zi Oil Emulsion Injection (QoL: RR 1.26, 1.06–1.51), Aidi Injection (QoL: 1.30, 1.13–1.50), Lentinan (QoL: 1.27, 1.05–1.54), and Yangzheng Xiaoji Capsules (ORR: 1.36, 1.05–1.77) showed intermediate efficacy for ORR and QoL. Regarding immune function improvement, with moderate to high certainty evidence, Shenmai Injection (CD3^+^: 10.03, 1.69 to 18.37; CD4^+^: 8.33, 0.64–16.02), Ginseng Polysaccharide Injection (CD3^+^: 10.55, 1.89–19.21), Huachansu preparations (CD3^+^: 7.42, 2.51–12.34), Kangai Injection (CD3^+^: 11.65, 6.81 to 16.50; CD4^+^/CD8^+^: 0.25, 0.02–0.47), Lentinan (CD4^+^: 9.43, 3.78–15.08), Shenqi Fuzheng Injection (CD4^+^: 5.72, 3.68–7.76), and Yangzheng Xiaoji Capsules (CD3^+^: 9.32, 0.84–17.80) showed improvements in specific immune parameters.

**Conclusion:**

No CCPP was optimal for all endpoints, Compound Mylabris showed superior tumor response, and Kangai Injection offered the most favorable risk-benefit profile, providing broad efficacy and statistically significantly reducing AEs. The choice of adjuvant CCPP should be individualized based on specific therapeutic priorities, balancing efficacy and safety.

**Systematic Review Registration:**

https://www.crd.york.ac.uk/PROSPERO/view/CRD42025646173.

## 1 Background

Gastric cancer (GC) remains a significant challenge in global health, ranking in fifth of incidence including 1.089 million cases, and third of cancer-related mortality including 768,000 deaths (Sung et al., 2021). Males show the highest prevalence in East Asia twice than females, particularly in China, Japan, and South Korea (Sung et al., 2021). China accounts for 48.6% of global GC-related mortality and the largest proportion of gastric cancer cases, with 358,700 new cases and 260,400 deaths in 2022 (Chen et al., 2022). Because of inapparent symptoms in early stage, GC is often identified at a late stage, leading to unfavorable outcomes and few available therapies (Smyth et al., 2020).

According to the recommendation of clinical practice guideline, oxaliplatin-based regimens (OX) are recommended as first-line treatments for advanced gastric cancer (Ajani et al., 2022; Lordick et al., 2022). The NCCN guidelines suggested FOLFOL and XELOX as standard treatments (Ajani et al., 2022). Furthermore, the ESMO guidelines also emphasized that oxaliplatin in combination with fluoropyrimidines as standard options for the first-line therapy for GC (Lordick et al., 2022). However, due to chemotherapy-related adverse effects OX regimens also have some challenges such as myelosuppression, gastrointestinal toxicity, and peripheral neuropathy, which impact quality of life (QoL) for GC patients and may lead to dose modification or treatment interruption, further affecting long-term outcomes (Smyth et al., 2020).

Commercial Chinese polyherbal preparations (CCPPs) have been receiving growing attention in the managing of GC in recent years due to its multi-target and multi-pathway mechanisms (Liu et al., 2024). Liu and colleagues’ studies showed that CCPP can improve clinical outcomes for GC patients, because CCPP could effectively inhibit the metastasis, proliferation, and invasion of tumor cells, as well as enhancing chemotherapy efficacy while reducing its toxic side effects (Liu et al., 2024). CCPP, which is a standardized form of herbal medicine, are widely used as an adjuvant therapy because of its distinctive advantages such as convenient administration, stable composition, and consistent efficacy (Wang et al., 2024). Systematic reviews (Wu et al., 2019; Wang et al., 2020; Li, 2019; Wei, 2014) have suggested that Xiao-Ai-Ping Injection (XAPI), cantharidin-based preparations, Aidi Injection, and Kangai Injection, can improve the objective response rate (ORR), disease control rate (DCR), and Karnofsky Performance Status (KPS) improvement rate, while also reducing the incidence of toxic side effects, such as leukopenia, liver damage, nausea and vomiting, and renal impairment, when combined with OX. CCPP exhibits multi-target and synergistic effects, and different dosage forms may influence drug release, absorption, and anticancer efficacy. However, no studies systematically compare the relative efficacy of various CCPPs till now, and assessed the quality of evidence using the GRADE (Grading of Recommendations, Assessment, Development, and Evaluation) approach.

Therefore, we aim to conduct a systematic review and network meta-analysis of randomized controlled trials (RCTs) to evaluate the relative effectiveness, safety, and certainty of evidence regarding different CCPPs in combination with oxaliplatin-based chemotherapy for the adjuvant treatment for GC. Our findings will provide comprehensively convincing evidence for clinical decision-making and the optimization of individualized treatment strategies.

## 2 Methods

This study was reported according to PRISMA 2020 (the preferred reporting items for systematic reviews and meta-analyses) (Page et al., 2021) and PRISMA—NMA (the Preferred Reporting Items for Systematic Reviews and Meta Analyses-Network Meta-analyses) guidelines (Hutton et al., 2015). The protocol of this study was registered with PROSPERO (CRD42025646173) (Pan and Ge, 2024).

### 2.1 Standard evaluation of the composition of CCPPs

To ensure methodological rigor, the phytochemical characterization of Traditional Chinese Medicine Ingredients in this study strictly adhered to the ConPhyMP consensus guidelines for medicinal plant extract analysis. We standardized botanical nomenclature for all medicinal plant components using Medical Plant Name Service (MPNS, http://mpns.kew.org/mpns-portal/) and/or Plants of the World Online (POWO, http://www.plantsoftheworldonline.org). Composition summaries were structured according to the Four Pillars of Ethnopharmacology framework (Table 1). More detail information on the CCPPs included in our review could be found in Supplementary Appendix S1.

**TABLE 1:** The summary of botanical nomenclature for all medicinal plant components of included CCPPs.

Pharmacopeial drug name	Composition	Source species (family)	Level of reporting in the original study
Compound Kushen Injection	Sophora flavescens Aiton (Kushen in Chinese)	Fabaceae	Inadequate
Shenqi Fuzheng Injection	Codonopsis pilosula (Franch.) Nannf (Dangshen in Chinese) Astragalus mongholicus Bunge (Huangqi in Chinese)	Campanulaceae Fabaceae	Inadequate
Compound Mylabris preparations	Panax ginseng C. A. Mey (Renshen in Chinese) Astragalus mongholicus Bunge (Huangqi in Chinese) Eleutherococcus senticosus (Rupr. and Maxim.) Maxim. (Ciwujia in Chinese) Sparganium stoloniferum (Buch.-Ham. ex Graebn.) Buch.-Ham. ex Juz. (Sanleng in Chinese) Scutellaria barbata D.Don (Banzhilian in Chinese) Curcuma phaeocaulis Valeton (Ezhu in Chinese) Cornus officinalis Siebold and Zucc. (Shanzhuyu in Chinese) Ligustrum lucidum W.T.Aiton (Nvzhenzi in Chinese) Glycyrrhiza uralensis Fisch. ex DC. (Gancao in Chinese)	Araliaceae Fabaceae Typhaceae Lamiaceae Zingiberaceae Cornaceae Oleaceae	Inadequate
Aidi Injection	Panax ginseng C. A. Mey (Renshen in Chinese) Astragalus mongholicus Bunge (Huangqi in Chinese) Eleutherococcus senticosus (Rupr. and Maxim.) Maxim. (Ciwujia in Chinese)	Araliaceae Fabaceae	Inadequate
Ya Dan Zi Oil Emulsion Injection	Brucea javanica (L.) Merr. (Ya dan zi oil in Chinese) Soya Lecithin (Dadou Linzhi in Chinese)	Simaroubaceae	Inadequate
Kangai Injection	Astragalus mongholicus Bunge (Huangqi in Chinese) Sophora flavescens Aiton (Kushen in Chinese) Panax ginseng C. A. Mey (Renshen in Chinese)	Fabaceae Araliaceae	Inadequate
Yangzheng Xiaoji Capsules	Astragalus mongholicus Bunge (Huangqi in Chinese) Ligustrum lucidum W.T.Aiton (Nvzhenzi in Chinese) Panax ginseng C. A. Mey (Renshen in Chinese) Curcuma phaeocaulis Valeton (Ezhu in Chinese) Rhinacanthus nasutus (L.) Kurz (Lingzhi in Chinese) Broteroa atractylodes (C.Winkl.) Kuntze. (Baishu in Chinese) Scutellaria barbata D.Don (Banzhilian in Chinese) Smilax glabra Roxb. (Fuling in Chinese) Cynanchum paniculatum (Bge.) Kitag (Xuchangqing)	Fabaceae Oleaceae Araliaceae Zingiberaceae Acanthaceae Asteraceae Lamiaceae Asclepiadaceae	Inadequate
Kanglixin Capsules	Ferula sinkiangensis K.M.Shen (Awei in Chinese) Rheum palmatum L. (Dahuang in Chinese) Curcuma Longa L. (Jianghuang in Chinese) Terminalia chebula Retz (Hezi in Chinese) Syzygium aromaticum (L.) Merr. and L.M.Perry (Dingxiang in Chinese)	Apiaceae Polygonaceae Zingiberaceae Combretaceae Myrtaceae	Inadequate
Weimaining Capsules	Fagopyrum cymosum (Trevir.) Meisn. (Jinqiaomai in Chinese)	Polygonaceae	Inadequate
Shenmai Injection	Panax ginseng C.A.Mey (Hongshen in Chinese) Ophiopogon japonicus (Thunb.) Ker Gawl. (Maidong in Chinese)	Araliaceae Asparagaceae	Inadequate
Pingxiao Capsules	Curcuma wenyujin Y. H. Chen et C. Ling (Yujin in Chinese) Strychnos nux-vomica L. (Maqianzi in Chinese) Agrimonia pilosa Ledeb. (Xianhecao in Chinese) Toxicodendron vernicifluum (Stokes) F.A.Barkley (Ganqi in Chinese) Citrus × aurantium L. (Zhiqiao in Chinese)	Zingiberaceae Loganiaceae Rosaceae Anacardiaceae Rutaceae	Inadequate
Xiaoaiping Injection	Gongronemopsis tenacissima (Roxb.) S.Reuss, Liede and Meve (Tongguanteng in Chinese)	Apocynaceae	Inadequate
Kanglaite Injection	Coix lacryma-jobi var. ma-yuen (Rom.Caill.) Stapf (Yiyiren in Chinese)	Poaceae	Inadequate
Huai’er Granules	Trametes robiniophila Murr (Huai’er in Chinese)	Smilacaceae	Inadequate
Xihuang Capsules	Moschus berezovskii Flerov (Shexiang in Chinese) Commiphora myrrha (T.Nees) Engl. (Moyao in Chinese) Boswellia Roxb. ex Colebr. (ruxiang in Chinese)	Cervidae Burseraceae	Inadequate
Astragalus preparations	Astragalus mongholicus Bunge (Huangqi in Chinese)	Fabaceae	Inadequate
Qizhen Capsule	Astragalus mongholicus Bunge (Huangqi in Chinese) Panax notoginseng (Burk.) F. H. Chen (Sanqi in Chinese)、 Isatis indigotica Fort. (Daqingye in Chinese)、 Paris polyphylla Smith var.yunnanensis (Franch.)Hand.-Mazz. (Chonglou in Chinese)	Fabaceae Araliaceae Brassicaceae liliaceae	Inadequate
Shengxue Granules	Codonopsis pilosula (Franch.) Nannf (Dangshen in Chinese) Smilax glabra Roxb. (Fuling in Chinese) Atractylodes macrocephala Koidz. (Baishu in Chinese) Astragalus mongholicus Bunge (Huangqi in Chinese) Ziziphus jujuba Mill. (Dazao in Chinese)	Campanulaceae Smilacaceae Asteraceae Fabaceae Rhamnaceae	Inadequate
Diyu Shengbai Tablets	Sanguisorba officinalis L (Diyu in Chinese)	Rosaceae	Inadequate
Shenlian Capsule	Dolichos lablab L (Baibiandou in Chinese) Scutellaria barbata D.Don (Banzhilian in Chinese) Cullen corylifolium (L.) Medik. (Buguzhi in Chinese) Salvia miltiorrhiza Bunge (Danshen in Chinese) Curcuma phaeocaulis Valeton (Ezhu in Chinese) Stephania tetrandra S.Moore (Fangji in Chinese) Sophora flavescens Aition (Kushen in Chinese) Semen Armeniacae Amarum (Kuxingren in Chinese) Sparganium stoloniferum (Buch.-Ham. ex Graebn.) Buch.-Ham. ex Juz. (Sanleng in Chinese) Sophora tonkinensis Gagnep (Shandougen in Chinese) Prunus mume (Sieb.)Sieb.etZucc (Wumei in Chinese)	Fabaceae Lamiaceae Zingiberaceae Menispermaceae Rosaceae Typhaceae	Inadequate
Compound Tianxian Capsules	Trichosanthes kirilowii Maxim (Tianhuafen in Chinese) Clematis chinensis Osbeck (Weilingxian in Chinese) Solanum nigrum L (Longkui in Chinese) Arisaema erubescens (Wall.) Schott (Sannanxing in Chinese) Boswellia sacra Flück. (ruxiang in Chinese) Commiphora myrrha (T.Nees) Engl. (Moyao in Chinese) Panax ginseng C. A. Mey (Renshen in Chinese) Astragalus mongholicus Bunge (Huangqi in Chinese) Polyporus umbellatus (Pers.) Fries (Zhuling in Chinese)	Cucurbitaceae Ranunculus Solanaceae Araceae Burseraceae Araliaceae Fabaceae Smilacaceae	Inadequate
Zhenqi Fuzheng Granules	Astragalus mongholicus Bunge (Huangqi in Chinese) Ligustrum lucidum W.T.Aiton (Nvzhenzi in Chinese)	Fabaceae Oleaceae	Inadequate
Shenfu Injection	Panax ginseng C.A.Mey (Hongshen in Chinese) Aconitum carmichaelii Debeaux (Heifupian in Chinese)	Araliaceae Ranunculaceae	Inadequate

### 2.2 Search strategy and information sources

Six databases were searched including PubMed, Cochrane Central, Embase, CNKI, CBM, and WanFang from inception to 6 August 2025, using a comprehensive search strategy. Supplementary Appendix S2 presents the search strategies for all databases. And to identify unpublished or ongoing studies, we systematically searched ClinicalTrials.gov and the Chinese Clinical Trial Registry (ChiCTR). We also tracked reference lists of relevant studies to identify potentially missed citations. We did not applied restrictions for the year of publication, and publication status. We only considered studies published in English and Chinese.

### 2.3 Eligibility criteria

Randomized controlled trials (RCTs) that compared CCPP alone or in combination with OX against other eligible active treatments for the management of GC in adults (aged ≥18 years) were included. GC should be diagnosed by gastroscopy or surgical pathology. CCPPs are defined as formulations based on CCPPs theories, prepared from medicinal botanical drug or herbal extracts according to standardized prescriptions and manufacturing processes. They typically dosage form includes oral formulations (such as pills, tablets, capsules, and granules) as well as injectable preparations, all of which should be approved by the National Medical Products Administration (NMPA) of China for clinical application. The outcomes of interest included DCR, ORR, improvement rate in quality of life (QoL), survival rate (1-year overall survival rate and 2-year overall survival rate), traditional Chinese medicine (TCM) syndrome score, immune function indicators (CD3+T cells CD4+T cells, CD8+T cells, CD4+/CD8+ratio, and natural killer (NK) cells), tumor markers (carcinoembryonic antigen (CEA), carbohydrate antigen 125 (CA125), carbohydrate antigen 199 (CA199), and carbohydrate antigen 724 (CA724)), and adverse events (AEs). The definition of each outcome is described in Supplementary Appendix S3.

Cross-over trials and cluster-randomised trials were excluded. Additionally, studies focused on non-pharmacological treatments, including acupuncture, acupoint sticking therapy, and other traditional Chinese medicine therapies, as well as combination therapy studies (e.g., combination of drug A and drug B versus drug A alone), and adjuvant treatment with radiotherapy, immunotherapy, and neoadjuvant chemotherapy.

### 2.4 Study selection

We first used EndNote to remove duplicate records and then imported the remaining references into Rayyan (Ouzzani et al., 2016), an online literature management software. Following the standardized study eligibility form, teams of two reviewers (PB and HX, MN and LXW) independently screened the titles and abstracts of all retrieved bibliographic records. Any conflicting cases were subjected to full-text evaluation. Discrepancies were resolved through consensus.

### 2.5 Data extraction

A standardized data collection form was developed for reviewers to extract relevant data. Paired reviewers (PB and HX, MN and LXW) independently extracted the following data in duplicate: first author, year of publication, single-center or multi-center status, clinical stage, duration of the interventions, TCM syndrome, total sample size, age, gender distribution, gastric cancer diagnosis, number of patients for each arm, drug name, chemotherapy regimen, route of administration, and outcomes of interest (efficacy endpoints and AE).

### 2.6 Risk of bias assessment

Paired reviewers assessed (PB and HX, MN and LXW) the risk of bias for each included RCTs in duplicate using a modified version of Cochrane risk of s tool bias (Evidence Partners, 2011), which include six items as following: allocation sequence adequately generated, allocation adequately concealed, blinding of patients and healthcare providers, blinding of outcome assessors, missing outcome data, and selective outcome reporting. We applied response options of “definitely or probably yes” (low risk of bias) and “definitely or probably no” (high risk of bias). Conflicts were resolved by a third reviewer (GL). We rated individual studies as having a low risk of bias if at least four out of six questions were rated as low risk (definitely or probably yes). Otherwise, we classified the studies as having a high risk of bias.

### 2.7 Data synthesis and statistical method

We selected the mean difference (MD) with 95% confidence intervals (CIs) as the measure of effect for continuous outcomes. For binary variables, we calculated risk ratios (RRs) with 95% CIs. We performed pairwise meta-analyses using a random-effects model for each direct comparison. A random-effects arm-based network meta-analysis (NMA) was conducted within the frequentist framework using the ‘netmeta’ package as primary analysis (Rücker et al., 2019). We also conducted fixed-effect model analysis as sensitivity analysis to assess the robustness of results. Inconsistencies between direct and indirect estimates for each closed loop were assessed using the node-splitting method (Dias et al., 2010). We utilized the ‘decomp.design’ function to evaluate homogeneity across different levels, including global network homogeneity, intra-design homogeneity, and inter-design homogeneity/consistency. P-scores were used to rank interventions and were computed exclusively from the point estimates and standard errors of the network estimates. These scores indicate the overall confidence in a treatment being better than another, based on comparisons across all competing treatments in the network (Rücker and Schwarchwarer, 2015).

We performed network meta-regression analyses with the OX group as the reference to examine the impact of the following variables: age, intervention duration, clinical staging, sample size, chemotherapy regimen, and risk of bias. If the network meta-regression variables affected the results, we additionally conducted traditional meta-regression to investigate their influence at the pairwise comparison level. We also performed sensetivity analysis by excluding high risk of bias studies. Small-study effects bias was assessed using funnel plots, Egger, Begg, and Thompson-Sharp test. We also compared the distributions of characteristics across the study arms grouped by individual agents to assess the transitivity assumption of indirect comparisons by drawing a box plot.

All statistical analysis were performed using R software.

### 2.8 GRADE certainty of evidence assessment

The certainty (quality) of evidence for direct, indirect, and network comparisons was assessed using the Grading of Recommendations Assessment, Development, and Evaluation (GRADE) approach (Puhan et al., 2014; Brignardello-Petersen et al., 2018). The certainty of the evidence was categorized into four levels: high certainty, moderate certainty, low certainty, or very low certainty. For direct comparison, the initial level of evidence certainty is high but could be downgraded if there are serious concerns related to inconsistency (heterogeneity), risk of bias, indirectness, or publication bias. We did not considered for the domain of imprecision in direct comparisons, which was taken into account for network estimates. For indirect evidence, we mainly focused on the most dominant-order loop and rated its quality based on the lowest certainty of the direct comparisons that contributed to this loop. Additionally, we considered intransitivity, which accounts for differences in effect modifiers between the two contributing direct comparisons (Chaimai et al., 2020). We based the certainty of evidence on the direct and/or indirect source that dominated the comparison and further considered incoherence and imprecision for network evidence. For risk of bias, the certainty was downgraded by one level if the combined weight of studies rated as having a high risk of bias contributed >50% to the network; for inconsistency: for heterogeneity, the certainty was downgraded by one level if the I^2^ statistic exceeded 60% in the direct comparison and the confidence intervals of the contributing studies showed substantial overlap (<50%); for indirectness: we did not proactively downgrade for indirectness, as the clinical and methodological homogeneity of the included studies (PICO elements) was a prerequisite for conducting the NMA; for publication bias: the certainty was downgraded by one level if statistical tests for funnel plot asymmetry (e.g., Egger’s test) yielded a p-value <0.05 for comparisons with sufficient (≥10) studies; for imprecision: the certainty was downgraded by one level if the 95% confidence interval (CI) of the summary estimate crossed the line of no effect or if the optimal information size (OIS) criterion was not met for a conclusive judgment. For indirect and network estimates, the initial certainty rating was based on the lower certainty of the contributing direct comparisons. We then assessed for incoherence using the node-splitting method; evidence was downgraded by one level for statistically significant incoherence (p < 0.05).

We applied a novel GRADE approach (Brignardello-Petersen et al., 2020) to categorize network meta-analysis results into three efficacy-based clusters, ranging from the most to the least effective, based on the certainty of evidence and the magnitude of effects. We chose the null effect as the decision threshold and OX as the key reference comparator. Initially, treatments were classified as either different or not different from OX. Subsequently, we further categorized them based on whether they differed from at least one treatment already identified as different from OX. This process resulted in three efficacy-based clusters: Cluster 1 (Least Effective) included drugs showing no statistical significant difference from OX, as indicated by the 95% confidence interval overlapping the null effect; Cluster 2 (Intermediately Effective) comprised drugs superior to placebo but not significantly more effective than any other treatment; and Cluster 3 (Most Effective) included drugs that demonstrated superiority over at least one drug in Cluster 2. Finally, we stratified these clusters based on evidence certainty relative to placebo: a relatively high-certainty group, where the evidence was rated as high or moderate certainty, and a relatively low-certainty group, where the evidence was rated as low or very low certainty relative to OX.

## 3 Results

### 3.1 Study selection and baseline characteristics

**FIGURE 1:**
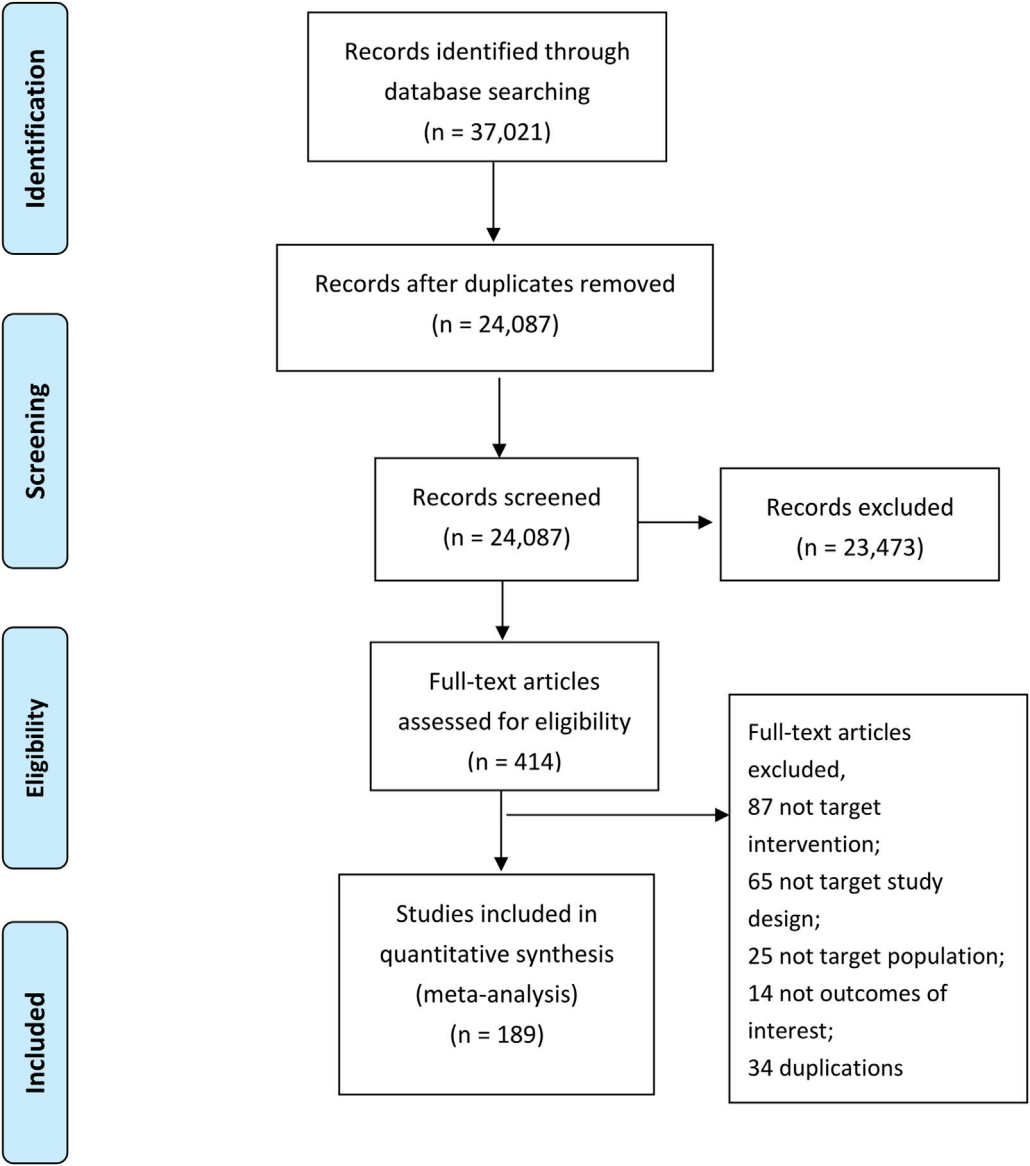
The process of study selection.

Figure 1 provides a detailed outline of the study selection process. A total of 37,021 potential studies were identified through database searches. After removing 12,934 duplicate records, 24,087 titles and abstracts were screened by teams of two independent reviewers. Following this screening, 414 studies were selected for full-text review. The detailed reasons for exclusion at the full-text screening stage are provided in Supplementary Appendix S4. Finally, 189 randomized controlled trials (RCTs) proved eligible. Supplementary Appendix S5 presents the references list of included studies.

**FIGURE 2:**
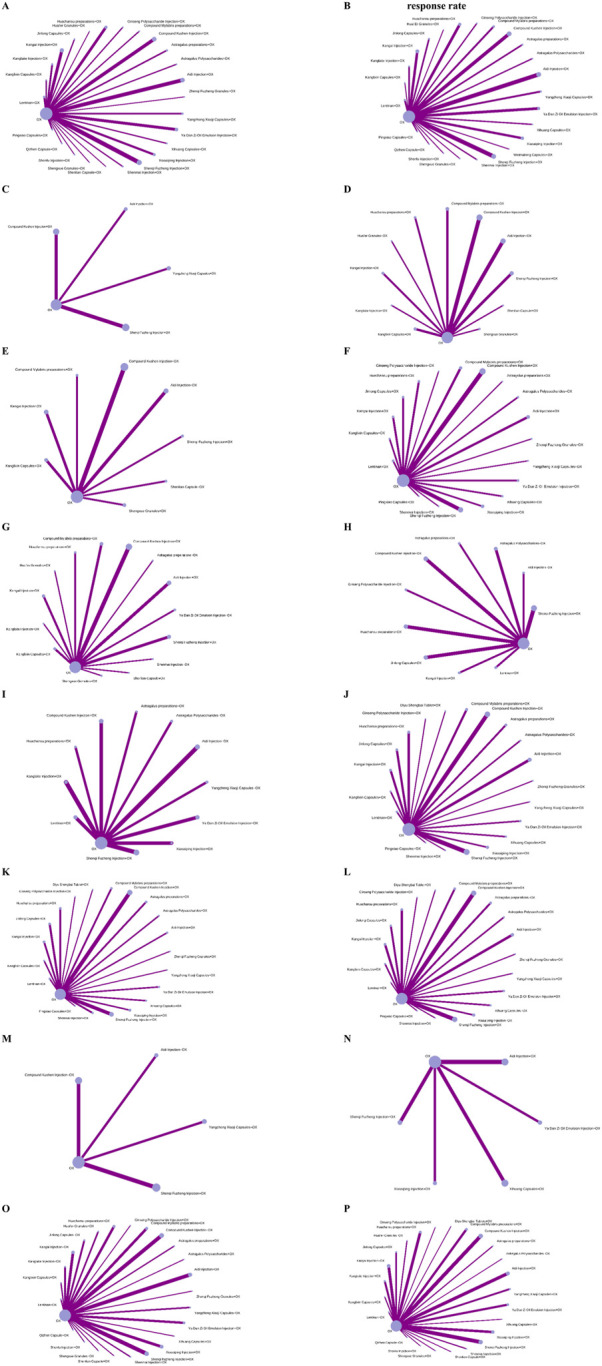
Network plots. Note: Nodes represent interventions (Size: Proportional to the number of studies evaluating the intervention); Edges represent direct comparisons (Thickness: Proportional to the number of trials for each head-to-head comparison; Gray value: Darkness indicates total sample size of direct evidence). **(A)** Disease contraol rate. **(B)** Objective response rate. **(C)** CA125. **(D)** CA199. **(E)** CA724. **(F)** CD+. **(G)** CEA. **(H)** NK cell. **(I)** Improvement rate in quality of life. **(J)** CD4+. **(K)** CD4+CD8+. **(L)** CD8+. **(M)** TCM syndrome score. **(N)** Overall survival rate. **(O)** Myelosuppression evevt. **(P)** Gastrointestinal event.

The 189 studies included a total of 15,320 participants. The mean age of participants was 55.39 years, and the median proportion of men was 62.50%. These studies were conducted across 37 regions in China. The duration of the intervention ranged from 10 to 180 days. A total of 27 CCPPs were compared in the network (Figure 2), of which, Aidi Injection combined with OX (n = 26), Compound Kushen Injection combined with OX (n = 26), and Shenqi Fuzheng Injection combined with OX (n = 32) were the most frequent treatment. Twenty studies (48.12%) reported the FOLFOX chemotherapy regimen, 54 (28.88%) reported the SOX chemotherapy regimen, 36 (19.25%) reported the XELOX chemotherapy regimen, five (2.67%) reported the EOF chemotherapy regimen, and one study (0.53%) each reported oxaliplatin and oxaliplatin-based chemotherapy regimens. Supplementary Appendix S6 shows the baseline characteristics of included studies.

### 3.2 Results of risk of bias assessment

Supplementary Appendix S7 provides detailed guidance on the risk of bias assessment, while the results of the assessment are shown in Supplementary Appendix S8. Key limitations of included studies were that not report the methods of allocation concealment, not sufficient report the implementation of blinding among patients, healthcare providers, and outcome assessor. All 187 studies were rated as having a low risk of bias in random sequence generation, incomplete data, selective outcome reporting, and other biases. Additionally, 43 studies had a low risk of bias in the blinding of outcome assessors, ten studies had a low risk of bias in the blinding of patients and healthcare providers, and five studies had a low risk of bias in allocation concealment. Overall, 48 studies (25.67%) were rated as low risk of bias.

### 3.3 Disease control rate and objective response rate

**FIGURE 3:**
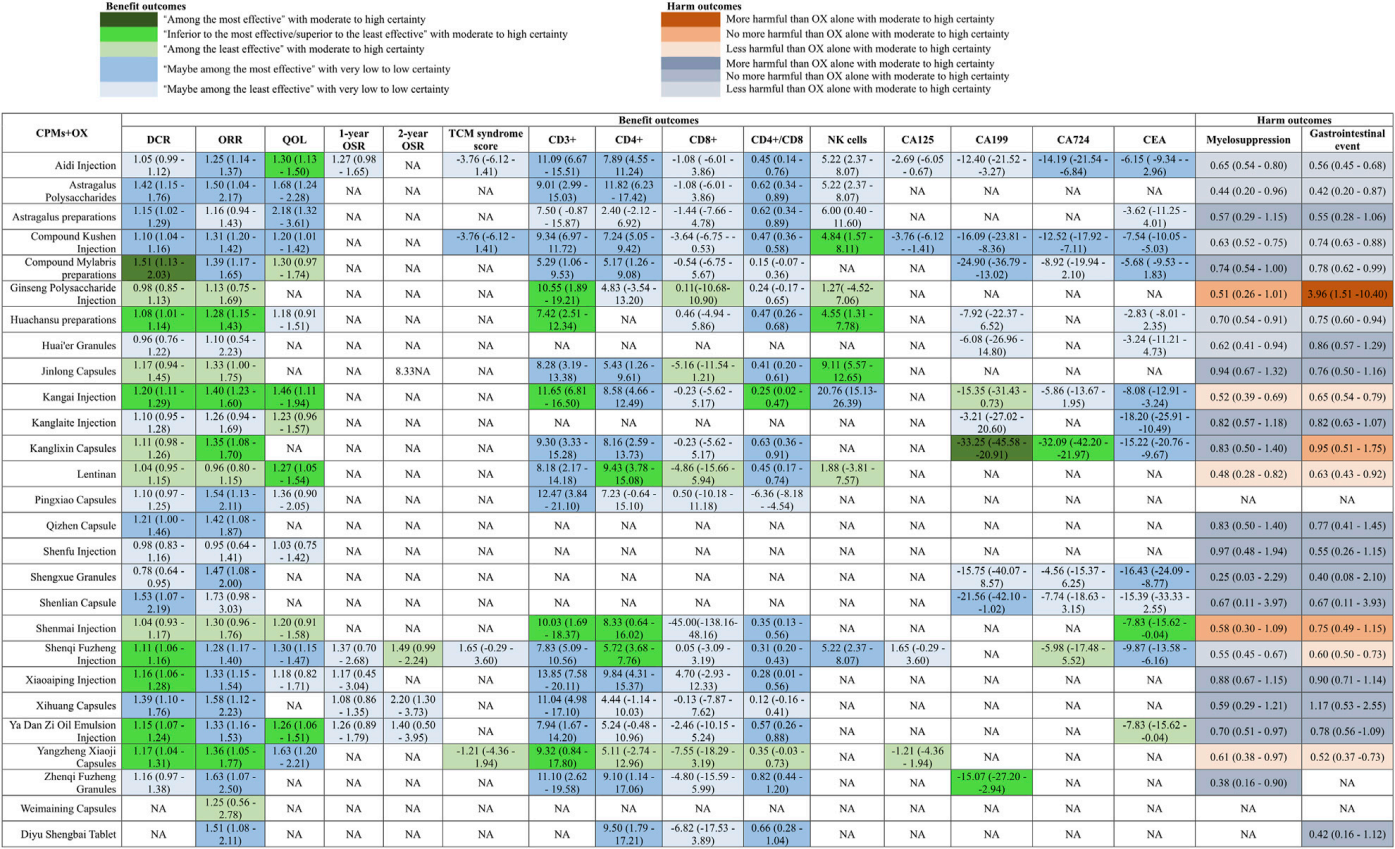
Summary of relative effects of individual drugs.

We included 127 studies with 10,128 participants in the analyses DCR and 154 studies with 11,311 participants in the analyses of ORR. Compared with OX alone, moderate to high certainty evidence showed that compound Mylabris preparations (OR 1.51, 95% CI 1.13 to 2.03, I^2^ = 0%) is among the most effective CCPPs, while, Kangai Injection (OR 1.20, 95% CI 1.11 to 1.29; I^2^ = 0%), Huachansu preparations (RR 1.08, 95% CI 1.01 to 1.14; I^2^ = 0%), Shenqi Fuzheng Injection (RR 1.11, 95% CI 1.06 to 1.16, I^2^ = 0%), Xiaoaiping Injection (RR 1.16, 95% CI 1.06 to 1.28, I^2^ = 2%), Ya Dan Zi Oil Emulsion Injection (RR 1.15, 95% CI 1.07 to 1.24, I^2^ = 15%), and Yangzheng Xiaoji Capsules (RR 1.17, 95% CI 1.04 to 1.31, I^2^ = 0%) were inferior to the most effective but superior to the least effective CCPPs in increase DCR (Figure 3 and Supplementary Appendix S9). For ORR, there were no drugs among the most effective, but, Huachansu preparations (RR 1.28, 95% CI 1.15 to 1.43, I^2^ = 0%), Kangai Injection (RR 1.40, 95% CI 1.28 to 1.77, I^2^ = 40%)), Kanglixin Capsules (RR 1.35 95% CI 1.08 to 1.70, I^2^ = NA), and Yangzheng Xiaoji Capsules (RR 1.36, 95% CI 1.05 to 1.77, I^2^ = 6%) were inferior to the most effective but superior to the least effective CCPPs in increase ORR (Figure 3 and Supplementary Appendix S9). With low or very low certainty evidence relative to OX alone, Astragalus Polysaccharides (RR 1.42, 95% CI 1.15 to 1.76, I^2^ = 37%), Astragalus preparations (RR 1.15, 95% CI 1.02 to 1.29, I^2^ = 50%), Compound Kushen Injection (RR 1.10, 95% CI 1.04 to 1.16, I^2^ = 63%), and Shenlian Capsule (RR 1.53, 95% CI 1.07 to 2.19, I^2^ = NA) might be the most effective CCPPs for increase in DCR, and Aidi Injection (RR 1.25, 95% CI 1.14 to 1.37, I^2^ = 20%), Astragalus Polysaccharides (RR 1.50, 95% CI 1.04 to 2.17, I^2^ = 0%), Compound Kushen Injection (RR 1.31, 95% CI 1.20 to 1.42, I^2^ = 54%), Pingxiao Capsules (RR 1.54, 95% CI 1.13 to 2.11, I^2^ = 0%), Qizhen Capsule (RR 1.42, 95% CI 1.08 to 1.87, I^2^ = NA), Shengxue Granules (RR 1.47, 95% CI 1.08 to 2.00, I^2^ = 0%), Shenqi Fuzheng Injection (RR 1.28, 95% CI 1.17 to 1.40, I^2^ = 0%), Xiaoaiping Injection (RR 1.58, 95% CI 1.12 to 2.23, I^2^ = 0%), Ya Dan Zi Oil Emulsion Injection (RR 1.33, 95% CI 1.16 to 1.53, I^2^ = 0%), Zhenqi Fuzheng Granules (RR 1.63, 95% CI 1.07 to 2.50, I^2^ = NA), and Diyu Shengbai Tablet (RR 1.51, 95% CI 1.08 to 2.11, I^2^ = NA) might be the most effective CCPPs for increase in ORR.

### 3.4 Improvement rate in quality of life (QoL)

Fifty-two studies with 3,658 participants involving sixteen CCPPs reported improvement rate of quality of life. With high or moderate certainty evidence relative to OX alone, there were no CCPPs might be the most effective, Aidi Injection (RR 1.30, 95% CI 1.13 to 1.50, I^2^ = 32%), Kangai Injection (RR 1.46, 95% CI 1.11 to 1.94, I^2^ = 84%), Lentinan (OR 1.27, 95% CI 1.05 to 1.54, I^2^ = 0%), and Ya Dan Zi Oil Emulsion Injection (RR 1.26, 95% CI 1.06 to 1.51, I^2^ = 0%) were CCPPs that inferior to the most effective but superior to the least effective (Figure 3 and Supplementary Appendix S9). With low or very low certainty evidence relative to OX alone, Astragalus Polysaccharides (RR 1.68, 95% CI 1.24 to 2.28, I^2^ = 84%), Astragalus preparations (RR 2.18, 95% CI 1.32 to 3.61, I^2^ = NA), Compound Kushen Injection (RR 1.20, 95% CI 1.01 to 1.42, I^2^ = 26%), Shenqi Fuzheng Injection (RR 1.30, 95% CI 1.15 to 1.47, I^2^ = 23%), and Yangzheng Xiaoji Capsules (RR 1.63, 95% CI 1.20 to 2.21, I^2^ = 94%) might be the most effective CCPPs.

### 3.5 Overall survival rate and TCM syndrome score

Nine studies with 526 participants involving five CCPPs reported 1-year overall survival rate and five studies with 457 participants involving three CCPPs reported 2-year overall survival rate. Seven studies with 545 participants involving four CCPPs reported TCM syndrome score. Low to moderate certainty evidence showed that there were no CCPPs combined with OX could increase 1-year and 2-year overall survival rate and improve TCM syndrome score (Figure 3 and Supplementary Appendix S9). However, above results are based on limited data, and the confidence intervals were wide, indicating that the power of the analyses may be relatively low.

### 3.6 Immune function indicators

Fifty-eight studies with 5,114 participants involving 19 CCPPs reported CD3^+^, seventy-two studies with 6,443 participants involving 19 CCPPs reported CD4^+,^ sixty-three studies with 5,612 participants involving 20 CCPPs reported CD8^+^, sixty-four studies with 5,776 participants involving 20 CCPPs reported CD4^+^/CD8^+^, and twenty studies with 1,477 participants involving 10 CCPPs reported NK cells. With high to moderate certainty of evidence relative to OX alone, no single CCPP was identified as the most effective for immune function indicators. However, several CCPPs demonstrated efficacy, which superior to the least effective but inferior to the most effective. Specifically, Ginseng Polysaccharide Injection (MD 10.55, 95% CI 1.89 to 19.21, I^2^ = NA), Huachansu preparations (MD 7.42, 95% CI 2.51 to 12.34, I^2^ = 88%), Kangai Injection (MD 11.65, 95% CI 6.81 to 16.50, I^2^ = 83%), Shenmai Injection (MD 10.03, 95% CI 1.69 to 18.37, I^2^ = NA), and Yangzheng Xiaoji Capsules (MD 9.32, 95% CI 0.84 to 17.80, I^2^ = NA) were superior to the least effective but inferior to the most effective for CD3^+^ improvement. For CD4^+^ improvement, Lentinan (MD 9.43, 95% CI 3.78 to 15.08, I^2^ = 73%), Shenmai Injection (MD 8.33, 95% CI 0.64 to 16.02, I^2^ = NA), and Shenqi Fuzheng Injection (MD 5.72, 95% CI 3.68 to 7.76, I^2^ = 87%) exhibited intermediate effectiveness. Kangai Injection (MD 0.25, 95% CI 0.02 to 0.47, I^2^ = 81%) was similarly ranked for CD4^+^/CD8^+^ improvement (Figure 3 and Supplementary Appendix S9). Additionally, Compound Kushen Injection (MD 4.84, 95% CI 1.57 to 8.11, I^2^ = 40%), Huachansu preparations (MD 4.55, 95% CI 1.31 to 7.78, I^2^ = 84%), and Jinlong Capsules (MD 9.11, 95% CI 5.57 to 12.65, I^2^ = 0%) were superior to the least effective but inferior to the most effective for NK cell improvement. With low or very low certainty evidence relative to OX alone, Aidi Injection (CD3^+^: MD 11.09, 95% CI 6.67 to 15.51, I^2^ = 97%; CD4^+^: MD 7.89, 95% CI 4.55 to 11.24, I^2^ = 87%; CD4^+^/CD8^+^: MD 0.45, 95% CI 0.14 to 0.76, I^2^ = 0%), Astragalus Polysaccharides (CD3^+^: MD 9.01, 95% CI 2.99 to 15.03, I^2^ = 72%; CD4^+^: MD 11.82, 95% CI 6.23 to 17.42, I^2^ = 95%; CD4^+^/CD8^+^: MD 0.62, 95% CI 0.34 to 0.89, I^2^ = 93%), Compound Kushen Injection (CD3^+^: MD 9.34, 95% CI 6.97 to 11.72, I^2^ = 95%; CD4^+^: MD 7.24, 95% CI 5.05 to 9.42, I^2^ = 98%; CD4^+^/CD8^+^: MD 0.47, 95% CI 0.36 to 0.58, I^2^ = 90%), Jinlong Capsules (CD3^+^: MD 8.28, 95% CI 3.19 to 13.38, I^2^ = 95%; CD4^+^: MD 5.43, 95% CI 1.26 to 9.61, I^2^ = 91%; CD4^+^/CD8^+^: MD 0.41, 95% CI 0.20 to 0.61, I^2^ = 94%), Kanglixin Capsules (CD3^+^: MD 9.30, 95% CI 3.33 to 15.18, I^2^ = 0%; CD4^+^: MD 8.16, 95% CI 2.59 to 13.73, I^2^ = 91%; CD4^+^/CD8^+^: MD 0.63, 95% CI 0.36 to 0.91, I^2^ = 93%), Xiaoaiping Injection (CD3^+^: MD 13.85, 95% CI 7.58 to 20.11, I^2^ = 99%; CD4^+^: MD 9.84, 95% CI 4.31 to 15.37, I^2^ = 99%; CD4^+^/CD8^+^: MD 0.28, 95% CI 0.01 to 0.56, I^2^ = 73%), Zhenqi Fuzheng Granules (CD3^+^: MD 11.10, 95% CI 2.62 to 19.58, I^2^ = NA; CD4^+^: MD 9.10, 95% CI 1.14 to 17.06, I^2^ = NA; CD4^+^/CD8^+^: MD 0.82, 95% CI 0.44 to 1.20, I^2^ = NA), and Diyu Shengbai Tablet (CD4^+^: MD 9.50, 95% CI 1.79 to 17.21, I^2^ = NA; CD4^+^/CD8^+^: MD 0.66, 95% CI 0.28 to 1.04, I^2^ = NA) might be the most effective CCPPs for improvement of immune function indicators (Figure 3 and Supplementary Appendix S9).

### 3.7 Tumor markers

Seven studies with 545 participants involving four CCPPs reported CA125, thirty-three studies with 2,718 involving 11 CCPPs reported CA199, sixteen studies with 1,325 participants involving eight CCPPs reported CA724, forty-one studies with 3,392 participants involving 14 CCPPs reported CEA. With high to moderate certainty of evidence relative to OX alone, Kanglixin Capsules was identified as the most effective for reduction of CA199 and superior to the least effective but inferior to the most effective for reduction of CA724, and Zhenqi Fuzheng Granules and Shenmai Injection were superior to the least effective but inferior to the most effective for CA199 and CEA respectively (Figure 3 and Supplementary Appendix S9). With low or very low certainty evidence relative to OX alone, Aidi Injection (CA724: MD -14.19, 95% CI -21.54 to −6.84, I^2^ = 97%; CEA: MD -6.15, 95% CI -9.34 to −2.96, I^2^ = 95%), Compound Kushen Injection (CA724: MD -12.52, 95% CI -17.92 to −7.11, I^2^ = 71%; CEA: MD -7.54, 95% CI -10.05 to −5.03, I^2^ = 95%; CA125: MD -3.76, 95%CI -6.12 to −1.41, I^2^ = 11%), and Compound Mylabris preparations (CA724: MD -24.90, 95% CI -36.79 to −13.02, I^2^ = NA; CEA: MD -5.68, 95% CI -9.53 to −1.83, I^2^ = 79%) might be the most effective CCPPs for reduction of tumor markers (Figure 3 and Supplementary Appendix S9).

### 3.8 Adverse event

One hundred and twenty-one studies with 9,689 participants involving 19 CCPPs reported myelosuppression event, one hundred and forty-one studies with 11,257 participants involving 24 CCPPs reported gastrointestinal event. With high to moderate certainty of evidence relative to OX alone, Kangai Injection (Myelosuppression event: RR 0.52, 95% CI 0.39 to 0.69, I^2^ = 0%; Gastrointestinal event: RR 0.65, 95% CI 0.54 to 0.79, I^2^ = 27%), Lentinan (Myelosuppression event: RR 0.48, 95% CI 0.28 to 0.82, I^2^ = 0%; Gastrointestinal event: RR 0.63, 95% CI 0.43 to 0.92, I^2^ = 67%), and Yangzheng Xiaoji Capsules (Myelosuppression event: RR 0.61, 95% CI 0.38 to 0.97, I^2^ = 0%; Gastrointestinal event: RR 0.52, 95% CI 0.37 to 0.73, I^2^ = 0%) could reduce risk of myelosuppression event and gastrointestinal event (Figure 3 and Supplementary Appendix S9). Low to very low certainty evidence showed that Aidi Injection (Myelosuppression event: RR 0.65, 95% CI 0.54 to 0.80, I^2^ = 72%; Gastrointestinal event: RR 0.56, 95% CI 0.45 to 0.68, I^2^ = 19%), Astragalus Polysaccharides (Myelosuppression event: RR 0.44, 95% CI 0.20 to 0.96, I^2^ = NA; Gastrointestinal event: RR 0.42, 95% CI 0.20 to 0.87, I^2^ = NA), Compound Kushen Injection (Myelosuppression event: RR 0.63, 95% CI 0.52 to 0.75, I^2^ = 0%; Gastrointestinal event: RR 0.74, 95% CI 0.63 to 0.88, I^2^ = 51%), and Huachansu preparations (Myelosuppression event: RR 0.70, 95% CI 0.54 to 0.91, I^2^ = 83%; Gastrointestinal event: RR 0.75, 95% CI 0.60 to 0.94, I^2^ = 15%) could reduce risk of myelosuppression event and gastrointestinal event when compared with OX alone (Figure 3 and Supplementary Appendix S9).

### 3.9 Other analyses

The results of sensitivity analysis for fixed-effect model are presented in Supplementary Appendix S11, The results of sensitivity analysis by excluding high risk of bias studies are presented in Supplementary Appendix S12, 13 shows the results of publication bias, and Supplementary Appendix S14 shows the results of meta-regression, Supplementary Appendix S15 presents the results of nodesplit, Supplementary Appendix S16 shows the results of GRADE assessment, Supplementary Appendix S16 shows the summary of ranking results, Supplementary Appendix S18 presents the results of intransitivity assessment. Fixed-effect model network meta-analysis showed the consistent results with random-effect model. And results of sensitivity analysis by excluding high risk bias studies showed the results are consistent with primary analysis. DCR, CEA, and improvement rate in quality of life showed publication bias in both Egger, Begg, and Thompson-Sharp test.

Results of meta-regression suggested that neither clinical staging, treat duration, risk of bias, age, and chemotherapy regimen could influence DCR, ORR, improvement rate in QoL, survival rate, TCM syndrome score, immune function indicator, tumor markers, and AEs, except for clinical staging for CA199 and treatment duration for ORR.

## 4 Discussion

### 4.1 Principal finding

This network meta-analysis comprehensively summarizes the effectiveness and safety of currently available CCPPs as adjuvant treatments for GC. Moderate-to high-certainty evidence suggests that Kangai Injection, Huachansu preparations, Xiaoaiping Injection, and Yangzheng Xiaoji Capsules are among the most effective CCPPs for improving DCR and ORR, while Kangai Injection, Aidi Injection, Lentinan, and Ya Dan Zi Oil Emulsion Injection demonstrate notable efficacy in enhancing QoL improvement rate in patients with GC. Regarding immune function improvement, the most effective CCPPs include Shenmai Injection (enhancing CD3^+^ and CD4^+^ levels), Compound Kushen Injection (boosting NK cell activity), Ginseng Polysaccharide Injection (enhancing CD3^+^ levels), Huachansu preparations (increasing CD3^+^ and NK cell levels), Kangai Injection (enhancing CD3^+^ levels and the CD4^+^/CD8^+^ ratio), Lentinan (promoting CD4^+^ levels), Shenqi Fuzheng Injection (improving CD4^+^ levels), and Yangzheng Xiaoji Capsules (enhancing CD3^+^ levels). Additionally, Kanglixin Capsules were identified as among the most effective CCPPs for reducing tumor markers (CA199, CA724). Furthermore, moderate-to high-certainty evidence suggests that Kangai Injection, Lentinan, and Yangzheng Xiaoji Capsules may contribute to a lower incidence of myelosuppression and gastrointestinal adverse events compared to OX alone. To assess the potential influence of effect modifiers we performed network meta-regression analyses, and the results showed that these variations were largely unexplained by clinical covariates except advanced stage reducing CA199 response and longer treatment enhancing ORR.

### 4.2 Strengths and limitations

The strengths of this network meta-analysis include its comprehensiveness, representing the most extensive systematic review and network meta-analysis to date that establishes a clear hierarchy and comparative effectiveness of all currently available CCPPs for the treatment of GC. This study was conducted in strict accordance with the PRISMA guidelines and incorporated rigorous and contemporary methodological approaches for network meta-analysis and GRADE assessment. By synthesizing high-quality evidence, the findings provide robust support for clinical decision-making and contribute to the optimization of personalized CCPP treatment strategies for GC.

Our systematic review also has some limitations. First, some insufficient information for risk of bias assessment, such as a lack of adequate details about randomization and blinding procedures, which may affect the methodological robustness of the included evidence. Second, this review does not specifically investigate AEs, highlighting the need for future studies to conduct a more in-depth exploration of specific AEs associated with different interventions. Thirdly, for some patient-important outcomes (OSR), only a very limited number of studies have reported on specific Chinese CCPPs. This underscores the need for more high-quality RCTs in the future to better evaluate these critical outcomes and provide stronger evidence. Third, for survival outcomes (1-year and 2-year OS), our study was based on limited data, and thus, the power of the analyses may be relatively low and the results may not be sufficiently informative. More trials are needed to investigate the efficacy and safety of CCPPs for survival outcomes. Finally, we only searched Chinese and English publications, which may be led to potential selection bias and limit generalizability. Although, the impact on our findings is likely minimal given the concentration of research in Chinese/English. Future studies should, where feasible, incorporate searches of literature in other relevant languages and trial registries from regions with high gastric cancer incidence.

### 4.3 Compared with other studies

This network meta-analysis included more studies than many existing reviews on CCPPs for GC. This allowed us to integrate the strengths of various CCPP approaches and draw more nuanced and precise conclusions. Tan et al. (2022) systematic review found that Chinese herbal medicine combined with OX chemotherapy could increase the ORR by 35% and DCR by 12% which was consistent with our finding. Li (2019) study showed that Kangai Injection and Shenqi Fuzheng Injection combined with SOX could increase DCR, ORR, and improve QoL rate, which were also found in our review. While, our study also did not support some existing findings, for example, Wu and colleagues (Wu et al., 2019) found that Xiaoaiping Injection combined with SOX regimen could not improve ORR or DCR, but it did enhance the KPS improvement rate. However, results from our review showed Xiaoaiping Injection combied with OX are among the most effective CCPPs for improving DCR and ORR but not QoL improvement rate. Results from Li et al.‘s study (Li, 2019) suggested that Aidi Injection and Kanglaite Injection combined with OX could increase DCR and ORR, and could reduce the incidence of leukopenia and peripheral neurotoxicity, which were inconsistent with our review. The main reason for the inconsistency may be that our network meta-analysis included more latest relevant studies and adopted different statistical analysis methods. Bu and colleagues also conducted a NMA to assess the effectiveness and safety of Chinese herbal injections combined with SOX chemotherapy regimens for advanced GC, they only focused on the Chinese herbal injections and advanced GC, which were different from our review. Additionally, we conducted NMA within the frequentist framework using the “netmeta” package, while Bu and colleagues within Bayesian framework using the “gemtc” package. We also applied a novel GRADE approach to categorize network meta-analysis results into three efficacy-based clusters, ranging from the most to the least effective, based on the certainty of evidence and the magnitude of effects, while Bu and colleagues interpreted findings primarily through SUCRA rankings.

### 4.4 Future research directions

The results of our network meta-analysis may provide important evidence into the role of CCPPs as adjuvant treatments in GC chemotherapy. We found some CCPPs that may increase immune function, tumor response, and lessen the toxicity associated with chemotherapy. Despite more and more evidence suggested that TCM can improve chemotherapy efficacy, lower toxicity, and alter the tumor microenvironment, there are still a lot of important problems need to be addressed. To increase the quality of research related to TCM and to provide more solid research foundation for TCM adjuvant treatment of GC, future research should focus on the following aspects: (1) Current RCTs on TCM had several important problems, including small sample sizes, study design biases, heterogeneity in treatment protocols, and variations in outcome assessments. To improve the quality of clinical evidence, future studies should prioritize multi-center, double-blind RCTs according to standardized methodologies. (2) Given the complex nature of TCM interventions and the importance of personalized treatment, real-word-study (RES) based on large-scale healthcare databases should be conducted. These studies can help assess the long-term safety and efficacy of TCM in diverse patient populations, addressing gaps that conventional RCTs may not fully capture.

### 4.5 Implications for clinical practice and guideline development

The findings of this NMA have direct and substantial implications for the adjuvant treatment of GC and the future updating of clinical practice guidelines. First, our analysis provides clinicians with a evidence-based hierarchy of CCPP options for specific therapeutic goals. For instance, to enhance tumor response, Kangai Injection, Huachansu preparations, Xiaoaiping Injection, and Yangzheng Xiaoji Capsules should be prioritized for consideration. For patients where the primary goal is to mitigate the detrimental effects of chemotherapy on quality of life, Kangai Injection, Aidi Injection, Lentinan, and Ya Dan Zi Oil Emulsion Injection emerge as the most compelling choices. This allows for a more personalized and goal-oriented approach to integrative oncology care. Second, Currently, many international oncology guidelines lack specific recommendations on the use of TCM due to a perceived lack of robust comparative effectiveness data. This study addresses that gap by providing moderate-to high-certainty evidence from a comprehensive comparative analysis. Specifically, our results strongly support the inclusion of certain CCPPs into supportive care sections of GC treatment guidelines.

## 5 Conclusion

Our study showed that no single CCPP is universally superior for all outcomes in GC adjuvant therapy. Compound Mylabris Preparation shows highest effectiveness for DCR, while Kangai Injection demonstrates the most robust profile due to its broad intermediate efficacy across multiple outcomes as well as statistical significant benefits in reducing AEs, with moderate to high certainty evidence. Furthermore, Huachansu preparations, Kanglixin Capsules, Shenmai Injection, Xiaoaiping Injection, Ya Dan Zi Oil Emulsion Injection, and Yangzheng Xiaoji Capsules also showed favorable efficacy in improving DCR and ORR, enhancing immune parameters, and reducing tumor markers, with high to moderate certainty evidence. The choice of adjuvant CCPP should be individualized based on specific therapeutic priorities, balancing efficacy and safety.

## Data Availability

The original contributions presented in the study are included in the article/Supplementary Material, further inquiries can be directed to the corresponding authors.

## Abbreviations

AEs, adverse events; CCPPs, commercial Chinese polyherbal preparations; CEA, carcinoembryonic antigen; CA125, carbohydrate antigen 125; CA199, carbohydrate antigen 199; CA724, carbohydrate antigen 724; DCR, disease control rate; GC Gastric cancer; NK cells, natural killer; OX, oxaliplatin-based chemotherapy; ORR, objective response rate; QoL, improvement rate in quality of life; TCM, Traditional Chinese Medicine.
