# Supplementary material for: Efficacy and safety of commercial Chinese polyherbal preparation combined with oxaliplatin-based chemotherapy for gastric cancer: a systematic review and network meta-analysis
Source: Front Pharmacol. 2025 Sep 9;16:1645079. doi: 10.3389/fphar.2025.1645079 (PMC12455072; doi:10.3389/fphar.2025.1645079)
Supplement: Supplementary file 1 [file Supplementaryfile1.zip › Appendix 14-19.docx]

**Efficacy and Safety of Commercial Chinese Polyherbal Preparation Combined with Oxaliplatin-Based Chemotherapy for Gastric Cancer: A Systematic Review and Network Meta-analysis**

# **Appendix 16 Results of GRADE assessment**

1. Disease control rate

| Arm_1 | Arm_2 | No_of_study | Sample_size | I2 | Direct_estimate | ROB | Inconsistency | Indirectness | Publication_bias | Direct_rating_without_imprecision | Indirect_estimate | Certainty_of_evidence_for_arm1 | Certainty_of_evidence_for_arm2 | Intransitivity | Indirect_rating_without_imprecision | Network_meta_analysis | Higher_rating_of_direct_and_indirect_without_imprecision | Incoherence | NMA_Imprecision | Final_network_rating | Final_rating_reason |
| --- | --- | --- | --- | --- | --- | --- | --- | --- | --- | --- | --- | --- | --- | --- | --- | --- | --- | --- | --- | --- | --- |
| Aidi Injection+OX | Astragalus Polysaccharides+OX | 0 | . | . | . |  |  |  |  |  | 0.74 [0.59; 0.92] | Low | Low | Not serious | Low | 0.74 [0.59; 0.92] | Low | . | Serious | Very low | NMA estimate was used because incoherence was not available. Imprecision was rated as Serious. |
| Aidi Injection+OX | Astragalus preparations+OX | 0 | . | . | . |  |  |  |  |  | 0.92 [0.80; 1.05] | Low | Low | Not serious | Low | 0.92 [0.80; 1.05] | Low | . | Serious | Very low | NMA estimate was used because incoherence was not available. Imprecision was rated as Serious. |
| Aidi Injection+OX | Compound Kushen Injection+OX | 0 | . | . | . |  |  |  |  |  | 0.96 [0.88; 1.04] | Low | Very low | Not serious | Very low | 0.96 [0.88; 1.04] | Very low | . | Serious | Very low | NMA estimate was used because incoherence was not available. Imprecision was rated as Serious. |
| Aidi Injection+OX | Compound Mylabris preparations+OX | 0 | . | . | . |  |  |  |  |  | 0.70 [0.51; 0.94] | Low | Moderate | Not serious | Low | 0.70 [0.51; 0.94] | Low | . | Serious | Very low | NMA estimate was used because incoherence was not available. Imprecision was rated as Serious. |
| Aidi Injection+OX | Ginseng Polysaccharide Injection+OX | 0 | . | . | . |  |  |  |  |  | 1.07 [0.92; 1.25] | Low | High | Not serious | Low | 1.07 [0.92; 1.25] | Low | . | Serious | Very low | NMA estimate was used because incoherence was not available. Imprecision was rated as Serious. |
| Aidi Injection+OX | Huachansu preparations+OX | 0 | . | . | . |  |  |  |  |  | 0.98 [0.90; 1.07] | Low | High | Not serious | Low | 0.98 [0.90; 1.07] | Low | . | Serious | Very low | NMA estimate was used because incoherence was not available. Imprecision was rated as Serious. |
| Aidi Injection+OX | Huai'er Granules+OX | 0 | . | . | . |  |  |  |  |  | 1.09 [0.86; 1.40] | Low | Low | Not serious | Low | 1.09 [0.86; 1.40] | Low | . | Serious | Very low | NMA estimate was used because incoherence was not available. Imprecision was rated as Serious. |
| Aidi Injection+OX | Jinlong Capsules+OX | 0 | . | . | . |  |  |  |  |  | 0.90 [0.72; 1.13] | Low | High | Not serious | Low | 0.90 [0.72; 1.13] | Low | . | Serious | Very low | NMA estimate was used because incoherence was not available. Imprecision was rated as Serious. |
| Aidi Injection+OX | Kangai Injection+OX | 0 | . | . | . |  |  |  |  |  | 0.88 [0.80; 0.97] | Low | High | Not serious | Low | 0.88 [0.80; 0.97] | Low | . | Serious | Very low | NMA estimate was used because incoherence was not available. Imprecision was rated as Serious. |
| Aidi Injection+OX | Kanglaite Injection+OX | 0 | . | . | . |  |  |  |  |  | 0.96 [0.81; 1.12] | Low | Moderate | Not serious | Low | 0.96 [0.81; 1.12] | Low | . | Serious | Very low | NMA estimate was used because incoherence was not available. Imprecision was rated as Serious. |
| Aidi Injection+OX | Kanglixin Capsules+OX | 0 | . | . | . |  |  |  |  |  | 0.95 [0.82; 1.09] | Low | High | Not serious | Low | 0.95 [0.82; 1.09] | Low | . | Serious | Very low | NMA estimate was used because incoherence was not available. Imprecision was rated as Serious. |
| Aidi Injection+OX | Lentinan+OX | 0 | . | . | . |  |  |  |  |  | 1.01 [0.90; 1.13] | Low | High | Not serious | Low | 1.01 [0.90; 1.13] | Low | . | Serious | Very low | NMA estimate was used because incoherence was not available. Imprecision was rated as Serious. |
| Aidi Injection+OX | OX | 17 | 1250 | 39.0% | 1.05 [0.99; 1.12] | Very serious | Not serious | Not serious | Not serious | Low | . |  |  |  |  | 1.05 [0.99; 1.12] | Low | . | Serious | Very low | NMA estimate was used because incoherence was not available. Imprecision was rated as Serious. |
| Aidi Injection+OX | Pingxiao Capsules+OX | 0 | . | . | . |  |  |  |  |  | 0.95 [0.83; 1.10] | Low | Low | Not serious | Low | 0.95 [0.83; 1.10] | Low | . | Serious | Very low | NMA estimate was used because incoherence was not available. Imprecision was rated as Serious. |
| Aidi Injection+OX | Qizhen Capsule+OX | 0 | . | . | . |  |  |  |  |  | 0.87 [0.72; 1.06] | Low | Low | Not serious | Low | 0.87 [0.72; 1.06] | Low | . | Serious | Very low | NMA estimate was used because incoherence was not available. Imprecision was rated as Serious. |
| Aidi Injection+OX | Shenfu Injection+OX | 0 | . | . | . |  |  |  |  |  | 1.07 [0.90; 1.28] | Low | Low | Not serious | Low | 1.07 [0.90; 1.28] | Low | . | Serious | Very low | NMA estimate was used because incoherence was not available. Imprecision was rated as Serious. |
| Aidi Injection+OX | Shengxue Granules+OX | 0 | . | . | . |  |  |  |  |  | 1.35 [1.10; 1.67] | Low | Low | Not serious | Low | 1.35 [1.10; 1.67] | Low | . | Not serious | Low | NMA estimate was used because incoherence was not available. Imprecision was rated as Serious. |
| Aidi Injection+OX | Shenlian Capsule+OX | 0 | . | . | . |  |  |  |  |  | 0.69 [0.48; 0.99] | Low | Low | Not serious | Low | 0.69 [0.48; 0.99] | Low | . | Serious | Very low | NMA estimate was used because incoherence was not available. Imprecision was rated as Serious. |
| Aidi Injection+OX | Shenmai Injection+OX | 0 | . | . | . |  |  |  |  |  | 1.01 [0.89; 1.15] | Low | High | Not serious | Low | 1.01 [0.89; 1.15] | Low | . | Serious | Very low | NMA estimate was used because incoherence was not available. Imprecision was rated as Serious. |
| Aidi Injection+OX | Shenqi Fuzheng Injection+OX | 0 | . | . | . |  |  |  |  |  | 0.95 [0.88; 1.02] | Low | Moderate | Not serious | Low | 0.95 [0.88; 1.02] | Low | . | Serious | Very low | NMA estimate was used because incoherence was not available. Imprecision was rated as Serious. |
| Aidi Injection+OX | Xiaoaiping Injection+OX | 0 | . | . | . |  |  |  |  |  | 0.91 [0.81; 1.01] | Low | Moderate | Not serious | Low | 0.91 [0.81; 1.01] | Low | . | Serious | Very low | NMA estimate was used because incoherence was not available. Imprecision was rated as Serious. |
| Aidi Injection+OX | Xihuang Capsules+OX | 0 | . | . | . |  |  |  |  |  | 0.76 [0.59; 0.96] | Low | Low | Not serious | Low | 0.76 [0.59; 0.96] | Low | . | Serious | Very low | NMA estimate was used because incoherence was not available. Imprecision was rated as Serious. |
| Aidi Injection+OX | Ya Dan Zi Oil Emulsion Injection+OX | 0 | . | . | . |  |  |  |  |  | 0.92 [0.83; 1.01] | Low | Moderate | Not serious | Low | 0.92 [0.83; 1.01] | Low | . | Serious | Very low | NMA estimate was used because incoherence was not available. Imprecision was rated as Serious. |
| Aidi Injection+OX | Yangzheng Xiaoji Capsules+OX | 0 | . | . | . |  |  |  |  |  | 0.90 [0.79; 1.03] | Low | High | Not serious | Low | 0.90 [0.79; 1.03] | Low | . | Serious | Very low | NMA estimate was used because incoherence was not available. Imprecision was rated as Serious. |
| Aidi Injection+OX | Zhenqi Fuzheng Granules+OX | 0 | . | . | . |  |  |  |  |  | 0.91 [0.75; 1.10] | Low | Low | Not serious | Low | 0.91 [0.75; 1.10] | Low | . | Serious | Very low | NMA estimate was used because incoherence was not available. Imprecision was rated as Serious. |
| Astragalus Polysaccharides+OX | Astragalus preparations+OX | 0 | . | . | . |  |  |  |  |  | 1.24 [0.97; 1.58] | Low | Low | Not serious | Low | 1.24 [0.97; 1.58] | Low | . | Serious | Very low | NMA estimate was used because incoherence was not available. Imprecision was rated as Serious. |
| Astragalus Polysaccharides+OX | Compound Kushen Injection+OX | 0 | . | . | . |  |  |  |  |  | 1.30 [1.04; 1.62] | Low | Very low | Not serious | Very low | 1.30 [1.04; 1.62] | Very low | . | Not serious | Low | NMA estimate was used because incoherence was not available. Imprecision was rated as Serious. |
| Astragalus Polysaccharides+OX | Compound Mylabris preparations+OX | 0 | . | . | . |  |  |  |  |  | 0.94 [0.65; 1.36] | Low | Moderate | Not serious | Low | 0.94 [0.65; 1.36] | Low | . | Serious | Very low | NMA estimate was used because incoherence was not available. Imprecision was rated as Serious. |
| Astragalus Polysaccharides+OX | Ginseng Polysaccharide Injection+OX | 0 | . | . | . |  |  |  |  |  | 1.45 [1.13; 1.88] | Low | High | Not serious | Low | 1.45 [1.13; 1.88] | Low | . | Not serious | Low | NMA estimate was used because incoherence was not available. Imprecision was rated as Serious. |
| Astragalus Polysaccharides+OX | Huachansu preparations+OX | 0 | . | . | . |  |  |  |  |  | 1.32 [1.06; 1.65] | Low | High | Not serious | Low | 1.32 [1.06; 1.65] | Low | . | Not serious | Low | NMA estimate was used because incoherence was not available. Imprecision was rated as Serious. |
| Astragalus Polysaccharides+OX | Huai'er Granules+OX | 0 | . | . | . |  |  |  |  |  | 1.48 [1.08; 2.04] | Low | Low | Not serious | Low | 1.48 [1.08; 2.04] | Low | . | Not serious | Low | NMA estimate was used because incoherence was not available. Imprecision was rated as Serious. |
| Astragalus Polysaccharides+OX | Jinlong Capsules+OX | 0 | . | . | . |  |  |  |  |  | 1.22 [0.90; 1.65] | Low | High | Not serious | Low | 1.22 [0.90; 1.65] | Low | . | Serious | Very low | NMA estimate was used because incoherence was not available. Imprecision was rated as Serious. |
| Astragalus Polysaccharides+OX | Kangai Injection+OX | 0 | . | . | . |  |  |  |  |  | 1.19 [0.95; 1.49] | Low | High | Not serious | Low | 1.19 [0.95; 1.49] | Low | . | Serious | Very low | NMA estimate was used because incoherence was not available. Imprecision was rated as Serious. |
| Astragalus Polysaccharides+OX | Kanglaite Injection+OX | 0 | . | . | . |  |  |  |  |  | 1.29 [1.00; 1.68] | Low | Moderate | Not serious | Low | 1.29 [1.00; 1.68] | Low | . | Not serious | Low | NMA estimate was used because incoherence was not available. Imprecision was rated as Serious. |
| Astragalus Polysaccharides+OX | Kanglixin Capsules+OX | 0 | . | . | . |  |  |  |  |  | 1.28 [1.00; 1.64] | Low | High | Not serious | Low | 1.28 [1.00; 1.64] | Low | . | Not serious | Low | NMA estimate was used because incoherence was not available. Imprecision was rated as Serious. |
| Astragalus Polysaccharides+OX | Lentinan+OX | 0 | . | . | . |  |  |  |  |  | 1.36 [1.08; 1.73] | Low | High | Not serious | Low | 1.36 [1.08; 1.73] | Low | . | Not serious | Low | NMA estimate was used because incoherence was not available. Imprecision was rated as Serious. |
| Astragalus Polysaccharides+OX | OX | 2 | 151 | 37.5% | 1.42 [1.15; 1.76] | Very serious | Not serious | Not serious | Undetected | Low | . |  |  |  |  | 1.42 [1.15; 1.76] | Low | . | Not serious | Low | NMA estimate was used because incoherence was not available. Imprecision was rated as Serious. |
| Astragalus Polysaccharides+OX | Pingxiao Capsules+OX | 0 | . | . | . |  |  |  |  |  | 1.29 [1.01; 1.65] | Low | Low | Not serious | Low | 1.29 [1.01; 1.65] | Low | . | Not serious | Low | NMA estimate was used because incoherence was not available. Imprecision was rated as Serious. |
| Astragalus Polysaccharides+OX | Qizhen Capsule+OX | 0 | . | . | . |  |  |  |  |  | 1.18 [0.89; 1.57] | Low | Low | Not serious | Low | 1.18 [0.89; 1.57] | Low | . | Serious | Very low | NMA estimate was used because incoherence was not available. Imprecision was rated as Serious. |
| Astragalus Polysaccharides+OX | Shenfu Injection+OX | 0 | . | . | . |  |  |  |  |  | 1.45 [1.11; 1.91] | Low | Low | Not serious | Low | 1.45 [1.11; 1.91] | Low | . | Not serious | Low | NMA estimate was used because incoherence was not available. Imprecision was rated as Serious. |
| Astragalus Polysaccharides+OX | Shengxue Granules+OX | 0 | . | . | . |  |  |  |  |  | 1.83 [1.37; 2.46] | Low | Low | Not serious | Low | 1.83 [1.37; 2.46] | Low | . | Not serious | Low | NMA estimate was used because incoherence was not available. Imprecision was rated as Serious. |
| Astragalus Polysaccharides+OX | Shenlian Capsule+OX | 0 | . | . | . |  |  |  |  |  | 0.93 [0.61; 1.41] | Low | Low | Not serious | Low | 0.93 [0.61; 1.41] | Low | . | Serious | Very low | NMA estimate was used because incoherence was not available. Imprecision was rated as Serious. |
| Astragalus Polysaccharides+OX | Shenmai Injection+OX | 0 | . | . | . |  |  |  |  |  | 1.36 [1.07; 1.74] | Low | High | Not serious | Low | 1.36 [1.07; 1.74] | Low | . | Not serious | Low | NMA estimate was used because incoherence was not available. Imprecision was rated as Serious. |
| Astragalus Polysaccharides+OX | Shenqi Fuzheng Injection+OX | 0 | . | . | . |  |  |  |  |  | 1.28 [1.03; 1.60] | Low | Moderate | Not serious | Low | 1.28 [1.03; 1.60] | Low | . | Not serious | Low | NMA estimate was used because incoherence was not available. Imprecision was rated as Serious. |
| Astragalus Polysaccharides+OX | Xiaoaiping Injection+OX | 0 | . | . | . |  |  |  |  |  | 1.23 [0.97; 1.55] | Low | Moderate | Not serious | Low | 1.23 [0.97; 1.55] | Low | . | Serious | Very low | NMA estimate was used because incoherence was not available. Imprecision was rated as Serious. |
| Astragalus Polysaccharides+OX | Xihuang Capsules+OX | 0 | . | . | . |  |  |  |  |  | 1.02 [0.75; 1.41] | Low | Low | Not serious | Low | 1.02 [0.75; 1.41] | Low | . | Serious | Very low | NMA estimate was used because incoherence was not available. Imprecision was rated as Serious. |
| Astragalus Polysaccharides+OX | Ya Dan Zi Oil Emulsion Injection+OX | 0 | . | . | . |  |  |  |  |  | 1.24 [0.99; 1.55] | Low | Moderate | Not serious | Low | 1.24 [0.99; 1.55] | Low | . | Serious | Very low | NMA estimate was used because incoherence was not available. Imprecision was rated as Serious. |
| Astragalus Polysaccharides+OX | Yangzheng Xiaoji Capsules+OX | 0 | . | . | . |  |  |  |  |  | 1.22 [0.96; 1.56] | Low | High | Not serious | Low | 1.22 [0.96; 1.56] | Low | . | Serious | Very low | NMA estimate was used because incoherence was not available. Imprecision was rated as Serious. |
| Astragalus Polysaccharides+OX | Zhenqi Fuzheng Granules+OX | 0 | . | . | . |  |  |  |  |  | 1.23 [0.93; 1.62] | Low | Low | Not serious | Low | 1.23 [0.93; 1.62] | Low | . | Serious | Very low | NMA estimate was used because incoherence was not available. Imprecision was rated as Serious. |
| Astragalus preparations+OX | Compound Kushen Injection+OX | 0 | . | . | . |  |  |  |  |  | 1.05 [0.92; 1.19] | Low | Very low | Not serious | Very low | 1.05 [0.92; 1.19] | Very low | . | Serious | Very low | NMA estimate was used because incoherence was not available. Imprecision was rated as Serious. |
| Astragalus preparations+OX | Compound Mylabris preparations+OX | 0 | . | . | . |  |  |  |  |  | 0.76 [0.55; 1.04] | Low | Moderate | Not serious | Low | 0.76 [0.55; 1.04] | Low | . | Serious | Very low | NMA estimate was used because incoherence was not available. Imprecision was rated as Serious. |
| Astragalus preparations+OX | Ginseng Polysaccharide Injection+OX | 0 | . | . | . |  |  |  |  |  | 1.17 [0.97; 1.41] | Low | High | Not serious | Low | 1.17 [0.97; 1.41] | Low | . | Serious | Very low | NMA estimate was used because incoherence was not available. Imprecision was rated as Serious. |
| Astragalus preparations+OX | Huachansu preparations+OX | 0 | . | . | . |  |  |  |  |  | 1.07 [0.94; 1.22] | Low | High | Not serious | Low | 1.07 [0.94; 1.22] | Low | . | Serious | Very low | NMA estimate was used because incoherence was not available. Imprecision was rated as Serious. |
| Astragalus preparations+OX | Huai'er Granules+OX | 0 | . | . | . |  |  |  |  |  | 1.19 [0.92; 1.55] | Low | Low | Not serious | Low | 1.19 [0.92; 1.55] | Low | . | Serious | Very low | NMA estimate was used because incoherence was not available. Imprecision was rated as Serious. |
| Astragalus preparations+OX | Jinlong Capsules+OX | 0 | . | . | . |  |  |  |  |  | 0.98 [0.77; 1.26] | Low | High | Not serious | Low | 0.98 [0.77; 1.26] | Low | . | Serious | Very low | NMA estimate was used because incoherence was not available. Imprecision was rated as Serious. |
| Astragalus preparations+OX | Kangai Injection+OX | 0 | . | . | . |  |  |  |  |  | 0.96 [0.83; 1.10] | Low | High | Not serious | Low | 0.96 [0.83; 1.10] | Low | . | Serious | Very low | NMA estimate was used because incoherence was not available. Imprecision was rated as Serious. |
| Astragalus preparations+OX | Kanglaite Injection+OX | 0 | . | . | . |  |  |  |  |  | 1.04 [0.86; 1.26] | Low | Moderate | Not serious | Low | 1.04 [0.86; 1.26] | Low | . | Serious | Very low | NMA estimate was used because incoherence was not available. Imprecision was rated as Serious. |
| Astragalus preparations+OX | Kanglixin Capsules+OX | 0 | . | . | . |  |  |  |  |  | 1.03 [0.87; 1.22] | Low | High | Not serious | Low | 1.03 [0.87; 1.22] | Low | . | Serious | Very low | NMA estimate was used because incoherence was not available. Imprecision was rated as Serious. |
| Astragalus preparations+OX | Lentinan+OX | 0 | . | . | . |  |  |  |  |  | 1.10 [0.94; 1.28] | Low | High | Not serious | Low | 1.10 [0.94; 1.28] | Low | . | Serious | Very low | NMA estimate was used because incoherence was not available. Imprecision was rated as Serious. |
| Astragalus preparations+OX | OX | 3 | 285 | 50.2% | 1.15 [1.02; 1.29] | Very serious | Not serious | Not serious | Undetected | Low | . |  |  |  |  | 1.15 [1.02; 1.29] | Low | . | Not serious | Low | NMA estimate was used because incoherence was not available. Imprecision was rated as Serious. |
| Astragalus preparations+OX | Pingxiao Capsules+OX | 0 | . | . | . |  |  |  |  |  | 1.04 [0.87; 1.24] | Low | Low | Not serious | Low | 1.04 [0.87; 1.24] | Low | . | Serious | Very low | NMA estimate was used because incoherence was not available. Imprecision was rated as Serious. |
| Astragalus preparations+OX | Qizhen Capsule+OX | 0 | . | . | . |  |  |  |  |  | 0.95 [0.76; 1.19] | Low | Low | Not serious | Low | 0.95 [0.76; 1.19] | Low | . | Serious | Very low | NMA estimate was used because incoherence was not available. Imprecision was rated as Serious. |
| Astragalus preparations+OX | Shenfu Injection+OX | 0 | . | . | . |  |  |  |  |  | 1.17 [0.95; 1.44] | Low | Low | Not serious | Low | 1.17 [0.95; 1.44] | Low | . | Serious | Very low | NMA estimate was used because incoherence was not available. Imprecision was rated as Serious. |
| Astragalus preparations+OX | Shengxue Granules+OX | 0 | . | . | . |  |  |  |  |  | 1.48 [1.17; 1.86] | Low | Low | Not serious | Low | 1.48 [1.17; 1.86] | Low | . | Not serious | Low | NMA estimate was used because incoherence was not available. Imprecision was rated as Serious. |
| Astragalus preparations+OX | Shenlian Capsule+OX | 0 | . | . | . |  |  |  |  |  | 0.75 [0.52; 1.09] | Low | Low | Not serious | Low | 0.75 [0.52; 1.09] | Low | . | Serious | Very low | NMA estimate was used because incoherence was not available. Imprecision was rated as Serious. |
| Astragalus preparations+OX | Shenmai Injection+OX | 0 | . | . | . |  |  |  |  |  | 1.10 [0.93; 1.29] | Low | High | Not serious | Low | 1.10 [0.93; 1.29] | Low | . | Serious | Very low | NMA estimate was used because incoherence was not available. Imprecision was rated as Serious. |
| Astragalus preparations+OX | Shenqi Fuzheng Injection+OX | 0 | . | . | . |  |  |  |  |  | 1.03 [0.91; 1.17] | Low | Moderate | Not serious | Low | 1.03 [0.91; 1.17] | Low | . | Serious | Very low | NMA estimate was used because incoherence was not available. Imprecision was rated as Serious. |
| Astragalus preparations+OX | Xiaoaiping Injection+OX | 0 | . | . | . |  |  |  |  |  | 0.99 [0.85; 1.15] | Low | Moderate | Not serious | Low | 0.99 [0.85; 1.15] | Low | . | Serious | Very low | NMA estimate was used because incoherence was not available. Imprecision was rated as Serious. |
| Astragalus preparations+OX | Xihuang Capsules+OX | 0 | . | . | . |  |  |  |  |  | 0.83 [0.64; 1.07] | Low | Low | Not serious | Low | 0.83 [0.64; 1.07] | Low | . | Serious | Very low | NMA estimate was used because incoherence was not available. Imprecision was rated as Serious. |
| Astragalus preparations+OX | Ya Dan Zi Oil Emulsion Injection+OX | 0 | . | . | . |  |  |  |  |  | 1.00 [0.87; 1.15] | Low | Moderate | Not serious | Low | 1.00 [0.87; 1.15] | Low | . | Serious | Very low | NMA estimate was used because incoherence was not available. Imprecision was rated as Serious. |
| Astragalus preparations+OX | Yangzheng Xiaoji Capsules+OX | 0 | . | . | . |  |  |  |  |  | 0.98 [0.83; 1.16] | Low | High | Not serious | Low | 0.98 [0.83; 1.16] | Low | . | Serious | Very low | NMA estimate was used because incoherence was not available. Imprecision was rated as Serious. |
| Astragalus preparations+OX | Zhenqi Fuzheng Granules+OX | 0 | . | . | . |  |  |  |  |  | 0.99 [0.80; 1.22] | Low | Low | Not serious | Low | 0.99 [0.80; 1.22] | Low | . | Serious | Very low | NMA estimate was used because incoherence was not available. Imprecision was rated as Serious. |
| Compound Kushen Injection+OX | Compound Mylabris preparations+OX | 0 | . | . | . |  |  |  |  |  | 0.72 [0.54; 0.98] | Very low | Moderate | Not serious | Very low | 0.72 [0.54; 0.98] | Very low | . | Serious | Very low | NMA estimate was used because incoherence was not available. Imprecision was rated as Serious. |
| Compound Kushen Injection+OX | Ginseng Polysaccharide Injection+OX | 0 | . | . | . |  |  |  |  |  | 1.12 [0.96; 1.30] | Very low | High | Not serious | Very low | 1.12 [0.96; 1.30] | Very low | . | Serious | Very low | NMA estimate was used because incoherence was not available. Imprecision was rated as Serious. |
| Compound Kushen Injection+OX | Huachansu preparations+OX | 0 | . | . | . |  |  |  |  |  | 1.02 [0.94; 1.11] | Very low | High | Not serious | Very low | 1.02 [0.94; 1.11] | Very low | . | Serious | Very low | NMA estimate was used because incoherence was not available. Imprecision was rated as Serious. |
| Compound Kushen Injection+OX | Huai'er Granules+OX | 0 | . | . | . |  |  |  |  |  | 1.14 [0.90; 1.45] | Very low | Low | Not serious | Very low | 1.14 [0.90; 1.45] | Very low | . | Serious | Very low | NMA estimate was used because incoherence was not available. Imprecision was rated as Serious. |
| Compound Kushen Injection+OX | Jinlong Capsules+OX | 0 | . | . | . |  |  |  |  |  | 0.94 [0.75; 1.18] | Very low | High | Not serious | Very low | 0.94 [0.75; 1.18] | Very low | . | Serious | Very low | NMA estimate was used because incoherence was not available. Imprecision was rated as Serious. |
| Compound Kushen Injection+OX | Kangai Injection+OX | 0 | . | . | . |  |  |  |  |  | 0.91 [0.83; 1.00] | Very low | High | Not serious | Very low | 0.91 [0.83; 1.00] | Very low | . | Serious | Very low | NMA estimate was used because incoherence was not available. Imprecision was rated as Serious. |
| Compound Kushen Injection+OX | Kanglaite Injection+OX | 0 | . | . | . |  |  |  |  |  | 1.00 [0.85; 1.17] | Very low | Moderate | Not serious | Very low | 1.00 [0.85; 1.17] | Very low | . | Serious | Very low | NMA estimate was used because incoherence was not available. Imprecision was rated as Serious. |
| Compound Kushen Injection+OX | Kanglixin Capsules+OX | 0 | . | . | . |  |  |  |  |  | 0.98 [0.86; 1.13] | Very low | High | Not serious | Very low | 0.98 [0.86; 1.13] | Very low | . | Serious | Very low | NMA estimate was used because incoherence was not available. Imprecision was rated as Serious. |
| Compound Kushen Injection+OX | Lentinan+OX | 0 | . | . | . |  |  |  |  |  | 1.05 [0.94; 1.18] | Very low | High | Not serious | Very low | 1.05 [0.94; 1.18] | Very low | . | Serious | Very low | NMA estimate was used because incoherence was not available. Imprecision was rated as Serious. |
| Compound Kushen Injection+OX | OX | 14 | 1255 | 63.2% | 1.10 [1.04; 1.16] | Very serious | Not serious | Not serious | Serious | Very low | . |  |  |  |  | 1.10 [1.04; 1.16] | Very low | . | Not serious | Very low | NMA estimate was used because incoherence was not available. Imprecision was rated as Not serious. |
| Compound Kushen Injection+OX | Pingxiao Capsules+OX | 0 | . | . | . |  |  |  |  |  | 0.99 [0.86; 1.14] | Very low | Low | Not serious | Very low | 0.99 [0.86; 1.14] | Very low | . | Serious | Very low | NMA estimate was used because incoherence was not available. Imprecision was rated as Serious. |
| Compound Kushen Injection+OX | Qizhen Capsule+OX | 0 | . | . | . |  |  |  |  |  | 0.91 [0.75; 1.10] | Very low | Low | Not serious | Very low | 0.91 [0.75; 1.10] | Very low | . | Serious | Very low | NMA estimate was used because incoherence was not available. Imprecision was rated as Serious. |
| Compound Kushen Injection+OX | Shenfu Injection+OX | 0 | . | . | . |  |  |  |  |  | 1.12 [0.94; 1.34] | Very low | Low | Not serious | Very low | 1.12 [0.94; 1.34] | Very low | . | Serious | Very low | NMA estimate was used because incoherence was not available. Imprecision was rated as Serious. |
| Compound Kushen Injection+OX | Shengxue Granules+OX | 0 | . | . | . |  |  |  |  |  | 1.41 [1.14; 1.74] | Very low | Low | Not serious | Very low | 1.41 [1.14; 1.74] | Very low | . | Not serious | Low | NMA estimate was used because incoherence was not available. Imprecision was rated as Serious. |
| Compound Kushen Injection+OX | Shenlian Capsule+OX | 0 | . | . | . |  |  |  |  |  | 0.72 [0.50; 1.03] | Very low | Low | Not serious | Very low | 0.72 [0.50; 1.03] | Very low | . | Serious | Very low | NMA estimate was used because incoherence was not available. Imprecision was rated as Serious. |
| Compound Kushen Injection+OX | Shenmai Injection+OX | 0 | . | . | . |  |  |  |  |  | 1.05 [0.92; 1.19] | Very low | High | Not serious | Very low | 1.05 [0.92; 1.19] | Very low | . | Serious | Very low | NMA estimate was used because incoherence was not available. Imprecision was rated as Serious. |
| Compound Kushen Injection+OX | Shenqi Fuzheng Injection+OX | 0 | . | . | . |  |  |  |  |  | 0.99 [0.92; 1.06] | Very low | Moderate | Not serious | Very low | 0.99 [0.92; 1.06] | Very low | . | Serious | Very low | NMA estimate was used because incoherence was not available. Imprecision was rated as Serious. |
| Compound Kushen Injection+OX | Xiaoaiping Injection+OX | 0 | . | . | . |  |  |  |  |  | 0.94 [0.84; 1.05] | Very low | Moderate | Not serious | Very low | 0.94 [0.84; 1.05] | Very low | . | Serious | Very low | NMA estimate was used because incoherence was not available. Imprecision was rated as Serious. |
| Compound Kushen Injection+OX | Xihuang Capsules+OX | 0 | . | . | . |  |  |  |  |  | 0.79 [0.62; 1.00] | Very low | Low | Not serious | Very low | 0.79 [0.62; 1.00] | Very low | . | Serious | Very low | NMA estimate was used because incoherence was not available. Imprecision was rated as Serious. |
| Compound Kushen Injection+OX | Ya Dan Zi Oil Emulsion Injection+OX | 0 | . | . | . |  |  |  |  |  | 0.95 [0.87; 1.05] | Very low | Moderate | Not serious | Very low | 0.95 [0.87; 1.05] | Very low | . | Serious | Very low | NMA estimate was used because incoherence was not available. Imprecision was rated as Serious. |
| Compound Kushen Injection+OX | Yangzheng Xiaoji Capsules+OX | 0 | . | . | . |  |  |  |  |  | 0.94 [0.83; 1.07] | Very low | High | Not serious | Very low | 0.94 [0.83; 1.07] | Very low | . | Serious | Very low | NMA estimate was used because incoherence was not available. Imprecision was rated as Serious. |
| Compound Kushen Injection+OX | Zhenqi Fuzheng Granules+OX | 0 | . | . | . |  |  |  |  |  | 0.95 [0.79; 1.14] | Very low | Low | Not serious | Very low | 0.95 [0.79; 1.14] | Very low | . | Serious | Very low | NMA estimate was used because incoherence was not available. Imprecision was rated as Serious. |
| Compound Mylabris preparations+OX | Ginseng Polysaccharide Injection+OX | 0 | . | . | . |  |  |  |  |  | 1.54 [1.11; 2.14] | Moderate | High | Not serious | Moderate | 1.54 [1.11; 2.14] | Moderate | . | Not serious | Moderate | NMA estimate was used because incoherence was not available. Imprecision was rated as Serious. |
| Compound Mylabris preparations+OX | Huachansu preparations+OX | 0 | . | . | . |  |  |  |  |  | 1.41 [1.04; 1.90] | Moderate | High | Not serious | Moderate | 1.41 [1.04; 1.90] | Moderate | . | Not serious | Moderate | NMA estimate was used because incoherence was not available. Imprecision was rated as Serious. |
| Compound Mylabris preparations+OX | Huai'er Granules+OX | 0 | . | . | . |  |  |  |  |  | 1.57 [1.08; 2.30] | Moderate | Low | Not serious | Low | 1.57 [1.08; 2.30] | Low | . | Not serious | Low | NMA estimate was used because incoherence was not available. Imprecision was rated as Serious. |
| Compound Mylabris preparations+OX | Jinlong Capsules+OX | 0 | . | . | . |  |  |  |  |  | 1.30 [0.90; 1.87] | Moderate | High | Not serious | Moderate | 1.30 [0.90; 1.87] | Moderate | . | Serious | Low | NMA estimate was used because incoherence was not available. Imprecision was rated as Serious. |
| Compound Mylabris preparations+OX | Kangai Injection+OX | 0 | . | . | . |  |  |  |  |  | 1.26 [0.93; 1.71] | Moderate | High | Not serious | Moderate | 1.26 [0.93; 1.71] | Moderate | . | Serious | Low | NMA estimate was used because incoherence was not available. Imprecision was rated as Serious. |
| Compound Mylabris preparations+OX | Kanglaite Injection+OX | 0 | . | . | . |  |  |  |  |  | 1.37 [0.99; 1.91] | Moderate | Moderate | Not serious | Moderate | 1.37 [0.99; 1.91] | Moderate | . | Serious | Low | NMA estimate was used because incoherence was not available. Imprecision was rated as Serious. |
| Compound Mylabris preparations+OX | Kanglixin Capsules+OX | 0 | . | . | . |  |  |  |  |  | 1.36 [0.99; 1.88] | Moderate | High | Not serious | Moderate | 1.36 [0.99; 1.88] | Moderate | . | Serious | Low | NMA estimate was used because incoherence was not available. Imprecision was rated as Serious. |
| Compound Mylabris preparations+OX | Lentinan+OX | 0 | . | . | . |  |  |  |  |  | 1.45 [1.06; 1.98] | Moderate | High | Not serious | Moderate | 1.45 [1.06; 1.98] | Moderate | . | Not serious | Moderate | NMA estimate was used because incoherence was not available. Imprecision was rated as Serious. |
| Compound Mylabris preparations+OX | OX | 2 | 139 | 0.0% | 1.51 [1.13; 2.03] | Serious | Not serious | Not serious | Undetected | Moderate | . |  |  |  |  | 1.51 [1.13; 2.03] | Moderate | . | Not serious | Moderate | NMA estimate was used because incoherence was not available. Imprecision was rated as Serious. |
| Compound Mylabris preparations+OX | Pingxiao Capsules+OX | 0 | . | . | . |  |  |  |  |  | 1.37 [0.99; 1.89] | Moderate | Low | Not serious | Low | 1.37 [0.99; 1.89] | Low | . | Serious | Very low | NMA estimate was used because incoherence was not available. Imprecision was rated as Serious. |
| Compound Mylabris preparations+OX | Qizhen Capsule+OX | 0 | . | . | . |  |  |  |  |  | 1.25 [0.88; 1.78] | Moderate | Low | Not serious | Low | 1.25 [0.88; 1.78] | Low | . | Serious | Very low | NMA estimate was used because incoherence was not available. Imprecision was rated as Serious. |
| Compound Mylabris preparations+OX | Shenfu Injection+OX | 0 | . | . | . |  |  |  |  |  | 1.54 [1.10; 2.17] | Moderate | Low | Not serious | Low | 1.54 [1.10; 2.17] | Low | . | Not serious | Low | NMA estimate was used because incoherence was not available. Imprecision was rated as Serious. |
| Compound Mylabris preparations+OX | Shengxue Granules+OX | 0 | . | . | . |  |  |  |  |  | 1.95 [1.36; 2.78] | Moderate | Low | Not serious | Low | 1.95 [1.36; 2.78] | Low | . | Not serious | Low | NMA estimate was used because incoherence was not available. Imprecision was rated as Serious. |
| Compound Mylabris preparations+OX | Shenlian Capsule+OX | 0 | . | . | . |  |  |  |  |  | 0.99 [0.62; 1.57] | Moderate | Low | Not serious | Low | 0.99 [0.62; 1.57] | Low | . | Serious | Very low | NMA estimate was used because incoherence was not available. Imprecision was rated as Serious. |
| Compound Mylabris preparations+OX | Shenmai Injection+OX | 0 | . | . | . |  |  |  |  |  | 1.45 [1.06; 1.99] | Moderate | High | Not serious | Moderate | 1.45 [1.06; 1.99] | Moderate | . | Not serious | Moderate | NMA estimate was used because incoherence was not available. Imprecision was rated as Serious. |
| Compound Mylabris preparations+OX | Shenqi Fuzheng Injection+OX | 0 | . | . | . |  |  |  |  |  | 1.36 [1.01; 1.84] | Moderate | Moderate | Not serious | Moderate | 1.36 [1.01; 1.84] | Moderate | . | Not serious | Moderate | NMA estimate was used because incoherence was not available. Imprecision was rated as Serious. |
| Compound Mylabris preparations+OX | Xiaoaiping Injection+OX | 0 | . | . | . |  |  |  |  |  | 1.30 [0.96; 1.78] | Moderate | Moderate | Not serious | Moderate | 1.30 [0.96; 1.78] | Moderate | . | Serious | Low | NMA estimate was used because incoherence was not available. Imprecision was rated as Serious. |
| Compound Mylabris preparations+OX | Xihuang Capsules+OX | 0 | . | . | . |  |  |  |  |  | 1.09 [0.75; 1.59] | Moderate | Low | Not serious | Low | 1.09 [0.75; 1.59] | Low | . | Serious | Very low | NMA estimate was used because incoherence was not available. Imprecision was rated as Serious. |
| Compound Mylabris preparations+OX | Ya Dan Zi Oil Emulsion Injection+OX | 0 | . | . | . |  |  |  |  |  | 1.32 [0.97; 1.79] | Moderate | Moderate | Not serious | Moderate | 1.32 [0.97; 1.79] | Moderate | . | Serious | Low | NMA estimate was used because incoherence was not available. Imprecision was rated as Serious. |
| Compound Mylabris preparations+OX | Yangzheng Xiaoji Capsules+OX | 0 | . | . | . |  |  |  |  |  | 1.30 [0.94; 1.78] | Moderate | High | Not serious | Moderate | 1.30 [0.94; 1.78] | Moderate | . | Serious | Low | NMA estimate was used because incoherence was not available. Imprecision was rated as Serious. |
| Compound Mylabris preparations+OX | Zhenqi Fuzheng Granules+OX | 0 | . | . | . |  |  |  |  |  | 1.31 [0.93; 1.84] | Moderate | Low | Not serious | Low | 1.31 [0.93; 1.84] | Low | . | Serious | Very low | NMA estimate was used because incoherence was not available. Imprecision was rated as Serious. |
| Ginseng Polysaccharide Injection+OX | Huachansu preparations+OX | 0 | . | . | . |  |  |  |  |  | 0.91 [0.78; 1.06] | High | High | Not serious | High | 0.91 [0.78; 1.06] | High | . | Serious | Moderate | NMA estimate was used because incoherence was not available. Imprecision was rated as Serious. |
| Ginseng Polysaccharide Injection+OX | Huai'er Granules+OX | 0 | . | . | . |  |  |  |  |  | 1.02 [0.77; 1.34] | High | Low | Not serious | Low | 1.02 [0.77; 1.34] | Low | . | Serious | Very low | NMA estimate was used because incoherence was not available. Imprecision was rated as Serious. |
| Ginseng Polysaccharide Injection+OX | Jinlong Capsules+OX | 0 | . | . | . |  |  |  |  |  | 0.84 [0.65; 1.09] | High | High | Not serious | High | 0.84 [0.65; 1.09] | High | . | Serious | Moderate | NMA estimate was used because incoherence was not available. Imprecision was rated as Serious. |
| Ginseng Polysaccharide Injection+OX | Kangai Injection+OX | 0 | . | . | . |  |  |  |  |  | 0.82 [0.70; 0.96] | High | High | Not serious | High | 0.82 [0.70; 0.96] | High | . | Serious | Moderate | NMA estimate was used because incoherence was not available. Imprecision was rated as Serious. |
| Ginseng Polysaccharide Injection+OX | Kanglaite Injection+OX | 0 | . | . | . |  |  |  |  |  | 0.89 [0.72; 1.09] | High | Moderate | Not serious | Moderate | 0.89 [0.72; 1.09] | Moderate | . | Serious | Low | NMA estimate was used because incoherence was not available. Imprecision was rated as Serious. |
| Ginseng Polysaccharide Injection+OX | Kanglixin Capsules+OX | 0 | . | . | . |  |  |  |  |  | 0.88 [0.73; 1.07] | High | High | Not serious | High | 0.88 [0.73; 1.07] | High | . | Serious | Moderate | NMA estimate was used because incoherence was not available. Imprecision was rated as Serious. |
| Ginseng Polysaccharide Injection+OX | Lentinan+OX | 0 | . | . | . |  |  |  |  |  | 0.94 [0.79; 1.12] | High | High | Not serious | High | 0.94 [0.79; 1.12] | High | . | Serious | Moderate | NMA estimate was used because incoherence was not available. Imprecision was rated as Serious. |
| Ginseng Polysaccharide Injection+OX | OX | 2 | 131 | 0.0% | 0.98 [0.85; 1.13] | Not serious | Not serious | Not serious | Undetected | High | . |  |  |  |  | 0.98 [0.85; 1.13] | High | . | Serious | Moderate | NMA estimate was used because incoherence was not available. Imprecision was rated as Serious. |
| Ginseng Polysaccharide Injection+OX | Pingxiao Capsules+OX | 0 | . | . | . |  |  |  |  |  | 0.89 [0.73; 1.07] | High | Low | Not serious | Low | 0.89 [0.73; 1.07] | Low | . | Serious | Very low | NMA estimate was used because incoherence was not available. Imprecision was rated as Serious. |
| Ginseng Polysaccharide Injection+OX | Qizhen Capsule+OX | 0 | . | . | . |  |  |  |  |  | 0.81 [0.64; 1.03] | High | Low | Not serious | Low | 0.81 [0.64; 1.03] | Low | . | Serious | Very low | NMA estimate was used because incoherence was not available. Imprecision was rated as Serious. |
| Ginseng Polysaccharide Injection+OX | Shenfu Injection+OX | 0 | . | . | . |  |  |  |  |  | 1.00 [0.80; 1.25] | High | Low | Not serious | Low | 1.00 [0.80; 1.25] | Low | . | Serious | Very low | NMA estimate was used because incoherence was not available. Imprecision was rated as Serious. |
| Ginseng Polysaccharide Injection+OX | Shengxue Granules+OX | 0 | . | . | . |  |  |  |  |  | 1.26 [0.98; 1.61] | High | Low | Not serious | Low | 1.26 [0.98; 1.61] | Low | . | Serious | Very low | NMA estimate was used because incoherence was not available. Imprecision was rated as Serious. |
| Ginseng Polysaccharide Injection+OX | Shenlian Capsule+OX | 0 | . | . | . |  |  |  |  |  | 0.64 [0.44; 0.94] | High | Low | Not serious | Low | 0.64 [0.44; 0.94] | Low | . | Serious | Very low | NMA estimate was used because incoherence was not available. Imprecision was rated as Serious. |
| Ginseng Polysaccharide Injection+OX | Shenmai Injection+OX | 0 | . | . | . |  |  |  |  |  | 0.94 [0.78; 1.13] | High | High | Not serious | High | 0.94 [0.78; 1.13] | High | . | Serious | Moderate | NMA estimate was used because incoherence was not available. Imprecision was rated as Serious. |
| Ginseng Polysaccharide Injection+OX | Shenqi Fuzheng Injection+OX | 0 | . | . | . |  |  |  |  |  | 0.88 [0.76; 1.03] | High | Moderate | Not serious | Moderate | 0.88 [0.76; 1.03] | Moderate | . | Serious | Low | NMA estimate was used because incoherence was not available. Imprecision was rated as Serious. |
| Ginseng Polysaccharide Injection+OX | Xiaoaiping Injection+OX | 0 | . | . | . |  |  |  |  |  | 0.84 [0.71; 1.00] | High | Moderate | Not serious | Moderate | 0.84 [0.71; 1.00] | Moderate | . | Serious | Low | NMA estimate was used because incoherence was not available. Imprecision was rated as Serious. |
| Ginseng Polysaccharide Injection+OX | Xihuang Capsules+OX | 0 | . | . | . |  |  |  |  |  | 0.70 [0.54; 0.93] | High | Low | Not serious | Low | 0.70 [0.54; 0.93] | Low | . | Serious | Very low | NMA estimate was used because incoherence was not available. Imprecision was rated as Serious. |
| Ginseng Polysaccharide Injection+OX | Ya Dan Zi Oil Emulsion Injection+OX | 0 | . | . | . |  |  |  |  |  | 0.85 [0.73; 1.00] | High | Moderate | Not serious | Moderate | 0.85 [0.73; 1.00] | Moderate | . | Serious | Low | NMA estimate was used because incoherence was not available. Imprecision was rated as Serious. |
| Ginseng Polysaccharide Injection+OX | Yangzheng Xiaoji Capsules+OX | 0 | . | . | . |  |  |  |  |  | 0.84 [0.70; 1.01] | High | High | Not serious | High | 0.84 [0.70; 1.01] | High | . | Serious | Moderate | NMA estimate was used because incoherence was not available. Imprecision was rated as Serious. |
| Ginseng Polysaccharide Injection+OX | Zhenqi Fuzheng Granules+OX | 0 | . | . | . |  |  |  |  |  | 0.85 [0.67; 1.06] | High | Low | Not serious | Low | 0.85 [0.67; 1.06] | Low | . | Serious | Very low | NMA estimate was used because incoherence was not available. Imprecision was rated as Serious. |
| Huachansu preparations+OX | Huai'er Granules+OX | 0 | . | . | . |  |  |  |  |  | 1.12 [0.88; 1.43] | High | Low | Not serious | Low | 1.12 [0.88; 1.43] | Low | . | Serious | Very low | NMA estimate was used because incoherence was not available. Imprecision was rated as Serious. |
| Huachansu preparations+OX | Jinlong Capsules+OX | 0 | . | . | . |  |  |  |  |  | 0.92 [0.74; 1.16] | High | High | Not serious | High | 0.92 [0.74; 1.16] | High | . | Serious | Moderate | NMA estimate was used because incoherence was not available. Imprecision was rated as Serious. |
| Huachansu preparations+OX | Kangai Injection+OX | 0 | . | . | . |  |  |  |  |  | 0.90 [0.81; 0.99] | High | High | Not serious | High | 0.90 [0.81; 0.99] | High | . | Serious | Moderate | NMA estimate was used because incoherence was not available. Imprecision was rated as Serious. |
| Huachansu preparations+OX | Kanglaite Injection+OX | 0 | . | . | . |  |  |  |  |  | 0.98 [0.83; 1.15] | High | Moderate | Not serious | Moderate | 0.98 [0.83; 1.15] | Moderate | . | Serious | Low | NMA estimate was used because incoherence was not available. Imprecision was rated as Serious. |
| Huachansu preparations+OX | Kanglixin Capsules+OX | 0 | . | . | . |  |  |  |  |  | 0.97 [0.84; 1.11] | High | High | Not serious | High | 0.97 [0.84; 1.11] | High | . | Serious | Moderate | NMA estimate was used because incoherence was not available. Imprecision was rated as Serious. |
| Huachansu preparations+OX | Lentinan+OX | 0 | . | . | . |  |  |  |  |  | 1.03 [0.92; 1.16] | High | High | Not serious | High | 1.03 [0.92; 1.16] | High | . | Serious | Moderate | NMA estimate was used because incoherence was not available. Imprecision was rated as Serious. |
| Huachansu preparations+OX | OX | 9 | 591 | 0.0% | 1.08 [1.01; 1.14] | Not serious | Not serious | Not serious | Undetected | High | . |  |  |  |  | 1.08 [1.01; 1.14] | High | . | Not serious | High | NMA estimate was used because incoherence was not available. Imprecision was rated as Serious. |
| Huachansu preparations+OX | Pingxiao Capsules+OX | 0 | . | . | . |  |  |  |  |  | 0.97 [0.85; 1.12] | High | Low | Not serious | Low | 0.97 [0.85; 1.12] | Low | . | Serious | Very low | NMA estimate was used because incoherence was not available. Imprecision was rated as Serious. |
| Huachansu preparations+OX | Qizhen Capsule+OX | 0 | . | . | . |  |  |  |  |  | 0.89 [0.73; 1.09] | High | Low | Not serious | Low | 0.89 [0.73; 1.09] | Low | . | Serious | Very low | NMA estimate was used because incoherence was not available. Imprecision was rated as Serious. |
| Huachansu preparations+OX | Shenfu Injection+OX | 0 | . | . | . |  |  |  |  |  | 1.10 [0.92; 1.31] | High | Low | Not serious | Low | 1.10 [0.92; 1.31] | Low | . | Serious | Very low | NMA estimate was used because incoherence was not available. Imprecision was rated as Serious. |
| Huachansu preparations+OX | Shengxue Granules+OX | 0 | . | . | . |  |  |  |  |  | 1.38 [1.12; 1.71] | High | Low | Not serious | Low | 1.38 [1.12; 1.71] | Low | . | Not serious | Low | NMA estimate was used because incoherence was not available. Imprecision was rated as Serious. |
| Huachansu preparations+OX | Shenlian Capsule+OX | 0 | . | . | . |  |  |  |  |  | 0.70 [0.49; 1.01] | High | Low | Not serious | Low | 0.70 [0.49; 1.01] | Low | . | Serious | Very low | NMA estimate was used because incoherence was not available. Imprecision was rated as Serious. |
| Huachansu preparations+OX | Shenmai Injection+OX | 0 | . | . | . |  |  |  |  |  | 1.03 [0.90; 1.17] | High | High | Not serious | High | 1.03 [0.90; 1.17] | High | . | Serious | Moderate | NMA estimate was used because incoherence was not available. Imprecision was rated as Serious. |
| Huachansu preparations+OX | Shenqi Fuzheng Injection+OX | 0 | . | . | . |  |  |  |  |  | 0.97 [0.90; 1.05] | High | Moderate | Not serious | Moderate | 0.97 [0.90; 1.05] | Moderate | . | Serious | Low | NMA estimate was used because incoherence was not available. Imprecision was rated as Serious. |
| Huachansu preparations+OX | Xiaoaiping Injection+OX | 0 | . | . | . |  |  |  |  |  | 0.93 [0.83; 1.04] | High | Moderate | Not serious | Moderate | 0.93 [0.83; 1.04] | Moderate | . | Serious | Low | NMA estimate was used because incoherence was not available. Imprecision was rated as Serious. |
| Huachansu preparations+OX | Xihuang Capsules+OX | 0 | . | . | . |  |  |  |  |  | 0.77 [0.61; 0.99] | High | Low | Not serious | Low | 0.77 [0.61; 0.99] | Low | . | Serious | Very low | NMA estimate was used because incoherence was not available. Imprecision was rated as Serious. |
| Huachansu preparations+OX | Ya Dan Zi Oil Emulsion Injection+OX | 0 | . | . | . |  |  |  |  |  | 0.94 [0.85; 1.03] | High | Moderate | Not serious | Moderate | 0.94 [0.85; 1.03] | Moderate | . | Serious | Low | NMA estimate was used because incoherence was not available. Imprecision was rated as Serious. |
| Huachansu preparations+OX | Yangzheng Xiaoji Capsules+OX | 0 | . | . | . |  |  |  |  |  | 0.92 [0.81; 1.05] | High | High | Not serious | High | 0.92 [0.81; 1.05] | High | . | Serious | Moderate | NMA estimate was used because incoherence was not available. Imprecision was rated as Serious. |
| Huachansu preparations+OX | Zhenqi Fuzheng Granules+OX | 0 | . | . | . |  |  |  |  |  | 0.93 [0.77; 1.12] | High | Low | Not serious | Low | 0.93 [0.77; 1.12] | Low | . | Serious | Very low | NMA estimate was used because incoherence was not available. Imprecision was rated as Serious. |
| Huai'er Granules+OX | Jinlong Capsules+OX | 0 | . | . | . |  |  |  |  |  | 0.82 [0.60; 1.13] | Low | High | Not serious | Low | 0.82 [0.60; 1.13] | Low | . | Serious | Very low | NMA estimate was used because incoherence was not available. Imprecision was rated as Serious. |
| Huai'er Granules+OX | Kangai Injection+OX | 0 | . | . | . |  |  |  |  |  | 0.80 [0.63; 1.03] | Low | High | Not serious | Low | 0.80 [0.63; 1.03] | Low | . | Serious | Very low | NMA estimate was used because incoherence was not available. Imprecision was rated as Serious. |
| Huai'er Granules+OX | Kanglaite Injection+OX | 0 | . | . | . |  |  |  |  |  | 0.87 [0.66; 1.15] | Low | Moderate | Not serious | Low | 0.87 [0.66; 1.15] | Low | . | Serious | Very low | NMA estimate was used because incoherence was not available. Imprecision was rated as Serious. |
| Huai'er Granules+OX | Kanglixin Capsules+OX | 0 | . | . | . |  |  |  |  |  | 0.86 [0.66; 1.13] | Low | High | Not serious | Low | 0.86 [0.66; 1.13] | Low | . | Serious | Very low | NMA estimate was used because incoherence was not available. Imprecision was rated as Serious. |
| Huai'er Granules+OX | Lentinan+OX | 0 | . | . | . |  |  |  |  |  | 0.92 [0.71; 1.19] | Low | High | Not serious | Low | 0.92 [0.71; 1.19] | Low | . | Serious | Very low | NMA estimate was used because incoherence was not available. Imprecision was rated as Serious. |
| Huai'er Granules+OX | OX | 1 | 60 | . | 0.96 [0.76; 1.22] | Very serious | Not serious | Not serious | Undetected | Low | . |  |  |  |  | 0.96 [0.76; 1.22] | Low | . | Serious | Very low | NMA estimate was used because incoherence was not available. Imprecision was rated as Serious. |
| Huai'er Granules+OX | Pingxiao Capsules+OX | 0 | . | . | . |  |  |  |  |  | 0.87 [0.67; 1.14] | Low | Low | Not serious | Low | 0.87 [0.67; 1.14] | Low | . | Serious | Very low | NMA estimate was used because incoherence was not available. Imprecision was rated as Serious. |
| Huai'er Granules+OX | Qizhen Capsule+OX | 0 | . | . | . |  |  |  |  |  | 0.80 [0.59; 1.08] | Low | Low | Not serious | Low | 0.80 [0.59; 1.08] | Low | . | Serious | Very low | NMA estimate was used because incoherence was not available. Imprecision was rated as Serious. |
| Huai'er Granules+OX | Shenfu Injection+OX | 0 | . | . | . |  |  |  |  |  | 0.98 [0.73; 1.31] | Low | Low | Not serious | Low | 0.98 [0.73; 1.31] | Low | . | Serious | Very low | NMA estimate was used because incoherence was not available. Imprecision was rated as Serious. |
| Huai'er Granules+OX | Shengxue Granules+OX | 0 | . | . | . |  |  |  |  |  | 1.24 [0.91; 1.68] | Low | Low | Not serious | Low | 1.24 [0.91; 1.68] | Low | . | Serious | Very low | NMA estimate was used because incoherence was not available. Imprecision was rated as Serious. |
| Huai'er Granules+OX | Shenlian Capsule+OX | 0 | . | . | . |  |  |  |  |  | 0.63 [0.41; 0.96] | Low | Low | Not serious | Low | 0.63 [0.41; 0.96] | Low | . | Serious | Very low | NMA estimate was used because incoherence was not available. Imprecision was rated as Serious. |
| Huai'er Granules+OX | Shenmai Injection+OX | 0 | . | . | . |  |  |  |  |  | 0.92 [0.71; 1.20] | Low | High | Not serious | Low | 0.92 [0.71; 1.20] | Low | . | Serious | Very low | NMA estimate was used because incoherence was not available. Imprecision was rated as Serious. |
| Huai'er Granules+OX | Shenqi Fuzheng Injection+OX | 0 | . | . | . |  |  |  |  |  | 0.87 [0.68; 1.10] | Low | Moderate | Not serious | Low | 0.87 [0.68; 1.10] | Low | . | Serious | Very low | NMA estimate was used because incoherence was not available. Imprecision was rated as Serious. |
| Huai'er Granules+OX | Xiaoaiping Injection+OX | 0 | . | . | . |  |  |  |  |  | 0.83 [0.64; 1.07] | Low | Moderate | Not serious | Low | 0.83 [0.64; 1.07] | Low | . | Serious | Very low | NMA estimate was used because incoherence was not available. Imprecision was rated as Serious. |
| Huai'er Granules+OX | Xihuang Capsules+OX | 0 | . | . | . |  |  |  |  |  | 0.69 [0.50; 0.96] | Low | Low | Not serious | Low | 0.69 [0.50; 0.96] | Low | . | Serious | Very low | NMA estimate was used because incoherence was not available. Imprecision was rated as Serious. |
| Huai'er Granules+OX | Ya Dan Zi Oil Emulsion Injection+OX | 0 | . | . | . |  |  |  |  |  | 0.84 [0.65; 1.07] | Low | Moderate | Not serious | Low | 0.84 [0.65; 1.07] | Low | . | Serious | Very low | NMA estimate was used because incoherence was not available. Imprecision was rated as Serious. |
| Huai'er Granules+OX | Yangzheng Xiaoji Capsules+OX | 0 | . | . | . |  |  |  |  |  | 0.82 [0.63; 1.07] | Low | High | Not serious | Low | 0.82 [0.63; 1.07] | Low | . | Serious | Very low | NMA estimate was used because incoherence was not available. Imprecision was rated as Serious. |
| Huai'er Granules+OX | Zhenqi Fuzheng Granules+OX | 0 | . | . | . |  |  |  |  |  | 0.83 [0.62; 1.11] | Low | Low | Not serious | Low | 0.83 [0.62; 1.11] | Low | . | Serious | Very low | NMA estimate was used because incoherence was not available. Imprecision was rated as Serious. |
| Jinlong Capsules+OX | Kangai Injection+OX | 0 | . | . | . |  |  |  |  |  | 0.97 [0.77; 1.22] | High | High | Not serious | High | 0.97 [0.77; 1.22] | High | . | Serious | Moderate | NMA estimate was used because incoherence was not available. Imprecision was rated as Serious. |
| Jinlong Capsules+OX | Kanglaite Injection+OX | 0 | . | . | . |  |  |  |  |  | 1.06 [0.81; 1.38] | High | Moderate | Not serious | Moderate | 1.06 [0.81; 1.38] | Moderate | . | Serious | Low | NMA estimate was used because incoherence was not available. Imprecision was rated as Serious. |
| Jinlong Capsules+OX | Kanglixin Capsules+OX | 0 | . | . | . |  |  |  |  |  | 1.05 [0.82; 1.35] | High | High | Not serious | High | 1.05 [0.82; 1.35] | High | . | Serious | Moderate | NMA estimate was used because incoherence was not available. Imprecision was rated as Serious. |
| Jinlong Capsules+OX | Lentinan+OX | 0 | . | . | . |  |  |  |  |  | 1.12 [0.88; 1.42] | High | High | Not serious | High | 1.12 [0.88; 1.42] | High | . | Serious | Moderate | NMA estimate was used because incoherence was not available. Imprecision was rated as Serious. |
| Jinlong Capsules+OX | OX | 2 | 137 | 43.0% | 1.17 [0.94; 1.45] | Not serious | Not serious | Not serious | Undetected | High | . |  |  |  |  | 1.17 [0.94; 1.45] | High | . | Serious | Moderate | NMA estimate was used because incoherence was not available. Imprecision was rated as Serious. |
| Jinlong Capsules+OX | Pingxiao Capsules+OX | 0 | . | . | . |  |  |  |  |  | 1.06 [0.82; 1.36] | High | Low | Not serious | Low | 1.06 [0.82; 1.36] | Low | . | Serious | Very low | NMA estimate was used because incoherence was not available. Imprecision was rated as Serious. |
| Jinlong Capsules+OX | Qizhen Capsule+OX | 0 | . | . | . |  |  |  |  |  | 0.97 [0.73; 1.29] | High | Low | Not serious | Low | 0.97 [0.73; 1.29] | Low | . | Serious | Very low | NMA estimate was used because incoherence was not available. Imprecision was rated as Serious. |
| Jinlong Capsules+OX | Shenfu Injection+OX | 0 | . | . | . |  |  |  |  |  | 1.19 [0.90; 1.57] | High | Low | Not serious | Low | 1.19 [0.90; 1.57] | Low | . | Serious | Very low | NMA estimate was used because incoherence was not available. Imprecision was rated as Serious. |
| Jinlong Capsules+OX | Shengxue Granules+OX | 0 | . | . | . |  |  |  |  |  | 1.50 [1.12; 2.02] | High | Low | Not serious | Low | 1.50 [1.12; 2.02] | Low | . | Not serious | Low | NMA estimate was used because incoherence was not available. Imprecision was rated as Serious. |
| Jinlong Capsules+OX | Shenlian Capsule+OX | 0 | . | . | . |  |  |  |  |  | 0.76 [0.50; 1.16] | High | Low | Not serious | Low | 0.76 [0.50; 1.16] | Low | . | Serious | Very low | NMA estimate was used because incoherence was not available. Imprecision was rated as Serious. |
| Jinlong Capsules+OX | Shenmai Injection+OX | 0 | . | . | . |  |  |  |  |  | 1.12 [0.87; 1.43] | High | High | Not serious | High | 1.12 [0.87; 1.43] | High | . | Serious | Moderate | NMA estimate was used because incoherence was not available. Imprecision was rated as Serious. |
| Jinlong Capsules+OX | Shenqi Fuzheng Injection+OX | 0 | . | . | . |  |  |  |  |  | 1.05 [0.84; 1.31] | High | Moderate | Not serious | Moderate | 1.05 [0.84; 1.31] | Moderate | . | Serious | Low | NMA estimate was used because incoherence was not available. Imprecision was rated as Serious. |
| Jinlong Capsules+OX | Xiaoaiping Injection+OX | 0 | . | . | . |  |  |  |  |  | 1.00 [0.79; 1.27] | High | Moderate | Not serious | Moderate | 1.00 [0.79; 1.27] | Moderate | . | Serious | Low | NMA estimate was used because incoherence was not available. Imprecision was rated as Serious. |
| Jinlong Capsules+OX | Xihuang Capsules+OX | 0 | . | . | . |  |  |  |  |  | 0.84 [0.61; 1.15] | High | Low | Not serious | Low | 0.84 [0.61; 1.15] | Low | . | Serious | Very low | NMA estimate was used because incoherence was not available. Imprecision was rated as Serious. |
| Jinlong Capsules+OX | Ya Dan Zi Oil Emulsion Injection+OX | 0 | . | . | . |  |  |  |  |  | 1.01 [0.81; 1.28] | High | Moderate | Not serious | Moderate | 1.01 [0.81; 1.28] | Moderate | . | Serious | Low | NMA estimate was used because incoherence was not available. Imprecision was rated as Serious. |
| Jinlong Capsules+OX | Yangzheng Xiaoji Capsules+OX | 0 | . | . | . |  |  |  |  |  | 1.00 [0.78; 1.28] | High | High | Not serious | High | 1.00 [0.78; 1.28] | High | . | Serious | Moderate | NMA estimate was used because incoherence was not available. Imprecision was rated as Serious. |
| Jinlong Capsules+OX | Zhenqi Fuzheng Granules+OX | 0 | . | . | . |  |  |  |  |  | 1.01 [0.76; 1.33] | High | Low | Not serious | Low | 1.01 [0.76; 1.33] | Low | . | Serious | Very low | NMA estimate was used because incoherence was not available. Imprecision was rated as Serious. |
| Kangai Injection+OX | Kanglaite Injection+OX | 0 | . | . | . |  |  |  |  |  | 1.09 [0.92; 1.29] | High | Moderate | Not serious | Moderate | 1.09 [0.92; 1.29] | Moderate | . | Serious | Low | NMA estimate was used because incoherence was not available. Imprecision was rated as Serious. |
| Kangai Injection+OX | Kanglixin Capsules+OX | 0 | . | . | . |  |  |  |  |  | 1.08 [0.93; 1.25] | High | High | Not serious | High | 1.08 [0.93; 1.25] | High | . | Serious | Moderate | NMA estimate was used because incoherence was not available. Imprecision was rated as Serious. |
| Kangai Injection+OX | Lentinan+OX | 0 | . | . | . |  |  |  |  |  | 1.15 [1.02; 1.30] | High | High | Not serious | High | 1.15 [1.02; 1.30] | High | . | Not serious | High | NMA estimate was used because incoherence was not available. Imprecision was rated as Serious. |
| Kangai Injection+OX | OX | 11 | 1101 | 0.0% | 1.20 [1.11; 1.29] | Not serious | Not serious | Not serious | Not serious | High | . |  |  |  |  | 1.20 [1.11; 1.29] | High | . | Not serious | High | NMA estimate was used because incoherence was not available. Imprecision was rated as Not serious. |
| Kangai Injection+OX | Pingxiao Capsules+OX | 0 | . | . | . |  |  |  |  |  | 1.09 [0.94; 1.26] | High | Low | Not serious | Low | 1.09 [0.94; 1.26] | Low | . | Serious | Very low | NMA estimate was used because incoherence was not available. Imprecision was rated as Serious. |
| Kangai Injection+OX | Qizhen Capsule+OX | 0 | . | . | . |  |  |  |  |  | 0.99 [0.81; 1.22] | High | Low | Not serious | Low | 0.99 [0.81; 1.22] | Low | . | Serious | Very low | NMA estimate was used because incoherence was not available. Imprecision was rated as Serious. |
| Kangai Injection+OX | Shenfu Injection+OX | 0 | . | . | . |  |  |  |  |  | 1.22 [1.02; 1.47] | High | Low | Not serious | Low | 1.22 [1.02; 1.47] | Low | . | Not serious | Low | NMA estimate was used because incoherence was not available. Imprecision was rated as Serious. |
| Kangai Injection+OX | Shengxue Granules+OX | 0 | . | . | . |  |  |  |  |  | 1.54 [1.25; 1.91] | High | Low | Not serious | Low | 1.54 [1.25; 1.91] | Low | . | Not serious | Low | NMA estimate was used because incoherence was not available. Imprecision was rated as Serious. |
| Kangai Injection+OX | Shenlian Capsule+OX | 0 | . | . | . |  |  |  |  |  | 0.78 [0.54; 1.13] | High | Low | Not serious | Low | 0.78 [0.54; 1.13] | Low | . | Serious | Very low | NMA estimate was used because incoherence was not available. Imprecision was rated as Serious. |
| Kangai Injection+OX | Shenmai Injection+OX | 0 | . | . | . |  |  |  |  |  | 1.15 [1.00; 1.32] | High | High | Not serious | High | 1.15 [1.00; 1.32] | High | . | Serious | Moderate | NMA estimate was used because incoherence was not available. Imprecision was rated as Serious. |
| Kangai Injection+OX | Shenqi Fuzheng Injection+OX | 0 | . | . | . |  |  |  |  |  | 1.08 [0.99; 1.18] | High | Moderate | Not serious | Moderate | 1.08 [0.99; 1.18] | Moderate | . | Serious | Low | NMA estimate was used because incoherence was not available. Imprecision was rated as Serious. |
| Kangai Injection+OX | Xiaoaiping Injection+OX | 0 | . | . | . |  |  |  |  |  | 1.03 [0.91; 1.17] | High | Moderate | Not serious | Moderate | 1.03 [0.91; 1.17] | Moderate | . | Serious | Low | NMA estimate was used because incoherence was not available. Imprecision was rated as Serious. |
| Kangai Injection+OX | Xihuang Capsules+OX | 0 | . | . | . |  |  |  |  |  | 0.86 [0.67; 1.10] | High | Low | Not serious | Low | 0.86 [0.67; 1.10] | Low | . | Serious | Very low | NMA estimate was used because incoherence was not available. Imprecision was rated as Serious. |
| Kangai Injection+OX | Ya Dan Zi Oil Emulsion Injection+OX | 0 | . | . | . |  |  |  |  |  | 1.04 [0.94; 1.16] | High | Moderate | Not serious | Moderate | 1.04 [0.94; 1.16] | Moderate | . | Serious | Low | NMA estimate was used because incoherence was not available. Imprecision was rated as Serious. |
| Kangai Injection+OX | Yangzheng Xiaoji Capsules+OX | 0 | . | . | . |  |  |  |  |  | 1.03 [0.90; 1.18] | High | High | Not serious | High | 1.03 [0.90; 1.18] | High | . | Serious | Moderate | NMA estimate was used because incoherence was not available. Imprecision was rated as Serious. |
| Kangai Injection+OX | Zhenqi Fuzheng Granules+OX | 0 | . | . | . |  |  |  |  |  | 1.04 [0.86; 1.26] | High | Low | Not serious | Low | 1.04 [0.86; 1.26] | Low | . | Serious | Very low | NMA estimate was used because incoherence was not available. Imprecision was rated as Serious. |
| Kanglaite Injection+OX | Kanglixin Capsules+OX | 0 | . | . | . |  |  |  |  |  | 0.99 [0.81; 1.20] | Moderate | High | Not serious | Moderate | 0.99 [0.81; 1.20] | Moderate | . | Serious | Low | NMA estimate was used because incoherence was not available. Imprecision was rated as Serious. |
| Kanglaite Injection+OX | Lentinan+OX | 0 | . | . | . |  |  |  |  |  | 1.05 [0.88; 1.26] | Moderate | High | Not serious | Moderate | 1.05 [0.88; 1.26] | Moderate | . | Serious | Low | NMA estimate was used because incoherence was not available. Imprecision was rated as Serious. |
| Kanglaite Injection+OX | OX | 4 | 272 | 16.9% | 1.10 [0.95; 1.28] | Serious | Not serious | Not serious | Undetected | Moderate | . |  |  |  |  | 1.10 [0.95; 1.28] | Moderate | . | Serious | Low | NMA estimate was used because incoherence was not available. Imprecision was rated as Serious. |
| Kanglaite Injection+OX | Pingxiao Capsules+OX | 0 | . | . | . |  |  |  |  |  | 1.00 [0.82; 1.21] | Moderate | Low | Not serious | Low | 1.00 [0.82; 1.21] | Low | . | Serious | Very low | NMA estimate was used because incoherence was not available. Imprecision was rated as Serious. |
| Kanglaite Injection+OX | Qizhen Capsule+OX | 0 | . | . | . |  |  |  |  |  | 0.91 [0.72; 1.16] | Moderate | Low | Not serious | Low | 0.91 [0.72; 1.16] | Low | . | Serious | Very low | NMA estimate was used because incoherence was not available. Imprecision was rated as Serious. |
| Kanglaite Injection+OX | Shenfu Injection+OX | 0 | . | . | . |  |  |  |  |  | 1.12 [0.90; 1.41] | Moderate | Low | Not serious | Low | 1.12 [0.90; 1.41] | Low | . | Serious | Very low | NMA estimate was used because incoherence was not available. Imprecision was rated as Serious. |
| Kanglaite Injection+OX | Shengxue Granules+OX | 0 | . | . | . |  |  |  |  |  | 1.42 [1.10; 1.82] | Moderate | Low | Not serious | Low | 1.42 [1.10; 1.82] | Low | . | Not serious | Low | NMA estimate was used because incoherence was not available. Imprecision was rated as Serious. |
| Kanglaite Injection+OX | Shenlian Capsule+OX | 0 | . | . | . |  |  |  |  |  | 0.72 [0.49; 1.06] | Moderate | Low | Not serious | Low | 0.72 [0.49; 1.06] | Low | . | Serious | Very low | NMA estimate was used because incoherence was not available. Imprecision was rated as Serious. |
| Kanglaite Injection+OX | Shenmai Injection+OX | 0 | . | . | . |  |  |  |  |  | 1.05 [0.87; 1.27] | Moderate | High | Not serious | Moderate | 1.05 [0.87; 1.27] | Moderate | . | Serious | Low | NMA estimate was used because incoherence was not available. Imprecision was rated as Serious. |
| Kanglaite Injection+OX | Shenqi Fuzheng Injection+OX | 0 | . | . | . |  |  |  |  |  | 0.99 [0.85; 1.16] | Moderate | Moderate | Not serious | Moderate | 0.99 [0.85; 1.16] | Moderate | . | Serious | Low | NMA estimate was used because incoherence was not available. Imprecision was rated as Serious. |
| Kanglaite Injection+OX | Xiaoaiping Injection+OX | 0 | . | . | . |  |  |  |  |  | 0.95 [0.79; 1.13] | Moderate | Moderate | Not serious | Moderate | 0.95 [0.79; 1.13] | Moderate | . | Serious | Low | NMA estimate was used because incoherence was not available. Imprecision was rated as Serious. |
| Kanglaite Injection+OX | Xihuang Capsules+OX | 0 | . | . | . |  |  |  |  |  | 0.79 [0.60; 1.04] | Moderate | Low | Not serious | Low | 0.79 [0.60; 1.04] | Low | . | Serious | Very low | NMA estimate was used because incoherence was not available. Imprecision was rated as Serious. |
| Kanglaite Injection+OX | Ya Dan Zi Oil Emulsion Injection+OX | 0 | . | . | . |  |  |  |  |  | 0.96 [0.81; 1.13] | Moderate | Moderate | Not serious | Moderate | 0.96 [0.81; 1.13] | Moderate | . | Serious | Low | NMA estimate was used because incoherence was not available. Imprecision was rated as Serious. |
| Kanglaite Injection+OX | Yangzheng Xiaoji Capsules+OX | 0 | . | . | . |  |  |  |  |  | 0.94 [0.78; 1.14] | Moderate | High | Not serious | Moderate | 0.94 [0.78; 1.14] | Moderate | . | Serious | Low | NMA estimate was used because incoherence was not available. Imprecision was rated as Serious. |
| Kanglaite Injection+OX | Zhenqi Fuzheng Granules+OX | 0 | . | . | . |  |  |  |  |  | 0.95 [0.75; 1.20] | Moderate | Low | Not serious | Low | 0.95 [0.75; 1.20] | Low | . | Serious | Very low | NMA estimate was used because incoherence was not available. Imprecision was rated as Serious. |
| Kanglixin Capsules+OX | Lentinan+OX | 0 | . | . | . |  |  |  |  |  | 1.07 [0.91; 1.25] | High | High | Not serious | High | 1.07 [0.91; 1.25] | High | . | Serious | Moderate | NMA estimate was used because incoherence was not available. Imprecision was rated as Serious. |
| Kanglixin Capsules+OX | OX | 2 | 120 | 55.2% | 1.11 [0.98; 1.26] | Not serious | Not serious | Not serious | Undetected | High | . |  |  |  |  | 1.11 [0.98; 1.26] | High | . | Serious | Moderate | NMA estimate was used because incoherence was not available. Imprecision was rated as Serious. |
| Kanglixin Capsules+OX | Pingxiao Capsules+OX | 0 | . | . | . |  |  |  |  |  | 1.01 [0.84; 1.21] | High | Low | Not serious | Low | 1.01 [0.84; 1.21] | Low | . | Serious | Very low | NMA estimate was used because incoherence was not available. Imprecision was rated as Serious. |
| Kanglixin Capsules+OX | Qizhen Capsule+OX | 0 | . | . | . |  |  |  |  |  | 0.92 [0.74; 1.16] | High | Low | Not serious | Low | 0.92 [0.74; 1.16] | Low | . | Serious | Very low | NMA estimate was used because incoherence was not available. Imprecision was rated as Serious. |
| Kanglixin Capsules+OX | Shenfu Injection+OX | 0 | . | . | . |  |  |  |  |  | 1.14 [0.92; 1.40] | High | Low | Not serious | Low | 1.14 [0.92; 1.40] | Low | . | Serious | Very low | NMA estimate was used because incoherence was not available. Imprecision was rated as Serious. |
| Kanglixin Capsules+OX | Shengxue Granules+OX | 0 | . | . | . |  |  |  |  |  | 1.43 [1.13; 1.82] | High | Low | Not serious | Low | 1.43 [1.13; 1.82] | Low | . | Not serious | Low | NMA estimate was used because incoherence was not available. Imprecision was rated as Serious. |
| Kanglixin Capsules+OX | Shenlian Capsule+OX | 0 | . | . | . |  |  |  |  |  | 0.73 [0.50; 1.06] | High | Low | Not serious | Low | 0.73 [0.50; 1.06] | Low | . | Serious | Very low | NMA estimate was used because incoherence was not available. Imprecision was rated as Serious. |
| Kanglixin Capsules+OX | Shenmai Injection+OX | 0 | . | . | . |  |  |  |  |  | 1.07 [0.90; 1.26] | High | High | Not serious | High | 1.07 [0.90; 1.26] | High | . | Serious | Moderate | NMA estimate was used because incoherence was not available. Imprecision was rated as Serious. |
| Kanglixin Capsules+OX | Shenqi Fuzheng Injection+OX | 0 | . | . | . |  |  |  |  |  | 1.00 [0.88; 1.15] | High | Moderate | Not serious | Moderate | 1.00 [0.88; 1.15] | Moderate | . | Serious | Low | NMA estimate was used because incoherence was not available. Imprecision was rated as Serious. |
| Kanglixin Capsules+OX | Xiaoaiping Injection+OX | 0 | . | . | . |  |  |  |  |  | 0.96 [0.82; 1.12] | High | Moderate | Not serious | Moderate | 0.96 [0.82; 1.12] | Moderate | . | Serious | Low | NMA estimate was used because incoherence was not available. Imprecision was rated as Serious. |
| Kanglixin Capsules+OX | Xihuang Capsules+OX | 0 | . | . | . |  |  |  |  |  | 0.80 [0.61; 1.04] | High | Low | Not serious | Low | 0.80 [0.61; 1.04] | Low | . | Serious | Very low | NMA estimate was used because incoherence was not available. Imprecision was rated as Serious. |
| Kanglixin Capsules+OX | Ya Dan Zi Oil Emulsion Injection+OX | 0 | . | . | . |  |  |  |  |  | 0.97 [0.83; 1.12] | High | Moderate | Not serious | Moderate | 0.97 [0.83; 1.12] | Moderate | . | Serious | Low | NMA estimate was used because incoherence was not available. Imprecision was rated as Serious. |
| Kanglixin Capsules+OX | Yangzheng Xiaoji Capsules+OX | 0 | . | . | . |  |  |  |  |  | 0.95 [0.80; 1.13] | High | High | Not serious | High | 0.95 [0.80; 1.13] | High | . | Serious | Moderate | NMA estimate was used because incoherence was not available. Imprecision was rated as Serious. |
| Kanglixin Capsules+OX | Zhenqi Fuzheng Granules+OX | 0 | . | . | . |  |  |  |  |  | 0.96 [0.77; 1.20] | High | Low | Not serious | Low | 0.96 [0.77; 1.20] | Low | . | Serious | Very low | NMA estimate was used because incoherence was not available. Imprecision was rated as Serious. |
| Lentinan+OX | OX | 4 | 357 | 29.2% | 1.04 [0.95; 1.15] | Not serious | Not serious | Not serious | Undetected | High | . |  |  |  |  | 1.04 [0.95; 1.15] | High | . | Serious | Moderate | NMA estimate was used because incoherence was not available. Imprecision was rated as Serious. |
| Lentinan+OX | Pingxiao Capsules+OX | 0 | . | . | . |  |  |  |  |  | 0.95 [0.80; 1.11] | High | Low | Not serious | Low | 0.95 [0.80; 1.11] | Low | . | Serious | Very low | NMA estimate was used because incoherence was not available. Imprecision was rated as Serious. |
| Lentinan+OX | Qizhen Capsule+OX | 0 | . | . | . |  |  |  |  |  | 0.86 [0.70; 1.07] | High | Low | Not serious | Low | 0.86 [0.70; 1.07] | Low | . | Serious | Very low | NMA estimate was used because incoherence was not available. Imprecision was rated as Serious. |
| Lentinan+OX | Shenfu Injection+OX | 0 | . | . | . |  |  |  |  |  | 1.06 [0.87; 1.30] | High | Low | Not serious | Low | 1.06 [0.87; 1.30] | Low | . | Serious | Very low | NMA estimate was used because incoherence was not available. Imprecision was rated as Serious. |
| Lentinan+OX | Shengxue Granules+OX | 0 | . | . | . |  |  |  |  |  | 1.34 [1.07; 1.68] | High | Low | Not serious | Low | 1.34 [1.07; 1.68] | Low | . | Not serious | Low | NMA estimate was used because incoherence was not available. Imprecision was rated as Serious. |
| Lentinan+OX | Shenlian Capsule+OX | 0 | . | . | . |  |  |  |  |  | 0.68 [0.47; 0.99] | High | Low | Not serious | Low | 0.68 [0.47; 0.99] | Low | . | Serious | Very low | NMA estimate was used because incoherence was not available. Imprecision was rated as Serious. |
| Lentinan+OX | Shenmai Injection+OX | 0 | . | . | . |  |  |  |  |  | 1.00 [0.86; 1.16] | High | High | Not serious | High | 1.00 [0.86; 1.16] | High | . | Serious | Moderate | NMA estimate was used because incoherence was not available. Imprecision was rated as Serious. |
| Lentinan+OX | Shenqi Fuzheng Injection+OX | 0 | . | . | . |  |  |  |  |  | 0.94 [0.84; 1.05] | High | Moderate | Not serious | Moderate | 0.94 [0.84; 1.05] | Moderate | . | Serious | Low | NMA estimate was used because incoherence was not available. Imprecision was rated as Serious. |
| Lentinan+OX | Xiaoaiping Injection+OX | 0 | . | . | . |  |  |  |  |  | 0.90 [0.78; 1.03] | High | Moderate | Not serious | Moderate | 0.90 [0.78; 1.03] | Moderate | . | Serious | Low | NMA estimate was used because incoherence was not available. Imprecision was rated as Serious. |
| Lentinan+OX | Xihuang Capsules+OX | 0 | . | . | . |  |  |  |  |  | 0.75 [0.58; 0.97] | High | Low | Not serious | Low | 0.75 [0.58; 0.97] | Low | . | Serious | Very low | NMA estimate was used because incoherence was not available. Imprecision was rated as Serious. |
| Lentinan+OX | Ya Dan Zi Oil Emulsion Injection+OX | 0 | . | . | . |  |  |  |  |  | 0.91 [0.80; 1.03] | High | Moderate | Not serious | Moderate | 0.91 [0.80; 1.03] | Moderate | . | Serious | Low | NMA estimate was used because incoherence was not available. Imprecision was rated as Serious. |
| Lentinan+OX | Yangzheng Xiaoji Capsules+OX | 0 | . | . | . |  |  |  |  |  | 0.89 [0.77; 1.04] | High | High | Not serious | High | 0.89 [0.77; 1.04] | High | . | Serious | Moderate | NMA estimate was used because incoherence was not available. Imprecision was rated as Serious. |
| Lentinan+OX | Zhenqi Fuzheng Granules+OX | 0 | . | . | . |  |  |  |  |  | 0.90 [0.74; 1.10] | High | Low | Not serious | Low | 0.90 [0.74; 1.10] | Low | . | Serious | Very low | NMA estimate was used because incoherence was not available. Imprecision was rated as Serious. |
| Pingxiao Capsules+OX | OX | 3 | 231 | 19.6% | 1.10 [0.97; 1.25] | Very serious | Not serious | Not serious | Undetected | Low | . |  |  |  |  | 1.10 [0.97; 1.25] | Low | . | Serious | Very low | NMA estimate was used because incoherence was not available. Imprecision was rated as Serious. |
| Qizhen Capsule+OX | OX | 1 | 160 | . | 1.21 [1.00; 1.46] | Very serious | Not serious | Not serious | Undetected | Low | . |  |  |  |  | 1.21 [1.00; 1.46] | Low | . | Serious | Very low | NMA estimate was used because incoherence was not available. Imprecision was rated as Serious. |
| Shenfu Injection+OX | OX | 1 | 135 | . | 0.98 [0.83; 1.16] | Very serious | Not serious | Not serious | Undetected | Low | . |  |  |  |  | 0.98 [0.83; 1.16] | Low | . | Serious | Very low | NMA estimate was used because incoherence was not available. Imprecision was rated as Serious. |
| Shengxue Granules+OX | OX | 1 | 71 | . | 0.78 [0.64; 0.95] | Very serious | Not serious | Not serious | Undetected | Low | . |  |  |  |  | 0.78 [0.64; 0.95] | Low | . | Serious | Very low | NMA estimate was used because incoherence was not available. Imprecision was rated as Serious. |
| Shenlian Capsule+OX | OX | 1 | 60 | . | 1.53 [1.07; 2.19] | Very serious | Not serious | Not serious | Undetected | Low | . |  |  |  |  | 1.53 [1.07; 2.19] | Low | . | Not serious | Low | NMA estimate was used because incoherence was not available. Imprecision was rated as Serious. |
| Shenmai Injection+OX | OX | 2 | 173 | 0.0% | 1.04 [0.93; 1.17] | Not serious | Not serious | Not serious | Undetected | High | . |  |  |  |  | 1.04 [0.93; 1.17] | High | . | Serious | Moderate | NMA estimate was used because incoherence was not available. Imprecision was rated as Serious. |
| Shenqi Fuzheng Injection+OX | OX | 22 | 1590 | 0.0% | 1.11 [1.06; 1.16] | Serious | Not serious | Not serious | Not serious | Moderate | . |  |  |  |  | 1.11 [1.06; 1.16] | Moderate | . | Not serious | Moderate | NMA estimate was used because incoherence was not available. Imprecision was rated as Not serious. |
| Xiaoaiping Injection+OX | OX | 6 | 515 | 1.7% | 1.16 [1.06; 1.28] | Serious | Not serious | Not serious | Undetected | Moderate | . |  |  |  |  | 1.16 [1.06; 1.28] | Moderate | . | Not serious | Moderate | NMA estimate was used because incoherence was not available. Imprecision was rated as Serious. |
| Xihuang Capsules+OX | OX | 2 | 164 | 0.0% | 1.39 [1.10; 1.76] | Very serious | Not serious | Not serious | Undetected | Low | . |  |  |  |  | 1.39 [1.10; 1.76] | Low | . | Not serious | Low | NMA estimate was used because incoherence was not available. Imprecision was rated as Serious. |
| Ya Dan Zi Oil Emulsion Injection+OX | OX | 10 | 791 | 15.3% | 1.15 [1.07; 1.24] | Serious | Not serious | Not serious | Not serious | Moderate | . |  |  |  |  | 1.15 [1.07; 1.24] | Moderate | . | Not serious | Moderate | NMA estimate was used because incoherence was not available. Imprecision was rated as Serious. |
| Yangzheng Xiaoji Capsules+OX | OX | 4 | 297 | 0.0% | 1.17 [1.04; 1.31] | Not serious | Not serious | Not serious | Undetected | High | . |  |  |  |  | 1.17 [1.04; 1.31] | High | . | Not serious | High | NMA estimate was used because incoherence was not available. Imprecision was rated as Serious. |
| Zhenqi Fuzheng Granules+OX | OX | 1 | 92 | . | 1.16 [0.97; 1.38] | Very serious | Not serious | Not serious | Undetected | Low | . |  |  |  |  | 1.16 [0.97; 1.38] | Low | . | Serious | Very low | NMA estimate was used because incoherence was not available. Imprecision was rated as Serious. |
| Pingxiao Capsules+OX | Qizhen Capsule+OX | 0 | . | . | . |  |  |  |  |  | 0.91 [0.73; 1.15] | Low | Low | Not serious | Low | 0.91 [0.73; 1.15] | Low | . | Serious | Very low | NMA estimate was used because incoherence was not available. Imprecision was rated as Serious. |
| Pingxiao Capsules+OX | Shenfu Injection+OX | 0 | . | . | . |  |  |  |  |  | 1.13 [0.91; 1.39] | Low | Low | Not serious | Low | 1.13 [0.91; 1.39] | Low | . | Serious | Very low | NMA estimate was used because incoherence was not available. Imprecision was rated as Serious. |
| Pingxiao Capsules+OX | Shengxue Granules+OX | 0 | . | . | . |  |  |  |  |  | 1.42 [1.12; 1.80] | Low | Low | Not serious | Low | 1.42 [1.12; 1.80] | Low | . | Not serious | Low | NMA estimate was used because incoherence was not available. Imprecision was rated as Serious. |
| Pingxiao Capsules+OX | Shenlian Capsule+OX | 0 | . | . | . |  |  |  |  |  | 0.72 [0.49; 1.05] | Low | Low | Not serious | Low | 0.72 [0.49; 1.05] | Low | . | Serious | Very low | NMA estimate was used because incoherence was not available. Imprecision was rated as Serious. |
| Pingxiao Capsules+OX | Shenmai Injection+OX | 0 | . | . | . |  |  |  |  |  | 1.06 [0.89; 1.25] | Low | High | Not serious | Low | 1.06 [0.89; 1.25] | Low | . | Serious | Very low | NMA estimate was used because incoherence was not available. Imprecision was rated as Serious. |
| Pingxiao Capsules+OX | Shenqi Fuzheng Injection+OX | 0 | . | . | . |  |  |  |  |  | 1.00 [0.87; 1.14] | Low | Moderate | Not serious | Low | 1.00 [0.87; 1.14] | Low | . | Serious | Very low | NMA estimate was used because incoherence was not available. Imprecision was rated as Serious. |
| Pingxiao Capsules+OX | Xiaoaiping Injection+OX | 0 | . | . | . |  |  |  |  |  | 0.95 [0.81; 1.12] | Low | Moderate | Not serious | Low | 0.95 [0.81; 1.12] | Low | . | Serious | Very low | NMA estimate was used because incoherence was not available. Imprecision was rated as Serious. |
| Pingxiao Capsules+OX | Xihuang Capsules+OX | 0 | . | . | . |  |  |  |  |  | 0.79 [0.61; 1.04] | Low | Low | Not serious | Low | 0.79 [0.61; 1.04] | Low | . | Serious | Very low | NMA estimate was used because incoherence was not available. Imprecision was rated as Serious. |
| Pingxiao Capsules+OX | Ya Dan Zi Oil Emulsion Injection+OX | 0 | . | . | . |  |  |  |  |  | 0.96 [0.83; 1.11] | Low | Moderate | Not serious | Low | 0.96 [0.83; 1.11] | Low | . | Serious | Very low | NMA estimate was used because incoherence was not available. Imprecision was rated as Serious. |
| Pingxiao Capsules+OX | Yangzheng Xiaoji Capsules+OX | 0 | . | . | . |  |  |  |  |  | 0.95 [0.80; 1.13] | Low | High | Not serious | Low | 0.95 [0.80; 1.13] | Low | . | Serious | Very low | NMA estimate was used because incoherence was not available. Imprecision was rated as Serious. |
| Pingxiao Capsules+OX | Zhenqi Fuzheng Granules+OX | 0 | . | . | . |  |  |  |  |  | 0.95 [0.77; 1.19] | Low | Low | Not serious | Low | 0.95 [0.77; 1.19] | Low | . | Serious | Very low | NMA estimate was used because incoherence was not available. Imprecision was rated as Serious. |
| Qizhen Capsule+OX | Shenfu Injection+OX | 0 | . | . | . |  |  |  |  |  | 1.23 [0.96; 1.58] | Low | Low | Not serious | Low | 1.23 [0.96; 1.58] | Low | . | Serious | Very low | NMA estimate was used because incoherence was not available. Imprecision was rated as Serious. |
| Qizhen Capsule+OX | Shengxue Granules+OX | 0 | . | . | . |  |  |  |  |  | 1.55 [1.18; 2.04] | Low | Low | Not serious | Low | 1.55 [1.18; 2.04] | Low | . | Not serious | Low | NMA estimate was used because incoherence was not available. Imprecision was rated as Serious. |
| Qizhen Capsule+OX | Shenlian Capsule+OX | 0 | . | . | . |  |  |  |  |  | 0.79 [0.53; 1.18] | Low | Low | Not serious | Low | 0.79 [0.53; 1.18] | Low | . | Serious | Very low | NMA estimate was used because incoherence was not available. Imprecision was rated as Serious. |
| Qizhen Capsule+OX | Shenmai Injection+OX | 0 | . | . | . |  |  |  |  |  | 1.16 [0.93; 1.44] | Low | High | Not serious | Low | 1.16 [0.93; 1.44] | Low | . | Serious | Very low | NMA estimate was used because incoherence was not available. Imprecision was rated as Serious. |
| Qizhen Capsule+OX | Shenqi Fuzheng Injection+OX | 0 | . | . | . |  |  |  |  |  | 1.09 [0.90; 1.32] | Low | Moderate | Not serious | Low | 1.09 [0.90; 1.32] | Low | . | Serious | Very low | NMA estimate was used because incoherence was not available. Imprecision was rated as Serious. |
| Qizhen Capsule+OX | Xiaoaiping Injection+OX | 0 | . | . | . |  |  |  |  |  | 1.04 [0.84; 1.28] | Low | Moderate | Not serious | Low | 1.04 [0.84; 1.28] | Low | . | Serious | Very low | NMA estimate was used because incoherence was not available. Imprecision was rated as Serious. |
| Qizhen Capsule+OX | Xihuang Capsules+OX | 0 | . | . | . |  |  |  |  |  | 0.87 [0.64; 1.17] | Low | Low | Not serious | Low | 0.87 [0.64; 1.17] | Low | . | Serious | Very low | NMA estimate was used because incoherence was not available. Imprecision was rated as Serious. |
| Qizhen Capsule+OX | Ya Dan Zi Oil Emulsion Injection+OX | 0 | . | . | . |  |  |  |  |  | 1.05 [0.86; 1.28] | Low | Moderate | Not serious | Low | 1.05 [0.86; 1.28] | Low | . | Serious | Very low | NMA estimate was used because incoherence was not available. Imprecision was rated as Serious. |
| Qizhen Capsule+OX | Yangzheng Xiaoji Capsules+OX | 0 | . | . | . |  |  |  |  |  | 1.03 [0.83; 1.29] | Low | High | Not serious | Low | 1.03 [0.83; 1.29] | Low | . | Serious | Very low | NMA estimate was used because incoherence was not available. Imprecision was rated as Serious. |
| Qizhen Capsule+OX | Zhenqi Fuzheng Granules+OX | 0 | . | . | . |  |  |  |  |  | 1.04 [0.81; 1.35] | Low | Low | Not serious | Low | 1.04 [0.81; 1.35] | Low | . | Serious | Very low | NMA estimate was used because incoherence was not available. Imprecision was rated as Serious. |
| Shenfu Injection+OX | Shengxue Granules+OX | 0 | . | . | . |  |  |  |  |  | 1.26 [0.97; 1.64] | Low | Low | Not serious | Low | 1.26 [0.97; 1.64] | Low | . | Serious | Very low | NMA estimate was used because incoherence was not available. Imprecision was rated as Serious. |
| Shenfu Injection+OX | Shenlian Capsule+OX | 0 | . | . | . |  |  |  |  |  | 0.64 [0.43; 0.95] | Low | Low | Not serious | Low | 0.64 [0.43; 0.95] | Low | . | Serious | Very low | NMA estimate was used because incoherence was not available. Imprecision was rated as Serious. |
| Shenfu Injection+OX | Shenmai Injection+OX | 0 | . | . | . |  |  |  |  |  | 0.94 [0.77; 1.15] | Low | High | Not serious | Low | 0.94 [0.77; 1.15] | Low | . | Serious | Very low | NMA estimate was used because incoherence was not available. Imprecision was rated as Serious. |
| Shenfu Injection+OX | Shenqi Fuzheng Injection+OX | 0 | . | . | . |  |  |  |  |  | 0.88 [0.74; 1.05] | Low | Moderate | Not serious | Low | 0.88 [0.74; 1.05] | Low | . | Serious | Very low | NMA estimate was used because incoherence was not available. Imprecision was rated as Serious. |
| Shenfu Injection+OX | Xiaoaiping Injection+OX | 0 | . | . | . |  |  |  |  |  | 0.84 [0.69; 1.03] | Low | Moderate | Not serious | Low | 0.84 [0.69; 1.03] | Low | . | Serious | Very low | NMA estimate was used because incoherence was not available. Imprecision was rated as Serious. |
| Shenfu Injection+OX | Xihuang Capsules+OX | 0 | . | . | . |  |  |  |  |  | 0.70 [0.53; 0.94] | Low | Low | Not serious | Low | 0.70 [0.53; 0.94] | Low | . | Serious | Very low | NMA estimate was used because incoherence was not available. Imprecision was rated as Serious. |
| Shenfu Injection+OX | Ya Dan Zi Oil Emulsion Injection+OX | 0 | . | . | . |  |  |  |  |  | 0.85 [0.71; 1.03] | Low | Moderate | Not serious | Low | 0.85 [0.71; 1.03] | Low | . | Serious | Very low | NMA estimate was used because incoherence was not available. Imprecision was rated as Serious. |
| Shenfu Injection+OX | Yangzheng Xiaoji Capsules+OX | 0 | . | . | . |  |  |  |  |  | 0.84 [0.68; 1.03] | Low | High | Not serious | Low | 0.84 [0.68; 1.03] | Low | . | Serious | Very low | NMA estimate was used because incoherence was not available. Imprecision was rated as Serious. |
| Shenfu Injection+OX | Zhenqi Fuzheng Granules+OX | 0 | . | . | . |  |  |  |  |  | 0.85 [0.66; 1.08] | Low | Low | Not serious | Low | 0.85 [0.66; 1.08] | Low | . | Serious | Very low | NMA estimate was used because incoherence was not available. Imprecision was rated as Serious. |
| Shengxue Granules+OX | Shenlian Capsule+OX | 0 | . | . | . |  |  |  |  |  | 0.51 [0.34; 0.77] | Low | Low | Not serious | Low | 0.51 [0.34; 0.77] | Low | . | Serious | Very low | NMA estimate was used because incoherence was not available. Imprecision was rated as Serious. |
| Shengxue Granules+OX | Shenmai Injection+OX | 0 | . | . | . |  |  |  |  |  | 0.74 [0.59; 0.94] | Low | High | Not serious | Low | 0.74 [0.59; 0.94] | Low | . | Serious | Very low | NMA estimate was used because incoherence was not available. Imprecision was rated as Serious. |
| Shengxue Granules+OX | Shenqi Fuzheng Injection+OX | 0 | . | . | . |  |  |  |  |  | 0.70 [0.57; 0.86] | Low | Moderate | Not serious | Low | 0.70 [0.57; 0.86] | Low | . | Serious | Very low | NMA estimate was used because incoherence was not available. Imprecision was rated as Serious. |
| Shengxue Granules+OX | Xiaoaiping Injection+OX | 0 | . | . | . |  |  |  |  |  | 0.67 [0.54; 0.84] | Low | Moderate | Not serious | Low | 0.67 [0.54; 0.84] | Low | . | Serious | Very low | NMA estimate was used because incoherence was not available. Imprecision was rated as Serious. |
| Shengxue Granules+OX | Xihuang Capsules+OX | 0 | . | . | . |  |  |  |  |  | 0.56 [0.41; 0.76] | Low | Low | Not serious | Low | 0.56 [0.41; 0.76] | Low | . | Serious | Very low | NMA estimate was used because incoherence was not available. Imprecision was rated as Serious. |
| Shengxue Granules+OX | Ya Dan Zi Oil Emulsion Injection+OX | 0 | . | . | . |  |  |  |  |  | 0.68 [0.55; 0.84] | Low | Moderate | Not serious | Low | 0.68 [0.55; 0.84] | Low | . | Serious | Very low | NMA estimate was used because incoherence was not available. Imprecision was rated as Serious. |
| Shengxue Granules+OX | Yangzheng Xiaoji Capsules+OX | 0 | . | . | . |  |  |  |  |  | 0.67 [0.53; 0.84] | Low | High | Not serious | Low | 0.67 [0.53; 0.84] | Low | . | Serious | Very low | NMA estimate was used because incoherence was not available. Imprecision was rated as Serious. |
| Shengxue Granules+OX | Zhenqi Fuzheng Granules+OX | 0 | . | . | . |  |  |  |  |  | 0.67 [0.51; 0.88] | Low | Low | Not serious | Low | 0.67 [0.51; 0.88] | Low | . | Serious | Very low | NMA estimate was used because incoherence was not available. Imprecision was rated as Serious. |
| Shenlian Capsule+OX | Shenmai Injection+OX | 0 | . | . | . |  |  |  |  |  | 1.46 [1.01; 2.13] | Low | High | Not serious | Low | 1.46 [1.01; 2.13] | Low | . | Not serious | Low | NMA estimate was used because incoherence was not available. Imprecision was rated as Serious. |
| Shenlian Capsule+OX | Shenqi Fuzheng Injection+OX | 0 | . | . | . |  |  |  |  |  | 1.38 [0.96; 1.98] | Low | Moderate | Not serious | Low | 1.38 [0.96; 1.98] | Low | . | Serious | Very low | NMA estimate was used because incoherence was not available. Imprecision was rated as Serious. |
| Shenlian Capsule+OX | Xiaoaiping Injection+OX | 0 | . | . | . |  |  |  |  |  | 1.32 [0.91; 1.91] | Low | Moderate | Not serious | Low | 1.32 [0.91; 1.91] | Low | . | Serious | Very low | NMA estimate was used because incoherence was not available. Imprecision was rated as Serious. |
| Shenlian Capsule+OX | Xihuang Capsules+OX | 0 | . | . | . |  |  |  |  |  | 1.10 [0.72; 1.69] | Low | Low | Not serious | Low | 1.10 [0.72; 1.69] | Low | . | Serious | Very low | NMA estimate was used because incoherence was not available. Imprecision was rated as Serious. |
| Shenlian Capsule+OX | Ya Dan Zi Oil Emulsion Injection+OX | 0 | . | . | . |  |  |  |  |  | 1.33 [0.92; 1.92] | Low | Moderate | Not serious | Low | 1.33 [0.92; 1.92] | Low | . | Serious | Very low | NMA estimate was used because incoherence was not available. Imprecision was rated as Serious. |
| Shenlian Capsule+OX | Yangzheng Xiaoji Capsules+OX | 0 | . | . | . |  |  |  |  |  | 1.31 [0.90; 1.91] | Low | High | Not serious | Low | 1.31 [0.90; 1.91] | Low | . | Serious | Very low | NMA estimate was used because incoherence was not available. Imprecision was rated as Serious. |
| Shenlian Capsule+OX | Zhenqi Fuzheng Granules+OX | 0 | . | . | . |  |  |  |  |  | 1.32 [0.89; 1.97] | Low | Low | Not serious | Low | 1.32 [0.89; 1.97] | Low | . | Serious | Very low | NMA estimate was used because incoherence was not available. Imprecision was rated as Serious. |
| Shenmai Injection+OX | Shenqi Fuzheng Injection+OX | 0 | . | . | . |  |  |  |  |  | 0.94 [0.83; 1.07] | High | Moderate | Not serious | Moderate | 0.94 [0.83; 1.07] | Moderate | . | Serious | Low | NMA estimate was used because incoherence was not available. Imprecision was rated as Serious. |
| Shenmai Injection+OX | Xiaoaiping Injection+OX | 0 | . | . | . |  |  |  |  |  | 0.90 [0.77; 1.04] | High | Moderate | Not serious | Moderate | 0.90 [0.77; 1.04] | Moderate | . | Serious | Low | NMA estimate was used because incoherence was not available. Imprecision was rated as Serious. |
| Shenmai Injection+OX | Xihuang Capsules+OX | 0 | . | . | . |  |  |  |  |  | 0.75 [0.58; 0.97] | High | Low | Not serious | Low | 0.75 [0.58; 0.97] | Low | . | Serious | Very low | NMA estimate was used because incoherence was not available. Imprecision was rated as Serious. |
| Shenmai Injection+OX | Ya Dan Zi Oil Emulsion Injection+OX | 0 | . | . | . |  |  |  |  |  | 0.91 [0.79; 1.04] | High | Moderate | Not serious | Moderate | 0.91 [0.79; 1.04] | Moderate | . | Serious | Low | NMA estimate was used because incoherence was not available. Imprecision was rated as Serious. |
| Shenmai Injection+OX | Yangzheng Xiaoji Capsules+OX | 0 | . | . | . |  |  |  |  |  | 0.90 [0.76; 1.05] | High | High | Not serious | High | 0.90 [0.76; 1.05] | High | . | Serious | Moderate | NMA estimate was used because incoherence was not available. Imprecision was rated as Serious. |
| Shenmai Injection+OX | Zhenqi Fuzheng Granules+OX | 0 | . | . | . |  |  |  |  |  | 0.90 [0.73; 1.11] | High | Low | Not serious | Low | 0.90 [0.73; 1.11] | Low | . | Serious | Very low | NMA estimate was used because incoherence was not available. Imprecision was rated as Serious. |
| Shenqi Fuzheng Injection+OX | Xiaoaiping Injection+OX | 0 | . | . | . |  |  |  |  |  | 0.95 [0.86; 1.06] | Moderate | Moderate | Not serious | Moderate | 0.95 [0.86; 1.06] | Moderate | . | Serious | Low | NMA estimate was used because incoherence was not available. Imprecision was rated as Serious. |
| Shenqi Fuzheng Injection+OX | Xihuang Capsules+OX | 0 | . | . | . |  |  |  |  |  | 0.80 [0.63; 1.01] | Moderate | Low | Not serious | Low | 0.80 [0.63; 1.01] | Low | . | Serious | Very low | NMA estimate was used because incoherence was not available. Imprecision was rated as Serious. |
| Shenqi Fuzheng Injection+OX | Ya Dan Zi Oil Emulsion Injection+OX | 0 | . | . | . |  |  |  |  |  | 0.96 [0.88; 1.06] | Moderate | Moderate | Not serious | Moderate | 0.96 [0.88; 1.06] | Moderate | . | Serious | Low | NMA estimate was used because incoherence was not available. Imprecision was rated as Serious. |
| Shenqi Fuzheng Injection+OX | Yangzheng Xiaoji Capsules+OX | 0 | . | . | . |  |  |  |  |  | 0.95 [0.84; 1.08] | Moderate | High | Not serious | Moderate | 0.95 [0.84; 1.08] | Moderate | . | Serious | Low | NMA estimate was used because incoherence was not available. Imprecision was rated as Serious. |
| Shenqi Fuzheng Injection+OX | Zhenqi Fuzheng Granules+OX | 0 | . | . | . |  |  |  |  |  | 0.96 [0.80; 1.15] | Moderate | Low | Not serious | Low | 0.96 [0.80; 1.15] | Low | . | Serious | Very low | NMA estimate was used because incoherence was not available. Imprecision was rated as Serious. |
| Xiaoaiping Injection+OX | Xihuang Capsules+OX | 0 | . | . | . |  |  |  |  |  | 0.84 [0.65; 1.08] | Moderate | Low | Not serious | Low | 0.84 [0.65; 1.08] | Low | . | Serious | Very low | NMA estimate was used because incoherence was not available. Imprecision was rated as Serious. |
| Xiaoaiping Injection+OX | Ya Dan Zi Oil Emulsion Injection+OX | 0 | . | . | . |  |  |  |  |  | 1.01 [0.89; 1.14] | Moderate | Moderate | Not serious | Moderate | 1.01 [0.89; 1.14] | Moderate | . | Serious | Low | NMA estimate was used because incoherence was not available. Imprecision was rated as Serious. |
| Xiaoaiping Injection+OX | Yangzheng Xiaoji Capsules+OX | 0 | . | . | . |  |  |  |  |  | 1.00 [0.86; 1.16] | Moderate | High | Not serious | Moderate | 1.00 [0.86; 1.16] | Moderate | . | Serious | Low | NMA estimate was used because incoherence was not available. Imprecision was rated as Serious. |
| Xiaoaiping Injection+OX | Zhenqi Fuzheng Granules+OX | 0 | . | . | . |  |  |  |  |  | 1.00 [0.82; 1.23] | Moderate | Low | Not serious | Low | 1.00 [0.82; 1.23] | Low | . | Serious | Very low | NMA estimate was used because incoherence was not available. Imprecision was rated as Serious. |
| Xihuang Capsules+OX | Ya Dan Zi Oil Emulsion Injection+OX | 0 | . | . | . |  |  |  |  |  | 1.21 [0.95; 1.55] | Low | Moderate | Not serious | Low | 1.21 [0.95; 1.55] | Low | . | Serious | Very low | NMA estimate was used because incoherence was not available. Imprecision was rated as Serious. |
| Xihuang Capsules+OX | Yangzheng Xiaoji Capsules+OX | 0 | . | . | . |  |  |  |  |  | 1.19 [0.92; 1.55] | Low | High | Not serious | Low | 1.19 [0.92; 1.55] | Low | . | Serious | Very low | NMA estimate was used because incoherence was not available. Imprecision was rated as Serious. |
| Xihuang Capsules+OX | Zhenqi Fuzheng Granules+OX | 0 | . | . | . |  |  |  |  |  | 1.20 [0.90; 1.61] | Low | Low | Not serious | Low | 1.20 [0.90; 1.61] | Low | . | Serious | Very low | NMA estimate was used because incoherence was not available. Imprecision was rated as Serious. |
| Ya Dan Zi Oil Emulsion Injection+OX | Yangzheng Xiaoji Capsules+OX | 0 | . | . | . |  |  |  |  |  | 0.99 [0.86; 1.13] | Moderate | High | Not serious | Moderate | 0.99 [0.86; 1.13] | Moderate | . | Serious | Low | NMA estimate was used because incoherence was not available. Imprecision was rated as Serious. |
| Ya Dan Zi Oil Emulsion Injection+OX | Zhenqi Fuzheng Granules+OX | 0 | . | . | . |  |  |  |  |  | 0.99 [0.82; 1.20] | Moderate | Low | Not serious | Low | 0.99 [0.82; 1.20] | Low | . | Serious | Very low | NMA estimate was used because incoherence was not available. Imprecision was rated as Serious. |
| Yangzheng Xiaoji Capsules+OX | Zhenqi Fuzheng Granules+OX | 0 | . | . | . |  |  |  |  |  | 1.01 [0.81; 1.25] | High | Low | Not serious | Low | 1.01 [0.81; 1.25] | Low | . | Serious | Very low | NMA estimate was used because incoherence was not available. Imprecision was rated as Serious. |

1. Objective response rate

| **Arm_1** | **Arm_2** | **No_of_study** | **Sample_size** | **I2** | **Direct_estimate** | **ROB** | **Inconsistency** | **Indirectness** | **Publication_bias** | **Direct_rating_without_imprecision** | **Indirect_estimate** | **Certainty_of_evidence_for_arm1** | **Certainty_of_evidence_for_arm2** | **Intransitivity** | **Indirect_rating_without_imprecision** | **Network_meta_analysis** | **Higher_rating_of_direct_and_indirect_without_imprecision** | **Incoherence** | **NMA_Imprecision** | **Final_network_rating** | **Final_rating_reason** |
| --- | --- | --- | --- | --- | --- | --- | --- | --- | --- | --- | --- | --- | --- | --- | --- | --- | --- | --- | --- | --- | --- |
| Aidi Injection+OX | Astragalus Polysaccharides+OX | 0 | . | . | . |  |  |  |  |  | 0.82 [0.57; 1.19] | Low | Low | Not serious | Low | 0.82 [0.57; 1.19] | Low | . | Serious | Very low | NMA estimate was used because incoherence was not available. Imprecision was rated as Serious. |
| Aidi Injection+OX | Astragalus preparations+OX | 0 | . | . | . |  |  |  |  |  | 1.05 [0.86; 1.29] | Low | Low | Not serious | Low | 1.05 [0.86; 1.29] | Low | . | Serious | Very low | NMA estimate was used because incoherence was not available. Imprecision was rated as Serious. |
| Aidi Injection+OX | Compound Kushen Injection+OX | 0 | . | . | . |  |  |  |  |  | 0.88 [0.76; 1.02] | Low | Very low | Not serious | Very low | 0.88 [0.76; 1.02] | Very low | . | Serious | Very low | NMA estimate was used because incoherence was not available. Imprecision was rated as Serious. |
| Aidi Injection+OX | Compound Mylabris preparations+OX | 0 | . | . | . |  |  |  |  |  | 0.79 [0.60; 1.05] | Low | Low | Not serious | Low | 0.79 [0.60; 1.05] | Low | . | Serious | Very low | NMA estimate was used because incoherence was not available. Imprecision was rated as Serious. |
| Aidi Injection+OX | Ginseng Polysaccharide Injection+OX | 0 | . | . | . |  |  |  |  |  | 1.10 [0.73; 1.65] | Low | High | Not serious | Low | 1.10 [0.73; 1.65] | Low | . | Serious | Very low | NMA estimate was used because incoherence was not available. Imprecision was rated as Serious. |
| Aidi Injection+OX | Huachansu preparations+OX | 0 | . | . | . |  |  |  |  |  | 1.00 [0.87; 1.16] | Low | High | Not serious | Low | 1.00 [0.87; 1.16] | Low | . | Serious | Very low | NMA estimate was used because incoherence was not available. Imprecision was rated as Serious. |
| Aidi Injection+OX | Huai Er Granules+OX | 0 | . | . | . |  |  |  |  |  | 1.12 [0.56; 2.26] | Low | Low | Not serious | Low | 1.12 [0.56; 2.26] | Low | . | Serious | Very low | NMA estimate was used because incoherence was not available. Imprecision was rated as Serious. |
| Aidi Injection+OX | Jinlong Capsules+OX | 0 | . | . | . |  |  |  |  |  | 1.01 [0.71; 1.44] | Low | High | Not serious | Low | 1.01 [0.71; 1.44] | Low | . | Serious | Very low | NMA estimate was used because incoherence was not available. Imprecision was rated as Serious. |
| Aidi Injection+OX | Kangai Injection+OX | 0 | . | . | . |  |  |  |  |  | 0.82 [0.68; 0.99] | Low | Moderate | Not serious | Low | 0.82 [0.68; 0.99] | Low | . | Serious | Very low | NMA estimate was used because incoherence was not available. Imprecision was rated as Serious. |
| Aidi Injection+OX | Kanglaite Injection+OX | 0 | . | . | . |  |  |  |  |  | 0.98 [0.73; 1.32] | Low | Moderate | Not serious | Low | 0.98 [0.73; 1.32] | Low | . | Serious | Very low | NMA estimate was used because incoherence was not available. Imprecision was rated as Serious. |
| Aidi Injection+OX | Kanglixin Capsules+OX | 0 | . | . | . |  |  |  |  |  | 0.97 [0.75; 1.25] | Low | High | Not serious | Low | 0.97 [0.75; 1.25] | Low | . | Serious | Very low | NMA estimate was used because incoherence was not available. Imprecision was rated as Serious. |
| Aidi Injection+OX | Lentinan+OX | 0 | . | . | . |  |  |  |  |  | 1.29 [1.06; 1.55] | Low | High | Not serious | Low | 1.29 [1.06; 1.55] | Low | . | Not serious | Low | NMA estimate was used because incoherence was not available. Imprecision was rated as Serious. |
| Aidi Injection+OX | OX | 18 | 1353 | 5.7% | 1.23 [1.12; 1.36] | Very serious | Not serious | Not serious | Not serious | Low | . |  |  |  |  | 1.23 [1.12; 1.36] | Low | . | Not serious | Low | NMA estimate was used because incoherence was not available. Imprecision was rated as Not serious. |
| Aidi Injection+OX | Pingxiao Capsules+OX | 0 | . | . | . |  |  |  |  |  | 0.80 [0.59; 1.09] | Low | Low | Not serious | Low | 0.80 [0.59; 1.09] | Low | . | Serious | Very low | NMA estimate was used because incoherence was not available. Imprecision was rated as Serious. |
| Aidi Injection+OX | Qizhen Capsule+OX | 0 | . | . | . |  |  |  |  |  | 0.87 [0.65; 1.17] | Low | Low | Not serious | Low | 0.87 [0.65; 1.17] | Low | . | Serious | Very low | NMA estimate was used because incoherence was not available. Imprecision was rated as Serious. |
| Aidi Injection+OX | Shenfu Injection+OX | 0 | . | . | . |  |  |  |  |  | 1.30 [0.89; 1.90] | Low | Low | Not serious | Low | 1.30 [0.89; 1.90] | Low | . | Serious | Very low | NMA estimate was used because incoherence was not available. Imprecision was rated as Serious. |
| Aidi Injection+OX | Shengxue Granules+OX | 0 | . | . | . |  |  |  |  |  | 1.05 [0.61; 1.80] | Low | Low | Not serious | Low | 1.05 [0.61; 1.80] | Low | . | Serious | Very low | NMA estimate was used because incoherence was not available. Imprecision was rated as Serious. |
| Aidi Injection+OX | Shenmai Injection+OX | 0 | . | . | . |  |  |  |  |  | 0.95 [0.70; 1.28] | Low | High | Not serious | Low | 0.95 [0.70; 1.28] | Low | . | Serious | Very low | NMA estimate was used because incoherence was not available. Imprecision was rated as Serious. |
| Aidi Injection+OX | Shenqi Fuzheng Injection+OX | 0 | . | . | . |  |  |  |  |  | 0.97 [0.84; 1.12] | Low | Low | Not serious | Low | 0.97 [0.84; 1.12] | Low | . | Serious | Very low | NMA estimate was used because incoherence was not available. Imprecision was rated as Serious. |
| Aidi Injection+OX | Weimaining Capsules+OX | 0 | . | . | . |  |  |  |  |  | 0.99 [0.45; 2.18] | Low | High | Not serious | Low | 0.99 [0.45; 2.18] | Low | . | Serious | Very low | NMA estimate was used because incoherence was not available. Imprecision was rated as Serious. |
| Aidi Injection+OX | Xiaoaiping Injection+OX | 0 | . | . | . |  |  |  |  |  | 0.93 [0.77; 1.11] | Low | Moderate | Not serious | Low | 0.93 [0.77; 1.11] | Low | . | Serious | Very low | NMA estimate was used because incoherence was not available. Imprecision was rated as Serious. |
| Aidi Injection+OX | Xihuang Capsules+OX | 0 | . | . | . |  |  |  |  |  | 0.78 [0.55; 1.10] | Low | Low | Not serious | Low | 0.78 [0.55; 1.10] | Low | . | Serious | Very low | NMA estimate was used because incoherence was not available. Imprecision was rated as Serious. |
| Aidi Injection+OX | Ya Dan Zi Oil Emulsion Injection+OX | 0 | . | . | . |  |  |  |  |  | 0.93 [0.80; 1.09] | Low | Low | Not serious | Low | 0.93 [0.80; 1.09] | Low | . | Serious | Very low | NMA estimate was used because incoherence was not available. Imprecision was rated as Serious. |
| Aidi Injection+OX | Yangzheng Xiaoji Capsules+OX | 0 | . | . | . |  |  |  |  |  | 0.91 [0.69; 1.19] | Low | High | Not serious | Low | 0.91 [0.69; 1.19] | Low | . | Serious | Very low | NMA estimate was used because incoherence was not available. Imprecision was rated as Serious. |
| Astragalus Polysaccharides+OX | Astragalus preparations+OX | 0 | . | . | . |  |  |  |  |  | 1.27 [0.86; 1.89] | Low | Low | Not serious | Low | 1.27 [0.86; 1.89] | Low | . | Serious | Very low | NMA estimate was used because incoherence was not available. Imprecision was rated as Serious. |
| Astragalus Polysaccharides+OX | Compound Kushen Injection+OX | 0 | . | . | . |  |  |  |  |  | 1.07 [0.74; 1.54] | Low | Very low | Not serious | Very low | 1.07 [0.74; 1.54] | Very low | . | Serious | Very low | NMA estimate was used because incoherence was not available. Imprecision was rated as Serious. |
| Astragalus Polysaccharides+OX | Compound Mylabris preparations+OX | 0 | . | . | . |  |  |  |  |  | 0.96 [0.62; 1.49] | Low | Low | Not serious | Low | 0.96 [0.62; 1.49] | Low | . | Serious | Very low | NMA estimate was used because incoherence was not available. Imprecision was rated as Serious. |
| Astragalus Polysaccharides+OX | Ginseng Polysaccharide Injection+OX | 0 | . | . | . |  |  |  |  |  | 1.33 [0.78; 2.26] | Low | High | Not serious | Low | 1.33 [0.78; 2.26] | Low | . | Serious | Very low | NMA estimate was used because incoherence was not available. Imprecision was rated as Serious. |
| Astragalus Polysaccharides+OX | Huachansu preparations+OX | 0 | . | . | . |  |  |  |  |  | 1.22 [0.84; 1.76] | Low | High | Not serious | Low | 1.22 [0.84; 1.76] | Low | . | Serious | Very low | NMA estimate was used because incoherence was not available. Imprecision was rated as Serious. |
| Astragalus Polysaccharides+OX | Huai Er Granules+OX | 0 | . | . | . |  |  |  |  |  | 1.36 [0.63; 2.96] | Low | Low | Not serious | Low | 1.36 [0.63; 2.96] | Low | . | Serious | Very low | NMA estimate was used because incoherence was not available. Imprecision was rated as Serious. |
| Astragalus Polysaccharides+OX | Jinlong Capsules+OX | 0 | . | . | . |  |  |  |  |  | 1.22 [0.75; 2.00] | Low | High | Not serious | Low | 1.22 [0.75; 2.00] | Low | . | Serious | Very low | NMA estimate was used because incoherence was not available. Imprecision was rated as Serious. |
| Astragalus Polysaccharides+OX | Kangai Injection+OX | 0 | . | . | . |  |  |  |  |  | 0.99 [0.67; 1.46] | Low | Moderate | Not serious | Low | 0.99 [0.67; 1.46] | Low | . | Serious | Very low | NMA estimate was used because incoherence was not available. Imprecision was rated as Serious. |
| Astragalus Polysaccharides+OX | Kanglaite Injection+OX | 0 | . | . | . |  |  |  |  |  | 1.19 [0.76; 1.87] | Low | Moderate | Not serious | Low | 1.19 [0.76; 1.87] | Low | . | Serious | Very low | NMA estimate was used because incoherence was not available. Imprecision was rated as Serious. |
| Astragalus Polysaccharides+OX | Kanglixin Capsules+OX | 0 | . | . | . |  |  |  |  |  | 1.18 [0.77; 1.80] | Low | High | Not serious | Low | 1.18 [0.77; 1.80] | Low | . | Serious | Very low | NMA estimate was used because incoherence was not available. Imprecision was rated as Serious. |
| Astragalus Polysaccharides+OX | Lentinan+OX | 0 | . | . | . |  |  |  |  |  | 1.56 [1.06; 2.30] | Low | High | Not serious | Low | 1.56 [1.06; 2.30] | Low | . | Not serious | Low | NMA estimate was used because incoherence was not available. Imprecision was rated as Serious. |
| Astragalus Polysaccharides+OX | OX | 2 | 151 | 0.0% | 1.50 [1.05; 2.13] | Very serious | Not serious | Not serious | Undetected | Low | . |  |  |  |  | 1.50 [1.05; 2.13] | Low | . | Not serious | Low | NMA estimate was used because incoherence was not available. Imprecision was rated as Serious. |
| Astragalus Polysaccharides+OX | Pingxiao Capsules+OX | 0 | . | . | . |  |  |  |  |  | 0.97 [0.61; 1.54] | Low | Low | Not serious | Low | 0.97 [0.61; 1.54] | Low | . | Serious | Very low | NMA estimate was used because incoherence was not available. Imprecision was rated as Serious. |
| Astragalus Polysaccharides+OX | Qizhen Capsule+OX | 0 | . | . | . |  |  |  |  |  | 1.05 [0.67; 1.65] | Low | Low | Not serious | Low | 1.05 [0.67; 1.65] | Low | . | Serious | Very low | NMA estimate was used because incoherence was not available. Imprecision was rated as Serious. |
| Astragalus Polysaccharides+OX | Shenfu Injection+OX | 0 | . | . | . |  |  |  |  |  | 1.57 [0.95; 2.62] | Low | Low | Not serious | Low | 1.57 [0.95; 2.62] | Low | . | Serious | Very low | NMA estimate was used because incoherence was not available. Imprecision was rated as Serious. |
| Astragalus Polysaccharides+OX | Shengxue Granules+OX | 0 | . | . | . |  |  |  |  |  | 1.27 [0.67; 2.40] | Low | Low | Not serious | Low | 1.27 [0.67; 2.40] | Low | . | Serious | Very low | NMA estimate was used because incoherence was not available. Imprecision was rated as Serious. |
| Astragalus Polysaccharides+OX | Shenmai Injection+OX | 0 | . | . | . |  |  |  |  |  | 1.15 [0.73; 1.81] | Low | High | Not serious | Low | 1.15 [0.73; 1.81] | Low | . | Serious | Very low | NMA estimate was used because incoherence was not available. Imprecision was rated as Serious. |
| Astragalus Polysaccharides+OX | Shenqi Fuzheng Injection+OX | 0 | . | . | . |  |  |  |  |  | 1.17 [0.81; 1.70] | Low | Low | Not serious | Low | 1.17 [0.81; 1.70] | Low | . | Serious | Very low | NMA estimate was used because incoherence was not available. Imprecision was rated as Serious. |
| Astragalus Polysaccharides+OX | Weimaining Capsules+OX | 0 | . | . | . |  |  |  |  |  | 1.20 [0.51; 2.83] | Low | High | Not serious | Low | 1.20 [0.51; 2.83] | Low | . | Serious | Very low | NMA estimate was used because incoherence was not available. Imprecision was rated as Serious. |
| Astragalus Polysaccharides+OX | Xiaoaiping Injection+OX | 0 | . | . | . |  |  |  |  |  | 1.12 [0.77; 1.65] | Low | Moderate | Not serious | Low | 1.12 [0.77; 1.65] | Low | . | Serious | Very low | NMA estimate was used because incoherence was not available. Imprecision was rated as Serious. |
| Astragalus Polysaccharides+OX | Xihuang Capsules+OX | 0 | . | . | . |  |  |  |  |  | 0.95 [0.58; 1.54] | Low | Low | Not serious | Low | 0.95 [0.58; 1.54] | Low | . | Serious | Very low | NMA estimate was used because incoherence was not available. Imprecision was rated as Serious. |
| Astragalus Polysaccharides+OX | Ya Dan Zi Oil Emulsion Injection+OX | 0 | . | . | . |  |  |  |  |  | 1.13 [0.78; 1.64] | Low | Low | Not serious | Low | 1.13 [0.78; 1.64] | Low | . | Serious | Very low | NMA estimate was used because incoherence was not available. Imprecision was rated as Serious. |
| Astragalus Polysaccharides+OX | Yangzheng Xiaoji Capsules+OX | 0 | . | . | . |  |  |  |  |  | 1.10 [0.71; 1.70] | Low | High | Not serious | Low | 1.10 [0.71; 1.70] | Low | . | Serious | Very low | NMA estimate was used because incoherence was not available. Imprecision was rated as Serious. |
| Astragalus preparations+OX | Compound Kushen Injection+OX | 0 | . | . | . |  |  |  |  |  | 0.84 [0.68; 1.03] | Low | Very low | Not serious | Very low | 0.84 [0.68; 1.03] | Very low | . | Serious | Very low | NMA estimate was used because incoherence was not available. Imprecision was rated as Serious. |
| Astragalus preparations+OX | Compound Mylabris preparations+OX | 0 | . | . | . |  |  |  |  |  | 0.75 [0.55; 1.03] | Low | Low | Not serious | Low | 0.75 [0.55; 1.03] | Low | . | Serious | Very low | NMA estimate was used because incoherence was not available. Imprecision was rated as Serious. |
| Astragalus preparations+OX | Ginseng Polysaccharide Injection+OX | 0 | . | . | . |  |  |  |  |  | 1.05 [0.68; 1.61] | Low | High | Not serious | Low | 1.05 [0.68; 1.61] | Low | . | Serious | Very low | NMA estimate was used because incoherence was not available. Imprecision was rated as Serious. |
| Astragalus preparations+OX | Huachansu preparations+OX | 0 | . | . | . |  |  |  |  |  | 0.96 [0.78; 1.17] | Low | High | Not serious | Low | 0.96 [0.78; 1.17] | Low | . | Serious | Very low | NMA estimate was used because incoherence was not available. Imprecision was rated as Serious. |
| Astragalus preparations+OX | Huai Er Granules+OX | 0 | . | . | . |  |  |  |  |  | 1.07 [0.52; 2.18] | Low | Low | Not serious | Low | 1.07 [0.52; 2.18] | Low | . | Serious | Very low | NMA estimate was used because incoherence was not available. Imprecision was rated as Serious. |
| Astragalus preparations+OX | Jinlong Capsules+OX | 0 | . | . | . |  |  |  |  |  | 0.96 [0.66; 1.41] | Low | High | Not serious | Low | 0.96 [0.66; 1.41] | Low | . | Serious | Very low | NMA estimate was used because incoherence was not available. Imprecision was rated as Serious. |
| Astragalus preparations+OX | Kangai Injection+OX | 0 | . | . | . |  |  |  |  |  | 0.78 [0.61; 0.99] | Low | Moderate | Not serious | Low | 0.78 [0.61; 0.99] | Low | . | Serious | Very low | NMA estimate was used because incoherence was not available. Imprecision was rated as Serious. |
| Astragalus preparations+OX | Kanglaite Injection+OX | 0 | . | . | . |  |  |  |  |  | 0.94 [0.67; 1.30] | Low | Moderate | Not serious | Low | 0.94 [0.67; 1.30] | Low | . | Serious | Very low | NMA estimate was used because incoherence was not available. Imprecision was rated as Serious. |
| Astragalus preparations+OX | Kanglixin Capsules+OX | 0 | . | . | . |  |  |  |  |  | 0.92 [0.69; 1.24] | Low | High | Not serious | Low | 0.92 [0.69; 1.24] | Low | . | Serious | Very low | NMA estimate was used because incoherence was not available. Imprecision was rated as Serious. |
| Astragalus preparations+OX | Lentinan+OX | 0 | . | . | . |  |  |  |  |  | 1.23 [0.97; 1.56] | Low | High | Not serious | Low | 1.23 [0.97; 1.56] | Low | . | Serious | Very low | NMA estimate was used because incoherence was not available. Imprecision was rated as Serious. |
| Astragalus preparations+OX | OX | 3 | 285 | 67.9% | 1.18 [0.99; 1.40] | Very serious | Not serious | Not serious | Undetected | Low | . |  |  |  |  | 1.18 [0.99; 1.40] | Low | . | Serious | Very low | NMA estimate was used because incoherence was not available. Imprecision was rated as Serious. |
| Astragalus preparations+OX | Pingxiao Capsules+OX | 0 | . | . | . |  |  |  |  |  | 0.76 [0.54; 1.08] | Low | Low | Not serious | Low | 0.76 [0.54; 1.08] | Low | . | Serious | Very low | NMA estimate was used because incoherence was not available. Imprecision was rated as Serious. |
| Astragalus preparations+OX | Qizhen Capsule+OX | 0 | . | . | . |  |  |  |  |  | 0.83 [0.60; 1.15] | Low | Low | Not serious | Low | 0.83 [0.60; 1.15] | Low | . | Serious | Very low | NMA estimate was used because incoherence was not available. Imprecision was rated as Serious. |
| Astragalus preparations+OX | Shenfu Injection+OX | 0 | . | . | . |  |  |  |  |  | 1.24 [0.82; 1.86] | Low | Low | Not serious | Low | 1.24 [0.82; 1.86] | Low | . | Serious | Very low | NMA estimate was used because incoherence was not available. Imprecision was rated as Serious. |
| Astragalus preparations+OX | Shengxue Granules+OX | 0 | . | . | . |  |  |  |  |  | 1.00 [0.57; 1.75] | Low | Low | Not serious | Low | 1.00 [0.57; 1.75] | Low | . | Serious | Very low | NMA estimate was used because incoherence was not available. Imprecision was rated as Serious. |
| Astragalus preparations+OX | Shenmai Injection+OX | 0 | . | . | . |  |  |  |  |  | 0.90 [0.65; 1.26] | Low | High | Not serious | Low | 0.90 [0.65; 1.26] | Low | . | Serious | Very low | NMA estimate was used because incoherence was not available. Imprecision was rated as Serious. |
| Astragalus preparations+OX | Shenqi Fuzheng Injection+OX | 0 | . | . | . |  |  |  |  |  | 0.92 [0.75; 1.13] | Low | Low | Not serious | Low | 0.92 [0.75; 1.13] | Low | . | Serious | Very low | NMA estimate was used because incoherence was not available. Imprecision was rated as Serious. |
| Astragalus preparations+OX | Weimaining Capsules+OX | 0 | . | . | . |  |  |  |  |  | 0.94 [0.42; 2.10] | Low | High | Not serious | Low | 0.94 [0.42; 2.10] | Low | . | Serious | Very low | NMA estimate was used because incoherence was not available. Imprecision was rated as Serious. |
| Astragalus preparations+OX | Xiaoaiping Injection+OX | 0 | . | . | . |  |  |  |  |  | 0.88 [0.70; 1.11] | Low | Moderate | Not serious | Low | 0.88 [0.70; 1.11] | Low | . | Serious | Very low | NMA estimate was used because incoherence was not available. Imprecision was rated as Serious. |
| Astragalus preparations+OX | Xihuang Capsules+OX | 0 | . | . | . |  |  |  |  |  | 0.74 [0.51; 1.08] | Low | Low | Not serious | Low | 0.74 [0.51; 1.08] | Low | . | Serious | Very low | NMA estimate was used because incoherence was not available. Imprecision was rated as Serious. |
| Astragalus preparations+OX | Ya Dan Zi Oil Emulsion Injection+OX | 0 | . | . | . |  |  |  |  |  | 0.89 [0.72; 1.10] | Low | Low | Not serious | Low | 0.89 [0.72; 1.10] | Low | . | Serious | Very low | NMA estimate was used because incoherence was not available. Imprecision was rated as Serious. |
| Astragalus preparations+OX | Yangzheng Xiaoji Capsules+OX | 0 | . | . | . |  |  |  |  |  | 0.86 [0.64; 1.17] | Low | High | Not serious | Low | 0.86 [0.64; 1.17] | Low | . | Serious | Very low | NMA estimate was used because incoherence was not available. Imprecision was rated as Serious. |
| Compound Kushen Injection+OX | Compound Mylabris preparations+OX | 0 | . | . | . |  |  |  |  |  | 0.90 [0.68; 1.20] | Very low | Low | Not serious | Very low | 0.90 [0.68; 1.20] | Very low | . | Serious | Very low | NMA estimate was used because incoherence was not available. Imprecision was rated as Serious. |
| Compound Kushen Injection+OX | Ginseng Polysaccharide Injection+OX | 0 | . | . | . |  |  |  |  |  | 1.25 [0.83; 1.88] | Very low | High | Not serious | Very low | 1.25 [0.83; 1.88] | Very low | . | Serious | Very low | NMA estimate was used because incoherence was not available. Imprecision was rated as Serious. |
| Compound Kushen Injection+OX | Huachansu preparations+OX | 0 | . | . | . |  |  |  |  |  | 1.14 [0.98; 1.33] | Very low | High | Not serious | Very low | 1.14 [0.98; 1.33] | Very low | . | Serious | Very low | NMA estimate was used because incoherence was not available. Imprecision was rated as Serious. |
| Compound Kushen Injection+OX | Huai Er Granules+OX | 0 | . | . | . |  |  |  |  |  | 1.28 [0.63; 2.57] | Very low | Low | Not serious | Very low | 1.28 [0.63; 2.57] | Very low | . | Serious | Very low | NMA estimate was used because incoherence was not available. Imprecision was rated as Serious. |
| Compound Kushen Injection+OX | Jinlong Capsules+OX | 0 | . | . | . |  |  |  |  |  | 1.15 [0.80; 1.64] | Very low | High | Not serious | Very low | 1.15 [0.80; 1.64] | Very low | . | Serious | Very low | NMA estimate was used because incoherence was not available. Imprecision was rated as Serious. |
| Compound Kushen Injection+OX | Kangai Injection+OX | 0 | . | . | . |  |  |  |  |  | 0.93 [0.77; 1.13] | Very low | Moderate | Not serious | Very low | 0.93 [0.77; 1.13] | Very low | . | Serious | Very low | NMA estimate was used because incoherence was not available. Imprecision was rated as Serious. |
| Compound Kushen Injection+OX | Kanglaite Injection+OX | 0 | . | . | . |  |  |  |  |  | 1.12 [0.83; 1.51] | Very low | Moderate | Not serious | Very low | 1.12 [0.83; 1.51] | Very low | . | Serious | Very low | NMA estimate was used because incoherence was not available. Imprecision was rated as Serious. |
| Compound Kushen Injection+OX | Kanglixin Capsules+OX | 0 | . | . | . |  |  |  |  |  | 1.10 [0.85; 1.43] | Very low | High | Not serious | Very low | 1.10 [0.85; 1.43] | Very low | . | Serious | Very low | NMA estimate was used because incoherence was not available. Imprecision was rated as Serious. |
| Compound Kushen Injection+OX | Lentinan+OX | 0 | . | . | . |  |  |  |  |  | 1.46 [1.21; 1.78] | Very low | High | Not serious | Very low | 1.46 [1.21; 1.78] | Very low | . | Not serious | Very low | NMA estimate was used because incoherence was not available. Imprecision was rated as Serious. |
| Compound Kushen Injection+OX | OX | 13 | 1105 | 0.0% | 1.40 [1.26; 1.56] | Very serious | Not serious | Not serious | Serious | Very low | . |  |  |  |  | 1.40 [1.26; 1.56] | Very low | . | Not serious | Very low | NMA estimate was used because incoherence was not available. Imprecision was rated as Not serious. |
| Compound Kushen Injection+OX | Pingxiao Capsules+OX | 0 | . | . | . |  |  |  |  |  | 0.91 [0.67; 1.25] | Very low | Low | Not serious | Very low | 0.91 [0.67; 1.25] | Very low | . | Serious | Very low | NMA estimate was used because incoherence was not available. Imprecision was rated as Serious. |
| Compound Kushen Injection+OX | Qizhen Capsule+OX | 0 | . | . | . |  |  |  |  |  | 0.99 [0.74; 1.33] | Very low | Low | Not serious | Very low | 0.99 [0.74; 1.33] | Very low | . | Serious | Very low | NMA estimate was used because incoherence was not available. Imprecision was rated as Serious. |
| Compound Kushen Injection+OX | Shenfu Injection+OX | 0 | . | . | . |  |  |  |  |  | 1.48 [1.01; 2.16] | Very low | Low | Not serious | Very low | 1.48 [1.01; 2.16] | Very low | . | Not serious | Very low | NMA estimate was used because incoherence was not available. Imprecision was rated as Serious. |
| Compound Kushen Injection+OX | Shengxue Granules+OX | 0 | . | . | . |  |  |  |  |  | 1.19 [0.69; 2.05] | Very low | Low | Not serious | Very low | 1.19 [0.69; 2.05] | Very low | . | Serious | Very low | NMA estimate was used because incoherence was not available. Imprecision was rated as Serious. |
| Compound Kushen Injection+OX | Shenmai Injection+OX | 0 | . | . | . |  |  |  |  |  | 1.08 [0.80; 1.46] | Very low | High | Not serious | Very low | 1.08 [0.80; 1.46] | Very low | . | Serious | Very low | NMA estimate was used because incoherence was not available. Imprecision was rated as Serious. |
| Compound Kushen Injection+OX | Shenqi Fuzheng Injection+OX | 0 | . | . | . |  |  |  |  |  | 1.10 [0.95; 1.28] | Very low | Low | Not serious | Very low | 1.10 [0.95; 1.28] | Very low | . | Serious | Very low | NMA estimate was used because incoherence was not available. Imprecision was rated as Serious. |
| Compound Kushen Injection+OX | Weimaining Capsules+OX | 0 | . | . | . |  |  |  |  |  | 1.12 [0.51; 2.48] | Very low | High | Not serious | Very low | 1.12 [0.51; 2.48] | Very low | . | Serious | Very low | NMA estimate was used because incoherence was not available. Imprecision was rated as Serious. |
| Compound Kushen Injection+OX | Xiaoaiping Injection+OX | 0 | . | . | . |  |  |  |  |  | 1.06 [0.88; 1.27] | Very low | Moderate | Not serious | Very low | 1.06 [0.88; 1.27] | Very low | . | Serious | Very low | NMA estimate was used because incoherence was not available. Imprecision was rated as Serious. |
| Compound Kushen Injection+OX | Xihuang Capsules+OX | 0 | . | . | . |  |  |  |  |  | 0.89 [0.63; 1.26] | Very low | Low | Not serious | Very low | 0.89 [0.63; 1.26] | Very low | . | Serious | Very low | NMA estimate was used because incoherence was not available. Imprecision was rated as Serious. |
| Compound Kushen Injection+OX | Ya Dan Zi Oil Emulsion Injection+OX | 0 | . | . | . |  |  |  |  |  | 1.06 [0.90; 1.25] | Very low | Low | Not serious | Very low | 1.06 [0.90; 1.25] | Very low | . | Serious | Very low | NMA estimate was used because incoherence was not available. Imprecision was rated as Serious. |
| Compound Kushen Injection+OX | Yangzheng Xiaoji Capsules+OX | 0 | . | . | . |  |  |  |  |  | 1.03 [0.79; 1.36] | Very low | High | Not serious | Very low | 1.03 [0.79; 1.36] | Very low | . | Serious | Very low | NMA estimate was used because incoherence was not available. Imprecision was rated as Serious. |
| Compound Mylabris preparations+OX | Ginseng Polysaccharide Injection+OX | 0 | . | . | . |  |  |  |  |  | 1.39 [0.86; 2.23] | Low | High | Not serious | Low | 1.39 [0.86; 2.23] | Low | . | Serious | Very low | NMA estimate was used because incoherence was not available. Imprecision was rated as Serious. |
| Compound Mylabris preparations+OX | Huachansu preparations+OX | 0 | . | . | . |  |  |  |  |  | 1.27 [0.96; 1.68] | Low | High | Not serious | Low | 1.27 [0.96; 1.68] | Low | . | Serious | Very low | NMA estimate was used because incoherence was not available. Imprecision was rated as Serious. |
| Compound Mylabris preparations+OX | Huai Er Granules+OX | 0 | . | . | . |  |  |  |  |  | 1.42 [0.68; 2.97] | Low | Low | Not serious | Low | 1.42 [0.68; 2.97] | Low | . | Serious | Very low | NMA estimate was used because incoherence was not available. Imprecision was rated as Serious. |
| Compound Mylabris preparations+OX | Jinlong Capsules+OX | 0 | . | . | . |  |  |  |  |  | 1.27 [0.83; 1.96] | Low | High | Not serious | Low | 1.27 [0.83; 1.96] | Low | . | Serious | Very low | NMA estimate was used because incoherence was not available. Imprecision was rated as Serious. |
| Compound Mylabris preparations+OX | Kangai Injection+OX | 0 | . | . | . |  |  |  |  |  | 1.03 [0.76; 1.41] | Low | Moderate | Not serious | Low | 1.03 [0.76; 1.41] | Low | . | Serious | Very low | NMA estimate was used because incoherence was not available. Imprecision was rated as Serious. |
| Compound Mylabris preparations+OX | Kanglaite Injection+OX | 0 | . | . | . |  |  |  |  |  | 1.24 [0.85; 1.82] | Low | Moderate | Not serious | Low | 1.24 [0.85; 1.82] | Low | . | Serious | Very low | NMA estimate was used because incoherence was not available. Imprecision was rated as Serious. |
| Compound Mylabris preparations+OX | Kanglixin Capsules+OX | 0 | . | . | . |  |  |  |  |  | 1.22 [0.86; 1.74] | Low | High | Not serious | Low | 1.22 [0.86; 1.74] | Low | . | Serious | Very low | NMA estimate was used because incoherence was not available. Imprecision was rated as Serious. |
| Compound Mylabris preparations+OX | Lentinan+OX | 0 | . | . | . |  |  |  |  |  | 1.62 [1.19; 2.21] | Low | High | Not serious | Low | 1.62 [1.19; 2.21] | Low | . | Not serious | Low | NMA estimate was used because incoherence was not available. Imprecision was rated as Serious. |
| Compound Mylabris preparations+OX | OX | 4 | 285 | 0.0% | 1.56 [1.20; 2.03] | Very serious | Not serious | Not serious | Undetected | Low | . |  |  |  |  | 1.56 [1.20; 2.03] | Low | . | Not serious | Low | NMA estimate was used because incoherence was not available. Imprecision was rated as Serious. |
| Compound Mylabris preparations+OX | Pingxiao Capsules+OX | 0 | . | . | . |  |  |  |  |  | 1.01 [0.68; 1.50] | Low | Low | Not serious | Low | 1.01 [0.68; 1.50] | Low | . | Serious | Very low | NMA estimate was used because incoherence was not available. Imprecision was rated as Serious. |
| Compound Mylabris preparations+OX | Qizhen Capsule+OX | 0 | . | . | . |  |  |  |  |  | 1.10 [0.75; 1.60] | Low | Low | Not serious | Low | 1.10 [0.75; 1.60] | Low | . | Serious | Very low | NMA estimate was used because incoherence was not available. Imprecision was rated as Serious. |
| Compound Mylabris preparations+OX | Shenfu Injection+OX | 0 | . | . | . |  |  |  |  |  | 1.64 [1.04; 2.57] | Low | Low | Not serious | Low | 1.64 [1.04; 2.57] | Low | . | Not serious | Low | NMA estimate was used because incoherence was not available. Imprecision was rated as Serious. |
| Compound Mylabris preparations+OX | Shengxue Granules+OX | 0 | . | . | . |  |  |  |  |  | 1.32 [0.73; 2.39] | Low | Low | Not serious | Low | 1.32 [0.73; 2.39] | Low | . | Serious | Very low | NMA estimate was used because incoherence was not available. Imprecision was rated as Serious. |
| Compound Mylabris preparations+OX | Shenmai Injection+OX | 0 | . | . | . |  |  |  |  |  | 1.20 [0.81; 1.76] | Low | High | Not serious | Low | 1.20 [0.81; 1.76] | Low | . | Serious | Very low | NMA estimate was used because incoherence was not available. Imprecision was rated as Serious. |
| Compound Mylabris preparations+OX | Shenqi Fuzheng Injection+OX | 0 | . | . | . |  |  |  |  |  | 1.22 [0.92; 1.62] | Low | Low | Not serious | Low | 1.22 [0.92; 1.62] | Low | . | Serious | Very low | NMA estimate was used because incoherence was not available. Imprecision was rated as Serious. |
| Compound Mylabris preparations+OX | Weimaining Capsules+OX | 0 | . | . | . |  |  |  |  |  | 1.25 [0.55; 2.85] | Low | High | Not serious | Low | 1.25 [0.55; 2.85] | Low | . | Serious | Very low | NMA estimate was used because incoherence was not available. Imprecision was rated as Serious. |
| Compound Mylabris preparations+OX | Xiaoaiping Injection+OX | 0 | . | . | . |  |  |  |  |  | 1.17 [0.87; 1.58] | Low | Moderate | Not serious | Low | 1.17 [0.87; 1.58] | Low | . | Serious | Very low | NMA estimate was used because incoherence was not available. Imprecision was rated as Serious. |
| Compound Mylabris preparations+OX | Xihuang Capsules+OX | 0 | . | . | . |  |  |  |  |  | 0.99 [0.65; 1.50] | Low | Low | Not serious | Low | 0.99 [0.65; 1.50] | Low | . | Serious | Very low | NMA estimate was used because incoherence was not available. Imprecision was rated as Serious. |
| Compound Mylabris preparations+OX | Ya Dan Zi Oil Emulsion Injection+OX | 0 | . | . | . |  |  |  |  |  | 1.18 [0.88; 1.57] | Low | Low | Not serious | Low | 1.18 [0.88; 1.57] | Low | . | Serious | Very low | NMA estimate was used because incoherence was not available. Imprecision was rated as Serious. |
| Compound Mylabris preparations+OX | Yangzheng Xiaoji Capsules+OX | 0 | . | . | . |  |  |  |  |  | 1.14 [0.80; 1.64] | Low | High | Not serious | Low | 1.14 [0.80; 1.64] | Low | . | Serious | Very low | NMA estimate was used because incoherence was not available. Imprecision was rated as Serious. |
| Ginseng Polysaccharide Injection+OX | Huachansu preparations+OX | 0 | . | . | . |  |  |  |  |  | 0.91 [0.61; 1.38] | High | High | Not serious | High | 0.91 [0.61; 1.38] | High | . | Serious | Moderate | NMA estimate was used because incoherence was not available. Imprecision was rated as Serious. |
| Ginseng Polysaccharide Injection+OX | Huai Er Granules+OX | 0 | . | . | . |  |  |  |  |  | 1.02 [0.46; 2.27] | High | Low | Not serious | Low | 1.02 [0.46; 2.27] | Low | . | Serious | Very low | NMA estimate was used because incoherence was not available. Imprecision was rated as Serious. |
| Ginseng Polysaccharide Injection+OX | Jinlong Capsules+OX | 0 | . | . | . |  |  |  |  |  | 0.92 [0.55; 1.55] | High | High | Not serious | High | 0.92 [0.55; 1.55] | High | . | Serious | Moderate | NMA estimate was used because incoherence was not available. Imprecision was rated as Serious. |
| Ginseng Polysaccharide Injection+OX | Kangai Injection+OX | 0 | . | . | . |  |  |  |  |  | 0.75 [0.49; 1.14] | High | Moderate | Not serious | Moderate | 0.75 [0.49; 1.14] | Moderate | . | Serious | Low | NMA estimate was used because incoherence was not available. Imprecision was rated as Serious. |
| Ginseng Polysaccharide Injection+OX | Kanglaite Injection+OX | 0 | . | . | . |  |  |  |  |  | 0.90 [0.55; 1.45] | High | Moderate | Not serious | Moderate | 0.90 [0.55; 1.45] | Moderate | . | Serious | Low | NMA estimate was used because incoherence was not available. Imprecision was rated as Serious. |
| Ginseng Polysaccharide Injection+OX | Kanglixin Capsules+OX | 0 | . | . | . |  |  |  |  |  | 0.88 [0.56; 1.40] | High | High | Not serious | High | 0.88 [0.56; 1.40] | High | . | Serious | Moderate | NMA estimate was used because incoherence was not available. Imprecision was rated as Serious. |
| Ginseng Polysaccharide Injection+OX | Lentinan+OX | 0 | . | . | . |  |  |  |  |  | 1.17 [0.76; 1.80] | High | High | Not serious | High | 1.17 [0.76; 1.80] | High | . | Serious | Moderate | NMA estimate was used because incoherence was not available. Imprecision was rated as Serious. |
| Ginseng Polysaccharide Injection+OX | OX | 2 | 131 | 0.0% | 1.13 [0.76; 1.67] | Not serious | Not serious | Not serious | Undetected | High | . |  |  |  |  | 1.13 [0.76; 1.67] | High | . | Serious | Moderate | NMA estimate was used because incoherence was not available. Imprecision was rated as Serious. |
| Ginseng Polysaccharide Injection+OX | Pingxiao Capsules+OX | 0 | . | . | . |  |  |  |  |  | 0.73 [0.45; 1.19] | High | Low | Not serious | Low | 0.73 [0.45; 1.19] | Low | . | Serious | Very low | NMA estimate was used because incoherence was not available. Imprecision was rated as Serious. |
| Ginseng Polysaccharide Injection+OX | Qizhen Capsule+OX | 0 | . | . | . |  |  |  |  |  | 0.79 [0.49; 1.28] | High | Low | Not serious | Low | 0.79 [0.49; 1.28] | Low | . | Serious | Very low | NMA estimate was used because incoherence was not available. Imprecision was rated as Serious. |
| Ginseng Polysaccharide Injection+OX | Shenfu Injection+OX | 0 | . | . | . |  |  |  |  |  | 1.18 [0.69; 2.03] | High | Low | Not serious | Low | 1.18 [0.69; 2.03] | Low | . | Serious | Very low | NMA estimate was used because incoherence was not available. Imprecision was rated as Serious. |
| Ginseng Polysaccharide Injection+OX | Shengxue Granules+OX | 0 | . | . | . |  |  |  |  |  | 0.95 [0.49; 1.85] | High | Low | Not serious | Low | 0.95 [0.49; 1.85] | Low | . | Serious | Very low | NMA estimate was used because incoherence was not available. Imprecision was rated as Serious. |
| Ginseng Polysaccharide Injection+OX | Shenmai Injection+OX | 0 | . | . | . |  |  |  |  |  | 0.86 [0.53; 1.41] | High | High | Not serious | High | 0.86 [0.53; 1.41] | High | . | Serious | Moderate | NMA estimate was used because incoherence was not available. Imprecision was rated as Serious. |
| Ginseng Polysaccharide Injection+OX | Shenqi Fuzheng Injection+OX | 0 | . | . | . |  |  |  |  |  | 0.88 [0.59; 1.33] | High | Low | Not serious | Low | 0.88 [0.59; 1.33] | Low | . | Serious | Very low | NMA estimate was used because incoherence was not available. Imprecision was rated as Serious. |
| Ginseng Polysaccharide Injection+OX | Weimaining Capsules+OX | 0 | . | . | . |  |  |  |  |  | 0.90 [0.37; 2.17] | High | High | Not serious | High | 0.90 [0.37; 2.17] | High | . | Serious | Moderate | NMA estimate was used because incoherence was not available. Imprecision was rated as Serious. |
| Ginseng Polysaccharide Injection+OX | Xiaoaiping Injection+OX | 0 | . | . | . |  |  |  |  |  | 0.84 [0.55; 1.29] | High | Moderate | Not serious | Moderate | 0.84 [0.55; 1.29] | Moderate | . | Serious | Low | NMA estimate was used because incoherence was not available. Imprecision was rated as Serious. |
| Ginseng Polysaccharide Injection+OX | Xihuang Capsules+OX | 0 | . | . | . |  |  |  |  |  | 0.71 [0.43; 1.19] | High | Low | Not serious | Low | 0.71 [0.43; 1.19] | Low | . | Serious | Very low | NMA estimate was used because incoherence was not available. Imprecision was rated as Serious. |
| Ginseng Polysaccharide Injection+OX | Ya Dan Zi Oil Emulsion Injection+OX | 0 | . | . | . |  |  |  |  |  | 0.85 [0.56; 1.29] | High | Low | Not serious | Low | 0.85 [0.56; 1.29] | Low | . | Serious | Very low | NMA estimate was used because incoherence was not available. Imprecision was rated as Serious. |
| Ginseng Polysaccharide Injection+OX | Yangzheng Xiaoji Capsules+OX | 0 | . | . | . |  |  |  |  |  | 0.83 [0.52; 1.32] | High | High | Not serious | High | 0.83 [0.52; 1.32] | High | . | Serious | Moderate | NMA estimate was used because incoherence was not available. Imprecision was rated as Serious. |
| Huachansu preparations+OX | Huai Er Granules+OX | 0 | . | . | . |  |  |  |  |  | 1.12 [0.56; 2.25] | High | Low | Not serious | Low | 1.12 [0.56; 2.25] | Low | . | Serious | Very low | NMA estimate was used because incoherence was not available. Imprecision was rated as Serious. |
| Huachansu preparations+OX | Jinlong Capsules+OX | 0 | . | . | . |  |  |  |  |  | 1.01 [0.70; 1.44] | High | High | Not serious | High | 1.01 [0.70; 1.44] | High | . | Serious | Moderate | NMA estimate was used because incoherence was not available. Imprecision was rated as Serious. |
| Huachansu preparations+OX | Kangai Injection+OX | 0 | . | . | . |  |  |  |  |  | 0.82 [0.67; 0.99] | High | Moderate | Not serious | Moderate | 0.82 [0.67; 0.99] | Moderate | . | Serious | Low | NMA estimate was used because incoherence was not available. Imprecision was rated as Serious. |
| Huachansu preparations+OX | Kanglaite Injection+OX | 0 | . | . | . |  |  |  |  |  | 0.98 [0.73; 1.32] | High | Moderate | Not serious | Moderate | 0.98 [0.73; 1.32] | Moderate | . | Serious | Low | NMA estimate was used because incoherence was not available. Imprecision was rated as Serious. |
| Huachansu preparations+OX | Kanglixin Capsules+OX | 0 | . | . | . |  |  |  |  |  | 0.97 [0.75; 1.25] | High | High | Not serious | High | 0.97 [0.75; 1.25] | High | . | Serious | Moderate | NMA estimate was used because incoherence was not available. Imprecision was rated as Serious. |
| Huachansu preparations+OX | Lentinan+OX | 0 | . | . | . |  |  |  |  |  | 1.28 [1.06; 1.55] | High | High | Not serious | High | 1.28 [1.06; 1.55] | High | . | Not serious | Moderate | NMA estimate was used because incoherence was not available. Imprecision was rated as Serious. |
| Huachansu preparations+OX | OX | 8 | 508 | 0.0% | 1.23 [1.11; 1.37] | Not serious | Not serious | Not serious | Undetected | High | . |  |  |  |  | 1.23 [1.11; 1.37] | High | . | Not serious | Moderate | NMA estimate was used because incoherence was not available. Imprecision was rated as Serious. |
| Huachansu preparations+OX | Pingxiao Capsules+OX | 0 | . | . | . |  |  |  |  |  | 0.80 [0.58; 1.09] | High | Low | Not serious | Low | 0.80 [0.58; 1.09] | Low | . | Serious | Very low | NMA estimate was used because incoherence was not available. Imprecision was rated as Serious. |
| Huachansu preparations+OX | Qizhen Capsule+OX | 0 | . | . | . |  |  |  |  |  | 0.87 [0.64; 1.16] | High | Low | Not serious | Low | 0.87 [0.64; 1.16] | Low | . | Serious | Very low | NMA estimate was used because incoherence was not available. Imprecision was rated as Serious. |
| Huachansu preparations+OX | Shenfu Injection+OX | 0 | . | . | . |  |  |  |  |  | 1.29 [0.88; 1.89] | High | Low | Not serious | Low | 1.29 [0.88; 1.89] | Low | . | Serious | Very low | NMA estimate was used because incoherence was not available. Imprecision was rated as Serious. |
| Huachansu preparations+OX | Shengxue Granules+OX | 0 | . | . | . |  |  |  |  |  | 1.04 [0.61; 1.79] | High | Low | Not serious | Low | 1.04 [0.61; 1.79] | Low | . | Serious | Very low | NMA estimate was used because incoherence was not available. Imprecision was rated as Serious. |
| Huachansu preparations+OX | Shenmai Injection+OX | 0 | . | . | . |  |  |  |  |  | 0.94 [0.70; 1.28] | High | High | Not serious | High | 0.94 [0.70; 1.28] | High | . | Serious | Moderate | NMA estimate was used because incoherence was not available. Imprecision was rated as Serious. |
| Huachansu preparations+OX | Shenqi Fuzheng Injection+OX | 0 | . | . | . |  |  |  |  |  | 0.96 [0.83; 1.12] | High | Low | Not serious | Low | 0.96 [0.83; 1.12] | Low | . | Serious | Very low | NMA estimate was used because incoherence was not available. Imprecision was rated as Serious. |
| Huachansu preparations+OX | Weimaining Capsules+OX | 0 | . | . | . |  |  |  |  |  | 0.98 [0.45; 2.17] | High | High | Not serious | High | 0.98 [0.45; 2.17] | High | . | Serious | Moderate | NMA estimate was used because incoherence was not available. Imprecision was rated as Serious. |
| Huachansu preparations+OX | Xiaoaiping Injection+OX | 0 | . | . | . |  |  |  |  |  | 0.92 [0.77; 1.11] | High | Moderate | Not serious | Moderate | 0.92 [0.77; 1.11] | Moderate | . | Serious | Low | NMA estimate was used because incoherence was not available. Imprecision was rated as Serious. |
| Huachansu preparations+OX | Xihuang Capsules+OX | 0 | . | . | . |  |  |  |  |  | 0.78 [0.55; 1.10] | High | Low | Not serious | Low | 0.78 [0.55; 1.10] | Low | . | Serious | Very low | NMA estimate was used because incoherence was not available. Imprecision was rated as Serious. |
| Huachansu preparations+OX | Ya Dan Zi Oil Emulsion Injection+OX | 0 | . | . | . |  |  |  |  |  | 0.93 [0.79; 1.09] | High | Low | Not serious | Low | 0.93 [0.79; 1.09] | Low | . | Serious | Very low | NMA estimate was used because incoherence was not available. Imprecision was rated as Serious. |
| Huachansu preparations+OX | Yangzheng Xiaoji Capsules+OX | 0 | . | . | . |  |  |  |  |  | 0.90 [0.69; 1.19] | High | High | Not serious | High | 0.90 [0.69; 1.19] | High | . | Serious | Moderate | NMA estimate was used because incoherence was not available. Imprecision was rated as Serious. |
| Huai Er Granules+OX | Jinlong Capsules+OX | 0 | . | . | . |  |  |  |  |  | 0.90 [0.42; 1.94] | Low | High | Not serious | Low | 0.90 [0.42; 1.94] | Low | . | Serious | Very low | NMA estimate was used because incoherence was not available. Imprecision was rated as Serious. |
| Huai Er Granules+OX | Kangai Injection+OX | 0 | . | . | . |  |  |  |  |  | 0.73 [0.36; 1.48] | Low | Moderate | Not serious | Low | 0.73 [0.36; 1.48] | Low | . | Serious | Very low | NMA estimate was used because incoherence was not available. Imprecision was rated as Serious. |
| Huai Er Granules+OX | Kanglaite Injection+OX | 0 | . | . | . |  |  |  |  |  | 0.88 [0.42; 1.85] | Low | Moderate | Not serious | Low | 0.88 [0.42; 1.85] | Low | . | Serious | Very low | NMA estimate was used because incoherence was not available. Imprecision was rated as Serious. |
| Huai Er Granules+OX | Kanglixin Capsules+OX | 0 | . | . | . |  |  |  |  |  | 0.86 [0.42; 1.79] | Low | High | Not serious | Low | 0.86 [0.42; 1.79] | Low | . | Serious | Very low | NMA estimate was used because incoherence was not available. Imprecision was rated as Serious. |
| Huai Er Granules+OX | Lentinan+OX | 0 | . | . | . |  |  |  |  |  | 1.15 [0.56; 2.33] | Low | High | Not serious | Low | 1.15 [0.56; 2.33] | Low | . | Serious | Very low | NMA estimate was used because incoherence was not available. Imprecision was rated as Serious. |
| Huai Er Granules+OX | OX | 1 | 60 | . | 1.10 [0.55; 2.19] | Very serious | Not serious | Not serious | Undetected | Low | . |  |  |  |  | 1.10 [0.55; 2.19] | Low | . | Serious | Very low | NMA estimate was used because incoherence was not available. Imprecision was rated as Serious. |
| Huai Er Granules+OX | Pingxiao Capsules+OX | 0 | . | . | . |  |  |  |  |  | 0.71 [0.34; 1.51] | Low | Low | Not serious | Low | 0.71 [0.34; 1.51] | Low | . | Serious | Very low | NMA estimate was used because incoherence was not available. Imprecision was rated as Serious. |
| Huai Er Granules+OX | Qizhen Capsule+OX | 0 | . | . | . |  |  |  |  |  | 0.77 [0.37; 1.63] | Low | Low | Not serious | Low | 0.77 [0.37; 1.63] | Low | . | Serious | Very low | NMA estimate was used because incoherence was not available. Imprecision was rated as Serious. |
| Huai Er Granules+OX | Shenfu Injection+OX | 0 | . | . | . |  |  |  |  |  | 1.16 [0.53; 2.53] | Low | Low | Not serious | Low | 1.16 [0.53; 2.53] | Low | . | Serious | Very low | NMA estimate was used because incoherence was not available. Imprecision was rated as Serious. |
| Huai Er Granules+OX | Shengxue Granules+OX | 0 | . | . | . |  |  |  |  |  | 0.93 [0.39; 2.23] | Low | Low | Not serious | Low | 0.93 [0.39; 2.23] | Low | . | Serious | Very low | NMA estimate was used because incoherence was not available. Imprecision was rated as Serious. |
| Huai Er Granules+OX | Shenmai Injection+OX | 0 | . | . | . |  |  |  |  |  | 0.84 [0.40; 1.78] | Low | High | Not serious | Low | 0.84 [0.40; 1.78] | Low | . | Serious | Very low | NMA estimate was used because incoherence was not available. Imprecision was rated as Serious. |
| Huai Er Granules+OX | Shenqi Fuzheng Injection+OX | 0 | . | . | . |  |  |  |  |  | 0.86 [0.43; 1.73] | Low | Low | Not serious | Low | 0.86 [0.43; 1.73] | Low | . | Serious | Very low | NMA estimate was used because incoherence was not available. Imprecision was rated as Serious. |
| Huai Er Granules+OX | Weimaining Capsules+OX | 0 | . | . | . |  |  |  |  |  | 0.88 [0.31; 2.50] | Low | High | Not serious | Low | 0.88 [0.31; 2.50] | Low | . | Serious | Very low | NMA estimate was used because incoherence was not available. Imprecision was rated as Serious. |
| Huai Er Granules+OX | Xiaoaiping Injection+OX | 0 | . | . | . |  |  |  |  |  | 0.83 [0.41; 1.68] | Low | Moderate | Not serious | Low | 0.83 [0.41; 1.68] | Low | . | Serious | Very low | NMA estimate was used because incoherence was not available. Imprecision was rated as Serious. |
| Huai Er Granules+OX | Xihuang Capsules+OX | 0 | . | . | . |  |  |  |  |  | 0.70 [0.32; 1.50] | Low | Low | Not serious | Low | 0.70 [0.32; 1.50] | Low | . | Serious | Very low | NMA estimate was used because incoherence was not available. Imprecision was rated as Serious. |
| Huai Er Granules+OX | Ya Dan Zi Oil Emulsion Injection+OX | 0 | . | . | . |  |  |  |  |  | 0.83 [0.41; 1.68] | Low | Low | Not serious | Low | 0.83 [0.41; 1.68] | Low | . | Serious | Very low | NMA estimate was used because incoherence was not available. Imprecision was rated as Serious. |
| Huai Er Granules+OX | Yangzheng Xiaoji Capsules+OX | 0 | . | . | . |  |  |  |  |  | 0.81 [0.39; 1.68] | Low | High | Not serious | Low | 0.81 [0.39; 1.68] | Low | . | Serious | Very low | NMA estimate was used because incoherence was not available. Imprecision was rated as Serious. |
| Jinlong Capsules+OX | Kangai Injection+OX | 0 | . | . | . |  |  |  |  |  | 0.81 [0.56; 1.18] | High | Moderate | Not serious | Moderate | 0.81 [0.56; 1.18] | Moderate | . | Serious | Low | NMA estimate was used because incoherence was not available. Imprecision was rated as Serious. |
| Jinlong Capsules+OX | Kanglaite Injection+OX | 0 | . | . | . |  |  |  |  |  | 0.97 [0.63; 1.51] | High | Moderate | Not serious | Moderate | 0.97 [0.63; 1.51] | Moderate | . | Serious | Low | NMA estimate was used because incoherence was not available. Imprecision was rated as Serious. |
| Jinlong Capsules+OX | Kanglixin Capsules+OX | 0 | . | . | . |  |  |  |  |  | 0.96 [0.64; 1.45] | High | High | Not serious | High | 0.96 [0.64; 1.45] | High | . | Serious | Moderate | NMA estimate was used because incoherence was not available. Imprecision was rated as Serious. |
| Jinlong Capsules+OX | Lentinan+OX | 0 | . | . | . |  |  |  |  |  | 1.27 [0.87; 1.86] | High | High | Not serious | High | 1.27 [0.87; 1.86] | High | . | Serious | Moderate | NMA estimate was used because incoherence was not available. Imprecision was rated as Serious. |
| Jinlong Capsules+OX | OX | 3 | 194 | 0.0% | 1.22 [0.87; 1.72] | Not serious | Not serious | Not serious | Undetected | High | . |  |  |  |  | 1.22 [0.87; 1.72] | High | . | Serious | Moderate | NMA estimate was used because incoherence was not available. Imprecision was rated as Serious. |
| Jinlong Capsules+OX | Pingxiao Capsules+OX | 0 | . | . | . |  |  |  |  |  | 0.79 [0.51; 1.24] | High | Low | Not serious | Low | 0.79 [0.51; 1.24] | Low | . | Serious | Very low | NMA estimate was used because incoherence was not available. Imprecision was rated as Serious. |
| Jinlong Capsules+OX | Qizhen Capsule+OX | 0 | . | . | . |  |  |  |  |  | 0.86 [0.56; 1.33] | High | Low | Not serious | Low | 0.86 [0.56; 1.33] | Low | . | Serious | Very low | NMA estimate was used because incoherence was not available. Imprecision was rated as Serious. |
| Jinlong Capsules+OX | Shenfu Injection+OX | 0 | . | . | . |  |  |  |  |  | 1.29 [0.78; 2.12] | High | Low | Not serious | Low | 1.29 [0.78; 2.12] | Low | . | Serious | Very low | NMA estimate was used because incoherence was not available. Imprecision was rated as Serious. |
| Jinlong Capsules+OX | Shengxue Granules+OX | 0 | . | . | . |  |  |  |  |  | 1.04 [0.55; 1.95] | High | Low | Not serious | Low | 1.04 [0.55; 1.95] | Low | . | Serious | Very low | NMA estimate was used because incoherence was not available. Imprecision was rated as Serious. |
| Jinlong Capsules+OX | Shenmai Injection+OX | 0 | . | . | . |  |  |  |  |  | 0.94 [0.60; 1.46] | High | High | Not serious | High | 0.94 [0.60; 1.46] | High | . | Serious | Moderate | NMA estimate was used because incoherence was not available. Imprecision was rated as Serious. |
| Jinlong Capsules+OX | Shenqi Fuzheng Injection+OX | 0 | . | . | . |  |  |  |  |  | 0.96 [0.67; 1.37] | High | Low | Not serious | Low | 0.96 [0.67; 1.37] | Low | . | Serious | Very low | NMA estimate was used because incoherence was not available. Imprecision was rated as Serious. |
| Jinlong Capsules+OX | Weimaining Capsules+OX | 0 | . | . | . |  |  |  |  |  | 0.98 [0.42; 2.30] | High | High | Not serious | High | 0.98 [0.42; 2.30] | High | . | Serious | Moderate | NMA estimate was used because incoherence was not available. Imprecision was rated as Serious. |
| Jinlong Capsules+OX | Xiaoaiping Injection+OX | 0 | . | . | . |  |  |  |  |  | 0.92 [0.63; 1.33] | High | Moderate | Not serious | Moderate | 0.92 [0.63; 1.33] | Moderate | . | Serious | Low | NMA estimate was used because incoherence was not available. Imprecision was rated as Serious. |
| Jinlong Capsules+OX | Xihuang Capsules+OX | 0 | . | . | . |  |  |  |  |  | 0.77 [0.48; 1.24] | High | Low | Not serious | Low | 0.77 [0.48; 1.24] | Low | . | Serious | Very low | NMA estimate was used because incoherence was not available. Imprecision was rated as Serious. |
| Jinlong Capsules+OX | Ya Dan Zi Oil Emulsion Injection+OX | 0 | . | . | . |  |  |  |  |  | 0.92 [0.64; 1.33] | High | Low | Not serious | Low | 0.92 [0.64; 1.33] | Low | . | Serious | Very low | NMA estimate was used because incoherence was not available. Imprecision was rated as Serious. |
| Jinlong Capsules+OX | Yangzheng Xiaoji Capsules+OX | 0 | . | . | . |  |  |  |  |  | 0.90 [0.59; 1.37] | High | High | Not serious | High | 0.90 [0.59; 1.37] | High | . | Serious | Moderate | NMA estimate was used because incoherence was not available. Imprecision was rated as Serious. |
| Kangai Injection+OX | Kanglaite Injection+OX | 0 | . | . | . |  |  |  |  |  | 1.20 [0.87; 1.66] | Moderate | Moderate | Not serious | Moderate | 1.20 [0.87; 1.66] | Moderate | . | Serious | Low | NMA estimate was used because incoherence was not available. Imprecision was rated as Serious. |
| Kangai Injection+OX | Kanglixin Capsules+OX | 0 | . | . | . |  |  |  |  |  | 1.19 [0.89; 1.58] | Moderate | High | Not serious | Moderate | 1.19 [0.89; 1.58] | Moderate | . | Serious | Low | NMA estimate was used because incoherence was not available. Imprecision was rated as Serious. |
| Kangai Injection+OX | Lentinan+OX | 0 | . | . | . |  |  |  |  |  | 1.57 [1.25; 1.97] | Moderate | High | Not serious | Moderate | 1.57 [1.25; 1.97] | Moderate | . | Not serious | Moderate | NMA estimate was used because incoherence was not available. Imprecision was rated as Serious. |
| Kangai Injection+OX | OX | 7 | 761 | 0.0% | 1.51 [1.28; 1.77] | Serious | Not serious | Not serious | Undetected | Moderate | . |  |  |  |  | 1.51 [1.28; 1.77] | Moderate | . | Not serious | Moderate | NMA estimate was used because incoherence was not available. Imprecision was rated as Serious. |
| Kangai Injection+OX | Pingxiao Capsules+OX | 0 | . | . | . |  |  |  |  |  | 0.98 [0.70; 1.37] | Moderate | Low | Not serious | Low | 0.98 [0.70; 1.37] | Low | . | Serious | Very low | NMA estimate was used because incoherence was not available. Imprecision was rated as Serious. |
| Kangai Injection+OX | Qizhen Capsule+OX | 0 | . | . | . |  |  |  |  |  | 1.06 [0.77; 1.46] | Moderate | Low | Not serious | Low | 1.06 [0.77; 1.46] | Low | . | Serious | Very low | NMA estimate was used because incoherence was not available. Imprecision was rated as Serious. |
| Kangai Injection+OX | Shenfu Injection+OX | 0 | . | . | . |  |  |  |  |  | 1.59 [1.06; 2.37] | Moderate | Low | Not serious | Low | 1.59 [1.06; 2.37] | Low | . | Not serious | Low | NMA estimate was used because incoherence was not available. Imprecision was rated as Serious. |
| Kangai Injection+OX | Shengxue Granules+OX | 0 | . | . | . |  |  |  |  |  | 1.28 [0.73; 2.23] | Moderate | Low | Not serious | Low | 1.28 [0.73; 2.23] | Low | . | Serious | Very low | NMA estimate was used because incoherence was not available. Imprecision was rated as Serious. |
| Kangai Injection+OX | Shenmai Injection+OX | 0 | . | . | . |  |  |  |  |  | 1.16 [0.83; 1.61] | Moderate | High | Not serious | Moderate | 1.16 [0.83; 1.61] | Moderate | . | Serious | Low | NMA estimate was used because incoherence was not available. Imprecision was rated as Serious. |
| Kangai Injection+OX | Shenqi Fuzheng Injection+OX | 0 | . | . | . |  |  |  |  |  | 1.18 [0.98; 1.43] | Moderate | Low | Not serious | Low | 1.18 [0.98; 1.43] | Low | . | Serious | Very low | NMA estimate was used because incoherence was not available. Imprecision was rated as Serious. |
| Kangai Injection+OX | Weimaining Capsules+OX | 0 | . | . | . |  |  |  |  |  | 1.21 [0.54; 2.69] | Moderate | High | Not serious | Moderate | 1.21 [0.54; 2.69] | Moderate | . | Serious | Low | NMA estimate was used because incoherence was not available. Imprecision was rated as Serious. |
| Kangai Injection+OX | Xiaoaiping Injection+OX | 0 | . | . | . |  |  |  |  |  | 1.13 [0.91; 1.41] | Moderate | Moderate | Not serious | Moderate | 1.13 [0.91; 1.41] | Moderate | . | Serious | Low | NMA estimate was used because incoherence was not available. Imprecision was rated as Serious. |
| Kangai Injection+OX | Xihuang Capsules+OX | 0 | . | . | . |  |  |  |  |  | 0.95 [0.66; 1.38] | Moderate | Low | Not serious | Low | 0.95 [0.66; 1.38] | Low | . | Serious | Very low | NMA estimate was used because incoherence was not available. Imprecision was rated as Serious. |
| Kangai Injection+OX | Ya Dan Zi Oil Emulsion Injection+OX | 0 | . | . | . |  |  |  |  |  | 1.14 [0.93; 1.39] | Moderate | Low | Not serious | Low | 1.14 [0.93; 1.39] | Low | . | Serious | Very low | NMA estimate was used because incoherence was not available. Imprecision was rated as Serious. |
| Kangai Injection+OX | Yangzheng Xiaoji Capsules+OX | 0 | . | . | . |  |  |  |  |  | 1.11 [0.82; 1.49] | Moderate | High | Not serious | Moderate | 1.11 [0.82; 1.49] | Moderate | . | Serious | Low | NMA estimate was used because incoherence was not available. Imprecision was rated as Serious. |
| Kanglaite Injection+OX | Kanglixin Capsules+OX | 0 | . | . | . |  |  |  |  |  | 0.99 [0.68; 1.42] | Moderate | High | Not serious | Moderate | 0.99 [0.68; 1.42] | Moderate | . | Serious | Low | NMA estimate was used because incoherence was not available. Imprecision was rated as Serious. |
| Kanglaite Injection+OX | Lentinan+OX | 0 | . | . | . |  |  |  |  |  | 1.31 [0.95; 1.81] | Moderate | High | Not serious | Moderate | 1.31 [0.95; 1.81] | Moderate | . | Serious | Low | NMA estimate was used because incoherence was not available. Imprecision was rated as Serious. |
| Kanglaite Injection+OX | OX | 3 | 201 | 0.0% | 1.26 [0.95; 1.66] | Serious | Not serious | Not serious | Undetected | Moderate | . |  |  |  |  | 1.26 [0.95; 1.66] | Moderate | . | Serious | Low | NMA estimate was used because incoherence was not available. Imprecision was rated as Serious. |
| Kanglaite Injection+OX | Pingxiao Capsules+OX | 0 | . | . | . |  |  |  |  |  | 0.81 [0.54; 1.22] | Moderate | Low | Not serious | Low | 0.81 [0.54; 1.22] | Low | . | Serious | Very low | NMA estimate was used because incoherence was not available. Imprecision was rated as Serious. |
| Kanglaite Injection+OX | Qizhen Capsule+OX | 0 | . | . | . |  |  |  |  |  | 0.88 [0.60; 1.31] | Moderate | Low | Not serious | Low | 0.88 [0.60; 1.31] | Low | . | Serious | Very low | NMA estimate was used because incoherence was not available. Imprecision was rated as Serious. |
| Kanglaite Injection+OX | Shenfu Injection+OX | 0 | . | . | . |  |  |  |  |  | 1.32 [0.83; 2.09] | Moderate | Low | Not serious | Low | 1.32 [0.83; 2.09] | Low | . | Serious | Very low | NMA estimate was used because incoherence was not available. Imprecision was rated as Serious. |
| Kanglaite Injection+OX | Shengxue Granules+OX | 0 | . | . | . |  |  |  |  |  | 1.06 [0.58; 1.94] | Moderate | Low | Not serious | Low | 1.06 [0.58; 1.94] | Low | . | Serious | Very low | NMA estimate was used because incoherence was not available. Imprecision was rated as Serious. |
| Kanglaite Injection+OX | Shenmai Injection+OX | 0 | . | . | . |  |  |  |  |  | 0.96 [0.65; 1.44] | Moderate | High | Not serious | Moderate | 0.96 [0.65; 1.44] | Moderate | . | Serious | Low | NMA estimate was used because incoherence was not available. Imprecision was rated as Serious. |
| Kanglaite Injection+OX | Shenqi Fuzheng Injection+OX | 0 | . | . | . |  |  |  |  |  | 0.98 [0.73; 1.33] | Moderate | Low | Not serious | Low | 0.98 [0.73; 1.33] | Low | . | Serious | Very low | NMA estimate was used because incoherence was not available. Imprecision was rated as Serious. |
| Kanglaite Injection+OX | Weimaining Capsules+OX | 0 | . | . | . |  |  |  |  |  | 1.00 [0.44; 2.31] | Moderate | High | Not serious | Moderate | 1.00 [0.44; 2.31] | Moderate | . | Serious | Low | NMA estimate was used because incoherence was not available. Imprecision was rated as Serious. |
| Kanglaite Injection+OX | Xiaoaiping Injection+OX | 0 | . | . | . |  |  |  |  |  | 0.94 [0.69; 1.30] | Moderate | Moderate | Not serious | Moderate | 0.94 [0.69; 1.30] | Moderate | . | Serious | Low | NMA estimate was used because incoherence was not available. Imprecision was rated as Serious. |
| Kanglaite Injection+OX | Xihuang Capsules+OX | 0 | . | . | . |  |  |  |  |  | 0.79 [0.52; 1.22] | Moderate | Low | Not serious | Low | 0.79 [0.52; 1.22] | Low | . | Serious | Very low | NMA estimate was used because incoherence was not available. Imprecision was rated as Serious. |
| Kanglaite Injection+OX | Ya Dan Zi Oil Emulsion Injection+OX | 0 | . | . | . |  |  |  |  |  | 0.95 [0.70; 1.29] | Moderate | Low | Not serious | Low | 0.95 [0.70; 1.29] | Low | . | Serious | Very low | NMA estimate was used because incoherence was not available. Imprecision was rated as Serious. |
| Kanglaite Injection+OX | Yangzheng Xiaoji Capsules+OX | 0 | . | . | . |  |  |  |  |  | 0.92 [0.63; 1.34] | Moderate | High | Not serious | Moderate | 0.92 [0.63; 1.34] | Moderate | . | Serious | Low | NMA estimate was used because incoherence was not available. Imprecision was rated as Serious. |
| Kanglixin Capsules+OX | Lentinan+OX | 0 | . | . | . |  |  |  |  |  | 1.33 [1.00; 1.76] | High | High | Not serious | High | 1.33 [1.00; 1.76] | High | . | Serious | Moderate | NMA estimate was used because incoherence was not available. Imprecision was rated as Serious. |
| Kanglixin Capsules+OX | OX | 1 | 60 | . | 1.27 [1.01; 1.61] | Not serious | Not serious | Not serious | Undetected | High | . |  |  |  |  | 1.27 [1.01; 1.61] | High | . | Serious | Moderate | NMA estimate was used because incoherence was not available. Imprecision was rated as Serious. |
| Kanglixin Capsules+OX | Pingxiao Capsules+OX | 0 | . | . | . |  |  |  |  |  | 0.83 [0.57; 1.20] | High | Low | Not serious | Low | 0.83 [0.57; 1.20] | Low | . | Serious | Very low | NMA estimate was used because incoherence was not available. Imprecision was rated as Serious. |
| Kanglixin Capsules+OX | Qizhen Capsule+OX | 0 | . | . | . |  |  |  |  |  | 0.90 [0.62; 1.29] | High | Low | Not serious | Low | 0.90 [0.62; 1.29] | Low | . | Serious | Very low | NMA estimate was used because incoherence was not available. Imprecision was rated as Serious. |
| Kanglixin Capsules+OX | Shenfu Injection+OX | 0 | . | . | . |  |  |  |  |  | 1.34 [0.87; 2.07] | High | Low | Not serious | Low | 1.34 [0.87; 2.07] | Low | . | Serious | Very low | NMA estimate was used because incoherence was not available. Imprecision was rated as Serious. |
| Kanglixin Capsules+OX | Shengxue Granules+OX | 0 | . | . | . |  |  |  |  |  | 1.08 [0.60; 1.93] | High | Low | Not serious | Low | 1.08 [0.60; 1.93] | Low | . | Serious | Very low | NMA estimate was used because incoherence was not available. Imprecision was rated as Serious. |
| Kanglixin Capsules+OX | Shenmai Injection+OX | 0 | . | . | . |  |  |  |  |  | 0.98 [0.68; 1.41] | High | High | Not serious | High | 0.98 [0.68; 1.41] | High | . | Serious | Moderate | NMA estimate was used because incoherence was not available. Imprecision was rated as Serious. |
| Kanglixin Capsules+OX | Shenqi Fuzheng Injection+OX | 0 | . | . | . |  |  |  |  |  | 1.00 [0.77; 1.29] | High | Low | Not serious | Low | 1.00 [0.77; 1.29] | Low | . | Serious | Very low | NMA estimate was used because incoherence was not available. Imprecision was rated as Serious. |
| Kanglixin Capsules+OX | Weimaining Capsules+OX | 0 | . | . | . |  |  |  |  |  | 1.02 [0.45; 2.31] | High | High | Not serious | High | 1.02 [0.45; 2.31] | High | . | Serious | Moderate | NMA estimate was used because incoherence was not available. Imprecision was rated as Serious. |
| Kanglixin Capsules+OX | Xiaoaiping Injection+OX | 0 | . | . | . |  |  |  |  |  | 0.96 [0.72; 1.26] | High | Moderate | Not serious | Moderate | 0.96 [0.72; 1.26] | Moderate | . | Serious | Low | NMA estimate was used because incoherence was not available. Imprecision was rated as Serious. |
| Kanglixin Capsules+OX | Xihuang Capsules+OX | 0 | . | . | . |  |  |  |  |  | 0.81 [0.54; 1.21] | High | Low | Not serious | Low | 0.81 [0.54; 1.21] | Low | . | Serious | Very low | NMA estimate was used because incoherence was not available. Imprecision was rated as Serious. |
| Kanglixin Capsules+OX | Ya Dan Zi Oil Emulsion Injection+OX | 0 | . | . | . |  |  |  |  |  | 0.96 [0.74; 1.25] | High | Low | Not serious | Low | 0.96 [0.74; 1.25] | Low | . | Serious | Very low | NMA estimate was used because incoherence was not available. Imprecision was rated as Serious. |
| Kanglixin Capsules+OX | Yangzheng Xiaoji Capsules+OX | 0 | . | . | . |  |  |  |  |  | 0.93 [0.66; 1.32] | High | High | Not serious | High | 0.93 [0.66; 1.32] | High | . | Serious | Moderate | NMA estimate was used because incoherence was not available. Imprecision was rated as Serious. |
| Lentinan+OX | OX | 4 | 357 | 79.1% | 0.96 [0.82; 1.13] | Not serious | Not serious | Not serious | Undetected | High | . |  |  |  |  | 0.96 [0.82; 1.13] | High | . | Serious | Moderate | NMA estimate was used because incoherence was not available. Imprecision was rated as Serious. |
| Lentinan+OX | Pingxiao Capsules+OX | 0 | . | . | . |  |  |  |  |  | 0.62 [0.44; 0.87] | High | Low | Not serious | Low | 0.62 [0.44; 0.87] | Low | . | Serious | Very low | NMA estimate was used because incoherence was not available. Imprecision was rated as Serious. |
| Lentinan+OX | Qizhen Capsule+OX | 0 | . | . | . |  |  |  |  |  | 0.68 [0.49; 0.93] | High | Low | Not serious | Low | 0.68 [0.49; 0.93] | Low | . | Serious | Very low | NMA estimate was used because incoherence was not available. Imprecision was rated as Serious. |
| Lentinan+OX | Shenfu Injection+OX | 0 | . | . | . |  |  |  |  |  | 1.01 [0.68; 1.50] | High | Low | Not serious | Low | 1.01 [0.68; 1.50] | Low | . | Serious | Very low | NMA estimate was used because incoherence was not available. Imprecision was rated as Serious. |
| Lentinan+OX | Shengxue Granules+OX | 0 | . | . | . |  |  |  |  |  | 0.81 [0.47; 1.42] | High | Low | Not serious | Low | 0.81 [0.47; 1.42] | Low | . | Serious | Very low | NMA estimate was used because incoherence was not available. Imprecision was rated as Serious. |
| Lentinan+OX | Shenmai Injection+OX | 0 | . | . | . |  |  |  |  |  | 0.74 [0.53; 1.02] | High | High | Not serious | High | 0.74 [0.53; 1.02] | High | . | Serious | Moderate | NMA estimate was used because incoherence was not available. Imprecision was rated as Serious. |
| Lentinan+OX | Shenqi Fuzheng Injection+OX | 0 | . | . | . |  |  |  |  |  | 0.75 [0.62; 0.91] | High | Low | Not serious | Low | 0.75 [0.62; 0.91] | Low | . | Serious | Very low | NMA estimate was used because incoherence was not available. Imprecision was rated as Serious. |
| Lentinan+OX | Weimaining Capsules+OX | 0 | . | . | . |  |  |  |  |  | 0.77 [0.34; 1.71] | High | High | Not serious | High | 0.77 [0.34; 1.71] | High | . | Serious | Moderate | NMA estimate was used because incoherence was not available. Imprecision was rated as Serious. |
| Lentinan+OX | Xiaoaiping Injection+OX | 0 | . | . | . |  |  |  |  |  | 0.72 [0.58; 0.90] | High | Moderate | Not serious | Moderate | 0.72 [0.58; 0.90] | Moderate | . | Serious | Low | NMA estimate was used because incoherence was not available. Imprecision was rated as Serious. |
| Lentinan+OX | Xihuang Capsules+OX | 0 | . | . | . |  |  |  |  |  | 0.61 [0.42; 0.88] | High | Low | Not serious | Low | 0.61 [0.42; 0.88] | Low | . | Serious | Very low | NMA estimate was used because incoherence was not available. Imprecision was rated as Serious. |
| Lentinan+OX | Ya Dan Zi Oil Emulsion Injection+OX | 0 | . | . | . |  |  |  |  |  | 0.72 [0.59; 0.89] | High | Low | Not serious | Low | 0.72 [0.59; 0.89] | Low | . | Serious | Very low | NMA estimate was used because incoherence was not available. Imprecision was rated as Serious. |
| Lentinan+OX | Yangzheng Xiaoji Capsules+OX | 0 | . | . | . |  |  |  |  |  | 0.70 [0.52; 0.95] | High | High | Not serious | High | 0.70 [0.52; 0.95] | High | . | Serious | Moderate | NMA estimate was used because incoherence was not available. Imprecision was rated as Serious. |
| Pingxiao Capsules+OX | OX | 2 | 164 | 0.0% | 1.54 [1.15; 2.07] | Very serious | Not serious | Not serious | Undetected | Low | . |  |  |  |  | 1.54 [1.15; 2.07] | Low | . | Not serious | Low | NMA estimate was used because incoherence was not available. Imprecision was rated as Serious. |
| Qizhen Capsule+OX | OX | 1 | 160 | . | 1.42 [1.08; 1.87] | Very serious | Not serious | Not serious | Undetected | Low | . |  |  |  |  | 1.42 [1.08; 1.87] | Low | . | Not serious | Low | NMA estimate was used because incoherence was not available. Imprecision was rated as Serious. |
| Shenfu Injection+OX | OX | 1 | 135 | . | 0.95 [0.66; 1.37] | Very serious | Not serious | Not serious | Undetected | Low | . |  |  |  |  | 0.95 [0.66; 1.37] | Low | . | Serious | Very low | NMA estimate was used because incoherence was not available. Imprecision was rated as Serious. |
| Shengxue Granules+OX | OX | 1 | 71 | . | 1.18 [0.69; 2.01] | Very serious | Not serious | Not serious | Undetected | Low | . |  |  |  |  | 1.18 [0.69; 2.01] | Low | . | Serious | Very low | NMA estimate was used because incoherence was not available. Imprecision was rated as Serious. |
| Shenmai Injection+OX | OX | 2 | 173 | 31.7% | 1.30 [0.98; 1.73] | Not serious | Not serious | Not serious | Undetected | High | . |  |  |  |  | 1.30 [0.98; 1.73] | High | . | Serious | Moderate | NMA estimate was used because incoherence was not available. Imprecision was rated as Serious. |
| Shenqi Fuzheng Injection+OX | OX | 19 | 1298 | 0.0% | 1.28 [1.15; 1.41] | Serious | Not serious | Not serious | Serious | Low | . |  |  |  |  | 1.28 [1.15; 1.41] | Low | . | Not serious | Low | NMA estimate was used because incoherence was not available. Imprecision was rated as Not serious. |
| Weimaining Capsules+OX | OX | 1 | 62 | . | 1.25 [0.57; 2.74] | Not serious | Not serious | Not serious | Undetected | High | . |  |  |  |  | 1.25 [0.57; 2.74] | High | . | Serious | Moderate | NMA estimate was used because incoherence was not available. Imprecision was rated as Serious. |
| Xiaoaiping Injection+OX | OX | 6 | 731 | 0.0% | 1.33 [1.15; 1.55] | Serious | Not serious | Not serious | Undetected | Moderate | . |  |  |  |  | 1.33 [1.15; 1.55] | Moderate | . | Not serious | Moderate | NMA estimate was used because incoherence was not available. Imprecision was rated as Serious. |
| Xihuang Capsules+OX | OX | 2 | 164 | 0.0% | 1.58 [1.14; 2.20] | Very serious | Not serious | Not serious | Undetected | Low | . |  |  |  |  | 1.58 [1.14; 2.20] | Low | . | Not serious | Low | NMA estimate was used because incoherence was not available. Imprecision was rated as Serious. |
| Ya Dan Zi Oil Emulsion Injection+OX | OX | 8 | 641 | 0.0% | 1.32 [1.17; 1.50] | Very serious | Not serious | Not serious | Undetected | Low | . |  |  |  |  | 1.32 [1.17; 1.50] | Low | . | Not serious | Low | NMA estimate was used because incoherence was not available. Imprecision was rated as Serious. |
| Yangzheng Xiaoji Capsules+OX | OX | 4 | 297 | 5.8% | 1.36 [1.06; 1.75] | Not serious | Not serious | Not serious | Undetected | High | . |  |  |  |  | 1.36 [1.06; 1.75] | High | . | Not serious | High | NMA estimate was used because incoherence was not available. Imprecision was rated as Serious. |
| Pingxiao Capsules+OX | Qizhen Capsule+OX | 0 | . | . | . |  |  |  |  |  | 1.09 [0.72; 1.63] | Low | Low | Not serious | Low | 1.09 [0.72; 1.63] | Low | . | Serious | Very low | NMA estimate was used because incoherence was not available. Imprecision was rated as Serious. |
| Pingxiao Capsules+OX | Shenfu Injection+OX | 0 | . | . | . |  |  |  |  |  | 1.62 [1.01; 2.60] | Low | Low | Not serious | Low | 1.62 [1.01; 2.60] | Low | . | Not serious | Low | NMA estimate was used because incoherence was not available. Imprecision was rated as Serious. |
| Pingxiao Capsules+OX | Shengxue Granules+OX | 0 | . | . | . |  |  |  |  |  | 1.31 [0.71; 2.40] | Low | Low | Not serious | Low | 1.31 [0.71; 2.40] | Low | . | Serious | Very low | NMA estimate was used because incoherence was not available. Imprecision was rated as Serious. |
| Pingxiao Capsules+OX | Shenmai Injection+OX | 0 | . | . | . |  |  |  |  |  | 1.18 [0.79; 1.79] | Low | High | Not serious | Low | 1.18 [0.79; 1.79] | Low | . | Serious | Very low | NMA estimate was used because incoherence was not available. Imprecision was rated as Serious. |
| Pingxiao Capsules+OX | Shenqi Fuzheng Injection+OX | 0 | . | . | . |  |  |  |  |  | 1.21 [0.88; 1.65] | Low | Low | Not serious | Low | 1.21 [0.88; 1.65] | Low | . | Serious | Very low | NMA estimate was used because incoherence was not available. Imprecision was rated as Serious. |
| Pingxiao Capsules+OX | Weimaining Capsules+OX | 0 | . | . | . |  |  |  |  |  | 1.23 [0.53; 2.86] | Low | High | Not serious | Low | 1.23 [0.53; 2.86] | Low | . | Serious | Very low | NMA estimate was used because incoherence was not available. Imprecision was rated as Serious. |
| Pingxiao Capsules+OX | Xiaoaiping Injection+OX | 0 | . | . | . |  |  |  |  |  | 1.16 [0.83; 1.61] | Low | Moderate | Not serious | Low | 1.16 [0.83; 1.61] | Low | . | Serious | Very low | NMA estimate was used because incoherence was not available. Imprecision was rated as Serious. |
| Pingxiao Capsules+OX | Xihuang Capsules+OX | 0 | . | . | . |  |  |  |  |  | 0.98 [0.63; 1.52] | Low | Low | Not serious | Low | 0.98 [0.63; 1.52] | Low | . | Serious | Very low | NMA estimate was used because incoherence was not available. Imprecision was rated as Serious. |
| Pingxiao Capsules+OX | Ya Dan Zi Oil Emulsion Injection+OX | 0 | . | . | . |  |  |  |  |  | 1.16 [0.85; 1.60] | Low | Low | Not serious | Low | 1.16 [0.85; 1.60] | Low | . | Serious | Very low | NMA estimate was used because incoherence was not available. Imprecision was rated as Serious. |
| Pingxiao Capsules+OX | Yangzheng Xiaoji Capsules+OX | 0 | . | . | . |  |  |  |  |  | 1.13 [0.77; 1.67] | Low | High | Not serious | Low | 1.13 [0.77; 1.67] | Low | . | Serious | Very low | NMA estimate was used because incoherence was not available. Imprecision was rated as Serious. |
| Qizhen Capsule+OX | Shenfu Injection+OX | 0 | . | . | . |  |  |  |  |  | 1.49 [0.94; 2.36] | Low | Low | Not serious | Low | 1.49 [0.94; 2.36] | Low | . | Serious | Very low | NMA estimate was used because incoherence was not available. Imprecision was rated as Serious. |
| Qizhen Capsule+OX | Shengxue Granules+OX | 0 | . | . | . |  |  |  |  |  | 1.20 [0.66; 2.19] | Low | Low | Not serious | Low | 1.20 [0.66; 2.19] | Low | . | Serious | Very low | NMA estimate was used because incoherence was not available. Imprecision was rated as Serious. |
| Qizhen Capsule+OX | Shenmai Injection+OX | 0 | . | . | . |  |  |  |  |  | 1.09 [0.73; 1.62] | Low | High | Not serious | Low | 1.09 [0.73; 1.62] | Low | . | Serious | Very low | NMA estimate was used because incoherence was not available. Imprecision was rated as Serious. |
| Qizhen Capsule+OX | Shenqi Fuzheng Injection+OX | 0 | . | . | . |  |  |  |  |  | 1.11 [0.83; 1.50] | Low | Low | Not serious | Low | 1.11 [0.83; 1.50] | Low | . | Serious | Very low | NMA estimate was used because incoherence was not available. Imprecision was rated as Serious. |
| Qizhen Capsule+OX | Weimaining Capsules+OX | 0 | . | . | . |  |  |  |  |  | 1.14 [0.49; 2.61] | Low | High | Not serious | Low | 1.14 [0.49; 2.61] | Low | . | Serious | Very low | NMA estimate was used because incoherence was not available. Imprecision was rated as Serious. |
| Qizhen Capsule+OX | Xiaoaiping Injection+OX | 0 | . | . | . |  |  |  |  |  | 1.07 [0.78; 1.46] | Low | Moderate | Not serious | Low | 1.07 [0.78; 1.46] | Low | . | Serious | Very low | NMA estimate was used because incoherence was not available. Imprecision was rated as Serious. |
| Qizhen Capsule+OX | Xihuang Capsules+OX | 0 | . | . | . |  |  |  |  |  | 0.90 [0.58; 1.38] | Low | Low | Not serious | Low | 0.90 [0.58; 1.38] | Low | . | Serious | Very low | NMA estimate was used because incoherence was not available. Imprecision was rated as Serious. |
| Qizhen Capsule+OX | Ya Dan Zi Oil Emulsion Injection+OX | 0 | . | . | . |  |  |  |  |  | 1.07 [0.79; 1.45] | Low | Low | Not serious | Low | 1.07 [0.79; 1.45] | Low | . | Serious | Very low | NMA estimate was used because incoherence was not available. Imprecision was rated as Serious. |
| Qizhen Capsule+OX | Yangzheng Xiaoji Capsules+OX | 0 | . | . | . |  |  |  |  |  | 1.04 [0.72; 1.52] | Low | High | Not serious | Low | 1.04 [0.72; 1.52] | Low | . | Serious | Very low | NMA estimate was used because incoherence was not available. Imprecision was rated as Serious. |
| Shenfu Injection+OX | Shengxue Granules+OX | 0 | . | . | . |  |  |  |  |  | 0.81 [0.42; 1.54] | Low | Low | Not serious | Low | 0.81 [0.42; 1.54] | Low | . | Serious | Very low | NMA estimate was used because incoherence was not available. Imprecision was rated as Serious. |
| Shenfu Injection+OX | Shenmai Injection+OX | 0 | . | . | . |  |  |  |  |  | 0.73 [0.46; 1.16] | Low | High | Not serious | Low | 0.73 [0.46; 1.16] | Low | . | Serious | Very low | NMA estimate was used because incoherence was not available. Imprecision was rated as Serious. |
| Shenfu Injection+OX | Shenqi Fuzheng Injection+OX | 0 | . | . | . |  |  |  |  |  | 0.75 [0.51; 1.09] | Low | Low | Not serious | Low | 0.75 [0.51; 1.09] | Low | . | Serious | Very low | NMA estimate was used because incoherence was not available. Imprecision was rated as Serious. |
| Shenfu Injection+OX | Weimaining Capsules+OX | 0 | . | . | . |  |  |  |  |  | 0.76 [0.32; 1.81] | Low | High | Not serious | Low | 0.76 [0.32; 1.81] | Low | . | Serious | Very low | NMA estimate was used because incoherence was not available. Imprecision was rated as Serious. |
| Shenfu Injection+OX | Xiaoaiping Injection+OX | 0 | . | . | . |  |  |  |  |  | 0.71 [0.48; 1.06] | Low | Moderate | Not serious | Low | 0.71 [0.48; 1.06] | Low | . | Serious | Very low | NMA estimate was used because incoherence was not available. Imprecision was rated as Serious. |
| Shenfu Injection+OX | Xihuang Capsules+OX | 0 | . | . | . |  |  |  |  |  | 0.60 [0.37; 0.99] | Low | Low | Not serious | Low | 0.60 [0.37; 0.99] | Low | . | Serious | Very low | NMA estimate was used because incoherence was not available. Imprecision was rated as Serious. |
| Shenfu Injection+OX | Ya Dan Zi Oil Emulsion Injection+OX | 0 | . | . | . |  |  |  |  |  | 0.72 [0.49; 1.06] | Low | Low | Not serious | Low | 0.72 [0.49; 1.06] | Low | . | Serious | Very low | NMA estimate was used because incoherence was not available. Imprecision was rated as Serious. |
| Shenfu Injection+OX | Yangzheng Xiaoji Capsules+OX | 0 | . | . | . |  |  |  |  |  | 0.70 [0.45; 1.09] | Low | High | Not serious | Low | 0.70 [0.45; 1.09] | Low | . | Serious | Very low | NMA estimate was used because incoherence was not available. Imprecision was rated as Serious. |
| Shengxue Granules+OX | Shenmai Injection+OX | 0 | . | . | . |  |  |  |  |  | 0.91 [0.50; 1.66] | Low | High | Not serious | Low | 0.91 [0.50; 1.66] | Low | . | Serious | Very low | NMA estimate was used because incoherence was not available. Imprecision was rated as Serious. |
| Shengxue Granules+OX | Shenqi Fuzheng Injection+OX | 0 | . | . | . |  |  |  |  |  | 0.93 [0.54; 1.59] | Low | Low | Not serious | Low | 0.93 [0.54; 1.59] | Low | . | Serious | Very low | NMA estimate was used because incoherence was not available. Imprecision was rated as Serious. |
| Shengxue Granules+OX | Weimaining Capsules+OX | 0 | . | . | . |  |  |  |  |  | 0.94 [0.37; 2.44] | Low | High | Not serious | Low | 0.94 [0.37; 2.44] | Low | . | Serious | Very low | NMA estimate was used because incoherence was not available. Imprecision was rated as Serious. |
| Shengxue Granules+OX | Xiaoaiping Injection+OX | 0 | . | . | . |  |  |  |  |  | 0.89 [0.51; 1.54] | Low | Moderate | Not serious | Low | 0.89 [0.51; 1.54] | Low | . | Serious | Very low | NMA estimate was used because incoherence was not available. Imprecision was rated as Serious. |
| Shengxue Granules+OX | Xihuang Capsules+OX | 0 | . | . | . |  |  |  |  |  | 0.75 [0.40; 1.40] | Low | Low | Not serious | Low | 0.75 [0.40; 1.40] | Low | . | Serious | Very low | NMA estimate was used because incoherence was not available. Imprecision was rated as Serious. |
| Shengxue Granules+OX | Ya Dan Zi Oil Emulsion Injection+OX | 0 | . | . | . |  |  |  |  |  | 0.89 [0.52; 1.54] | Low | Low | Not serious | Low | 0.89 [0.52; 1.54] | Low | . | Serious | Very low | NMA estimate was used because incoherence was not available. Imprecision was rated as Serious. |
| Shengxue Granules+OX | Yangzheng Xiaoji Capsules+OX | 0 | . | . | . |  |  |  |  |  | 0.87 [0.48; 1.56] | Low | High | Not serious | Low | 0.87 [0.48; 1.56] | Low | . | Serious | Very low | NMA estimate was used because incoherence was not available. Imprecision was rated as Serious. |
| Shenmai Injection+OX | Shenqi Fuzheng Injection+OX | 0 | . | . | . |  |  |  |  |  | 1.02 [0.75; 1.38] | High | Low | Not serious | Low | 1.02 [0.75; 1.38] | Low | . | Serious | Very low | NMA estimate was used because incoherence was not available. Imprecision was rated as Serious. |
| Shenmai Injection+OX | Weimaining Capsules+OX | 0 | . | . | . |  |  |  |  |  | 1.04 [0.45; 2.40] | High | High | Not serious | High | 1.04 [0.45; 2.40] | High | . | Serious | Moderate | NMA estimate was used because incoherence was not available. Imprecision was rated as Serious. |
| Shenmai Injection+OX | Xiaoaiping Injection+OX | 0 | . | . | . |  |  |  |  |  | 0.98 [0.71; 1.35] | High | Moderate | Not serious | Moderate | 0.98 [0.71; 1.35] | Moderate | . | Serious | Low | NMA estimate was used because incoherence was not available. Imprecision was rated as Serious. |
| Shenmai Injection+OX | Xihuang Capsules+OX | 0 | . | . | . |  |  |  |  |  | 0.82 [0.53; 1.27] | High | Low | Not serious | Low | 0.82 [0.53; 1.27] | Low | . | Serious | Very low | NMA estimate was used because incoherence was not available. Imprecision was rated as Serious. |
| Shenmai Injection+OX | Ya Dan Zi Oil Emulsion Injection+OX | 0 | . | . | . |  |  |  |  |  | 0.98 [0.72; 1.34] | High | Low | Not serious | Low | 0.98 [0.72; 1.34] | Low | . | Serious | Very low | NMA estimate was used because incoherence was not available. Imprecision was rated as Serious. |
| Shenmai Injection+OX | Yangzheng Xiaoji Capsules+OX | 0 | . | . | . |  |  |  |  |  | 0.96 [0.65; 1.40] | High | High | Not serious | High | 0.96 [0.65; 1.40] | High | . | Serious | Moderate | NMA estimate was used because incoherence was not available. Imprecision was rated as Serious. |
| Shenqi Fuzheng Injection+OX | Weimaining Capsules+OX | 0 | . | . | . |  |  |  |  |  | 1.02 [0.46; 2.25] | Low | High | Not serious | Low | 1.02 [0.46; 2.25] | Low | . | Serious | Very low | NMA estimate was used because incoherence was not available. Imprecision was rated as Serious. |
| Shenqi Fuzheng Injection+OX | Xiaoaiping Injection+OX | 0 | . | . | . |  |  |  |  |  | 0.96 [0.80; 1.15] | Low | Moderate | Not serious | Low | 0.96 [0.80; 1.15] | Low | . | Serious | Very low | NMA estimate was used because incoherence was not available. Imprecision was rated as Serious. |
| Shenqi Fuzheng Injection+OX | Xihuang Capsules+OX | 0 | . | . | . |  |  |  |  |  | 0.81 [0.57; 1.14] | Low | Low | Not serious | Low | 0.81 [0.57; 1.14] | Low | . | Serious | Very low | NMA estimate was used because incoherence was not available. Imprecision was rated as Serious. |
| Shenqi Fuzheng Injection+OX | Ya Dan Zi Oil Emulsion Injection+OX | 0 | . | . | . |  |  |  |  |  | 0.96 [0.82; 1.13] | Low | Low | Not serious | Low | 0.96 [0.82; 1.13] | Low | . | Serious | Very low | NMA estimate was used because incoherence was not available. Imprecision was rated as Serious. |
| Shenqi Fuzheng Injection+OX | Yangzheng Xiaoji Capsules+OX | 0 | . | . | . |  |  |  |  |  | 0.94 [0.71; 1.23] | Low | High | Not serious | Low | 0.94 [0.71; 1.23] | Low | . | Serious | Very low | NMA estimate was used because incoherence was not available. Imprecision was rated as Serious. |
| Weimaining Capsules+OX | Xiaoaiping Injection+OX | 0 | . | . | . |  |  |  |  |  | 0.94 [0.42; 2.09] | High | Moderate | Not serious | Moderate | 0.94 [0.42; 2.09] | Moderate | . | Serious | Low | NMA estimate was used because incoherence was not available. Imprecision was rated as Serious. |
| Weimaining Capsules+OX | Xihuang Capsules+OX | 0 | . | . | . |  |  |  |  |  | 0.79 [0.34; 1.85] | High | Low | Not serious | Low | 0.79 [0.34; 1.85] | Low | . | Serious | Very low | NMA estimate was used because incoherence was not available. Imprecision was rated as Serious. |
| Weimaining Capsules+OX | Ya Dan Zi Oil Emulsion Injection+OX | 0 | . | . | . |  |  |  |  |  | 0.94 [0.43; 2.09] | High | Low | Not serious | Low | 0.94 [0.43; 2.09] | Low | . | Serious | Very low | NMA estimate was used because incoherence was not available. Imprecision was rated as Serious. |
| Weimaining Capsules+OX | Yangzheng Xiaoji Capsules+OX | 0 | . | . | . |  |  |  |  |  | 0.92 [0.40; 2.09] | High | High | Not serious | High | 0.92 [0.40; 2.09] | High | . | Serious | Moderate | NMA estimate was used because incoherence was not available. Imprecision was rated as Serious. |
| Xiaoaiping Injection+OX | Xihuang Capsules+OX | 0 | . | . | . |  |  |  |  |  | 0.84 [0.59; 1.21] | Moderate | Low | Not serious | Low | 0.84 [0.59; 1.21] | Low | . | Serious | Very low | NMA estimate was used because incoherence was not available. Imprecision was rated as Serious. |
| Xiaoaiping Injection+OX | Ya Dan Zi Oil Emulsion Injection+OX | 0 | . | . | . |  |  |  |  |  | 1.01 [0.83; 1.22] | Moderate | Low | Not serious | Low | 1.01 [0.83; 1.22] | Low | . | Serious | Very low | NMA estimate was used because incoherence was not available. Imprecision was rated as Serious. |
| Xiaoaiping Injection+OX | Yangzheng Xiaoji Capsules+OX | 0 | . | . | . |  |  |  |  |  | 0.98 [0.73; 1.31] | Moderate | High | Not serious | Moderate | 0.98 [0.73; 1.31] | Moderate | . | Serious | Low | NMA estimate was used because incoherence was not available. Imprecision was rated as Serious. |
| Xihuang Capsules+OX | Ya Dan Zi Oil Emulsion Injection+OX | 0 | . | . | . |  |  |  |  |  | 1.19 [0.84; 1.70] | Low | Low | Not serious | Low | 1.19 [0.84; 1.70] | Low | . | Serious | Very low | NMA estimate was used because incoherence was not available. Imprecision was rated as Serious. |
| Xihuang Capsules+OX | Yangzheng Xiaoji Capsules+OX | 0 | . | . | . |  |  |  |  |  | 1.16 [0.77; 1.76] | Low | High | Not serious | Low | 1.16 [0.77; 1.76] | Low | . | Serious | Very low | NMA estimate was used because incoherence was not available. Imprecision was rated as Serious. |
| Ya Dan Zi Oil Emulsion Injection+OX | Yangzheng Xiaoji Capsules+OX | 0 | . | . | . |  |  |  |  |  | 0.97 [0.74; 1.29] | Low | High | Not serious | Low | 0.97 [0.74; 1.29] | Low | . | Serious | Very low | NMA estimate was used because incoherence was not available. Imprecision was rated as Serious. |

1. QoL

| **Arm_1** | **Arm_2** | **No_of_study** | **Sample_size** | **I2** | **Direct_estimate** | **ROB** | **Inconsistency** | **Indirectness** | **Publication_bias** | **Direct_rating_without_imprecision** | **Indirect_estimate** | **Certainty_of_evidence_for_arm1** | **Certainty_of_evidence_for_arm2** | **Intransitivity** | **Indirect_rating_without_imprecision** | **Network_meta_analysis** | **Higher_rating_of_direct_and_indirect_without_imprecision** | **Incoherence** | **NMA_Imprecision** | **Final_network_rating** | **Final_rating_reason** |
| --- | --- | --- | --- | --- | --- | --- | --- | --- | --- | --- | --- | --- | --- | --- | --- | --- | --- | --- | --- | --- | --- |
| Aidi Injection+OX | Astragalus Polysaccharides+OX | 0 | . | . | . |  |  |  |  |  | 0.98 [0.70; 1.38] | Moderate | Low | Not serious | Low | 0.98 [0.70; 1.38] | Low | . | Serious | Very low | NMA estimate was used because incoherence was not available. Imprecision was rated as Serious. |
| Aidi Injection+OX | Astragalus preparations+OX | 0 | . | . | . |  |  |  |  |  | 0.62 [0.38; 1.01] | Moderate | Low | Not serious | Low | 0.62 [0.38; 1.01] | Low | . | Serious | Very low | NMA estimate was used because incoherence was not available. Imprecision was rated as Serious. |
| Aidi Injection+OX | Compound Kushen Injection+OX | 0 | . | . | . |  |  |  |  |  | 1.16 [0.90; 1.49] | Moderate | Low | Not serious | Low | 1.16 [0.90; 1.49] | Low | . | Serious | Very low | NMA estimate was used because incoherence was not available. Imprecision was rated as Serious. |
| Aidi Injection+OX | Huachansu preparations+OX | 0 | . | . | . |  |  |  |  |  | 1.29 [0.94; 1.78] | Moderate | Low | Not serious | Low | 1.29 [0.94; 1.78] | Low | . | Serious | Very low | NMA estimate was used because incoherence was not available. Imprecision was rated as Serious. |
| Aidi Injection+OX | Kanglaite Injection+OX | 0 | . | . | . |  |  |  |  |  | 1.13 [0.86; 1.48] | Moderate | High | Not serious | Moderate | 1.13 [0.86; 1.48] | Moderate | . | Serious | Low | NMA estimate was used because incoherence was not available. Imprecision was rated as Serious. |
| Aidi Injection+OX | Lentinan Injection+OX | 0 | . | . | . |  |  |  |  |  | 0.90 [0.57; 1.43] | Moderate | High | Not serious | Moderate | 0.90 [0.57; 1.43] | Moderate | . | Serious | Low | NMA estimate was used because incoherence was not available. Imprecision was rated as Serious. |
| Aidi Injection+OX | Lentinan+OX | 0 | . | . | . |  |  |  |  |  | 1.08 [0.75; 1.56] | Moderate | High | Not serious | Moderate | 1.08 [0.75; 1.56] | Moderate | . | Serious | Low | NMA estimate was used because incoherence was not available. Imprecision was rated as Serious. |
| Aidi Injection+OX | OX | 3 | 196 | 0.4% | 1.35 [1.12; 1.64] | Serious | Not serious | Not serious | Undetected | Moderate | . |  |  |  |  | 1.35 [1.12; 1.64] | Moderate | . | Not serious | Moderate | NMA estimate was used because incoherence was not available. Imprecision was rated as Serious. |
| Aidi Injection+OX | Shenqi Fuzheng Injection+OX | 0 | . | . | . |  |  |  |  |  | 1.06 [0.82; 1.38] | Moderate | Low | Not serious | Low | 1.06 [0.82; 1.38] | Low | . | Serious | Very low | NMA estimate was used because incoherence was not available. Imprecision was rated as Serious. |
| Aidi Injection+OX | Xiaoaiping Injection+OX | 0 | . | . | . |  |  |  |  |  | 1.13 [0.77; 1.66] | Moderate | Low | Not serious | Low | 1.13 [0.77; 1.66] | Low | . | Serious | Very low | NMA estimate was used because incoherence was not available. Imprecision was rated as Serious. |
| Aidi Injection+OX | Ya Dan Zi Oil Emulsion Injection+OX | 0 | . | . | . |  |  |  |  |  | 1.07 [0.82; 1.41] | Moderate | Low | Not serious | Low | 1.07 [0.82; 1.41] | Low | . | Serious | Very low | NMA estimate was used because incoherence was not available. Imprecision was rated as Serious. |
| Aidi Injection+OX | Yangzheng Xiaoji Capsules+OX | 0 | . | . | . |  |  |  |  |  | 0.43 [0.26; 0.71] | Moderate | Low | Not serious | Low | 0.43 [0.26; 0.71] | Low | . | Serious | Very low | NMA estimate was used because incoherence was not available. Imprecision was rated as Serious. |
| Astragalus Polysaccharides+OX | Astragalus preparations+OX | 0 | . | . | . |  |  |  |  |  | 0.63 [0.37; 1.08] | Low | Low | Not serious | Low | 0.63 [0.37; 1.08] | Low | . | Serious | Very low | NMA estimate was used because incoherence was not available. Imprecision was rated as Serious. |
| Astragalus Polysaccharides+OX | Compound Kushen Injection+OX | 0 | . | . | . |  |  |  |  |  | 1.18 [0.85; 1.64] | Low | Low | Not serious | Low | 1.18 [0.85; 1.64] | Low | . | Serious | Very low | NMA estimate was used because incoherence was not available. Imprecision was rated as Serious. |
| Astragalus Polysaccharides+OX | Huachansu preparations+OX | 0 | . | . | . |  |  |  |  |  | 1.32 [0.90; 1.93] | Low | Low | Not serious | Low | 1.32 [0.90; 1.93] | Low | . | Serious | Very low | NMA estimate was used because incoherence was not available. Imprecision was rated as Serious. |
| Astragalus Polysaccharides+OX | Kanglaite Injection+OX | 0 | . | . | . |  |  |  |  |  | 1.15 [0.82; 1.62] | Low | High | Not serious | Low | 1.15 [0.82; 1.62] | Low | . | Serious | Very low | NMA estimate was used because incoherence was not available. Imprecision was rated as Serious. |
| Astragalus Polysaccharides+OX | Lentinan Injection+OX | 0 | . | . | . |  |  |  |  |  | 0.92 [0.56; 1.52] | Low | High | Not serious | Low | 0.92 [0.56; 1.52] | Low | . | Serious | Very low | NMA estimate was used because incoherence was not available. Imprecision was rated as Serious. |
| Astragalus Polysaccharides+OX | Lentinan+OX | 0 | . | . | . |  |  |  |  |  | 1.10 [0.72; 1.68] | Low | High | Not serious | Low | 1.10 [0.72; 1.68] | Low | . | Serious | Very low | NMA estimate was used because incoherence was not available. Imprecision was rated as Serious. |
| Astragalus Polysaccharides+OX | OX | 1 | 86 | . | 1.38 [1.04; 1.83] | Very serious | Not serious | Not serious | Undetected | Low | . |  |  |  |  | 1.38 [1.04; 1.83] | Low | . | Not serious | Low | NMA estimate was used because incoherence was not available. Imprecision was rated as Serious. |
| Astragalus Polysaccharides+OX | Shenqi Fuzheng Injection+OX | 0 | . | . | . |  |  |  |  |  | 1.09 [0.78; 1.52] | Low | Low | Not serious | Low | 1.09 [0.78; 1.52] | Low | . | Serious | Very low | NMA estimate was used because incoherence was not available. Imprecision was rated as Serious. |
| Astragalus Polysaccharides+OX | Xiaoaiping Injection+OX | 0 | . | . | . |  |  |  |  |  | 1.16 [0.75; 1.79] | Low | Low | Not serious | Low | 1.16 [0.75; 1.79] | Low | . | Serious | Very low | NMA estimate was used because incoherence was not available. Imprecision was rated as Serious. |
| Astragalus Polysaccharides+OX | Ya Dan Zi Oil Emulsion Injection+OX | 0 | . | . | . |  |  |  |  |  | 1.10 [0.78; 1.55] | Low | Low | Not serious | Low | 1.10 [0.78; 1.55] | Low | . | Serious | Very low | NMA estimate was used because incoherence was not available. Imprecision was rated as Serious. |
| Astragalus Polysaccharides+OX | Yangzheng Xiaoji Capsules+OX | 0 | . | . | . |  |  |  |  |  | 0.44 [0.26; 0.76] | Low | Low | Not serious | Low | 0.44 [0.26; 0.76] | Low | . | Serious | Very low | NMA estimate was used because incoherence was not available. Imprecision was rated as Serious. |
| Astragalus preparations+OX | Compound Kushen Injection+OX | 0 | . | . | . |  |  |  |  |  | 1.87 [1.16; 3.02] | Low | Low | Not serious | Low | 1.87 [1.16; 3.02] | Low | . | Not serious | Low | NMA estimate was used because incoherence was not available. Imprecision was rated as Serious. |
| Astragalus preparations+OX | Huachansu preparations+OX | 0 | . | . | . |  |  |  |  |  | 2.09 [1.24; 3.50] | Low | Low | Not serious | Low | 2.09 [1.24; 3.50] | Low | . | Not serious | Low | NMA estimate was used because incoherence was not available. Imprecision was rated as Serious. |
| Astragalus preparations+OX | Kanglaite Injection+OX | 0 | . | . | . |  |  |  |  |  | 1.82 [1.11; 2.97] | Low | High | Not serious | Low | 1.82 [1.11; 2.97] | Low | . | Not serious | Low | NMA estimate was used because incoherence was not available. Imprecision was rated as Serious. |
| Astragalus preparations+OX | Lentinan Injection+OX | 0 | . | . | . |  |  |  |  |  | 1.46 [0.79; 2.69] | Low | High | Not serious | Low | 1.46 [0.79; 2.69] | Low | . | Serious | Very low | NMA estimate was used because incoherence was not available. Imprecision was rated as Serious. |
| Astragalus preparations+OX | Lentinan+OX | 0 | . | . | . |  |  |  |  |  | 1.75 [1.01; 3.02] | Low | High | Not serious | Low | 1.75 [1.01; 3.02] | Low | . | Not serious | Low | NMA estimate was used because incoherence was not available. Imprecision was rated as Serious. |
| Astragalus preparations+OX | OX | 1 | 67 | . | 2.18 [1.39; 3.43] | Very serious | Not serious | Not serious | Undetected | Low | . |  |  |  |  | 2.18 [1.39; 3.43] | Low | . | Not serious | Low | NMA estimate was used because incoherence was not available. Imprecision was rated as Serious. |
| Astragalus preparations+OX | Shenqi Fuzheng Injection+OX | 0 | . | . | . |  |  |  |  |  | 1.72 [1.06; 2.79] | Low | Low | Not serious | Low | 1.72 [1.06; 2.79] | Low | . | Not serious | Low | NMA estimate was used because incoherence was not available. Imprecision was rated as Serious. |
| Astragalus preparations+OX | Xiaoaiping Injection+OX | 0 | . | . | . |  |  |  |  |  | 1.83 [1.05; 3.20] | Low | Low | Not serious | Low | 1.83 [1.05; 3.20] | Low | . | Not serious | Low | NMA estimate was used because incoherence was not available. Imprecision was rated as Serious. |
| Astragalus preparations+OX | Ya Dan Zi Oil Emulsion Injection+OX | 0 | . | . | . |  |  |  |  |  | 1.74 [1.06; 2.84] | Low | Low | Not serious | Low | 1.74 [1.06; 2.84] | Low | . | Not serious | Low | NMA estimate was used because incoherence was not available. Imprecision was rated as Serious. |
| Astragalus preparations+OX | Yangzheng Xiaoji Capsules+OX | 0 | . | . | . |  |  |  |  |  | 0.70 [0.37; 1.33] | Low | Low | Not serious | Low | 0.70 [0.37; 1.33] | Low | . | Serious | Very low | NMA estimate was used because incoherence was not available. Imprecision was rated as Serious. |
| Compound Kushen Injection+OX | Huachansu preparations+OX | 0 | . | . | . |  |  |  |  |  | 1.12 [0.83; 1.51] | Low | Low | Not serious | Low | 1.12 [0.83; 1.51] | Low | . | Serious | Very low | NMA estimate was used because incoherence was not available. Imprecision was rated as Serious. |
| Compound Kushen Injection+OX | Kanglaite Injection+OX | 0 | . | . | . |  |  |  |  |  | 0.97 [0.76; 1.26] | Low | High | Not serious | Low | 0.97 [0.76; 1.26] | Low | . | Serious | Very low | NMA estimate was used because incoherence was not available. Imprecision was rated as Serious. |
| Compound Kushen Injection+OX | Lentinan Injection+OX | 0 | . | . | . |  |  |  |  |  | 0.78 [0.50; 1.22] | Low | High | Not serious | Low | 0.78 [0.50; 1.22] | Low | . | Serious | Very low | NMA estimate was used because incoherence was not available. Imprecision was rated as Serious. |
| Compound Kushen Injection+OX | Lentinan+OX | 0 | . | . | . |  |  |  |  |  | 0.93 [0.66; 1.33] | Low | High | Not serious | Low | 0.93 [0.66; 1.33] | Low | . | Serious | Very low | NMA estimate was used because incoherence was not available. Imprecision was rated as Serious. |
| Compound Kushen Injection+OX | OX | 2 | 250 | 0.0% | 1.17 [0.99; 1.38] | Very serious | Not serious | Not serious | Undetected | Low | . |  |  |  |  | 1.17 [0.99; 1.38] | Low | . | Serious | Very low | NMA estimate was used because incoherence was not available. Imprecision was rated as Serious. |
| Compound Kushen Injection+OX | Shenqi Fuzheng Injection+OX | 0 | . | . | . |  |  |  |  |  | 0.92 [0.72; 1.17] | Low | Low | Not serious | Low | 0.92 [0.72; 1.17] | Low | . | Serious | Very low | NMA estimate was used because incoherence was not available. Imprecision was rated as Serious. |
| Compound Kushen Injection+OX | Xiaoaiping Injection+OX | 0 | . | . | . |  |  |  |  |  | 0.98 [0.68; 1.42] | Low | Low | Not serious | Low | 0.98 [0.68; 1.42] | Low | . | Serious | Very low | NMA estimate was used because incoherence was not available. Imprecision was rated as Serious. |
| Compound Kushen Injection+OX | Ya Dan Zi Oil Emulsion Injection+OX | 0 | . | . | . |  |  |  |  |  | 0.93 [0.72; 1.20] | Low | Low | Not serious | Low | 0.93 [0.72; 1.20] | Low | . | Serious | Very low | NMA estimate was used because incoherence was not available. Imprecision was rated as Serious. |
| Compound Kushen Injection+OX | Yangzheng Xiaoji Capsules+OX | 0 | . | . | . |  |  |  |  |  | 0.37 [0.23; 0.61] | Low | Low | Not serious | Low | 0.37 [0.23; 0.61] | Low | . | Serious | Very low | NMA estimate was used because incoherence was not available. Imprecision was rated as Serious. |
| Huachansu preparations+OX | Kanglaite Injection+OX | 0 | . | . | . |  |  |  |  |  | 0.87 [0.63; 1.20] | Low | High | Not serious | Low | 0.87 [0.63; 1.20] | Low | . | Serious | Very low | NMA estimate was used because incoherence was not available. Imprecision was rated as Serious. |
| Huachansu preparations+OX | Lentinan Injection+OX | 0 | . | . | . |  |  |  |  |  | 0.70 [0.43; 1.14] | Low | High | Not serious | Low | 0.70 [0.43; 1.14] | Low | . | Serious | Very low | NMA estimate was used because incoherence was not available. Imprecision was rated as Serious. |
| Huachansu preparations+OX | Lentinan+OX | 0 | . | . | . |  |  |  |  |  | 0.84 [0.56; 1.25] | Low | High | Not serious | Low | 0.84 [0.56; 1.25] | Low | . | Serious | Very low | NMA estimate was used because incoherence was not available. Imprecision was rated as Serious. |
| Huachansu preparations+OX | OX | 1 | 50 | . | 1.05 [0.81; 1.35] | Very serious | Not serious | Not serious | Undetected | Low | . |  |  |  |  | 1.05 [0.81; 1.35] | Low | . | Serious | Very low | NMA estimate was used because incoherence was not available. Imprecision was rated as Serious. |
| Huachansu preparations+OX | Shenqi Fuzheng Injection+OX | 0 | . | . | . |  |  |  |  |  | 0.82 [0.60; 1.12] | Low | Low | Not serious | Low | 0.82 [0.60; 1.12] | Low | . | Serious | Very low | NMA estimate was used because incoherence was not available. Imprecision was rated as Serious. |
| Huachansu preparations+OX | Xiaoaiping Injection+OX | 0 | . | . | . |  |  |  |  |  | 0.88 [0.58; 1.33] | Low | Low | Not serious | Low | 0.88 [0.58; 1.33] | Low | . | Serious | Very low | NMA estimate was used because incoherence was not available. Imprecision was rated as Serious. |
| Huachansu preparations+OX | Ya Dan Zi Oil Emulsion Injection+OX | 0 | . | . | . |  |  |  |  |  | 0.83 [0.60; 1.14] | Low | Low | Not serious | Low | 0.83 [0.60; 1.14] | Low | . | Serious | Very low | NMA estimate was used because incoherence was not available. Imprecision was rated as Serious. |
| Huachansu preparations+OX | Yangzheng Xiaoji Capsules+OX | 0 | . | . | . |  |  |  |  |  | 0.33 [0.20; 0.57] | Low | Low | Not serious | Low | 0.33 [0.20; 0.57] | Low | . | Serious | Very low | NMA estimate was used because incoherence was not available. Imprecision was rated as Serious. |
| Kanglaite Injection+OX | Lentinan Injection+OX | 0 | . | . | . |  |  |  |  |  | 0.80 [0.51; 1.27] | High | High | Not serious | High | 0.80 [0.51; 1.27] | High | . | Serious | Moderate | NMA estimate was used because incoherence was not available. Imprecision was rated as Serious. |
| Kanglaite Injection+OX | Lentinan+OX | 0 | . | . | . |  |  |  |  |  | 0.96 [0.66; 1.39] | High | High | Not serious | High | 0.96 [0.66; 1.39] | High | . | Serious | Moderate | NMA estimate was used because incoherence was not available. Imprecision was rated as Serious. |
| Kanglaite Injection+OX | OX | 3 | 201 | 9.2% | 1.20 [0.99; 1.45] | Not serious | Not serious | Not serious | Undetected | High | . |  |  |  |  | 1.20 [0.99; 1.45] | High | . | Serious | Moderate | NMA estimate was used because incoherence was not available. Imprecision was rated as Serious. |
| Kanglaite Injection+OX | Shenqi Fuzheng Injection+OX | 0 | . | . | . |  |  |  |  |  | 0.94 [0.72; 1.23] | High | Low | Not serious | Low | 0.94 [0.72; 1.23] | Low | . | Serious | Very low | NMA estimate was used because incoherence was not available. Imprecision was rated as Serious. |
| Kanglaite Injection+OX | Xiaoaiping Injection+OX | 0 | . | . | . |  |  |  |  |  | 1.01 [0.68; 1.48] | High | Low | Not serious | Low | 1.01 [0.68; 1.48] | Low | . | Serious | Very low | NMA estimate was used because incoherence was not available. Imprecision was rated as Serious. |
| Kanglaite Injection+OX | Ya Dan Zi Oil Emulsion Injection+OX | 0 | . | . | . |  |  |  |  |  | 0.95 [0.72; 1.25] | High | Low | Not serious | Low | 0.95 [0.72; 1.25] | Low | . | Serious | Very low | NMA estimate was used because incoherence was not available. Imprecision was rated as Serious. |
| Kanglaite Injection+OX | Yangzheng Xiaoji Capsules+OX | 0 | . | . | . |  |  |  |  |  | 0.38 [0.23; 0.63] | High | Low | Not serious | Low | 0.38 [0.23; 0.63] | Low | . | Serious | Very low | NMA estimate was used because incoherence was not available. Imprecision was rated as Serious. |
| Lentinan Injection+OX | Lentinan+OX | 0 | . | . | . |  |  |  |  |  | 1.20 [0.71; 2.01] | High | High | Not serious | High | 1.20 [0.71; 2.01] | High | . | Serious | Moderate | NMA estimate was used because incoherence was not available. Imprecision was rated as Serious. |
| Lentinan Injection+OX | OX | 1 | 68 | . | 1.50 [0.99; 2.27] | Not serious | Not serious | Not serious | Undetected | High | . |  |  |  |  | 1.50 [0.99; 2.27] | High | . | Serious | Moderate | NMA estimate was used because incoherence was not available. Imprecision was rated as Serious. |
| Lentinan Injection+OX | Shenqi Fuzheng Injection+OX | 0 | . | . | . |  |  |  |  |  | 1.18 [0.75; 1.85] | High | Low | Not serious | Low | 1.18 [0.75; 1.85] | Low | . | Serious | Very low | NMA estimate was used because incoherence was not available. Imprecision was rated as Serious. |
| Lentinan Injection+OX | Xiaoaiping Injection+OX | 0 | . | . | . |  |  |  |  |  | 1.26 [0.74; 2.14] | High | Low | Not serious | Low | 1.26 [0.74; 2.14] | Low | . | Serious | Very low | NMA estimate was used because incoherence was not available. Imprecision was rated as Serious. |
| Lentinan Injection+OX | Ya Dan Zi Oil Emulsion Injection+OX | 0 | . | . | . |  |  |  |  |  | 1.19 [0.75; 1.88] | High | Low | Not serious | Low | 1.19 [0.75; 1.88] | Low | . | Serious | Very low | NMA estimate was used because incoherence was not available. Imprecision was rated as Serious. |
| Lentinan Injection+OX | Yangzheng Xiaoji Capsules+OX | 0 | . | . | . |  |  |  |  |  | 0.48 [0.26; 0.89] | High | Low | Not serious | Low | 0.48 [0.26; 0.89] | Low | . | Serious | Very low | NMA estimate was used because incoherence was not available. Imprecision was rated as Serious. |
| Lentinan+OX | OX | 1 | 86 | . | 1.25 [0.91; 1.71] | Not serious | Not serious | Not serious | Undetected | High | . |  |  |  |  | 1.25 [0.91; 1.71] | High | . | Serious | Moderate | NMA estimate was used because incoherence was not available. Imprecision was rated as Serious. |
| Lentinan+OX | Shenqi Fuzheng Injection+OX | 0 | . | . | . |  |  |  |  |  | 0.98 [0.69; 1.41] | High | Low | Not serious | Low | 0.98 [0.69; 1.41] | Low | . | Serious | Very low | NMA estimate was used because incoherence was not available. Imprecision was rated as Serious. |
| Lentinan+OX | Xiaoaiping Injection+OX | 0 | . | . | . |  |  |  |  |  | 1.05 [0.66; 1.65] | High | Low | Not serious | Low | 1.05 [0.66; 1.65] | Low | . | Serious | Very low | NMA estimate was used because incoherence was not available. Imprecision was rated as Serious. |
| Lentinan+OX | Ya Dan Zi Oil Emulsion Injection+OX | 0 | . | . | . |  |  |  |  |  | 0.99 [0.69; 1.44] | High | Low | Not serious | Low | 0.99 [0.69; 1.44] | Low | . | Serious | Very low | NMA estimate was used because incoherence was not available. Imprecision was rated as Serious. |
| Lentinan+OX | Yangzheng Xiaoji Capsules+OX | 0 | . | . | . |  |  |  |  |  | 0.40 [0.23; 0.70] | High | Low | Not serious | Low | 0.40 [0.23; 0.70] | Low | . | Serious | Very low | NMA estimate was used because incoherence was not available. Imprecision was rated as Serious. |
| Shenqi Fuzheng Injection+OX | OX | 3 | 196 | 51.0% | 1.27 [1.06; 1.52] | Very serious | Not serious | Not serious | Undetected | Low | . |  |  |  |  | 1.27 [1.06; 1.52] | Low | . | Not serious | Low | NMA estimate was used because incoherence was not available. Imprecision was rated as Serious. |
| Xiaoaiping Injection+OX | OX | 2 | 104 | 62.2% | 1.19 [0.86; 1.66] | Very serious | Not serious | Not serious | Undetected | Low | . |  |  |  |  | 1.19 [0.86; 1.66] | Low | . | Serious | Very low | NMA estimate was used because incoherence was not available. Imprecision was rated as Serious. |
| Ya Dan Zi Oil Emulsion Injection+OX | OX | 2 | 202 | 0.0% | 1.26 [1.03; 1.53] | Very serious | Not serious | Not serious | Undetected | Low | . |  |  |  |  | 1.26 [1.03; 1.53] | Low | . | Not serious | Low | NMA estimate was used because incoherence was not available. Imprecision was rated as Serious. |
| Yangzheng Xiaoji Capsules+OX | OX | 1 | 100 | . | 3.13 [1.97; 4.98] | Very serious | Not serious | Not serious | Undetected | Low | . |  |  |  |  | 3.13 [1.97; 4.98] | Low | . | Not serious | Low | NMA estimate was used because incoherence was not available. Imprecision was rated as Serious. |
| Shenqi Fuzheng Injection+OX | Xiaoaiping Injection+OX | 0 | . | . | . |  |  |  |  |  | 1.07 [0.73; 1.55] | Low | Low | Not serious | Low | 1.07 [0.73; 1.55] | Low | . | Serious | Very low | NMA estimate was used because incoherence was not available. Imprecision was rated as Serious. |
| Shenqi Fuzheng Injection+OX | Ya Dan Zi Oil Emulsion Injection+OX | 0 | . | . | . |  |  |  |  |  | 1.01 [0.77; 1.32] | Low | Low | Not serious | Low | 1.01 [0.77; 1.32] | Low | . | Serious | Very low | NMA estimate was used because incoherence was not available. Imprecision was rated as Serious. |
| Shenqi Fuzheng Injection+OX | Yangzheng Xiaoji Capsules+OX | 0 | . | . | . |  |  |  |  |  | 0.41 [0.25; 0.67] | Low | Low | Not serious | Low | 0.41 [0.25; 0.67] | Low | . | Serious | Very low | NMA estimate was used because incoherence was not available. Imprecision was rated as Serious. |
| Xiaoaiping Injection+OX | Ya Dan Zi Oil Emulsion Injection+OX | 0 | . | . | . |  |  |  |  |  | 0.95 [0.64; 1.39] | Low | Low | Not serious | Low | 0.95 [0.64; 1.39] | Low | . | Serious | Very low | NMA estimate was used because incoherence was not available. Imprecision was rated as Serious. |
| Xiaoaiping Injection+OX | Yangzheng Xiaoji Capsules+OX | 0 | . | . | . |  |  |  |  |  | 0.38 [0.22; 0.67] | Low | Low | Not serious | Low | 0.38 [0.22; 0.67] | Low | . | Serious | Very low | NMA estimate was used because incoherence was not available. Imprecision was rated as Serious. |
| Ya Dan Zi Oil Emulsion Injection+OX | Yangzheng Xiaoji Capsules+OX | 0 | . | . | . |  |  |  |  |  | 0.40 [0.24; 0.66] | Low | Low | Not serious | Low | 0.40 [0.24; 0.66] | Low | . | Serious | Very low | NMA estimate was used because incoherence was not available. Imprecision was rated as Serious. |

1. **TCM syndrome score**

| **Arm_1** | **Arm_2** | **No_of_study** | **Sample_size** | **I2** | **Direct_estimate** | **ROB** | **Inconsistency** | **Indirectness** | **Publication_bias** | **Direct_rating_without_imprecision** | **Indirect_estimate** | **Certainty_of_evidence_for_arm1** | **Certainty_of_evidence_for_arm2** | **Intransitivity** | **Indirect_rating_without_imprecision** | **Network_meta_analysis** | **Higher_rating_of_direct_and_indirect_without_imprecision** | **Incoherence** | **NMA_Imprecision** | **Final_network_rating** | **Final_rating_reason** |
| --- | --- | --- | --- | --- | --- | --- | --- | --- | --- | --- | --- | --- | --- | --- | --- | --- | --- | --- | --- | --- | --- |
| Aidi Injection+OX | Compound Kushen Injection+OX | 0 | . | . | . |  |  |  |  |  | 1.07 [-3.03; 5.18] | Low | Low | Not serious | Low | 1.07 [-3.03; 5.18] | Low | . | Serious | Very low | NMA estimate was used because incoherence was not available. Imprecision was rated as Serious. |
| Aidi Injection+OX | OX | 1 | 60 | . | -2.69 [-6.05; 0.67] | Very serious | Not serious | Not serious | Undetected | Low | . |  |  |  |  | -2.69 [-6.05; 0.67] | Low | . | Serious | Very low | NMA estimate was used because incoherence was not available. Imprecision was rated as Serious. |
| Aidi Injection+OX | Shenqi Fuzheng Injection+OX | 0 | . | . | . |  |  |  |  |  | -4.34 [-8.22; -0.46] | Low | Very low | Not serious | Very low | -4.34 [-8.22; -0.46] | Very low | . | Not serious | Very low | NMA estimate was used because incoherence was not available. Imprecision was rated as Serious. |
| Aidi Injection+OX | Yangzheng Xiaoji Capsules+OX | 0 | . | . | . |  |  |  |  |  | -1.48 [-6.09; 3.13] | Low | High | Not serious | Low | -1.48 [-6.09; 3.13] | Low | . | Serious | Very low | NMA estimate was used because incoherence was not available. Imprecision was rated as Serious. |
| Compound Kushen Injection+OX | OX | 2 | 174 | 11.0% | -3.76 [-6.12; -1.41] | Very serious | Not serious | Not serious | Undetected | Low | . |  |  |  |  | -3.76 [-6.12; -1.41] | Low | . | Not serious | Low | NMA estimate was used because incoherence was not available. Imprecision was rated as Serious. |
| Compound Kushen Injection+OX | Shenqi Fuzheng Injection+OX | 0 | . | . | . |  |  |  |  |  | -5.41 [-8.47; -2.36] | Low | Very low | Not serious | Very low | -5.41 [-8.47; -2.36] | Very low | . | Not serious | Very low | NMA estimate was used because incoherence was not available. Imprecision was rated as Serious. |
| Compound Kushen Injection+OX | Yangzheng Xiaoji Capsules+OX | 0 | . | . | . |  |  |  |  |  | -2.55 [-6.49; 1.38] | Low | High | Not serious | Low | -2.55 [-6.49; 1.38] | Low | . | Serious | Very low | NMA estimate was used because incoherence was not available. Imprecision was rated as Serious. |
| Shenqi Fuzheng Injection+OX | OX | 3 | 231 | 96.4% | 1.65 [-0.29; 3.60] | Very serious | Very serious | Not serious | Undetected | Very low | . |  |  |  |  | 1.65 [-0.29; 3.60] | Very low | . | Serious | Very low | NMA estimate was used because incoherence was not available. Imprecision was rated as Serious. |
| Yangzheng Xiaoji Capsules+OX | OX | 1 | 80 | . | -1.21 [-4.36; 1.94] | Not serious | Not serious | Not serious | Undetected | High | . |  |  |  |  | -1.21 [-4.36; 1.94] | High | . | Serious | Moderate | NMA estimate was used because incoherence was not available. Imprecision was rated as Serious. |
| Shenqi Fuzheng Injection+OX | Yangzheng Xiaoji Capsules+OX | 0 | . | . | . |  |  |  |  |  | 2.86 [-0.85; 6.57] | Very low | High | Not serious | Very low | 2.86 [-0.85; 6.57] | Very low | . | Serious | Very low | NMA estimate was used because incoherence was not available. Imprecision was rated as Serious. |

1. **1-year Overall survival**

| **Arm_1** | **Arm_2** | **No_of_study** | **Sample_size** | **I2** | **Direct_estimate** | **ROB** | **Inconsistency** | **Indirectness** | **Publication_bias** | **Direct_rating_without_imprecision** | **Indirect_estimate** | **Certainty_of_evidence_for_arm1** | **Certainty_of_evidence_for_arm2** | **Intransitivity** | **Indirect_rating_without_imprecision** | **Network_meta_analysis** | **Higher_rating_of_direct_and_indirect_without_imprecision** | **Incoherence** | **NMA_Imprecision** | **Final_network_rating** | **Final_rating_reason** |
| --- | --- | --- | --- | --- | --- | --- | --- | --- | --- | --- | --- | --- | --- | --- | --- | --- | --- | --- | --- | --- | --- |
| Aidi Injection+OX | OX | 3 | 169 | 19.0% | 1.27 [0.98; 1.65] | Very serious | Not serious | Not serious | Undetected | Low | . |  |  |  |  | 1.27 [0.98; 1.65] | Low | . | Serious | Very low | NMA estimate was used because incoherence was not available. Imprecision was rated as Serious. |
| Aidi Injection+OX | Shenqi Fuzheng Injection+OX | 0 | . | . | . |  |  |  |  |  | 0.93 [0.45; 1.91] | Low | Moderate | Not serious | Low | 0.93 [0.45; 1.91] | Low | . | Serious | Very low | NMA estimate was used because incoherence was not available. Imprecision was rated as Serious. |
| Aidi Injection+OX | Xiaoaiping Injection+OX | 0 | . | . | . |  |  |  |  |  | 1.09 [0.40; 2.94] | Low | Low | Not serious | Low | 1.09 [0.40; 2.94] | Low | . | Serious | Very low | NMA estimate was used because incoherence was not available. Imprecision was rated as Serious. |
| Aidi Injection+OX | Xihuang Capsules+OX | 0 | . | . | . |  |  |  |  |  | 1.18 [0.84; 1.66] | Low | Low | Not serious | Low | 1.18 [0.84; 1.66] | Low | . | Serious | Very low | NMA estimate was used because incoherence was not available. Imprecision was rated as Serious. |
| Aidi Injection+OX | Ya Dan Zi Oil Emulsion Injection+OX | 0 | . | . | . |  |  |  |  |  | 1.01 [0.65; 1.56] | Low | Low | Not serious | Low | 1.01 [0.65; 1.56] | Low | . | Serious | Very low | NMA estimate was used because incoherence was not available. Imprecision was rated as Serious. |
| Shenqi Fuzheng Injection+OX | OX | 2 | 73 | 0.0% | 1.37 [0.70; 2.68] | Serious | Not serious | Not serious | Undetected | Moderate | . |  |  |  |  | 1.37 [0.70; 2.68] | Moderate | . | Serious | Low | NMA estimate was used because incoherence was not available. Imprecision was rated as Serious. |
| Xiaoaiping Injection+OX | OX | 1 | 56 | . | 1.17 [0.45; 3.04] | Very serious | Not serious | Not serious | Undetected | Low | . |  |  |  |  | 1.17 [0.45; 3.04] | Low | . | Serious | Very low | NMA estimate was used because incoherence was not available. Imprecision was rated as Serious. |
| Xihuang Capsules+OX | OX | 2 | 164 | 0.0% | 1.08 [0.86; 1.35] | Very serious | Not serious | Not serious | Undetected | Low | . |  |  |  |  | 1.08 [0.86; 1.35] | Low | . | Serious | Very low | NMA estimate was used because incoherence was not available. Imprecision was rated as Serious. |
| Ya Dan Zi Oil Emulsion Injection+OX | OX | 1 | 64 | . | 1.26 [0.89; 1.79] | Very serious | Not serious | Not serious | Undetected | Low | . |  |  |  |  | 1.26 [0.89; 1.79] | Low | . | Serious | Very low | NMA estimate was used because incoherence was not available. Imprecision was rated as Serious. |
| Shenqi Fuzheng Injection+OX | Xiaoaiping Injection+OX | 0 | . | . | . |  |  |  |  |  | 1.17 [0.36; 3.78] | Moderate | Low | Not serious | Low | 1.17 [0.36; 3.78] | Low | . | Serious | Very low | NMA estimate was used because incoherence was not available. Imprecision was rated as Serious. |
| Shenqi Fuzheng Injection+OX | Xihuang Capsules+OX | 0 | . | . | . |  |  |  |  |  | 1.27 [0.62; 2.58] | Moderate | Low | Not serious | Low | 1.27 [0.62; 2.58] | Low | . | Serious | Very low | NMA estimate was used because incoherence was not available. Imprecision was rated as Serious. |
| Shenqi Fuzheng Injection+OX | Ya Dan Zi Oil Emulsion Injection+OX | 0 | . | . | . |  |  |  |  |  | 1.08 [0.51; 2.31] | Moderate | Low | Not serious | Low | 1.08 [0.51; 2.31] | Low | . | Serious | Very low | NMA estimate was used because incoherence was not available. Imprecision was rated as Serious. |
| Xiaoaiping Injection+OX | Xihuang Capsules+OX | 0 | . | . | . |  |  |  |  |  | 1.08 [0.40; 2.89] | Low | Low | Not serious | Low | 1.08 [0.40; 2.89] | Low | . | Serious | Very low | NMA estimate was used because incoherence was not available. Imprecision was rated as Serious. |
| Xiaoaiping Injection+OX | Ya Dan Zi Oil Emulsion Injection+OX | 0 | . | . | . |  |  |  |  |  | 0.92 [0.33; 2.56] | Low | Low | Not serious | Low | 0.92 [0.33; 2.56] | Low | . | Serious | Very low | NMA estimate was used because incoherence was not available. Imprecision was rated as Serious. |
| Xihuang Capsules+OX | Ya Dan Zi Oil Emulsion Injection+OX | 0 | . | . | . |  |  |  |  |  | 0.85 [0.56; 1.30] | Low | Low | Not serious | Low | 0.85 [0.56; 1.30] | Low | . | Serious | Very low | NMA estimate was used because incoherence was not available. Imprecision was rated as Serious. |

1. **2-year Overall survival**

| **Arm_1** | **Arm_2** | **No_of_study** | **Sample_size** | **I2** | **Direct_estimate** | **ROB** | **Inconsistency** | **Indirectness** | **Publication_bias** | **Direct_rating_without_imprecision** | **Indirect_estimate** | **Certainty_of_evidence_for_arm1** | **Certainty_of_evidence_for_arm2** | **Intransitivity** | **Indirect_rating_without_imprecision** | **Network_meta_analysis** | **Higher_rating_of_direct_and_indirect_without_imprecision** | **Incoherence** | **NMA_Imprecision** | **Final_network_rating** | **Final_rating_reason** |
| --- | --- | --- | --- | --- | --- | --- | --- | --- | --- | --- | --- | --- | --- | --- | --- | --- | --- | --- | --- | --- | --- |
| Shenqi Fuzheng Injection+OX | OX | 2 | 229 | 0.0% | 1.49 [0.99; 2.24] | Not serious | Not serious | Not serious | Undetected | High | . |  |  |  |  | 1.49 [0.99; 2.24] | High | . | Serious | Moderate | NMA estimate was used because incoherence was not available. Imprecision was rated as Serious. |
| Xihuang Capsules+OX | OX | 2 | 164 | 0.0% | 2.20 [1.30; 3.73] | Very serious | Not serious | Not serious | Undetected | Low | . |  |  |  |  | 2.20 [1.30; 3.73] | Low | . | Serious | Very low | NMA estimate was used because incoherence was not available. Imprecision was rated as Serious. |
| Ya Dan Zi Oil Emulsion Injection+OX | OX | 1 | 64 | . | 1.40 [0.50; 3.95] | Very serious | Not serious | Not serious | Undetected | Low | . |  |  |  |  | 1.40 [0.50; 3.95] | Low | . | Serious | Very low | NMA estimate was used because incoherence was not available. Imprecision was rated as Serious. |
| Shenqi Fuzheng Injection+OX | Xihuang Capsules+OX | 0 | . | . | . |  |  |  |  |  | 0.68 [0.35; 1.32] | High | Low | Not serious | Low | 0.68 [0.35; 1.32] | Low | . | Serious | Very low | NMA estimate was used because incoherence was not available. Imprecision was rated as Serious. |
| Shenqi Fuzheng Injection+OX | Ya Dan Zi Oil Emulsion Injection+OX | 0 | . | . | . |  |  |  |  |  | 1.06 [0.35; 3.24] | High | Low | Not serious | Low | 1.06 [0.35; 3.24] | Low | . | Serious | Very low | NMA estimate was used because incoherence was not available. Imprecision was rated as Serious. |
| Xihuang Capsules+OX | Ya Dan Zi Oil Emulsion Injection+OX | 0 | . | . | . |  |  |  |  |  | 1.57 [0.49; 5.03] | Low | Low | Not serious | Low | 1.57 [0.49; 5.03] | Low | . | Serious | Very low | NMA estimate was used because incoherence was not available. Imprecision was rated as Serious. |

1. **CD3+**

| **Arm_1** | **Arm_2** | **No_of_study** | **Sample_size** | **I2** | **Direct_estimate** | **ROB** | **Inconsistency** | **Indirectness** | **Publication_bias** | **Direct_rating_without_imprecision** | **Indirect_estimate** | **Certainty_of_evidence_for_arm1** | **Certainty_of_evidence_for_arm2** | **Intransitivity** | **Indirect_rating_without_imprecision** | **Network_meta_analysis** | **Higher_rating_of_direct_and_indirect_without_imprecision** | **Incoherence** | **NMA_Imprecision** | **Final_network_rating** | **Final_rating_reason** |
| --- | --- | --- | --- | --- | --- | --- | --- | --- | --- | --- | --- | --- | --- | --- | --- | --- | --- | --- | --- | --- | --- |
| Aidi Injection+OX | Astragalus Polysaccharides+OX | 0 | . | . | . |  |  |  |  |  | 2.08 [ -5.42; 9.57] | Very low | Low | Not serious | Very low | 2.08 [ -5.42; 9.57] | Very low | . | Serious | Very low | NMA estimate was used because incoherence was not available. Imprecision was rated as Serious. |
| Aidi Injection+OX | Astragalus preparations+OX | 0 | . | . | . |  |  |  |  |  | 3.58 [ -5.92; 13.09] | Very low | Low | Not serious | Very low | 3.58 [ -5.92; 13.09] | Very low | . | Serious | Very low | NMA estimate was used because incoherence was not available. Imprecision was rated as Serious. |
| Aidi Injection+OX | Compound Kushen Injection+OX | 0 | . | . | . |  |  |  |  |  | 1.74 [ -3.30; 6.78] | Very low | Very low | Not serious | Very low | 1.74 [ -3.30; 6.78] | Very low | . | Serious | Very low | NMA estimate was used because incoherence was not available. Imprecision was rated as Serious. |
| Aidi Injection+OX | Compound Mylabris preparations+OX | 0 | . | . | . |  |  |  |  |  | 5.79 [ -0.36; 11.94] | Very low | Low | Not serious | Very low | 5.79 [ -0.36; 11.94] | Very low | . | Serious | Very low | NMA estimate was used because incoherence was not available. Imprecision was rated as Serious. |
| Aidi Injection+OX | Ginseng Polysaccharide Injection+OX | 0 | . | . | . |  |  |  |  |  | 0.53 [ -9.23; 10.30] | Very low | High | Not serious | Very low | 0.53 [ -9.23; 10.30] | Very low | . | Serious | Very low | NMA estimate was used because incoherence was not available. Imprecision was rated as Serious. |
| Aidi Injection+OX | Huachansu preparations+OX | 0 | . | . | . |  |  |  |  |  | 3.66 [ -2.98; 10.30] | Very low | High | Not serious | Very low | 3.66 [ -2.98; 10.30] | Very low | . | Serious | Very low | NMA estimate was used because incoherence was not available. Imprecision was rated as Serious. |
| Aidi Injection+OX | Jinlong Capsules+OX | 0 | . | . | . |  |  |  |  |  | 2.81 [ -3.97; 9.58] | Very low | Very low | Not serious | Very low | 2.81 [ -3.97; 9.58] | Very low | . | Serious | Very low | NMA estimate was used because incoherence was not available. Imprecision was rated as Serious. |
| Aidi Injection+OX | Kangai Injection+OX | 0 | . | . | . |  |  |  |  |  | -0.57 [ -7.15; 6.02] | Very low | Moderate | Not serious | Very low | -0.57 [ -7.15; 6.02] | Very low | . | Serious | Very low | NMA estimate was used because incoherence was not available. Imprecision was rated as Serious. |
| Aidi Injection+OX | Kanglixin Capsules+OX | 0 | . | . | . |  |  |  |  |  | 1.78 [ -5.68; 9.24] | Very low | Low | Not serious | Very low | 1.78 [ -5.68; 9.24] | Very low | . | Serious | Very low | NMA estimate was used because incoherence was not available. Imprecision was rated as Serious. |
| Aidi Injection+OX | Lentinan+OX | 0 | . | . | . |  |  |  |  |  | 2.91 [ -4.58; 10.39] | Very low | Low | Not serious | Very low | 2.91 [ -4.58; 10.39] | Very low | . | Serious | Very low | NMA estimate was used because incoherence was not available. Imprecision was rated as Serious. |
| Aidi Injection+OX | OX | 4 | 402 | 96.6% | 11.08 [ 6.65; 15.52] | Very serious | Very serious | Not serious | Undetected | Very low | . |  |  |  |  | 11.08 [ 6.65; 15.52] | Very low | . | Not serious | Very low | NMA estimate was used because incoherence was not available. Imprecision was rated as Serious. |
| Aidi Injection+OX | Pingxiao Capsules+OX | 0 | . | . | . |  |  |  |  |  | -1.39 [-11.12; 8.35] | Very low | Low | Not serious | Very low | -1.39 [-11.12; 8.35] | Very low | . | Serious | Very low | NMA estimate was used because incoherence was not available. Imprecision was rated as Serious. |
| Aidi Injection+OX | Shenmai Injection+OX | 0 | . | . | . |  |  |  |  |  | 1.05 [ -8.42; 10.53] | Very low | High | Not serious | Very low | 1.05 [ -8.42; 10.53] | Very low | . | Serious | Very low | NMA estimate was used because incoherence was not available. Imprecision was rated as Serious. |
| Aidi Injection+OX | Shenqi Fuzheng Injection+OX | 0 | . | . | . |  |  |  |  |  | 3.34 [ -1.95; 8.63] | Very low | Very low | Not serious | Very low | 3.34 [ -1.95; 8.63] | Very low | . | Serious | Very low | NMA estimate was used because incoherence was not available. Imprecision was rated as Serious. |
| Aidi Injection+OX | Shenqi Fuzheng Injection+SOX | 0 | . | . | . |  |  |  |  |  | 2.48 [ -7.47; 12.44] | Very low | Low | Not serious | Very low | 2.48 [ -7.47; 12.44] | Very low | . | Serious | Very low | NMA estimate was used because incoherence was not available. Imprecision was rated as Serious. |
| Aidi Injection+OX | Xiaoaiping Injection+OX | 0 | . | . | . |  |  |  |  |  | -2.77 [-10.47; 4.92] | Very low | Very low | Not serious | Very low | -2.77 [-10.47; 4.92] | Very low | . | Serious | Very low | NMA estimate was used because incoherence was not available. Imprecision was rated as Serious. |
| Aidi Injection+OX | Xihuang Capsules+OX | 0 | . | . | . |  |  |  |  |  | 0.04 [ -7.49; 7.58] | Very low | Low | Not serious | Very low | 0.04 [ -7.49; 7.58] | Very low | . | Serious | Very low | NMA estimate was used because incoherence was not available. Imprecision was rated as Serious. |
| Aidi Injection+OX | Ya Dan Zi Oil Emulsion Injection+OX | 0 | . | . | . |  |  |  |  |  | 3.15 [ -4.54; 10.85] | Very low | Very low | Not serious | Very low | 3.15 [ -4.54; 10.85] | Very low | . | Serious | Very low | NMA estimate was used because incoherence was not available. Imprecision was rated as Serious. |
| Aidi Injection+OX | Yangzheng Xiaoji Capsules+OX | 0 | . | . | . |  |  |  |  |  | 1.76 [ -7.84; 11.37] | Very low | High | Not serious | Very low | 1.76 [ -7.84; 11.37] | Very low | . | Serious | Very low | NMA estimate was used because incoherence was not available. Imprecision was rated as Serious. |
| Aidi Injection+OX | Zhenqi Fuzheng Granules+OX | 0 | . | . | . |  |  |  |  |  | -0.02 [ -9.62; 9.59] | Very low | Low | Not serious | Very low | -0.02 [ -9.62; 9.59] | Very low | . | Serious | Very low | NMA estimate was used because incoherence was not available. Imprecision was rated as Serious. |
| Astragalus Polysaccharides+OX | Astragalus preparations+OX | 0 | . | . | . |  |  |  |  |  | 1.51 [ -8.84; 11.86] | Low | Low | Not serious | Low | 1.51 [ -8.84; 11.86] | Low | . | Serious | Very low | NMA estimate was used because incoherence was not available. Imprecision was rated as Serious. |
| Astragalus Polysaccharides+OX | Compound Kushen Injection+OX | 0 | . | . | . |  |  |  |  |  | -0.34 [ -6.83; 6.16] | Low | Very low | Not serious | Very low | -0.34 [ -6.83; 6.16] | Very low | . | Serious | Very low | NMA estimate was used because incoherence was not available. Imprecision was rated as Serious. |
| Astragalus Polysaccharides+OX | Compound Mylabris preparations+OX | 0 | . | . | . |  |  |  |  |  | 3.71 [ -3.68; 11.10] | Low | Low | Not serious | Low | 3.71 [ -3.68; 11.10] | Low | . | Serious | Very low | NMA estimate was used because incoherence was not available. Imprecision was rated as Serious. |
| Astragalus Polysaccharides+OX | Ginseng Polysaccharide Injection+OX | 0 | . | . | . |  |  |  |  |  | -1.54 [-12.13; 9.05] | Low | High | Not serious | Low | -1.54 [-12.13; 9.05] | Low | . | Serious | Very low | NMA estimate was used because incoherence was not available. Imprecision was rated as Serious. |
| Astragalus Polysaccharides+OX | Huachansu preparations+OX | 0 | . | . | . |  |  |  |  |  | 1.58 [ -6.22; 9.39] | Low | High | Not serious | Low | 1.58 [ -6.22; 9.39] | Low | . | Serious | Very low | NMA estimate was used because incoherence was not available. Imprecision was rated as Serious. |
| Astragalus Polysaccharides+OX | Jinlong Capsules+OX | 0 | . | . | . |  |  |  |  |  | 0.73 [ -7.19; 8.65] | Low | Very low | Not serious | Very low | 0.73 [ -7.19; 8.65] | Very low | . | Serious | Very low | NMA estimate was used because incoherence was not available. Imprecision was rated as Serious. |
| Astragalus Polysaccharides+OX | Kangai Injection+OX | 0 | . | . | . |  |  |  |  |  | -2.64 [-10.40; 5.11] | Low | Moderate | Not serious | Low | -2.64 [-10.40; 5.11] | Low | . | Serious | Very low | NMA estimate was used because incoherence was not available. Imprecision was rated as Serious. |
| Astragalus Polysaccharides+OX | Kanglixin Capsules+OX | 0 | . | . | . |  |  |  |  |  | -0.30 [ -8.81; 8.22] | Low | Low | Not serious | Low | -0.30 [ -8.81; 8.22] | Low | . | Serious | Very low | NMA estimate was used because incoherence was not available. Imprecision was rated as Serious. |
| Astragalus Polysaccharides+OX | Lentinan+OX | 0 | . | . | . |  |  |  |  |  | 0.83 [ -7.71; 9.37] | Low | Low | Not serious | Low | 0.83 [ -7.71; 9.37] | Low | . | Serious | Very low | NMA estimate was used because incoherence was not available. Imprecision was rated as Serious. |
| Astragalus Polysaccharides+OX | OX | 2 | 151 | 72.3% | 9.01 [ 2.96; 15.05] | Very serious | Not serious | Not serious | Undetected | Low | . |  |  |  |  | 9.01 [ 2.96; 15.05] | Low | . | Not serious | Low | NMA estimate was used because incoherence was not available. Imprecision was rated as Serious. |
| Astragalus Polysaccharides+OX | Pingxiao Capsules+OX | 0 | . | . | . |  |  |  |  |  | -3.46 [-14.03; 7.10] | Low | Low | Not serious | Low | -3.46 [-14.03; 7.10] | Low | . | Serious | Very low | NMA estimate was used because incoherence was not available. Imprecision was rated as Serious. |
| Astragalus Polysaccharides+OX | Shenmai Injection+OX | 0 | . | . | . |  |  |  |  |  | -1.02 [-11.35; 9.31] | Low | High | Not serious | Low | -1.02 [-11.35; 9.31] | Low | . | Serious | Very low | NMA estimate was used because incoherence was not available. Imprecision was rated as Serious. |
| Astragalus Polysaccharides+OX | Shenqi Fuzheng Injection+OX | 0 | . | . | . |  |  |  |  |  | 1.26 [ -5.43; 7.96] | Low | Very low | Not serious | Very low | 1.26 [ -5.43; 7.96] | Very low | . | Serious | Very low | NMA estimate was used because incoherence was not available. Imprecision was rated as Serious. |
| Astragalus Polysaccharides+OX | Shenqi Fuzheng Injection+SOX | 0 | . | . | . |  |  |  |  |  | 0.41 [-10.36; 11.18] | Low | Low | Not serious | Low | 0.41 [-10.36; 11.18] | Low | . | Serious | Very low | NMA estimate was used because incoherence was not available. Imprecision was rated as Serious. |
| Astragalus Polysaccharides+OX | Xiaoaiping Injection+OX | 0 | . | . | . |  |  |  |  |  | -4.85 [-13.57; 3.87] | Low | Very low | Not serious | Very low | -4.85 [-13.57; 3.87] | Very low | . | Serious | Very low | NMA estimate was used because incoherence was not available. Imprecision was rated as Serious. |
| Astragalus Polysaccharides+OX | Xihuang Capsules+OX | 0 | . | . | . |  |  |  |  |  | -2.03 [-10.61; 6.55] | Low | Low | Not serious | Low | -2.03 [-10.61; 6.55] | Low | . | Serious | Very low | NMA estimate was used because incoherence was not available. Imprecision was rated as Serious. |
| Astragalus Polysaccharides+OX | Ya Dan Zi Oil Emulsion Injection+OX | 0 | . | . | . |  |  |  |  |  | 1.08 [ -7.64; 9.79] | Low | Very low | Not serious | Very low | 1.08 [ -7.64; 9.79] | Very low | . | Serious | Very low | NMA estimate was used because incoherence was not available. Imprecision was rated as Serious. |
| Astragalus Polysaccharides+OX | Yangzheng Xiaoji Capsules+OX | 0 | . | . | . |  |  |  |  |  | -0.31 [-10.76; 10.13] | Low | High | Not serious | Low | -0.31 [-10.76; 10.13] | Low | . | Serious | Very low | NMA estimate was used because incoherence was not available. Imprecision was rated as Serious. |
| Astragalus Polysaccharides+OX | Zhenqi Fuzheng Granules+OX | 0 | . | . | . |  |  |  |  |  | -2.09 [-12.54; 8.35] | Low | Low | Not serious | Low | -2.09 [-12.54; 8.35] | Low | . | Serious | Very low | NMA estimate was used because incoherence was not available. Imprecision was rated as Serious. |
| Astragalus preparations+OX | Compound Kushen Injection+OX | 0 | . | . | . |  |  |  |  |  | -1.84 [-10.58; 6.89] | Low | Very low | Not serious | Very low | -1.84 [-10.58; 6.89] | Very low | . | Serious | Very low | NMA estimate was used because incoherence was not available. Imprecision was rated as Serious. |
| Astragalus preparations+OX | Compound Mylabris preparations+OX | 0 | . | . | . |  |  |  |  |  | 2.21 [ -7.21; 11.63] | Low | Low | Not serious | Low | 2.21 [ -7.21; 11.63] | Low | . | Serious | Very low | NMA estimate was used because incoherence was not available. Imprecision was rated as Serious. |
| Astragalus preparations+OX | Ginseng Polysaccharide Injection+OX | 0 | . | . | . |  |  |  |  |  | -3.05 [-15.15; 9.05] | Low | High | Not serious | Low | -3.05 [-15.15; 9.05] | Low | . | Serious | Very low | NMA estimate was used because incoherence was not available. Imprecision was rated as Serious. |
| Astragalus preparations+OX | Huachansu preparations+OX | 0 | . | . | . |  |  |  |  |  | 0.08 [ -9.67; 9.82] | Low | High | Not serious | Low | 0.08 [ -9.67; 9.82] | Low | . | Serious | Very low | NMA estimate was used because incoherence was not available. Imprecision was rated as Serious. |
| Astragalus preparations+OX | Jinlong Capsules+OX | 0 | . | . | . |  |  |  |  |  | -0.78 [-10.62; 9.06] | Low | Very low | Not serious | Very low | -0.78 [-10.62; 9.06] | Very low | . | Serious | Very low | NMA estimate was used because incoherence was not available. Imprecision was rated as Serious. |
| Astragalus preparations+OX | Kangai Injection+OX | 0 | . | . | . |  |  |  |  |  | -4.15 [-13.86; 5.56] | Low | Moderate | Not serious | Low | -4.15 [-13.86; 5.56] | Low | . | Serious | Very low | NMA estimate was used because incoherence was not available. Imprecision was rated as Serious. |
| Astragalus preparations+OX | Kanglixin Capsules+OX | 0 | . | . | . |  |  |  |  |  | -1.80 [-12.13; 8.52] | Low | Low | Not serious | Low | -1.80 [-12.13; 8.52] | Low | . | Serious | Very low | NMA estimate was used because incoherence was not available. Imprecision was rated as Serious. |
| Astragalus preparations+OX | Lentinan+OX | 0 | . | . | . |  |  |  |  |  | -0.68 [-11.02; 9.67] | Low | Low | Not serious | Low | -0.68 [-11.02; 9.67] | Low | . | Serious | Very low | NMA estimate was used because incoherence was not available. Imprecision was rated as Serious. |
| Astragalus preparations+OX | OX | 1 | 67 | . | 7.50 [-0.91; 15.91] | Very serious | Not serious | Not serious | Undetected | Low | . |  |  |  |  | 7.50 [ -0.91; 15.91] | Low | . | Serious | Very low | NMA estimate was used because incoherence was not available. Imprecision was rated as Serious. |
| Astragalus preparations+OX | Pingxiao Capsules+OX | 0 | . | . | . |  |  |  |  |  | -4.97 [-17.04; 7.10] | Low | Low | Not serious | Low | -4.97 [-17.04; 7.10] | Low | . | Serious | Very low | NMA estimate was used because incoherence was not available. Imprecision was rated as Serious. |
| Astragalus preparations+OX | Shenmai Injection+OX | 0 | . | . | . |  |  |  |  |  | -2.53 [-14.40; 9.34] | Low | High | Not serious | Low | -2.53 [-14.40; 9.34] | Low | . | Serious | Very low | NMA estimate was used because incoherence was not available. Imprecision was rated as Serious. |
| Astragalus preparations+OX | Shenqi Fuzheng Injection+OX | 0 | . | . | . |  |  |  |  |  | -0.24 [ -9.13; 8.64] | Low | Very low | Not serious | Very low | -0.24 [ -9.13; 8.64] | Very low | . | Serious | Very low | NMA estimate was used because incoherence was not available. Imprecision was rated as Serious. |
| Astragalus preparations+OX | Shenqi Fuzheng Injection+SOX | 0 | . | . | . |  |  |  |  |  | -1.10 [-13.35; 11.15] | Low | Low | Not serious | Low | -1.10 [-13.35; 11.15] | Low | . | Serious | Very low | NMA estimate was used because incoherence was not available. Imprecision was rated as Serious. |
| Astragalus preparations+OX | Xiaoaiping Injection+OX | 0 | . | . | . |  |  |  |  |  | -6.36 [-16.85; 4.14] | Low | Very low | Not serious | Very low | -6.36 [-16.85; 4.14] | Very low | . | Serious | Very low | NMA estimate was used because incoherence was not available. Imprecision was rated as Serious. |
| Astragalus preparations+OX | Xihuang Capsules+OX | 0 | . | . | . |  |  |  |  |  | -3.54 [-13.92; 6.84] | Low | Low | Not serious | Low | -3.54 [-13.92; 6.84] | Low | . | Serious | Very low | NMA estimate was used because incoherence was not available. Imprecision was rated as Serious. |
| Astragalus preparations+OX | Ya Dan Zi Oil Emulsion Injection+OX | 0 | . | . | . |  |  |  |  |  | -0.43 [-10.93; 10.06] | Low | Very low | Not serious | Very low | -0.43 [-10.93; 10.06] | Very low | . | Serious | Very low | NMA estimate was used because incoherence was not available. Imprecision was rated as Serious. |
| Astragalus preparations+OX | Yangzheng Xiaoji Capsules+OX | 0 | . | . | . |  |  |  |  |  | -1.82 [-13.79; 10.15] | Low | High | Not serious | Low | -1.82 [-13.79; 10.15] | Low | . | Serious | Very low | NMA estimate was used because incoherence was not available. Imprecision was rated as Serious. |
| Astragalus preparations+OX | Zhenqi Fuzheng Granules+OX | 0 | . | . | . |  |  |  |  |  | -3.60 [-15.57; 8.37] | Low | Low | Not serious | Low | -3.60 [-15.57; 8.37] | Low | . | Serious | Very low | NMA estimate was used because incoherence was not available. Imprecision was rated as Serious. |
| Compound Kushen Injection+OX | Compound Mylabris preparations+OX | 0 | . | . | . |  |  |  |  |  | 4.05 [ -0.82; 8.92] | Very low | Low | Not serious | Very low | 4.05 [ -0.82; 8.92] | Very low | . | Serious | Very low | NMA estimate was used because incoherence was not available. Imprecision was rated as Serious. |
| Compound Kushen Injection+OX | Ginseng Polysaccharide Injection+OX | 0 | . | . | . |  |  |  |  |  | -1.21 [-10.23; 7.81] | Very low | High | Not serious | Very low | -1.21 [-10.23; 7.81] | Very low | . | Serious | Very low | NMA estimate was used because incoherence was not available. Imprecision was rated as Serious. |
| Compound Kushen Injection+OX | Huachansu preparations+OX | 0 | . | . | . |  |  |  |  |  | 1.92 [ -3.56; 7.40] | Very low | High | Not serious | Very low | 1.92 [ -3.56; 7.40] | Very low | . | Serious | Very low | NMA estimate was used because incoherence was not available. Imprecision was rated as Serious. |
| Compound Kushen Injection+OX | Jinlong Capsules+OX | 0 | . | . | . |  |  |  |  |  | 1.07 [ -4.58; 6.71] | Very low | Very low | Not serious | Very low | 1.07 [ -4.58; 6.71] | Very low | . | Serious | Very low | NMA estimate was used because incoherence was not available. Imprecision was rated as Serious. |
| Compound Kushen Injection+OX | Kangai Injection+OX | 0 | . | . | . |  |  |  |  |  | -2.31 [ -7.72; 3.11] | Very low | Moderate | Not serious | Very low | -2.31 [ -7.72; 3.11] | Very low | . | Serious | Very low | NMA estimate was used because incoherence was not available. Imprecision was rated as Serious. |
| Compound Kushen Injection+OX | Kanglixin Capsules+OX | 0 | . | . | . |  |  |  |  |  | 0.04 [ -6.42; 6.50] | Very low | Low | Not serious | Very low | 0.04 [ -6.42; 6.50] | Very low | . | Serious | Very low | NMA estimate was used because incoherence was not available. Imprecision was rated as Serious. |
| Compound Kushen Injection+OX | Lentinan+OX | 0 | . | . | . |  |  |  |  |  | 1.16 [ -5.32; 7.65] | Very low | Low | Not serious | Very low | 1.16 [ -5.32; 7.65] | Very low | . | Serious | Very low | NMA estimate was used because incoherence was not available. Imprecision was rated as Serious. |
| Compound Kushen Injection+OX | OX | 13 | 1223 | 94.7% | 9.34 [ 6.96; 11.73] | Very serious | Very serious | Not serious | Not serious | Very low | . |  |  |  |  | 9.34 [ 6.96; 11.73] | Very low | . | Not serious | Very low | NMA estimate was used because incoherence was not available. Imprecision was rated as Not serious. |
| Compound Kushen Injection+OX | Pingxiao Capsules+OX | 0 | . | . | . |  |  |  |  |  | -3.13 [-12.11; 5.86] | Very low | Low | Not serious | Very low | -3.13 [-12.11; 5.86] | Very low | . | Serious | Very low | NMA estimate was used because incoherence was not available. Imprecision was rated as Serious. |
| Compound Kushen Injection+OX | Shenmai Injection+OX | 0 | . | . | . |  |  |  |  |  | -0.69 [ -9.39; 8.02] | Very low | High | Not serious | Very low | -0.69 [ -9.39; 8.02] | Very low | . | Serious | Very low | NMA estimate was used because incoherence was not available. Imprecision was rated as Serious. |
| Compound Kushen Injection+OX | Shenqi Fuzheng Injection+OX | 0 | . | . | . |  |  |  |  |  | 1.60 [ -2.14; 5.34] | Very low | Very low | Not serious | Very low | 1.60 [ -2.14; 5.34] | Very low | . | Serious | Very low | NMA estimate was used because incoherence was not available. Imprecision was rated as Serious. |
| Compound Kushen Injection+OX | Shenqi Fuzheng Injection+SOX | 0 | . | . | . |  |  |  |  |  | 0.74 [ -8.49; 9.97] | Very low | Low | Not serious | Very low | 0.74 [ -8.49; 9.97] | Very low | . | Serious | Very low | NMA estimate was used because incoherence was not available. Imprecision was rated as Serious. |
| Compound Kushen Injection+OX | Xiaoaiping Injection+OX | 0 | . | . | . |  |  |  |  |  | -4.51 [-11.24; 2.21] | Very low | Very low | Not serious | Very low | -4.51 [-11.24; 2.21] | Very low | . | Serious | Very low | NMA estimate was used because incoherence was not available. Imprecision was rated as Serious. |
| Compound Kushen Injection+OX | Xihuang Capsules+OX | 0 | . | . | . |  |  |  |  |  | -1.70 [ -8.23; 4.84] | Very low | Low | Not serious | Very low | -1.70 [ -8.23; 4.84] | Very low | . | Serious | Very low | NMA estimate was used because incoherence was not available. Imprecision was rated as Serious. |
| Compound Kushen Injection+OX | Ya Dan Zi Oil Emulsion Injection+OX | 0 | . | . | . |  |  |  |  |  | 1.41 [ -5.31; 8.13] | Very low | Very low | Not serious | Very low | 1.41 [ -5.31; 8.13] | Very low | . | Serious | Very low | NMA estimate was used because incoherence was not available. Imprecision was rated as Serious. |
| Compound Kushen Injection+OX | Yangzheng Xiaoji Capsules+OX | 0 | . | . | . |  |  |  |  |  | 0.02 [ -8.82; 8.87] | Very low | High | Not serious | Very low | 0.02 [ -8.82; 8.87] | Very low | . | Serious | Very low | NMA estimate was used because incoherence was not available. Imprecision was rated as Serious. |
| Compound Kushen Injection+OX | Zhenqi Fuzheng Granules+OX | 0 | . | . | . |  |  |  |  |  | -1.76 [-10.60; 7.09] | Very low | Low | Not serious | Very low | -1.76 [-10.60; 7.09] | Very low | . | Serious | Very low | NMA estimate was used because incoherence was not available. Imprecision was rated as Serious. |
| Compound Mylabris preparations+OX | Ginseng Polysaccharide Injection+OX | 0 | . | . | . |  |  |  |  |  | -5.26 [-14.94; 4.43] | Low | High | Not serious | Low | -5.26 [-14.94; 4.43] | Low | . | Serious | Very low | NMA estimate was used because incoherence was not available. Imprecision was rated as Serious. |
| Compound Mylabris preparations+OX | Huachansu preparations+OX | 0 | . | . | . |  |  |  |  |  | -2.13 [ -8.65; 4.39] | Low | High | Not serious | Low | -2.13 [ -8.65; 4.39] | Low | . | Serious | Very low | NMA estimate was used because incoherence was not available. Imprecision was rated as Serious. |
| Compound Mylabris preparations+OX | Jinlong Capsules+OX | 0 | . | . | . |  |  |  |  |  | -2.98 [ -9.64; 3.67] | Low | Very low | Not serious | Very low | -2.98 [ -9.64; 3.67] | Very low | . | Serious | Very low | NMA estimate was used because incoherence was not available. Imprecision was rated as Serious. |
| Compound Mylabris preparations+OX | Kangai Injection+OX | 0 | . | . | . |  |  |  |  |  | -6.36 [-12.82; 0.10] | Low | Moderate | Not serious | Low | -6.36 [-12.82; 0.10] | Low | . | Serious | Very low | NMA estimate was used because incoherence was not available. Imprecision was rated as Serious. |
| Compound Mylabris preparations+OX | Kanglixin Capsules+OX | 0 | . | . | . |  |  |  |  |  | -4.01 [-11.37; 3.35] | Low | Low | Not serious | Low | -4.01 [-11.37; 3.35] | Low | . | Serious | Very low | NMA estimate was used because incoherence was not available. Imprecision was rated as Serious. |
| Compound Mylabris preparations+OX | Lentinan+OX | 0 | . | . | . |  |  |  |  |  | -2.88 [-10.26; 4.49] | Low | Low | Not serious | Low | -2.88 [-10.26; 4.49] | Low | . | Serious | Very low | NMA estimate was used because incoherence was not available. Imprecision was rated as Serious. |
| Compound Mylabris preparations+OX | OX | 4 | 333 | 80.6% | 5.29 [ 1.04; 9.55] | Very serious | Not serious | Not serious | Undetected | Low | . |  |  |  |  | 5.29 [ 1.04; 9.55] | Low | . | Not serious | Low | NMA estimate was used because incoherence was not available. Imprecision was rated as Serious. |
| Compound Mylabris preparations+OX | Pingxiao Capsules+OX | 0 | . | . | . |  |  |  |  |  | -7.18 [-16.83; 2.48] | Low | Low | Not serious | Low | -7.18 [-16.83; 2.48] | Low | . | Serious | Very low | NMA estimate was used because incoherence was not available. Imprecision was rated as Serious. |
| Compound Mylabris preparations+OX | Shenmai Injection+OX | 0 | . | . | . |  |  |  |  |  | -4.74 [-14.13; 4.66] | Low | High | Not serious | Low | -4.74 [-14.13; 4.66] | Low | . | Serious | Very low | NMA estimate was used because incoherence was not available. Imprecision was rated as Serious. |
| Compound Mylabris preparations+OX | Shenqi Fuzheng Injection+OX | 0 | . | . | . |  |  |  |  |  | -2.45 [ -7.59; 2.69] | Low | Very low | Not serious | Very low | -2.45 [ -7.59; 2.69] | Very low | . | Serious | Very low | NMA estimate was used because incoherence was not available. Imprecision was rated as Serious. |
| Compound Mylabris preparations+OX | Shenqi Fuzheng Injection+SOX | 0 | . | . | . |  |  |  |  |  | -3.31 [-13.18; 6.57] | Low | Low | Not serious | Low | -3.31 [-13.18; 6.57] | Low | . | Serious | Very low | NMA estimate was used because incoherence was not available. Imprecision was rated as Serious. |
| Compound Mylabris preparations+OX | Xiaoaiping Injection+OX | 0 | . | . | . |  |  |  |  |  | -8.56 [-16.15; -0.97] | Low | Very low | Not serious | Very low | -8.56 [-16.15; -0.97] | Very low | . | Serious | Very low | NMA estimate was used because incoherence was not available. Imprecision was rated as Serious. |
| Compound Mylabris preparations+OX | Xihuang Capsules+OX | 0 | . | . | . |  |  |  |  |  | -5.75 [-13.17; 1.68] | Low | Low | Not serious | Low | -5.75 [-13.17; 1.68] | Low | . | Serious | Very low | NMA estimate was used because incoherence was not available. Imprecision was rated as Serious. |
| Compound Mylabris preparations+OX | Ya Dan Zi Oil Emulsion Injection+OX | 0 | . | . | . |  |  |  |  |  | -2.64 [-10.23; 4.95] | Low | Very low | Not serious | Very low | -2.64 [-10.23; 4.95] | Very low | . | Serious | Very low | NMA estimate was used because incoherence was not available. Imprecision was rated as Serious. |
| Compound Mylabris preparations+OX | Yangzheng Xiaoji Capsules+OX | 0 | . | . | . |  |  |  |  |  | -4.03 [-13.55; 5.50] | Low | High | Not serious | Low | -4.03 [-13.55; 5.50] | Low | . | Serious | Very low | NMA estimate was used because incoherence was not available. Imprecision was rated as Serious. |
| Compound Mylabris preparations+OX | Zhenqi Fuzheng Granules+OX | 0 | . | . | . |  |  |  |  |  | -5.81 [-15.33; 3.71] | Low | Low | Not serious | Low | -5.81 [-15.33; 3.71] | Low | . | Serious | Very low | NMA estimate was used because incoherence was not available. Imprecision was rated as Serious. |
| Ginseng Polysaccharide Injection+OX | Huachansu preparations+OX | 0 | . | . | . |  |  |  |  |  | 3.13 [ -6.88; 13.13] | High | High | Not serious | High | 3.13 [ -6.88; 13.13] | High | . | Serious | Moderate | NMA estimate was used because incoherence was not available. Imprecision was rated as Serious. |
| Ginseng Polysaccharide Injection+OX | Jinlong Capsules+OX | 0 | . | . | . |  |  |  |  |  | 2.27 [ -7.82; 12.36] | High | Very low | Not serious | Very low | 2.27 [ -7.82; 12.36] | Very low | . | Serious | Very low | NMA estimate was used because incoherence was not available. Imprecision was rated as Serious. |
| Ginseng Polysaccharide Injection+OX | Kangai Injection+OX | 0 | . | . | . |  |  |  |  |  | -1.10 [-11.07; 8.86] | High | Moderate | Not serious | Moderate | -1.10 [-11.07; 8.86] | Moderate | . | Serious | Low | NMA estimate was used because incoherence was not available. Imprecision was rated as Serious. |
| Ginseng Polysaccharide Injection+OX | Kanglixin Capsules+OX | 0 | . | . | . |  |  |  |  |  | 1.25 [ -9.32; 11.81] | High | Low | Not serious | Low | 1.25 [ -9.32; 11.81] | Low | . | Serious | Very low | NMA estimate was used because incoherence was not available. Imprecision was rated as Serious. |
| Ginseng Polysaccharide Injection+OX | Lentinan+OX | 0 | . | . | . |  |  |  |  |  | 2.37 [ -8.21; 12.96] | High | Low | Not serious | Low | 2.37 [ -8.21; 12.96] | Low | . | Serious | Very low | NMA estimate was used because incoherence was not available. Imprecision was rated as Serious. |
| Ginseng Polysaccharide Injection+OX | OX | 1 | 68 | . | 10.55 [ 1.85; 19.25] | Not serious | Not serious | Not serious | Undetected | High | . |  |  |  |  | 10.55 [ 1.85; 19.25] | High | . | Not serious | High | NMA estimate was used because incoherence was not available. Imprecision was rated as Serious. |
| Ginseng Polysaccharide Injection+OX | Pingxiao Capsules+OX | 0 | . | . | . |  |  |  |  |  | -1.92 [-14.20; 10.36] | High | Low | Not serious | Low | -1.92 [-14.20; 10.36] | Low | . | Serious | Very low | NMA estimate was used because incoherence was not available. Imprecision was rated as Serious. |
| Ginseng Polysaccharide Injection+OX | Shenmai Injection+OX | 0 | . | . | . |  |  |  |  |  | 0.52 [-11.56; 12.60] | High | High | Not serious | High | 0.52 [-11.56; 12.60] | High | . | Serious | Moderate | NMA estimate was used because incoherence was not available. Imprecision was rated as Serious. |
| Ginseng Polysaccharide Injection+OX | Shenqi Fuzheng Injection+OX | 0 | . | . | . |  |  |  |  |  | 2.81 [ -6.36; 11.97] | High | Very low | Not serious | Very low | 2.81 [ -6.36; 11.97] | Very low | . | Serious | Very low | NMA estimate was used because incoherence was not available. Imprecision was rated as Serious. |
| Ginseng Polysaccharide Injection+OX | Shenqi Fuzheng Injection+SOX | 0 | . | . | . |  |  |  |  |  | 1.95 [-10.51; 14.41] | High | Low | Not serious | Low | 1.95 [-10.51; 14.41] | Low | . | Serious | Very low | NMA estimate was used because incoherence was not available. Imprecision was rated as Serious. |
| Ginseng Polysaccharide Injection+OX | Xiaoaiping Injection+OX | 0 | . | . | . |  |  |  |  |  | -3.31 [-14.04; 7.43] | High | Very low | Not serious | Very low | -3.31 [-14.04; 7.43] | Very low | . | Serious | Very low | NMA estimate was used because incoherence was not available. Imprecision was rated as Serious. |
| Ginseng Polysaccharide Injection+OX | Xihuang Capsules+OX | 0 | . | . | . |  |  |  |  |  | -0.49 [-11.11; 10.13] | High | Low | Not serious | Low | -0.49 [-11.11; 10.13] | Low | . | Serious | Very low | NMA estimate was used because incoherence was not available. Imprecision was rated as Serious. |
| Ginseng Polysaccharide Injection+OX | Ya Dan Zi Oil Emulsion Injection+OX | 0 | . | . | . |  |  |  |  |  | 2.62 [ -8.12; 13.35] | High | Very low | Not serious | Very low | 2.62 [ -8.12; 13.35] | Very low | . | Serious | Very low | NMA estimate was used because incoherence was not available. Imprecision was rated as Serious. |
| Ginseng Polysaccharide Injection+OX | Yangzheng Xiaoji Capsules+OX | 0 | . | . | . |  |  |  |  |  | 1.23 [-10.95; 13.41] | High | High | Not serious | High | 1.23 [-10.95; 13.41] | High | . | Serious | Moderate | NMA estimate was used because incoherence was not available. Imprecision was rated as Serious. |
| Ginseng Polysaccharide Injection+OX | Zhenqi Fuzheng Granules+OX | 0 | . | . | . |  |  |  |  |  | -0.55 [-12.72; 11.62] | High | Low | Not serious | Low | -0.55 [-12.72; 11.62] | Low | . | Serious | Very low | NMA estimate was used because incoherence was not available. Imprecision was rated as Serious. |
| Huachansu preparations+OX | Jinlong Capsules+OX | 0 | . | . | . |  |  |  |  |  | -0.85 [ -7.96; 6.25] | High | Very low | Not serious | Very low | -0.85 [ -7.96; 6.25] | Very low | . | Serious | Very low | NMA estimate was used because incoherence was not available. Imprecision was rated as Serious. |
| Huachansu preparations+OX | Kangai Injection+OX | 0 | . | . | . |  |  |  |  |  | -4.23 [-11.16; 2.70] | High | Moderate | Not serious | Moderate | -4.23 [-11.16; 2.70] | Moderate | . | Serious | Low | NMA estimate was used because incoherence was not available. Imprecision was rated as Serious. |
| Huachansu preparations+OX | Kanglixin Capsules+OX | 0 | . | . | . |  |  |  |  |  | -1.88 [ -9.65; 5.89] | High | Low | Not serious | Low | -1.88 [ -9.65; 5.89] | Low | . | Serious | Very low | NMA estimate was used because incoherence was not available. Imprecision was rated as Serious. |
| Huachansu preparations+OX | Lentinan+OX | 0 | . | . | . |  |  |  |  |  | -0.75 [ -8.55; 7.04] | High | Low | Not serious | Low | -0.75 [ -8.55; 7.04] | Low | . | Serious | Very low | NMA estimate was used because incoherence was not available. Imprecision was rated as Serious. |
| Huachansu preparations+OX | OX | 3 | 213 | 87.6% | 7.42 [ 2.49; 12.36] | Not serious | Not serious | Not serious | Undetected | High | . |  |  |  |  | 7.42 [ 2.49; 12.36] | High | . | Not serious | High | NMA estimate was used because incoherence was not available. Imprecision was rated as Serious. |
| Huachansu preparations+OX | Pingxiao Capsules+OX | 0 | . | . | . |  |  |  |  |  | -5.05 [-15.02; 4.93] | High | Low | Not serious | Low | -5.05 [-15.02; 4.93] | Low | . | Serious | Very low | NMA estimate was used because incoherence was not available. Imprecision was rated as Serious. |
| Huachansu preparations+OX | Shenmai Injection+OX | 0 | . | . | . |  |  |  |  |  | -2.61 [-12.33; 7.12] | High | High | Not serious | High | -2.61 [-12.33; 7.12] | High | . | Serious | Moderate | NMA estimate was used because incoherence was not available. Imprecision was rated as Serious. |
| Huachansu preparations+OX | Shenqi Fuzheng Injection+OX | 0 | . | . | . |  |  |  |  |  | -0.32 [ -6.04; 5.40] | High | Very low | Not serious | Very low | -0.32 [ -6.04; 5.40] | Very low | . | Serious | Very low | NMA estimate was used because incoherence was not available. Imprecision was rated as Serious. |
| Huachansu preparations+OX | Shenqi Fuzheng Injection+SOX | 0 | . | . | . |  |  |  |  |  | -1.18 [-11.37; 9.02] | High | Low | Not serious | Low | -1.18 [-11.37; 9.02] | Low | . | Serious | Very low | NMA estimate was used because incoherence was not available. Imprecision was rated as Serious. |
| Huachansu preparations+OX | Xiaoaiping Injection+OX | 0 | . | . | . |  |  |  |  |  | -6.43 [-14.43; 1.56] | High | Very low | Not serious | Very low | -6.43 [-14.43; 1.56] | Very low | . | Serious | Very low | NMA estimate was used because incoherence was not available. Imprecision was rated as Serious. |
| Huachansu preparations+OX | Xihuang Capsules+OX | 0 | . | . | . |  |  |  |  |  | -3.62 [-11.45; 4.22] | High | Low | Not serious | Low | -3.62 [-11.45; 4.22] | Low | . | Serious | Very low | NMA estimate was used because incoherence was not available. Imprecision was rated as Serious. |
| Huachansu preparations+OX | Ya Dan Zi Oil Emulsion Injection+OX | 0 | . | . | . |  |  |  |  |  | -0.51 [ -8.50; 7.48] | High | Very low | Not serious | Very low | -0.51 [ -8.50; 7.48] | Very low | . | Serious | Very low | NMA estimate was used because incoherence was not available. Imprecision was rated as Serious. |
| Huachansu preparations+OX | Yangzheng Xiaoji Capsules+OX | 0 | . | . | . |  |  |  |  |  | -1.90 [-11.74; 7.95] | High | High | Not serious | High | -1.90 [-11.74; 7.95] | High | . | Serious | Moderate | NMA estimate was used because incoherence was not available. Imprecision was rated as Serious. |
| Huachansu preparations+OX | Zhenqi Fuzheng Granules+OX | 0 | . | . | . |  |  |  |  |  | -3.68 [-13.52; 6.17] | High | Low | Not serious | Low | -3.68 [-13.52; 6.17] | Low | . | Serious | Very low | NMA estimate was used because incoherence was not available. Imprecision was rated as Serious. |
| Jinlong Capsules+OX | Kangai Injection+OX | 0 | . | . | . |  |  |  |  |  | -3.37 [-10.43; 3.68] | Very low | Moderate | Not serious | Very low | -3.37 [-10.43; 3.68] | Very low | . | Serious | Very low | NMA estimate was used because incoherence was not available. Imprecision was rated as Serious. |
| Jinlong Capsules+OX | Kanglixin Capsules+OX | 0 | . | . | . |  |  |  |  |  | -1.03 [ -8.91; 6.86] | Very low | Low | Not serious | Very low | -1.03 [ -8.91; 6.86] | Very low | . | Serious | Very low | NMA estimate was used because incoherence was not available. Imprecision was rated as Serious. |
| Jinlong Capsules+OX | Lentinan+OX | 0 | . | . | . |  |  |  |  |  | 0.10 [ -7.81; 8.01] | Very low | Low | Not serious | Very low | 0.10 [ -7.81; 8.01] | Very low | . | Serious | Very low | NMA estimate was used because incoherence was not available. Imprecision was rated as Serious. |
| Jinlong Capsules+OX | OX | 3 | 207 | 95.4% | 8.28 [ 3.16; 13.39] | Serious | Very serious | Not serious | Undetected | Very low | . |  |  |  |  | 8.28 [ 3.16; 13.39] | Very low | . | Not serious | Very low | NMA estimate was used because incoherence was not available. Imprecision was rated as Serious. |
| Jinlong Capsules+OX | Pingxiao Capsules+OX | 0 | . | . | . |  |  |  |  |  | -4.19 [-14.26; 5.87] | Very low | Low | Not serious | Very low | -4.19 [-14.26; 5.87] | Very low | . | Serious | Very low | NMA estimate was used because incoherence was not available. Imprecision was rated as Serious. |
| Jinlong Capsules+OX | Shenmai Injection+OX | 0 | . | . | . |  |  |  |  |  | -1.75 [-11.57; 8.06] | Very low | High | Not serious | Very low | -1.75 [-11.57; 8.06] | Very low | . | Serious | Very low | NMA estimate was used because incoherence was not available. Imprecision was rated as Serious. |
| Jinlong Capsules+OX | Shenqi Fuzheng Injection+OX | 0 | . | . | . |  |  |  |  |  | 0.53 [ -5.34; 6.40] | Very low | Very low | Not serious | Very low | 0.53 [ -5.34; 6.40] | Very low | . | Serious | Very low | NMA estimate was used because incoherence was not available. Imprecision was rated as Serious. |
| Jinlong Capsules+OX | Shenqi Fuzheng Injection+SOX | 0 | . | . | . |  |  |  |  |  | -0.32 [-10.60; 9.96] | Very low | Low | Not serious | Very low | -0.32 [-10.60; 9.96] | Very low | . | Serious | Very low | NMA estimate was used because incoherence was not available. Imprecision was rated as Serious. |
| Jinlong Capsules+OX | Xiaoaiping Injection+OX | 0 | . | . | . |  |  |  |  |  | -5.58 [-13.68; 2.53] | Very low | Very low | Not serious | Very low | -5.58 [-13.68; 2.53] | Very low | . | Serious | Very low | NMA estimate was used because incoherence was not available. Imprecision was rated as Serious. |
| Jinlong Capsules+OX | Xihuang Capsules+OX | 0 | . | . | . |  |  |  |  |  | -2.76 [-10.71; 5.19] | Very low | Low | Not serious | Very low | -2.76 [-10.71; 5.19] | Very low | . | Serious | Very low | NMA estimate was used because incoherence was not available. Imprecision was rated as Serious. |
| Jinlong Capsules+OX | Ya Dan Zi Oil Emulsion Injection+OX | 0 | . | . | . |  |  |  |  |  | 0.35 [ -7.76; 8.45] | Very low | Very low | Not serious | Very low | 0.35 [ -7.76; 8.45] | Very low | . | Serious | Very low | NMA estimate was used because incoherence was not available. Imprecision was rated as Serious. |
| Jinlong Capsules+OX | Yangzheng Xiaoji Capsules+OX | 0 | . | . | . |  |  |  |  |  | -1.04 [-10.98; 8.90] | Very low | High | Not serious | Very low | -1.04 [-10.98; 8.90] | Very low | . | Serious | Very low | NMA estimate was used because incoherence was not available. Imprecision was rated as Serious. |
| Jinlong Capsules+OX | Zhenqi Fuzheng Granules+OX | 0 | . | . | . |  |  |  |  |  | -2.82 [-12.76; 7.11] | Very low | Low | Not serious | Very low | -2.82 [-12.76; 7.11] | Very low | . | Serious | Very low | NMA estimate was used because incoherence was not available. Imprecision was rated as Serious. |
| Kangai Injection+OX | Kanglixin Capsules+OX | 0 | . | . | . |  |  |  |  |  | 2.35 [ -5.38; 10.07] | Moderate | Low | Not serious | Low | 2.35 [ -5.38; 10.07] | Low | . | Serious | Very low | NMA estimate was used because incoherence was not available. Imprecision was rated as Serious. |
| Kangai Injection+OX | Lentinan+OX | 0 | . | . | . |  |  |  |  |  | 3.47 [ -4.27; 11.22] | Moderate | Low | Not serious | Low | 3.47 [ -4.27; 11.22] | Low | . | Serious | Very low | NMA estimate was used because incoherence was not available. Imprecision was rated as Serious. |
| Kangai Injection+OX | OX | 3 | 211 | 82.7% | 11.65 [ 6.79; 16.52] | Serious | Not serious | Not serious | Undetected | Moderate | . |  |  |  |  | 11.65 [ 6.79; 16.52] | Moderate | . | Not serious | Moderate | NMA estimate was used because incoherence was not available. Imprecision was rated as Serious. |
| Kangai Injection+OX | Pingxiao Capsules+OX | 0 | . | . | . |  |  |  |  |  | -0.82 [-10.76; 9.12] | Moderate | Low | Not serious | Low | -0.82 [-10.76; 9.12] | Low | . | Serious | Very low | NMA estimate was used because incoherence was not available. Imprecision was rated as Serious. |
| Kangai Injection+OX | Shenmai Injection+OX | 0 | . | . | . |  |  |  |  |  | 1.62 [ -8.06; 11.31] | Moderate | High | Not serious | Moderate | 1.62 [ -8.06; 11.31] | Moderate | . | Serious | Low | NMA estimate was used because incoherence was not available. Imprecision was rated as Serious. |
| Kangai Injection+OX | Shenqi Fuzheng Injection+OX | 0 | . | . | . |  |  |  |  |  | 3.91 [ -1.75; 9.56] | Moderate | Very low | Not serious | Very low | 3.91 [ -1.75; 9.56] | Very low | . | Serious | Very low | NMA estimate was used because incoherence was not available. Imprecision was rated as Serious. |
| Kangai Injection+OX | Shenqi Fuzheng Injection+SOX | 0 | . | . | . |  |  |  |  |  | 3.05 [ -7.10; 13.21] | Moderate | Low | Not serious | Low | 3.05 [ -7.10; 13.21] | Low | . | Serious | Very low | NMA estimate was used because incoherence was not available. Imprecision was rated as Serious. |
| Kangai Injection+OX | Xiaoaiping Injection+OX | 0 | . | . | . |  |  |  |  |  | -2.20 [-10.15; 5.75] | Moderate | Very low | Not serious | Very low | -2.20 [-10.15; 5.75] | Very low | . | Serious | Very low | NMA estimate was used because incoherence was not available. Imprecision was rated as Serious. |
| Kangai Injection+OX | Xihuang Capsules+OX | 0 | . | . | . |  |  |  |  |  | 0.61 [ -7.18; 8.40] | Moderate | Low | Not serious | Low | 0.61 [ -7.18; 8.40] | Low | . | Serious | Very low | NMA estimate was used because incoherence was not available. Imprecision was rated as Serious. |
| Kangai Injection+OX | Ya Dan Zi Oil Emulsion Injection+OX | 0 | . | . | . |  |  |  |  |  | 3.72 [ -4.23; 11.67] | Moderate | Very low | Not serious | Very low | 3.72 [ -4.23; 11.67] | Very low | . | Serious | Very low | NMA estimate was used because incoherence was not available. Imprecision was rated as Serious. |
| Kangai Injection+OX | Yangzheng Xiaoji Capsules+OX | 0 | . | . | . |  |  |  |  |  | 2.33 [ -7.48; 12.14] | Moderate | High | Not serious | Moderate | 2.33 [ -7.48; 12.14] | Moderate | . | Serious | Low | NMA estimate was used because incoherence was not available. Imprecision was rated as Serious. |
| Kangai Injection+OX | Zhenqi Fuzheng Granules+OX | 0 | . | . | . |  |  |  |  |  | 0.55 [ -9.26; 10.36] | Moderate | Low | Not serious | Low | 0.55 [ -9.26; 10.36] | Low | . | Serious | Very low | NMA estimate was used because incoherence was not available. Imprecision was rated as Serious. |
| Kanglixin Capsules+OX | Lentinan+OX | 0 | . | . | . |  |  |  |  |  | 1.13 [ -7.38; 9.63] | Low | Low | Not serious | Low | 1.13 [ -7.38; 9.63] | Low | . | Serious | Very low | NMA estimate was used because incoherence was not available. Imprecision was rated as Serious. |
| Kanglixin Capsules+OX | OX | 2 | 140 | 0.0% | 9.30 [ 3.30; 15.31] | Very serious | Not serious | Not serious | Undetected | Low | . |  |  |  |  | 9.30 [ 3.30; 15.31] | Low | . | Not serious | Low | NMA estimate was used because incoherence was not available. Imprecision was rated as Serious. |
| Kanglixin Capsules+OX | Pingxiao Capsules+OX | 0 | . | . | . |  |  |  |  |  | -3.17 [-13.71; 7.38] | Low | Low | Not serious | Low | -3.17 [-13.71; 7.38] | Low | . | Serious | Very low | NMA estimate was used because incoherence was not available. Imprecision was rated as Serious. |
| Kanglixin Capsules+OX | Shenmai Injection+OX | 0 | . | . | . |  |  |  |  |  | -0.73 [-11.03; 9.58] | Low | High | Not serious | Low | -0.73 [-11.03; 9.58] | Low | . | Serious | Very low | NMA estimate was used because incoherence was not available. Imprecision was rated as Serious. |
| Kanglixin Capsules+OX | Shenqi Fuzheng Injection+OX | 0 | . | . | . |  |  |  |  |  | 1.56 [ -5.10; 8.22] | Low | Very low | Not serious | Very low | 1.56 [ -5.10; 8.22] | Very low | . | Serious | Very low | NMA estimate was used because incoherence was not available. Imprecision was rated as Serious. |
| Kanglixin Capsules+OX | Shenqi Fuzheng Injection+SOX | 0 | . | . | . |  |  |  |  |  | 0.70 [-10.04; 11.45] | Low | Low | Not serious | Low | 0.70 [-10.04; 11.45] | Low | . | Serious | Very low | NMA estimate was used because incoherence was not available. Imprecision was rated as Serious. |
| Kanglixin Capsules+OX | Xiaoaiping Injection+OX | 0 | . | . | . |  |  |  |  |  | -4.55 [-13.24; 4.14] | Low | Very low | Not serious | Very low | -4.55 [-13.24; 4.14] | Very low | . | Serious | Very low | NMA estimate was used because incoherence was not available. Imprecision was rated as Serious. |
| Kanglixin Capsules+OX | Xihuang Capsules+OX | 0 | . | . | . |  |  |  |  |  | -1.74 [-10.28; 6.81] | Low | Low | Not serious | Low | -1.74 [-10.28; 6.81] | Low | . | Serious | Very low | NMA estimate was used because incoherence was not available. Imprecision was rated as Serious. |
| Kanglixin Capsules+OX | Ya Dan Zi Oil Emulsion Injection+OX | 0 | . | . | . |  |  |  |  |  | 1.37 [ -7.32; 10.06] | Low | Very low | Not serious | Very low | 1.37 [ -7.32; 10.06] | Very low | . | Serious | Very low | NMA estimate was used because incoherence was not available. Imprecision was rated as Serious. |
| Kanglixin Capsules+OX | Yangzheng Xiaoji Capsules+OX | 0 | . | . | . |  |  |  |  |  | -0.02 [-10.44; 10.41] | Low | High | Not serious | Low | -0.02 [-10.44; 10.41] | Low | . | Serious | Very low | NMA estimate was used because incoherence was not available. Imprecision was rated as Serious. |
| Kanglixin Capsules+OX | Zhenqi Fuzheng Granules+OX | 0 | . | . | . |  |  |  |  |  | -1.80 [-12.22; 8.62] | Low | Low | Not serious | Low | -1.80 [-12.22; 8.62] | Low | . | Serious | Very low | NMA estimate was used because incoherence was not available. Imprecision was rated as Serious. |
| Lentinan+OX | OX | 2 | 206 | 97.7% | 8.18 [ 2.15; 14.21] | Not serious | Very serious | Not serious | Undetected | Low | . |  |  |  |  | 8.18 [ 2.15; 14.21] | Low | . | Not serious | Low | NMA estimate was used because incoherence was not available. Imprecision was rated as Serious. |
| Lentinan+OX | Pingxiao Capsules+OX | 0 | . | . | . |  |  |  |  |  | -4.29 [-14.85; 6.27] | Low | Low | Not serious | Low | -4.29 [-14.85; 6.27] | Low | . | Serious | Very low | NMA estimate was used because incoherence was not available. Imprecision was rated as Serious. |
| Lentinan+OX | Shenmai Injection+OX | 0 | . | . | . |  |  |  |  |  | -1.85 [-12.17; 8.47] | Low | High | Not serious | Low | -1.85 [-12.17; 8.47] | Low | . | Serious | Very low | NMA estimate was used because incoherence was not available. Imprecision was rated as Serious. |
| Lentinan+OX | Shenqi Fuzheng Injection+OX | 0 | . | . | . |  |  |  |  |  | 0.43 [ -6.25; 7.12] | Low | Very low | Not serious | Very low | 0.43 [ -6.25; 7.12] | Very low | . | Serious | Very low | NMA estimate was used because incoherence was not available. Imprecision was rated as Serious. |
| Lentinan+OX | Shenqi Fuzheng Injection+SOX | 0 | . | . | . |  |  |  |  |  | -0.42 [-11.19; 10.34] | Low | Low | Not serious | Low | -0.42 [-11.19; 10.34] | Low | . | Serious | Very low | NMA estimate was used because incoherence was not available. Imprecision was rated as Serious. |
| Lentinan+OX | Xiaoaiping Injection+OX | 0 | . | . | . |  |  |  |  |  | -5.68 [-14.39; 3.04] | Low | Very low | Not serious | Very low | -5.68 [-14.39; 3.04] | Very low | . | Serious | Very low | NMA estimate was used because incoherence was not available. Imprecision was rated as Serious. |
| Lentinan+OX | Xihuang Capsules+OX | 0 | . | . | . |  |  |  |  |  | -2.86 [-11.43; 5.71] | Low | Low | Not serious | Low | -2.86 [-11.43; 5.71] | Low | . | Serious | Very low | NMA estimate was used because incoherence was not available. Imprecision was rated as Serious. |
| Lentinan+OX | Ya Dan Zi Oil Emulsion Injection+OX | 0 | . | . | . |  |  |  |  |  | 0.25 [ -8.46; 8.96] | Low | Very low | Not serious | Very low | 0.25 [ -8.46; 8.96] | Very low | . | Serious | Very low | NMA estimate was used because incoherence was not available. Imprecision was rated as Serious. |
| Lentinan+OX | Yangzheng Xiaoji Capsules+OX | 0 | . | . | . |  |  |  |  |  | -1.14 [-11.58; 9.30] | Low | High | Not serious | Low | -1.14 [-11.58; 9.30] | Low | . | Serious | Very low | NMA estimate was used because incoherence was not available. Imprecision was rated as Serious. |
| Lentinan+OX | Zhenqi Fuzheng Granules+OX | 0 | . | . | . |  |  |  |  |  | -2.92 [-13.36; 7.52] | Low | Low | Not serious | Low | -2.92 [-13.36; 7.52] | Low | . | Serious | Very low | NMA estimate was used because incoherence was not available. Imprecision was rated as Serious. |
| Pingxiao Capsules+OX | OX | 1 | 86 | . | 12.47 [ 3.80; 21.14] | Very serious | Not serious | Not serious | Undetected | Low | . |  |  |  |  | 12.47 [ 3.80; 21.14] | Low | . | Not serious | Low | NMA estimate was used because incoherence was not available. Imprecision was rated as Serious. |
| Shenmai Injection+OX | OX | 1 | 82 | . | 10.03 [ 1.65; 18.41] | Not serious | Not serious | Not serious | Undetected | High | . |  |  |  |  | 10.03 [ 1.65; 18.41] | High | . | Not serious | High | NMA estimate was used because incoherence was not available. Imprecision was rated as Serious. |
| Shenqi Fuzheng Injection+OX | OX | 9 | 765 | 93.1% | 7.74 [ 4.86; 10.63] | Serious | Very serious | Not serious | Undetected | Very low | . |  |  |  |  | 7.74 [ 4.86; 10.63] | Very low | . | Not serious | Very low | NMA estimate was used because incoherence was not available. Imprecision was rated as Serious. |
| Shenqi Fuzheng Injection+SOX | OX | 1 | 66 | . | 8.60 [-0.32; 17.52] | Very serious | Not serious | Not serious | Undetected | Low | . |  |  |  |  | 8.60 [ -0.32; 17.52] | Low | . | Serious | Very low | NMA estimate was used because incoherence was not available. Imprecision was rated as Serious. |
| Xiaoaiping Injection+OX | OX | 2 | 384 | 98.5% | 13.86 [ 7.57; 20.14] | Very serious | Very serious | Not serious | Undetected | Very low | . |  |  |  |  | 13.86 [ 7.57; 20.14] | Very low | . | Not serious | Very low | NMA estimate was used because incoherence was not available. Imprecision was rated as Serious. |
| Xihuang Capsules+OX | OX | 2 | 164 | 0.0% | 11.04 [ 4.95; 17.13] | Very serious | Not serious | Not serious | Undetected | Low | . |  |  |  |  | 11.04 [ 4.95; 17.13] | Low | . | Not serious | Low | NMA estimate was used because incoherence was not available. Imprecision was rated as Serious. |
| Ya Dan Zi Oil Emulsion Injection+OX | OX | 2 | 204 | 98.8% | 7.93 [ 1.65; 14.22] | Serious | Very serious | Not serious | Undetected | Very low | . |  |  |  |  | 7.93 [ 1.65; 14.22] | Very low | . | Not serious | Very low | NMA estimate was used because incoherence was not available. Imprecision was rated as Serious. |
| Yangzheng Xiaoji Capsules+OX | OX | 1 | 80 | . | 9.32 [ 0.80; 17.84] | Not serious | Not serious | Not serious | Undetected | High | . |  |  |  |  | 9.32 [ 0.80; 17.84] | High | . | Not serious | High | NMA estimate was used because incoherence was not available. Imprecision was rated as Serious. |
| Zhenqi Fuzheng Granules+OX | OX | 1 | 92 | . | 11.10 [ 2.58; 19.62] | Very serious | Not serious | Not serious | Undetected | Low | . |  |  |  |  | 11.10 [ 2.58; 19.62] | Low | . | Not serious | Low | NMA estimate was used because incoherence was not available. Imprecision was rated as Serious. |
| Pingxiao Capsules+OX | Shenmai Injection+OX | 0 | . | . | . |  |  |  |  |  | 2.44 [ -9.61; 14.49] | Low | High | Not serious | Low | 2.44 [ -9.61; 14.49] | Low | . | Serious | Very low | NMA estimate was used because incoherence was not available. Imprecision was rated as Serious. |
| Pingxiao Capsules+OX | Shenqi Fuzheng Injection+OX | 0 | . | . | . |  |  |  |  |  | 4.73 [ -4.41; 13.86] | Low | Very low | Not serious | Very low | 4.73 [ -4.41; 13.86] | Very low | . | Serious | Very low | NMA estimate was used because incoherence was not available. Imprecision was rated as Serious. |
| Pingxiao Capsules+OX | Shenqi Fuzheng Injection+SOX | 0 | . | . | . |  |  |  |  |  | 3.87 [ -8.56; 16.30] | Low | Low | Not serious | Low | 3.87 [ -8.56; 16.30] | Low | . | Serious | Very low | NMA estimate was used because incoherence was not available. Imprecision was rated as Serious. |
| Pingxiao Capsules+OX | Xiaoaiping Injection+OX | 0 | . | . | . |  |  |  |  |  | -1.39 [-12.09; 9.32] | Low | Very low | Not serious | Very low | -1.39 [-12.09; 9.32] | Very low | . | Serious | Very low | NMA estimate was used because incoherence was not available. Imprecision was rated as Serious. |
| Pingxiao Capsules+OX | Xihuang Capsules+OX | 0 | . | . | . |  |  |  |  |  | 1.43 [ -9.16; 12.02] | Low | Low | Not serious | Low | 1.43 [ -9.16; 12.02] | Low | . | Serious | Very low | NMA estimate was used because incoherence was not available. Imprecision was rated as Serious. |
| Pingxiao Capsules+OX | Ya Dan Zi Oil Emulsion Injection+OX | 0 | . | . | . |  |  |  |  |  | 4.54 [ -6.17; 15.24] | Low | Very low | Not serious | Very low | 4.54 [ -6.17; 15.24] | Very low | . | Serious | Very low | NMA estimate was used because incoherence was not available. Imprecision was rated as Serious. |
| Pingxiao Capsules+OX | Yangzheng Xiaoji Capsules+OX | 0 | . | . | . |  |  |  |  |  | 3.15 [ -9.00; 15.30] | Low | High | Not serious | Low | 3.15 [ -9.00; 15.30] | Low | . | Serious | Very low | NMA estimate was used because incoherence was not available. Imprecision was rated as Serious. |
| Pingxiao Capsules+OX | Zhenqi Fuzheng Granules+OX | 0 | . | . | . |  |  |  |  |  | 1.37 [-10.78; 13.52] | Low | Low | Not serious | Low | 1.37 [-10.78; 13.52] | Low | . | Serious | Very low | NMA estimate was used because incoherence was not available. Imprecision was rated as Serious. |
| Shenmai Injection+OX | Shenqi Fuzheng Injection+OX | 0 | . | . | . |  |  |  |  |  | 2.29 [ -6.57; 11.14] | High | Very low | Not serious | Very low | 2.29 [ -6.57; 11.14] | Very low | . | Serious | Very low | NMA estimate was used because incoherence was not available. Imprecision was rated as Serious. |
| Shenmai Injection+OX | Shenqi Fuzheng Injection+SOX | 0 | . | . | . |  |  |  |  |  | 1.43 [-10.80; 13.66] | High | Low | Not serious | Low | 1.43 [-10.80; 13.66] | Low | . | Serious | Very low | NMA estimate was used because incoherence was not available. Imprecision was rated as Serious. |
| Shenmai Injection+OX | Xiaoaiping Injection+OX | 0 | . | . | . |  |  |  |  |  | -3.83 [-14.30; 6.65] | High | Very low | Not serious | Very low | -3.83 [-14.30; 6.65] | Very low | . | Serious | Very low | NMA estimate was used because incoherence was not available. Imprecision was rated as Serious. |
| Shenmai Injection+OX | Xihuang Capsules+OX | 0 | . | . | . |  |  |  |  |  | -1.01 [-11.36; 9.34] | High | Low | Not serious | Low | -1.01 [-11.36; 9.34] | Low | . | Serious | Very low | NMA estimate was used because incoherence was not available. Imprecision was rated as Serious. |
| Shenmai Injection+OX | Ya Dan Zi Oil Emulsion Injection+OX | 0 | . | . | . |  |  |  |  |  | 2.10 [ -8.38; 12.57] | High | Very low | Not serious | Very low | 2.10 [ -8.38; 12.57] | Very low | . | Serious | Very low | NMA estimate was used because incoherence was not available. Imprecision was rated as Serious. |
| Shenmai Injection+OX | Yangzheng Xiaoji Capsules+OX | 0 | . | . | . |  |  |  |  |  | 0.71 [-11.24; 12.66] | High | High | Not serious | High | 0.71 [-11.24; 12.66] | High | . | Serious | Moderate | NMA estimate was used because incoherence was not available. Imprecision was rated as Serious. |
| Shenmai Injection+OX | Zhenqi Fuzheng Granules+OX | 0 | . | . | . |  |  |  |  |  | -1.07 [-13.02; 10.88] | High | Low | Not serious | Low | -1.07 [-13.02; 10.88] | Low | . | Serious | Very low | NMA estimate was used because incoherence was not available. Imprecision was rated as Serious. |
| Shenqi Fuzheng Injection+OX | Shenqi Fuzheng Injection+SOX | 0 | . | . | . |  |  |  |  |  | -0.86 [-10.23; 8.52] | Very low | Low | Not serious | Very low | -0.86 [-10.23; 8.52] | Very low | . | Serious | Very low | NMA estimate was used because incoherence was not available. Imprecision was rated as Serious. |
| Shenqi Fuzheng Injection+OX | Xiaoaiping Injection+OX | 0 | . | . | . |  |  |  |  |  | -6.11 [-13.03; 0.80] | Very low | Very low | Not serious | Very low | -6.11 [-13.03; 0.80] | Very low | . | Serious | Very low | NMA estimate was used because incoherence was not available. Imprecision was rated as Serious. |
| Shenqi Fuzheng Injection+OX | Xihuang Capsules+OX | 0 | . | . | . |  |  |  |  |  | -3.30 [-10.03; 3.44] | Very low | Low | Not serious | Very low | -3.30 [-10.03; 3.44] | Very low | . | Serious | Very low | NMA estimate was used because incoherence was not available. Imprecision was rated as Serious. |
| Shenqi Fuzheng Injection+OX | Ya Dan Zi Oil Emulsion Injection+OX | 0 | . | . | . |  |  |  |  |  | -0.19 [ -7.10; 6.73] | Very low | Very low | Not serious | Very low | -0.19 [ -7.10; 6.73] | Very low | . | Serious | Very low | NMA estimate was used because incoherence was not available. Imprecision was rated as Serious. |
| Shenqi Fuzheng Injection+OX | Yangzheng Xiaoji Capsules+OX | 0 | . | . | . |  |  |  |  |  | -1.58 [-10.57; 7.42] | Very low | High | Not serious | Very low | -1.58 [-10.57; 7.42] | Very low | . | Serious | Very low | NMA estimate was used because incoherence was not available. Imprecision was rated as Serious. |
| Shenqi Fuzheng Injection+OX | Zhenqi Fuzheng Granules+OX | 0 | . | . | . |  |  |  |  |  | -3.36 [-12.35; 5.64] | Very low | Low | Not serious | Very low | -3.36 [-12.35; 5.64] | Very low | . | Serious | Very low | NMA estimate was used because incoherence was not available. Imprecision was rated as Serious. |
| Shenqi Fuzheng Injection+SOX | Xiaoaiping Injection+OX | 0 | . | . | . |  |  |  |  |  | -5.26 [-16.17; 5.65] | Low | Very low | Not serious | Very low | -5.26 [-16.17; 5.65] | Very low | . | Serious | Very low | NMA estimate was used because incoherence was not available. Imprecision was rated as Serious. |
| Shenqi Fuzheng Injection+SOX | Xihuang Capsules+OX | 0 | . | . | . |  |  |  |  |  | -2.44 [-13.24; 8.36] | Low | Low | Not serious | Low | -2.44 [-13.24; 8.36] | Low | . | Serious | Very low | NMA estimate was used because incoherence was not available. Imprecision was rated as Serious. |
| Shenqi Fuzheng Injection+SOX | Ya Dan Zi Oil Emulsion Injection+OX | 0 | . | . | . |  |  |  |  |  | 0.67 [-10.24; 11.58] | Low | Very low | Not serious | Very low | 0.67 [-10.24; 11.58] | Very low | . | Serious | Very low | NMA estimate was used because incoherence was not available. Imprecision was rated as Serious. |
| Shenqi Fuzheng Injection+SOX | Yangzheng Xiaoji Capsules+OX | 0 | . | . | . |  |  |  |  |  | -0.72 [-13.05; 11.61] | Low | High | Not serious | Low | -0.72 [-13.05; 11.61] | Low | . | Serious | Very low | NMA estimate was used because incoherence was not available. Imprecision was rated as Serious. |
| Shenqi Fuzheng Injection+SOX | Zhenqi Fuzheng Granules+OX | 0 | . | . | . |  |  |  |  |  | -2.50 [-14.83; 9.83] | Low | Low | Not serious | Low | -2.50 [-14.83; 9.83] | Low | . | Serious | Very low | NMA estimate was used because incoherence was not available. Imprecision was rated as Serious. |
| Xiaoaiping Injection+OX | Xihuang Capsules+OX | 0 | . | . | . |  |  |  |  |  | 2.82 [ -5.94; 11.57] | Very low | Low | Not serious | Very low | 2.82 [ -5.94; 11.57] | Very low | . | Serious | Very low | NMA estimate was used because incoherence was not available. Imprecision was rated as Serious. |
| Xiaoaiping Injection+OX | Ya Dan Zi Oil Emulsion Injection+OX | 0 | . | . | . |  |  |  |  |  | 5.92 [ -2.97; 14.81] | Very low | Very low | Not serious | Very low | 5.92 [ -2.97; 14.81] | Very low | . | Serious | Very low | NMA estimate was used because incoherence was not available. Imprecision was rated as Serious. |
| Xiaoaiping Injection+OX | Yangzheng Xiaoji Capsules+OX | 0 | . | . | . |  |  |  |  |  | 4.54 [ -6.05; 15.13] | Very low | High | Not serious | Very low | 4.54 [ -6.05; 15.13] | Very low | . | Serious | Very low | NMA estimate was used because incoherence was not available. Imprecision was rated as Serious. |
| Xiaoaiping Injection+OX | Zhenqi Fuzheng Granules+OX | 0 | . | . | . |  |  |  |  |  | 2.76 [ -7.83; 13.34] | Very low | Low | Not serious | Very low | 2.76 [ -7.83; 13.34] | Very low | . | Serious | Very low | NMA estimate was used because incoherence was not available. Imprecision was rated as Serious. |
| Xihuang Capsules+OX | Ya Dan Zi Oil Emulsion Injection+OX | 0 | . | . | . |  |  |  |  |  | 3.11 [ -5.64; 11.86] | Low | Very low | Not serious | Very low | 3.11 [ -5.64; 11.86] | Very low | . | Serious | Very low | NMA estimate was used because incoherence was not available. Imprecision was rated as Serious. |
| Xihuang Capsules+OX | Yangzheng Xiaoji Capsules+OX | 0 | . | . | . |  |  |  |  |  | 1.72 [ -8.75; 12.19] | Low | High | Not serious | Low | 1.72 [ -8.75; 12.19] | Low | . | Serious | Very low | NMA estimate was used because incoherence was not available. Imprecision was rated as Serious. |
| Xihuang Capsules+OX | Zhenqi Fuzheng Granules+OX | 0 | . | . | . |  |  |  |  |  | -0.06 [-10.53; 10.41] | Low | Low | Not serious | Low | -0.06 [-10.53; 10.41] | Low | . | Serious | Very low | NMA estimate was used because incoherence was not available. Imprecision was rated as Serious. |
| Ya Dan Zi Oil Emulsion Injection+OX | Yangzheng Xiaoji Capsules+OX | 0 | . | . | . |  |  |  |  |  | -1.39 [-11.98; 9.20] | Very low | High | Not serious | Very low | -1.39 [-11.98; 9.20] | Very low | . | Serious | Very low | NMA estimate was used because incoherence was not available. Imprecision was rated as Serious. |
| Ya Dan Zi Oil Emulsion Injection+OX | Zhenqi Fuzheng Granules+OX | 0 | . | . | . |  |  |  |  |  | -3.17 [-13.75; 7.42] | Very low | Low | Not serious | Very low | -3.17 [-13.75; 7.42] | Very low | . | Serious | Very low | NMA estimate was used because incoherence was not available. Imprecision was rated as Serious. |
| Yangzheng Xiaoji Capsules+OX | Zhenqi Fuzheng Granules+OX | 0 | . | . | . |  |  |  |  |  | -1.78 [-13.83; 10.27] | High | Low | Not serious | Low | -1.78 [-13.83; 10.27] | Low | . | Serious | Very low | NMA estimate was used because incoherence was not available. Imprecision was rated as Serious. |

1. **CD4+**

| **Arm_1** | **Arm_2** | **No_of_study** | **Sample_size** | **I2** | **Direct_estimate** | **ROB** | **Inconsistency** | **Indirectness** | **Publication_bias** | **Direct_rating_without_imprecision** | **Indirect_estimate** | **Certainty_of_evidence_for_arm1** | **Certainty_of_evidence_for_arm2** | **Intransitivity** | **Indirect_rating_without_imprecision** | **Network_meta_analysis** | **Higher_rating_of_direct_and_indirect_without_imprecision** | **Incoherence** | **NMA_Imprecision** | **Final_network_rating** | **Final_rating_reason** |
| --- | --- | --- | --- | --- | --- | --- | --- | --- | --- | --- | --- | --- | --- | --- | --- | --- | --- | --- | --- | --- | --- |
| Aidi Injection+OX | Astragalus Polysaccharides+OX | 0 | . | . | . |  |  |  |  |  | -3.93 [-10.45; 2.59] | Low | Very low | Not serious | Very low | -3.93 [-10.45; 2.59] | Very low | . | Serious | Very low | NMA estimate was used because incoherence was not available. Imprecision was rated as Serious. |
| Aidi Injection+OX | Astragalus preparations+OX | 0 | . | . | . |  |  |  |  |  | 5.50 [ -0.12; 11.12] | Low | Very low | Not serious | Very low | 5.50 [ -0.12; 11.12] | Very low | . | Serious | Very low | NMA estimate was used because incoherence was not available. Imprecision was rated as Serious. |
| Aidi Injection+OX | Compound Kushen Injection+OX | 0 | . | . | . |  |  |  |  |  | 0.66 [ -3.34; 4.65] | Low | Very low | Not serious | Very low | 0.66 [ -3.34; 4.65] | Very low | . | Serious | Very low | NMA estimate was used because incoherence was not available. Imprecision was rated as Serious. |
| Aidi Injection+OX | Compound Mylabris preparations+OX | 0 | . | . | . |  |  |  |  |  | 2.73 [ -2.42; 7.87] | Low | Low | Not serious | Low | 2.73 [ -2.42; 7.87] | Low | . | Serious | Very low | NMA estimate was used because incoherence was not available. Imprecision was rated as Serious. |
| Aidi Injection+OX | Diyu Shengbai Tablet+OX | 0 | . | . | . |  |  |  |  |  | -1.61 [-10.01; 6.80] | Low | Low | Not serious | Low | -1.61 [-10.01; 6.80] | Low | . | Serious | Very low | NMA estimate was used because incoherence was not available. Imprecision was rated as Serious. |
| Aidi Injection+OX | Ginseng Polysaccharide Injection+OX | 0 | . | . | . |  |  |  |  |  | 3.06 [ -5.95; 12.08] | Low | Low | Not serious | Low | 3.06 [ -5.95; 12.08] | Low | . | Serious | Very low | NMA estimate was used because incoherence was not available. Imprecision was rated as Serious. |
| Aidi Injection+OX | Huachansu preparations+OX | 0 | . | . | . |  |  |  |  |  | 1.42 [ -3.45; 6.29] | Low | High | Not serious | Low | 1.42 [ -3.45; 6.29] | Low | . | Serious | Very low | NMA estimate was used because incoherence was not available. Imprecision was rated as Serious. |
| Aidi Injection+OX | Jinlong Capsules+OX | 0 | . | . | . |  |  |  |  |  | 2.46 [ -2.89; 7.81] | Low | Very low | Not serious | Very low | 2.46 [ -2.89; 7.81] | Very low | . | Serious | Very low | NMA estimate was used because incoherence was not available. Imprecision was rated as Serious. |
| Aidi Injection+OX | Kangai Injection+OX | 0 | . | . | . |  |  |  |  |  | -0.68 [ -5.83; 4.47] | Low | Very low | Not serious | Very low | -0.68 [ -5.83; 4.47] | Very low | . | Serious | Very low | NMA estimate was used because incoherence was not available. Imprecision was rated as Serious. |
| Aidi Injection+OX | Kanglixin Capsules+OX | 0 | . | . | . |  |  |  |  |  | -0.26 [ -6.76; 6.23] | Low | Very low | Not serious | Very low | -0.26 [ -6.76; 6.23] | Very low | . | Serious | Very low | NMA estimate was used because incoherence was not available. Imprecision was rated as Serious. |
| Aidi Injection+OX | Lentinan+OX | 0 | . | . | . |  |  |  |  |  | -1.54 [ -8.10; 5.03] | Low | High | Not serious | Low | -1.54 [ -8.10; 5.03] | Low | . | Serious | Very low | NMA estimate was used because incoherence was not available. Imprecision was rated as Serious. |
| Aidi Injection+OX | OX | 6 | 612 | 87.1% | 7.89 [ 4.55; 11.24] | Very serious | Not serious | Not serious | Undetected | Low | . |  |  |  |  | 7.89 [ 4.55; 11.24] | Low | . | Not serious | Low | NMA estimate was used because incoherence was not available. Imprecision was rated as Serious. |
| Aidi Injection+OX | Pingxiao Capsules+OX | 0 | . | . | . |  |  |  |  |  | 0.66 [ -7.89; 9.22] | Low | Low | Not serious | Low | 0.66 [ -7.89; 9.22] | Low | . | Serious | Very low | NMA estimate was used because incoherence was not available. Imprecision was rated as Serious. |
| Aidi Injection+OX | Shenmai Injection+OX | 0 | . | . | . |  |  |  |  |  | -0.44 [ -8.82; 7.95] | Low | High | Not serious | Low | -0.44 [ -8.82; 7.95] | Low | . | Serious | Very low | NMA estimate was used because incoherence was not available. Imprecision was rated as Serious. |
| Aidi Injection+OX | Shenqi Fuzheng Injection+OX | 0 | . | . | . |  |  |  |  |  | 2.17 [ -1.75; 6.09] | Low | Moderate | Not serious | Low | 2.17 [ -1.75; 6.09] | Low | . | Serious | Very low | NMA estimate was used because incoherence was not available. Imprecision was rated as Serious. |
| Aidi Injection+OX | Xiaoaiping Injection+OX | 0 | . | . | . |  |  |  |  |  | -1.94 [ -8.41; 4.52] | Low | Very low | Not serious | Very low | -1.94 [ -8.41; 4.52] | Very low | . | Serious | Very low | NMA estimate was used because incoherence was not available. Imprecision was rated as Serious. |
| Aidi Injection+OX | Xihuang Capsules+OX | 0 | . | . | . |  |  |  |  |  | 3.45 [ -3.06; 9.96] | Low | Low | Not serious | Low | 3.45 [ -3.06; 9.96] | Low | . | Serious | Very low | NMA estimate was used because incoherence was not available. Imprecision was rated as Serious. |
| Aidi Injection+OX | Ya Dan Zi Oil Emulsion Injection+OX | 0 | . | . | . |  |  |  |  |  | 2.66 [ -3.97; 9.29] | Low | Very low | Not serious | Very low | 2.66 [ -3.97; 9.29] | Very low | . | Serious | Very low | NMA estimate was used because incoherence was not available. Imprecision was rated as Serious. |
| Aidi Injection+OX | Yangzheng Xiaoji Capsules+OX | 0 | . | . | . |  |  |  |  |  | 2.78 [ -5.75; 11.32] | Low | High | Not serious | Low | 2.78 [ -5.75; 11.32] | Low | . | Serious | Very low | NMA estimate was used because incoherence was not available. Imprecision was rated as Serious. |
| Aidi Injection+OX | Zhenqi Fuzheng Granules+OX | 0 | . | . | . |  |  |  |  |  | -1.21 [ -9.84; 7.43] | Low | Low | Not serious | Low | -1.21 [ -9.84; 7.43] | Low | . | Serious | Very low | NMA estimate was used because incoherence was not available. Imprecision was rated as Serious. |
| Astragalus Polysaccharides+OX | Astragalus preparations+OX | 0 | . | . | . |  |  |  |  |  | 9.43 [ 2.23; 16.62] | Very low | Very low | Not serious | Very low | 9.43 [ 2.23; 16.62] | Very low | . | Not serious | Very low | NMA estimate was used because incoherence was not available. Imprecision was rated as Serious. |
| Astragalus Polysaccharides+OX | Compound Kushen Injection+OX | 0 | . | . | . |  |  |  |  |  | 4.59 [ -1.42; 10.60] | Very low | Very low | Not serious | Very low | 4.59 [ -1.42; 10.60] | Very low | . | Serious | Very low | NMA estimate was used because incoherence was not available. Imprecision was rated as Serious. |
| Astragalus Polysaccharides+OX | Compound Mylabris preparations+OX | 0 | . | . | . |  |  |  |  |  | 6.66 [ -0.17; 13.48] | Very low | Low | Not serious | Very low | 6.66 [ -0.17; 13.48] | Very low | . | Serious | Very low | NMA estimate was used because incoherence was not available. Imprecision was rated as Serious. |
| Astragalus Polysaccharides+OX | Diyu Shengbai Tablet+OX | 0 | . | . | . |  |  |  |  |  | 2.32 [ -7.20; 11.85] | Very low | Low | Not serious | Very low | 2.32 [ -7.20; 11.85] | Very low | . | Serious | Very low | NMA estimate was used because incoherence was not available. Imprecision was rated as Serious. |
| Astragalus Polysaccharides+OX | Ginseng Polysaccharide Injection+OX | 0 | . | . | . |  |  |  |  |  | 6.99 [ -3.07; 17.06] | Very low | Low | Not serious | Very low | 6.99 [ -3.07; 17.06] | Very low | . | Serious | Very low | NMA estimate was used because incoherence was not available. Imprecision was rated as Serious. |
| Astragalus Polysaccharides+OX | Huachansu preparations+OX | 0 | . | . | . |  |  |  |  |  | 5.35 [ -1.27; 11.97] | Very low | High | Not serious | Very low | 5.35 [ -1.27; 11.97] | Very low | . | Serious | Very low | NMA estimate was used because incoherence was not available. Imprecision was rated as Serious. |
| Astragalus Polysaccharides+OX | Jinlong Capsules+OX | 0 | . | . | . |  |  |  |  |  | 6.39 [ -0.59; 13.37] | Very low | Very low | Not serious | Very low | 6.39 [ -0.59; 13.37] | Very low | . | Serious | Very low | NMA estimate was used because incoherence was not available. Imprecision was rated as Serious. |
| Astragalus Polysaccharides+OX | Kangai Injection+OX | 0 | . | . | . |  |  |  |  |  | 3.25 [ -3.58; 10.08] | Very low | Very low | Not serious | Very low | 3.25 [ -3.58; 10.08] | Very low | . | Serious | Very low | NMA estimate was used because incoherence was not available. Imprecision was rated as Serious. |
| Astragalus Polysaccharides+OX | Kanglixin Capsules+OX | 0 | . | . | . |  |  |  |  |  | 3.67 [ -4.23; 11.56] | Very low | Very low | Not serious | Very low | 3.67 [ -4.23; 11.56] | Very low | . | Serious | Very low | NMA estimate was used because incoherence was not available. Imprecision was rated as Serious. |
| Astragalus Polysaccharides+OX | Lentinan+OX | 0 | . | . | . |  |  |  |  |  | 2.39 [ -5.56; 10.34] | Very low | High | Not serious | Very low | 2.39 [ -5.56; 10.34] | Very low | . | Serious | Very low | NMA estimate was used because incoherence was not available. Imprecision was rated as Serious. |
| Astragalus Polysaccharides+OX | OX | 2 | 151 | 94.6% | 11.82 [ 6.23; 17.42] | Very serious | Very serious | Not serious | Undetected | Very low | . |  |  |  |  | 11.82 [ 6.23; 17.42] | Very low | . | Not serious | Very low | NMA estimate was used because incoherence was not available. Imprecision was rated as Serious. |
| Astragalus Polysaccharides+OX | Pingxiao Capsules+OX | 0 | . | . | . |  |  |  |  |  | 4.59 [ -5.06; 14.25] | Very low | Low | Not serious | Very low | 4.59 [ -5.06; 14.25] | Very low | . | Serious | Very low | NMA estimate was used because incoherence was not available. Imprecision was rated as Serious. |
| Astragalus Polysaccharides+OX | Shenmai Injection+OX | 0 | . | . | . |  |  |  |  |  | 3.49 [ -6.02; 13.01] | Very low | High | Not serious | Very low | 3.49 [ -6.02; 13.01] | Very low | . | Serious | Very low | NMA estimate was used because incoherence was not available. Imprecision was rated as Serious. |
| Astragalus Polysaccharides+OX | Shenqi Fuzheng Injection+OX | 0 | . | . | . |  |  |  |  |  | 6.10 [ 0.15; 12.06] | Very low | Moderate | Not serious | Very low | 6.10 [ 0.15; 12.06] | Very low | . | Not serious | Very low | NMA estimate was used because incoherence was not available. Imprecision was rated as Serious. |
| Astragalus Polysaccharides+OX | Xiaoaiping Injection+OX | 0 | . | . | . |  |  |  |  |  | 1.99 [ -5.88; 9.85] | Very low | Very low | Not serious | Very low | 1.99 [ -5.88; 9.85] | Very low | . | Serious | Very low | NMA estimate was used because incoherence was not available. Imprecision was rated as Serious. |
| Astragalus Polysaccharides+OX | Xihuang Capsules+OX | 0 | . | . | . |  |  |  |  |  | 7.38 [ -0.53; 15.29] | Very low | Low | Not serious | Very low | 7.38 [ -0.53; 15.29] | Very low | . | Serious | Very low | NMA estimate was used because incoherence was not available. Imprecision was rated as Serious. |
| Astragalus Polysaccharides+OX | Ya Dan Zi Oil Emulsion Injection+OX | 0 | . | . | . |  |  |  |  |  | 6.59 [ -1.41; 14.59] | Very low | Very low | Not serious | Very low | 6.59 [ -1.41; 14.59] | Very low | . | Serious | Very low | NMA estimate was used because incoherence was not available. Imprecision was rated as Serious. |
| Astragalus Polysaccharides+OX | Yangzheng Xiaoji Capsules+OX | 0 | . | . | . |  |  |  |  |  | 6.71 [ -2.93; 16.35] | Very low | High | Not serious | Very low | 6.71 [ -2.93; 16.35] | Very low | . | Serious | Very low | NMA estimate was used because incoherence was not available. Imprecision was rated as Serious. |
| Astragalus Polysaccharides+OX | Zhenqi Fuzheng Granules+OX | 0 | . | . | . |  |  |  |  |  | 2.72 [ -7.01; 12.45] | Very low | Low | Not serious | Very low | 2.72 [ -7.01; 12.45] | Very low | . | Serious | Very low | NMA estimate was used because incoherence was not available. Imprecision was rated as Serious. |
| Astragalus preparations+OX | Compound Kushen Injection+OX | 0 | . | . | . |  |  |  |  |  | -4.84 [ -9.86; 0.18] | Very low | Very low | Not serious | Very low | -4.84 [ -9.86; 0.18] | Very low | . | Serious | Very low | NMA estimate was used because incoherence was not available. Imprecision was rated as Serious. |
| Astragalus preparations+OX | Compound Mylabris preparations+OX | 0 | . | . | . |  |  |  |  |  | -2.77 [ -8.75; 3.20] | Very low | Low | Not serious | Very low | -2.77 [ -8.75; 3.20] | Very low | . | Serious | Very low | NMA estimate was used because incoherence was not available. Imprecision was rated as Serious. |
| Astragalus preparations+OX | Diyu Shengbai Tablet+OX | 0 | . | . | . |  |  |  |  |  | -7.10 [-16.04; 1.83] | Very low | Low | Not serious | Very low | -7.10 [-16.04; 1.83] | Very low | . | Serious | Very low | NMA estimate was used because incoherence was not available. Imprecision was rated as Serious. |
| Astragalus preparations+OX | Ginseng Polysaccharide Injection+OX | 0 | . | . | . |  |  |  |  |  | -2.43 [-11.95; 7.08] | Very low | Low | Not serious | Very low | -2.43 [-11.95; 7.08] | Very low | . | Serious | Very low | NMA estimate was used because incoherence was not available. Imprecision was rated as Serious. |
| Astragalus preparations+OX | Huachansu preparations+OX | 0 | . | . | . |  |  |  |  |  | -4.08 [ -9.82; 1.66] | Very low | High | Not serious | Very low | -4.08 [ -9.82; 1.66] | Very low | . | Serious | Very low | NMA estimate was used because incoherence was not available. Imprecision was rated as Serious. |
| Astragalus preparations+OX | Jinlong Capsules+OX | 0 | . | . | . |  |  |  |  |  | -3.04 [ -9.19; 3.12] | Very low | Very low | Not serious | Very low | -3.04 [ -9.19; 3.12] | Very low | . | Serious | Very low | NMA estimate was used because incoherence was not available. Imprecision was rated as Serious. |
| Astragalus preparations+OX | Kangai Injection+OX | 0 | . | . | . |  |  |  |  |  | -6.18 [-12.16; -0.20] | Very low | Very low | Not serious | Very low | -6.18 [-12.16; -0.20] | Very low | . | Serious | Very low | NMA estimate was used because incoherence was not available. Imprecision was rated as Serious. |
| Astragalus preparations+OX | Kanglixin Capsules+OX | 0 | . | . | . |  |  |  |  |  | -5.76 [-12.93; 1.41] | Very low | Very low | Not serious | Very low | -5.76 [-12.93; 1.41] | Very low | . | Serious | Very low | NMA estimate was used because incoherence was not available. Imprecision was rated as Serious. |
| Astragalus preparations+OX | Lentinan+OX | 0 | . | . | . |  |  |  |  |  | -7.04 [-14.27; 0.20] | Very low | High | Not serious | Very low | -7.04 [-14.27; 0.20] | Very low | . | Serious | Very low | NMA estimate was used because incoherence was not available. Imprecision was rated as Serious. |
| Astragalus preparations+OX | OX | 3 | 295 | 98.9% | 2.40 [-2.12; 6.92] | Very serious | Very serious | Not serious | Undetected | Very low | . |  |  |  |  | 2.40 [ -2.12; 6.92] | Very low | . | Serious | Very low | NMA estimate was used because incoherence was not available. Imprecision was rated as Serious. |
| Astragalus preparations+OX | Pingxiao Capsules+OX | 0 | . | . | . |  |  |  |  |  | -4.83 [-13.91; 4.24] | Very low | Low | Not serious | Very low | -4.83 [-13.91; 4.24] | Very low | . | Serious | Very low | NMA estimate was used because incoherence was not available. Imprecision was rated as Serious. |
| Astragalus preparations+OX | Shenmai Injection+OX | 0 | . | . | . |  |  |  |  |  | -5.93 [-14.86; 2.99] | Very low | High | Not serious | Very low | -5.93 [-14.86; 2.99] | Very low | . | Serious | Very low | NMA estimate was used because incoherence was not available. Imprecision was rated as Serious. |
| Astragalus preparations+OX | Shenqi Fuzheng Injection+OX | 0 | . | . | . |  |  |  |  |  | -3.33 [ -8.29; 1.63] | Very low | Moderate | Not serious | Very low | -3.33 [ -8.29; 1.63] | Very low | . | Serious | Very low | NMA estimate was used because incoherence was not available. Imprecision was rated as Serious. |
| Astragalus preparations+OX | Xiaoaiping Injection+OX | 0 | . | . | . |  |  |  |  |  | -7.44 [-14.59; -0.30] | Very low | Very low | Not serious | Very low | -7.44 [-14.59; -0.30] | Very low | . | Serious | Very low | NMA estimate was used because incoherence was not available. Imprecision was rated as Serious. |
| Astragalus preparations+OX | Xihuang Capsules+OX | 0 | . | . | . |  |  |  |  |  | -2.05 [ -9.23; 5.14] | Very low | Low | Not serious | Very low | -2.05 [ -9.23; 5.14] | Very low | . | Serious | Very low | NMA estimate was used because incoherence was not available. Imprecision was rated as Serious. |
| Astragalus preparations+OX | Ya Dan Zi Oil Emulsion Injection+OX | 0 | . | . | . |  |  |  |  |  | -2.84 [-10.13; 4.45] | Very low | Very low | Not serious | Very low | -2.84 [-10.13; 4.45] | Very low | . | Serious | Very low | NMA estimate was used because incoherence was not available. Imprecision was rated as Serious. |
| Astragalus preparations+OX | Yangzheng Xiaoji Capsules+OX | 0 | . | . | . |  |  |  |  |  | -2.71 [-11.77; 6.34] | Very low | High | Not serious | Very low | -2.71 [-11.77; 6.34] | Very low | . | Serious | Very low | NMA estimate was used because incoherence was not available. Imprecision was rated as Serious. |
| Astragalus preparations+OX | Zhenqi Fuzheng Granules+OX | 0 | . | . | . |  |  |  |  |  | -6.70 [-15.86; 2.45] | Very low | Low | Not serious | Very low | -6.70 [-15.86; 2.45] | Very low | . | Serious | Very low | NMA estimate was used because incoherence was not available. Imprecision was rated as Serious. |
| Compound Kushen Injection+OX | Compound Mylabris preparations+OX | 0 | . | . | . |  |  |  |  |  | 2.07 [ -2.41; 6.54] | Very low | Low | Not serious | Very low | 2.07 [ -2.41; 6.54] | Very low | . | Serious | Very low | NMA estimate was used because incoherence was not available. Imprecision was rated as Serious. |
| Compound Kushen Injection+OX | Diyu Shengbai Tablet+OX | 0 | . | . | . |  |  |  |  |  | -2.26 [-10.28; 5.75] | Very low | Low | Not serious | Very low | -2.26 [-10.28; 5.75] | Very low | . | Serious | Very low | NMA estimate was used because incoherence was not available. Imprecision was rated as Serious. |
| Compound Kushen Injection+OX | Ginseng Polysaccharide Injection+OX | 0 | . | . | . |  |  |  |  |  | 2.41 [ -6.24; 11.06] | Very low | Low | Not serious | Very low | 2.41 [ -6.24; 11.06] | Very low | . | Serious | Very low | NMA estimate was used because incoherence was not available. Imprecision was rated as Serious. |
| Compound Kushen Injection+OX | Huachansu preparations+OX | 0 | . | . | . |  |  |  |  |  | 0.76 [ -3.40; 4.92] | Very low | High | Not serious | Very low | 0.76 [ -3.40; 4.92] | Very low | . | Serious | Very low | NMA estimate was used because incoherence was not available. Imprecision was rated as Serious. |
| Compound Kushen Injection+OX | Jinlong Capsules+OX | 0 | . | . | . |  |  |  |  |  | 1.80 [ -2.91; 6.51] | Very low | Very low | Not serious | Very low | 1.80 [ -2.91; 6.51] | Very low | . | Serious | Very low | NMA estimate was used because incoherence was not available. Imprecision was rated as Serious. |
| Compound Kushen Injection+OX | Kangai Injection+OX | 0 | . | . | . |  |  |  |  |  | -1.34 [ -5.82; 3.14] | Very low | Very low | Not serious | Very low | -1.34 [ -5.82; 3.14] | Very low | . | Serious | Very low | NMA estimate was used because incoherence was not available. Imprecision was rated as Serious. |
| Compound Kushen Injection+OX | Kanglixin Capsules+OX | 0 | . | . | . |  |  |  |  |  | -0.92 [ -6.90; 5.06] | Very low | Very low | Not serious | Very low | -0.92 [ -6.90; 5.06] | Very low | . | Serious | Very low | NMA estimate was used because incoherence was not available. Imprecision was rated as Serious. |
| Compound Kushen Injection+OX | Lentinan+OX | 0 | . | . | . |  |  |  |  |  | -2.20 [ -8.25; 3.86] | Very low | High | Not serious | Very low | -2.20 [ -8.25; 3.86] | Very low | . | Serious | Very low | NMA estimate was used because incoherence was not available. Imprecision was rated as Serious. |
| Compound Kushen Injection+OX | OX | 13 | 1177 | 98.4% | 7.24 [ 5.05; 9.42] | Very serious | Very serious | Not serious | Serious | Very low | . |  |  |  |  | 7.24 [ 5.05; 9.42] | Very low | . | Not serious | Very low | NMA estimate was used because incoherence was not available. Imprecision was rated as Not serious. |
| Compound Kushen Injection+OX | Pingxiao Capsules+OX | 0 | . | . | . |  |  |  |  |  | 0.01 [ -8.16; 8.17] | Very low | Low | Not serious | Very low | 0.01 [ -8.16; 8.17] | Very low | . | Serious | Very low | NMA estimate was used because incoherence was not available. Imprecision was rated as Serious. |
| Compound Kushen Injection+OX | Shenmai Injection+OX | 0 | . | . | . |  |  |  |  |  | -1.09 [ -9.09; 6.90] | Very low | High | Not serious | Very low | -1.09 [ -9.09; 6.90] | Very low | . | Serious | Very low | NMA estimate was used because incoherence was not available. Imprecision was rated as Serious. |
| Compound Kushen Injection+OX | Shenqi Fuzheng Injection+OX | 0 | . | . | . |  |  |  |  |  | 1.51 [ -1.47; 4.50] | Very low | Moderate | Not serious | Very low | 1.51 [ -1.47; 4.50] | Very low | . | Serious | Very low | NMA estimate was used because incoherence was not available. Imprecision was rated as Serious. |
| Compound Kushen Injection+OX | Xiaoaiping Injection+OX | 0 | . | . | . |  |  |  |  |  | -2.60 [ -8.55; 3.34] | Very low | Very low | Not serious | Very low | -2.60 [ -8.55; 3.34] | Very low | . | Serious | Very low | NMA estimate was used because incoherence was not available. Imprecision was rated as Serious. |
| Compound Kushen Injection+OX | Xihuang Capsules+OX | 0 | . | . | . |  |  |  |  |  | 2.79 [ -3.21; 8.79] | Very low | Low | Not serious | Very low | 2.79 [ -3.21; 8.79] | Very low | . | Serious | Very low | NMA estimate was used because incoherence was not available. Imprecision was rated as Serious. |
| Compound Kushen Injection+OX | Ya Dan Zi Oil Emulsion Injection+OX | 0 | . | . | . |  |  |  |  |  | 2.00 [ -4.12; 8.12] | Very low | Very low | Not serious | Very low | 2.00 [ -4.12; 8.12] | Very low | . | Serious | Very low | NMA estimate was used because incoherence was not available. Imprecision was rated as Serious. |
| Compound Kushen Injection+OX | Yangzheng Xiaoji Capsules+OX | 0 | . | . | . |  |  |  |  |  | 2.13 [ -6.02; 10.27] | Very low | High | Not serious | Very low | 2.13 [ -6.02; 10.27] | Very low | . | Serious | Very low | NMA estimate was used because incoherence was not available. Imprecision was rated as Serious. |
| Compound Kushen Injection+OX | Zhenqi Fuzheng Granules+OX | 0 | . | . | . |  |  |  |  |  | -1.86 [-10.12; 6.39] | Very low | Low | Not serious | Very low | -1.86 [-10.12; 6.39] | Very low | . | Serious | Very low | NMA estimate was used because incoherence was not available. Imprecision was rated as Serious. |
| Compound Mylabris preparations+OX | Diyu Shengbai Tablet+OX | 0 | . | . | . |  |  |  |  |  | -4.33 [-12.97; 4.31] | Low | Low | Not serious | Low | -4.33 [-12.97; 4.31] | Low | . | Serious | Very low | NMA estimate was used because incoherence was not available. Imprecision was rated as Serious. |
| Compound Mylabris preparations+OX | Ginseng Polysaccharide Injection+OX | 0 | . | . | . |  |  |  |  |  | 0.34 [ -8.90; 9.58] | Low | Low | Not serious | Low | 0.34 [ -8.90; 9.58] | Low | . | Serious | Very low | NMA estimate was used because incoherence was not available. Imprecision was rated as Serious. |
| Compound Mylabris preparations+OX | Huachansu preparations+OX | 0 | . | . | . |  |  |  |  |  | -1.31 [ -6.58; 3.97] | Low | High | Not serious | Low | -1.31 [ -6.58; 3.97] | Low | . | Serious | Very low | NMA estimate was used because incoherence was not available. Imprecision was rated as Serious. |
| Compound Mylabris preparations+OX | Jinlong Capsules+OX | 0 | . | . | . |  |  |  |  |  | -0.27 [ -5.99; 5.45] | Low | Very low | Not serious | Very low | -0.27 [ -5.99; 5.45] | Very low | . | Serious | Very low | NMA estimate was used because incoherence was not available. Imprecision was rated as Serious. |
| Compound Mylabris preparations+OX | Kangai Injection+OX | 0 | . | . | . |  |  |  |  |  | -3.41 [ -8.94; 2.12] | Low | Very low | Not serious | Very low | -3.41 [ -8.94; 2.12] | Very low | . | Serious | Very low | NMA estimate was used because incoherence was not available. Imprecision was rated as Serious. |
| Compound Mylabris preparations+OX | Kanglixin Capsules+OX | 0 | . | . | . |  |  |  |  |  | -2.99 [ -9.79; 3.81] | Low | Very low | Not serious | Very low | -2.99 [ -9.79; 3.81] | Very low | . | Serious | Very low | NMA estimate was used because incoherence was not available. Imprecision was rated as Serious. |
| Compound Mylabris preparations+OX | Lentinan+OX | 0 | . | . | . |  |  |  |  |  | -4.26 [-11.13; 2.61] | Low | High | Not serious | Low | -4.26 [-11.13; 2.61] | Low | . | Serious | Very low | NMA estimate was used because incoherence was not available. Imprecision was rated as Serious. |
| Compound Mylabris preparations+OX | OX | 4 | 333 | 89.8% | 5.17 [ 1.26; 9.08] | Very serious | Not serious | Not serious | Undetected | Low | . |  |  |  |  | 5.17 [ 1.26; 9.08] | Low | . | Not serious | Low | NMA estimate was used because incoherence was not available. Imprecision was rated as Serious. |
| Compound Mylabris preparations+OX | Pingxiao Capsules+OX | 0 | . | . | . |  |  |  |  |  | -2.06 [-10.85; 6.72] | Low | Low | Not serious | Low | -2.06 [-10.85; 6.72] | Low | . | Serious | Very low | NMA estimate was used because incoherence was not available. Imprecision was rated as Serious. |
| Compound Mylabris preparations+OX | Shenmai Injection+OX | 0 | . | . | . |  |  |  |  |  | -3.16 [-11.79; 5.47] | Low | High | Not serious | Low | -3.16 [-11.79; 5.47] | Low | . | Serious | Very low | NMA estimate was used because incoherence was not available. Imprecision was rated as Serious. |
| Compound Mylabris preparations+OX | Shenqi Fuzheng Injection+OX | 0 | . | . | . |  |  |  |  |  | -0.55 [ -4.96; 3.85] | Low | Moderate | Not serious | Low | -0.55 [ -4.96; 3.85] | Low | . | Serious | Very low | NMA estimate was used because incoherence was not available. Imprecision was rated as Serious. |
| Compound Mylabris preparations+OX | Xiaoaiping Injection+OX | 0 | . | . | . |  |  |  |  |  | -4.67 [-11.44; 2.10] | Low | Very low | Not serious | Very low | -4.67 [-11.44; 2.10] | Very low | . | Serious | Very low | NMA estimate was used because incoherence was not available. Imprecision was rated as Serious. |
| Compound Mylabris preparations+OX | Xihuang Capsules+OX | 0 | . | . | . |  |  |  |  |  | 0.72 [ -6.09; 7.54] | Low | Low | Not serious | Low | 0.72 [ -6.09; 7.54] | Low | . | Serious | Very low | NMA estimate was used because incoherence was not available. Imprecision was rated as Serious. |
| Compound Mylabris preparations+OX | Ya Dan Zi Oil Emulsion Injection+OX | 0 | . | . | . |  |  |  |  |  | -0.07 [ -7.00; 6.86] | Low | Very low | Not serious | Very low | -0.07 [ -7.00; 6.86] | Very low | . | Serious | Very low | NMA estimate was used because incoherence was not available. Imprecision was rated as Serious. |
| Compound Mylabris preparations+OX | Yangzheng Xiaoji Capsules+OX | 0 | . | . | . |  |  |  |  |  | 0.06 [ -8.71; 8.83] | Low | High | Not serious | Low | 0.06 [ -8.71; 8.83] | Low | . | Serious | Very low | NMA estimate was used because incoherence was not available. Imprecision was rated as Serious. |
| Compound Mylabris preparations+OX | Zhenqi Fuzheng Granules+OX | 0 | . | . | . |  |  |  |  |  | -3.93 [-12.80; 4.94] | Low | Low | Not serious | Low | -3.93 [-12.80; 4.94] | Low | . | Serious | Very low | NMA estimate was used because incoherence was not available. Imprecision was rated as Serious. |
| Diyu Shengbai Tablet+OX | Ginseng Polysaccharide Injection+OX | 0 | . | . | . |  |  |  |  |  | 4.67 [ -6.71; 16.05] | Low | Low | Not serious | Low | 4.67 [ -6.71; 16.05] | Low | . | Serious | Very low | NMA estimate was used because incoherence was not available. Imprecision was rated as Serious. |
| Diyu Shengbai Tablet+OX | Huachansu preparations+OX | 0 | . | . | . |  |  |  |  |  | 3.02 [ -5.46; 11.51] | Low | High | Not serious | Low | 3.02 [ -5.46; 11.51] | Low | . | Serious | Very low | NMA estimate was used because incoherence was not available. Imprecision was rated as Serious. |
| Diyu Shengbai Tablet+OX | Jinlong Capsules+OX | 0 | . | . | . |  |  |  |  |  | 4.07 [ -4.70; 12.83] | Low | Very low | Not serious | Very low | 4.07 [ -4.70; 12.83] | Very low | . | Serious | Very low | NMA estimate was used because incoherence was not available. Imprecision was rated as Serious. |
| Diyu Shengbai Tablet+OX | Kangai Injection+OX | 0 | . | . | . |  |  |  |  |  | 0.92 [ -7.72; 9.57] | Low | Very low | Not serious | Very low | 0.92 [ -7.72; 9.57] | Very low | . | Serious | Very low | NMA estimate was used because incoherence was not available. Imprecision was rated as Serious. |
| Diyu Shengbai Tablet+OX | Kanglixin Capsules+OX | 0 | . | . | . |  |  |  |  |  | 1.34 [ -8.17; 10.85] | Low | Very low | Not serious | Very low | 1.34 [ -8.17; 10.85] | Very low | . | Serious | Very low | NMA estimate was used because incoherence was not available. Imprecision was rated as Serious. |
| Diyu Shengbai Tablet+OX | Lentinan+OX | 0 | . | . | . |  |  |  |  |  | 0.07 [ -9.49; 9.62] | Low | High | Not serious | Low | 0.07 [ -9.49; 9.62] | Low | . | Serious | Very low | NMA estimate was used because incoherence was not available. Imprecision was rated as Serious. |
| Diyu Shengbai Tablet+OX | OX | 1 | 101 | . | 9.50 [ 1.79; 17.21] | Very serious | Not serious | Not serious | Undetected | Low | . |  |  |  |  | 9.50 [ 1.79; 17.21] | Low | . | Not serious | Low | NMA estimate was used because incoherence was not available. Imprecision was rated as Serious. |
| Diyu Shengbai Tablet+OX | Pingxiao Capsules+OX | 0 | . | . | . |  |  |  |  |  | 2.27 [ -8.75; 13.29] | Low | Low | Not serious | Low | 2.27 [ -8.75; 13.29] | Low | . | Serious | Very low | NMA estimate was used because incoherence was not available. Imprecision was rated as Serious. |
| Diyu Shengbai Tablet+OX | Shenmai Injection+OX | 0 | . | . | . |  |  |  |  |  | 1.17 [ -9.72; 12.06] | Low | High | Not serious | Low | 1.17 [ -9.72; 12.06] | Low | . | Serious | Very low | NMA estimate was used because incoherence was not available. Imprecision was rated as Serious. |
| Diyu Shengbai Tablet+OX | Shenqi Fuzheng Injection+OX | 0 | . | . | . |  |  |  |  |  | 3.78 [ -4.20; 11.75] | Low | Moderate | Not serious | Low | 3.78 [ -4.20; 11.75] | Low | . | Serious | Very low | NMA estimate was used because incoherence was not available. Imprecision was rated as Serious. |
| Diyu Shengbai Tablet+OX | Xiaoaiping Injection+OX | 0 | . | . | . |  |  |  |  |  | -0.34 [ -9.83; 9.15] | Low | Very low | Not serious | Very low | -0.34 [ -9.83; 9.15] | Very low | . | Serious | Very low | NMA estimate was used because incoherence was not available. Imprecision was rated as Serious. |
| Diyu Shengbai Tablet+OX | Xihuang Capsules+OX | 0 | . | . | . |  |  |  |  |  | 5.06 [ -4.46; 14.57] | Low | Low | Not serious | Low | 5.06 [ -4.46; 14.57] | Low | . | Serious | Very low | NMA estimate was used because incoherence was not available. Imprecision was rated as Serious. |
| Diyu Shengbai Tablet+OX | Ya Dan Zi Oil Emulsion Injection+OX | 0 | . | . | . |  |  |  |  |  | 4.26 [ -5.34; 13.86] | Low | Very low | Not serious | Very low | 4.26 [ -5.34; 13.86] | Very low | . | Serious | Very low | NMA estimate was used because incoherence was not available. Imprecision was rated as Serious. |
| Diyu Shengbai Tablet+OX | Yangzheng Xiaoji Capsules+OX | 0 | . | . | . |  |  |  |  |  | 4.39 [ -6.61; 15.39] | Low | High | Not serious | Low | 4.39 [ -6.61; 15.39] | Low | . | Serious | Very low | NMA estimate was used because incoherence was not available. Imprecision was rated as Serious. |
| Diyu Shengbai Tablet+OX | Zhenqi Fuzheng Granules+OX | 0 | . | . | . |  |  |  |  |  | 0.40 [-10.68; 11.48] | Low | Low | Not serious | Low | 0.40 [-10.68; 11.48] | Low | . | Serious | Very low | NMA estimate was used because incoherence was not available. Imprecision was rated as Serious. |
| Ginseng Polysaccharide Injection+OX | Huachansu preparations+OX | 0 | . | . | . |  |  |  |  |  | -1.65 [-10.73; 7.44] | Low | High | Not serious | Low | -1.65 [-10.73; 7.44] | Low | . | Serious | Very low | NMA estimate was used because incoherence was not available. Imprecision was rated as Serious. |
| Ginseng Polysaccharide Injection+OX | Jinlong Capsules+OX | 0 | . | . | . |  |  |  |  |  | -0.60 [ -9.96; 8.75] | Low | Very low | Not serious | Very low | -0.60 [ -9.96; 8.75] | Very low | . | Serious | Very low | NMA estimate was used because incoherence was not available. Imprecision was rated as Serious. |
| Ginseng Polysaccharide Injection+OX | Kangai Injection+OX | 0 | . | . | . |  |  |  |  |  | -3.75 [-12.99; 5.49] | Low | Very low | Not serious | Very low | -3.75 [-12.99; 5.49] | Very low | . | Serious | Very low | NMA estimate was used because incoherence was not available. Imprecision was rated as Serious. |
| Ginseng Polysaccharide Injection+OX | Kanglixin Capsules+OX | 0 | . | . | . |  |  |  |  |  | -3.33 [-13.38; 6.73] | Low | Very low | Not serious | Very low | -3.33 [-13.38; 6.73] | Very low | . | Serious | Very low | NMA estimate was used because incoherence was not available. Imprecision was rated as Serious. |
| Ginseng Polysaccharide Injection+OX | Lentinan+OX | 0 | . | . | . |  |  |  |  |  | -4.60 [-14.70; 5.50] | Low | High | Not serious | Low | -4.60 [-14.70; 5.50] | Low | . | Serious | Very low | NMA estimate was used because incoherence was not available. Imprecision was rated as Serious. |
| Ginseng Polysaccharide Injection+OX | OX | 1 | 68 | . | 4.83 [-3.54; 13.20] | Very serious | Not serious | Not serious | Undetected | Low | . |  |  |  |  | 4.83 [ -3.54; 13.20] | Low | . | Serious | Very low | NMA estimate was used because incoherence was not available. Imprecision was rated as Serious. |
| Ginseng Polysaccharide Injection+OX | Pingxiao Capsules+OX | 0 | . | . | . |  |  |  |  |  | -2.40 [-13.89; 9.09] | Low | Low | Not serious | Low | -2.40 [-13.89; 9.09] | Low | . | Serious | Very low | NMA estimate was used because incoherence was not available. Imprecision was rated as Serious. |
| Ginseng Polysaccharide Injection+OX | Shenmai Injection+OX | 0 | . | . | . |  |  |  |  |  | -3.50 [-14.87; 7.87] | Low | High | Not serious | Low | -3.50 [-14.87; 7.87] | Low | . | Serious | Very low | NMA estimate was used because incoherence was not available. Imprecision was rated as Serious. |
| Ginseng Polysaccharide Injection+OX | Shenqi Fuzheng Injection+OX | 0 | . | . | . |  |  |  |  |  | -0.89 [ -9.51; 7.72] | Low | Moderate | Not serious | Low | -0.89 [ -9.51; 7.72] | Low | . | Serious | Very low | NMA estimate was used because incoherence was not available. Imprecision was rated as Serious. |
| Ginseng Polysaccharide Injection+OX | Xiaoaiping Injection+OX | 0 | . | . | . |  |  |  |  |  | -5.01 [-15.04; 5.02] | Low | Very low | Not serious | Very low | -5.01 [-15.04; 5.02] | Very low | . | Serious | Very low | NMA estimate was used because incoherence was not available. Imprecision was rated as Serious. |
| Ginseng Polysaccharide Injection+OX | Xihuang Capsules+OX | 0 | . | . | . |  |  |  |  |  | 0.39 [ -9.68; 10.45] | Low | Low | Not serious | Low | 0.39 [ -9.68; 10.45] | Low | . | Serious | Very low | NMA estimate was used because incoherence was not available. Imprecision was rated as Serious. |
| Ginseng Polysaccharide Injection+OX | Ya Dan Zi Oil Emulsion Injection+OX | 0 | . | . | . |  |  |  |  |  | -0.41 [-10.54; 9.73] | Low | Very low | Not serious | Very low | -0.41 [-10.54; 9.73] | Very low | . | Serious | Very low | NMA estimate was used because incoherence was not available. Imprecision was rated as Serious. |
| Ginseng Polysaccharide Injection+OX | Yangzheng Xiaoji Capsules+OX | 0 | . | . | . |  |  |  |  |  | -0.28 [-11.75; 11.19] | Low | High | Not serious | Low | -0.28 [-11.75; 11.19] | Low | . | Serious | Very low | NMA estimate was used because incoherence was not available. Imprecision was rated as Serious. |
| Ginseng Polysaccharide Injection+OX | Zhenqi Fuzheng Granules+OX | 0 | . | . | . |  |  |  |  |  | -4.27 [-15.82; 7.28] | Low | Low | Not serious | Low | -4.27 [-15.82; 7.28] | Low | . | Serious | Very low | NMA estimate was used because incoherence was not available. Imprecision was rated as Serious. |
| Huachansu preparations+OX | Jinlong Capsules+OX | 0 | . | . | . |  |  |  |  |  | 1.04 [ -4.43; 6.52] | High | Very low | Not serious | Very low | 1.04 [ -4.43; 6.52] | Very low | . | Serious | Very low | NMA estimate was used because incoherence was not available. Imprecision was rated as Serious. |
| Huachansu preparations+OX | Kangai Injection+OX | 0 | . | . | . |  |  |  |  |  | -2.10 [ -7.38; 3.18] | High | Very low | Not serious | Very low | -2.10 [ -7.38; 3.18] | Very low | . | Serious | Very low | NMA estimate was used because incoherence was not available. Imprecision was rated as Serious. |
| Huachansu preparations+OX | Kanglixin Capsules+OX | 0 | . | . | . |  |  |  |  |  | -1.68 [ -8.28; 4.92] | High | Very low | Not serious | Very low | -1.68 [ -8.28; 4.92] | Very low | . | Serious | Very low | NMA estimate was used because incoherence was not available. Imprecision was rated as Serious. |
| Huachansu preparations+OX | Lentinan+OX | 0 | . | . | . |  |  |  |  |  | -2.96 [ -9.62; 3.71] | High | High | Not serious | High | -2.96 [ -9.62; 3.71] | High | . | Serious | Moderate | NMA estimate was used because incoherence was not available. Imprecision was rated as Serious. |
| Huachansu preparations+OX | OX | 5 | 347 | 62.7% | 6.48 [ 2.93; 10.02] | Not serious | Not serious | Not serious | Undetected | High | . |  |  |  |  | 6.48 [ 2.93; 10.02] | High | . | Not serious | High | NMA estimate was used because incoherence was not available. Imprecision was rated as Serious. |
| Huachansu preparations+OX | Pingxiao Capsules+OX | 0 | . | . | . |  |  |  |  |  | -0.75 [ -9.38; 7.88] | High | Low | Not serious | Low | -0.75 [ -9.38; 7.88] | Low | . | Serious | Very low | NMA estimate was used because incoherence was not available. Imprecision was rated as Serious. |
| Huachansu preparations+OX | Shenmai Injection+OX | 0 | . | . | . |  |  |  |  |  | -1.85 [-10.32; 6.62] | High | High | Not serious | High | -1.85 [-10.32; 6.62] | High | . | Serious | Moderate | NMA estimate was used because incoherence was not available. Imprecision was rated as Serious. |
| Huachansu preparations+OX | Shenqi Fuzheng Injection+OX | 0 | . | . | . |  |  |  |  |  | 0.75 [ -3.33; 4.84] | High | Moderate | Not serious | Moderate | 0.75 [ -3.33; 4.84] | Moderate | . | Serious | Low | NMA estimate was used because incoherence was not available. Imprecision was rated as Serious. |
| Huachansu preparations+OX | Xiaoaiping Injection+OX | 0 | . | . | . |  |  |  |  |  | -3.36 [ -9.93; 3.20] | High | Very low | Not serious | Very low | -3.36 [ -9.93; 3.20] | Very low | . | Serious | Very low | NMA estimate was used because incoherence was not available. Imprecision was rated as Serious. |
| Huachansu preparations+OX | Xihuang Capsules+OX | 0 | . | . | . |  |  |  |  |  | 2.03 [ -4.58; 8.64] | High | Low | Not serious | Low | 2.03 [ -4.58; 8.64] | Low | . | Serious | Very low | NMA estimate was used because incoherence was not available. Imprecision was rated as Serious. |
| Huachansu preparations+OX | Ya Dan Zi Oil Emulsion Injection+OX | 0 | . | . | . |  |  |  |  |  | 1.24 [ -5.49; 7.97] | High | Very low | Not serious | Very low | 1.24 [ -5.49; 7.97] | Very low | . | Serious | Very low | NMA estimate was used because incoherence was not available. Imprecision was rated as Serious. |
| Huachansu preparations+OX | Yangzheng Xiaoji Capsules+OX | 0 | . | . | . |  |  |  |  |  | 1.37 [ -7.25; 9.98] | High | High | Not serious | High | 1.37 [ -7.25; 9.98] | High | . | Serious | Moderate | NMA estimate was used because incoherence was not available. Imprecision was rated as Serious. |
| Huachansu preparations+OX | Zhenqi Fuzheng Granules+OX | 0 | . | . | . |  |  |  |  |  | -2.62 [-11.34; 6.09] | High | Low | Not serious | Low | -2.62 [-11.34; 6.09] | Low | . | Serious | Very low | NMA estimate was used because incoherence was not available. Imprecision was rated as Serious. |
| Jinlong Capsules+OX | Kangai Injection+OX | 0 | . | . | . |  |  |  |  |  | -3.14 [ -8.86; 2.58] | Very low | Very low | Not serious | Very low | -3.14 [ -8.86; 2.58] | Very low | . | Serious | Very low | NMA estimate was used because incoherence was not available. Imprecision was rated as Serious. |
| Jinlong Capsules+OX | Kanglixin Capsules+OX | 0 | . | . | . |  |  |  |  |  | -2.72 [ -9.68; 4.24] | Very low | Very low | Not serious | Very low | -2.72 [ -9.68; 4.24] | Very low | . | Serious | Very low | NMA estimate was used because incoherence was not available. Imprecision was rated as Serious. |
| Jinlong Capsules+OX | Lentinan+OX | 0 | . | . | . |  |  |  |  |  | -4.00 [-11.02; 3.03] | Very low | High | Not serious | Very low | -4.00 [-11.02; 3.03] | Very low | . | Serious | Very low | NMA estimate was used because incoherence was not available. Imprecision was rated as Serious. |
| Jinlong Capsules+OX | OX | 4 | 287 | 90.5% | 5.43 [ 1.26; 9.61] | Serious | Very serious | Not serious | Undetected | Very low | . |  |  |  |  | 5.43 [ 1.26; 9.61] | Very low | . | Not serious | Very low | NMA estimate was used because incoherence was not available. Imprecision was rated as Serious. |
| Jinlong Capsules+OX | Pingxiao Capsules+OX | 0 | . | . | . |  |  |  |  |  | -1.80 [-10.70; 7.11] | Very low | Low | Not serious | Very low | -1.80 [-10.70; 7.11] | Very low | . | Serious | Very low | NMA estimate was used because incoherence was not available. Imprecision was rated as Serious. |
| Jinlong Capsules+OX | Shenmai Injection+OX | 0 | . | . | . |  |  |  |  |  | -2.90 [-11.65; 5.86] | Very low | High | Not serious | Very low | -2.90 [-11.65; 5.86] | Very low | . | Serious | Very low | NMA estimate was used because incoherence was not available. Imprecision was rated as Serious. |
| Jinlong Capsules+OX | Shenqi Fuzheng Injection+OX | 0 | . | . | . |  |  |  |  |  | -0.29 [ -4.94; 4.36] | Very low | Moderate | Not serious | Very low | -0.29 [ -4.94; 4.36] | Very low | . | Serious | Very low | NMA estimate was used because incoherence was not available. Imprecision was rated as Serious. |
| Jinlong Capsules+OX | Xiaoaiping Injection+OX | 0 | . | . | . |  |  |  |  |  | -4.40 [-11.33; 2.53] | Very low | Very low | Not serious | Very low | -4.40 [-11.33; 2.53] | Very low | . | Serious | Very low | NMA estimate was used because incoherence was not available. Imprecision was rated as Serious. |
| Jinlong Capsules+OX | Xihuang Capsules+OX | 0 | . | . | . |  |  |  |  |  | 0.99 [ -5.98; 7.96] | Very low | Low | Not serious | Very low | 0.99 [ -5.98; 7.96] | Very low | . | Serious | Very low | NMA estimate was used because incoherence was not available. Imprecision was rated as Serious. |
| Jinlong Capsules+OX | Ya Dan Zi Oil Emulsion Injection+OX | 0 | . | . | . |  |  |  |  |  | 0.20 [ -6.88; 7.28] | Very low | Very low | Not serious | Very low | 0.20 [ -6.88; 7.28] | Very low | . | Serious | Very low | NMA estimate was used because incoherence was not available. Imprecision was rated as Serious. |
| Jinlong Capsules+OX | Yangzheng Xiaoji Capsules+OX | 0 | . | . | . |  |  |  |  |  | 0.32 [ -8.57; 9.21] | Very low | High | Not serious | Very low | 0.32 [ -8.57; 9.21] | Very low | . | Serious | Very low | NMA estimate was used because incoherence was not available. Imprecision was rated as Serious. |
| Jinlong Capsules+OX | Zhenqi Fuzheng Granules+OX | 0 | . | . | . |  |  |  |  |  | -3.67 [-12.65; 5.32] | Very low | Low | Not serious | Very low | -3.67 [-12.65; 5.32] | Very low | . | Serious | Very low | NMA estimate was used because incoherence was not available. Imprecision was rated as Serious. |
| Kangai Injection+OX | Kanglixin Capsules+OX | 0 | . | . | . |  |  |  |  |  | 0.42 [ -6.39; 7.23] | Very low | Very low | Not serious | Very low | 0.42 [ -6.39; 7.23] | Very low | . | Serious | Very low | NMA estimate was used because incoherence was not available. Imprecision was rated as Serious. |
| Kangai Injection+OX | Lentinan+OX | 0 | . | . | . |  |  |  |  |  | -0.85 [ -7.73; 6.02] | Very low | High | Not serious | Very low | -0.85 [ -7.73; 6.02] | Very low | . | Serious | Very low | NMA estimate was used because incoherence was not available. Imprecision was rated as Serious. |
| Kangai Injection+OX | OX | 4 | 289 | 96.9% | 8.58 [ 4.66; 12.49] | Serious | Very serious | Not serious | Undetected | Very low | . |  |  |  |  | 8.58 [ 4.66; 12.49] | Very low | . | Not serious | Very low | NMA estimate was used because incoherence was not available. Imprecision was rated as Serious. |
| Kangai Injection+OX | Pingxiao Capsules+OX | 0 | . | . | . |  |  |  |  |  | 1.35 [ -7.44; 10.13] | Very low | Low | Not serious | Very low | 1.35 [ -7.44; 10.13] | Very low | . | Serious | Very low | NMA estimate was used because incoherence was not available. Imprecision was rated as Serious. |
| Kangai Injection+OX | Shenmai Injection+OX | 0 | . | . | . |  |  |  |  |  | 0.25 [ -8.39; 8.88] | Very low | High | Not serious | Very low | 0.25 [ -8.39; 8.88] | Very low | . | Serious | Very low | NMA estimate was used because incoherence was not available. Imprecision was rated as Serious. |
| Kangai Injection+OX | Shenqi Fuzheng Injection+OX | 0 | . | . | . |  |  |  |  |  | 2.85 [ -1.56; 7.27] | Very low | Moderate | Not serious | Very low | 2.85 [ -1.56; 7.27] | Very low | . | Serious | Very low | NMA estimate was used because incoherence was not available. Imprecision was rated as Serious. |
| Kangai Injection+OX | Xiaoaiping Injection+OX | 0 | . | . | . |  |  |  |  |  | -1.26 [ -8.04; 5.51] | Very low | Very low | Not serious | Very low | -1.26 [ -8.04; 5.51] | Very low | . | Serious | Very low | NMA estimate was used because incoherence was not available. Imprecision was rated as Serious. |
| Kangai Injection+OX | Xihuang Capsules+OX | 0 | . | . | . |  |  |  |  |  | 4.13 [ -2.69; 10.95] | Very low | Low | Not serious | Very low | 4.13 [ -2.69; 10.95] | Very low | . | Serious | Very low | NMA estimate was used because incoherence was not available. Imprecision was rated as Serious. |
| Kangai Injection+OX | Ya Dan Zi Oil Emulsion Injection+OX | 0 | . | . | . |  |  |  |  |  | 3.34 [ -3.59; 10.27] | Very low | Very low | Not serious | Very low | 3.34 [ -3.59; 10.27] | Very low | . | Serious | Very low | NMA estimate was used because incoherence was not available. Imprecision was rated as Serious. |
| Kangai Injection+OX | Yangzheng Xiaoji Capsules+OX | 0 | . | . | . |  |  |  |  |  | 3.47 [ -5.30; 12.24] | Very low | High | Not serious | Very low | 3.47 [ -5.30; 12.24] | Very low | . | Serious | Very low | NMA estimate was used because incoherence was not available. Imprecision was rated as Serious. |
| Kangai Injection+OX | Zhenqi Fuzheng Granules+OX | 0 | . | . | . |  |  |  |  |  | -0.52 [ -9.39; 8.35] | Very low | Low | Not serious | Very low | -0.52 [ -9.39; 8.35] | Very low | . | Serious | Very low | NMA estimate was used because incoherence was not available. Imprecision was rated as Serious. |
| Kanglixin Capsules+OX | Lentinan+OX | 0 | . | . | . |  |  |  |  |  | -1.27 [ -9.21; 6.66] | Very low | High | Not serious | Very low | -1.27 [ -9.21; 6.66] | Very low | . | Serious | Very low | NMA estimate was used because incoherence was not available. Imprecision was rated as Serious. |
| Kanglixin Capsules+OX | OX | 2 | 140 | 90.6% | 8.16 [ 2.59; 13.73] | Very serious | Very serious | Not serious | Undetected | Very low | . |  |  |  |  | 8.16 [ 2.59; 13.73] | Very low | . | Not serious | Very low | NMA estimate was used because incoherence was not available. Imprecision was rated as Serious. |
| Kanglixin Capsules+OX | Pingxiao Capsules+OX | 0 | . | . | . |  |  |  |  |  | 0.93 [ -8.71; 10.57] | Very low | Low | Not serious | Very low | 0.93 [ -8.71; 10.57] | Very low | . | Serious | Very low | NMA estimate was used because incoherence was not available. Imprecision was rated as Serious. |
| Kanglixin Capsules+OX | Shenmai Injection+OX | 0 | . | . | . |  |  |  |  |  | -0.17 [ -9.67; 9.32] | Very low | High | Not serious | Very low | -0.17 [ -9.67; 9.32] | Very low | . | Serious | Very low | NMA estimate was used because incoherence was not available. Imprecision was rated as Serious. |
| Kanglixin Capsules+OX | Shenqi Fuzheng Injection+OX | 0 | . | . | . |  |  |  |  |  | 2.43 [ -3.50; 8.36] | Very low | Moderate | Not serious | Very low | 2.43 [ -3.50; 8.36] | Very low | . | Serious | Very low | NMA estimate was used because incoherence was not available. Imprecision was rated as Serious. |
| Kanglixin Capsules+OX | Xiaoaiping Injection+OX | 0 | . | . | . |  |  |  |  |  | -1.68 [ -9.53; 6.17] | Very low | Very low | Not serious | Very low | -1.68 [ -9.53; 6.17] | Very low | . | Serious | Very low | NMA estimate was used because incoherence was not available. Imprecision was rated as Serious. |
| Kanglixin Capsules+OX | Xihuang Capsules+OX | 0 | . | . | . |  |  |  |  |  | 3.71 [ -4.17; 11.60] | Very low | Low | Not serious | Very low | 3.71 [ -4.17; 11.60] | Very low | . | Serious | Very low | NMA estimate was used because incoherence was not available. Imprecision was rated as Serious. |
| Kanglixin Capsules+OX | Ya Dan Zi Oil Emulsion Injection+OX | 0 | . | . | . |  |  |  |  |  | 2.92 [ -5.06; 10.90] | Very low | Very low | Not serious | Very low | 2.92 [ -5.06; 10.90] | Very low | . | Serious | Very low | NMA estimate was used because incoherence was not available. Imprecision was rated as Serious. |
| Kanglixin Capsules+OX | Yangzheng Xiaoji Capsules+OX | 0 | . | . | . |  |  |  |  |  | 3.05 [ -6.58; 12.67] | Very low | High | Not serious | Very low | 3.05 [ -6.58; 12.67] | Very low | . | Serious | Very low | NMA estimate was used because incoherence was not available. Imprecision was rated as Serious. |
| Kanglixin Capsules+OX | Zhenqi Fuzheng Granules+OX | 0 | . | . | . |  |  |  |  |  | -0.94 [-10.66; 8.77] | Very low | Low | Not serious | Very low | -0.94 [-10.66; 8.77] | Very low | . | Serious | Very low | NMA estimate was used because incoherence was not available. Imprecision was rated as Serious. |
| Lentinan+OX | OX | 2 | 206 | 73.1% | 9.43 [ 3.78; 15.08] | Not serious | Not serious | Not serious | Undetected | High | . |  |  |  |  | 9.43 [ 3.78; 15.08] | High | . | Not serious | High | NMA estimate was used because incoherence was not available. Imprecision was rated as Serious. |
| Lentinan+OX | Pingxiao Capsules+OX | 0 | . | . | . |  |  |  |  |  | 2.20 [ -7.48; 11.89] | High | Low | Not serious | Low | 2.20 [ -7.48; 11.89] | Low | . | Serious | Very low | NMA estimate was used because incoherence was not available. Imprecision was rated as Serious. |
| Lentinan+OX | Shenmai Injection+OX | 0 | . | . | . |  |  |  |  |  | 1.10 [ -8.44; 10.65] | High | High | Not serious | High | 1.10 [ -8.44; 10.65] | High | . | Serious | Moderate | NMA estimate was used because incoherence was not available. Imprecision was rated as Serious. |
| Lentinan+OX | Shenqi Fuzheng Injection+OX | 0 | . | . | . |  |  |  |  |  | 3.71 [ -2.30; 9.71] | High | Moderate | Not serious | Moderate | 3.71 [ -2.30; 9.71] | Moderate | . | Serious | Low | NMA estimate was used because incoherence was not available. Imprecision was rated as Serious. |
| Lentinan+OX | Xiaoaiping Injection+OX | 0 | . | . | . |  |  |  |  |  | -0.41 [ -8.31; 7.50] | High | Very low | Not serious | Very low | -0.41 [ -8.31; 7.50] | Very low | . | Serious | Very low | NMA estimate was used because incoherence was not available. Imprecision was rated as Serious. |
| Lentinan+OX | Xihuang Capsules+OX | 0 | . | . | . |  |  |  |  |  | 4.99 [ -2.96; 12.93] | High | Low | Not serious | Low | 4.99 [ -2.96; 12.93] | Low | . | Serious | Very low | NMA estimate was used because incoherence was not available. Imprecision was rated as Serious. |
| Lentinan+OX | Ya Dan Zi Oil Emulsion Injection+OX | 0 | . | . | . |  |  |  |  |  | 4.19 [ -3.84; 12.23] | High | Very low | Not serious | Very low | 4.19 [ -3.84; 12.23] | Very low | . | Serious | Very low | NMA estimate was used because incoherence was not available. Imprecision was rated as Serious. |
| Lentinan+OX | Yangzheng Xiaoji Capsules+OX | 0 | . | . | . |  |  |  |  |  | 4.32 [ -5.35; 13.99] | High | High | Not serious | High | 4.32 [ -5.35; 13.99] | High | . | Serious | Moderate | NMA estimate was used because incoherence was not available. Imprecision was rated as Serious. |
| Lentinan+OX | Zhenqi Fuzheng Granules+OX | 0 | . | . | . |  |  |  |  |  | 0.33 [ -9.43; 10.09] | High | Low | Not serious | Low | 0.33 [ -9.43; 10.09] | Low | . | Serious | Very low | NMA estimate was used because incoherence was not available. Imprecision was rated as Serious. |
| Pingxiao Capsules+OX | OX | 1 | 86 | . | 7.23 [-0.64; 15.10] | Very serious | Not serious | Not serious | Undetected | Low | . |  |  |  |  | 7.23 [ -0.64; 15.10] | Low | . | Serious | Very low | NMA estimate was used because incoherence was not available. Imprecision was rated as Serious. |
| Shenmai Injection+OX | OX | 1 | 82 | . | 8.33 [ 0.64; 16.02] | Not serious | Not serious | Not serious | Undetected | High | . |  |  |  |  | 8.33 [ 0.64; 16.02] | High | . | Not serious | High | NMA estimate was used because incoherence was not available. Imprecision was rated as Serious. |
| Shenqi Fuzheng Injection+OX | OX | 15 | 1345 | 86.9% | 5.72 [ 3.68; 7.76] | Serious | Not serious | Not serious | Not serious | Moderate | . |  |  |  |  | 5.72 [ 3.68; 7.76] | Moderate | . | Not serious | Moderate | NMA estimate was used because incoherence was not available. Imprecision was rated as Not serious. |
| Xiaoaiping Injection+OX | OX | 2 | 384 | 98.9% | 9.84 [ 4.31; 15.37] | Very serious | Very serious | Not serious | Undetected | Very low | . |  |  |  |  | 9.84 [ 4.31; 15.37] | Very low | . | Not serious | Very low | NMA estimate was used because incoherence was not available. Imprecision was rated as Serious. |
| Xihuang Capsules+OX | OX | 2 | 164 | 0.0% | 4.44 [-1.14; 10.03] | Very serious | Not serious | Not serious | Undetected | Low | . |  |  |  |  | 4.44 [ -1.14; 10.03] | Low | . | Serious | Very low | NMA estimate was used because incoherence was not available. Imprecision was rated as Serious. |
| Ya Dan Zi Oil Emulsion Injection+OX | OX | 2 | 204 | 98.5% | 5.24 [-0.48; 10.96] | Serious | Very serious | Not serious | Undetected | Very low | . |  |  |  |  | 5.24 [ -0.48; 10.96] | Very low | . | Serious | Very low | NMA estimate was used because incoherence was not available. Imprecision was rated as Serious. |
| Yangzheng Xiaoji Capsules+OX | OX | 1 | 80 | . | 5.11 [-2.74; 12.96] | Not serious | Not serious | Not serious | Undetected | High | . |  |  |  |  | 5.11 [ -2.74; 12.96] | High | . | Serious | Moderate | NMA estimate was used because incoherence was not available. Imprecision was rated as Serious. |
| Zhenqi Fuzheng Granules+OX | OX | 1 | 92 | . | 9.10 [ 1.14; 17.06] | Very serious | Not serious | Not serious | Undetected | Low | . |  |  |  |  | 9.10 [ 1.14; 17.06] | Low | . | Not serious | Low | NMA estimate was used because incoherence was not available. Imprecision was rated as Serious. |
| Pingxiao Capsules+OX | Shenmai Injection+OX | 0 | . | . | . |  |  |  |  |  | -1.10 [-12.11; 9.91] | Low | High | Not serious | Low | -1.10 [-12.11; 9.91] | Low | . | Serious | Very low | NMA estimate was used because incoherence was not available. Imprecision was rated as Serious. |
| Pingxiao Capsules+OX | Shenqi Fuzheng Injection+OX | 0 | . | . | . |  |  |  |  |  | 1.51 [ -6.62; 9.64] | Low | Moderate | Not serious | Low | 1.51 [ -6.62; 9.64] | Low | . | Serious | Very low | NMA estimate was used because incoherence was not available. Imprecision was rated as Serious. |
| Pingxiao Capsules+OX | Xiaoaiping Injection+OX | 0 | . | . | . |  |  |  |  |  | -2.61 [-12.23; 7.01] | Low | Very low | Not serious | Very low | -2.61 [-12.23; 7.01] | Very low | . | Serious | Very low | NMA estimate was used because incoherence was not available. Imprecision was rated as Serious. |
| Pingxiao Capsules+OX | Xihuang Capsules+OX | 0 | . | . | . |  |  |  |  |  | 2.79 [ -6.86; 12.43] | Low | Low | Not serious | Low | 2.79 [ -6.86; 12.43] | Low | . | Serious | Very low | NMA estimate was used because incoherence was not available. Imprecision was rated as Serious. |
| Pingxiao Capsules+OX | Ya Dan Zi Oil Emulsion Injection+OX | 0 | . | . | . |  |  |  |  |  | 1.99 [ -7.73; 11.72] | Low | Very low | Not serious | Very low | 1.99 [ -7.73; 11.72] | Very low | . | Serious | Very low | NMA estimate was used because incoherence was not available. Imprecision was rated as Serious. |
| Pingxiao Capsules+OX | Yangzheng Xiaoji Capsules+OX | 0 | . | . | . |  |  |  |  |  | 2.12 [ -8.99; 13.23] | Low | High | Not serious | Low | 2.12 [ -8.99; 13.23] | Low | . | Serious | Very low | NMA estimate was used because incoherence was not available. Imprecision was rated as Serious. |
| Pingxiao Capsules+OX | Zhenqi Fuzheng Granules+OX | 0 | . | . | . |  |  |  |  |  | -1.87 [-13.06; 9.32] | Low | Low | Not serious | Low | -1.87 [-13.06; 9.32] | Low | . | Serious | Very low | NMA estimate was used because incoherence was not available. Imprecision was rated as Serious. |
| Shenmai Injection+OX | Shenqi Fuzheng Injection+OX | 0 | . | . | . |  |  |  |  |  | 2.61 [ -5.35; 10.57] | High | Moderate | Not serious | Moderate | 2.61 [ -5.35; 10.57] | Moderate | . | Serious | Low | NMA estimate was used because incoherence was not available. Imprecision was rated as Serious. |
| Shenmai Injection+OX | Xiaoaiping Injection+OX | 0 | . | . | . |  |  |  |  |  | -1.51 [-10.98; 7.97] | High | Very low | Not serious | Very low | -1.51 [-10.98; 7.97] | Very low | . | Serious | Very low | NMA estimate was used because incoherence was not available. Imprecision was rated as Serious. |
| Shenmai Injection+OX | Xihuang Capsules+OX | 0 | . | . | . |  |  |  |  |  | 3.89 [ -5.62; 13.39] | High | Low | Not serious | Low | 3.89 [ -5.62; 13.39] | Low | . | Serious | Very low | NMA estimate was used because incoherence was not available. Imprecision was rated as Serious. |
| Shenmai Injection+OX | Ya Dan Zi Oil Emulsion Injection+OX | 0 | . | . | . |  |  |  |  |  | 3.09 [ -6.49; 12.68] | High | Very low | Not serious | Very low | 3.09 [ -6.49; 12.68] | Very low | . | Serious | Very low | NMA estimate was used because incoherence was not available. Imprecision was rated as Serious. |
| Shenmai Injection+OX | Yangzheng Xiaoji Capsules+OX | 0 | . | . | . |  |  |  |  |  | 3.22 [ -7.77; 14.21] | High | High | Not serious | High | 3.22 [ -7.77; 14.21] | High | . | Serious | Moderate | NMA estimate was used because incoherence was not available. Imprecision was rated as Serious. |
| Shenmai Injection+OX | Zhenqi Fuzheng Granules+OX | 0 | . | . | . |  |  |  |  |  | -0.77 [-11.84; 10.30] | High | Low | Not serious | Low | -0.77 [-11.84; 10.30] | Low | . | Serious | Very low | NMA estimate was used because incoherence was not available. Imprecision was rated as Serious. |
| Shenqi Fuzheng Injection+OX | Xiaoaiping Injection+OX | 0 | . | . | . |  |  |  |  |  | -4.12 [-10.01; 1.78] | Moderate | Very low | Not serious | Very low | -4.12 [-10.01; 1.78] | Very low | . | Serious | Very low | NMA estimate was used because incoherence was not available. Imprecision was rated as Serious. |
| Shenqi Fuzheng Injection+OX | Xihuang Capsules+OX | 0 | . | . | . |  |  |  |  |  | 1.28 [ -4.67; 7.22] | Moderate | Low | Not serious | Low | 1.28 [ -4.67; 7.22] | Low | . | Serious | Very low | NMA estimate was used because incoherence was not available. Imprecision was rated as Serious. |
| Shenqi Fuzheng Injection+OX | Ya Dan Zi Oil Emulsion Injection+OX | 0 | . | . | . |  |  |  |  |  | 0.49 [ -5.59; 6.56] | Moderate | Very low | Not serious | Very low | 0.49 [ -5.59; 6.56] | Very low | . | Serious | Very low | NMA estimate was used because incoherence was not available. Imprecision was rated as Serious. |
| Shenqi Fuzheng Injection+OX | Yangzheng Xiaoji Capsules+OX | 0 | . | . | . |  |  |  |  |  | 0.61 [ -7.50; 8.72] | Moderate | High | Not serious | Moderate | 0.61 [ -7.50; 8.72] | Moderate | . | Serious | Low | NMA estimate was used because incoherence was not available. Imprecision was rated as Serious. |
| Shenqi Fuzheng Injection+OX | Zhenqi Fuzheng Granules+OX | 0 | . | . | . |  |  |  |  |  | -3.38 [-11.60; 4.84] | Moderate | Low | Not serious | Low | -3.38 [-11.60; 4.84] | Low | . | Serious | Very low | NMA estimate was used because incoherence was not available. Imprecision was rated as Serious. |
| Xiaoaiping Injection+OX | Xihuang Capsules+OX | 0 | . | . | . |  |  |  |  |  | 5.39 [ -2.47; 13.25] | Very low | Low | Not serious | Very low | 5.39 [ -2.47; 13.25] | Very low | . | Serious | Very low | NMA estimate was used because incoherence was not available. Imprecision was rated as Serious. |
| Xiaoaiping Injection+OX | Ya Dan Zi Oil Emulsion Injection+OX | 0 | . | . | . |  |  |  |  |  | 4.60 [ -3.35; 12.56] | Very low | Very low | Not serious | Very low | 4.60 [ -3.35; 12.56] | Very low | . | Serious | Very low | NMA estimate was used because incoherence was not available. Imprecision was rated as Serious. |
| Xiaoaiping Injection+OX | Yangzheng Xiaoji Capsules+OX | 0 | . | . | . |  |  |  |  |  | 4.73 [ -4.87; 14.33] | Very low | High | Not serious | Very low | 4.73 [ -4.87; 14.33] | Very low | . | Serious | Very low | NMA estimate was used because incoherence was not available. Imprecision was rated as Serious. |
| Xiaoaiping Injection+OX | Zhenqi Fuzheng Granules+OX | 0 | . | . | . |  |  |  |  |  | 0.74 [ -8.95; 10.43] | Very low | Low | Not serious | Very low | 0.74 [ -8.95; 10.43] | Very low | . | Serious | Very low | NMA estimate was used because incoherence was not available. Imprecision was rated as Serious. |
| Xihuang Capsules+OX | Ya Dan Zi Oil Emulsion Injection+OX | 0 | . | . | . |  |  |  |  |  | -0.79 [ -8.79; 7.20] | Low | Very low | Not serious | Very low | -0.79 [ -8.79; 7.20] | Very low | . | Serious | Very low | NMA estimate was used because incoherence was not available. Imprecision was rated as Serious. |
| Xihuang Capsules+OX | Yangzheng Xiaoji Capsules+OX | 0 | . | . | . |  |  |  |  |  | -0.67 [-10.30; 8.97] | Low | High | Not serious | Low | -0.67 [-10.30; 8.97] | Low | . | Serious | Very low | NMA estimate was used because incoherence was not available. Imprecision was rated as Serious. |
| Xihuang Capsules+OX | Zhenqi Fuzheng Granules+OX | 0 | . | . | . |  |  |  |  |  | -4.66 [-14.38; 5.07] | Low | Low | Not serious | Low | -4.66 [-14.38; 5.07] | Low | . | Serious | Very low | NMA estimate was used because incoherence was not available. Imprecision was rated as Serious. |
| Ya Dan Zi Oil Emulsion Injection+OX | Yangzheng Xiaoji Capsules+OX | 0 | . | . | . |  |  |  |  |  | 0.13 [ -9.59; 9.84] | Very low | High | Not serious | Very low | 0.13 [ -9.59; 9.84] | Very low | . | Serious | Very low | NMA estimate was used because incoherence was not available. Imprecision was rated as Serious. |
| Ya Dan Zi Oil Emulsion Injection+OX | Zhenqi Fuzheng Granules+OX | 0 | . | . | . |  |  |  |  |  | -3.86 [-13.67; 5.94] | Very low | Low | Not serious | Very low | -3.86 [-13.67; 5.94] | Very low | . | Serious | Very low | NMA estimate was used because incoherence was not available. Imprecision was rated as Serious. |
| Yangzheng Xiaoji Capsules+OX | Zhenqi Fuzheng Granules+OX | 0 | . | . | . |  |  |  |  |  | -3.99 [-15.17; 7.19] | High | Low | Not serious | Low | -3.99 [-15.17; 7.19] | Low | . | Serious | Very low | NMA estimate was used because incoherence was not available. Imprecision was rated as Serious. |

1. **CD8+**

| **Arm_1** | **Arm_2** | **No_of_study** | **Sample_size** | **I2** | **Direct_estimate** | **ROB** | **Inconsistency** | **Indirectness** | **Publication_bias** | **Direct_rating_without_imprecision** | **Indirect_estimate** | **Certainty_of_evidence_for_arm1** | **Certainty_of_evidence_for_arm2** | **Intransitivity** | **Indirect_rating_without_imprecision** | **Network_meta_analysis** | **Higher_rating_of_direct_and_indirect_without_imprecision** | **Incoherence** | **NMA_Imprecision** | **Final_network_rating** | **Final_rating_reason** |
| --- | --- | --- | --- | --- | --- | --- | --- | --- | --- | --- | --- | --- | --- | --- | --- | --- | --- | --- | --- | --- | --- |
| Aidi Injection+OX | Astragalus Polysaccharides+OX | 0 | . | . | . |  |  |  |  |  | 2.91 [ -6.17; 11.99] | Very low | Low | Not serious | Very low | 2.91 [ -6.17; 11.99] | Very low | . | Serious | Very low | NMA estimate was used because incoherence was not available. Imprecision was rated as Serious. |
| Aidi Injection+OX | Astragalus preparations+OX | 0 | . | . | . |  |  |  |  |  | 0.36 [ -7.58; 8.30] | Very low | Very low | Not serious | Very low | 0.36 [ -7.58; 8.30] | Very low | . | Serious | Very low | NMA estimate was used because incoherence was not available. Imprecision was rated as Serious. |
| Aidi Injection+OX | Compound Kushen Injection+OX | 0 | . | . | . |  |  |  |  |  | 2.57 [ -3.27; 8.40] | Very low | Very low | Not serious | Very low | 2.57 [ -3.27; 8.40] | Very low | . | Serious | Very low | NMA estimate was used because incoherence was not available. Imprecision was rated as Serious. |
| Aidi Injection+OX | Compound Mylabris preparations+OX | 0 | . | . | . |  |  |  |  |  | -0.54 [ -8.47; 7.39] | Very low | Low | Not serious | Very low | -0.54 [ -8.47; 7.39] | Very low | . | Serious | Very low | NMA estimate was used because incoherence was not available. Imprecision was rated as Serious. |
| Aidi Injection+OX | Diyu Shengbai Tablet+OX | 0 | . | . | . |  |  |  |  |  | 5.74 [ -6.05; 17.54] | Very low | Low | Not serious | Very low | 5.74 [ -6.05; 17.54] | Very low | . | Serious | Very low | NMA estimate was used because incoherence was not available. Imprecision was rated as Serious. |
| Aidi Injection+OX | Ginseng Polysaccharide Injection+OX | 0 | . | . | . |  |  |  |  |  | -1.19 [ -13.05; 10.68] | Very low | High | Not serious | Very low | -1.19 [ -13.05; 10.68] | Very low | . | Serious | Very low | NMA estimate was used because incoherence was not available. Imprecision was rated as Serious. |
| Aidi Injection+OX | Huachansu preparations+OX | 0 | . | . | . |  |  |  |  |  | -1.53 [ -8.85; 5.78] | Very low | Very low | Not serious | Very low | -1.53 [ -8.85; 5.78] | Very low | . | Serious | Very low | NMA estimate was used because incoherence was not available. Imprecision was rated as Serious. |
| Aidi Injection+OX | Jinlong Capsules+OX | 0 | . | . | . |  |  |  |  |  | 4.09 [ -3.97; 12.14] | Very low | High | Not serious | Very low | 4.09 [ -3.97; 12.14] | Very low | . | Serious | Very low | NMA estimate was used because incoherence was not available. Imprecision was rated as Serious. |
| Aidi Injection+OX | Kangai Injection+OX | 0 | . | . | . |  |  |  |  |  | -0.85 [ -8.16; 6.46] | Very low | Very low | Not serious | Very low | -0.85 [ -8.16; 6.46] | Very low | . | Serious | Very low | NMA estimate was used because incoherence was not available. Imprecision was rated as Serious. |
| Aidi Injection+OX | Kanglixin Capsules+OX | 0 | . | . | . |  |  |  |  |  | 4.93 [ -4.18; 14.04] | Very low | Low | Not serious | Very low | 4.93 [ -4.18; 14.04] | Very low | . | Serious | Very low | NMA estimate was used because incoherence was not available. Imprecision was rated as Serious. |
| Aidi Injection+OX | Lentinan+OX | 0 | . | . | . |  |  |  |  |  | 3.78 [ -8.09; 15.65] | Very low | High | Not serious | Very low | 3.78 [ -8.09; 15.65] | Very low | . | Serious | Very low | NMA estimate was used because incoherence was not available. Imprecision was rated as Serious. |
| Aidi Injection+OX | OX | 5 | 462 | 99.5% | -1.08 [ -6.01; 3.86] | Very serious | Very serious | Not serious | Undetected | Very low | . |  |  |  |  | -1.08 [ -6.01; 3.86] | Very low | . | Serious | Very low | NMA estimate was used because incoherence was not available. Imprecision was rated as Serious. |
| Aidi Injection+OX | Pingxiao Capsules+OX | 0 | . | . | . |  |  |  |  |  | -1.58 [ -13.34; 10.18] | Very low | Low | Not serious | Very low | -1.58 [ -13.34; 10.18] | Very low | . | Serious | Very low | NMA estimate was used because incoherence was not available. Imprecision was rated as Serious. |
| Aidi Injection+OX | Shenmai Injection+OX | 0 | . | . | . |  |  |  |  |  | 43.92 [ -49.37; 137.21] | Very low | Low | Not serious | Very low | 43.92 [ -49.37; 137.21] | Very low | . | Serious | Very low | NMA estimate was used because incoherence was not available. Imprecision was rated as Serious. |
| Aidi Injection+OX | Shenqi Fuzheng Injection+OX | 0 | . | . | . |  |  |  |  |  | -1.13 [ -6.97; 4.72] | Very low | Very low | Not serious | Very low | -1.13 [ -6.97; 4.72] | Very low | . | Serious | Very low | NMA estimate was used because incoherence was not available. Imprecision was rated as Serious. |
| Aidi Injection+OX | Xiaoaiping Injection+OX | 0 | . | . | . |  |  |  |  |  | -5.77 [ -14.86; 3.31] | Very low | Very low | Not serious | Very low | -5.77 [ -14.86; 3.31] | Very low | . | Serious | Very low | NMA estimate was used because incoherence was not available. Imprecision was rated as Serious. |
| Aidi Injection+OX | Xihuang Capsules+OX | 0 | . | . | . |  |  |  |  |  | -0.95 [ -10.13; 8.23] | Very low | Low | Not serious | Very low | -0.95 [ -10.13; 8.23] | Very low | . | Serious | Very low | NMA estimate was used because incoherence was not available. Imprecision was rated as Serious. |
| Aidi Injection+OX | Ya Dan Zi Oil Emulsion Injection+OX | 0 | . | . | . |  |  |  |  |  | 1.38 [ -7.76; 10.52] | Very low | Very low | Not serious | Very low | 1.38 [ -7.76; 10.52] | Very low | . | Serious | Very low | NMA estimate was used because incoherence was not available. Imprecision was rated as Serious. |
| Aidi Injection+OX | Yangzheng Xiaoji Capsules+OX | 0 | . | . | . |  |  |  |  |  | 6.47 [ -5.34; 18.29] | Very low | High | Not serious | Very low | 6.47 [ -5.34; 18.29] | Very low | . | Serious | Very low | NMA estimate was used because incoherence was not available. Imprecision was rated as Serious. |
| Aidi Injection+OX | Zhenqi Fuzheng Granules+OX | 0 | . | . | . |  |  |  |  |  | 3.72 [ -8.14; 15.59] | Very low | Low | Not serious | Very low | 3.72 [ -8.14; 15.59] | Very low | . | Serious | Very low | NMA estimate was used because incoherence was not available. Imprecision was rated as Serious. |
| Astragalus Polysaccharides+OX | Astragalus preparations+OX | 0 | . | . | . |  |  |  |  |  | -2.55 [ -12.39; 7.29] | Low | Very low | Not serious | Very low | -2.55 [ -12.39; 7.29] | Very low | . | Serious | Very low | NMA estimate was used because incoherence was not available. Imprecision was rated as Serious. |
| Astragalus Polysaccharides+OX | Compound Kushen Injection+OX | 0 | . | . | . |  |  |  |  |  | -0.35 [ -8.58; 7.88] | Low | Very low | Not serious | Very low | -0.35 [ -8.58; 7.88] | Very low | . | Serious | Very low | NMA estimate was used because incoherence was not available. Imprecision was rated as Serious. |
| Astragalus Polysaccharides+OX | Compound Mylabris preparations+OX | 0 | . | . | . |  |  |  |  |  | -3.45 [ -13.28; 6.38] | Low | Low | Not serious | Low | -3.45 [ -13.28; 6.38] | Low | . | Serious | Very low | NMA estimate was used because incoherence was not available. Imprecision was rated as Serious. |
| Astragalus Polysaccharides+OX | Diyu Shengbai Tablet+OX | 0 | . | . | . |  |  |  |  |  | 2.83 [ -10.31; 15.98] | Low | Low | Not serious | Low | 2.83 [ -10.31; 15.98] | Low | . | Serious | Very low | NMA estimate was used because incoherence was not available. Imprecision was rated as Serious. |
| Astragalus Polysaccharides+OX | Ginseng Polysaccharide Injection+OX | 0 | . | . | . |  |  |  |  |  | -4.10 [ -17.30; 9.11] | Low | High | Not serious | Low | -4.10 [ -17.30; 9.11] | Low | . | Serious | Very low | NMA estimate was used because incoherence was not available. Imprecision was rated as Serious. |
| Astragalus Polysaccharides+OX | Huachansu preparations+OX | 0 | . | . | . |  |  |  |  |  | -4.44 [ -13.78; 4.90] | Low | Very low | Not serious | Very low | -4.44 [ -13.78; 4.90] | Very low | . | Serious | Very low | NMA estimate was used because incoherence was not available. Imprecision was rated as Serious. |
| Astragalus Polysaccharides+OX | Jinlong Capsules+OX | 0 | . | . | . |  |  |  |  |  | 1.18 [ -8.76; 11.11] | Low | High | Not serious | Low | 1.18 [ -8.76; 11.11] | Low | . | Serious | Very low | NMA estimate was used because incoherence was not available. Imprecision was rated as Serious. |
| Astragalus Polysaccharides+OX | Kangai Injection+OX | 0 | . | . | . |  |  |  |  |  | -3.76 [ -13.10; 5.58] | Low | Very low | Not serious | Very low | -3.76 [ -13.10; 5.58] | Very low | . | Serious | Very low | NMA estimate was used because incoherence was not available. Imprecision was rated as Serious. |
| Astragalus Polysaccharides+OX | Kanglixin Capsules+OX | 0 | . | . | . |  |  |  |  |  | 2.02 [ -8.78; 12.83] | Low | Low | Not serious | Low | 2.02 [ -8.78; 12.83] | Low | . | Serious | Very low | NMA estimate was used because incoherence was not available. Imprecision was rated as Serious. |
| Astragalus Polysaccharides+OX | Lentinan+OX | 0 | . | . | . |  |  |  |  |  | 0.87 [ -12.34; 14.09] | Low | High | Not serious | Low | 0.87 [ -12.34; 14.09] | Low | . | Serious | Very low | NMA estimate was used because incoherence was not available. Imprecision was rated as Serious. |
| Astragalus Polysaccharides+OX | OX | 2 | 151 | 0.0% | -3.99 [ -11.61; 3.63] | Very serious | Not serious | Not serious | Undetected | Low | . |  |  |  |  | -3.99 [ -11.61; 3.63] | Low | . | Serious | Very low | NMA estimate was used because incoherence was not available. Imprecision was rated as Serious. |
| Astragalus Polysaccharides+OX | Pingxiao Capsules+OX | 0 | . | . | . |  |  |  |  |  | -4.49 [ -17.60; 8.63] | Low | Low | Not serious | Low | -4.49 [ -17.60; 8.63] | Low | . | Serious | Very low | NMA estimate was used because incoherence was not available. Imprecision was rated as Serious. |
| Astragalus Polysaccharides+OX | Shenmai Injection+OX | 0 | . | . | . |  |  |  |  |  | 41.01 [ -52.46; 134.48] | Low | Low | Not serious | Low | 41.01 [ -52.46; 134.48] | Low | . | Serious | Very low | NMA estimate was used because incoherence was not available. Imprecision was rated as Serious. |
| Astragalus Polysaccharides+OX | Shenqi Fuzheng Injection+OX | 0 | . | . | . |  |  |  |  |  | -4.04 [ -12.28; 4.20] | Low | Very low | Not serious | Very low | -4.04 [ -12.28; 4.20] | Very low | . | Serious | Very low | NMA estimate was used because incoherence was not available. Imprecision was rated as Serious. |
| Astragalus Polysaccharides+OX | Xiaoaiping Injection+OX | 0 | . | . | . |  |  |  |  |  | -8.68 [ -19.47; 2.10] | Low | Very low | Not serious | Very low | -8.68 [ -19.47; 2.10] | Very low | . | Serious | Very low | NMA estimate was used because incoherence was not available. Imprecision was rated as Serious. |
| Astragalus Polysaccharides+OX | Xihuang Capsules+OX | 0 | . | . | . |  |  |  |  |  | -3.86 [ -14.72; 7.00] | Low | Low | Not serious | Low | -3.86 [ -14.72; 7.00] | Low | . | Serious | Very low | NMA estimate was used because incoherence was not available. Imprecision was rated as Serious. |
| Astragalus Polysaccharides+OX | Ya Dan Zi Oil Emulsion Injection+OX | 0 | . | . | . |  |  |  |  |  | -1.53 [ -12.36; 9.30] | Low | Very low | Not serious | Very low | -1.53 [ -12.36; 9.30] | Very low | . | Serious | Very low | NMA estimate was used because incoherence was not available. Imprecision was rated as Serious. |
| Astragalus Polysaccharides+OX | Yangzheng Xiaoji Capsules+OX | 0 | . | . | . |  |  |  |  |  | 3.56 [ -9.61; 16.73] | Low | High | Not serious | Low | 3.56 [ -9.61; 16.73] | Low | . | Serious | Very low | NMA estimate was used because incoherence was not available. Imprecision was rated as Serious. |
| Astragalus Polysaccharides+OX | Zhenqi Fuzheng Granules+OX | 0 | . | . | . |  |  |  |  |  | 0.81 [ -12.40; 14.02] | Low | Low | Not serious | Low | 0.81 [ -12.40; 14.02] | Low | . | Serious | Very low | NMA estimate was used because incoherence was not available. Imprecision was rated as Serious. |
| Astragalus preparations+OX | Compound Kushen Injection+OX | 0 | . | . | . |  |  |  |  |  | 2.20 [ -4.75; 9.16] | Very low | Very low | Not serious | Very low | 2.20 [ -4.75; 9.16] | Very low | . | Serious | Very low | NMA estimate was used because incoherence was not available. Imprecision was rated as Serious. |
| Astragalus preparations+OX | Compound Mylabris preparations+OX | 0 | . | . | . |  |  |  |  |  | -0.90 [ -9.69; 7.89] | Very low | Low | Not serious | Very low | -0.90 [ -9.69; 7.89] | Very low | . | Serious | Very low | NMA estimate was used because incoherence was not available. Imprecision was rated as Serious. |
| Astragalus preparations+OX | Diyu Shengbai Tablet+OX | 0 | . | . | . |  |  |  |  |  | 5.38 [ -7.00; 17.77] | Very low | Low | Not serious | Very low | 5.38 [ -7.00; 17.77] | Very low | . | Serious | Very low | NMA estimate was used because incoherence was not available. Imprecision was rated as Serious. |
| Astragalus preparations+OX | Ginseng Polysaccharide Injection+OX | 0 | . | . | . |  |  |  |  |  | -1.55 [ -14.00; 10.91] | Very low | High | Not serious | Very low | -1.55 [ -14.00; 10.91] | Very low | . | Serious | Very low | NMA estimate was used because incoherence was not available. Imprecision was rated as Serious. |
| Astragalus preparations+OX | Huachansu preparations+OX | 0 | . | . | . |  |  |  |  |  | -1.89 [ -10.13; 6.35] | Very low | Very low | Not serious | Very low | -1.89 [ -10.13; 6.35] | Very low | . | Serious | Very low | NMA estimate was used because incoherence was not available. Imprecision was rated as Serious. |
| Astragalus preparations+OX | Jinlong Capsules+OX | 0 | . | . | . |  |  |  |  |  | 3.73 [ -5.18; 12.63] | Very low | High | Not serious | Very low | 3.73 [ -5.18; 12.63] | Very low | . | Serious | Very low | NMA estimate was used because incoherence was not available. Imprecision was rated as Serious. |
| Astragalus preparations+OX | Kangai Injection+OX | 0 | . | . | . |  |  |  |  |  | -1.21 [ -9.45; 7.03] | Very low | Very low | Not serious | Very low | -1.21 [ -9.45; 7.03] | Very low | . | Serious | Very low | NMA estimate was used because incoherence was not available. Imprecision was rated as Serious. |
| Astragalus preparations+OX | Kanglixin Capsules+OX | 0 | . | . | . |  |  |  |  |  | 4.57 [ -5.29; 14.44] | Very low | Low | Not serious | Very low | 4.57 [ -5.29; 14.44] | Very low | . | Serious | Very low | NMA estimate was used because incoherence was not available. Imprecision was rated as Serious. |
| Astragalus preparations+OX | Lentinan+OX | 0 | . | . | . |  |  |  |  |  | 3.42 [ -9.04; 15.88] | Very low | High | Not serious | Very low | 3.42 [ -9.04; 15.88] | Very low | . | Serious | Very low | NMA estimate was used because incoherence was not available. Imprecision was rated as Serious. |
| Astragalus preparations+OX | OX | 3 | 295 | 91.6% | -1.44 [ -7.66; 4.78] | Very serious | Very serious | Not serious | Undetected | Very low | . |  |  |  |  | -1.44 [ -7.66; 4.78] | Very low | . | Serious | Very low | NMA estimate was used because incoherence was not available. Imprecision was rated as Serious. |
| Astragalus preparations+OX | Pingxiao Capsules+OX | 0 | . | . | . |  |  |  |  |  | -1.94 [ -14.29; 10.42] | Very low | Low | Not serious | Very low | -1.94 [ -14.29; 10.42] | Very low | . | Serious | Very low | NMA estimate was used because incoherence was not available. Imprecision was rated as Serious. |
| Astragalus preparations+OX | Shenmai Injection+OX | 0 | . | . | . |  |  |  |  |  | 43.56 [ -49.80; 136.93] | Very low | Low | Not serious | Very low | 43.56 [ -49.80; 136.93] | Very low | . | Serious | Very low | NMA estimate was used because incoherence was not available. Imprecision was rated as Serious. |
| Astragalus preparations+OX | Shenqi Fuzheng Injection+OX | 0 | . | . | . |  |  |  |  |  | -1.49 [ -8.45; 5.48] | Very low | Very low | Not serious | Very low | -1.49 [ -8.45; 5.48] | Very low | . | Serious | Very low | NMA estimate was used because incoherence was not available. Imprecision was rated as Serious. |
| Astragalus preparations+OX | Xiaoaiping Injection+OX | 0 | . | . | . |  |  |  |  |  | -6.13 [ -15.98; 3.71] | Very low | Very low | Not serious | Very low | -6.13 [ -15.98; 3.71] | Very low | . | Serious | Very low | NMA estimate was used because incoherence was not available. Imprecision was rated as Serious. |
| Astragalus preparations+OX | Xihuang Capsules+OX | 0 | . | . | . |  |  |  |  |  | -1.31 [ -11.24; 8.62] | Very low | Low | Not serious | Very low | -1.31 [ -11.24; 8.62] | Very low | . | Serious | Very low | NMA estimate was used because incoherence was not available. Imprecision was rated as Serious. |
| Astragalus preparations+OX | Ya Dan Zi Oil Emulsion Injection+OX | 0 | . | . | . |  |  |  |  |  | 1.02 [ -8.88; 10.92] | Very low | Very low | Not serious | Very low | 1.02 [ -8.88; 10.92] | Very low | . | Serious | Very low | NMA estimate was used because incoherence was not available. Imprecision was rated as Serious. |
| Astragalus preparations+OX | Yangzheng Xiaoji Capsules+OX | 0 | . | . | . |  |  |  |  |  | 6.11 [ -6.30; 18.53] | Very low | High | Not serious | Very low | 6.11 [ -6.30; 18.53] | Very low | . | Serious | Very low | NMA estimate was used because incoherence was not available. Imprecision was rated as Serious. |
| Astragalus preparations+OX | Zhenqi Fuzheng Granules+OX | 0 | . | . | . |  |  |  |  |  | 3.36 [ -9.09; 15.82] | Very low | Low | Not serious | Very low | 3.36 [ -9.09; 15.82] | Very low | . | Serious | Very low | NMA estimate was used because incoherence was not available. Imprecision was rated as Serious. |
| Compound Kushen Injection+OX | Compound Mylabris preparations+OX | 0 | . | . | . |  |  |  |  |  | -3.10 [ -10.05; 3.84] | Very low | Low | Not serious | Very low | -3.10 [ -10.05; 3.84] | Very low | . | Serious | Very low | NMA estimate was used because incoherence was not available. Imprecision was rated as Serious. |
| Compound Kushen Injection+OX | Diyu Shengbai Tablet+OX | 0 | . | . | . |  |  |  |  |  | 3.18 [ -7.97; 14.33] | Very low | Low | Not serious | Very low | 3.18 [ -7.97; 14.33] | Very low | . | Serious | Very low | NMA estimate was used because incoherence was not available. Imprecision was rated as Serious. |
| Compound Kushen Injection+OX | Ginseng Polysaccharide Injection+OX | 0 | . | . | . |  |  |  |  |  | -3.75 [ -14.98; 7.48] | Very low | High | Not serious | Very low | -3.75 [ -14.98; 7.48] | Very low | . | Serious | Very low | NMA estimate was used because incoherence was not available. Imprecision was rated as Serious. |
| Compound Kushen Injection+OX | Huachansu preparations+OX | 0 | . | . | . |  |  |  |  |  | -4.10 [ -10.33; 2.14] | Very low | Very low | Not serious | Very low | -4.10 [ -10.33; 2.14] | Very low | . | Serious | Very low | NMA estimate was used because incoherence was not available. Imprecision was rated as Serious. |
| Compound Kushen Injection+OX | Jinlong Capsules+OX | 0 | . | . | . |  |  |  |  |  | 1.52 [ -5.57; 8.61] | Very low | High | Not serious | Very low | 1.52 [ -5.57; 8.61] | Very low | . | Serious | Very low | NMA estimate was used because incoherence was not available. Imprecision was rated as Serious. |
| Compound Kushen Injection+OX | Kangai Injection+OX | 0 | . | . | . |  |  |  |  |  | -3.41 [ -9.64; 2.81] | Very low | Very low | Not serious | Very low | -3.41 [ -9.64; 2.81] | Very low | . | Serious | Very low | NMA estimate was used because incoherence was not available. Imprecision was rated as Serious. |
| Compound Kushen Injection+OX | Kanglixin Capsules+OX | 0 | . | . | . |  |  |  |  |  | 2.37 [ -5.90; 10.64] | Very low | Low | Not serious | Very low | 2.37 [ -5.90; 10.64] | Very low | . | Serious | Very low | NMA estimate was used because incoherence was not available. Imprecision was rated as Serious. |
| Compound Kushen Injection+OX | Lentinan+OX | 0 | . | . | . |  |  |  |  |  | 1.22 [ -10.02; 12.46] | Very low | High | Not serious | Very low | 1.22 [ -10.02; 12.46] | Very low | . | Serious | Very low | NMA estimate was used because incoherence was not available. Imprecision was rated as Serious. |
| Compound Kushen Injection+OX | OX | 12 | 1075 | 98.6% | -3.64 [ -6.75; -0.53] | Very serious | Very serious | Not serious | Not serious | Very low | . |  |  |  |  | -3.64 [ -6.75; -0.53] | Very low | . | Not serious | Very low | NMA estimate was used because incoherence was not available. Imprecision was rated as Not serious. |
| Compound Kushen Injection+OX | Pingxiao Capsules+OX | 0 | . | . | . |  |  |  |  |  | -4.14 [ -15.26; 6.98] | Very low | Low | Not serious | Very low | -4.14 [ -15.26; 6.98] | Very low | . | Serious | Very low | NMA estimate was used because incoherence was not available. Imprecision was rated as Serious. |
| Compound Kushen Injection+OX | Shenmai Injection+OX | 0 | . | . | . |  |  |  |  |  | 41.36 [ -51.85; 134.57] | Very low | Low | Not serious | Very low | 41.36 [ -51.85; 134.57] | Very low | . | Serious | Very low | NMA estimate was used because incoherence was not available. Imprecision was rated as Serious. |
| Compound Kushen Injection+OX | Shenqi Fuzheng Injection+OX | 0 | . | . | . |  |  |  |  |  | -3.69 [ -8.11; 0.73] | Very low | Very low | Not serious | Very low | -3.69 [ -8.11; 0.73] | Very low | . | Serious | Very low | NMA estimate was used because incoherence was not available. Imprecision was rated as Serious. |
| Compound Kushen Injection+OX | Xiaoaiping Injection+OX | 0 | . | . | . |  |  |  |  |  | -8.34 [ -16.58; -0.10] | Very low | Very low | Not serious | Very low | -8.34 [ -16.58; -0.10] | Very low | . | Serious | Very low | NMA estimate was used because incoherence was not available. Imprecision was rated as Serious. |
| Compound Kushen Injection+OX | Xihuang Capsules+OX | 0 | . | . | . |  |  |  |  |  | -3.52 [ -11.86; 4.83] | Very low | Low | Not serious | Very low | -3.52 [ -11.86; 4.83] | Very low | . | Serious | Very low | NMA estimate was used because incoherence was not available. Imprecision was rated as Serious. |
| Compound Kushen Injection+OX | Ya Dan Zi Oil Emulsion Injection+OX | 0 | . | . | . |  |  |  |  |  | -1.19 [ -9.49; 7.12] | Very low | Very low | Not serious | Very low | -1.19 [ -9.49; 7.12] | Very low | . | Serious | Very low | NMA estimate was used because incoherence was not available. Imprecision was rated as Serious. |
| Compound Kushen Injection+OX | Yangzheng Xiaoji Capsules+OX | 0 | . | . | . |  |  |  |  |  | 3.91 [ -7.27; 15.09] | Very low | High | Not serious | Very low | 3.91 [ -7.27; 15.09] | Very low | . | Serious | Very low | NMA estimate was used because incoherence was not available. Imprecision was rated as Serious. |
| Compound Kushen Injection+OX | Zhenqi Fuzheng Granules+OX | 0 | . | . | . |  |  |  |  |  | 1.16 [ -10.07; 12.39] | Very low | Low | Not serious | Very low | 1.16 [ -10.07; 12.39] | Very low | . | Serious | Very low | NMA estimate was used because incoherence was not available. Imprecision was rated as Serious. |
| Compound Mylabris preparations+OX | Diyu Shengbai Tablet+OX | 0 | . | . | . |  |  |  |  |  | 6.28 [ -6.10; 18.66] | Low | Low | Not serious | Low | 6.28 [ -6.10; 18.66] | Low | . | Serious | Very low | NMA estimate was used because incoherence was not available. Imprecision was rated as Serious. |
| Compound Mylabris preparations+OX | Ginseng Polysaccharide Injection+OX | 0 | . | . | . |  |  |  |  |  | -0.65 [ -13.09; 11.80] | Low | High | Not serious | Low | -0.65 [ -13.09; 11.80] | Low | . | Serious | Very low | NMA estimate was used because incoherence was not available. Imprecision was rated as Serious. |
| Compound Mylabris preparations+OX | Huachansu preparations+OX | 0 | . | . | . |  |  |  |  |  | -0.99 [ -9.22; 7.24] | Low | Very low | Not serious | Very low | -0.99 [ -9.22; 7.24] | Very low | . | Serious | Very low | NMA estimate was used because incoherence was not available. Imprecision was rated as Serious. |
| Compound Mylabris preparations+OX | Jinlong Capsules+OX | 0 | . | . | . |  |  |  |  |  | 4.63 [ -4.27; 13.52] | Low | High | Not serious | Low | 4.63 [ -4.27; 13.52] | Low | . | Serious | Very low | NMA estimate was used because incoherence was not available. Imprecision was rated as Serious. |
| Compound Mylabris preparations+OX | Kangai Injection+OX | 0 | . | . | . |  |  |  |  |  | -0.31 [ -8.54; 7.92] | Low | Very low | Not serious | Very low | -0.31 [ -8.54; 7.92] | Very low | . | Serious | Very low | NMA estimate was used because incoherence was not available. Imprecision was rated as Serious. |
| Compound Mylabris preparations+OX | Kanglixin Capsules+OX | 0 | . | . | . |  |  |  |  |  | 5.47 [ -4.39; 15.33] | Low | Low | Not serious | Low | 5.47 [ -4.39; 15.33] | Low | . | Serious | Very low | NMA estimate was used because incoherence was not available. Imprecision was rated as Serious. |
| Compound Mylabris preparations+OX | Lentinan+OX | 0 | . | . | . |  |  |  |  |  | 4.32 [ -8.13; 16.78] | Low | High | Not serious | Low | 4.32 [ -8.13; 16.78] | Low | . | Serious | Very low | NMA estimate was used because incoherence was not available. Imprecision was rated as Serious. |
| Compound Mylabris preparations+OX | OX | 3 | 273 | 53.2% | -0.54 [ -6.75; 5.67] | Very serious | Not serious | Not serious | Undetected | Low | . |  |  |  |  | -0.54 [ -6.75; 5.67] | Low | . | Serious | Very low | NMA estimate was used because incoherence was not available. Imprecision was rated as Serious. |
| Compound Mylabris preparations+OX | Pingxiao Capsules+OX | 0 | . | . | . |  |  |  |  |  | -1.04 [ -13.39; 11.31] | Low | Low | Not serious | Low | -1.04 [ -13.39; 11.31] | Low | . | Serious | Very low | NMA estimate was used because incoherence was not available. Imprecision was rated as Serious. |
| Compound Mylabris preparations+OX | Shenmai Injection+OX | 0 | . | . | . |  |  |  |  |  | 44.46 [ -48.90; 137.83] | Low | Low | Not serious | Low | 44.46 [ -48.90; 137.83] | Low | . | Serious | Very low | NMA estimate was used because incoherence was not available. Imprecision was rated as Serious. |
| Compound Mylabris preparations+OX | Shenqi Fuzheng Injection+OX | 0 | . | . | . |  |  |  |  |  | -0.59 [ -7.54; 6.37] | Low | Very low | Not serious | Very low | -0.59 [ -7.54; 6.37] | Very low | . | Serious | Very low | NMA estimate was used because incoherence was not available. Imprecision was rated as Serious. |
| Compound Mylabris preparations+OX | Xiaoaiping Injection+OX | 0 | . | . | . |  |  |  |  |  | -5.23 [ -15.07; 4.60] | Low | Very low | Not serious | Very low | -5.23 [ -15.07; 4.60] | Very low | . | Serious | Very low | NMA estimate was used because incoherence was not available. Imprecision was rated as Serious. |
| Compound Mylabris preparations+OX | Xihuang Capsules+OX | 0 | . | . | . |  |  |  |  |  | -0.41 [ -10.34; 9.51] | Low | Low | Not serious | Low | -0.41 [ -10.34; 9.51] | Low | . | Serious | Very low | NMA estimate was used because incoherence was not available. Imprecision was rated as Serious. |
| Compound Mylabris preparations+OX | Ya Dan Zi Oil Emulsion Injection+OX | 0 | . | . | . |  |  |  |  |  | 1.92 [ -7.97; 11.81] | Low | Very low | Not serious | Very low | 1.92 [ -7.97; 11.81] | Very low | . | Serious | Very low | NMA estimate was used because incoherence was not available. Imprecision was rated as Serious. |
| Compound Mylabris preparations+OX | Yangzheng Xiaoji Capsules+OX | 0 | . | . | . |  |  |  |  |  | 7.01 [ -5.39; 19.42] | Low | High | Not serious | Low | 7.01 [ -5.39; 19.42] | Low | . | Serious | Very low | NMA estimate was used because incoherence was not available. Imprecision was rated as Serious. |
| Compound Mylabris preparations+OX | Zhenqi Fuzheng Granules+OX | 0 | . | . | . |  |  |  |  |  | 4.26 [ -8.19; 16.71] | Low | Low | Not serious | Low | 4.26 [ -8.19; 16.71] | Low | . | Serious | Very low | NMA estimate was used because incoherence was not available. Imprecision was rated as Serious. |
| Diyu Shengbai Tablet+OX | Ginseng Polysaccharide Injection+OX | 0 | . | . | . |  |  |  |  |  | -6.93 [ -22.13; 8.27] | Low | High | Not serious | Low | -6.93 [ -22.13; 8.27] | Low | . | Serious | Very low | NMA estimate was used because incoherence was not available. Imprecision was rated as Serious. |
| Diyu Shengbai Tablet+OX | Huachansu preparations+OX | 0 | . | . | . |  |  |  |  |  | -7.28 [ -19.27; 4.72] | Low | Very low | Not serious | Very low | -7.28 [ -19.27; 4.72] | Very low | . | Serious | Very low | NMA estimate was used because incoherence was not available. Imprecision was rated as Serious. |
| Diyu Shengbai Tablet+OX | Jinlong Capsules+OX | 0 | . | . | . |  |  |  |  |  | -1.66 [ -14.12; 10.81] | Low | High | Not serious | Low | -1.66 [ -14.12; 10.81] | Low | . | Serious | Very low | NMA estimate was used because incoherence was not available. Imprecision was rated as Serious. |
| Diyu Shengbai Tablet+OX | Kangai Injection+OX | 0 | . | . | . |  |  |  |  |  | -6.59 [ -18.59; 5.40] | Low | Very low | Not serious | Very low | -6.59 [ -18.59; 5.40] | Very low | . | Serious | Very low | NMA estimate was used because incoherence was not available. Imprecision was rated as Serious. |
| Diyu Shengbai Tablet+OX | Kanglixin Capsules+OX | 0 | . | . | . |  |  |  |  |  | -0.81 [ -13.98; 12.36] | Low | Low | Not serious | Low | -0.81 [ -13.98; 12.36] | Low | . | Serious | Very low | NMA estimate was used because incoherence was not available. Imprecision was rated as Serious. |
| Diyu Shengbai Tablet+OX | Lentinan+OX | 0 | . | . | . |  |  |  |  |  | -1.96 [ -17.17; 13.25] | Low | High | Not serious | Low | -1.96 [ -17.17; 13.25] | Low | . | Serious | Very low | NMA estimate was used because incoherence was not available. Imprecision was rated as Serious. |
| Diyu Shengbai Tablet+OX | OX | 1 | 101 | . | -6.82 [ -17.53; 3.89] | Very serious | Not serious | Not serious | Undetected | Low | . |  |  |  |  | -6.82 [ -17.53; 3.89] | Low | . | Serious | Very low | NMA estimate was used because incoherence was not available. Imprecision was rated as Serious. |
| Diyu Shengbai Tablet+OX | Pingxiao Capsules+OX | 0 | . | . | . |  |  |  |  |  | -7.32 [ -22.44; 7.80] | Low | Low | Not serious | Low | -7.32 [ -22.44; 7.80] | Low | . | Serious | Very low | NMA estimate was used because incoherence was not available. Imprecision was rated as Serious. |
| Diyu Shengbai Tablet+OX | Shenmai Injection+OX | 0 | . | . | . |  |  |  |  |  | 38.18 [ -55.59; 131.95] | Low | Low | Not serious | Low | 38.18 [ -55.59; 131.95] | Low | . | Serious | Very low | NMA estimate was used because incoherence was not available. Imprecision was rated as Serious. |
| Diyu Shengbai Tablet+OX | Shenqi Fuzheng Injection+OX | 0 | . | . | . |  |  |  |  |  | -6.87 [ -18.03; 4.29] | Low | Very low | Not serious | Very low | -6.87 [ -18.03; 4.29] | Very low | . | Serious | Very low | NMA estimate was used because incoherence was not available. Imprecision was rated as Serious. |
| Diyu Shengbai Tablet+OX | Xiaoaiping Injection+OX | 0 | . | . | . |  |  |  |  |  | -11.52 [ -24.67; 1.63] | Low | Very low | Not serious | Very low | -11.52 [ -24.67; 1.63] | Very low | . | Serious | Very low | NMA estimate was used because incoherence was not available. Imprecision was rated as Serious. |
| Diyu Shengbai Tablet+OX | Xihuang Capsules+OX | 0 | . | . | . |  |  |  |  |  | -6.69 [ -19.91; 6.52] | Low | Low | Not serious | Low | -6.69 [ -19.91; 6.52] | Low | . | Serious | Very low | NMA estimate was used because incoherence was not available. Imprecision was rated as Serious. |
| Diyu Shengbai Tablet+OX | Ya Dan Zi Oil Emulsion Injection+OX | 0 | . | . | . |  |  |  |  |  | -4.36 [ -17.55; 8.83] | Low | Very low | Not serious | Very low | -4.36 [ -17.55; 8.83] | Very low | . | Serious | Very low | NMA estimate was used because incoherence was not available. Imprecision was rated as Serious. |
| Diyu Shengbai Tablet+OX | Yangzheng Xiaoji Capsules+OX | 0 | . | . | . |  |  |  |  |  | 0.73 [ -14.44; 15.90] | Low | High | Not serious | Low | 0.73 [ -14.44; 15.90] | Low | . | Serious | Very low | NMA estimate was used because incoherence was not available. Imprecision was rated as Serious. |
| Diyu Shengbai Tablet+OX | Zhenqi Fuzheng Granules+OX | 0 | . | . | . |  |  |  |  |  | -2.02 [ -17.23; 13.19] | Low | Low | Not serious | Low | -2.02 [ -17.23; 13.19] | Low | . | Serious | Very low | NMA estimate was used because incoherence was not available. Imprecision was rated as Serious. |
| Ginseng Polysaccharide Injection+OX | Huachansu preparations+OX | 0 | . | . | . |  |  |  |  |  | -0.35 [ -12.41; 11.72] | High | Very low | Not serious | Very low | -0.35 [ -12.41; 11.72] | Very low | . | Serious | Very low | NMA estimate was used because incoherence was not available. Imprecision was rated as Serious. |
| Ginseng Polysaccharide Injection+OX | Jinlong Capsules+OX | 0 | . | . | . |  |  |  |  |  | 5.27 [ -7.26; 17.80] | High | High | Not serious | High | 5.27 [ -7.26; 17.80] | High | . | Serious | Moderate | NMA estimate was used because incoherence was not available. Imprecision was rated as Serious. |
| Ginseng Polysaccharide Injection+OX | Kangai Injection+OX | 0 | . | . | . |  |  |  |  |  | 0.34 [ -11.73; 12.40] | High | Very low | Not serious | Very low | 0.34 [ -11.73; 12.40] | Very low | . | Serious | Very low | NMA estimate was used because incoherence was not available. Imprecision was rated as Serious. |
| Ginseng Polysaccharide Injection+OX | Kanglixin Capsules+OX | 0 | . | . | . |  |  |  |  |  | 6.12 [ -7.11; 19.35] | High | Low | Not serious | Low | 6.12 [ -7.11; 19.35] | Low | . | Serious | Very low | NMA estimate was used because incoherence was not available. Imprecision was rated as Serious. |
| Ginseng Polysaccharide Injection+OX | Lentinan+OX | 0 | . | . | . |  |  |  |  |  | 4.97 [ -10.29; 20.23] | High | High | Not serious | High | 4.97 [ -10.29; 20.23] | High | . | Serious | Moderate | NMA estimate was used because incoherence was not available. Imprecision was rated as Serious. |
| Ginseng Polysaccharide Injection+OX | OX | 1 | 68 | . | 0.11 [ -10.68; 10.90] | Not serious | Not serious | Not serious | Undetected | High | . |  |  |  |  | 0.11 [ -10.68; 10.90] | High | . | Serious | Moderate | NMA estimate was used because incoherence was not available. Imprecision was rated as Serious. |
| Ginseng Polysaccharide Injection+OX | Pingxiao Capsules+OX | 0 | . | . | . |  |  |  |  |  | -0.39 [ -15.57; 14.79] | High | Low | Not serious | Low | -0.39 [ -15.57; 14.79] | Low | . | Serious | Very low | NMA estimate was used because incoherence was not available. Imprecision was rated as Serious. |
| Ginseng Polysaccharide Injection+OX | Shenmai Injection+OX | 0 | . | . | . |  |  |  |  |  | 45.11 [ -48.67; 138.89] | High | Low | Not serious | Low | 45.11 [ -48.67; 138.89] | Low | . | Serious | Very low | NMA estimate was used because incoherence was not available. Imprecision was rated as Serious. |
| Ginseng Polysaccharide Injection+OX | Shenqi Fuzheng Injection+OX | 0 | . | . | . |  |  |  |  |  | 0.06 [ -11.18; 11.29] | High | Very low | Not serious | Very low | 0.06 [ -11.18; 11.29] | Very low | . | Serious | Very low | NMA estimate was used because incoherence was not available. Imprecision was rated as Serious. |
| Ginseng Polysaccharide Injection+OX | Xiaoaiping Injection+OX | 0 | . | . | . |  |  |  |  |  | -4.59 [ -17.80; 8.63] | High | Very low | Not serious | Very low | -4.59 [ -17.80; 8.63] | Very low | . | Serious | Very low | NMA estimate was used because incoherence was not available. Imprecision was rated as Serious. |
| Ginseng Polysaccharide Injection+OX | Xihuang Capsules+OX | 0 | . | . | . |  |  |  |  |  | 0.24 [ -13.04; 13.51] | High | Low | Not serious | Low | 0.24 [ -13.04; 13.51] | Low | . | Serious | Very low | NMA estimate was used because incoherence was not available. Imprecision was rated as Serious. |
| Ginseng Polysaccharide Injection+OX | Ya Dan Zi Oil Emulsion Injection+OX | 0 | . | . | . |  |  |  |  |  | 2.57 [ -10.69; 15.82] | High | Very low | Not serious | Very low | 2.57 [ -10.69; 15.82] | Very low | . | Serious | Very low | NMA estimate was used because incoherence was not available. Imprecision was rated as Serious. |
| Ginseng Polysaccharide Injection+OX | Yangzheng Xiaoji Capsules+OX | 0 | . | . | . |  |  |  |  |  | 7.66 [ -7.56; 22.88] | High | High | Not serious | High | 7.66 [ -7.56; 22.88] | High | . | Serious | Moderate | NMA estimate was used because incoherence was not available. Imprecision was rated as Serious. |
| Ginseng Polysaccharide Injection+OX | Zhenqi Fuzheng Granules+OX | 0 | . | . | . |  |  |  |  |  | 4.91 [ -10.35; 20.17] | High | Low | Not serious | Low | 4.91 [ -10.35; 20.17] | Low | . | Serious | Very low | NMA estimate was used because incoherence was not available. Imprecision was rated as Serious. |
| Huachansu preparations+OX | Jinlong Capsules+OX | 0 | . | . | . |  |  |  |  |  | 5.62 [ -2.73; 13.97] | Very low | High | Not serious | Very low | 5.62 [ -2.73; 13.97] | Very low | . | Serious | Very low | NMA estimate was used because incoherence was not available. Imprecision was rated as Serious. |
| Huachansu preparations+OX | Kangai Injection+OX | 0 | . | . | . |  |  |  |  |  | 0.68 [ -6.95; 8.32] | Very low | Very low | Not serious | Very low | 0.68 [ -6.95; 8.32] | Very low | . | Serious | Very low | NMA estimate was used because incoherence was not available. Imprecision was rated as Serious. |
| Huachansu preparations+OX | Kanglixin Capsules+OX | 0 | . | . | . |  |  |  |  |  | 6.47 [ -2.91; 15.84] | Very low | Low | Not serious | Very low | 6.47 [ -2.91; 15.84] | Very low | . | Serious | Very low | NMA estimate was used because incoherence was not available. Imprecision was rated as Serious. |
| Huachansu preparations+OX | Lentinan+OX | 0 | . | . | . |  |  |  |  |  | 5.32 [ -6.76; 17.39] | Very low | High | Not serious | Very low | 5.32 [ -6.76; 17.39] | Very low | . | Serious | Very low | NMA estimate was used because incoherence was not available. Imprecision was rated as Serious. |
| Huachansu preparations+OX | OX | 4 | 283 | 96.5% | 0.46 [ -4.94; 5.86] | Serious | Very serious | Not serious | Undetected | Very low | . |  |  |  |  | 0.46 [ -4.94; 5.86] | Very low | . | Serious | Very low | NMA estimate was used because incoherence was not available. Imprecision was rated as Serious. |
| Huachansu preparations+OX | Pingxiao Capsules+OX | 0 | . | . | . |  |  |  |  |  | -0.04 [ -12.01; 11.92] | Very low | Low | Not serious | Very low | -0.04 [ -12.01; 11.92] | Very low | . | Serious | Very low | NMA estimate was used because incoherence was not available. Imprecision was rated as Serious. |
| Huachansu preparations+OX | Shenmai Injection+OX | 0 | . | . | . |  |  |  |  |  | 45.46 [ -47.86; 138.77] | Very low | Low | Not serious | Very low | 45.46 [ -47.86; 138.77] | Very low | . | Serious | Very low | NMA estimate was used because incoherence was not available. Imprecision was rated as Serious. |
| Huachansu preparations+OX | Shenqi Fuzheng Injection+OX | 0 | . | . | . |  |  |  |  |  | 0.40 [ -5.84; 6.65] | Very low | Very low | Not serious | Very low | 0.40 [ -5.84; 6.65] | Very low | . | Serious | Very low | NMA estimate was used because incoherence was not available. Imprecision was rated as Serious. |
| Huachansu preparations+OX | Xiaoaiping Injection+OX | 0 | . | . | . |  |  |  |  |  | -4.24 [ -13.59; 5.11] | Very low | Very low | Not serious | Very low | -4.24 [ -13.59; 5.11] | Very low | . | Serious | Very low | NMA estimate was used because incoherence was not available. Imprecision was rated as Serious. |
| Huachansu preparations+OX | Xihuang Capsules+OX | 0 | . | . | . |  |  |  |  |  | 0.58 [ -8.86; 10.02] | Very low | Low | Not serious | Very low | 0.58 [ -8.86; 10.02] | Very low | . | Serious | Very low | NMA estimate was used because incoherence was not available. Imprecision was rated as Serious. |
| Huachansu preparations+OX | Ya Dan Zi Oil Emulsion Injection+OX | 0 | . | . | . |  |  |  |  |  | 2.91 [ -6.49; 12.32] | Very low | Very low | Not serious | Very low | 2.91 [ -6.49; 12.32] | Very low | . | Serious | Very low | NMA estimate was used because incoherence was not available. Imprecision was rated as Serious. |
| Huachansu preparations+OX | Yangzheng Xiaoji Capsules+OX | 0 | . | . | . |  |  |  |  |  | 8.01 [ -4.02; 20.03] | Very low | High | Not serious | Very low | 8.01 [ -4.02; 20.03] | Very low | . | Serious | Very low | NMA estimate was used because incoherence was not available. Imprecision was rated as Serious. |
| Huachansu preparations+OX | Zhenqi Fuzheng Granules+OX | 0 | . | . | . |  |  |  |  |  | 5.26 [ -6.81; 17.32] | Very low | Low | Not serious | Very low | 5.26 [ -6.81; 17.32] | Very low | . | Serious | Very low | NMA estimate was used because incoherence was not available. Imprecision was rated as Serious. |
| Jinlong Capsules+OX | Kangai Injection+OX | 0 | . | . | . |  |  |  |  |  | -4.94 [ -13.29; 3.42] | High | Very low | Not serious | Very low | -4.94 [ -13.29; 3.42] | Very low | . | Serious | Very low | NMA estimate was used because incoherence was not available. Imprecision was rated as Serious. |
| Jinlong Capsules+OX | Kanglixin Capsules+OX | 0 | . | . | . |  |  |  |  |  | 0.85 [ -9.12; 10.81] | High | Low | Not serious | Low | 0.85 [ -9.12; 10.81] | Low | . | Serious | Very low | NMA estimate was used because incoherence was not available. Imprecision was rated as Serious. |
| Jinlong Capsules+OX | Lentinan+OX | 0 | . | . | . |  |  |  |  |  | -0.30 [ -12.84; 12.23] | High | High | Not serious | High | -0.30 [ -12.84; 12.23] | High | . | Serious | Moderate | NMA estimate was used because incoherence was not available. Imprecision was rated as Serious. |
| Jinlong Capsules+OX | OX | 3 | 194 | 61.9% | -5.16 [ -11.54; 1.21] | Not serious | Not serious | Not serious | Undetected | High | . |  |  |  |  | -5.16 [ -11.54; 1.21] | High | . | Serious | Moderate | NMA estimate was used because incoherence was not available. Imprecision was rated as Serious. |
| Jinlong Capsules+OX | Pingxiao Capsules+OX | 0 | . | . | . |  |  |  |  |  | -5.66 [ -18.10; 6.77] | High | Low | Not serious | Low | -5.66 [ -18.10; 6.77] | Low | . | Serious | Very low | NMA estimate was used because incoherence was not available. Imprecision was rated as Serious. |
| Jinlong Capsules+OX | Shenmai Injection+OX | 0 | . | . | . |  |  |  |  |  | 39.84 [ -53.54; 133.21] | High | Low | Not serious | Low | 39.84 [ -53.54; 133.21] | Low | . | Serious | Very low | NMA estimate was used because incoherence was not available. Imprecision was rated as Serious. |
| Jinlong Capsules+OX | Shenqi Fuzheng Injection+OX | 0 | . | . | . |  |  |  |  |  | -5.21 [ -12.32; 1.89] | High | Very low | Not serious | Very low | -5.21 [ -12.32; 1.89] | Very low | . | Serious | Very low | NMA estimate was used because incoherence was not available. Imprecision was rated as Serious. |
| Jinlong Capsules+OX | Xiaoaiping Injection+OX | 0 | . | . | . |  |  |  |  |  | -9.86 [ -19.80; 0.08] | High | Very low | Not serious | Very low | -9.86 [ -19.80; 0.08] | Very low | . | Serious | Very low | NMA estimate was used because incoherence was not available. Imprecision was rated as Serious. |
| Jinlong Capsules+OX | Xihuang Capsules+OX | 0 | . | . | . |  |  |  |  |  | -5.04 [ -15.06; 4.99] | High | Low | Not serious | Low | -5.04 [ -15.06; 4.99] | Low | . | Serious | Very low | NMA estimate was used because incoherence was not available. Imprecision was rated as Serious. |
| Jinlong Capsules+OX | Ya Dan Zi Oil Emulsion Injection+OX | 0 | . | . | . |  |  |  |  |  | -2.71 [ -12.70; 7.29] | High | Very low | Not serious | Very low | -2.71 [ -12.70; 7.29] | Very low | . | Serious | Very low | NMA estimate was used because incoherence was not available. Imprecision was rated as Serious. |
| Jinlong Capsules+OX | Yangzheng Xiaoji Capsules+OX | 0 | . | . | . |  |  |  |  |  | 2.39 [ -10.10; 14.88] | High | High | Not serious | High | 2.39 [ -10.10; 14.88] | High | . | Serious | Moderate | NMA estimate was used because incoherence was not available. Imprecision was rated as Serious. |
| Jinlong Capsules+OX | Zhenqi Fuzheng Granules+OX | 0 | . | . | . |  |  |  |  |  | -0.36 [ -12.90; 12.17] | High | Low | Not serious | Low | -0.36 [ -12.90; 12.17] | Low | . | Serious | Very low | NMA estimate was used because incoherence was not available. Imprecision was rated as Serious. |
| Kangai Injection+OX | Kanglixin Capsules+OX | 0 | . | . | . |  |  |  |  |  | 5.78 [ -3.59; 15.15] | Very low | Low | Not serious | Very low | 5.78 [ -3.59; 15.15] | Very low | . | Serious | Very low | NMA estimate was used because incoherence was not available. Imprecision was rated as Serious. |
| Kangai Injection+OX | Lentinan+OX | 0 | . | . | . |  |  |  |  |  | 4.63 [ -7.44; 16.70] | Very low | High | Not serious | Very low | 4.63 [ -7.44; 16.70] | Very low | . | Serious | Very low | NMA estimate was used because incoherence was not available. Imprecision was rated as Serious. |
| Kangai Injection+OX | OX | 4 | 289 | 97.9% | -0.23 [ -5.62; 5.17] | Serious | Very serious | Not serious | Undetected | Very low | . |  |  |  |  | -0.23 [ -5.62; 5.17] | Very low | . | Serious | Very low | NMA estimate was used because incoherence was not available. Imprecision was rated as Serious. |
| Kangai Injection+OX | Pingxiao Capsules+OX | 0 | . | . | . |  |  |  |  |  | -0.73 [ -12.69; 11.24] | Very low | Low | Not serious | Very low | -0.73 [ -12.69; 11.24] | Very low | . | Serious | Very low | NMA estimate was used because incoherence was not available. Imprecision was rated as Serious. |
| Kangai Injection+OX | Shenmai Injection+OX | 0 | . | . | . |  |  |  |  |  | 44.77 [ -48.54; 138.09] | Very low | Low | Not serious | Very low | 44.77 [ -48.54; 138.09] | Very low | . | Serious | Very low | NMA estimate was used because incoherence was not available. Imprecision was rated as Serious. |
| Kangai Injection+OX | Shenqi Fuzheng Injection+OX | 0 | . | . | . |  |  |  |  |  | -0.28 [ -6.52; 5.96] | Very low | Very low | Not serious | Very low | -0.28 [ -6.52; 5.96] | Very low | . | Serious | Very low | NMA estimate was used because incoherence was not available. Imprecision was rated as Serious. |
| Kangai Injection+OX | Xiaoaiping Injection+OX | 0 | . | . | . |  |  |  |  |  | -4.92 [ -14.27; 4.42] | Very low | Very low | Not serious | Very low | -4.92 [ -14.27; 4.42] | Very low | . | Serious | Very low | NMA estimate was used because incoherence was not available. Imprecision was rated as Serious. |
| Kangai Injection+OX | Xihuang Capsules+OX | 0 | . | . | . |  |  |  |  |  | -0.10 [ -9.54; 9.34] | Very low | Low | Not serious | Very low | -0.10 [ -9.54; 9.34] | Very low | . | Serious | Very low | NMA estimate was used because incoherence was not available. Imprecision was rated as Serious. |
| Kangai Injection+OX | Ya Dan Zi Oil Emulsion Injection+OX | 0 | . | . | . |  |  |  |  |  | 2.23 [ -7.17; 11.63] | Very low | Very low | Not serious | Very low | 2.23 [ -7.17; 11.63] | Very low | . | Serious | Very low | NMA estimate was used because incoherence was not available. Imprecision was rated as Serious. |
| Kangai Injection+OX | Yangzheng Xiaoji Capsules+OX | 0 | . | . | . |  |  |  |  |  | 7.32 [ -4.70; 19.34] | Very low | High | Not serious | Very low | 7.32 [ -4.70; 19.34] | Very low | . | Serious | Very low | NMA estimate was used because incoherence was not available. Imprecision was rated as Serious. |
| Kangai Injection+OX | Zhenqi Fuzheng Granules+OX | 0 | . | . | . |  |  |  |  |  | 4.57 [ -7.49; 16.64] | Very low | Low | Not serious | Very low | 4.57 [ -7.49; 16.64] | Very low | . | Serious | Very low | NMA estimate was used because incoherence was not available. Imprecision was rated as Serious. |
| Kanglixin Capsules+OX | Lentinan+OX | 0 | . | . | . |  |  |  |  |  | -1.15 [ -14.39; 12.09] | Low | High | Not serious | Low | -1.15 [ -14.39; 12.09] | Low | . | Serious | Very low | NMA estimate was used because incoherence was not available. Imprecision was rated as Serious. |
| Kanglixin Capsules+OX | OX | 2 | 140 | 41.0% | -6.01 [ -13.67; 1.65] | Very serious | Not serious | Not serious | Undetected | Low | . |  |  |  |  | -6.01 [ -13.67; 1.65] | Low | . | Serious | Very low | NMA estimate was used because incoherence was not available. Imprecision was rated as Serious. |
| Kanglixin Capsules+OX | Pingxiao Capsules+OX | 0 | . | . | . |  |  |  |  |  | -6.51 [ -19.65; 6.63] | Low | Low | Not serious | Low | -6.51 [ -19.65; 6.63] | Low | . | Serious | Very low | NMA estimate was used because incoherence was not available. Imprecision was rated as Serious. |
| Kanglixin Capsules+OX | Shenmai Injection+OX | 0 | . | . | . |  |  |  |  |  | 38.99 [ -54.48; 132.46] | Low | Low | Not serious | Low | 38.99 [ -54.48; 132.46] | Low | . | Serious | Very low | NMA estimate was used because incoherence was not available. Imprecision was rated as Serious. |
| Kanglixin Capsules+OX | Shenqi Fuzheng Injection+OX | 0 | . | . | . |  |  |  |  |  | -6.06 [ -14.34; 2.22] | Low | Very low | Not serious | Very low | -6.06 [ -14.34; 2.22] | Very low | . | Serious | Very low | NMA estimate was used because incoherence was not available. Imprecision was rated as Serious. |
| Kanglixin Capsules+OX | Xiaoaiping Injection+OX | 0 | . | . | . |  |  |  |  |  | -10.71 [ -21.52; 0.11] | Low | Very low | Not serious | Very low | -10.71 [ -21.52; 0.11] | Very low | . | Serious | Very low | NMA estimate was used because incoherence was not available. Imprecision was rated as Serious. |
| Kanglixin Capsules+OX | Xihuang Capsules+OX | 0 | . | . | . |  |  |  |  |  | -5.89 [ -16.78; 5.01] | Low | Low | Not serious | Low | -5.89 [ -16.78; 5.01] | Low | . | Serious | Very low | NMA estimate was used because incoherence was not available. Imprecision was rated as Serious. |
| Kanglixin Capsules+OX | Ya Dan Zi Oil Emulsion Injection+OX | 0 | . | . | . |  |  |  |  |  | -3.55 [ -14.41; 7.31] | Low | Very low | Not serious | Very low | -3.55 [ -14.41; 7.31] | Very low | . | Serious | Very low | NMA estimate was used because incoherence was not available. Imprecision was rated as Serious. |
| Kanglixin Capsules+OX | Yangzheng Xiaoji Capsules+OX | 0 | . | . | . |  |  |  |  |  | 1.54 [ -11.65; 14.73] | Low | High | Not serious | Low | 1.54 [ -11.65; 14.73] | Low | . | Serious | Very low | NMA estimate was used because incoherence was not available. Imprecision was rated as Serious. |
| Kanglixin Capsules+OX | Zhenqi Fuzheng Granules+OX | 0 | . | . | . |  |  |  |  |  | -1.21 [ -14.44; 12.02] | Low | Low | Not serious | Low | -1.21 [ -14.44; 12.02] | Low | . | Serious | Very low | NMA estimate was used because incoherence was not available. Imprecision was rated as Serious. |
| Lentinan+OX | OX | 1 | 86 | . | -4.86 [ -15.66; 5.94] | Not serious | Not serious | Not serious | Undetected | High | . |  |  |  |  | -4.86 [ -15.66; 5.94] | High | . | Serious | Moderate | NMA estimate was used because incoherence was not available. Imprecision was rated as Serious. |
| Lentinan+OX | Pingxiao Capsules+OX | 0 | . | . | . |  |  |  |  |  | -5.36 [ -20.55; 9.83] | High | Low | Not serious | Low | -5.36 [ -20.55; 9.83] | Low | . | Serious | Very low | NMA estimate was used because incoherence was not available. Imprecision was rated as Serious. |
| Lentinan+OX | Shenmai Injection+OX | 0 | . | . | . |  |  |  |  |  | 40.14 [ -53.64; 133.92] | High | Low | Not serious | Low | 40.14 [ -53.64; 133.92] | Low | . | Serious | Very low | NMA estimate was used because incoherence was not available. Imprecision was rated as Serious. |
| Lentinan+OX | Shenqi Fuzheng Injection+OX | 0 | . | . | . |  |  |  |  |  | -4.91 [ -16.16; 6.33] | High | Very low | Not serious | Very low | -4.91 [ -16.16; 6.33] | Very low | . | Serious | Very low | NMA estimate was used because incoherence was not available. Imprecision was rated as Serious. |
| Lentinan+OX | Xiaoaiping Injection+OX | 0 | . | . | . |  |  |  |  |  | -9.56 [ -22.78; 3.67] | High | Very low | Not serious | Very low | -9.56 [ -22.78; 3.67] | Very low | . | Serious | Very low | NMA estimate was used because incoherence was not available. Imprecision was rated as Serious. |
| Lentinan+OX | Xihuang Capsules+OX | 0 | . | . | . |  |  |  |  |  | -4.73 [ -18.02; 8.55] | High | Low | Not serious | Low | -4.73 [ -18.02; 8.55] | Low | . | Serious | Very low | NMA estimate was used because incoherence was not available. Imprecision was rated as Serious. |
[truncated: 264,115 more chars]
