# Supplementary material for: Efficacy and safety of commercial Chinese polyherbal preparation combined with oxaliplatin-based chemotherapy for gastric cancer: a systematic review and network meta-analysis
Source: Front Pharmacol. 2025 Sep 9;16:1645079. doi: 10.3389/fphar.2025.1645079 (PMC12455072; doi:10.3389/fphar.2025.1645079)

# **Appendix 9 Summary of results for all outcomes**

| Outcomes | Certainty of Evidence | **Classification** | **Intervention** | **OR/MD (95% CrI) vs OX** |
| --- | --- | --- | --- | --- |
| Disease control rate | Higher (Moderate to High) | Among the most effective | Compound Mylabris preparations+OX | 1.51 (1.13 - 2.03) |
|  |  | Inferior to the most effective / superior to the least effective | Huachansu preparations+OX | 1.08 (1.01 - 1.14) |
|  |  |  | Kangai Injection+OX | 1.20 (1.11 - 1.29) |
|  |  |  | Shenqi Fuzheng Injection+OX | 1.11 (1.06 - 1.16) |
|  |  |  | Xiaoaiping Injection+OX | 1.16 (1.06 - 1.28) |
|  |  |  | Ya Dan Zi Oil Emulsion Injection+OX | 1.15 (1.07 - 1.24) |
|  |  |  | Yangzheng Xiaoji Capsules+OX | 1.17 (1.04 - 1.31) |
|  |  | Among the least effective | Kanglixin Capsules+OX | 1.11 (0.98 - 1.26) |
|  |  |  | Lentinan+OX | 1.04 (0.95 - 1.15) |
|  |  |  | Shenmai Injection+OX | 1.04 (0.93 - 1.17) |
|  |  |  | Jinlong Capsules+OX | 1.17 (0.94 - 1.45) |
|  |  |  | Ginseng Polysaccharide Injection+OX | 0.98 (0.85 - 1.13) |
|  | Low (Low to Very low) | May be the most effective | Astragalus Polysaccharides+OX | 1.42 (1.15 - 1.76) |
|  |  |  | Astragalus preparations+OX | 1.15 (1.02 - 1.29) |
|  |  |  | Compound Kushen Injection+OX | 1.10 (1.04 - 1.16) |
|  |  |  | Qizhen Capsule+OX | 1.21 (1.00 - 1.46) |
|  |  |  | Shenlian Capsule+OX | 1.53 (1.07 - 2.19) |
|  |  |  | Xihuang Capsules+OX | 1.39 (1.10 - 1.76) |
|  |  | May be the least effective | Aidi Injection+OX | 1.05 (0.99 - 1.12) |
|  |  |  | Huai'er Granules+OX | 0.96 (0.76 - 1.22) |
|  |  |  | Kanglaite Injection+OX | 1.10 (0.95 - 1.28) |
|  |  |  | Pingxiao Capsules+OX | 1.10 (0.97 - 1.25) |
|  |  |  | Shenfu Injection+OX | 0.98 (0.83 - 1.16) |
|  |  |  | Shengxue Granules+OX | 0.78 (0.64 - 0.95) |
|  |  |  | Zhenqi Fuzheng Granules+OX | 1.16 (0.97 - 1.38) |
| Objective response rate | Higher (Moderate to High) | Among the most effective | / | / |
|  |  | Inferior to the most effective / superior to the least effective | Huachansu preparations+OX | 1.28 (1.15 - 1.43) |
|  |  |  | Kangai Injection+OX | 1.40 (1.23 - 1.60) |
|  |  |  | Kanglixin Capsules+OX | 1.35 (1.08 - 1.70) |
|  |  |  | Yangzheng Xiaoji Capsules+OX | 1.36 (1.05 - 1.77) |
|  |  | Among the least effective | Ginseng Polysaccharide Injection+OX | 1.13 (0.75 - 1.69) |
|  |  |  | Jinlong Capsules+OX | 1.33 (1.00 - 1.75) |
|  |  |  | Lentinan+OX | 0.96 (0.80 - 1.15) |
|  |  |  | Shenmai Injection+OX | 1.30 (0.96 - 1.76) |
|  |  |  | Weimaining Capsules+OX | 1.25 (0.56 - 2.78) |
|  | Low (Low to Very low) | May be the most effective | Aidi Injection+OX | 1.25 (1.14 - 1.37) |
|  |  |  | Astragalus Polysaccharides+OX | 1.50 (1.04 - 2.17) |
|  |  |  | Compound Kushen Injection+OX | 1.31 (1.20 - 1.42) |
|  |  |  | Compound Mylabris preparations+OX | 1.39 (1.17 - 1.65) |
|  |  |  | Diyu Shengbai Tablets+OX | 1.51 (1.08 - 2.11) |
|  |  |  | Pingxiao Capsules+OX | 1.54 (1.13 - 2.11) |
|  |  |  | Qizhen Capsule+OX | 1.42 (1.04 - 1.94) |
|  |  |  | Shengxue Granules+OX | 1.47 (1.08 - 2.00) |
|  |  |  | Xiaoaiping Injection+OX | 1.33 (1.15 - 1.54) |
|  |  |  | Shenqi Fuzheng Injection+OX | 1.28 (1.17 - 1.40) |
|  |  |  | Xihuang Capsules+OX | 1.58 (1.12 - 2.23) |
|  |  |  | Ya Dan Zi Oil Emulsion Injection+OX | 1.33 (1.16 - 1.53) |
|  |  |  | Zhenqi Fuzheng Granules+OX | 1.63 (1.07 - 2.50) |
|  |  | May be the least effective | Astragalus preparations+OX | 1.16 (0.94 - 1.43) |
|  |  |  | Huai Er Granules+OX | 1.10 (0.54 - 2.23) |
|  |  |  | Kanglaite Injection+OX | 1.26 (0.94 - 1.69) |
|  |  |  | Shenfu Injection+OX | 0.95 (0.64 - 1.41) |
|  |  |  | Shenlian Capsules+OX | 1.73 (0.98 - 3.03) |
|  |  |  | Compound Tianxian Capsules+OX | 1.73 (0.98 - 3.03) |
| Improvement rate of QOL | Higher (Moderate to High) | Among the most effective | / | / |
|  |  | Inferior to the most effective / superior to the least effective | Aidi Injection+OX | 1.30 (1.13 - 1.50) |
|  |  |  | Lentinan+OX | 1.27 (1.05 - 1.54) |
|  |  |  | Kangai Injection+OX | 1.46 (1.11 - 1.94) |
|  |  |  | Ya Dan Zi Oil Emulsion Injection+OX | 1.26 (1.06 - 1.51) |
|  |  | Among the least effective | Compound Mylabris preparations+OX | 1.30 (0.97 - 1.74) |
|  |  |  | Shenmai Injection+OX | 1.20 (0.91 - 1.58) |
|  |  |  | Kanglaite Injection+OX | 1.23 (0.96 - 1.57) |
|  | Low (Low to Very low) | May be the most effective | Astragalus Polysaccharides+OX | 1.68 (1.24 - 2.28) |
|  |  |  | Astragalus preparations+OX | 2.18 (1.32 - 3.61) |
|  |  |  | Compound Kushen Injection+OX | 1.20 (1.01 - 1.42) |
|  |  |  | Shenqi Fuzheng Injection+OX | 1.30 (1.15 - 1.47) |
|  |  |  | Yangzheng Xiaoji Capsules+OX | 1.63 (1.20 - 2.21) |
|  |  | May be the least effective | Xiaoaiping Injection+OX | 1.18 (0.82 - 1.71) |
|  |  |  | Huachansu preparations+OX | 1.18 (0.91 - 1.51) |
|  |  |  | Pingxiao Capsules+OX | 1.36 (0.90 - 2.05) |
|  |  |  | Shenfu Injection+OX | 1.03 (0.75 - 1.42) |
| 1-year overall survival rate | Higher (Moderate to High) | Among the most effective | / | / |
|  |  | Inferior to the most effective / superior to the least effective | / | / |
|  |  | Among the least effective | / | / |
|  | Low (Low to Very low) | May be the most effective | / | / |
|  |  | May be the least effective | Aidi Injection+OX | 1.27 (0.98 - 1.65) |
|  |  |  | Shenqi Fuzheng Injection+OX | 1.37 (0.70 - 2.68) |
|  |  |  | Xiaoaiping Injection+OX | 1.17 (0.45 - 3.04) |
|  |  |  | Xihuang Capsules+OX | 1.08 (0.86 - 1.35) |
|  |  |  | Ya Dan Zi Oil Emulsion Injection+OX | c |
| 2-year overall survival rate | Higher (Moderate to High) | Among the most effective | / | / |
|  |  | Inferior to the most effective / superior to the least effective | / | / |
|  |  | Among the least effective | Shenqi Fuzheng Injection+OX | 1.49 (0.99 - 2.24) |
|  | Low (Low to Very low) | May be the most effective | / | // |
|  |  | May be the least effective | Xihuang Capsules+OX | 2.20 (1.30 - 3.73) |
|  |  |  | Ya Dan Zi Oil Emulsion Injection+OX | 1.40 (0.50 - 3.95) |
| TCM scores | Higher (Moderate to High) | Among the most effective | / | / |
|  |  | Inferior to the most effective / superior to the least effective | / | / |
|  |  | Among the least effective | Yangzheng Xiaoji Capsules+OX | -1.21 (-4.36 - 1.94) |
|  | Low (Low to Very low) | May be the most effective | Compound Kushen Injection+OX | -3.76 (-6.12 - -1.41) |
|  |  | May be the least effective | Aidi Injection+OX | -2.69 (-6.05 - 0.67) |
|  |  |  | Shenqi Fuzheng Injection+OX | 1.65 (-0.29 - 3.60) |
| CD3+ | Higher (Moderate to High) | Among the most effective | / | / |
|  |  | Inferior to the most effective / superior to the least effective | Ginseng Polysaccharide Injection+OX | 10.55 ( 1.89 - 19.21) |
|  |  |  | Huachansu preparations+OX | 7.42 ( 2.51 - 12.34) |
|  |  |  | Kangai Injection+OX | 11.65 ( 6.81 - 16.50) |
|  |  |  | Shenmai Injection+OX | 10.03 ( 1.69 - 18.37) |
|  |  |  | Yangzheng Xiaoji Capsules+OX | 9.32 ( 0.84 - 17.80) |
|  |  | Among the least effective | / | / |
|  | Low (Low to Very low) | May be the most effective | Aidi Injection+OX | 11.09 ( 6.67 - 15.51) |
|  |  |  | Astragalus Polysaccharides+OX | 9.01 ( 2.99 - 15.03) |
|  |  |  | Compound Kushen Injection+OX | 9.34 ( 6.97 - 11.72) |
|  |  |  | Compound Mylabris preparations+OX | 5.29 ( 1.06 - 9.53) |
|  |  |  | Jinlong Capsules+OX | 8.28 ( 3.19 - 13.38) |
|  |  |  | Kanglixin Capsules+OX | 9.30 ( 3.33 - 15.28) |
|  |  |  | Lentinan+OX | 8.18 ( 2.17 - 14.18) |
|  |  |  | Pingxiao Capsules+OX | 12.47 ( 3.84 - 21.10) |
|  |  |  | Shenqi Fuzheng Injection+OX | 7.83 ( 5.09 - 10.56) |
|  |  |  | Xiaoaiping Injection+OX | 13.85 ( 7.58 - 20.11) |
|  |  |  | Xihuang Capsules+OX | 11.04 ( 4.98 - 17.10) |
|  |  |  | Zhenqi Fuzheng Granules+OX | 11.10 ( 2.62 - 19.58) |
|  |  |  | Ya Dan Zi Oil Emulsion Injection+OX | 7.94 ( 1.67 - 14.20) |
|  |  | May be the least effective | Astragalus preparations+OX | 7.50 ( -0.87 - 15.87) |
| CD4+T cells | Higher (Moderate to High) | Among the most effective | / | / |
|  |  | Inferior to the most effective / superior to the least effective | Lentinan+OX | 9.43 ( 3.78 - 15.08) |
|  |  |  | Shenmai Injection+OX | 8.33 ( 0.64 - 16.02) |
|  |  |  | Shenqi Fuzheng Injection+OX | 5.72 ( 3.68 - 7.76) |
|  |  | Among the least effective | Yangzheng Xiaoji Capsules+OX | 5.11 ( -2.74 - 12.96) |
|  | Low (Low to Very low) | May be the most effective | Aidi Injection+OX | 7.89 ( 4.55 - 11.24) |
|  |  |  | Astragalus Polysaccharides+OX | 11.82 ( 6.23 - 17.42) |
|  |  |  | Compound Kushen Injection+OX | 7.24 ( 5.05 - 9.42) |
|  |  |  | Compound Mylabris preparations+OX | 5.17 ( 1.26 - 9.08) |
|  |  |  | Diyu Shengbai Tablet+OX | 9.50 ( 1.79 - 17.21) |
|  |  |  | Jinlong Capsules+OX | 5.43 ( 1.26 - 9.61) |
|  |  |  | Kangai Injection+OX | 8.58 ( 4.66 - 12.49) |
|  |  |  | Kanglixin Capsules+OX | 8.16 ( 2.59 - 13.73) |
|  |  |  | Xiaoaiping Injection+OX | 9.84 ( 4.31 - 15.37) |
|  |  |  | Zhenqi Fuzheng Granules+OX | 9.10 ( 1.14 - 17.06) |
|  |  | May be the least effective | Astragalus preparations+OX | 2.40 ( -2.12 - 6.92) |
|  |  |  | Ginseng Polysaccharide Injection+OX | 4.83 ( -3.54 - 13.20) |
|  |  |  | Pingxiao Capsules+OX | 7.23 ( -0.64 - 15.10) |
|  |  |  | Xihuang Capsules+OX | 4.44 ( -1.14 - 10.03) |
|  |  |  | Ya Dan Zi Oil Emulsion Injection+OX | 5.24 ( -0.48 - 10.96) |
| CD8+T cells | Higher (Moderate to High) | Among the most effective | / | / |
|  |  | Inferior to the most effective / superior to the least effective | / | / |
|  |  | Among the least effective | Ginseng Polysaccharide Injection+OX | 0.11 ( -10.68 - 10.90) |
|  |  |  | Jinlong Capsules+OX | -5.16 ( -11.54 - 1.21) |
|  |  |  | Lentinan+OX | -4.86 ( -15.66 - 5.94) |
|  |  |  | Yangzheng Xiaoji Capsules+OX | -7.55 ( -18.29 - 3.19) |
|  | Low (Low to Very low) | May be the most effective | / | / |
|  |  | May be the least effective | Aidi Injection+OX | -1.08 ( -6.01 - 3.86) |
|  |  |  | Astragalus Polysaccharides+OX | -3.99 ( -11.61 - 3.63) |
|  |  |  | Astragalus preparations+OX | -1.44 ( -7.66 - 4.78) |
|  |  |  | Compound Kushen Injection+OX | -3.64 ( -6.75 - -0.53) |
|  |  |  | Compound Mylabris preparations+OX | -0.54 ( -6.75 - 5.67) |
|  |  |  | Diyu Shengbai Tablet+OX | -6.82 ( -17.53 - 3.89) |
|  |  |  | Huachansu preparations+OX | 0.46 ( -4.94 - 5.86) |
|  |  |  | Kangai Injection+OX | -0.23 ( -5.62 - 5.17) |
|  |  |  | Kanglixin Capsules+OX | -6.01 ( -13.67 - 1.65) |
|  |  |  | Pingxiao Capsules+OX | 0.50 ( -10.18 - 11.18) |
|  |  |  | Shenmai Injection+OX | -45.00 (-138.16 - 48.16) |
|  |  |  | Shenqi Fuzheng Injection+OX | 0.05 ( -3.09 - 3.19) |
|  |  |  | Xiaoaiping Injection+OX | 4.70 ( -2.93 - 12.33) |
|  |  |  | Xihuang Capsules+OX | -0.13 ( -7.87 - 7.62) |
|  |  |  | Ya Dan Zi Oil Emulsion Injection+OX | -2.46 ( -10.15 - 5.24) |
|  |  |  | Zhenqi Fuzheng Granules+OX | -4.80 ( -15.59 - 5.99) |
| CD4+/CD8+ratio | Higher (Moderate to High) | Among the most effective | / | / |
|  |  | Inferior to the most effective / superior to the least effective | Kangai Injection+OX | 0.25 ( 0.02 - 0.47) |
|  |  | Among the least effective | Yangzheng Xiaoji Capsules+OX | 0.35 (-0.03 - 0.73) |
|  | Low (Low to Very low) | May be the most effective | Aidi Injection+OX | 0.45 ( 0.14 - 0.76) |
|  |  |  | Astragalus Polysaccharides+OX | 0.62 ( 0.34 - 0.89) |
|  |  |  | Astragalus preparations+OX | 0.38 ( 0.16 - 0.61) |
|  |  |  | Compound Kushen Injection+OX | 0.47 ( 0.36 - 0.58) |
|  |  |  | Diyu Shengbai Tablet+OX | 0.66 ( 0.28 - 1.04) |
|  |  |  | Huachansu preparations+OX | 0.47 ( 0.26 - 0.68) |
|  |  |  | Kanglixin Capsules+OX | 0.63 ( 0.36 - 0.91) |
|  |  |  | Lentinan+OX | 0.45 ( 0.17 - 0.74) |
|  |  |  | Shenmai Injection+OX | 0.35 ( 0.13 - 0.56) |
|  |  |  | Shenqi Fuzheng Injection+OX | 0.31 ( 0.20 - 0.43) |
|  |  |  | Xiaoaiping Injection+OX | 0.28 ( 0.01 - 0.56) |
|  |  |  | Ya Dan Zi Oil Emulsion Injection+OX | 0.57 ( 0.26 - 0.88) |
|  |  |  | Zhenqi Fuzheng Granules+OX | 0.82 ( 0.44 - 1.20) |
|  |  |  | Jinlong Capsules+OX | 0.41 ( 0.20 - 0.61) |
|  |  | May be the least effective | Xihuang Capsules+OX | 0.12 (-0.16 - 0.41) |
|  |  |  | Pingxiao Capsules+OX | -6.36 (-8.18 - -4.54) |
|  |  |  | Ginseng Polysaccharide Injection+OX | 0.24 (-0.17 - 0.65) |
|  |  |  | Compound Mylabris preparations+OX | 0.15 (-0.07 - 0.36) |
| NK cell | Higher (Moderate to High) | Among the most effective | / | / |
|  |  | Inferior to the most effective / superior to the least effective | Compound Kushen Injection+OX | 4.84 ( 1.57 - 8.11) |
|  |  |  | Huachansu preparations+OX | 4.55 ( 1.31 - 7.78) |
|  |  |  | Jinlong Capsules+OX | 9.11 ( 5.57 - 12.65) |
|  |  | Among the least effective | Ginseng Polysaccharide Injection+OX | 1.27 ( -4.52 - 7.06) |
|  |  |  | Lentinan+OX | 1.88 ( -3.81 - 7.57) |
|  | Low (Low to Very low) | May be the most effective | Astragalus preparations+OX | 6.00 ( 0.40 - 11.60) |
|  |  |  | Kangai Injection+OX | 20.76 ( 15.13 - 26.39) |
|  |  |  | Shenqi Fuzheng Injection+OX | 5.22 ( 2.37 - 8.07) |
|  |  | May be the least effective | Aidi Injection+OX | 2.66 ( -2.76 - 8.08) |
|  |  |  | Astragalus Polysaccharides+OX | -1.29 ( -5.24 - 2.66) |
| CEA | Higher (Moderate to High) | Among the most effective | / | / |
|  |  | Inferior to the most effective / superior to the least effective | Shenmai Injection+OX | -7.83 (-15.62 - -0.04) |
|  |  | Among the least effective | Ya Dan Zi Oil Emulsion Injection+OX | -3.28 (-10.15 - 3.59) |
|  | Low (Low to Very low) | May be the most effective | Aidi Injection+OX | -6.15 ( -9.34 - -2.96) |
|  |  |  | Compound Kushen Injection+OX | -7.54 (-10.05 - -5.03) |
|  |  |  | Compound Mylabris preparations+OX | -5.68 ( -9.53 - -1.83) |
|  |  |  | Kangai Injection+OX | -8.08 (-12.91 - -3.24) |
|  |  |  | Kanglaite Injection+OX | -18.20 (-25.91 - -10.49) |
|  |  |  | Kanglixin Capsules+OX | -15.22 (-20.76 - -9.67) |
|  |  |  | Shengxue Granules+OX | -16.43 (-24.09 - -8.77) |
|  |  |  | Shenqi Fuzheng Injection+OX | -9.87 (-13.58 - -6.16) |
|  |  | May be the least effective | Astragalus preparations+OX | -3.62 (-11.25 - 4.01) |
|  |  |  | Huachansu preparations+OX | -2.83 ( -8.01 - 2.35) |
|  |  |  | Huai'er Granules+OX | -3.24 (-11.21 - 4.73) |
|  |  |  | Shenlian Capsule+OX | -15.39 (-33.33 - 2.55) |
| CA125 | Higher (Moderate to High) | Among the least effective | / | / |
|  |  | Inferior to the most effective / superior to the least effective | / | / |
|  |  | Among the least effective | Yangzheng Xiaoji Capsules+OX | -1.21 (-4.36 - 1.94) |
|  | Low (Low to Very low) | May be the most effective | Compound Kushen Injection+OX | -3.76 (-6.12 - -1.41) |
|  |  | May be the least effective | Aidi Injection+OX | -2.69 (-6.05 - 0.67) |
|  |  |  | Shenqi Fuzheng Injection+OX | 1.65 (-0.29 - 3.60) |
| CA199 | Higher (Moderate to High) | Among the most effective | Kanglixin Capsules+OX | -33.25 (-45.58 - -20.91) |
|  |  | Inferior to the most effective / superior to the least effective | Shenqi Fuzheng Injection+OX | -15.07 (-27.20 - -2.94) |
|  |  | Among the least effective | Kangai Injection+OX | -15.35 (-31.43 - 0.73) |
|  | Low (Low to Very low) | May be the most effective | Aidi Injection+OX | -12.40 (-21.52 - -3.27) |
|  |  |  | Compound Kushen Injection+OX | -16.09 (-23.81 - -8.36) |
|  |  |  | Compound Mylabris preparations+OX | -24.90 (-36.79 - -13.02) |
|  |  |  | Shenlian Capsule+OX | -21.56 (-42.10 - -1.02) |
|  |  | May be the least effective | Huachansu preparations+OX | -7.92 (-22.37 - 6.52) |
|  |  |  | Huai'er Granules+OX | -6.08 (-26.96 - 14.80) |
|  |  |  | Kanglaite Injection+OX | -3.21 (-27.02 - 20.60) |
| CA724 |  |  | Shengxue Granules+OX | -15.75 (-40.07 - 8.57) |
|  | Higher (Moderate to High) | Among the most effective | / | / |
|  |  | Inferior to the most effective / superior to the least effective | Kanglixin Capsules+OX | -32.09 (-42.20 - -21.97) |
|  |  | Among the least effective | Shenqi Fuzheng Injection+OX | -5.98 (-17.48 - 5.52) |
|  | Low (Low to Very low) | May be the most effective | Aidi Injection+OX | -14.19 (-21.54 - -6.84) |
|  |  |  | Compound Kushen Injection+OX | -12.52 (-17.92 - -7.11) |
|  |  | May be the least effective | Compound Mylabris preparations+OX | -8.92 (-19.94 - 2.10) |
|  |  |  | Kangai Injection+OX | -5.86 (-13.67 - 1.95) |
|  |  |  | Shengxue Granules+OX | -4.56 (-15.37 - 6.25) |
|  |  |  | Shenlian Capsule+OX | -7.74 (-18.63 - 3.15) |
| Gastrointestinal reaction | Higher (Moderate to High) | Less harmful than OX alone | Kangai Injection+OX | 0.65 (0.54 - 0.79) |
|  |  |  | Shenqi Fuzheng Injection+OX | 0.60 (0.50 - 0.73) |
|  |  |  | Lentinan+OX | 0.63 (0.43 - 0.92) |
|  |  |  | Yangzheng Xiaoji Capsules+OX | 0.52 (0.37 - 0.73) |
|  |  | No more harmful than OX alone | Kanglixin Capsules+OX | 0.95 (0.51 - 1.75) |
|  |  |  | Shenmai Injection+OX | 0.75 (0.49 - 1.15) |
|  |  | More harmful than OX alone | Ginseng Polysaccharide Injection+OX | 3.96 (1.51 - 10.40) |
|  | Low (Low to Very low) | Less harmful than OX alone | Aidi Injection+OX | 0.56 (0.45 - 0.68) |
|  |  |  | Astragalus Polysaccharides+OX | 0.42 (0.20 - 0.87) |
|  |  |  | Compound Kushen Injection+OX | 0.74 (0.63 - 0.88) |
|  |  |  | Compound Mylabris preparations+OX | 0.78 (0.62 - 0.99) |
|  |  |  | Huachansu preparations+OX | 0.75 (0.60 - 0.94) |
|  |  | No more harmful than OX alone | Astragalus preparations+OX | 0.55 (0.28 - 1.06) |
|  |  |  | Diyu Shengbai Tablets+OX | 0.42 (0.16 - 1.12) |
|  |  |  | Huai'er Granules+OX | 0.86 (0.57 - 1.29) |
|  |  |  | Jinlong Capsules+OX | 0.76 (0.50 - 1.16) |
|  |  |  | Kanglaite Injection+OX | 0.82 (0.63 - 1.07) |
|  |  |  | Qizhen Capsule+OX | 0.77 (0.41 - 1.45) |
|  |  |  | Shenfu Injection+OX | 0.55 (0.26 - 1.15) |
|  |  |  | Shengxue Granules+OX | 0.40 (0.08 - 2.10) |
|  |  |  | Shenlian Capsule+OX | 0.67 (0.11 - 3.93) |
|  |  |  | Xiaoaiping Injection+OX | 0.90 (0.71 - 1.14) |
|  |  |  | Xihuang Capsules+OX | 1.17 (0.53 - 2.55) |
|  |  |  | Ya Dan Zi Oil Emulsion Injection+OX | 0.78 (0.56 - 1.09) |
|  |  | More harmful than OX alone | / | / |
| Myelosuppression | Higher (Moderate to High) | Less harmful than OX alone | Kangai Injection+OX | 0.52 (0.39 - 0.69) |
|  |  |  | Lentinan+OX | 0.48 (0.28 - 0.82) |
|  |  |  | Yangzheng Xiaoji Capsules+OX | 0.61 (0.38 - 0.97) |
|  |  | No more harmful than OX alone | Ginseng Polysaccharide Injection+OX | 0.51 (0.26 - 1.01) |
|  |  |  | Shenmai Injection+OX | 0.58 (0.30 - 1.09) |
|  |  | More harmful than OX alone | / | / |
|  | Low (Low to Very low) | Less harmful than OX alone | Aidi Injection+OX | 0.65 (0.54 - 0.80) |
|  |  |  | Astragalus Polysaccharides+OX | 0.44 (0.20 - 0.96) |
|  |  |  | Compound Kushen Injection+OX | 0.63 (0.52 - 0.75) |
|  |  |  | Huachansu preparations+OX | 0.70 (0.54 - 0.91) |
|  |  |  | Huai'er Granules+OX | 0.62 (0.41 - 0.94) |
|  |  |  | Shenqi Fuzheng Injection+OX | 0.55 (0.45 - 0.67) |
|  |  |  | Ya Dan Zi Oil Emulsion Injection+OX | 0.70 (0.51 - 0.97) |
|  |  |  | Zhenqi Fuzheng Granules+OX | 0.38 (0.16 - 0.90) |
|  |  | No more harmful than OX alone | Astragalus preparations+OX | 0.57 (0.29 - 1.15) |
|  |  |  | Compound Mylabris preparations+OX | 0.74 (0.54 - 1.00) |
|  |  |  | Jinlong Capsules+OX | 0.94 (0.67 - 1.32) |
|  |  |  | Kanglaite Injection+OX | 0.82 (0.57 - 1.18) |
|  |  |  | Kanglixin Capsules+OX | 0.83 (0.50 - 1.40) |
|  |  |  | Qizhen Capsule+OX | 0.75 (0.37 - 1.54) |
|  |  |  | Shenfu Injection+OX | 0.97 (0.48 - 1.94) |
|  |  |  | Shengxue Granules+OX | 0.25 (0.03 - 2.29) |
|  |  |  | Shenlian Capsule+OX | 0.67 (0.11 - 3.97) |
|  |  |  | Xiaoaiping Injection+OX | 0.88 (0.67 - 1.15) |
|  |  |  | Xihuang Capsules+OX | 0.59 (0.29 - 1.21) |
|  |  | More harmful than OX alone | / | / |

# **Appendix 10 Results of random model for league table**

1. Disease Control Rate

| Aidi Injection+OX | . | . | . | . | . | . | . | . | . | . | . | . | . | . | . | . | . | . | . | . | . | . | . | . | 1.05 (0.99 - 1.12) |
| --- | --- | --- | --- | --- | --- | --- | --- | --- | --- | --- | --- | --- | --- | --- | --- | --- | --- | --- | --- | --- | --- | --- | --- | --- | --- |
| 0.74 (0.59 - 0.92) | AP+OX | . | . | . | . | . | . | . | . | . | . | . | . | . | . | . | . | . | . | . | . | . | . | . | 1.42 (1.15 - 1.76) |
| 0.92 (0.80 - 1.05) | 1.24 (0.97 - 1.58) | Astragalus preparations | . | . | . | . | . | . | . | . | . | . | . | . | . | . | . | . | . | . | . | . | . | . | 1.15 (1.02 - 1.29) |
| 0.96 (0.88 - 1.04) | 1.30 (1.04 - 1.62) | 1.05 (0.92 - 1.19) | CKI+OX | . | . | . | . | . | . | . | . | . | . | . | . | . | . | . | . | . | . | . | . | . | 1.10 (1.04 - 1.16) |
| 0.70 (0.51 - 0.94) | 0.94 (0.65 - 1.36) | 0.76 (0.55 - 1.04) | 0.72 (0.54 - 0.98) | CMP+OX | . | . | . | . | . | . | . | . | . | . | . | . | . | . | . | . | . | . | . | . | 1.51 (1.13 - 2.03) |
| 1.07 (0.92 - 1.25) | 1.45 (1.13 - 1.88) | 1.17 (0.97 - 1.41) | 1.12 (0.96 - 1.30) | 1.54 (1.11 - 2.14) | GPI+OX | . | . | . | . | . | . | . | . | . | . | . | . | . | . | . | . | . | . | . | 0.98 (0.85 - 1.13) |
| 0.98 (0.90 - 1.07) | 1.32 (1.06 - 1.65) | 1.07 (0.94 - 1.22) | 1.02 (0.94 - 1.11) | 1.41 (1.04 - 1.90) | 0.91 (0.78 - 1.06) | HCSP+OX | . | . | . | . | . | . | . | . | . | . | . | . | . | . | . | . | . | . | 1.08 (1.01 - 1.14) |
| 1.09 (0.86 - 1.40) | 1.48 (1.08 - 2.04) | 1.19 (0.92 - 1.55) | 1.14 (0.90 - 1.45) | 1.57 (1.08 - 2.30) | 1.02 (0.77 - 1.34) | 1.12 (0.88 - 1.43) | HEG+OX | . | . | . | . | . | . | . | . | . | . | . | . | . | . | . | . | . | 0.96 (0.76 - 1.22) |
| 0.90 (0.72 - 1.13) | 1.22 (0.90 - 1.65) | 0.98 (0.77 - 1.26) | 0.94 (0.75 - 1.18) | 1.30 (0.90 - 1.87) | 0.84 (0.65 - 1.09) | 0.92 (0.74 - 1.16) | 0.82 (0.60 - 1.13) | JLC+OX | . | . | . | . | . | . | . | . | . | . | . | . | . | . | . | . | 1.17 (0.94 - 1.45) |
| 0.88 (0.80 - 0.97) | 1.19 (0.95 - 1.49) | 0.96 (0.83 - 1.10) | 0.91 (0.83 - 1.00) | 1.26 (0.93 - 1.71) | 0.82 (0.70 - 0.96) | 0.90 (0.81 - 0.99) | 0.80 (0.63 - 1.03) | 0.97 (0.77 - 1.22) | KAI+OX | . | . | . | . | . | . | . | . | . | . | . | . | . | . | . | 1.20 (1.11 - 1.29) |
| 0.96 (0.81 - 1.12) | 1.29 (1.00 - 1.68) | 1.04 (0.86 - 1.26) | 1.00 (0.85 - 1.17) | 1.37 (0.99 - 1.91) | 0.89 (0.72 - 1.09) | 0.98 (0.83 - 1.15) | 0.87 (0.66 - 1.15) | 1.06 (0.81 - 1.38) | 1.09 (0.92 - 1.29) | KLI+OX | . | . | . | . | . | . | . | . | . | . | . | . | . | . | 1.10 (0.95 - 1.28) |
| 0.95 (0.82 - 1.09) | 1.28 (1.00 - 1.64) | 1.03 (0.87 - 1.22) | 0.98 (0.86 - 1.13) | 1.36 (0.99 - 1.88) | 0.88 (0.73 - 1.07) | 0.97 (0.84 - 1.11) | 0.86 (0.66 - 1.13) | 1.05 (0.82 - 1.35) | 1.08 (0.93 - 1.25) | 0.99 (0.81 - 1.20) | KLC+OX | . | . | . | . | . | . | . | . | . | . | . | . | . | 1.11 (0.98 - 1.26) |
| 1.01 (0.90 - 1.13) | 1.36 (1.08 - 1.73) | 1.10 (0.94 - 1.28) | 1.05 (0.94 - 1.18) | 1.45 (1.06 - 1.98) | 0.94 (0.79 - 1.12) | 1.03 (0.92 - 1.16) | 0.92 (0.71 - 1.19) | 1.12 (0.88 - 1.42) | 1.15 (1.02 - 1.30) | 1.05 (0.88 - 1.26) | 1.07 (0.91 - 1.25) | Lentinan+OX | . | . | . | . | . | . | . | . | . | . | . | . | 1.04 (0.95 - 1.15) |
| 0.95 (0.83 - 1.10) | 1.29 (1.01 - 1.65) | 1.04 (0.87 - 1.24) | 0.99 (0.86 - 1.14) | 1.37 (0.99 - 1.89) | 0.89 (0.73 - 1.07) | 0.97 (0.85 - 1.12) | 0.87 (0.67 - 1.14) | 1.06 (0.82 - 1.36) | 1.09 (0.94 - 1.26) | 1.00 (0.82 - 1.21) | 1.01 (0.84 - 1.21) | 0.95 (0.80 - 1.11) | PXC+OX | . | . | . | . | . | . | . | . | . | . | . | 1.10 (0.97 - 1.25) |
| 0.87 (0.72 - 1.06) | 1.18 (0.89 - 1.57) | 0.95 (0.76 - 1.19) | 0.91 (0.75 - 1.10) | 1.25 (0.88 - 1.78) | 0.81 (0.64 - 1.03) | 0.89 (0.73 - 1.09) | 0.80 (0.59 - 1.08) | 0.97 (0.73 - 1.29) | 0.99 (0.81 - 1.22) | 0.91 (0.72 - 1.16) | 0.92 (0.74 - 1.16) | 0.86 (0.70 - 1.07) | 0.91 (0.73 - 1.15) | QZC+OX | . | . | . | . | . | . | . | . | . | . | 1.21 (1.00 - 1.46) |
| 1.07 (0.90 - 1.28) | 1.45 (1.11 - 1.91) | 1.17 (0.95 - 1.44) | 1.12 (0.94 - 1.34) | 1.54 (1.10 - 2.17) | 1.00 (0.80 - 1.25) | 1.10 (0.92 - 1.31) | 0.98 (0.73 - 1.31) | 1.19 (0.90 - 1.57) | 1.22 (1.02 - 1.47) | 1.12 (0.90 - 1.41) | 1.14 (0.92 - 1.40) | 1.06 (0.87 - 1.30) | 1.13 (0.91 - 1.39) | 1.23 (0.96 - 1.58) | SFI+OX | . | . | . | . | . | . | . | . | . | 0.98 (0.83 - 1.16) |
| 1.35 (1.10 - 1.67) | 1.83 (1.37 - 2.46) | 1.48 (1.17 - 1.86) | 1.41 (1.14 - 1.74) | 1.95 (1.36 - 2.78) | 1.26 (0.98 - 1.61) | 1.38 (1.12 - 1.71) | 1.24 (0.91 - 1.68) | 1.50 (1.12 - 2.02) | 1.54 (1.25 - 1.91) | 1.42 (1.10 - 1.82) | 1.43 (1.13 - 1.82) | 1.34 (1.07 - 1.68) | 1.42 (1.12 - 1.80) | 1.55 (1.18 - 2.04) | 1.26 (0.97 - 1.64) | SXG+OX | . | . | . | . | . | . | . | . | 0.78 (0.64 - 0.95) |
| 0.69 (0.48 - 0.99) | 0.93 (0.61 - 1.41) | 0.75 (0.52 - 1.09) | 0.72 (0.50 - 1.03) | 0.99 (0.62 - 1.57) | 0.64 (0.44 - 0.94) | 0.70 (0.49 - 1.01) | 0.63 (0.41 - 0.96) | 0.76 (0.50 - 1.16) | 0.78 (0.54 - 1.13) | 0.72 (0.49 - 1.06) | 0.73 (0.50 - 1.06) | 0.68 (0.47 - 0.99) | 0.72 (0.49 - 1.05) | 0.79 (0.53 - 1.18) | 0.64 (0.43 - 0.95) | 0.51 (0.34 - 0.77) | SLC+OX | . | . | . | . | . | . | . | 1.53 (1.07 - 2.19) |
| 1.01 (0.89 - 1.15) | 1.36 (1.07 - 1.74) | 1.10 (0.93 - 1.29) | 1.05 (0.92 - 1.19) | 1.45 (1.06 - 1.99) | 0.94 (0.78 - 1.13) | 1.03 (0.90 - 1.17) | 0.92 (0.71 - 1.20) | 1.12 (0.87 - 1.43) | 1.15 (1.00 - 1.32) | 1.05 (0.87 - 1.27) | 1.07 (0.90 - 1.26) | 1.00 (0.86 - 1.16) | 1.06 (0.89 - 1.25) | 1.16 (0.93 - 1.44) | 0.94 (0.77 - 1.15) | 0.74 (0.59 - 0.94) | 1.46 (1.01 - 2.13) | SMI+OX | . | . | . | . | . | . | 1.04 (0.93 - 1.17) |
| 0.95 (0.88 - 1.02) | 1.28 (1.03 - 1.60) | 1.03 (0.91 - 1.17) | 0.99 (0.92 - 1.06) | 1.36 (1.01 - 1.84) | 0.88 (0.76 - 1.03) | 0.97 (0.90 - 1.05) | 0.87 (0.68 - 1.10) | 1.05 (0.84 - 1.31) | 1.08 (0.99 - 1.18) | 0.99 (0.85 - 1.16) | 1.00 (0.88 - 1.15) | 0.94 (0.84 - 1.05) | 1.00 (0.87 - 1.14) | 1.09 (0.90 - 1.32) | 0.88 (0.74 - 1.05) | 0.70 (0.57 - 0.86) | 1.38 (0.96 - 1.98) | 0.94 (0.83 - 1.07) | SQFZJ+OX | . | . | . | . | . | 1.11 (1.06 - 1.16) |
| 0.91 (0.81 - 1.01) | 1.23 (0.97 - 1.55) | 0.99 (0.85 - 1.15) | 0.94 (0.84 - 1.05) | 1.30 (0.96 - 1.78) | 0.84 (0.71 - 1.00) | 0.93 (0.83 - 1.04) | 0.83 (0.64 - 1.07) | 1.00 (0.79 - 1.27) | 1.03 (0.91 - 1.17) | 0.95 (0.79 - 1.13) | 0.96 (0.82 - 1.12) | 0.90 (0.78 - 1.03) | 0.95 (0.81 - 1.12) | 1.04 (0.84 - 1.28) | 0.84 (0.69 - 1.03) | 0.67 (0.54 - 0.84) | 1.32 (0.91 - 1.91) | 0.90 (0.77 - 1.04) | 0.95 (0.86 - 1.06) | XAPI+OX | . | . | . | . | 1.16 (1.06 - 1.28) |
| 0.76 (0.59 - 0.96) | 1.02 (0.75 - 1.41) | 0.83 (0.64 - 1.07) | 0.79 (0.62 - 1.00) | 1.09 (0.75 - 1.59) | 0.70 (0.54 - 0.93) | 0.77 (0.61 - 0.99) | 0.69 (0.50 - 0.96) | 0.84 (0.61 - 1.15) | 0.86 (0.67 - 1.10) | 0.79 (0.60 - 1.04) | 0.80 (0.61 - 1.04) | 0.75 (0.58 - 0.97) | 0.79 (0.61 - 1.04) | 0.87 (0.64 - 1.17) | 0.70 (0.53 - 0.94) | 0.56 (0.41 - 0.76) | 1.10 (0.72 - 1.69) | 0.75 (0.58 - 0.97) | 0.80 (0.63 - 1.01) | 0.84 (0.65 - 1.08) | XHC+OX | . | . | . | 1.39 (1.10 - 1.76) |
| 0.92 (0.83 - 1.01) | 1.24 (0.99 - 1.55) | 1.00 (0.87 - 1.15) | 0.95 (0.87 - 1.05) | 1.32 (0.97 - 1.79) | 0.85 (0.73 - 1.00) | 0.94 (0.85 - 1.03) | 0.84 (0.65 - 1.07) | 1.01 (0.81 - 1.28) | 1.04 (0.94 - 1.16) | 0.96 (0.81 - 1.13) | 0.97 (0.83 - 1.12) | 0.91 (0.80 - 1.03) | 0.96 (0.83 - 1.11) | 1.05 (0.86 - 1.28) | 0.85 (0.71 - 1.03) | 0.68 (0.55 - 0.84) | 1.33 (0.92 - 1.92) | 0.91 (0.79 - 1.04) | 0.96 (0.88 - 1.06) | 1.01 (0.89 - 1.14) | 1.21 (0.95 - 1.55) | YDZOI+OX | . | . | 1.15 (1.07 - 1.24) |
| 0.90 (0.79 - 1.03) | 1.22 (0.96 - 1.56) | 0.98 (0.83 - 1.16) | 0.94 (0.83 - 1.07) | 1.30 (0.94 - 1.78) | 0.84 (0.70 - 1.01) | 0.92 (0.81 - 1.05) | 0.82 (0.63 - 1.07) | 1.00 (0.78 - 1.28) | 1.03 (0.90 - 1.18) | 0.94 (0.78 - 1.14) | 0.95 (0.80 - 1.13) | 0.89 (0.77 - 1.04) | 0.95 (0.80 - 1.13) | 1.03 (0.83 - 1.29) | 0.84 (0.68 - 1.03) | 0.67 (0.53 - 0.84) | 1.31 (0.90 - 1.91) | 0.90 (0.76 - 1.05) | 0.95 (0.84 - 1.08) | 1.00 (0.86 - 1.16) | 1.19 (0.92 - 1.55) | 0.99 (0.86 - 1.13) | YZXJC+OX | . | 1.17 (1.04 - 1.31) |
| 0.91 (0.75 - 1.10) | 1.23 (0.93 - 1.62) | 0.99 (0.80 - 1.22) | 0.95 (0.79 - 1.14) | 1.31 (0.93 - 1.84) | 0.85 (0.67 - 1.06) | 0.93 (0.77 - 1.12) | 0.83 (0.62 - 1.11) | 1.01 (0.76 - 1.33) | 1.04 (0.86 - 1.26) | 0.95 (0.75 - 1.20) | 0.96 (0.77 - 1.20) | 0.90 (0.74 - 1.10) | 0.95 (0.77 - 1.19) | 1.04 (0.81 - 1.35) | 0.85 (0.66 - 1.08) | 0.67 (0.51 - 0.88) | 1.32 (0.89 - 1.97) | 0.90 (0.73 - 1.11) | 0.96 (0.80 - 1.15) | 1.00 (0.82 - 1.23) | 1.20 (0.90 - 1.61) | 0.99 (0.82 - 1.20) | 1.01 (0.81 - 1.25) | ZQFXG+OX | 1.16 (0.97 - 1.38) |
| 1.05 (0.99 - 1.12) | 1.42 (1.15 - 1.76) | 1.15 (1.02 - 1.29) | 1.10 (1.04 - 1.16) | 1.51 (1.13 - 2.03) | 0.98 (0.85 - 1.13) | 1.08 (1.01 - 1.14) | 0.96 (0.76 - 1.22) | 1.17 (0.94 - 1.45) | 1.20 (1.11 - 1.29) | 1.10 (0.95 - 1.28) | 1.11 (0.98 - 1.26) | 1.04 (0.95 - 1.15) | 1.10 (0.97 - 1.25) | 1.21 (1.00 - 1.46) | 0.98 (0.83 - 1.16) | 0.78 (0.64 - 0.95) | 1.53 (1.07 - 2.19) | 1.04 (0.93 - 1.17) | 1.11 (1.06 - 1.16) | 1.16 (1.06 - 1.28) | 1.39 (1.10 - 1.76) | 1.15 (1.07 - 1.24) | 1.17 (1.04 - 1.31) | 1.16 (0.97 - 1.38) | OX |

1. **Objective response rate**

| Aidi Injection+OX | . | . | . | . | . | . | . | . | . | . | . | . | . | . | . | . | . | . | . | . | . | . | . | 1.23 (1.12 - 1.36) |
| --- | --- | --- | --- | --- | --- | --- | --- | --- | --- | --- | --- | --- | --- | --- | --- | --- | --- | --- | --- | --- | --- | --- | --- | --- |
| 0.82 (0.57 - 1.19) | APs+OX | . | . | . | . | . | . | . | . | . | . | . | . | . | . | . | . | . | . | . | . | . | . | 1.50 (1.05 - 2.13) |
| 1.05 (0.86 - 1.29) | 1.27 (0.86 - 1.89) | Astragalus preparations+OX | . | . | . | . | . | . | . | . | . | . | . | . | . | . | . | . | . | . | . | . | . | 1.18 (0.99 - 1.40) |
| 0.88 (0.76 - 1.02) | 1.07 (0.74 - 1.54) | 0.84 (0.68 - 1.03) | CKSJ+OX | . | . | . | . | . | . | . | . | . | . | . | . | . | . | . | . | . | . | . | . | 1.40 (1.26 - 1.56) |
| 0.79 (0.60 - 1.05) | 0.96 (0.62 - 1.49) | 0.75 (0.55 - 1.03) | 0.90 (0.68 - 1.20) | CMP+OX | . | . | . | . | . | . | . | . | . | . | . | . | . | . | . | . | . | . | . | 1.56 (1.20 - 2.03) |
| 1.10 (0.73 - 1.65) | 1.33 (0.78 - 2.26) | 1.05 (0.68 - 1.61) | 1.25 (0.83 - 1.88) | 1.39 (0.86 - 2.23) | GPI+OX | . | . | . | . | . | . | . | . | . | . | . | . | . | . | . | . | . | . | 1.13 (0.76 - 1.67) |
| 1.00 (0.87 - 1.16) | 1.22 (0.84 - 1.76) | 0.96 (0.78 - 1.17) | 1.14 (0.98 - 1.33) | 1.27 (0.96 - 1.68) | 0.91 (0.61 - 1.38) | HCSP+OX | . | . | . | . | . | . | . | . | . | . | . | . | . | . | . | . | . | 1.23 (1.11 - 1.37) |
| 1.12 (0.56 - 2.26) | 1.36 (0.63 - 2.96) | 1.07 (0.52 - 2.18) | 1.28 (0.63 - 2.57) | 1.42 (0.68 - 2.97) | 1.02 (0.46 - 2.27) | 1.12 (0.56 - 2.25) | HEG+OX | . | . | . | . | . | . | . | . | . | . | . | . | . | . | . | . | 1.10 (0.55 - 2.19) |
| 1.01 (0.71 - 1.44) | 1.22 (0.75 - 2.00) | 0.96 (0.66 - 1.41) | 1.15 (0.80 - 1.64) | 1.27 (0.83 - 1.96) | 0.92 (0.55 - 1.55) | 1.01 (0.70 - 1.44) | 0.90 (0.42 - 1.94) | JLC+OX | . | . | . | . | . | . | . | . | . | . | . | . | . | . | . | 1.22 (0.87 - 1.72) |
| 0.82 (0.68 - 0.99) | 0.99 (0.67 - 1.46) | 0.78 (0.61 - 0.99) | 0.93 (0.77 - 1.13) | 1.03 (0.76 - 1.41) | 0.75 (0.49 - 1.14) | 0.82 (0.67 - 0.99) | 0.73 (0.36 - 1.48) | 0.81 (0.56 - 1.18) | KAI+OX | . | . | . | . | . | . | . | . | . | . | . | . | . | . | 1.51 (1.28 - 1.77) |
| 0.98 (0.73 - 1.32) | 1.19 (0.76 - 1.87) | 0.94 (0.67 - 1.30) | 1.12 (0.83 - 1.51) | 1.24 (0.85 - 1.82) | 0.90 (0.55 - 1.45) | 0.98 (0.73 - 1.32) | 0.88 (0.42 - 1.85) | 0.97 (0.63 - 1.51) | 1.20 (0.87 - 1.66) | KLTI+OX | . | . | . | . | . | . | . | . | . | . | . | . | . | 1.26 (0.95 - 1.66) |
| 0.97 (0.75 - 1.25) | 1.18 (0.77 - 1.80) | 0.92 (0.69 - 1.24) | 1.10 (0.85 - 1.43) | 1.22 (0.86 - 1.74) | 0.88 (0.56 - 1.40) | 0.97 (0.75 - 1.25) | 0.86 (0.42 - 1.79) | 0.96 (0.64 - 1.45) | 1.19 (0.89 - 1.58) | 0.99 (0.68 - 1.42) | KLXI+OX | . | . | . | . | . | . | . | . | . | . | . | . | 1.27 (1.01 - 1.61) |
| 1.29 (1.06 - 1.55) | 1.56 (1.06 - 2.30) | 1.23 (0.97 - 1.56) | 1.46 (1.21 - 1.78) | 1.62 (1.19 - 2.21) | 1.17 (0.76 - 1.80) | 1.28 (1.06 - 1.55) | 1.15 (0.56 - 2.33) | 1.27 (0.87 - 1.86) | 1.57 (1.25 - 1.97) | 1.31 (0.95 - 1.81) | 1.33 (1.00 - 1.76) | Lentinan+OX | . | . | . | . | . | . | . | . | . | . | . | 0.96 (0.82 - 1.13) |
| 0.80 (0.59 - 1.09) | 0.97 (0.61 - 1.54) | 0.76 (0.54 - 1.08) | 0.91 (0.67 - 1.25) | 1.01 (0.68 - 1.50) | 0.73 (0.45 - 1.19) | 0.80 (0.58 - 1.09) | 0.71 (0.34 - 1.51) | 0.79 (0.51 - 1.24) | 0.98 (0.70 - 1.37) | 0.81 (0.54 - 1.22) | 0.83 (0.57 - 1.20) | 0.62 (0.44 - 0.87) | PXC+OX | . | . | . | . | . | . | . | . | . | . | 1.54 (1.15 - 2.07) |
| 0.87 (0.65 - 1.17) | 1.05 (0.67 - 1.65) | 0.83 (0.60 - 1.15) | 0.99 (0.74 - 1.33) | 1.10 (0.75 - 1.60) | 0.79 (0.49 - 1.28) | 0.87 (0.64 - 1.16) | 0.77 (0.37 - 1.63) | 0.86 (0.56 - 1.33) | 1.06 (0.77 - 1.46) | 0.88 (0.60 - 1.31) | 0.90 (0.62 - 1.29) | 0.68 (0.49 - 0.93) | 1.09 (0.72 - 1.63) | QZC+OX | . | . | . | . | . | . | . | . | . | 1.42 (1.08 - 1.87) |
| 1.30 (0.89 - 1.90) | 1.57 (0.95 - 2.62) | 1.24 (0.82 - 1.86) | 1.48 (1.01 - 2.16) | 1.64 (1.04 - 2.57) | 1.18 (0.69 - 2.03) | 1.29 (0.88 - 1.89) | 1.16 (0.53 - 2.53) | 1.29 (0.78 - 2.12) | 1.59 (1.06 - 2.37) | 1.32 (0.83 - 2.09) | 1.34 (0.87 - 2.07) | 1.01 (0.68 - 1.50) | 1.62 (1.01 - 2.60) | 1.49 (0.94 - 2.36) | SFI+OX | . | . | . | . | . | . | . | . | 0.95 (0.66 - 1.37) |
| 1.05 (0.61 - 1.80) | 1.27 (0.67 - 2.40) | 1.00 (0.57 - 1.75) | 1.19 (0.69 - 2.05) | 1.32 (0.73 - 2.39) | 0.95 (0.49 - 1.85) | 1.04 (0.61 - 1.79) | 0.93 (0.39 - 2.23) | 1.04 (0.55 - 1.95) | 1.28 (0.73 - 2.23) | 1.06 (0.58 - 1.94) | 1.08 (0.60 - 1.93) | 0.81 (0.47 - 1.42) | 1.31 (0.71 - 2.40) | 1.20 (0.66 - 2.19) | 0.81 (0.42 - 1.54) | SXG+OX | . | . | . | . | . | . | . | 1.18 (0.69 - 2.01) |
| 0.95 (0.70 - 1.28) | 1.15 (0.73 - 1.81) | 0.90 (0.65 - 1.26) | 1.08 (0.80 - 1.46) | 1.20 (0.81 - 1.76) | 0.86 (0.53 - 1.41) | 0.94 (0.70 - 1.28) | 0.84 (0.40 - 1.78) | 0.94 (0.60 - 1.46) | 1.16 (0.83 - 1.61) | 0.96 (0.65 - 1.44) | 0.98 (0.68 - 1.41) | 0.74 (0.53 - 1.02) | 1.18 (0.79 - 1.79) | 1.09 (0.73 - 1.62) | 0.73 (0.46 - 1.16) | 0.91 (0.50 - 1.66) | SMI+OX | . | . | . | . | . | . | 1.30 (0.98 - 1.73) |
| 0.97 (0.84 - 1.12) | 1.17 (0.81 - 1.70) | 0.92 (0.75 - 1.13) | 1.10 (0.95 - 1.28) | 1.22 (0.92 - 1.62) | 0.88 (0.59 - 1.33) | 0.96 (0.83 - 1.12) | 0.86 (0.43 - 1.73) | 0.96 (0.67 - 1.37) | 1.18 (0.98 - 1.43) | 0.98 (0.73 - 1.33) | 1.00 (0.77 - 1.29) | 0.75 (0.62 - 0.91) | 1.21 (0.88 - 1.65) | 1.11 (0.83 - 1.50) | 0.75 (0.51 - 1.09) | 0.93 (0.54 - 1.59) | 1.02 (0.75 - 1.38) | SQFZI+OX | . | . | . | . | . | 1.28 (1.15 - 1.41) |
| 0.99 (0.45 - 2.18) | 1.20 (0.51 - 2.83) | 0.94 (0.42 - 2.10) | 1.12 (0.51 - 2.48) | 1.25 (0.55 - 2.85) | 0.90 (0.37 - 2.17) | 0.98 (0.45 - 2.17) | 0.88 (0.31 - 2.50) | 0.98 (0.42 - 2.30) | 1.21 (0.54 - 2.69) | 1.00 (0.44 - 2.31) | 1.02 (0.45 - 2.31) | 0.77 (0.34 - 1.71) | 1.23 (0.53 - 2.86) | 1.14 (0.49 - 2.61) | 0.76 (0.32 - 1.81) | 0.94 (0.37 - 2.44) | 1.04 (0.45 - 2.40) | 1.02 (0.46 - 2.25) | WMNC+OX | . | . | . | . | 1.25 (0.57 - 2.74) |
| 0.93 (0.77 - 1.11) | 1.12 (0.77 - 1.65) | 0.88 (0.70 - 1.11) | 1.06 (0.88 - 1.27) | 1.17 (0.87 - 1.58) | 0.84 (0.55 - 1.29) | 0.92 (0.77 - 1.11) | 0.83 (0.41 - 1.68) | 0.92 (0.63 - 1.33) | 1.13 (0.91 - 1.41) | 0.94 (0.69 - 1.30) | 0.96 (0.72 - 1.26) | 0.72 (0.58 - 0.90) | 1.16 (0.83 - 1.61) | 1.07 (0.78 - 1.46) | 0.71 (0.48 - 1.06) | 0.89 (0.51 - 1.54) | 0.98 (0.71 - 1.35) | 0.96 (0.80 - 1.15) | 0.94 (0.42 - 2.09) | XAPI+OX | . | . | . | 1.33 (1.15 - 1.55) |
| 0.78 (0.55 - 1.10) | 0.95 (0.58 - 1.54) | 0.74 (0.51 - 1.08) | 0.89 (0.63 - 1.26) | 0.99 (0.65 - 1.50) | 0.71 (0.43 - 1.19) | 0.78 (0.55 - 1.10) | 0.70 (0.32 - 1.50) | 0.77 (0.48 - 1.24) | 0.95 (0.66 - 1.38) | 0.79 (0.52 - 1.22) | 0.81 (0.54 - 1.21) | 0.61 (0.42 - 0.88) | 0.98 (0.63 - 1.52) | 0.90 (0.58 - 1.38) | 0.60 (0.37 - 0.99) | 0.75 (0.40 - 1.40) | 0.82 (0.53 - 1.27) | 0.81 (0.57 - 1.14) | 0.79 (0.34 - 1.85) | 0.84 (0.59 - 1.21) | XHC+OX | . | . | 1.58 (1.14 - 2.20) |
| 0.93 (0.80 - 1.09) | 1.13 (0.78 - 1.64) | 0.89 (0.72 - 1.10) | 1.06 (0.90 - 1.25) | 1.18 (0.88 - 1.57) | 0.85 (0.56 - 1.29) | 0.93 (0.79 - 1.09) | 0.83 (0.41 - 1.68) | 0.92 (0.64 - 1.33) | 1.14 (0.93 - 1.39) | 0.95 (0.70 - 1.29) | 0.96 (0.74 - 1.25) | 0.72 (0.59 - 0.89) | 1.16 (0.85 - 1.60) | 1.07 (0.79 - 1.45) | 0.72 (0.49 - 1.06) | 0.89 (0.52 - 1.54) | 0.98 (0.72 - 1.34) | 0.96 (0.82 - 1.13) | 0.94 (0.43 - 2.09) | 1.01 (0.83 - 1.22) | 1.19 (0.84 - 1.70) | YDZOI+OX | . | 1.32 (1.17 - 1.50) |
| 0.91 (0.69 - 1.19) | 1.10 (0.71 - 1.70) | 0.86 (0.64 - 1.17) | 1.03 (0.79 - 1.36) | 1.14 (0.80 - 1.64) | 0.83 (0.52 - 1.32) | 0.90 (0.69 - 1.19) | 0.81 (0.39 - 1.68) | 0.90 (0.59 - 1.37) | 1.11 (0.82 - 1.49) | 0.92 (0.63 - 1.34) | 0.93 (0.66 - 1.32) | 0.70 (0.52 - 0.95) | 1.13 (0.77 - 1.67) | 1.04 (0.72 - 1.52) | 0.70 (0.45 - 1.09) | 0.87 (0.48 - 1.56) | 0.96 (0.65 - 1.40) | 0.94 (0.71 - 1.23) | 0.92 (0.40 - 2.09) | 0.98 (0.73 - 1.31) | 1.16 (0.77 - 1.76) | 0.97 (0.74 - 1.29) | YZXJC+OX | 1.36 (1.06 - 1.75) |
| 1.23 (1.12 - 1.36) | 1.50 (1.05 - 2.13) | 1.18 (0.99 - 1.40) | 1.40 (1.26 - 1.56) | 1.56 (1.20 - 2.03) | 1.13 (0.76 - 1.67) | 1.23 (1.11 - 1.37) | 1.10 (0.55 - 2.19) | 1.22 (0.87 - 1.72) | 1.51 (1.28 - 1.77) | 1.26 (0.95 - 1.66) | 1.27 (1.01 - 1.61) | 0.96 (0.82 - 1.13) | 1.54 (1.15 - 2.07) | 1.42 (1.08 - 1.87) | 0.95 (0.66 - 1.37) | 1.18 (0.69 - 2.01) | 1.30 (0.98 - 1.73) | 1.28 (1.15 - 1.41) | 1.25 (0.57 - 2.74) | 1.33 (1.15 - 1.55) | 1.58 (1.14 - 2.20) | 1.32 (1.17 - 1.50) | 1.36 (1.06 - 1.75) | OX |

1. **1-year OS**

| Aidi Injection+OX | . | . | . | . | 1.27 (0.98 - 1.65) |
| --- | --- | --- | --- | --- | --- |
| 0.93 (0.45 - 1.91) | Shenqi Fuzheng Injection+OX | . | . | . | 1.37 (0.70 - 2.68) |
| 1.09 (0.40 - 2.94) | 1.17 (0.36 - 3.78) | Xiaoaiping Injection+OX | . | . | 1.17 (0.45 - 3.04) |
| 1.18 (0.84 - 1.66) | 1.27 (0.62 - 2.58) | 1.08 (0.40 - 2.89) | Xihuang Capsules+OX | . | 1.08 (0.86 - 1.35) |
| 1.01 (0.65 - 1.56) | 1.08 (0.51 - 2.31) | 0.92 (0.33 - 2.56) | 0.85 (0.56 - 1.30) | Ya Dan Zi Oil Emulsion Injection+OX | 1.26 (0.89 - 1.79) |
| 1.27 (0.98 - 1.65) | 1.37 (0.70 - 2.68) | 1.17 (0.45 - 3.04) | 1.08 (0.86 - 1.35) | 1.26 (0.89 - 1.79) | OX |

1. **2-year OS**

| Shenqi Fuzheng Injection+OX | . | . | 1.49 (0.99 - 2.24) |
| --- | --- | --- | --- |
| 0.68 (0.35 - 1.32) | Xihuang Capsules+OX | . | 2.20 (1.30 - 3.73) |
| 1.06 (0.35 - 3.24) | 1.57 (0.49 - 5.03) | Ya Dan Zi Oil Emulsion Injection+OX | 1.40 (0.50 - 3.95) |
| 1.49 (0.99 - 2.24) | 2.20 (1.30 - 3.73) | 1.40 (0.50 - 3.95) | OX |

1. **CA125**

| Aidi Injection+OX | . | . | . | -2.69 (-6.05 - 0.67) |
| --- | --- | --- | --- | --- |
| 1.07 (-3.03 - 5.18) | Compound Kushen Injection+OX | . | . | -3.76 (-6.12 - -1.41) |
| -4.34 (-8.22 - -0.46) | -5.41 (-8.47 - -2.36) | Shenqi Fuzheng Injection+OX | . | 1.65 (-0.29 - 3.60) |
| -1.48 (-6.09 - 3.13) | -2.55 (-6.49 - 1.38) | 2.86 (-0.85 - 6.57) | Yangzheng Xiaoji Capsules+OX | -1.21 (-4.36 - 1.94) |
| -2.69 (-6.05 - 0.67) | -3.76 (-6.12 - -1.41) | 1.65 (-0.29 - 3.60) | -1.21 (-4.36 - 1.94) | OX |

1. **CA199**

| Aidi Injection+OX | . | . | . | . | . | . | . | . | . | . | -12.40 (-21.52 - -3.27) |
| --- | --- | --- | --- | --- | --- | --- | --- | --- | --- | --- | --- |
| 3.69 ( -8.27 - 15.65) | Compound Kushen Injection+OX | . | . | . | . | . | . | . | . | . | -16.09 (-23.81 - -8.36) |
| 12.51 ( -2.48 - 27.49) | 8.82 ( -5.36 - 22.99) | Compound Mylabris preparations+OX | . | . | . | . | . | . | . | . | -24.90 (-36.79 - -13.02) |
| -4.47 (-21.56 - 12.62) | -8.16 (-24.54 - 8.22) | -16.98 (-35.68 - 1.73) | Huachansu preparations+OX | . | . | . | . | . | . | . | -7.92 (-22.37 - 6.52) |
| -6.32 (-29.11 - 16.48) | -10.01 (-32.28 - 12.26) | -18.82 (-42.85 - 5.21) | -1.84 (-27.24 - 23.55) | Huai'er Granules+OX | . | . | . | . | . | . | -6.08 (-26.96 - 14.80) |
| 2.96 (-15.53 - 21.45) | -0.74 (-18.58 - 17.11) | -9.55 (-29.55 - 10.45) | 7.43 (-14.19 - 29.04) | 9.27 (-17.09 - 35.63) | Kangai Injection+OX | . | . | . | . | . | -15.35 (-31.43 - 0.73) |
| -9.19 (-34.69 - 16.31) | -12.88 (-37.91 - 12.16) | -21.69 (-48.31 - 4.92) | -4.71 (-32.56 - 23.13) | -2.87 (-34.54 - 28.80) | -12.14 (-40.87 - 16.59) | Kanglaite Injection+OX | . | . | . | . | -3.21 (-27.02 - 20.60) |
| 20.85 ( 5.51 - 36.20) | 17.16 ( 2.60 - 31.71) | 8.34 ( -8.79 - 25.47) | 25.32 ( 6.33 - 44.32) | 27.17 ( 2.91 - 51.42) | 17.89 ( -2.37 - 38.16) | 30.04 ( 3.22 - 56.85) | Kanglixin Capsules+OX | . | . | . | -33.25 (-45.58 - -20.91) |
| 3.35 (-22.62 - 29.33) | -0.34 (-25.85 - 25.18) | -9.15 (-36.22 - 17.91) | 7.83 (-20.46 - 36.11) | 9.67 (-22.39 - 41.73) | 0.40 (-28.76 - 29.55) | 12.54 (-21.49 - 46.57) | -17.50 (-44.76 - 9.77) | Shengxue Granules+OX | . | . | -15.75 (-40.07 - 8.57) |
| 9.16 (-13.31 - 31.64) | 5.47 (-16.47 - 27.42) | -3.34 (-27.07 - 20.39) | 13.64 (-11.48 - 38.75) | 15.48 (-13.81 - 44.77) | 6.21 (-19.88 - 32.29) | 18.35 (-13.10 - 49.80) | -11.69 (-35.65 - 12.27) | 5.81 (-26.02 - 37.64) | Shenlian Capsule+OX | . | -21.56 (-42.10 - -1.02) |
| 2.67 (-12.51 - 17.85) | -1.02 (-15.40 - 13.36) | -9.84 (-26.82 - 7.15) | 7.14 (-11.72 - 26.00) | 8.99 (-15.17 - 33.14) | -0.29 (-20.43 - 19.86) | 11.86 (-14.87 - 38.58) | -18.18 (-35.48 - -0.88) | -0.68 (-27.86 - 26.49) | -6.49 (-30.35 - 17.36) | Shenqi Fuzheng Injection+OX | -15.07 (-27.20 - -2.94) |
| -12.40 (-21.52 - -3.27) | -16.09 (-23.81 - -8.36) | -24.90 (-36.79 - -13.02) | -7.92 (-22.37 - 6.52) | -6.08 (-26.96 - 14.80) | -15.35 (-31.43 - 0.73) | -3.21 (-27.02 - 20.60) | -33.25 (-45.58 - -20.91) | -15.75 (-40.07 - 8.57) | -21.56 (-42.10 - -1.02) | -15.07 (-27.20 - -2.94) | OX |

1. **CA724**

| Aidi Injection+OX | . | . | . | . | . | . | . | -14.19 (-21.54 - -6.84) |
| --- | --- | --- | --- | --- | --- | --- | --- | --- |
| -1.68 (-10.80 - 7.45) | Compound Kushen Injection+OX | . | . | . | . | . | . | -12.52 (-17.92 - -7.11) |
| -5.27 (-18.52 - 7.98) | -3.60 (-15.87 - 8.68) | Compound Mylabris preparations+OX | . | . | . | . | . | -8.92 (-19.94 - 2.10) |
| -8.33 (-19.06 - 2.39) | -6.66 (-16.15 - 2.84) | -3.06 (-16.57 - 10.45) | Kangai Injection+OX | . | . | . | . | -5.86 (-13.67 - 1.95) |
| 17.90 ( 5.39 - 30.40) | 19.57 ( 8.10 - 31.04) | 23.17 ( 8.21 - 38.13) | 26.23 ( 13.45 - 39.01) | Kanglixin Capsules+OX | . | . | . | -32.09 (-42.20 - -21.97) |
| -9.63 (-22.70 - 3.44) | -7.96 (-20.04 - 4.13) | -4.36 (-19.80 - 11.08) | -1.30 (-14.63 - 12.03) | -27.53 (-42.33 - -12.73) | Shengxue Granules+OX | . | . | -4.56 (-15.37 - 6.25) |
| -6.45 (-19.59 - 6.69) | -4.78 (-16.93 - 7.38) | -1.18 (-16.68 - 14.32) | 1.88 (-11.52 - 15.28) | -24.35 (-39.21 - -9.49) | 3.18 (-12.16 - 18.52) | Shenlian Capsule+OX | . | -7.74 (-18.63 - 3.15) |
| -8.21 (-21.86 - 5.43) | -6.54 (-19.24 - 6.17) | -2.94 (-18.87 - 12.99) | 0.12 (-13.78 - 14.02) | -26.11 (-41.42 - -10.80) | 1.42 (-14.36 - 17.20) | -1.76 (-17.59 - 14.07) | Shenqi Fuzheng Injection+OX | -5.98 (-17.48 - 5.52) |
| -14.19 (-21.54 - -6.84) | -12.52 (-17.92 - -7.11) | -8.92 (-19.94 - 2.10) | -5.86 (-13.67 - 1.95) | -32.09 (-42.20 - -21.97) | -4.56 (-15.37 - 6.25) | -7.74 (-18.63 - 3.15) | -5.98 (-17.48 - 5.52) | OX |

1. **CEA**

| Aidi Injection+OX | . | . | . | . | . | . | . | . | . | . | . | . | . | -6.15 ( -9.34 - -2.96) |
| --- | --- | --- | --- | --- | --- | --- | --- | --- | --- | --- | --- | --- | --- | --- |
| -2.53 (-10.80 - 5.74) | Astragalus preparations+OX | . | . | . | . | . | . | . | . | . | . | . | . | -3.62 (-11.25 - 4.01) |
| 1.39 ( -2.67 - 5.45) | 3.92 ( -4.11 - 11.95) | Compound Kushen Injection+OX | . | . | . | . | . | . | . | . | . | . | . | -7.54 (-10.05 - -5.03) |
| -0.47 ( -5.47 - 4.53) | 2.06 ( -6.49 - 10.60) | -1.86 ( -6.46 - 2.74) | Compound Mylabris preparations+OX | . | . | . | . | . | . | . | . | . | . | -5.68 ( -9.53 - -1.83) |
| -3.32 ( -9.41 - 2.77) | -0.79 (-10.01 - 8.43) | -4.71 (-10.47 - 1.05) | -2.85 ( -9.31 - 3.61) | Huachansu preparations+OX | . | . | . | . | . | . | . | . | . | -2.83 ( -8.01 - 2.35) |
| -2.91 (-11.50 - 5.68) | -0.38 (-11.41 - 10.65) | -4.30 (-12.66 - 4.06) | -2.44 (-11.29 - 6.41) | 0.41 ( -9.10 - 9.92) | Huai'er Granules+OX | . | . | . | . | . | . | . | . | -3.24 (-11.21 - 4.73) |
| 1.93 ( -3.87 - 7.72) | 4.46 ( -4.57 - 13.49) | 0.54 ( -4.91 - 5.99) | 2.40 ( -3.79 - 8.58) | 5.25 ( -1.84 - 12.34) | 4.84 ( -4.49 - 14.16) | Kangai Injection+OX | . | . | . | . | . | . | . | -8.08 (-12.91 - -3.24) |
| 12.05 ( 3.71 - 20.39) | 14.58 ( 3.74 - 25.42) | 10.66 ( 2.55 - 18.76) | 12.52 ( 3.90 - 21.14) | 15.37 ( 6.08 - 24.66) | 14.96 ( 3.87 - 26.05) | 10.12 ( 1.02 - 19.22) | Kanglaite Injection+OX | . | . | . | . | . | . | -18.20 (-25.91 - -10.49) |
| 9.07 ( 2.67 - 15.46) | 11.60 ( 2.17 - 21.03) | 7.68 ( 1.59 - 13.76) | 9.54 ( 2.79 - 16.29) | 12.39 ( 4.80 - 19.98) | 11.98 ( 2.27 - 21.69) | 7.14 ( -0.22 - 14.50) | -2.98 (-12.48 - 6.51) | Kanglixin Capsules+OX | . | . | . | . | . | -15.22 (-20.76 - -9.67) |
| 10.28 ( 1.99 - 18.57) | 12.81 ( 2.00 - 23.62) | 8.89 ( 0.83 - 16.95) | 10.75 ( 2.18 - 19.32) | 13.60 ( 4.35 - 22.85) | 13.19 ( 2.14 - 24.24) | 8.35 ( -0.70 - 17.41) | -1.77 (-12.63 - 9.09) | 1.21 ( -8.24 - 10.67) | Shengxue Granules+OX | . | . | . | . | -16.43 (-24.09 - -8.77) |
| 9.24 ( -8.98 - 27.46) | 11.77 ( -7.72 - 31.26) | 7.85 (-10.26 - 25.96) | 9.71 ( -8.64 - 28.06) | 12.56 ( -6.11 - 31.23) | 12.15 ( -7.48 - 31.78) | 7.31 (-11.27 - 25.89) | -2.81 (-22.33 - 16.71) | 0.17 (-18.60 - 18.95) | -1.04 (-20.54 - 18.46) | Shenlian Capsule+OX | . | . | . | -15.39 (-33.33 - 2.55) |
| 1.68 ( -6.74 - 10.10) | 4.21 ( -6.69 - 15.11) | 0.29 ( -7.89 - 8.47) | 2.15 ( -6.54 - 10.84) | 5.00 ( -4.35 - 14.35) | 4.59 ( -6.55 - 15.73) | -0.25 ( -9.41 - 8.92) | -10.37 (-21.33 - 0.59) | -7.39 (-16.95 - 2.17) | -8.60 (-19.52 - 2.32) | -7.56 (-27.12 - 12.00) | Shenmai Injection+OX | . | . | -7.83 (-15.62 - -0.04) |
| 3.72 ( -1.17 - 8.62) | 6.25 ( -2.23 - 14.73) | 2.33 ( -2.15 - 6.81) | 4.19 ( -1.16 - 9.54) | 7.04 ( 0.67 - 13.42) | 6.63 ( -2.16 - 15.42) | 1.79 ( -4.30 - 7.89) | -8.33 (-16.88 - 0.23) | -5.35 (-12.02 - 1.33) | -6.56 (-15.07 - 1.95) | -5.52 (-23.84 - 12.80) | 2.04 ( -6.59 - 10.67) | Shenqi Fuzheng Injection+OX | . | -9.87 (-13.58 - -6.16) |
| -2.87 (-10.44 - 4.70) | -0.34 (-10.60 - 9.93) | -4.26 (-11.57 - 3.05) | -2.40 (-10.27 - 5.48) | 0.45 ( -8.15 - 9.06) | 0.04 (-10.48 - 10.56) | -4.80 (-13.20 - 3.61) | -14.92 (-25.24 - -4.59) | -11.94 (-20.76 - -3.11) | -13.15 (-23.43 - -2.86) | -12.11 (-31.32 - 7.10) | -4.55 (-14.93 - 5.84) | -6.59 (-14.40 - 1.22) | Ya Dan Zi Oil Emulsion Injection+OX | -3.28 (-10.15 - 3.59) |
| -6.15 ( -9.34 - -2.96) | -3.62 (-11.25 - 4.01) | -7.54 (-10.05 - -5.03) | -5.68 ( -9.53 - -1.83) | -2.83 ( -8.01 - 2.35) | -3.24 (-11.21 - 4.73) | -8.08 (-12.91 - -3.24) | -18.20 (-25.91 - -10.49) | -15.22 (-20.76 - -9.67) | -16.43 (-24.09 - -8.77) | -15.39 (-33.33 - 2.55) | -7.83 (-15.62 - -0.04) | -9.87 (-13.58 - -6.16) | -3.28 (-10.15 - 3.59) | OX |

1. **CD3+**

| Aidi Injection+OX | . | . | . | . | . | . | . | . | . | . | . | . | . | . | . | . | . | . | 11.09 ( 6.67 - 15.51) |
| --- | --- | --- | --- | --- | --- | --- | --- | --- | --- | --- | --- | --- | --- | --- | --- | --- | --- | --- | --- |
| 2.08 ( -5.39 - 9.54) | Astragalus Polysaccharides+OX | . | . | . | . | . | . | . | . | . | . | . | . | . | . | . | . | . | 9.01 ( 2.99 - 15.03) |
| 3.59 ( -5.88 - 13.05) | 1.51 ( -8.80 - 11.82) | Astragalus preparations+OX | . | . | . | . | . | . | . | . | . | . | . | . | . | . | . | . | 7.50 ( -0.87 - 15.87) |
| 1.74 ( -3.27 - 6.76) | -0.34 ( -6.80 - 6.13) | -1.84 (-10.54 - 6.85) | Compound Kushen Injection+OX | . | . | . | . | . | . | . | . | . | . | . | . | . | . | . | 9.34 ( 6.97 - 11.72) |
| 5.79 ( -0.33 - 11.91) | 3.71 ( -3.64 - 11.07) | 2.21 ( -7.17 - 11.59) | 4.05 ( -0.80 - 8.90) | Compound Mylabris preparations+OX | . | . | . | . | . | . | . | . | . | . | . | . | . | . | 5.29 ( 1.06 - 9.53) |
| 0.54 ( -9.19 - 10.26) | -1.54 (-12.09 - 9.01) | -3.05 (-15.10 - 9.00) | -1.21 (-10.19 - 7.78) | -5.26 (-14.90 - 4.39) | Ginseng Polysaccharide Injection+OX | . | . | . | . | . | . | . | . | . | . | . | . | . | 10.55 ( 1.89 - 19.21) |
| 3.66 ( -2.95 - 10.27) | 1.58 ( -6.19 - 9.35) | 0.08 ( -9.63 - 9.78) | 1.92 ( -3.54 - 7.38) | -2.13 ( -8.62 - 4.36) | 3.13 ( -6.84 - 13.09) | Huachansu preparations+OX | . | . | . | . | . | . | . | . | . | . | . | . | 7.42 ( 2.51 - 12.34) |
| 2.80 ( -3.94 - 9.55) | 0.73 ( -7.16 - 8.61) | -0.78 (-10.58 - 9.02) | 1.06 ( -4.56 - 6.68) | -2.99 ( -9.61 - 3.64) | 2.27 ( -7.78 - 12.32) | -0.86 ( -7.94 - 6.22) | Jinlong Capsules+OX | . | . | . | . | . | . | . | . | . | . | . | 8.28 ( 3.19 - 13.38) |
| -0.57 ( -7.12 - 5.99) | -2.64 (-10.37 - 5.08) | -4.15 (-13.82 - 5.52) | -2.31 ( -7.70 - 3.08) | -6.36 (-12.79 - 0.07) | -1.10 (-11.03 - 8.82) | -4.23 (-11.13 - 2.67) | -3.37 (-10.40 - 3.66) | Kangai Injection+OX | . | . | . | . | . | . | . | . | . | . | 11.65 ( 6.81 - 16.50) |
| 1.78 ( -5.65 - 9.21) | -0.30 ( -8.78 - 8.18) | -1.80 (-12.09 - 8.48) | 0.04 ( -6.39 - 6.47) | -4.01 (-11.34 - 3.31) | 1.25 ( -9.28 - 11.77) | -1.88 ( -9.62 - 5.86) | -1.02 ( -8.88 - 6.83) | 2.35 ( -5.34 - 10.04) | Kanglixin Capsules+OX | . | . | . | . | . | . | . | . | . | 9.30 ( 3.33 - 15.28) |
| 2.91 ( -4.55 - 10.36) | 0.83 ( -7.67 - 9.33) | -0.68 (-10.98 - 9.62) | 1.17 ( -5.29 - 7.62) | -2.89 (-10.23 - 4.46) | 2.37 ( -8.17 - 12.91) | -0.75 ( -8.52 - 7.01) | 0.10 ( -7.77 - 7.98) | 3.47 ( -4.24 - 11.19) | 1.13 ( -7.35 - 9.60) | Lentinan+OX | . | . | . | . | . | . | . | . | 8.18 ( 2.17 - 14.18) |
| -1.38 (-11.08 - 8.31) | -3.46 (-13.98 - 7.06) | -4.97 (-16.99 - 7.05) | -3.13 (-12.08 - 5.83) | -7.18 (-16.79 - 2.44) | -1.92 (-14.15 - 10.31) | -5.05 (-14.98 - 4.89) | -4.19 (-14.21 - 5.83) | -0.82 (-10.71 - 9.08) | -3.17 (-13.66 - 7.33) | -4.29 (-14.81 - 6.22) | Pingxiao Capsules+OX | . | . | . | . | . | . | . | 12.47 ( 3.84 - 21.10) |
| 1.06 ( -8.38 - 10.49) | -1.02 (-11.31 - 9.26) | -2.53 (-14.34 - 9.28) | -0.69 ( -9.36 - 7.98) | -4.74 (-14.09 - 4.62) | 0.52 (-11.51 - 12.55) | -2.61 (-12.29 - 7.07) | -1.75 (-11.52 - 8.02) | 1.62 ( -8.02 - 11.27) | -0.73 (-10.98 - 9.53) | -1.85 (-12.13 - 8.43) | 2.44 ( -9.56 - 14.44) | Shenmai Injection+OX | . | . | . | . | . | . | 10.03 ( 1.69 - 18.37) |
| 3.26 ( -1.93 - 8.46) | 1.18 ( -5.43 - 7.79) | -0.33 ( -9.13 - 8.48) | 1.52 ( -2.10 - 5.14) | -2.53 ( -7.57 - 2.51) | 2.72 ( -6.36 - 11.81) | -0.40 ( -6.02 - 5.22) | 0.46 ( -5.32 - 6.24) | 3.83 ( -1.73 - 9.39) | 1.48 ( -5.09 - 8.05) | 0.35 ( -6.24 - 6.95) | 4.64 ( -4.41 - 13.70) | 2.20 ( -6.57 - 10.98) | Shenqi Fuzheng Injection+OX | . | . | . | . | . | 7.83 ( 5.09 - 10.56) |
| -2.76 (-10.42 - 4.91) | -4.84 (-13.52 - 3.85) | -6.35 (-16.80 - 4.11) | -4.50 (-11.20 - 2.19) | -8.55 (-16.11 - -0.99) | -3.30 (-13.99 - 7.39) | -6.42 (-14.38 - 1.54) | -5.56 (-13.64 - 2.51) | -2.19 (-10.11 - 5.72) | -4.54 (-13.20 - 4.11) | -5.67 (-14.34 - 3.01) | -1.38 (-12.04 - 9.29) | -3.82 (-14.24 - 6.61) | -6.02 (-12.85 - 0.81) | Xiaoaiping Injection+OX | . | . | . | . | 13.85 ( 7.58 - 20.11) |
| 0.05 ( -7.46 - 7.55) | -2.03 (-10.57 - 6.51) | -3.54 (-13.87 - 6.79) | -1.70 ( -8.21 - 4.81) | -5.75 (-13.14 - 1.65) | -0.49 (-11.06 - 10.08) | -3.62 (-11.42 - 4.19) | -2.76 (-10.68 - 5.16) | 0.61 ( -7.15 - 8.37) | -1.74 (-10.25 - 6.78) | -2.86 (-11.39 - 5.67) | 1.43 ( -9.12 - 11.98) | -1.01 (-11.32 - 9.30) | -3.22 ( -9.86 - 3.43) | 2.81 ( -5.91 - 11.52) | Xihuang Capsules+OX | . | . | . | 11.04 ( 4.98 - 17.10) |
| 3.15 ( -4.51 - 10.81) | 1.07 ( -7.61 - 9.76) | -0.44 (-10.89 - 10.02) | 1.41 ( -5.29 - 8.10) | -2.64 (-10.20 - 4.92) | 2.61 ( -8.08 - 13.30) | -0.51 ( -8.47 - 7.45) | 0.35 ( -7.73 - 8.42) | 3.72 ( -4.20 - 11.63) | 1.37 ( -7.29 - 10.02) | 0.24 ( -8.43 - 8.92) | 4.53 ( -6.13 - 15.20) | 2.09 ( -8.33 - 12.52) | -0.11 ( -6.94 - 6.72) | 5.91 ( -2.95 - 14.77) | 3.10 ( -5.61 - 11.82) | Ya Dan Zi Oil Emulsion Injection+OX | . | . | 7.94 ( 1.67 - 14.20) |
| 1.77 ( -7.80 - 11.33) | -0.31 (-10.71 - 10.09) | -1.82 (-13.74 - 10.10) | 0.02 ( -8.79 - 8.83) | -4.03 (-13.51 - 5.46) | 1.23 (-10.90 - 13.36) | -1.90 (-11.70 - 7.91) | -1.04 (-10.94 - 8.86) | 2.33 ( -7.44 - 12.10) | -0.02 (-10.39 - 10.36) | -1.14 (-11.54 - 9.25) | 3.15 ( -8.95 - 15.25) | 0.71 (-11.19 - 12.61) | -1.49 (-10.41 - 7.42) | 4.53 ( -6.02 - 15.07) | 1.72 ( -8.71 - 12.15) | -1.38 (-11.93 - 9.16) | Yangzheng Xiaoji Capsules+OX | . | 9.32 ( 0.84 - 17.80) |
| -0.01 ( -9.58 - 9.55) | -2.09 (-12.49 - 8.31) | -3.60 (-15.51 - 8.31) | -1.76 (-10.56 - 7.05) | -5.81 (-15.29 - 3.67) | -0.55 (-12.67 - 11.57) | -3.68 (-13.48 - 6.13) | -2.82 (-12.71 - 7.08) | 0.55 ( -9.21 - 10.32) | -1.80 (-12.17 - 8.58) | -2.92 (-13.31 - 7.47) | 1.37 (-10.73 - 13.47) | -1.07 (-12.96 - 10.82) | -3.27 (-12.18 - 5.63) | 2.75 ( -7.80 - 13.29) | -0.06 (-10.48 - 10.37) | -3.16 (-13.71 - 7.38) | -1.78 (-13.78 - 10.22) | Zhenqi Fuzheng Granules+OX | 11.10 ( 2.62 - 19.58) |
| 11.09 ( 6.67 - 15.51) | 9.01 ( 2.99 - 15.03) | 7.50 ( -0.87 - 15.87) | 9.34 ( 6.97 - 11.72) | 5.29 ( 1.06 - 9.53) | 10.55 ( 1.89 - 19.21) | 7.42 ( 2.51 - 12.34) | 8.28 ( 3.19 - 13.38) | 11.65 ( 6.81 - 16.50) | 9.30 ( 3.33 - 15.28) | 8.18 ( 2.17 - 14.18) | 12.47 ( 3.84 - 21.10) | 10.03 ( 1.69 - 18.37) | 7.83 ( 5.09 - 10.56) | 13.85 ( 7.58 - 20.11) | 11.04 ( 4.98 - 17.10) | 7.94 ( 1.67 - 14.20) | 9.32 ( 0.84 - 17.80) | 11.10 ( 2.62 - 19.58) | OX |

1. **NK cell**

| Aidi Injection+OX | . | . | . | . | . | . | . | . | . | 2.66 ( -2.76 - 8.08) |
| --- | --- | --- | --- | --- | --- | --- | --- | --- | --- | --- |
| 3.95 ( -2.75 - 10.66) | Astragalus Polysaccharides+OX | . | . | . | . | . | . | . | . | -1.29 ( -5.24 - 2.66) |
| -3.34 (-11.14 - 4.46) | -7.29 (-14.15 - -0.44) | Astragalus preparations+OX | . | . | . | . | . | . | . | 6.00 ( 0.40 - 11.60) |
| -2.18 ( -8.51 - 4.15) | -6.13 (-11.26 - -1.01) | 1.16 ( -5.33 - 7.65) | Compound Kushen Injection+OX | . | . | . | . | . | . | 4.84 ( 1.57 - 8.11) |
| 1.39 ( -6.54 - 9.32) | -2.56 ( -9.57 - 4.44) | 4.73 ( -3.32 - 12.78) | 3.57 ( -3.07 - 10.21) | Ginseng Polysaccharide Injection+OX | . | . | . | . | . | 1.27 ( -4.52 - 7.06) |
| -1.89 ( -8.20 - 4.42) | -5.84 (-10.94 - -0.74) | 1.45 ( -5.02 - 7.92) | 0.29 ( -4.31 - 4.89) | -3.28 ( -9.91 - 3.35) | Huachansu preparations+OX | . | . | . | . | 4.55 ( 1.31 - 7.78) |
| -6.45 (-12.93 - 0.02) | -10.41 (-15.71 - -5.10) | -3.11 ( -9.74 - 3.51) | -4.27 ( -9.09 - 0.54) | -7.84 (-14.62 - -1.06) | -4.56 ( -9.36 - 0.23) | Jinlong Capsules+OX | . | . | . | 9.11 ( 5.57 - 12.65) |
| -18.10 (-25.92 - -10.28) | -22.05 (-28.93 - -15.17) | -14.76 (-22.70 - -6.82) | -15.92 (-22.43 - -9.41) | -19.49 (-27.56 - -11.42) | -16.21 (-22.71 - -9.72) | -11.65 (-18.30 - -5.00) | Kangai Injection+OX | . | . | 20.76 ( 15.13 - 26.39) |
| 0.78 ( -7.08 - 8.64) | -3.17 (-10.10 - 3.76) | 4.12 ( -3.87 - 12.11) | 2.96 ( -3.60 - 9.53) | -0.61 ( -8.73 - 7.51) | 2.67 ( -3.88 - 9.22) | 7.23 ( 0.53 - 13.94) | 18.88 ( 10.87 - 26.89) | Lentinan+OX | . | 1.88 ( -3.81 - 7.57) |
| -2.56 ( -8.68 - 3.57) | -6.51 (-11.38 - -1.64) | 0.78 ( -5.50 - 7.07) | -0.38 ( -4.71 - 3.96) | -3.95 (-10.40 - 2.50) | -0.67 ( -4.98 - 3.64) | 3.90 ( -0.65 - 8.44) | 15.54 ( 9.23 - 21.86) | -3.34 ( -9.70 - 3.03) | Shenqi Fuzheng Injection+OX | 5.22 ( 2.37 - 8.07) |
| 2.66 ( -2.76 - 8.08) | -1.29 ( -5.24 - 2.66) | 6.00 ( 0.40 - 11.60) | 4.84 ( 1.57 - 8.11) | 1.27 ( -4.52 - 7.06) | 4.55 ( 1.31 - 7.78) | 9.11 ( 5.57 - 12.65) | 20.76 ( 15.13 - 26.39) | 1.88 ( -3.81 - 7.57) | 5.22 ( 2.37 - 8.07) | OX |

1. **CD4+**

| Aidi Injection+OX | . | . | . | . | . | . | . | . | . | . | . | . | . | . | . | . | . | . | . | 7.89 ( 4.55 - 11.24) |
| --- | --- | --- | --- | --- | --- | --- | --- | --- | --- | --- | --- | --- | --- | --- | --- | --- | --- | --- | --- | --- |
| -3.93 (-10.45 - 2.59) | Astragalus Polysaccharides+OX | . | . | . | . | . | . | . | . | . | . | . | . | . | . | . | . | . | . | 11.82 ( 6.23 - 17.42) |
| 5.50 ( -0.12 - 11.12) | 9.43 ( 2.23 - 16.62) | Astragalus preparations+OX | . | . | . | . | . | . | . | . | . | . | . | . | . | . | . | . | . | 2.40 ( -2.12 - 6.92) |
| 0.66 ( -3.34 - 4.65) | 4.59 ( -1.42 - 10.60) | -4.84 ( -9.86 - 0.18) | Compound Kushen Injection+OX | . | . | . | . | . | . | . | . | . | . | . | . | . | . | . | . | 7.24 ( 5.05 - 9.42) |
| 2.73 ( -2.42 - 7.87) | 6.66 ( -0.17 - 13.48) | -2.77 ( -8.75 - 3.20) | 2.07 ( -2.41 - 6.54) | Compound Mylabris preparations+OX | . | . | . | . | . | . | . | . | . | . | . | . | . | . | . | 5.17 ( 1.26 - 9.08) |
| -1.61 (-10.01 - 6.80) | 2.32 ( -7.20 - 11.85) | -7.10 (-16.04 - 1.83) | -2.26 (-10.28 - 5.75) | -4.33 (-12.97 - 4.31) | Diyu Shengbai Tablet+OX | . | . | . | . | . | . | . | . | . | . | . | . | . | . | 9.50 ( 1.79 - 17.21) |
| 3.06 ( -5.95 - 12.08) | 6.99 ( -3.07 - 17.06) | -2.43 (-11.95 - 7.08) | 2.41 ( -6.24 - 11.06) | 0.34 ( -8.90 - 9.58) | 4.67 ( -6.71 - 16.05) | Ginseng Polysaccharide Injection+OX | . | . | . | . | . | . | . | . | . | . | . | . | . | 4.83 ( -3.54 - 13.20) |
| 1.42 ( -3.45 - 6.29) | 5.35 ( -1.27 - 11.97) | -4.08 ( -9.82 - 1.66) | 0.76 ( -3.40 - 4.92) | -1.31 ( -6.58 - 3.97) | 3.02 ( -5.46 - 11.51) | -1.65 (-10.73 - 7.44) | Huachansu preparations+OX | . | . | . | . | . | . | . | . | . | . | . | . | 6.48 ( 2.93 - 10.02) |
| 2.46 ( -2.89 - 7.81) | 6.39 ( -0.59 - 13.37) | -3.04 ( -9.19 - 3.12) | 1.80 ( -2.91 - 6.51) | -0.27 ( -5.99 - 5.45) | 4.07 ( -4.70 - 12.83) | -0.60 ( -9.96 - 8.75) | 1.04 ( -4.43 - 6.52) | Jinlong Capsules+OX | . | . | . | . | . | . | . | . | . | . | . | 5.43 ( 1.26 - 9.61) |
| -0.68 ( -5.83 - 4.47) | 3.25 ( -3.58 - 10.08) | -6.18 (-12.16 - -0.20) | -1.34 ( -5.82 - 3.14) | -3.41 ( -8.94 - 2.12) | 0.92 ( -7.72 - 9.57) | -3.75 (-12.99 - 5.49) | -2.10 ( -7.38 - 3.18) | -3.14 ( -8.86 - 2.58) | Kangai Injection+OX | . | . | . | . | . | . | . | . | . | . | 8.58 ( 4.66 - 12.49) |
| -0.26 ( -6.76 - 6.23) | 3.67 ( -4.23 - 11.56) | -5.76 (-12.93 - 1.41) | -0.92 ( -6.90 - 5.06) | -2.99 ( -9.79 - 3.81) | 1.34 ( -8.17 - 10.85) | -3.33 (-13.38 - 6.73) | -1.68 ( -8.28 - 4.92) | -2.72 ( -9.68 - 4.24) | 0.42 ( -6.39 - 7.23) | Kanglixin Capsules+OX | . | . | . | . | . | . | . | . | . | 8.16 ( 2.59 - 13.73) |
| -1.54 ( -8.10 - 5.03) | 2.39 ( -5.56 - 10.34) | -7.04 (-14.27 - 0.20) | -2.20 ( -8.25 - 3.86) | -4.26 (-11.13 - 2.61) | 0.07 ( -9.49 - 9.62) | -4.60 (-14.70 - 5.50) | -2.96 ( -9.62 - 3.71) | -4.00 (-11.02 - 3.03) | -0.85 ( -7.73 - 6.02) | -1.27 ( -9.21 - 6.66) | Lentinan+OX | . | . | . | . | . | . | . | . | 9.43 ( 3.78 - 15.08) |
| 0.66 ( -7.89 - 9.22) | 4.59 ( -5.06 - 14.25) | -4.83 (-13.91 - 4.24) | 0.01 ( -8.16 - 8.17) | -2.06 (-10.85 - 6.72) | 2.27 ( -8.75 - 13.29) | -2.40 (-13.89 - 9.09) | -0.75 ( -9.38 - 7.88) | -1.80 (-10.70 - 7.11) | 1.35 ( -7.44 - 10.13) | 0.93 ( -8.71 - 10.57) | 2.20 ( -7.48 - 11.89) | Pingxiao Capsules+OX | . | . | . | . | . | . | . | 7.23 ( -0.64 - 15.10) |
| -0.44 ( -8.82 - 7.95) | 3.49 ( -6.02 - 13.01) | -5.93 (-14.86 - 2.99) | -1.09 ( -9.09 - 6.90) | -3.16 (-11.79 - 5.47) | 1.17 ( -9.72 - 12.06) | -3.50 (-14.87 - 7.87) | -1.85 (-10.32 - 6.62) | -2.90 (-11.65 - 5.86) | 0.25 ( -8.39 - 8.88) | -0.17 ( -9.67 - 9.32) | 1.10 ( -8.44 - 10.65) | -1.10 (-12.11 - 9.91) | Shenmai Injection+OX | . | . | . | . | . | . | 8.33 ( 0.64 - 16.02) |
| 2.17 ( -1.75 - 6.09) | 6.10 ( 0.15 - 12.06) | -3.33 ( -8.29 - 1.63) | 1.51 ( -1.47 - 4.50) | -0.55 ( -4.96 - 3.85) | 3.78 ( -4.20 - 11.75) | -0.89 ( -9.51 - 7.72) | 0.75 ( -3.33 - 4.84) | -0.29 ( -4.94 - 4.36) | 2.85 ( -1.56 - 7.27) | 2.43 ( -3.50 - 8.36) | 3.71 ( -2.30 - 9.71) | 1.51 ( -6.62 - 9.64) | 2.61 ( -5.35 - 10.57) | Shenqi Fuzheng Injection+OX | . | . | . | . | . | 5.72 ( 3.68 - 7.76) |
| -1.94 ( -8.41 - 4.52) | 1.99 ( -5.88 - 9.85) | -7.44 (-14.59 - -0.30) | -2.60 ( -8.55 - 3.34) | -4.67 (-11.44 - 2.10) | -0.34 ( -9.83 - 9.15) | -5.01 (-15.04 - 5.02) | -3.36 ( -9.93 - 3.20) | -4.40 (-11.33 - 2.53) | -1.26 ( -8.04 - 5.51) | -1.68 ( -9.53 - 6.17) | -0.41 ( -8.31 - 7.50) | -2.61 (-12.23 - 7.01) | -1.51 (-10.98 - 7.97) | -4.12 (-10.01 - 1.78) | Xiaoaiping Injection+OX | . | . | . | . | 9.84 ( 4.31 - 15.37) |
| 3.45 ( -3.06 - 9.96) | 7.38 ( -0.53 - 15.29) | -2.05 ( -9.23 - 5.14) | 2.79 ( -3.21 - 8.79) | 0.72 ( -6.09 - 7.54) | 5.06 ( -4.46 - 14.57) | 0.39 ( -9.68 - 10.45) | 2.03 ( -4.58 - 8.64) | 0.99 ( -5.98 - 7.96) | 4.13 ( -2.69 - 10.95) | 3.71 ( -4.17 - 11.60) | 4.99 ( -2.96 - 12.93) | 2.79 ( -6.86 - 12.43) | 3.89 ( -5.62 - 13.39) | 1.28 ( -4.67 - 7.22) | 5.39 ( -2.47 - 13.25) | Xihuang Capsules+OX | . | . | . | 4.44 ( -1.14 - 10.03) |
| 2.66 ( -3.97 - 9.29) | 6.59 ( -1.41 - 14.59) | -2.84 (-10.13 - 4.45) | 2.00 ( -4.12 - 8.12) | -0.07 ( -7.00 - 6.86) | 4.26 ( -5.34 - 13.86) | -0.41 (-10.54 - 9.73) | 1.24 ( -5.49 - 7.97) | 0.20 ( -6.88 - 7.28) | 3.34 ( -3.59 - 10.27) | 2.92 ( -5.06 - 10.90) | 4.19 ( -3.84 - 12.23) | 1.99 ( -7.73 - 11.72) | 3.09 ( -6.49 - 12.68) | 0.49 ( -5.59 - 6.56) | 4.60 ( -3.35 - 12.56) | -0.79 ( -8.79 - 7.20) | Ya Dan Zi Oil Emulsion Injection+OX | . | . | 5.24 ( -0.48 - 10.96) |
| 2.78 ( -5.75 - 11.32) | 6.71 ( -2.93 - 16.35) | -2.71 (-11.77 - 6.34) | 2.13 ( -6.02 - 10.27) | 0.06 ( -8.71 - 8.83) | 4.39 ( -6.61 - 15.39) | -0.28 (-11.75 - 11.19) | 1.37 ( -7.25 - 9.98) | 0.32 ( -8.57 - 9.21) | 3.47 ( -5.30 - 12.24) | 3.05 ( -6.58 - 12.67) | 4.32 ( -5.35 - 13.99) | 2.12 ( -8.99 - 13.23) | 3.22 ( -7.77 - 14.21) | 0.61 ( -7.50 - 8.72) | 4.73 ( -4.87 - 14.33) | -0.67 (-10.30 - 8.97) | 0.13 ( -9.59 - 9.84) | Yangzheng Xiaoji Capsules+OX | . | 5.11 ( -2.74 - 12.96) |
| -1.21 ( -9.84 - 7.43) | 2.72 ( -7.01 - 12.45) | -6.70 (-15.86 - 2.45) | -1.86 (-10.12 - 6.39) | -3.93 (-12.80 - 4.94) | 0.40 (-10.68 - 11.48) | -4.27 (-15.82 - 7.28) | -2.62 (-11.34 - 6.09) | -3.67 (-12.65 - 5.32) | -0.52 ( -9.39 - 8.35) | -0.94 (-10.66 - 8.77) | 0.33 ( -9.43 - 10.09) | -1.87 (-13.06 - 9.32) | -0.77 (-11.84 - 10.30) | -3.38 (-11.60 - 4.84) | 0.74 ( -8.95 - 10.43) | -4.66 (-14.38 - 5.07) | -3.86 (-13.67 - 5.94) | -3.99 (-15.17 - 7.19) | Zhenqi Fuzheng Granules+OX | 9.10 ( 1.14 - 17.06) |
| 7.89 ( 4.55 - 11.24) | 11.82 ( 6.23 - 17.42) | 2.40 ( -2.12 - 6.92) | 7.24 ( 5.05 - 9.42) | 5.17 ( 1.26 - 9.08) | 9.50 ( 1.79 - 17.21) | 4.83 ( -3.54 - 13.20) | 6.48 ( 2.93 - 10.02) | 5.43 ( 1.26 - 9.61) | 8.58 ( 4.66 - 12.49) | 8.16 ( 2.59 - 13.73) | 9.43 ( 3.78 - 15.08) | 7.23 ( -0.64 - 15.10) | 8.33 ( 0.64 - 16.02) | 5.72 ( 3.68 - 7.76) | 9.84 ( 4.31 - 15.37) | 4.44 ( -1.14 - 10.03) | 5.24 ( -0.48 - 10.96) | 5.11 ( -2.74 - 12.96) | 9.10 ( 1.14 - 17.06) | OX |

1. **CD4+CD8+**

| Aidi Injection+OX | . | . | . | . | . | . | . | . | . | . | . | . | . | . | . | . | . | . | . | 0.45 ( 0.14 - 0.76) |
| --- | --- | --- | --- | --- | --- | --- | --- | --- | --- | --- | --- | --- | --- | --- | --- | --- | --- | --- | --- | --- |
| -0.17 (-0.59 - 0.24) | Astragalus Polysaccharides+OX | . | . | . | . | . | . | . | . | . | . | . | . | . | . | . | . | . | . | 0.62 ( 0.34 - 0.89) |
| 0.06 (-0.32 - 0.45) | 0.23 (-0.12 - 0.59) | Astragalus preparations+OX | . | . | . | . | . | . | . | . | . | . | . | . | . | . | . | . | . | 0.38 ( 0.16 - 0.61) |
| -0.02 (-0.35 - 0.31) | 0.15 (-0.14 - 0.45) | -0.08 (-0.33 - 0.16) | Compound Kushen Injection+OX | . | . | . | . | . | . | . | . | . | . | . | . | . | . | . | . | 0.47 ( 0.36 - 0.58) |
| 0.30 (-0.08 - 0.68) | 0.47 ( 0.12 - 0.82) | 0.24 (-0.07 - 0.55) | 0.32 ( 0.08 - 0.56) | Compound Mylabris preparations+OX | . | . | . | . | . | . | . | . | . | . | . | . | . | . | . | 0.15 (-0.07 - 0.36) |
| -0.21 (-0.70 - 0.28) | -0.04 (-0.51 - 0.42) | -0.28 (-0.71 - 0.16) | -0.19 (-0.59 - 0.20) | -0.51 (-0.95 - -0.08) | Diyu Shengbai Tablet+OX | . | . | . | . | . | . | . | . | . | . | . | . | . | . | 0.66 ( 0.28 - 1.04) |
| 0.21 (-0.31 - 0.72) | 0.38 (-0.12 - 0.87) | 0.14 (-0.32 - 0.61) | 0.23 (-0.20 - 0.65) | -0.09 (-0.56 - 0.37) | 0.42 (-0.14 - 0.98) | Ginseng Polysaccharide Injection+OX | . | . | . | . | . | . | . | . | . | . | . | . | . | 0.24 (-0.17 - 0.65) |
| -0.02 (-0.40 - 0.35) | 0.15 (-0.20 - 0.49) | -0.09 (-0.39 - 0.22) | -0.00 (-0.24 - 0.24) | -0.32 (-0.62 - -0.02) | 0.19 (-0.24 - 0.62) | -0.23 (-0.69 - 0.23) | Huachansu preparations+OX | . | . | . | . | . | . | . | . | . | . | . | . | 0.47 ( 0.26 - 0.68) |
| 0.04 (-0.33 - 0.41) | 0.21 (-0.13 - 0.55) | -0.02 (-0.33 - 0.28) | 0.06 (-0.17 - 0.29) | -0.26 (-0.56 - 0.03) | 0.25 (-0.18 - 0.68) | -0.17 (-0.63 - 0.29) | 0.06 (-0.23 - 0.35) | Jinlong Capsules+OX | . | . | . | . | . | . | . | . | . | . | . | 0.41 ( 0.20 - 0.61) |
| 0.20 (-0.18 - 0.59) | 0.37 ( 0.02 - 0.73) | 0.14 (-0.18 - 0.46) | 0.22 (-0.03 - 0.48) | -0.10 (-0.41 - 0.21) | 0.41 (-0.03 - 0.86) | -0.01 (-0.48 - 0.47) | 0.22 (-0.09 - 0.53) | 0.16 (-0.15 - 0.47) | Kangai Injection+OX | . | . | . | . | . | . | . | . | . | . | 0.25 ( 0.02 - 0.47) |
| -0.18 (-0.60 - 0.23) | -0.01 (-0.40 - 0.38) | -0.25 (-0.60 - 0.11) | -0.16 (-0.46 - 0.13) | -0.49 (-0.84 - -0.14) | 0.03 (-0.44 - 0.50) | -0.39 (-0.89 - 0.10) | -0.16 (-0.51 - 0.18) | -0.22 (-0.57 - 0.12) | -0.39 (-0.74 - -0.03) | Kanglixin Capsules+OX | . | . | . | . | . | . | . | . | . | 0.63 ( 0.36 - 0.91) |
| -0.01 (-0.43 - 0.41) | 0.17 (-0.23 - 0.56) | -0.07 (-0.43 - 0.29) | 0.01 (-0.29 - 0.32) | -0.31 (-0.66 - 0.05) | 0.21 (-0.26 - 0.68) | -0.21 (-0.71 - 0.29) | 0.02 (-0.34 - 0.37) | -0.04 (-0.39 - 0.30) | -0.21 (-0.57 - 0.16) | 0.18 (-0.22 - 0.57) | Lentinan+OX | . | . | . | . | . | . | . | . | 0.45 ( 0.17 - 0.74) |
| 6.81 ( 4.96 - 8.65) | 6.98 ( 5.14 - 8.82) | 6.74 ( 4.91 - 8.58) | 6.83 ( 5.01 - 8.65) | 6.51 ( 4.67 - 8.34) | 7.02 ( 5.16 - 8.88) | 6.60 ( 4.74 - 8.46) | 6.83 ( 5.00 - 8.66) | 6.77 ( 4.94 - 8.60) | 6.61 ( 4.77 - 8.44) | 6.99 ( 5.15 - 8.83) | 6.81 ( 4.97 - 8.65) | Pingxiao Capsules+OX | . | . | . | . | . | . | . | -6.36 (-8.18 - -4.54) |
| 0.10 (-0.28 - 0.48) | 0.27 (-0.08 - 0.62) | 0.04 (-0.27 - 0.35) | 0.12 (-0.12 - 0.36) | -0.20 (-0.51 - 0.10) | 0.31 (-0.12 - 0.75) | -0.11 (-0.57 - 0.36) | 0.12 (-0.18 - 0.42) | 0.06 (-0.24 - 0.36) | -0.10 (-0.42 - 0.21) | 0.28 (-0.07 - 0.63) | 0.11 (-0.25 - 0.46) | -6.71 (-8.54 - -4.88) | Shenmai Injection+OX | . | . | . | . | . | . | 0.35 ( 0.13 - 0.56) |
| 0.13 (-0.20 - 0.46) | 0.30 ( 0.01 - 0.60) | 0.07 (-0.18 - 0.32) | 0.15 (-0.01 - 0.31) | -0.17 (-0.41 - 0.08) | 0.35 (-0.05 - 0.74) | -0.07 (-0.50 - 0.35) | 0.15 (-0.09 - 0.39) | 0.09 (-0.14 - 0.33) | -0.07 (-0.33 - 0.19) | 0.32 ( 0.02 - 0.62) | 0.14 (-0.17 - 0.44) | -6.67 (-8.50 - -4.85) | 0.03 (-0.21 - 0.28) | Shenqi Fuzheng Injection+OX | . | . | . | . | . | 0.31 ( 0.20 - 0.43) |
| 0.16 (-0.25 - 0.58) | 0.33 (-0.05 - 0.72) | 0.10 (-0.25 - 0.45) | 0.18 (-0.11 - 0.48) | -0.14 (-0.49 - 0.21) | 0.38 (-0.09 - 0.84) | -0.04 (-0.54 - 0.45) | 0.18 (-0.16 - 0.53) | 0.12 (-0.22 - 0.47) | -0.04 (-0.40 - 0.32) | 0.35 (-0.04 - 0.74) | 0.17 (-0.23 - 0.56) | -6.64 (-8.48 - -4.80) | 0.06 (-0.29 - 0.41) | 0.03 (-0.27 - 0.33) | Xiaoaiping Injection+OX | . | . | . | . | 0.28 ( 0.01 - 0.56) |
| 0.33 (-0.10 - 0.75) | 0.50 ( 0.10 - 0.89) | 0.26 (-0.10 - 0.62) | 0.35 ( 0.04 - 0.65) | 0.02 (-0.33 - 0.38) | 0.54 ( 0.07 - 1.01) | 0.12 (-0.38 - 0.62) | 0.35 (-0.01 - 0.70) | 0.29 (-0.06 - 0.64) | 0.12 (-0.24 - 0.49) | 0.51 ( 0.11 - 0.91) | 0.33 (-0.07 - 0.73) | -6.48 (-8.32 - -4.64) | 0.23 (-0.13 - 0.58) | 0.19 (-0.11 - 0.50) | 0.16 (-0.23 - 0.56) | Xihuang Capsules+OX | . | . | . | 0.12 (-0.16 - 0.41) |
| -0.13 (-0.56 - 0.31) | 0.05 (-0.37 - 0.46) | -0.19 (-0.57 - 0.19) | -0.11 (-0.43 - 0.22) | -0.43 (-0.80 - -0.05) | 0.09 (-0.40 - 0.58) | -0.33 (-0.85 - 0.18) | -0.10 (-0.48 - 0.27) | -0.16 (-0.54 - 0.21) | -0.33 (-0.71 - 0.06) | 0.06 (-0.36 - 0.47) | -0.12 (-0.54 - 0.30) | -6.93 (-8.78 - -5.09) | -0.23 (-0.60 - 0.15) | -0.26 (-0.59 - 0.07) | -0.29 (-0.70 - 0.13) | -0.45 (-0.87 - -0.03) | Ya Dan Zi Oil Emulsion Injection+OX | . | . | 0.57 ( 0.26 - 0.88) |
| 0.10 (-0.40 - 0.59) | 0.27 (-0.20 - 0.74) | 0.03 (-0.41 - 0.48) | 0.12 (-0.28 - 0.52) | -0.20 (-0.65 - 0.24) | 0.31 (-0.23 - 0.85) | -0.11 (-0.67 - 0.45) | 0.12 (-0.32 - 0.56) | 0.06 (-0.38 - 0.49) | -0.10 (-0.55 - 0.34) | 0.28 (-0.19 - 0.75) | 0.10 (-0.38 - 0.58) | -6.71 (-8.57 - -4.85) | -0.00 (-0.45 - 0.44) | -0.04 (-0.44 - 0.37) | -0.07 (-0.54 - 0.41) | -0.23 (-0.71 - 0.25) | 0.22 (-0.27 - 0.72) | Yangzheng Xiaoji Capsules+OX | . | 0.35 (-0.03 - 0.73) |
| -0.37 (-0.87 - 0.12) | -0.20 (-0.67 - 0.27) | -0.44 (-0.88 - 0.01) | -0.35 (-0.75 - 0.05) | -0.67 (-1.12 - -0.23) | -0.16 (-0.70 - 0.38) | -0.58 (-1.14 - -0.02) | -0.35 (-0.79 - 0.09) | -0.41 (-0.85 - 0.02) | -0.57 (-1.02 - -0.13) | -0.19 (-0.66 - 0.28) | -0.37 (-0.84 - 0.11) | -7.18 (-9.04 - -5.32) | -0.47 (-0.91 - -0.03) | -0.51 (-0.91 - -0.10) | -0.54 (-1.01 - -0.06) | -0.70 (-1.18 - -0.22) | -0.25 (-0.74 - 0.25) | -0.47 (-1.01 - 0.07) | Zhenqi Fuzheng Granules+OX | 0.82 ( 0.44 - 1.20) |
| 0.45 ( 0.14 - 0.76) | 0.62 ( 0.34 - 0.89) | 0.38 ( 0.16 - 0.61) | 0.47 ( 0.36 - 0.58) | 0.15 (-0.07 - 0.36) | 0.66 ( 0.28 - 1.04) | 0.24 (-0.17 - 0.65) | 0.47 ( 0.26 - 0.68) | 0.41 ( 0.20 - 0.61) | 0.25 ( 0.02 - 0.47) | 0.63 ( 0.36 - 0.91) | 0.45 ( 0.17 - 0.74) | -6.36 (-8.18 - -4.54) | 0.35 ( 0.13 - 0.56) | 0.31 ( 0.20 - 0.43) | 0.28 ( 0.01 - 0.56) | 0.12 (-0.16 - 0.41) | 0.57 ( 0.26 - 0.88) | 0.35 (-0.03 - 0.73) | 0.82 ( 0.44 - 1.20) | OX |

1. **CD8+**

| Aidi Injection+OX | . | . | . | . | . | . | . | . | . | . | . | . | . | . | . | . | . | . | . | -1.08 ( -6.01 - 3.86) |
| --- | --- | --- | --- | --- | --- | --- | --- | --- | --- | --- | --- | --- | --- | --- | --- | --- | --- | --- | --- | --- |
| 2.91 ( -6.17 - 11.99) | Astragalus Polysaccharides+OX | . | . | . | . | . | . | . | . | . | . | . | . | . | . | . | . | . | . | -3.99 ( -11.61 - 3.63) |
| 0.36 ( -7.58 - 8.30) | -2.55 ( -12.39 - 7.29) | Astragalus preparations+OX | . | . | . | . | . | . | . | . | . | . | . | . | . | . | . | . | . | -1.44 ( -7.66 - 4.78) |
| 2.57 ( -3.27 - 8.40) | -0.35 ( -8.58 - 7.88) | 2.20 ( -4.75 - 9.16) | Compound Kushen Injection+OX | . | . | . | . | . | . | . | . | . | . | . | . | . | . | . | . | -3.64 ( -6.75 - -0.53) |
| -0.54 ( -8.47 - 7.39) | -3.45 ( -13.28 - 6.38) | -0.90 ( -9.69 - 7.89) | -3.10 ( -10.05 - 3.84) | Compound Mylabris preparations+OX | . | . | . | . | . | . | . | . | . | . | . | . | . | . | . | -0.54 ( -6.75 - 5.67) |
| 5.74 ( -6.05 - 17.54) | 2.83 ( -10.31 - 15.98) | 5.38 ( -7.00 - 17.77) | 3.18 ( -7.97 - 14.33) | 6.28 ( -6.10 - 18.66) | Diyu Shengbai Tablet+OX | . | . | . | . | . | . | . | . | . | . | . | . | . | . | -6.82 ( -17.53 - 3.89) |
| -1.19 ( -13.05 - 10.68) | -4.10 ( -17.30 - 9.11) | -1.55 ( -14.00 - 10.91) | -3.75 ( -14.98 - 7.48) | -0.65 ( -13.09 - 11.80) | -6.93 ( -22.13 - 8.27) | Ginseng Polysaccharide Injection+OX | . | . | . | . | . | . | . | . | . | . | . | . | . | 0.11 ( -10.68 - 10.90) |
| -1.53 ( -8.85 - 5.78) | -4.44 ( -13.78 - 4.90) | -1.89 ( -10.13 - 6.35) | -4.10 ( -10.33 - 2.14) | -0.99 ( -9.22 - 7.24) | -7.28 ( -19.27 - 4.72) | -0.35 ( -12.41 - 11.72) | Huachansu preparations+OX | . | . | . | . | . | . | . | . | . | . | . | . | 0.46 ( -4.94 - 5.86) |
| 4.09 ( -3.97 - 12.14) | 1.18 ( -8.76 - 11.11) | 3.73 ( -5.18 - 12.63) | 1.52 ( -5.57 - 8.61) | 4.63 ( -4.27 - 13.52) | -1.66 ( -14.12 - 10.81) | 5.27 ( -7.26 - 17.80) | 5.62 ( -2.73 - 13.97) | Jinlong Capsules+OX | . | . | . | . | . | . | . | . | . | . | . | -5.16 ( -11.54 - 1.21) |
| -0.85 ( -8.16 - 6.46) | -3.76 ( -13.10 - 5.58) | -1.21 ( -9.45 - 7.03) | -3.41 ( -9.64 - 2.81) | -0.31 ( -8.54 - 7.92) | -6.59 ( -18.59 - 5.40) | 0.34 ( -11.73 - 12.40) | 0.68 ( -6.95 - 8.32) | -4.94 ( -13.29 - 3.42) | Kangai Injection+OX | . | . | . | . | . | . | . | . | . | . | -0.23 ( -5.62 - 5.17) |
| 4.93 ( -4.18 - 14.04) | 2.02 ( -8.78 - 12.83) | 4.57 ( -5.29 - 14.44) | 2.37 ( -5.90 - 10.64) | 5.47 ( -4.39 - 15.33) | -0.81 ( -13.98 - 12.36) | 6.12 ( -7.11 - 19.35) | 6.47 ( -2.91 - 15.84) | 0.85 ( -9.12 - 10.81) | 5.78 ( -3.59 - 15.15) | Kanglixin Capsules+OX | . | . | . | . | . | . | . | . | . | -6.01 ( -13.67 - 1.65) |
| 3.78 ( -8.09 - 15.65) | 0.87 ( -12.34 - 14.09) | 3.42 ( -9.04 - 15.88) | 1.22 ( -10.02 - 12.46) | 4.32 ( -8.13 - 16.78) | -1.96 ( -17.17 - 13.25) | 4.97 ( -10.29 - 20.23) | 5.32 ( -6.76 - 17.39) | -0.30 ( -12.84 - 12.23) | 4.63 ( -7.44 - 16.70) | -1.15 ( -14.39 - 12.09) | Lentinan+OX | . | . | . | . | . | . | . | . | -4.86 ( -15.66 - 5.94) |
| -1.58 ( -13.34 - 10.18) | -4.49 ( -17.60 - 8.63) | -1.94 ( -14.29 - 10.42) | -4.14 ( -15.26 - 6.98) | -1.04 ( -13.39 - 11.31) | -7.32 ( -22.44 - 7.80) | -0.39 ( -15.57 - 14.79) | -0.04 ( -12.01 - 11.92) | -5.66 ( -18.10 - 6.77) | -0.73 ( -12.69 - 11.24) | -6.51 ( -19.65 - 6.63) | -5.36 ( -20.55 - 9.83) | Pingxiao Capsules+OX | . | . | . | . | . | . | . | 0.50 ( -10.18 - 11.18) |
| 43.92 ( -49.37 - 137.21) | 41.01 ( -52.46 - 134.48) | 43.56 ( -49.80 - 136.93) | 41.36 ( -51.85 - 134.57) | 44.46 ( -48.90 - 137.83) | 38.18 ( -55.59 - 131.95) | 45.11 ( -48.67 - 138.89) | 45.46 ( -47.86 - 138.77) | 39.84 ( -53.54 - 133.21) | 44.77 ( -48.54 - 138.09) | 38.99 ( -54.48 - 132.46) | 40.14 ( -53.64 - 133.92) | 45.50 ( -48.27 - 139.27) | Shenmai Injection+OX | . | . | . | . | . | . | -45.00 (-138.16 - 48.16) |
| -1.13 ( -6.97 - 4.72) | -4.04 ( -12.28 - 4.20) | -1.49 ( -8.45 - 5.48) | -3.69 ( -8.11 - 0.73) | -0.59 ( -7.54 - 6.37) | -6.87 ( -18.03 - 4.29) | 0.06 ( -11.18 - 11.29) | 0.40 ( -5.84 - 6.65) | -5.21 ( -12.32 - 1.89) | -0.28 ( -6.52 - 5.96) | -6.06 ( -14.34 - 2.22) | -4.91 ( -16.16 - 6.33) | 0.45 ( -10.68 - 11.58) | -45.05 (-138.26 - 48.16) | Shenqi Fuzheng Injection+OX | . | . | . | . | . | 0.05 ( -3.09 - 3.19) |
| -5.77 ( -14.86 - 3.31) | -8.68 ( -19.47 - 2.10) | -6.13 ( -15.98 - 3.71) | -8.34 ( -16.58 - -0.10) | -5.23 ( -15.07 - 4.60) | -11.52 ( -24.67 - 1.63) | -4.59 ( -17.80 - 8.63) | -4.24 ( -13.59 - 5.11) | -9.86 ( -19.80 - 0.08) | -4.92 ( -14.27 - 4.42) | -10.71 ( -21.52 - 0.11) | -9.56 ( -22.78 - 3.67) | -4.20 ( -17.32 - 8.93) | -49.70 (-143.17 - 43.77) | -4.65 ( -12.90 - 3.61) | Xiaoaiping Injection+OX | . | . | . | . | 4.70 ( -2.93 - 12.33) |
| -0.95 ( -10.13 - 8.23) | -3.86 ( -14.72 - 7.00) | -1.31 ( -11.24 - 8.62) | -3.52 ( -11.86 - 4.83) | -0.41 ( -10.34 - 9.51) | -6.69 ( -19.91 - 6.52) | 0.24 ( -13.04 - 13.51) | 0.58 ( -8.86 - 10.02) | -5.04 ( -15.06 - 4.99) | -0.10 ( -9.54 - 9.34) | -5.89 ( -16.78 - 5.01) | -4.73 ( -18.02 - 8.55) | 0.63 ( -12.56 - 13.81) | -44.87 (-138.36 - 48.61) | 0.18 ( -8.18 - 8.53) | 4.82 ( -6.05 - 15.69) | Xihuang Capsules+OX | . | . | . | -0.13 ( -7.87 - 7.62) |
| 1.38 ( -7.76 - 10.52) | -1.53 ( -12.36 - 9.30) | 1.02 ( -8.88 - 10.92) | -1.19 ( -9.49 - 7.12) | 1.92 ( -7.97 - 11.81) | -4.36 ( -17.55 - 8.83) | 2.57 ( -10.69 - 15.82) | 2.91 ( -6.49 - 12.32) | -2.71 ( -12.70 - 7.29) | 2.23 ( -7.17 - 11.63) | -3.55 ( -14.41 - 7.31) | -2.40 ( -15.66 - 10.86) | 2.96 ( -10.21 - 16.12) | -42.54 (-136.02 - 50.93) | 2.51 ( -5.81 - 10.82) | 7.15 ( -3.69 - 17.99) | 2.33 ( -8.59 - 13.25) | Ya Dan Zi Oil Emulsion Injection+OX | . | . | -2.46 ( -10.15 - 5.24) |
| 6.47 ( -5.34 - 18.29) | 3.56 ( -9.61 - 16.73) | 6.11 ( -6.30 - 18.53) | 3.91 ( -7.27 - 15.09) | 7.01 ( -5.39 - 19.42) | 0.73 ( -14.44 - 15.90) | 7.66 ( -7.56 - 22.88) | 8.01 ( -4.02 - 20.03) | 2.39 ( -10.10 - 14.88) | 7.32 ( -4.70 - 19.34) | 1.54 ( -11.65 - 14.73) | 2.69 ( -12.54 - 17.92) | 8.05 ( -7.10 - 23.20) | -37.45 (-131.23 - 56.33) | 7.60 ( -3.59 - 18.79) | 12.25 ( -0.93 - 25.42) | 7.42 ( -5.81 - 20.66) | 5.09 ( -8.12 - 18.31) | Yangzheng Xiaoji Capsules+OX | . | -7.55 ( -18.29 - 3.19) |
| 3.72 ( -8.14 - 15.59) | 0.81 ( -12.40 - 14.02) | 3.36 ( -9.09 - 15.82) | 1.16 ( -10.07 - 12.39) | 4.26 ( -8.19 - 16.71) | -2.02 ( -17.23 - 13.19) | 4.91 ( -10.35 - 20.17) | 5.26 ( -6.81 - 17.32) | -0.36 ( -12.90 - 12.17) | 4.57 ( -7.49 - 16.64) | -1.21 ( -14.44 - 12.02) | -0.06 ( -15.33 - 15.21) | 5.30 ( -9.88 - 20.48) | -40.20 (-133.98 - 53.58) | 4.85 ( -6.39 - 16.09) | 9.50 ( -3.72 - 22.72) | 4.67 ( -8.61 - 17.96) | 2.34 ( -10.91 - 15.60) | -2.75 ( -17.98 - 12.48) | Zhenqi Fuzheng Granules+OX | -4.80 ( -15.59 - 5.99) |
| -1.08 ( -6.01 - 3.86) | -3.99 ( -11.61 - 3.63) | -1.44 ( -7.66 - 4.78) | -3.64 ( -6.75 - -0.53) | -0.54 ( -6.75 - 5.67) | -6.82 ( -17.53 - 3.89) | 0.11 ( -10.68 - 10.90) | 0.46 ( -4.94 - 5.86) | -5.16 ( -11.54 - 1.21) | -0.23 ( -5.62 - 5.17) | -6.01 ( -13.67 - 1.65) | -4.86 ( -15.66 - 5.94) | 0.50 ( -10.18 - 11.18) | -45.00 (-138.16 - 48.16) | 0.05 ( -3.09 - 3.19) | 4.70 ( -2.93 - 12.33) | -0.13 ( -7.87 - 7.62) | -2.46 ( -10.15 - 5.24) | -7.55 ( -18.29 - 3.19) | -4.80 ( -15.59 - 5.99) | OX |

**N. Traditional Chinese medicine (TCM) syndrome score**

| Aidi Injection+OX | . | . | . | -2.69 (-6.05 - 0.67) |
| --- | --- | --- | --- | --- |
| 1.07 (-3.03 - 5.18) | Compound Kushen Injection+OX | . | . | -3.76 (-6.12 - -1.41) |
| -4.34 (-8.22 - -0.46) | -5.41 (-8.47 - -2.36) | Shenqi Fuzheng Injection+OX | . | 1.65 (-0.29 - 3.60) |
| -1.48 (-6.09 - 3.13) | -2.55 (-6.49 - 1.38) | 2.86 (-0.85 - 6.57) | Yangzheng Xiaoji Capsules+OX | -1.21 (-4.36 - 1.94) |
| -2.69 (-6.05 - 0.67) | -3.76 (-6.12 - -1.41) | 1.65 (-0.29 - 3.60) | -1.21 (-4.36 - 1.94) | OX |

**O. Improvement rate in quality of life**

| Aidi Injection+OX | . | . | . | . | . | . | . | . | . | . | . | . | . | . | . | 1.30 (1.13 - 1.50) |
| --- | --- | --- | --- | --- | --- | --- | --- | --- | --- | --- | --- | --- | --- | --- | --- | --- |
| 0.77 (0.55 - 1.08) | Astragalus Polysaccharides+OX | . | . | . | . | . | . | . | . | . | . | . | . | . | . | 1.68 (1.24 - 2.28) |
| 0.60 (0.35 - 1.01) | 0.77 (0.43 - 1.39) | Astragalus preparations+OX | . | . | . | . | . | . | . | . | . | . | . | . | . | 2.18 (1.32 - 3.61) |
| 1.09 (0.87 - 1.36) | 1.40 (0.99 - 1.99) | 1.82 (1.07 - 3.10) | Compound Kushen Injection+OX | . | . | . | . | . | . | . | . | . | . | . | . | 1.20 (1.01 - 1.42) |
| 1.00 (0.72 - 1.38) | 1.29 (0.85 - 1.97) | 1.68 (0.94 - 3.00) | 0.92 (0.66 - 1.29) | Compound Mylabris preparations+OX | . | . | . | . | . | . | . | . | . | . | . | 1.30 (0.97 - 1.74) |
| 1.11 (0.83 - 1.48) | 1.43 (0.96 - 2.12) | 1.86 (1.06 - 3.26) | 1.02 (0.75 - 1.38) | 1.11 (0.75 - 1.62) | Huachansu preparations+OX | . | . | . | . | . | . | . | . | . | . | 1.18 (0.91 - 1.51) |
| 0.89 (0.65 - 1.22) | 1.15 (0.76 - 1.74) | 1.49 (0.84 - 2.65) | 0.82 (0.59 - 1.14) | 0.89 (0.59 - 1.33) | 0.80 (0.55 - 1.17) | Kangai Injection+OX | . | . | . | . | . | . | . | . | . | 1.46 (1.11 - 1.94) |
| 1.06 (0.80 - 1.41) | 1.37 (0.93 - 2.03) | 1.78 (1.02 - 3.12) | 0.98 (0.72 - 1.32) | 1.06 (0.73 - 1.55) | 0.96 (0.68 - 1.37) | 1.19 (0.82 - 1.73) | Kanglaite Injection+OX | . | . | . | . | . | . | . | . | 1.23 (0.96 - 1.57) |
| 1.03 (0.81 - 1.30) | 1.33 (0.93 - 1.90) | 1.72 (1.00 - 2.95) | 0.94 (0.73 - 1.22) | 1.03 (0.72 - 1.45) | 0.93 (0.68 - 1.27) | 1.15 (0.82 - 1.62) | 0.97 (0.71 - 1.32) | Lentinan+OX | . | . | . | . | . | . | . | 1.27 (1.05 - 1.54) |
| 0.96 (0.62 - 1.48) | 1.24 (0.74 - 2.06) | 1.61 (0.84 - 3.07) | 0.88 (0.56 - 1.37) | 0.96 (0.58 - 1.58) | 0.87 (0.53 - 1.40) | 1.08 (0.65 - 1.77) | 0.90 (0.56 - 1.45) | 0.93 (0.59 - 1.47) | Pingxiao Capsules+OX | . | . | . | . | . | . | 1.36 (0.90 - 2.05) |
| 1.26 (0.89 - 1.79) | 1.63 (1.05 - 2.53) | 2.11 (1.16 - 3.84) | 1.16 (0.81 - 1.67) | 1.26 (0.82 - 1.94) | 1.14 (0.76 - 1.71) | 1.42 (0.93 - 2.17) | 1.19 (0.79 - 1.77) | 1.23 (0.85 - 1.78) | 1.32 (0.78 - 2.21) | Shenfu Injection+OX | . | . | . | . | . | 1.03 (0.75 - 1.42) |
| 1.09 (0.80 - 1.48) | 1.40 (0.93 - 2.11) | 1.82 (1.03 - 3.23) | 1.00 (0.72 - 1.38) | 1.08 (0.73 - 1.62) | 0.98 (0.68 - 1.43) | 1.22 (0.82 - 1.81) | 1.02 (0.71 - 1.48) | 1.06 (0.76 - 1.48) | 1.13 (0.69 - 1.86) | 0.86 (0.56 - 1.31) | Shenmai Injection+OX | . | . | . | . | 1.20 (0.91 - 1.58) |
| 1.00 (0.83 - 1.21) | 1.29 (0.93 - 1.80) | 1.68 (1.00 - 2.82) | 0.92 (0.74 - 1.14) | 1.00 (0.73 - 1.37) | 0.91 (0.68 - 1.20) | 1.13 (0.83 - 1.53) | 0.94 (0.72 - 1.24) | 0.98 (0.78 - 1.23) | 1.05 (0.68 - 1.61) | 0.80 (0.56 - 1.12) | 0.92 (0.68 - 1.25) | Shenqi Fuzheng Injection+OX | . | . | . | 1.30 (1.15 - 1.47) |
| 1.10 (0.74 - 1.64) | 1.42 (0.88 - 2.29) | 1.85 (0.99 - 3.45) | 1.01 (0.67 - 1.52) | 1.10 (0.69 - 1.76) | 1.00 (0.64 - 1.56) | 1.24 (0.78 - 1.97) | 1.04 (0.67 - 1.61) | 1.07 (0.71 - 1.63) | 1.15 (0.66 - 2.00) | 0.87 (0.54 - 1.42) | 1.01 (0.64 - 1.61) | 1.10 (0.75 - 1.62) | Xiaoaiping Injection+OX | . | . | 1.18 (0.82 - 1.71) |
| 1.03 (0.82 - 1.30) | 1.33 (0.94 - 1.90) | 1.73 (1.02 - 2.95) | 0.95 (0.74 - 1.22) | 1.03 (0.73 - 1.45) | 0.93 (0.69 - 1.27) | 1.16 (0.83 - 1.62) | 0.97 (0.72 - 1.32) | 1.01 (0.77 - 1.31) | 1.08 (0.69 - 1.69) | 0.82 (0.57 - 1.18) | 0.95 (0.68 - 1.32) | 1.03 (0.83 - 1.28) | 0.94 (0.62 - 1.41) | Ya Dan Zi Oil Emulsion Injection+OX | . | 1.26 (1.06 - 1.51) |
| 0.80 (0.57 - 1.12) | 1.03 (0.67 - 1.59) | 1.34 (0.74 - 2.41) | 0.74 (0.52 - 1.04) | 0.80 (0.52 - 1.22) | 0.72 (0.49 - 1.07) | 0.90 (0.59 - 1.36) | 0.75 (0.51 - 1.11) | 0.78 (0.54 - 1.12) | 0.83 (0.50 - 1.39) | 0.63 (0.41 - 0.99) | 0.74 (0.49 - 1.11) | 0.80 (0.57 - 1.11) | 0.73 (0.45 - 1.17) | 0.77 (0.54 - 1.10) | Yangzheng Xiaoji Capsules+OX | 1.63 (1.20 - 2.21) |
| 1.30 (1.13 - 1.50) | 1.68 (1.24 - 2.28) | 2.18 (1.32 - 3.61) | 1.20 (1.01 - 1.42) | 1.30 (0.97 - 1.74) | 1.18 (0.91 - 1.51) | 1.46 (1.11 - 1.94) | 1.23 (0.96 - 1.57) | 1.27 (1.05 - 1.54) | 1.36 (0.90 - 2.05) | 1.03 (0.75 - 1.42) | 1.20 (0.91 - 1.58) | 1.30 (1.15 - 1.47) | 1.18 (0.82 - 1.71) | 1.26 (1.06 - 1.51) | 1.63 (1.20 - 2.21) | OX |

**P. Myelosuppression event**

| Aidi Injection+OX | . | . | . | . | . | . | . | . | . | . | . | . | . | . | . | . | . | . | . | . | . | . | . | 0.65 (0.54 - 0.80) |
| --- | --- | --- | --- | --- | --- | --- | --- | --- | --- | --- | --- | --- | --- | --- | --- | --- | --- | --- | --- | --- | --- | --- | --- | --- |
| 1.50 (0.66 - 3.38) | Astragalus Polysaccharides+OX | . | . | . | . | . | . | . | . | . | . | . | . | . | . | . | . | . | . | . | . | . | . | 0.44 (0.20 - 0.96) |
| 1.14 (0.55 - 2.35) | 0.76 (0.27 - 2.18) | Astragalus preparations+OX | . | . | . | . | . | . | . | . | . | . | . | . | . | . | . | . | . | . | . | . | . | 0.57 (0.29 - 1.15) |
| 1.04 (0.79 - 1.37) | 0.70 (0.31 - 1.57) | 0.91 (0.44 - 1.88) | Compound Kushen Injection+OX | . | . | . | . | . | . | . | . | . | . | . | . | . | . | . | . | . | . | . | . | 0.63 (0.52 - 0.75) |
| 0.88 (0.61 - 1.27) | 0.59 (0.25 - 1.38) | 0.78 (0.36 - 1.66) | 0.85 (0.59 - 1.21) | Compound Mylabris preparations+OX | . | . | . | . | . | . | . | . | . | . | . | . | . | . | . | . | . | . | . | 0.74 (0.54 - 1.00) |
| 1.28 (0.63 - 2.61) | 0.85 (0.30 - 2.43) | 1.12 (0.42 - 2.99) | 1.23 (0.60 - 2.50) | 1.45 (0.68 - 3.07) | Ginseng Polysaccharide Injection+OX | . | . | . | . | . | . | . | . | . | . | . | . | . | . | . | . | . | . | 0.51 (0.26 - 1.01) |
| 0.93 (0.67 - 1.29) | 0.62 (0.27 - 1.43) | 0.82 (0.39 - 1.72) | 0.89 (0.65 - 1.23) | 1.05 (0.70 - 1.57) | 0.73 (0.35 - 1.51) | Huachansu preparations+OX | . | . | . | . | . | . | . | . | . | . | . | . | . | . | . | . | . | 0.70 (0.54 - 0.91) |
| 1.05 (0.66 - 1.65) | 0.70 (0.29 - 1.70) | 0.92 (0.41 - 2.06) | 1.00 (0.64 - 1.58) | 1.18 (0.71 - 1.98) | 0.82 (0.37 - 1.82) | 1.13 (0.69 - 1.83) | Huai'er Granules+OX | . | . | . | . | . | . | . | . | . | . | . | . | . | . | . | . | 0.62 (0.41 - 0.94) |
| 0.70 (0.47 - 1.03) | 0.47 (0.20 - 1.10) | 0.61 (0.28 - 1.33) | 0.67 (0.45 - 0.99) | 0.79 (0.50 - 1.25) | 0.54 (0.25 - 1.17) | 0.75 (0.49 - 1.15) | 0.67 (0.39 - 1.14) | Jinlong Capsules+OX | . | . | . | . | . | . | . | . | . | . | . | . | . | . | . | 0.94 (0.67 - 1.32) |
| 1.25 (0.89 - 1.76) | 0.84 (0.36 - 1.93) | 1.10 (0.52 - 2.33) | 1.20 (0.86 - 1.68) | 1.42 (0.93 - 2.14) | 0.98 (0.47 - 2.05) | 1.35 (0.92 - 1.98) | 1.20 (0.73 - 1.97) | 1.79 (1.15 - 2.79) | Kangai Injection+OX | . | . | . | . | . | . | . | . | . | . | . | . | . | . | 0.52 (0.39 - 0.69) |
| 0.79 (0.52 - 1.20) | 0.53 (0.22 - 1.26) | 0.70 (0.32 - 1.53) | 0.76 (0.51 - 1.14) | 0.90 (0.56 - 1.44) | 0.62 (0.29 - 1.35) | 0.85 (0.54 - 1.34) | 0.76 (0.44 - 1.31) | 1.14 (0.69 - 1.87) | 0.63 (0.40 - 1.00) | Kanglaite Injection+OX | . | . | . | . | . | . | . | . | . | . | . | . | . | 0.82 (0.57 - 1.18) |
| 0.78 (0.45 - 1.37) | 0.52 (0.20 - 1.35) | 0.69 (0.29 - 1.64) | 0.75 (0.43 - 1.30) | 0.89 (0.49 - 1.62) | 0.61 (0.26 - 1.45) | 0.84 (0.47 - 1.51) | 0.75 (0.39 - 1.45) | 1.12 (0.61 - 2.09) | 0.63 (0.35 - 1.13) | 0.99 (0.53 - 1.86) | Kanglixin Capsules+OX | . | . | . | . | . | . | . | . | . | . | . | . | 0.83 (0.50 - 1.40) |
| 1.36 (0.77 - 2.41) | 0.91 (0.35 - 2.36) | 1.19 (0.50 - 2.88) | 1.31 (0.74 - 2.30) | 1.54 (0.83 - 2.85) | 1.06 (0.45 - 2.53) | 1.46 (0.81 - 2.65) | 1.30 (0.66 - 2.55) | 1.95 (1.03 - 3.68) | 1.09 (0.59 - 1.99) | 1.71 (0.90 - 3.27) | 1.73 (0.82 - 3.65) | Lentinan+OX | . | . | . | . | . | . | . | . | . | . | . | 0.48 (0.28 - 0.82) |
| 0.87 (0.41 - 1.84) | 0.58 (0.20 - 1.69) | 0.77 (0.28 - 2.08) | 0.84 (0.40 - 1.76) | 0.99 (0.45 - 2.16) | 0.68 (0.25 - 1.84) | 0.94 (0.44 - 2.02) | 0.83 (0.36 - 1.91) | 1.25 (0.56 - 2.77) | 0.70 (0.32 - 1.51) | 1.10 (0.49 - 2.46) | 1.11 (0.46 - 2.69) | 0.64 (0.26 - 1.57) | Qizhen Capsule+OX | . | . | . | . | . | . | . | . | . | . | 0.75 (0.37 - 1.54) |
| 0.67 (0.33 - 1.39) | 0.45 (0.16 - 1.29) | 0.59 (0.22 - 1.59) | 0.65 (0.32 - 1.33) | 0.76 (0.36 - 1.63) | 0.53 (0.20 - 1.40) | 0.73 (0.34 - 1.53) | 0.64 (0.29 - 1.45) | 0.97 (0.45 - 2.10) | 0.54 (0.25 - 1.14) | 0.85 (0.39 - 1.86) | 0.86 (0.36 - 2.05) | 0.50 (0.21 - 1.19) | 0.77 (0.28 - 2.11) | Shenfu Injection+OX | . | . | . | . | . | . | . | . | . | 0.97 (0.48 - 1.94) |
| 2.61 (0.28 - 24.15) | 1.75 (0.17 - 18.31) | 2.30 (0.23 - 23.40) | 2.51 (0.27 - 23.15) | 2.96 (0.32 - 27.65) | 2.04 (0.20 - 20.74) | 2.81 (0.30 - 26.15) | 2.50 (0.26 - 23.74) | 3.75 (0.40 - 35.22) | 2.09 (0.22 - 19.47) | 3.30 (0.35 - 31.08) | 3.33 (0.34 - 32.38) | 1.92 (0.20 - 18.75) | 3.00 (0.29 - 30.78) | 3.88 (0.38 - 39.47) | Shengxue Granules+OX | . | . | . | . | . | . | . | . | 0.25 (0.03 - 2.29) |
| 0.98 (0.16 - 5.91) | 0.65 (0.09 - 4.61) | 0.86 (0.13 - 5.85) | 0.94 (0.16 - 5.66) | 1.11 (0.18 - 6.78) | 0.77 (0.11 - 5.18) | 1.05 (0.17 - 6.41) | 0.94 (0.15 - 5.85) | 1.41 (0.23 - 8.65) | 0.78 (0.13 - 4.77) | 1.24 (0.20 - 7.64) | 1.25 (0.19 - 8.01) | 0.72 (0.11 - 4.64) | 1.12 (0.16 - 7.71) | 1.45 (0.21 - 9.87) | 0.38 (0.02 - 6.44) | Shenlian Capsule+OX | . | . | . | . | . | . | . | 0.67 (0.11 - 3.97) |
| 1.13 (0.58 - 2.21) | 0.76 (0.27 - 2.09) | 1.00 (0.39 - 2.56) | 1.09 (0.56 - 2.12) | 1.28 (0.63 - 2.61) | 0.89 (0.35 - 2.26) | 1.22 (0.61 - 2.43) | 1.08 (0.51 - 2.32) | 1.63 (0.79 - 3.35) | 0.91 (0.45 - 1.82) | 1.43 (0.69 - 2.98) | 1.45 (0.64 - 3.29) | 0.83 (0.36 - 1.92) | 1.30 (0.50 - 3.41) | 1.68 (0.65 - 4.32) | 0.43 (0.04 - 4.35) | 1.16 (0.17 - 7.70) | Shenmai Injection+OX | . | . | . | . | . | . | 0.58 (0.30 - 1.09) |
| 1.19 (0.90 - 1.57) | 0.79 (0.35 - 1.79) | 1.04 (0.51 - 2.15) | 1.14 (0.87 - 1.49) | 1.34 (0.94 - 1.93) | 0.93 (0.46 - 1.89) | 1.28 (0.92 - 1.77) | 1.14 (0.72 - 1.79) | 1.70 (1.15 - 2.52) | 0.95 (0.68 - 1.34) | 1.50 (0.99 - 2.26) | 1.51 (0.87 - 2.63) | 0.87 (0.49 - 1.54) | 1.36 (0.65 - 2.87) | 1.76 (0.86 - 3.63) | 0.45 (0.05 - 4.20) | 1.21 (0.20 - 7.30) | 1.05 (0.54 - 2.04) | Shenqi Fuzheng Injection+OX | . | . | . | . | . | 0.55 (0.45 - 0.67) |
| 0.75 (0.53 - 1.04) | 0.50 (0.22 - 1.14) | 0.65 (0.31 - 1.38) | 0.71 (0.52 - 0.99) | 0.84 (0.56 - 1.27) | 0.58 (0.28 - 1.22) | 0.80 (0.55 - 1.17) | 0.71 (0.44 - 1.16) | 1.07 (0.69 - 1.65) | 0.60 (0.40 - 0.88) | 0.94 (0.60 - 1.48) | 0.95 (0.53 - 1.70) | 0.55 (0.30 - 1.00) | 0.85 (0.40 - 1.84) | 1.10 (0.52 - 2.33) | 0.28 (0.03 - 2.65) | 0.76 (0.12 - 4.62) | 0.66 (0.33 - 1.31) | 0.63 (0.45 - 0.87) | Xiaoaiping Injection+OX | . | . | . | . | 0.88 (0.67 - 1.15) |
| 1.11 (0.52 - 2.33) | 0.74 (0.25 - 2.15) | 0.97 (0.36 - 2.65) | 1.06 (0.50 - 2.23) | 1.25 (0.57 - 2.74) | 0.86 (0.32 - 2.34) | 1.19 (0.55 - 2.56) | 1.06 (0.46 - 2.42) | 1.59 (0.71 - 3.52) | 0.88 (0.41 - 1.92) | 1.39 (0.62 - 3.12) | 1.41 (0.58 - 3.42) | 0.81 (0.33 - 1.99) | 1.27 (0.46 - 3.51) | 1.64 (0.60 - 4.46) | 0.42 (0.04 - 4.34) | 1.13 (0.16 - 7.73) | 0.98 (0.37 - 2.55) | 0.93 (0.44 - 1.96) | 1.48 (0.69 - 3.20) | Xihuang Capsules+OX | . | . | . | 0.59 (0.29 - 1.21) |
| 0.93 (0.64 - 1.36) | 0.62 (0.27 - 1.46) | 0.82 (0.38 - 1.77) | 0.90 (0.62 - 1.30) | 1.06 (0.68 - 1.65) | 0.73 (0.34 - 1.56) | 1.00 (0.66 - 1.52) | 0.89 (0.53 - 1.50) | 1.34 (0.84 - 2.14) | 0.75 (0.49 - 1.15) | 1.18 (0.72 - 1.91) | 1.19 (0.65 - 2.19) | 0.69 (0.37 - 1.28) | 1.07 (0.49 - 2.36) | 1.38 (0.64 - 2.98) | 0.36 (0.04 - 3.35) | 0.95 (0.16 - 5.84) | 0.82 (0.40 - 1.68) | 0.79 (0.54 - 1.15) | 1.25 (0.82 - 1.91) | 0.84 (0.38 - 1.86) | Ya Dan Zi Oil Emulsion Injection+OX | . | . | 0.70 (0.51 - 0.97) |
| 1.08 (0.65 - 1.80) | 0.72 (0.29 - 1.80) | 0.95 (0.41 - 2.20) | 1.03 (0.62 - 1.72) | 1.22 (0.69 - 2.14) | 0.84 (0.37 - 1.94) | 1.16 (0.67 - 1.99) | 1.03 (0.55 - 1.92) | 1.54 (0.86 - 2.77) | 0.86 (0.50 - 1.49) | 1.36 (0.75 - 2.47) | 1.37 (0.68 - 2.77) | 0.79 (0.39 - 1.62) | 1.24 (0.52 - 2.92) | 1.60 (0.69 - 3.70) | 0.41 (0.04 - 3.97) | 1.10 (0.17 - 6.96) | 0.95 (0.43 - 2.10) | 0.91 (0.54 - 1.51) | 1.45 (0.84 - 2.49) | 0.97 (0.41 - 2.30) | 1.15 (0.65 - 2.05) | Yangzheng Xiaoji Capsules+OX | . | 0.61 (0.38 - 0.97) |
| 1.72 (0.71 - 4.14) | 1.15 (0.36 - 3.68) | 1.51 (0.50 - 4.55) | 1.65 (0.68 - 3.96) | 1.94 (0.78 - 4.83) | 1.34 (0.45 - 4.02) | 1.85 (0.75 - 4.53) | 1.64 (0.63 - 4.24) | 2.46 (0.98 - 6.19) | 1.37 (0.56 - 3.38) | 2.16 (0.85 - 5.49) | 2.19 (0.80 - 5.96) | 1.26 (0.46 - 3.47) | 1.97 (0.64 - 6.03) | 2.54 (0.84 - 7.68) | 0.66 (0.06 - 7.05) | 1.75 (0.24 - 12.68) | 1.51 (0.52 - 4.41) | 1.44 (0.60 - 3.48) | 2.30 (0.94 - 5.66) | 1.55 (0.51 - 4.76) | 1.84 (0.73 - 4.60) | 1.59 (0.60 - 4.24) | Zhenqi Fuzheng Granules+OX | 0.38 (0.16 - 0.90) |
| 0.65 (0.54 - 0.80) | 0.44 (0.20 - 0.96) | 0.57 (0.29 - 1.15) | 0.63 (0.52 - 0.75) | 0.74 (0.54 - 1.00) | 0.51 (0.26 - 1.01) | 0.70 (0.54 - 0.91) | 0.62 (0.41 - 0.94) | 0.94 (0.67 - 1.32) | 0.52 (0.39 - 0.69) | 0.82 (0.57 - 1.18) | 0.83 (0.50 - 1.40) | 0.48 (0.28 - 0.82) | 0.75 (0.37 - 1.54) | 0.97 (0.48 - 1.94) | 0.25 (0.03 - 2.29) | 0.67 (0.11 - 3.97) | 0.58 (0.30 - 1.09) | 0.55 (0.45 - 0.67) | 0.88 (0.67 - 1.15) | 0.59 (0.29 - 1.21) | 0.70 (0.51 - 0.97) | 0.61 (0.38 - 0.97) | 0.38 (0.16 - 0.90) | OX |

**Q. Gastrointestinal event**

| Aidi Injection+OX | . | . | . | . | . | . | . | . | . | . | . | . | . | . | . | . | . | . | . | . | . | . | . | 0.56 (0.45 - 0.68) |
| --- | --- | --- | --- | --- | --- | --- | --- | --- | --- | --- | --- | --- | --- | --- | --- | --- | --- | --- | --- | --- | --- | --- | --- | --- |
| 1.32 (0.62 - 2.80) | Astragalus Polysaccharides+OX | . | . | . | . | . | . | . | . | . | . | . | . | . | . | . | . | . | . | . | . | . | . | 0.42 (0.20 - 0.87) |
| 1.02 (0.51 - 2.03) | 0.77 (0.29 - 2.05) | Astragalus preparations+OX | . | . | . | . | . | . | . | . | . | . | . | . | . | . | . | . | . | . | . | . | . | 0.55 (0.28 - 1.06) |
| 0.75 (0.57 - 0.98) | 0.57 (0.27 - 1.19) | 0.73 (0.37 - 1.45) | Compound Kushen Injection+OX | . | . | . | . | . | . | . | . | . | . | . | . | . | . | . | . | . | . | . | . | 0.74 (0.63 - 0.88) |
| 0.71 (0.52 - 0.97) | 0.54 (0.25 - 1.15) | 0.70 (0.35 - 1.41) | 0.95 (0.71 - 1.27) | Compound Mylabris preparations+OX | . | . | . | . | . | . | . | . | . | . | . | . | . | . | . | . | . | . | . | 0.78 (0.62 - 0.99) |
| 1.33 (0.48 - 3.62) | 1.00 (0.30 - 3.40) | 1.30 (0.40 - 4.25) | 1.77 (0.65 - 4.80) | 1.86 (0.68 - 5.12) | Diyu Shengbai Tablets+OX | . | . | . | . | . | . | . | . | . | . | . | . | . | . | . | . | . | . | 0.42 (0.16 - 1.12) |
| 0.14 (0.05 - 0.38) | 0.11 (0.03 - 0.36) | 0.14 (0.04 - 0.44) | 0.19 (0.07 - 0.50) | 0.20 (0.07 - 0.53) | 0.11 (0.03 - 0.42) | Ginseng Polysaccharide Injection+OX | . | . | . | . | . | . | . | . | . | . | . | . | . | . | . | . | . | 3.96 (1.51 - 10.40) |
| 0.74 (0.55 - 1.00) | 0.56 (0.26 - 1.19) | 0.73 (0.36 - 1.46) | 0.99 (0.75 - 1.31) | 1.04 (0.75 - 1.44) | 0.56 (0.20 - 1.53) | 5.27 (1.96 - 14.19) | Huachansu preparations+OX | . | . | . | . | . | . | . | . | . | . | . | . | . | . | . | . | 0.75 (0.60 - 0.94) |
| 0.65 (0.41 - 1.03) | 0.49 (0.21 - 1.13) | 0.64 (0.29 - 1.38) | 0.87 (0.56 - 1.35) | 0.91 (0.57 - 1.46) | 0.49 (0.17 - 1.42) | 4.62 (1.62 - 13.18) | 0.88 (0.55 - 1.39) | Huai'er Granules+OX | . | . | . | . | . | . | . | . | . | . | . | . | . | . | . | 0.86 (0.57 - 1.29) |
| 0.73 (0.46 - 1.16) | 0.55 (0.24 - 1.27) | 0.71 (0.33 - 1.56) | 0.97 (0.62 - 1.53) | 1.02 (0.63 - 1.65) | 0.55 (0.19 - 1.60) | 5.19 (1.81 - 14.85) | 0.98 (0.61 - 1.58) | 1.12 (0.63 - 2.01) | Jinlong Capsules+OX | . | . | . | . | . | . | . | . | . | . | . | . | . | . | 0.76 (0.50 - 1.16) |
| 0.85 (0.65 - 1.13) | 0.65 (0.31 - 1.37) | 0.84 (0.42 - 1.66) | 1.14 (0.88 - 1.47) | 1.20 (0.89 - 1.62) | 0.65 (0.24 - 1.76) | 6.08 (2.27 - 16.26) | 1.15 (0.86 - 1.54) | 1.32 (0.84 - 2.06) | 1.17 (0.74 - 1.86) | Kangai Injection+OX | . | . | . | . | . | . | . | . | . | . | . | . | . | 0.65 (0.54 - 0.79) |
| 0.68 (0.49 - 0.95) | 0.52 (0.24 - 1.11) | 0.67 (0.33 - 1.36) | 0.91 (0.66 - 1.25) | 0.96 (0.67 - 1.36) | 0.51 (0.19 - 1.43) | 4.85 (1.78 - 13.20) | 0.92 (0.65 - 1.30) | 1.05 (0.64 - 1.71) | 0.94 (0.57 - 1.53) | 0.80 (0.57 - 1.11) | Kanglaite Injection+OX | . | . | . | . | . | . | . | . | . | . | . | . | 0.82 (0.63 - 1.07) |
| 0.59 (0.31 - 1.13) | 0.45 (0.17 - 1.15) | 0.58 (0.23 - 1.42) | 0.79 (0.41 - 1.49) | 0.83 (0.43 - 1.60) | 0.44 (0.14 - 1.42) | 4.18 (1.33 - 13.14) | 0.79 (0.41 - 1.53) | 0.91 (0.43 - 1.90) | 0.81 (0.38 - 1.70) | 0.69 (0.36 - 1.31) | 0.86 (0.44 - 1.69) | Kanglixin Capsules+OX | . | . | . | . | . | . | . | . | . | . | . | 0.95 (0.51 - 1.75) |
| 0.89 (0.58 - 1.36) | 0.67 (0.30 - 1.52) | 0.87 (0.41 - 1.86) | 1.18 (0.78 - 1.79) | 1.24 (0.80 - 1.94) | 0.67 (0.23 - 1.92) | 6.30 (2.24 - 17.77) | 1.20 (0.77 - 1.85) | 1.36 (0.78 - 2.38) | 1.22 (0.69 - 2.14) | 1.04 (0.68 - 1.58) | 1.30 (0.82 - 2.06) | 1.51 (0.73 - 3.10) | Lentinan+OX | . | . | . | . | . | . | . | . | . | . | 0.63 (0.43 - 0.92) |
| 0.73 (0.37 - 1.42) | 0.55 (0.21 - 1.44) | 0.71 (0.28 - 1.78) | 0.97 (0.50 - 1.87) | 1.02 (0.52 - 2.01) | 0.55 (0.17 - 1.77) | 5.17 (1.63 - 16.42) | 0.98 (0.50 - 1.92) | 1.12 (0.52 - 2.38) | 1.00 (0.46 - 2.13) | 0.85 (0.44 - 1.65) | 1.07 (0.53 - 2.12) | 1.23 (0.51 - 2.99) | 0.82 (0.39 - 1.72) | Qizhen Capsule+OX | . | . | . | . | . | . | . | . | . | 0.77 (0.41 - 1.45) |
| 1.01 (0.47 - 2.18) | 0.77 (0.27 - 2.16) | 0.99 (0.37 - 2.68) | 1.35 (0.63 - 2.89) | 1.42 (0.65 - 3.09) | 0.76 (0.22 - 2.62) | 7.20 (2.13 - 24.31) | 1.37 (0.63 - 2.96) | 1.56 (0.67 - 3.63) | 1.39 (0.59 - 3.25) | 1.18 (0.55 - 2.55) | 1.49 (0.68 - 3.26) | 1.72 (0.66 - 4.51) | 1.14 (0.50 - 2.62) | 1.39 (0.53 - 3.70) | Shenfu Injection+OX | . | . | . | . | . | . | . | . | 0.55 (0.26 - 1.15) |
| 1.39 (0.26 - 7.41) | 1.05 (0.17 - 6.44) | 1.36 (0.23 - 8.14) | 1.86 (0.35 - 9.86) | 1.95 (0.37 - 10.44) | 1.05 (0.15 - 7.24) | 9.90 (1.45 - 67.55) | 1.88 (0.35 - 10.02) | 2.14 (0.39 - 11.84) | 1.91 (0.34 - 10.57) | 1.63 (0.31 - 8.66) | 2.04 (0.38 - 10.97) | 2.37 (0.40 - 13.90) | 1.57 (0.29 - 8.62) | 1.92 (0.32 - 11.34) | 1.37 (0.22 - 8.46) | Shengxue Granules+OX | . | . | . | . | . | . | . | 0.40 (0.08 - 2.10) |
| 0.84 (0.14 - 4.99) | 0.63 (0.09 - 4.30) | 0.82 (0.12 - 5.44) | 1.11 (0.19 - 6.63) | 1.17 (0.20 - 7.02) | 0.63 (0.08 - 4.80) | 5.94 (0.79 - 44.81) | 1.13 (0.19 - 6.74) | 1.29 (0.21 - 7.95) | 1.15 (0.18 - 7.10) | 0.98 (0.16 - 5.83) | 1.22 (0.20 - 7.37) | 1.42 (0.22 - 9.30) | 0.94 (0.15 - 5.79) | 1.15 (0.17 - 7.58) | 0.82 (0.12 - 5.64) | 0.60 (0.05 - 6.82) | Shenlian Capsule+OX | . | . | . | . | . | . | 0.67 (0.11 - 3.93) |
| 0.75 (0.46 - 1.20) | 0.56 (0.24 - 1.31) | 0.73 (0.33 - 1.61) | 1.00 (0.63 - 1.58) | 1.05 (0.64 - 1.71) | 0.56 (0.19 - 1.65) | 5.30 (1.84 - 15.26) | 1.01 (0.62 - 1.63) | 1.15 (0.63 - 2.08) | 1.02 (0.56 - 1.86) | 0.87 (0.54 - 1.40) | 1.09 (0.66 - 1.81) | 1.27 (0.60 - 2.69) | 0.84 (0.47 - 1.49) | 1.03 (0.48 - 2.21) | 0.74 (0.31 - 1.73) | 0.54 (0.10 - 2.98) | 0.89 (0.14 - 5.55) | Shenmai Injection+OX | . | . | . | . | . | 0.75 (0.49 - 1.15) |
| 0.92 (0.70 - 1.22) | 0.70 (0.33 - 1.47) | 0.90 (0.46 - 1.80) | 1.23 (0.96 - 1.59) | 1.30 (0.96 - 1.75) | 0.70 (0.26 - 1.90) | 6.56 (2.46 - 17.55) | 1.25 (0.93 - 1.66) | 1.42 (0.91 - 2.23) | 1.27 (0.80 - 2.00) | 1.08 (0.83 - 1.41) | 1.35 (0.98 - 1.88) | 1.57 (0.82 - 2.99) | 1.04 (0.68 - 1.59) | 1.27 (0.65 - 2.47) | 0.91 (0.42 - 1.96) | 0.66 (0.12 - 3.53) | 1.11 (0.19 - 6.59) | 1.24 (0.77 - 1.98) | Shenqi Fuzheng Injection+OX | . | . | . | . | 0.60 (0.50 - 0.73) |
| 0.62 (0.45 - 0.85) | 0.47 (0.22 - 1.00) | 0.61 (0.30 - 1.22) | 0.83 (0.62 - 1.11) | 0.87 (0.62 - 1.22) | 0.47 (0.17 - 1.29) | 4.40 (1.63 - 11.90) | 0.83 (0.60 - 1.16) | 0.95 (0.59 - 1.53) | 0.85 (0.52 - 1.37) | 0.72 (0.53 - 0.98) | 0.91 (0.63 - 1.30) | 1.05 (0.54 - 2.04) | 0.70 (0.45 - 1.09) | 0.85 (0.43 - 1.68) | 0.61 (0.28 - 1.33) | 0.44 (0.08 - 2.38) | 0.74 (0.12 - 4.44) | 0.83 (0.51 - 1.36) | 0.67 (0.49 - 0.91) | Xiaoaiping Injection+OX | . | . | . | 0.90 (0.71 - 1.14) |
| 0.48 (0.21 - 1.07) | 0.36 (0.12 - 1.05) | 0.47 (0.17 - 1.30) | 0.64 (0.29 - 1.42) | 0.67 (0.30 - 1.51) | 0.36 (0.10 - 1.26) | 3.39 (0.98 - 11.74) | 0.64 (0.29 - 1.45) | 0.73 (0.30 - 1.77) | 0.65 (0.27 - 1.59) | 0.56 (0.25 - 1.25) | 0.70 (0.31 - 1.60) | 0.81 (0.30 - 2.19) | 0.54 (0.23 - 1.28) | 0.66 (0.24 - 1.80) | 0.47 (0.16 - 1.38) | 0.34 (0.05 - 2.15) | 0.57 (0.08 - 3.97) | 0.64 (0.26 - 1.56) | 0.52 (0.23 - 1.15) | 0.77 (0.34 - 1.75) | Xihuang Capsules+OX | . | . | 1.17 (0.53 - 2.55) |
| 0.71 (0.48 - 1.06) | 0.54 (0.24 - 1.20) | 0.70 (0.33 - 1.46) | 0.95 (0.65 - 1.39) | 1.00 (0.66 - 1.51) | 0.54 (0.19 - 1.52) | 5.07 (1.82 - 14.08) | 0.96 (0.64 - 1.44) | 1.10 (0.65 - 1.86) | 0.98 (0.57 - 1.67) | 0.83 (0.57 - 1.23) | 1.05 (0.68 - 1.60) | 1.21 (0.60 - 2.44) | 0.80 (0.48 - 1.33) | 0.98 (0.48 - 2.02) | 0.70 (0.31 - 1.59) | 0.51 (0.09 - 2.78) | 0.85 (0.14 - 5.20) | 0.96 (0.55 - 1.65) | 0.77 (0.53 - 1.13) | 1.15 (0.76 - 1.74) | 1.49 (0.64 - 3.49) | Ya Dan Zi Oil Emulsion Injection+OX | . | 0.78 (0.56 - 1.09) |
| 1.08 (0.72 - 1.61) | 0.82 (0.37 - 1.82) | 1.06 (0.50 - 2.23) | 1.44 (0.98 - 2.12) | 1.51 (1.00 - 2.30) | 0.81 (0.29 - 2.31) | 7.68 (2.75 - 21.40) | 1.46 (0.97 - 2.19) | 1.66 (0.97 - 2.84) | 1.48 (0.86 - 2.55) | 1.26 (0.85 - 1.87) | 1.58 (1.02 - 2.45) | 1.84 (0.91 - 3.72) | 1.22 (0.73 - 2.03) | 1.49 (0.72 - 3.07) | 1.07 (0.47 - 2.41) | 0.78 (0.14 - 4.23) | 1.29 (0.21 - 7.89) | 1.45 (0.83 - 2.51) | 1.17 (0.79 - 1.73) | 1.74 (1.15 - 2.66) | 2.26 (0.96 - 5.31) | 1.51 (0.94 - 2.45) | Yangzheng Xiaoji Capsules+OX | 0.52 (0.37 - 0.73) |
| 0.56 (0.45 - 0.68) | 0.42 (0.20 - 0.87) | 0.55 (0.28 - 1.06) | 0.74 (0.63 - 0.88) | 0.78 (0.62 - 0.99) | 0.42 (0.16 - 1.12) | 3.96 (1.51 - 10.40) | 0.75 (0.60 - 0.94) | 0.86 (0.57 - 1.29) | 0.76 (0.50 - 1.16) | 0.65 (0.54 - 0.79) | 0.82 (0.63 - 1.07) | 0.95 (0.51 - 1.75) | 0.63 (0.43 - 0.92) | 0.77 (0.41 - 1.45) | 0.55 (0.26 - 1.15) | 0.40 (0.08 - 2.10) | 0.67 (0.11 - 3.93) | 0.75 (0.49 - 1.15) | 0.60 (0.50 - 0.73) | 0.90 (0.71 - 1.14) | 1.17 (0.53 - 2.55) | 0.78 (0.56 - 1.09) | 0.52 (0.37 - 0.73) | OX |

# **Appendix 11 Results of fixed model**

1. **Disease control rate**


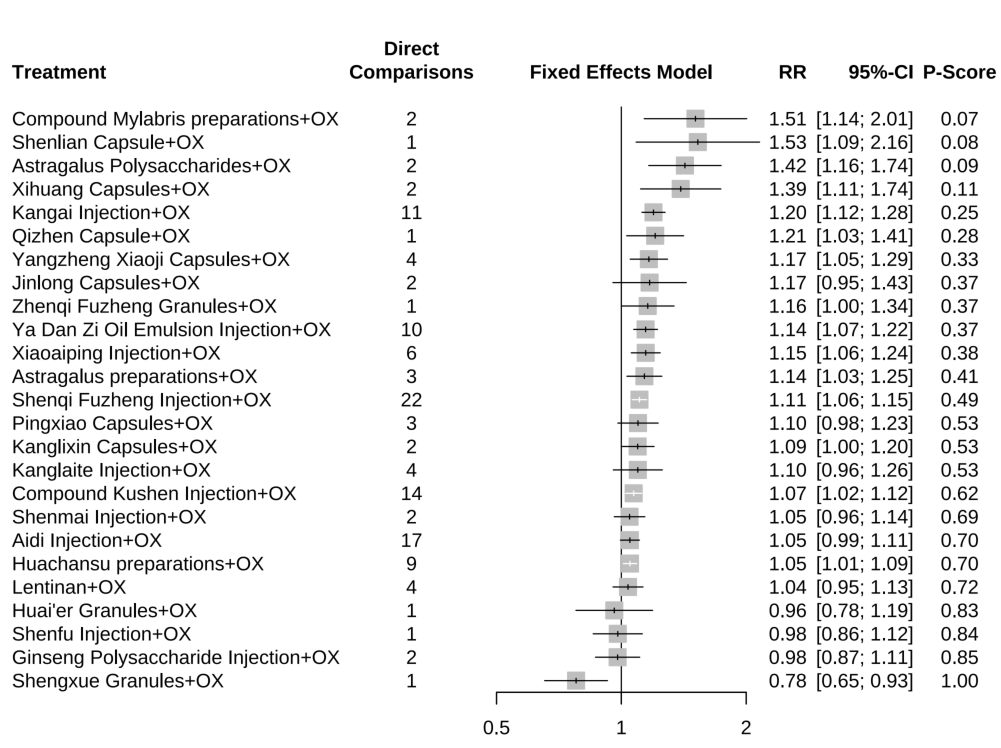


1. **Objective response rate**


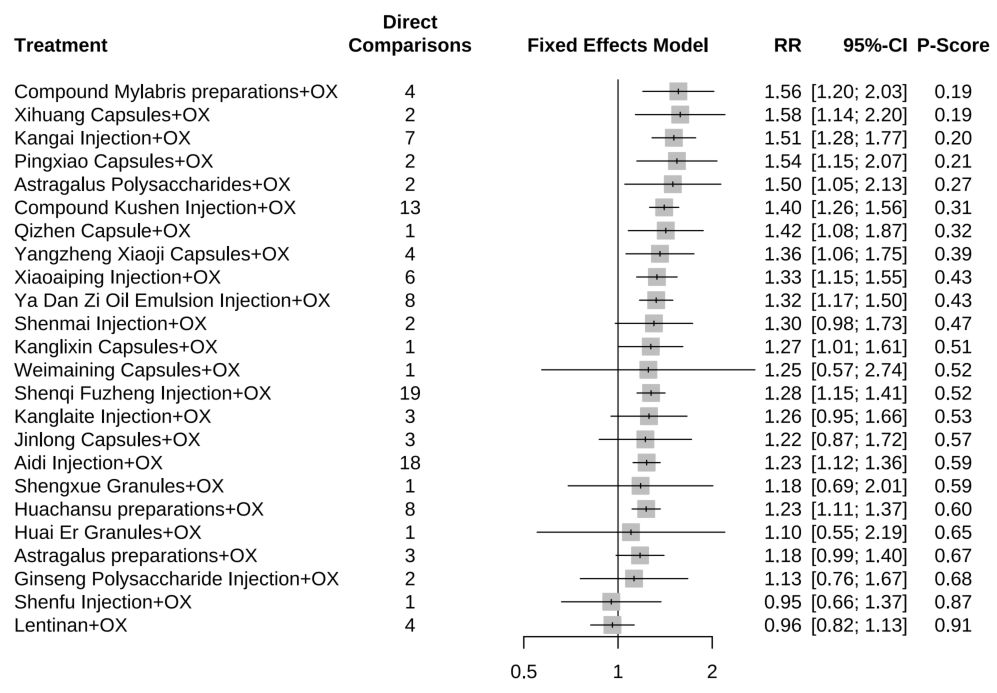


1. **1-year OS**


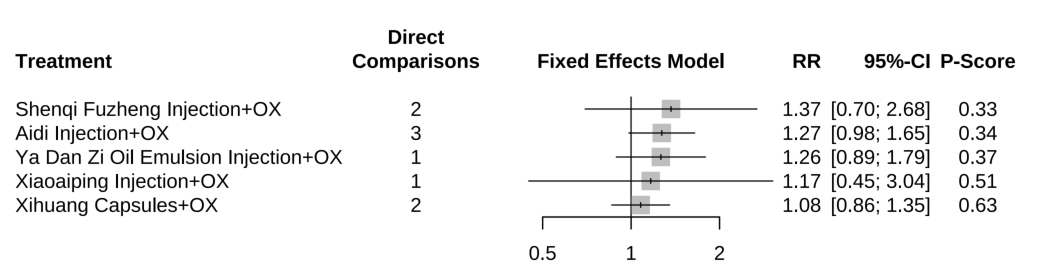


1. **2-year OS**


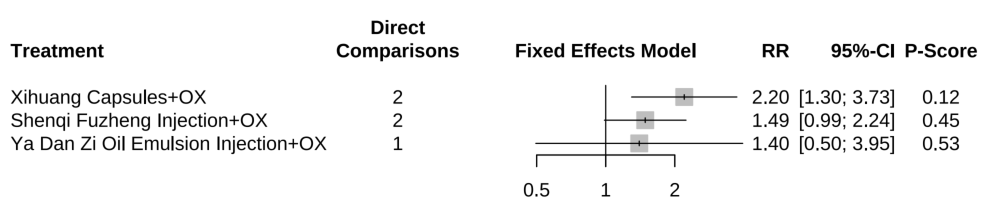


1. **CA125**


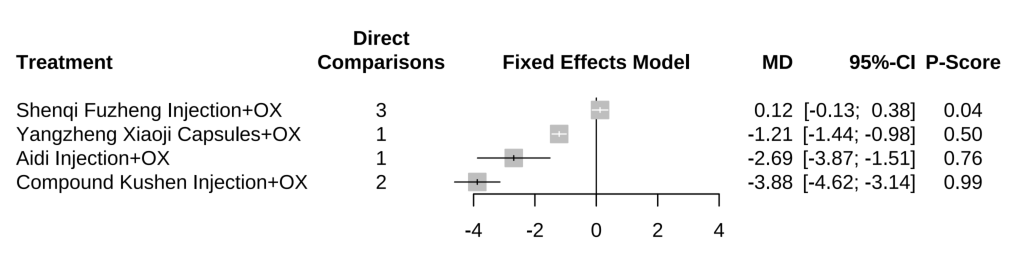


1. **CA199**


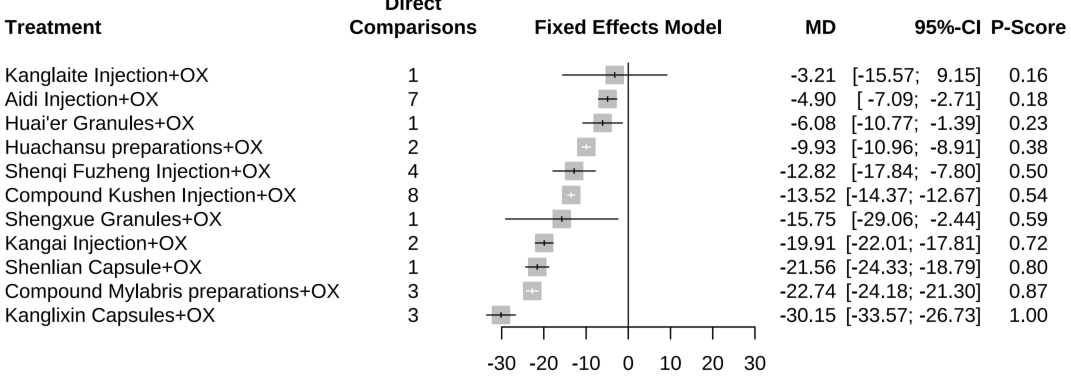


1. **CA724**


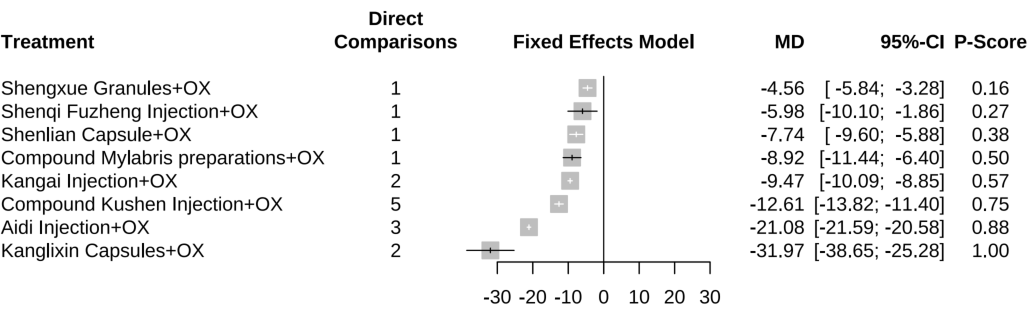


1. **CEA**


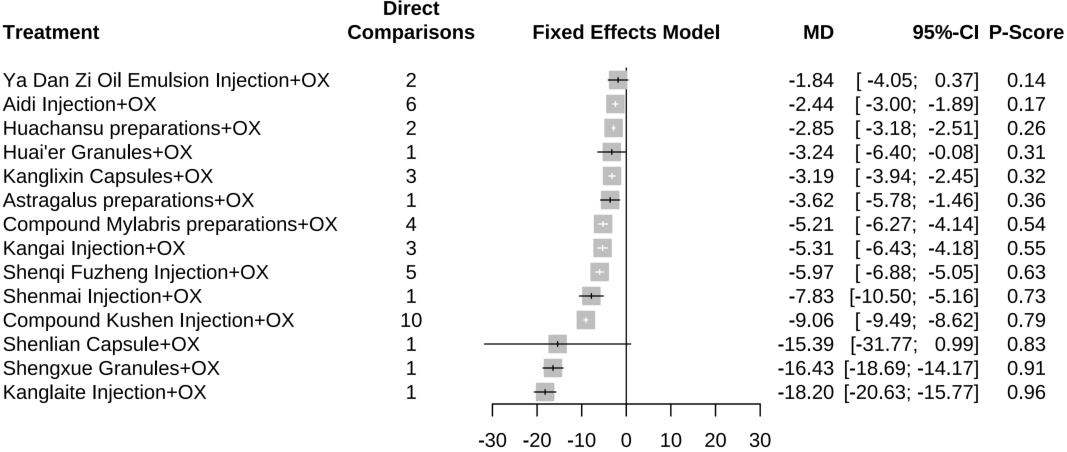


1. **CD3+**


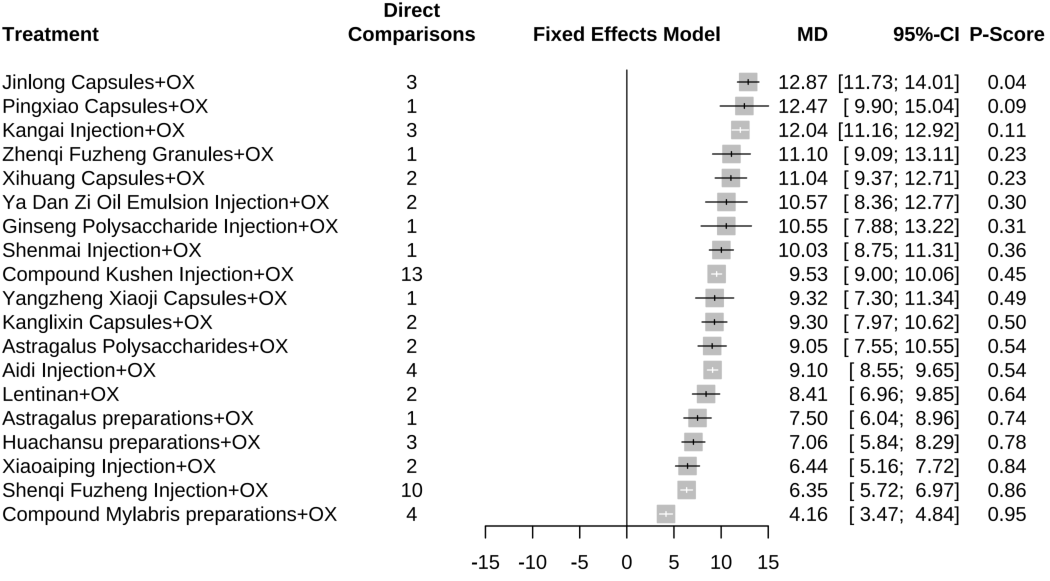


1. **NK cell**


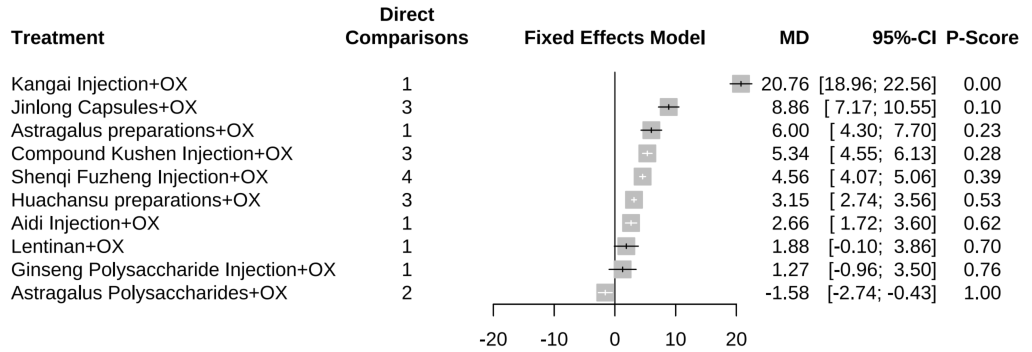


1. **CD4+**


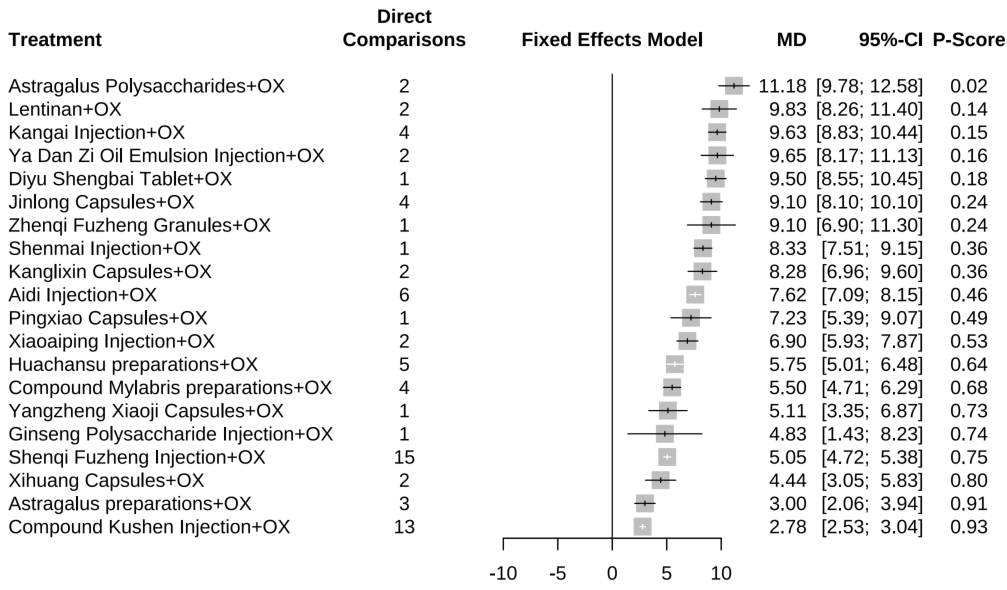


1. **CD4+CD8+**


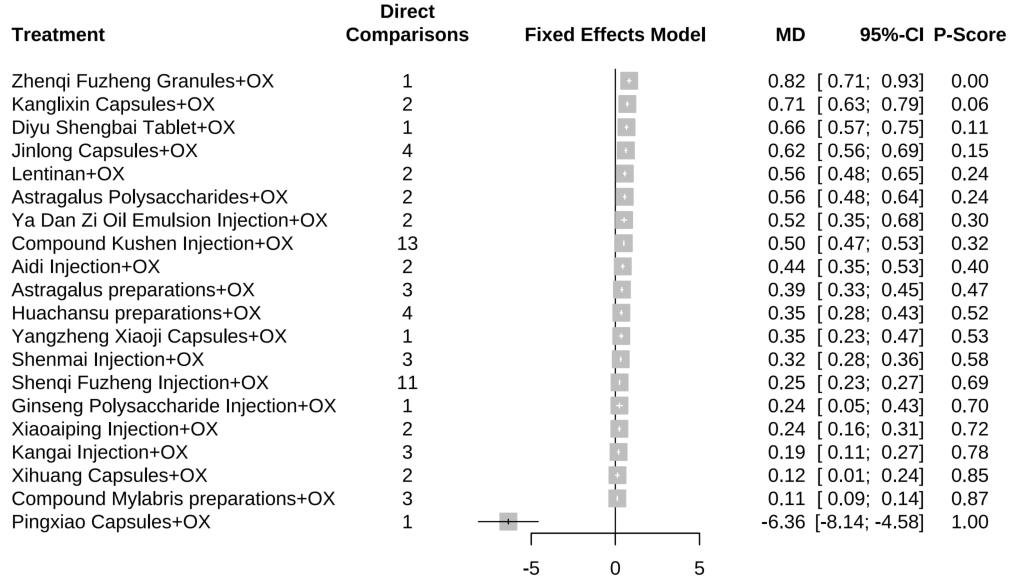


1. **CD8+**


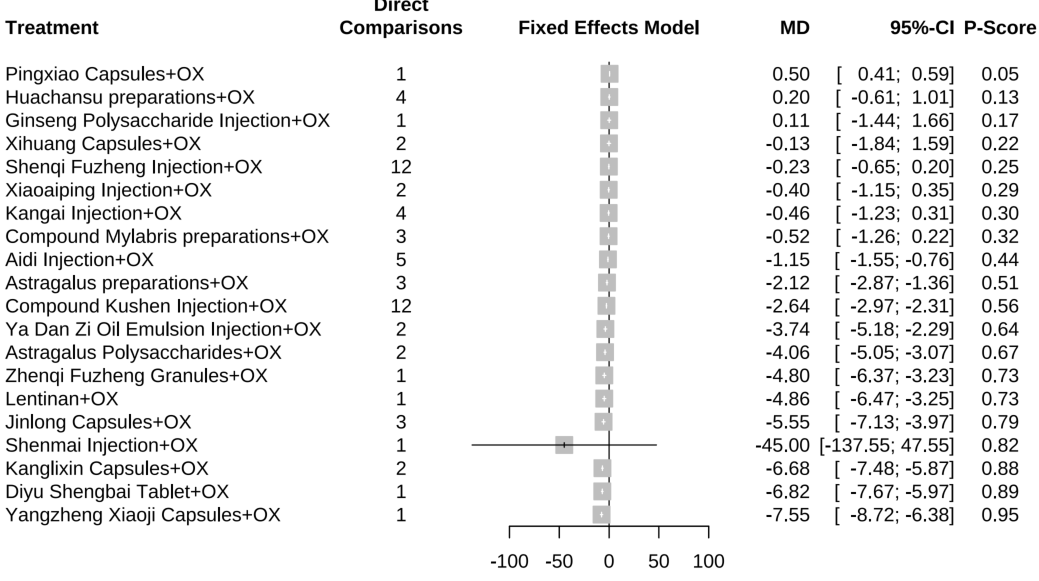


1. **Traditional Chinese medicine (TCM) syndrome score**


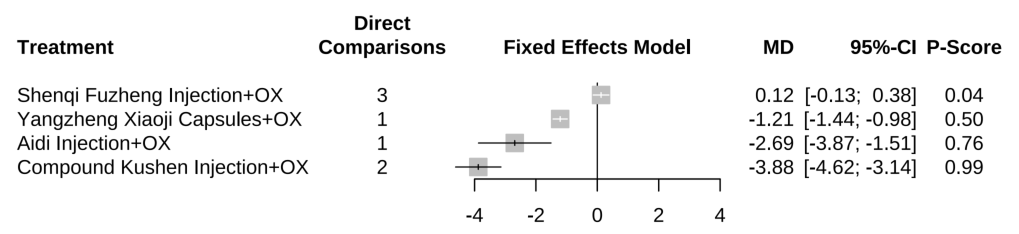


1. **Improvement rate in quality of life**


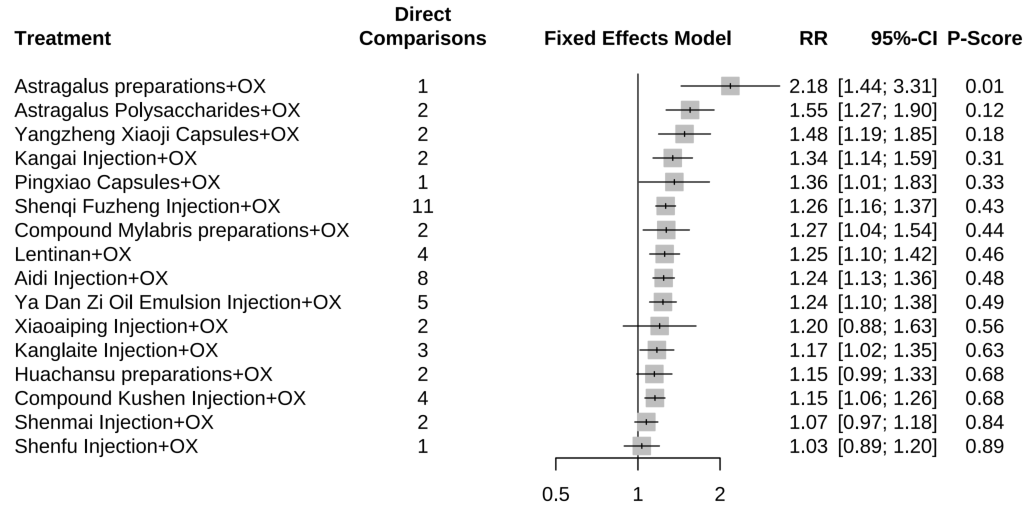


1. **Myelosuppression event**


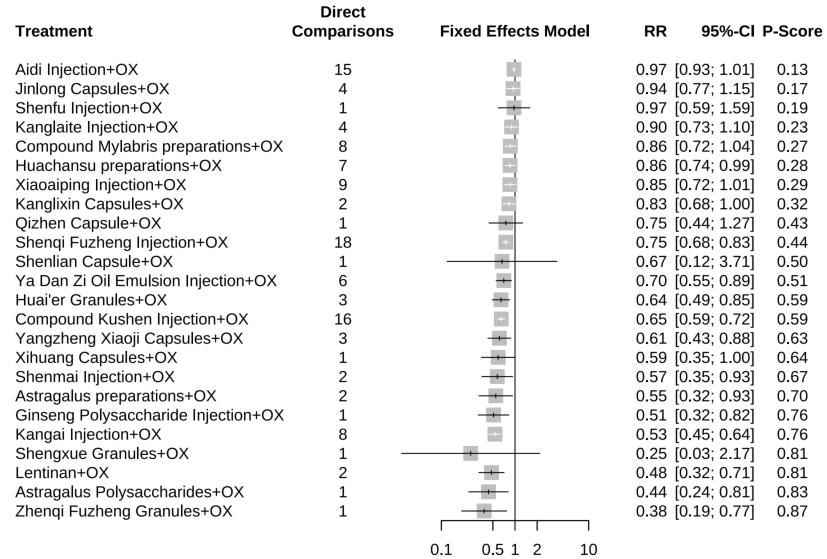


**Q. Gastrointestinal event**


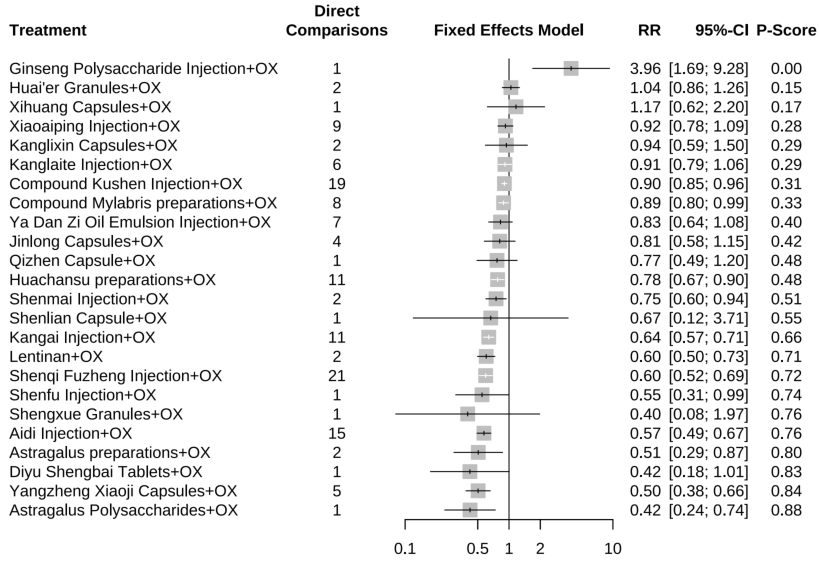


# **Appendix 12 Results of sensitivity analysis by excluding high risk bias**

1. **Disease control rate**

1. **Objective response rate**

1. **QoL**

1. **1-year OS**

1. **2-year OS**

1. **CD3+**

1. **CD4+**

1. **CD8+**

1. **CD4+/CD8+**

1. **CA199**

1. **CA724**

1. **CEA**

1. **Myelosuppression event**

1. **Gastrointestinal event**

# **Appendix 13 Results of publication bias with funnel plot**

1. **Disease control rate**


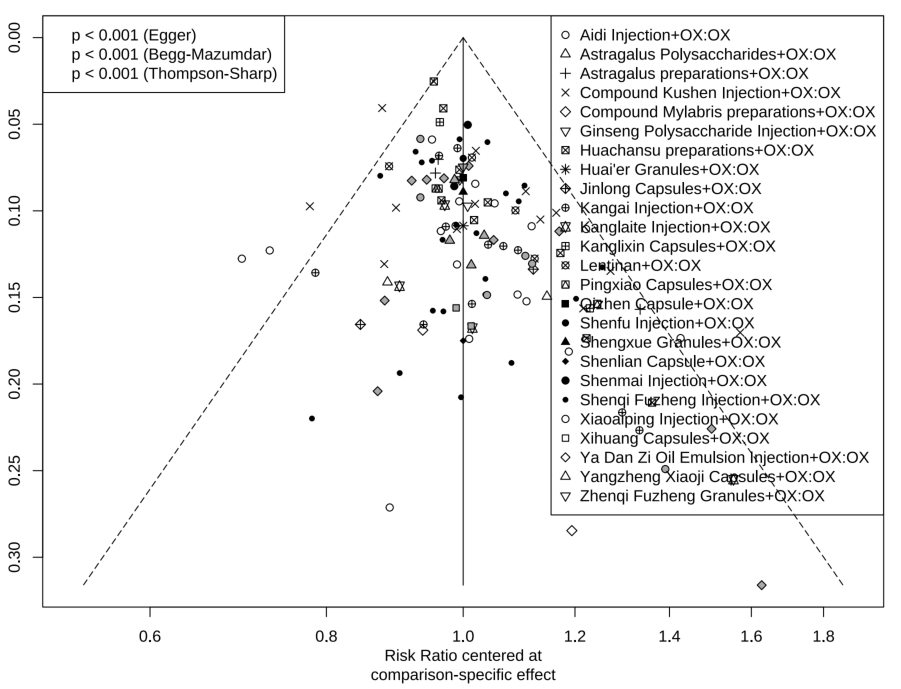


1. **Objective response rate**


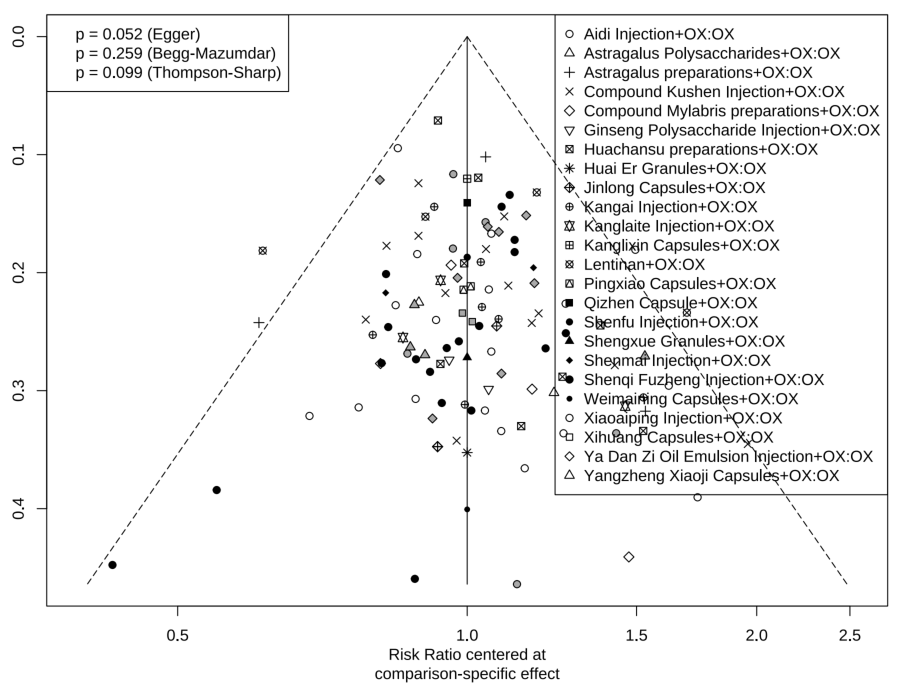


1. **1-year OS**


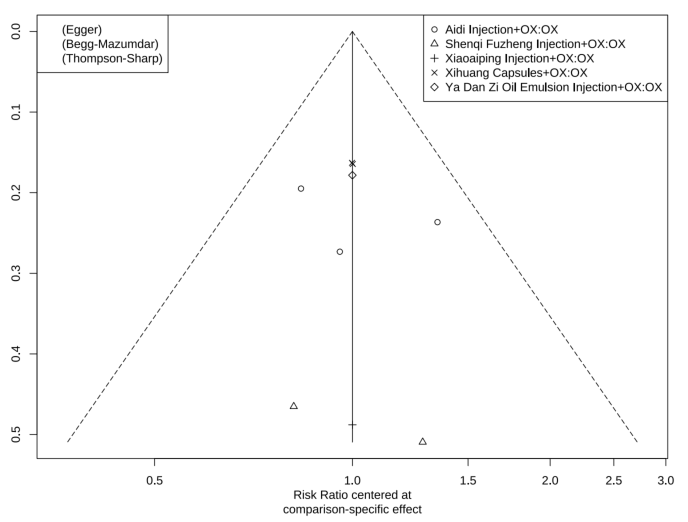


1. **2-year OS**


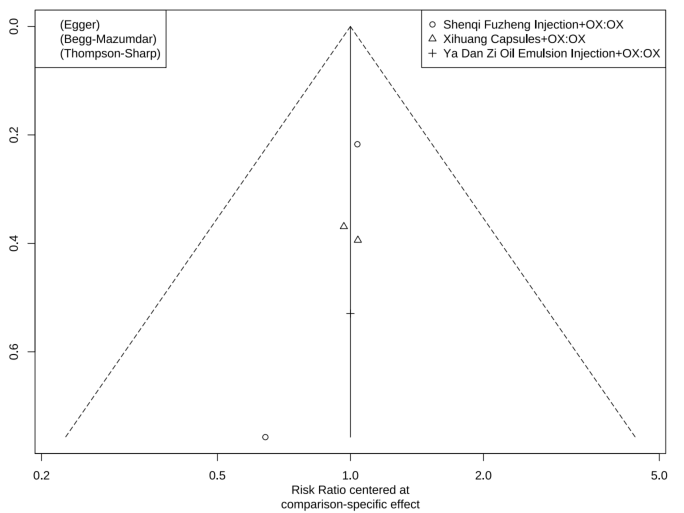


1. **CA125**


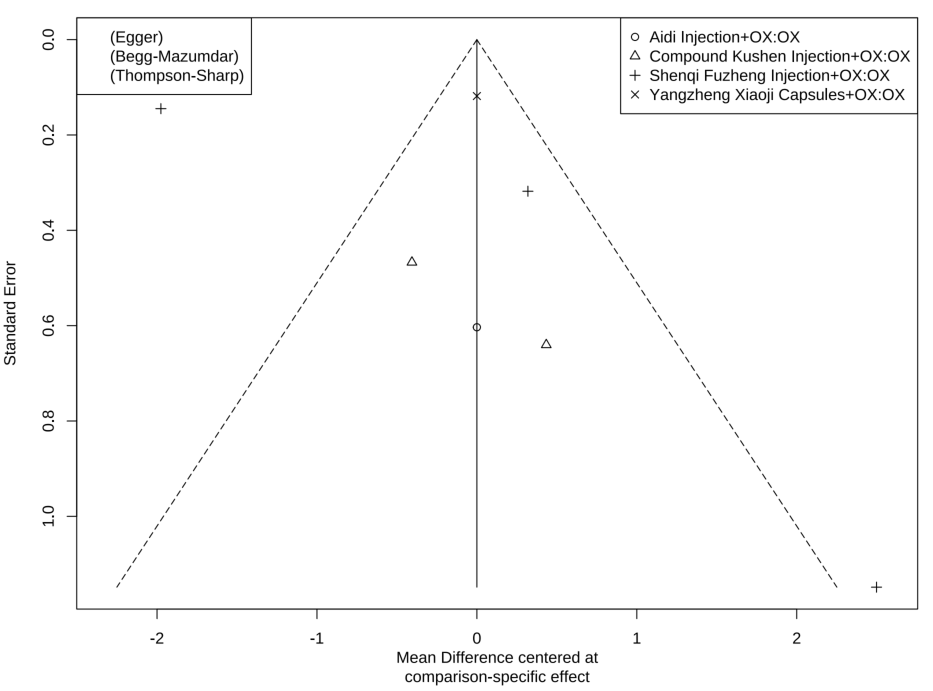


1. **CA199**


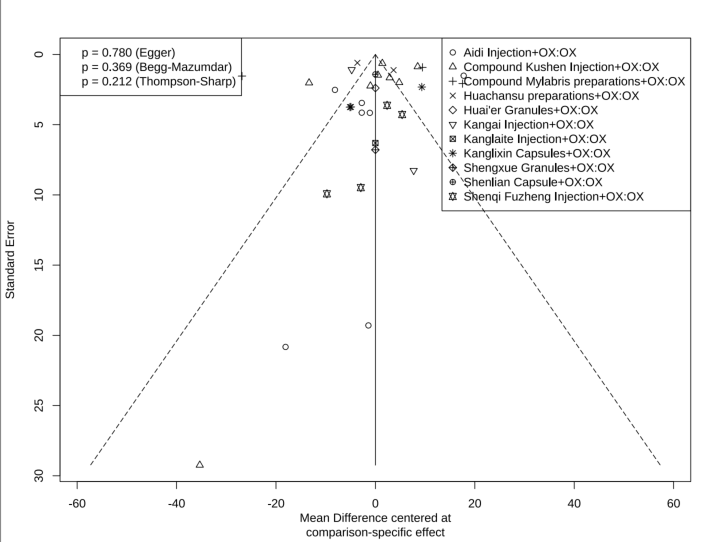


1. **CA724**


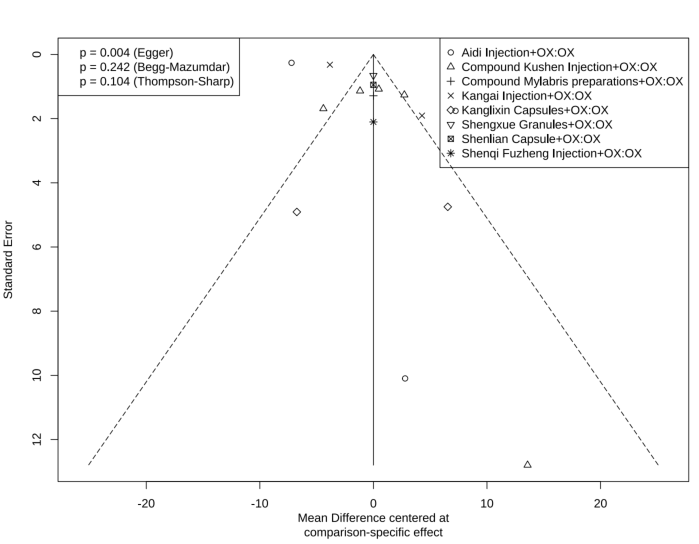


1. **CEA**


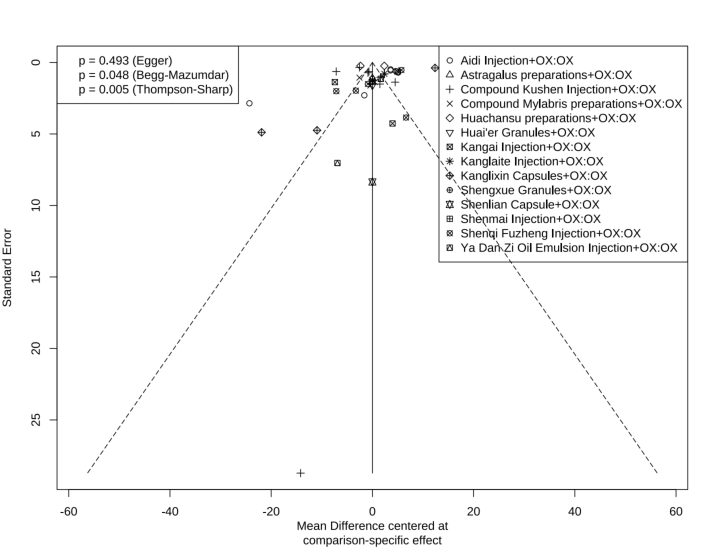


1. **CD3+**


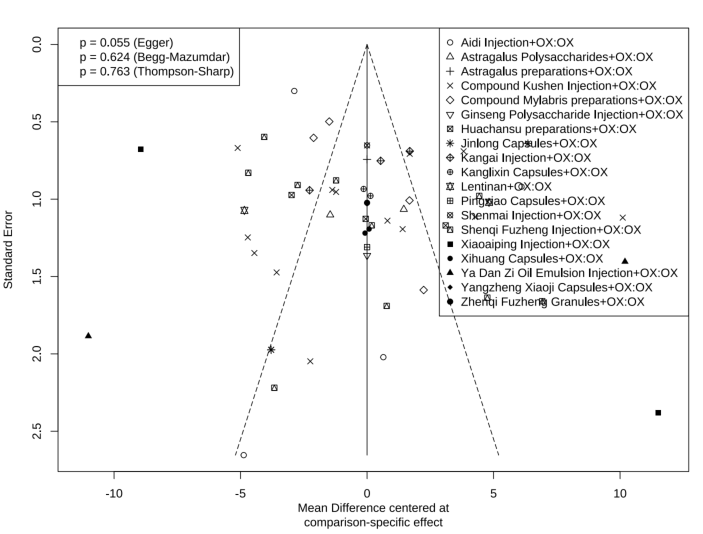


1. **NK cell**


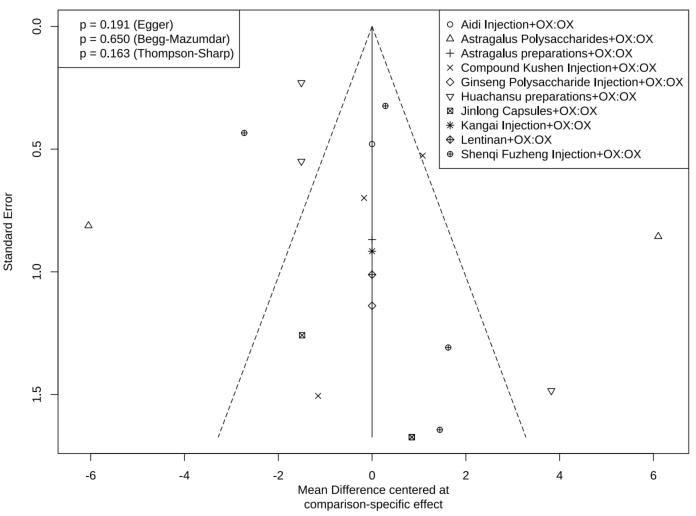


1. **CD4+**


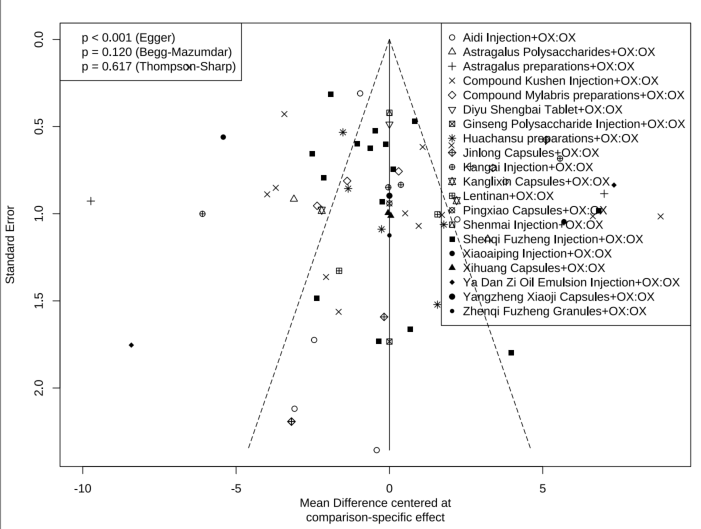


1. **CD4+/CD8+**


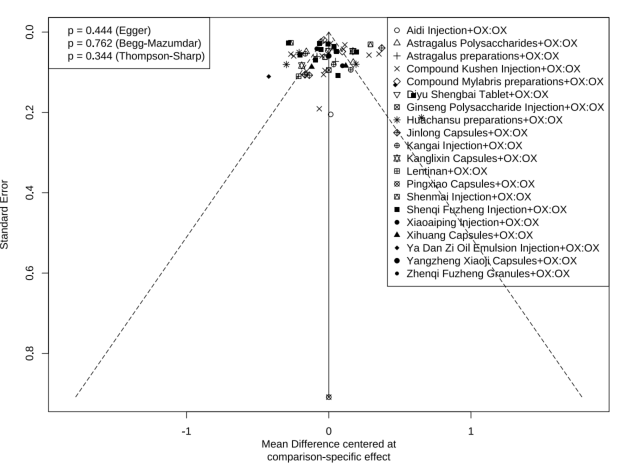


1. **CD8+**


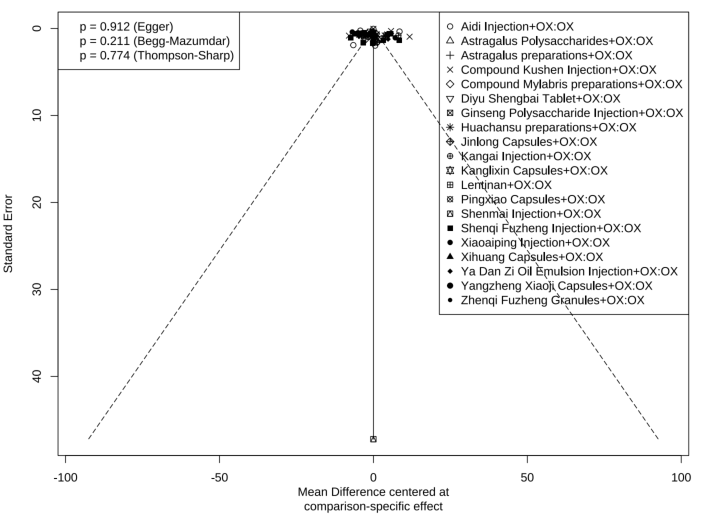


1. **Traditional Chinese medicine (TCM) syndrome score**


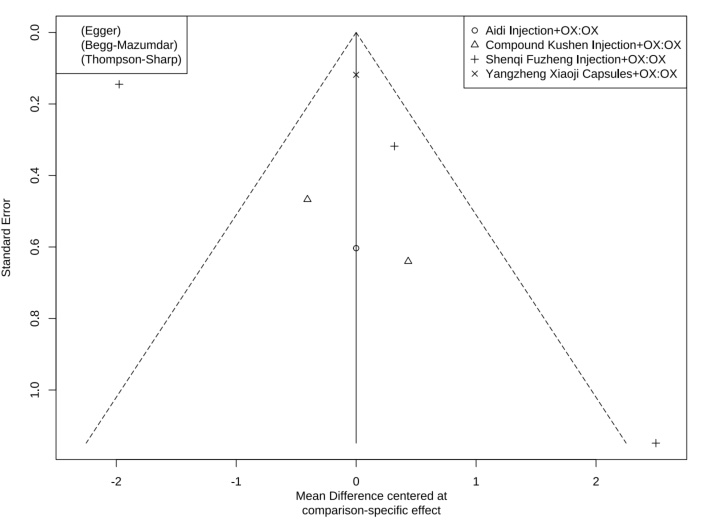


1. **Improvement rate in quality of life**


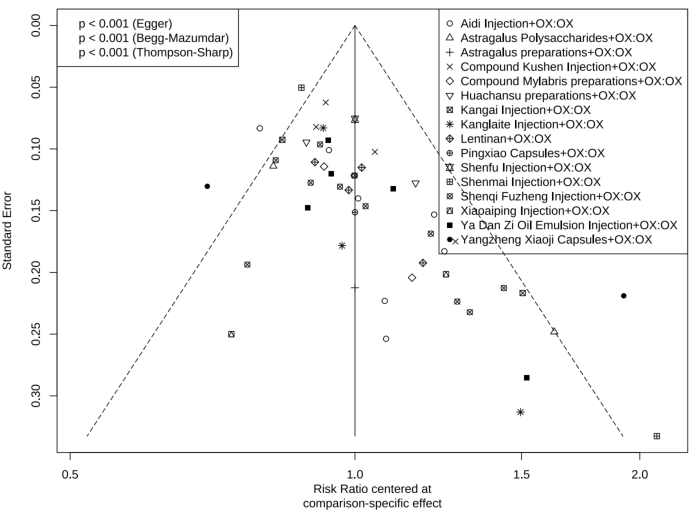


1. **Myelosuppression event**


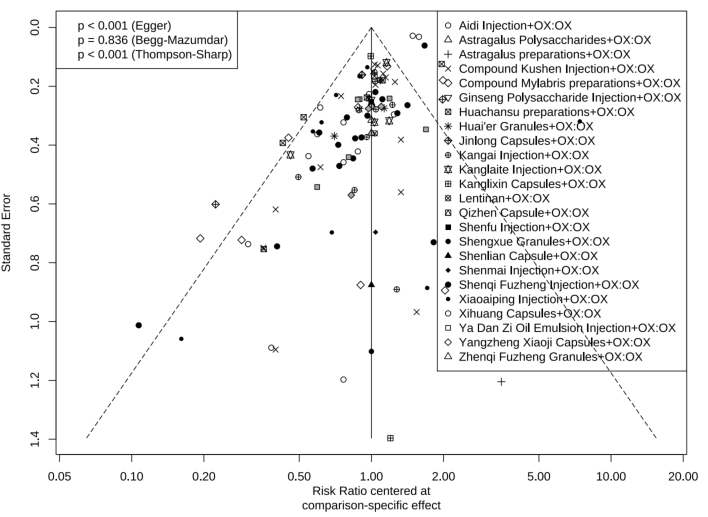


**Q. Gastrointestinal event**


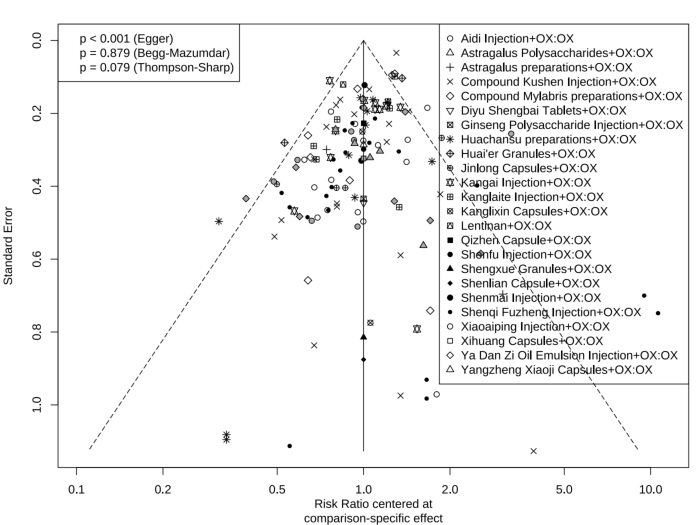

Supplement: Supplementary file 1 [file Supplementaryfile1.zip › Appendix 9-13.DOCX]
