# Supplementary material for: Efficacy and safety of commercial Chinese polyherbal preparation combined with oxaliplatin-based chemotherapy for gastric cancer: a systematic review and network meta-analysis
Source: Front Pharmacol. 2025 Sep 9;16:1645079. doi: 10.3389/fphar.2025.1645079 (PMC12455072; doi:10.3389/fphar.2025.1645079)
Supplement: Supplementary file 1 [file Supplementaryfile1.zip › Appendix 1-8.DOCX]

**Efficacy and Safety of Commercial Chinese Polyherbal Preparation Combined with Oxaliplatin-Based Chemotherapy for Gastric Cancer: A Systematic Review and Network Meta-analysis**

# **Appendix 1 Basic information of included Chinese patent medicine**

| **Pharmacopeial Drug Name** | **Composition** | **Implementation standards** | **Batch numbers** |
| --- | --- | --- | --- |
| Kangai Injection | Huangqi, Renshen, Kushen | China Food and Drug Administration National Drug Standard WS-11222 (ZD-1222)-2002-2012Z | Z20026868 |
| Shenqi Fuzheng Injection | Dangshen, Huangqi | China Food and Drug Administration National Drug Standard WS3-387 (Z-50)-2003(Z)-2011 | Z19990065 |
| Compound Mylabris preparations | Banmao, Renshen, Huangqi, Ciwujia, Sanleng, Banzhilian, Ezhu, Shanzhuyu, Nvzhenzi, Gancao | China Food and Drug Administration National Drug Standard WS3-B-3272-98 | Z20013204  Z20003270  Z19993409  Z19993294  Z20013152  Z52020238  Z20013212 |
| Aidi Injection | Banmao, Renshen, Huangqi, Ciwujia | China Food and Drug Administration National Drug Standard WS3-B-3809-99-2002 | Z52020236 |
| Ya Dan Zi Oil Emulsion Injection | Ya dan zi oil, Dadou Linzhi | China Food and Drug Administration National Drug Standard YBZ12472004 | Z20044247  Z19993152  Z44021325  Z21020639 |
| Huachansu preparations | Ganchanpi | China Food and Drug Administration National Drug Standard YBZ30992005-2011Z | Z20090944  Z20050846 |
| Compound Kushen Injection | Kushen, Baituling | China Food and Drug Administration National Drug Standard WS3-B-2752-97-2014 | Z14021230  Z14021231 |
| Yangzheng Xiaoji Capsules | Huangqi, Nvzhenzi, Renshen, Ezhu, Lingzhi, Baishu, Banzhilian, Fuling, Xuchangqing | Chinese Pharmacopoeia 2015 Edition, Volume I | Z20040095 |
| Kanglixin Capsules | Awei, Dahuang, Jianghuang, Hezi, Dingxiang, Dongchongxiacai | China Food and Drug Administration National Drug Standard WS-10069 (ZD-0069)-2002 | Z20025075 |
| Lentinan | Lentinan | China Food and Drug Administration National Drug Standard YBZ10182008 | Z10920012 |
| Weimaining Capsules | Jinqiaomai | China Food and Drug Administration National Drug Standard WS3-353 (Z-034)-2010Z | Z20010072 |
| Shenmai Injection | Hongshen, Maidong | China Food and Drug Administration National Drug Standard WS3-B-3428-98-2010 | Z20093648  Z51021263  Z53021720  Z13021166  Z51021845 |
| Pingxiao Capsules | Yujin, Maqianzi, Xianhecao, Ganqi, Zhiqiao, Xiaoshi | Chinese Pharmacopoeia 2010 Edition, Volume I | Z61021330 |
| Xiaoaiping Injection | Tongguanteng | China Food and Drug Administration National Drug Standard WS-10630 (ZD-0630)-2002-2013Z-2019 | Z20025869  Z20025868 |
| Kanglaite Injection | Yiyiren | China Food and Drug Administration National Drug Standard WS3-301 (Z-038)-2006(Z)-2013 | Z10970091 |
| Jinlong Capsules | Fresh Tokay Gecko, Fresh Juvenile Banded Krait, Fresh Sharp-snouted Pit Viper | China Food and Drug Administration National Drug Standard WS3-158 (Z-036)-2001(Z) | Z10980041 |
| Huai'er Granules | Huai'er | China Food and Drug Administration National Drug Standard YBZ04202003-2009Z-2012 | Z20000109 |
| Xihuang Capsules | Shexiang, Moyao, ruxiang | China Food and Drug Administration National Drug Standard WS3-B-3210-98-2016 | Z20153041  Z20053216 |
| Astragalus preparations | Astragalus | China Food and Drug Administration National Drug Standard WS-330 (Z-030)-2001 | Z20040085 |
| Qizhen Capsule | Zhenzhu, Huangqi, Sanqi, Daqingye, Chonglou | China Food and Drug Administration National Drug Standard WS3-125 (Z-018)-2004(Z) | Z20010074 |
| Ginseng Polysaccharide Injection | Ginseng Polysaccharide | China Food and Drug Administration National Drug Standard WS-10001-(HD-0302)-2002 | Z20025235 |
| Shengxue Granules | Dangshen, Fuling, Baishu, Huangqi, Dazao | China Food and Drug Administration National Drug Standard WS-11222(ZD-1222)-2002-2012Z | Z20033218 |
| Diyu Shengbai Tablets | Diyu | China Food and Drug Administration National Drug Standard WS-11020 (ZD-1020)-2002-2012Z | Z20026497 |
| Shenlian Capsule | Baibiandou, Banzhilian, Buguzhi, Danshen, Ezhu, Fangji, Kushen, Kuxingren, Sanleng, Shandougen, Wumei | China Food and Drug Administration National Drug Standard WS3-217 (Z-039)-97-(Z) | Z20043139  Z20054114  Z20033068  Z20054816 |
| Compound Tianxian Capsules | Tianhuafen, Weilingxian, Longkui, Sannanxing, ruxiang, Moyao, Renshen, Huangqi, Zhenzhu, Zhuling, Shetui, Shexing | China Food and Drug Administration National Drug Standard WS3-27 (X-18)-91(Z) | Z10880008 |
| Zhenqi Fuzheng Granules | Huangqi, Nvzhenzi | China Food and Drug Administration National Drug Standard WS3-B-3211-98 | Z14020998  Z62020415  Z22022497  Z22026055  Z20053398 |
| Shenfu Injection | Hongshen, Heifupian | China Food and Drug Administration National Drug Standard WS3-B-3427-98-2013 | Z20043116  Z51021920  Z51020664  Z20043117 |
| **Identification** | | |  |
| Kangai Injection | Take 10 ml of this product, add water to make up to 30 ml, extract twice by shaking with 20 ml each time of water-saturated n-butanol. Combine the n-butanol extracts, wash twice with water, discard the aqueous phase, and evaporate the n-butanol to dryness. Dissolve the residue in 0.5 ml methanol to prepare the test solution. Separately, take astragaloside reference standard and dissolve in methanol to prepare a solution containing 1 mg per ml, to be used as the reference solution.  According to the thin-layer chromatography method (Appendix VI B, Volume I, Chinese Pharmacopoeia, 2000 Edition), draw 2 µl each of the above two solutions and spot them respectively on the same silica gel G thin-layer plate. Use the lower layer solution separated at below 10°C from chloroform-methanol-water (13:7:2) as the developing solvent, develop the plate, remove and dry it, spray with 10% ethanolic sulfuric acid solution, and heat at 105°C until spots develop distinctly. Observe the chromatogram under both daylight and ultraviolet light (365 nm). In the test sample chromatogram, at the positions corresponding to those in the reference chromatogram, there should be the same brown spot under daylight, and the same orange-yellow fluorescent spot under ultraviolet light (365 nm) | | |
| Shenqi Fuzheng Injection | 1. Take 100 ml of this product, evaporate to dryness in a water bath, dissolve the residue in 15 ml of methanol, and apply it to a prepared neutral alumina column (100–200 mesh, 5 g, inner diameter 10–15 mm, wet-packed and prewashed with 30 ml methanol). Elute with 60 ml of 40% methanol, collect the eluate, evaporate to dryness in a water bath, dissolve the residue in 15 ml water, and extract twice with 20 ml each time of water-saturated n-butanol. Combine the n-butanol extracts, wash once with 10 ml n-butanol-saturated water, discard the aqueous layer, evaporate the n-butanol to dryness, and dissolve the residue in 0.5 ml methanol to obtain the test solution. Separately, prepare the reference solution by dissolving astragaloside reference standard in methanol to a concentration of 1 mg/ml.  According to the thin-layer chromatography method (Appendix VI B, Volume I, Chinese Pharmacopoeia, 2000 Edition), spot 6 μl of test solution and 2 μl of reference solution respectively onto the same silica gel G plate. Use the lower layer of chloroform–methanol–water (13:7:2) that has been allowed to separate at below 10°C for 12 hours as the developing solvent. Develop at below 10°C, remove, dry, spray with 10% ethanolic sulfuric acid, and heat at 105°C until the spots are clear. On the test sample chromatogram, at corresponding positions to the reference sample, spots of the same color appear. Under UV light (365 nm), an orange-yellow fluorescent spot appears at the same position.  2. Take 4 g of Codonopsis Radix, add an appropriate amount of water, and decoct twice—first for 1 hour, then for 0.5 hour. Combine the decoctions, filter, concentrate the filtrate to 5 ml, cool, and place in an ice water bath. Add ethanol to reach an ethanol content of 80%, stirring during addition to ensure complete precipitation, then filter. Evaporate the filtrate to dryness, dissolve the residue in 15 ml methanol to obtain the reference crude drug solution according to the procedure for the test solution in Identification 1.  According to the thin-layer chromatography method (Appendix VI B, Volume I, Chinese Pharmacopoeia, 2000 Edition), spot 2 μl each of the test solution and the reference crude drug solution on the same silica gel G plate using sodium carboxymethyl cellulose as binder. Use n-butanol–glacial acetic acid–water (7:1:1) as the developing solvent, develop, remove, dry, spray with 10% ethanolic sulfuric acid, and heat at 105°C until the spots appear clearly. Observe under UV lamps at 254 nm and 365 nm. In the test sample chromatogram, at the corresponding position to the reference sample, there should be a spot of the same color and fluorescence.  3. This product should show the identification reactions for sodium salts and chlorides (Appendix IV, Volume I, Chinese Pharmacopoeia, 2000 Edition). | | |
| Compound Mylabris preparations | 1. Cantharidin TLC Identification  Test Solution: Weigh 2 g of capsule contents, add 30 ml chloroform, ultrasonicate to extract, filter, and concentrate the filtrate to 1 ml.  Reference Solution: Cantharidin reference substance dissolved in chloroform (1 mg/ml).  TLC Conditions:  Plate: Silica gel G pre-coated plate  Developing Solvent: Petroleum ether–ethyl acetate (8:2), develop protected from light  Visualization: Spray with 0.5% o-toluidine ethanol solution; observe under UV lamp (365 nm)  Criteria: A blue fluorescent spot with the same color and Rf value (0.55 ± 0.05) as the reference appears in the chromatogram of the test solution.  2. Ginsenoside Rg1/TLC Identification  Test Solution: Weigh 3 g of capsule contents, extract with methanol under reflux, extract the residue with n-butanol, wash with ammonia solution, evaporate to dryness, and redissolve in methanol to a final volume of 2 ml.  Reference Solution: Ginsenoside Rg1 reference substance dissolved in methanol (0.5 mg/ml).  TLC Conditions: Developing Solvent: n-Butanol–ethyl acetate–water (4:1:5), upper layer  Visualization: Spray with 10% ethanolic sulfuric acid, heat at 105°C, observe under both daylight and UV 365 nm  Criteria: Spots of the same purplish-red color appear under daylight, and yellow fluorescent spots under UV, as compared to the reference.  3. Astragaloside Microscopic Identification  Method: Prepare a slide of the capsule contents and observe under a microscope.  Criteria: Astragalus: Presence of crystal sheath fibers (pale yellow, bundled) and fragments of reticulate vessels  Cantharides: Presence of black bristle fragments (serrated edges) | | |
| Aidi Injection | 1. Take 50 ml of this product, concentrate to approximately 10 ml, add 50 ml of ethanol, mix well, and filter. Wash the precipitate twice with ethanol. Dissolve the precipitate in 5 ml of distilled water, take 1 ml and place it in a test tube. Warm gently, then add 5 drops of 5% α-naphthol ethanol solution, shake well, and slowly add 0.5 ml of concentrated sulfuric acid along the wall of the tube. At the interface of the two liquids, a purplish-red ring appears.  2. Take 50 ml of this product, place in a separatory funnel, and extract twice with 30 ml each time of water-saturated n-butanol. Combine the extracts, evaporate to dryness in a water bath, dissolve the residue in 3 ml of distilled water, and apply it to a previously prepared DA-201 resin column (inner diameter 1–1.5 cm, length 15 cm; packed with 12 cm DA-201 resin, topped with 2 g neutral alumina). Wash the column with 100 ml water, then elute with 50 ml of 40% methanol. Collect the eluate, evaporate to dryness, and dissolve the residue in 1 ml methanol to obtain the test solution.  Separately, prepare a mixed reference solution by dissolving ginsenosides Re, Rg₁, Rb, and astragaloside in methanol to give a concentration of 1 mg/ml for each component. According to the Thin Layer Chromatography method (Appendix VI B), spot 5 μl each of the above two solutions on the same silica gel G plate. Use the lower layer of chloroform–ethyl acetate–methanol–water (4:8:3:4), after leaving at below 10°C, as the developing solvent. Develop, remove, dry, spray with 10% ethanolic sulfuric acid solution, and heat at 105°C for several minutes. Observe under UV lamp (365 nm). In the test sample chromatogram, spots of the same fluorescent color as the reference sample should appear at the corresponding positions. | | |
| Ya Dan Zi Oil Emulsion Injection | Take 0.2 ml of this product, add anhydrous ethanol to make up to 10 ml, and measure according to the spectrophotometric method (Appendix V). There is a maximum absorption at 270 nm. | | |
| Huachansu preparations | 1. Identification by Thin Layer Chromatography (TLC)  Reference substances: Cinobufagin, Resibufogenin  Sample preparation: Take the capsule contents and extract with ethanol using ultrasonic extraction → evaporate the filtrate to dryness → dissolve the residue in methanol to obtain the test solution.  Developing solvent: Cyclohexane–chloroform–acetone (4:3:3)  Color development: Spray with 10% ethanolic sulfuric acid solution, heat at 105°C until spots are clear.  Determination: In the test sample chromatogram, spots of the same color appear at positions corresponding to those of the reference substances.  2. Microscopic Identification (required by some standards)  Under the microscope, characteristic secretory clumps of Bufonis Venenum can be observed (faint yellow, semi-transparent, irregular fragments). | | |
| Compound Kushen Injection | 1. Use this product as the test solution. Separately, take reference substances of matrine, sophocarpine, and oxymatrine, dissolve in ethanol to make a mixed reference solution containing 1 mg of each per 1 ml. According to the Thin Layer Chromatography method (Appendix VI B), spot 4 μl each of the test solution and the reference solution on the same silica gel G plate (prepared with 0.4% sodium hydroxide solution). Use benzene–acetone–methanol (8:3:1.5) as the developing solvent. Saturate in ammonia vapor, develop, remove, dry, and spray with modified potassium bismuth iodide reagent. In the test sample chromatogram, three orange-red spots appear at positions corresponding to those in the reference chromatogram.  2. Take 20 ml of this product, add 15 ml of hydrochloric acid and 5 ml of ethanol, hydrolyze under reflux in a boiling water bath for 3 hours, and filter. Evaporate the ethanol from the filtrate in a water bath and concentrate the liquid to about 15 ml. Transfer to a separatory funnel and extract three times with 50 ml of chloroform in total (20, 15, and 15 ml). Combine the chloroform layers, recover the chloroform, and dissolve the residue in 1 ml of ethanol as the test solution. Separately, weigh 10 g of reference Radix Dioscoreae (Baituling), add 100 ml of ethanol, reflux for 1 hour, and filter. Concentrate the filtrate to 1 ml, add 9 ml of hydrochloric acid solution (3 mol/L) and 2 ml of ethanol, and prepare in the same manner to obtain the reference sample solution. According to the Thin Layer Chromatography method (VI B), spot 4 μl each of the above two solutions on the same silica gel G plate, use the upper layer of benzene–acetone–acetic acid (5:1:0.2) as the developing solvent, develop, remove, dry, and observe under UV light at 254 nm. In the test sample chromatogram, yellow-green fluorescent spots appear at positions corresponding to those in the reference sample chromatogram. | | |
| Yangzheng Xiaoji Capsules | 1. Microscopic Identification  Method: Prepare a slide using the contents of the capsule and observe under a microscope.  Standard: Characteristic features of Poria (Fu Ling) are visible—irregular, branched, colorless clumps, which dissolve upon contact with chloral hydrate solution; hyphae appear colorless or pale brown, with a diameter of 4–6 μm.  2. Thin Layer Chromatography (TLC) Identification  Different specific developing systems and color-developing methods are used for different components, as follows:  For Curcuma zedoaria oil, petroleum ether (60–90°C) is used as the developing solvent. The plate is sprayed with 2% vanillin sulfuric acid solution and heated at 105°C. The test sample should show spots of the same color as the reference extract.  For paeonol, cyclohexane-ethyl acetate (6:1) is used as the developing solvent. The plate is sprayed with 2% ferric chloride ethanol solution and heated at 105°C. The test sample should show spots of the same color as the reference standard.  For ginsenosides Rb1 and Rg1, as well as astragaloside IV, n-butanol–ethyl acetate–water (4:1:5, upper layer) is used as the developing solvent. The plate is sprayed with 10% ethanolic sulfuric acid solution and observed under 365 nm UV light. The test sample should show fluorescent bands of the same color as the reference standard.  For Artemisia capillaris (Yinchen), petroleum ether–ethyl acetate–acetone (9:1.5:0.5) is used as the developing solvent. The plate is sprayed with 10% ethanolic sulfuric acid solution and observed under 254 nm UV light. The test sample should show at least one major fluorescent spot matching the reference extract. | | |
| Kanglixin Capsules | 1. Identification of Eugenol (Clove Component)  Test Solution: Take 3 g of the sample, add 30 ml of ether, extract by ultrasound for 20 minutes; concentrate the filtrate to 1 ml.  Reference Solution: Eugenol reference standard (dissolved in ether, 1 mg/ml).  TLC Conditions:  TLC plate: Silica gel G plate  Developing solvent: Petroleum ether–ethyl acetate (9:1)  Visualization: Spray with 10% vanillin–sulfuric acid solution, heat at 105°C for 5 minutes  Identification: The test chromatogram should display a bluish-purple spot (Rf value 0.45 ± 0.05) identical to the reference standard.  2. Identification of Aucklandia (Volatile Oil Component, costus root)  Test Solution: Take 5 g of the sample, add 50 ml of methanol, reflux for 1 hour; concentrate the filtrate to 5 ml.  Reference Herbal Solution:  Prepare similarly using Aucklandia reference herb.  TLC Conditions:  TLC plate: Silica gel GF₂₅₄ plate (fluorescent plate)  Developing solvent: Cyclohexane–ethyl acetate (7:3)  Observation: Examine under a 254 nm UV lamp  Identification: The test chromatogram should display a main blue fluorescent spot identical to the reference herbal chromatogram.  3. Identification of Emodin/Chrysophanol (Rhubarb Components)  Test Solution: Take 2 g of the sample, add 20 ml of methanol, sonicate; evaporate the filtrate to dryness; add 1 ml of hydrochloric acid and 2 ml of ethanol to the residue, hydrolyze for 1 hour; extract with ether; concentrate the ether layer to 1 ml.  Reference Solution: Emodin and chrysophanol mixed reference standard (dissolved in methanol, 0.5 mg/ml each).  TLC Conditions:  TLC plate: Silica gel H plate  Developing solvent: Petroleum ether (60–90°C)–ethyl formate–formic acid (15:5:1)  Observation: Examine under a 365 nm UV lamp  Identification: The test chromatogram should display orange-yellow fluorescent spots identical to the reference standard. | | |
| Lentinan | Molecular Exclusion Chromatography (HPLC-SEC)  Test solution: Take 50 mg of the sample, add 10 ml of 0.9% NaCl solution, dissolve in an 80°C water bath, and filter through a 0.45 μm membrane.  Reference solution: Lentinan reference standard (molecular weight 100–500 kDa, 0.5 mg/ml in 0.9% NaCl solution).  Chromatographic conditions: Column: TSK-GEL G4000PWxl (7.8×300 mm) Mobile phase: 0.1 mol/L Na₂SO₄ solution Flow rate: 0.6 ml/min Detector: Refractive index detector (RID) Column temperature: 35°C  Identification: The main peak of the test sample should have a retention time consistent with the reference (±0.5 min) and a symmetrical peak shape (symmetry factor 0.9–1.2).  Sulfuric Acid–Anthrone Color Reaction  Test solution: Take 20 mg of the sample, dissolve in 5 ml of distilled water, and reserve 0.5 ml for testing.  Reaction method: Take 0.5 ml of the test solution, add 5 ml of freshly prepared 0.2% anthrone in sulfuric acid solution, heat in a boiling water bath for 10 minutes, then cool in cold water.  Identification: The solution should show a blue-green color (with a characteristic absorption at λmax = 620 nm), which distinguishes it from starch adulterants that yield a brown-yellow color.  Infrared Spectrum (IR) Identification  Sample preparation: Take 5 mg of the sample and mix with 200 mg of potassium bromide, then press into a pellet.  Characteristic peaks: At around 3400 cm⁻¹, a strong and broad peak due to O–H stretching vibrations from polysaccharide hydroxyl groups should be present. At around 1650 cm⁻¹, a medium-strong absorption due to C=O stretching vibrations from β-glucan should be observed. At 890 cm⁻¹, a characteristic peak of the β-glycosidic bond must be present; this peak will not be present in α-glucan. | | |
| Weimaining Capsules | Thin Layer Chromatography (TLC) Identification  Target: Characteristic component of Fagopyrum cymosum (procyanidin B2)  Test solution: Take 0.5 g of the sample, add 10 ml of 70% ethanol, extract by ultrasonication, evaporate the filtrate to dryness, and dissolve the residue in 1 ml of methanol.  Reference solution: Procyanidin B2 reference standard, dissolved in methanol at a concentration of 0.2 mg/ml.  TLC conditions:  Plate: High-performance silica gel G plate (Merck, HPTLC Silica Gel 60)  Developing solvent: Toluene–ethyl acetate–formic acid (6:3:1)  Visualization: Spray with 1% vanillin–sulfuric acid solution, heat at 105°C for 5 minutes  Identification: The test chromatogram should show a brick-red spot with the same Rf value as the reference standard (Rf = 0.38 ± 0.03).  Epicatechin HPLC Retention Time Comparison  Test solution: Prepare according to the sample preparation described for content determination (extraction with methanol–0.1% phosphoric acid water).  Reference solution: Epicatechin reference standard, 0.1 mg/ml in methanol.  Chromatographic conditions: Column: Agilent Zorbax SB-C18 (250 × 4.6 mm, 5 μm)  Mobile phase: 0.1% phosphoric acid in water (A) and acetonitrile (B) using gradient elution  Flow rate: 1.0 ml/min  Detection wavelength: 280 nm  Column temperature: 30°C  Gradient program: 0 min (5% B) → 15 min (15% B) → 25 min (20% B)  Identification: The retention time of the epicatechin peak in the test chromatogram (tR ≈ 9.8 min) should match that of the reference standard within ±0.2 min. | | |
| Shenmai Injection | Take 5 ml of the sample, evaporate to dryness in a water bath, then dissolve the residue in 1 ml of ethanol to prepare the test solution. Separately, take reference standards of ginsenoside Rb1, Rg1, and Re, and dissolve in ethanol to prepare a mixed solution containing 2 mg of each per 1 ml, to be used as the reference solution. According to the Thin Layer Chromatography method (Appendix VI B of the Chinese Pharmacopoeia, 2010 Edition, Volume I), spot 2–5 μl each of the test solution and the reference solution on the same silica gel GF254 plate. Use the lower layer (after phase separation below 10°C) of chloroform:methanol:water (13:7:2) as the developing solvent. Develop, remove, dry, spray with 10% ethanolic sulfuric acid solution, and heat at 105°C until the spots are clearly visible. Inspect under UV light at 365 nm. In the chromatogram of the test solution, spots of the same color will appear at positions corresponding to those of the reference standard.  Take 40 ml of the sample and add 3 ml of hydrochloric acid. Heat in a water bath for 1 hour, cool, then extract with 40 ml of diethyl ether by shaking. Evaporate the ether layer to dryness and dissolve the residue in 1 ml of chloroform to prepare the test solution. Separately, take 2 g of Ophiopogon japonicus (Mai Dong) reference medicinal material, decoct with water for 30 minutes, filter, concentrate the filtrate to about 40 ml, and prepare the reference solution in the same manner. According to the Thin Layer Chromatography method (Appendix VI B of the Chinese Pharmacopoeia, 2010 Edition, Volume I), spot 5–10 μl each of the test solution and the reference solution on the same silica gel G plate. Use dichloromethane:acetone (4:1) as the developing solvent. Develop, remove, dry, spray with 10% ethanolic sulfuric acid solution, and heat at 105°C until the spots are clearly visible. In the chromatogram of the test solution, spots of the same color will appear at positions corresponding to those of the reference medicinal material.  Take 1 ml of the sample, dilute to about 10 ml with 50% acetonitrile, and mix well to prepare the test solution. Separately, take an appropriate amount of Shenmai reference extract and make a solution containing 2 mg per 1 ml with 50% acetonitrile as the reference extract solution. According to the High Performance Liquid Chromatography (HPLC) method (Appendix VI D of the Chinese Pharmacopoeia, 2010 Edition, Volume I), use an Alltech PrevailTM Carbohydrate ES column as the stationary phase, acetonitrile:water (80:20) as the mobile phase, and use an evaporative light scattering detector for detection. Inject 10 μl each of the test solution and the reference extract solution into the chromatograph. In the chromatogram of the test solution, four major peaks should be observed at the same retention times as those in the reference extract. | | |
| Pingxiao Capsules | Take the contents of this product and observe under a microscope: single-celled non-glandular hairs resembling fibers can be seen, which are mostly broken; their bases are enlarged, resembling stone cells, and are lignified (characteristic of Nux Vomica powder).  Weigh 4 g of the contents of this product, grind finely, add 10 ml of dichloromethane-ethanol (10:1) mixed solution and 0.5 ml of concentrated ammonia solution, seal, shake for 5 minutes, leave to stand for 2 hours, and filter. Use the filtrate as the test solution. Separately, prepare a reference solution by dissolving 2 mg each of strychnine reference substance and brucine reference substance per 1 ml of dichloromethane. According to the method for Thin Layer Chromatography (General Rule 0502), apply 10 μl each of the test solution and the reference solution on the same silica gel G plate. Use toluene-acetone-ethanol-concentrated ammonia solution (4:5:0.6:0.4) as the developing solvent. Develop, remove, dry, and spray with dilute potassium bismuth iodide solution. In the chromatogram of the test solution, spots of the same color appear at the corresponding positions as those in the reference solution.  Weigh 2 g of the contents of this product, add 20 ml of methanol, heat under reflux for 2 hours, and filter. Use the filtrate as the test solution. Separately, dissolve naringin reference substance in methanol to make a solution containing 1 mg per 1 ml as the reference solution. According to the method for Thin Layer Chromatography (General Rule 0502), apply 6 μl of the test solution and 4 μl of the reference solution on the same silica gel G plate. Use the upper layer of ethyl acetate-formic acid-water (10:2:3) as the developing solvent. Develop, remove, dry, spray with aluminum chloride solution, and dry again. Observe under a UV lamp (365 nm). In the chromatogram of the test solution, fluorescent spots of the same color appear at the corresponding positions as those in the reference solution. | | |
| Xiaoaiping Injection | Take 10 ml of this product, add water to make a total of 20 ml, then add 1 ml of concentrated ammonia solution. Wash twice with 20 ml of chloroform each time by shaking; discard the chloroform layers. Wash the remaining aqueous layer twice with 15 ml of n-butanol each time by shaking; discard the n-butanol layers. Concentrate the aqueous layer to dryness and dissolve the residue in 1 ml of methanol. Allow to stand, then use the supernatant as the test solution.  Separately, take 2 g of Ventilago (Tongguan Teng) reference medicinal material, add 50 ml of water, soak for 24 hours, treat with ultrasound for 20 minutes, then filter. Concentrate the filtrate to 20 ml and prepare the reference solution in the same manner.  Following the Thin Layer Chromatography method (Appendix VI B of the Chinese Pharmacopoeia, 2000 Edition, Volume I), spot 5 µl each of the above two solutions on the same silica gel GF254 plate. Use chloroform–acetone–formic acid (15:3:2) as the developing solvent. Develop, remove, dry, and observe under a UV lamp (254 nm). In the chromatogram of the test solution, spots of the same color appear at the corresponding positions as in the chromatogram of the reference medicinal material. | | |
| Kanglaite Injection | Take 5 ml of the sample and heat in a water bath to allow phase separation. Collect the oil layer and prepare a solution with petroleum ether (60–90℃) to a concentration of 40 mg/ml.  Reference Solution: Prepare a reference solution of Coix seed oil in the same manner, making a 40 mg/ml solution in petroleum ether.  Thin Layer Chromatography Conditions:  Plate: Silica gel G  Developing solvent: Petroleum ether–diethyl ether–acetic acid (9:1:0.1)  Visualization: Spray with 5% vanillin–sulfuric acid solution and heat at 105°C for 5 minutes.  Judgment Criteria:  In the chromatogram of the test solution, spots of the same color (typically purplish red or blue-purple) should appear at the same positions as those in the chromatogram of the reference solution. | | |
| Jinlong Capsules | Test solution: Weigh 2 g of the contents, add 20 ml of methanol, extract by ultrasonication, filter, and concentrate the filtrate to 2 ml.  Reference material: Prepare reference gecko medicinal material solution in the same manner.  Thin layer chromatography conditions:  Plate: Silica gel G  Developing solvent: Toluene–ethyl acetate–glacial acetic acid (12:4:0.5)  Visualization: Spray with 10% sulfuric acid in ethanol, then heat at 105°C for 5 minutes.  Criteria: In the chromatogram of the test solution, purple-red spots (Rf value 0.35 ± 0.05) should appear at the corresponding positions as those in the chromatogram of the reference medicinal material.  Test solution: Weigh 3 g of the contents, add 10 ml of 0.9% NaCl solution, centrifuge, and collect the supernatant.  Reference material: Prepare reference Agkistrodon medicinal material decoction solution in the same manner.  Thin layer chromatography conditions:  Plate: Silica gel GF₂₅₄  Developing solvent: n-butanol–glacial acetic acid–water (4:1:1)  Detection: Observe under a UV lamp at 365 nm.  Criteria: In the chromatogram of the test solution, blue fluorescent spots should appear at the same positions as those in the chromatogram of the reference medicinal material. | | |
| Huai'er Granules | Thin Layer Chromatography (TLC) Identification  Target: Characteristic polyphenol from Polyporus umbellatus (Ellagic acid)  Test solution: Take 1 g of the granules, add 10 ml of a methanol–25% hydrochloric acid mixture (4:1), hydrolyze in a 90°C water bath for 30 minutes, cool, extract with ethyl acetate, and concentrate the extract to 1 ml.  Reference solution: Ellagic acid reference standard, dissolved in methanol at 0.1 mg/ml.  TLC conditions:  Plate: High-performance silica gel GF254 plate (Merck)  Developing solvent: Toluene–ethyl formate–formic acid (5:4:1)  Observation: Under 365 nm UV lamp  Identification: The test chromatogram should display a bright blue fluorescent spot with the same Rf value as the reference (Rf = 0.50 ± 0.05).  Polysaccharide Infrared Spectrum (IR) Identification  Sample preparation: Take 0.5 g of granules, defat with anhydrous ethanol, dissolve the residue in water, remove salts by dialysis, freeze-dry to obtain polysaccharide powder, and press into a pellet with potassium bromide.  Characteristic peaks: A broad and strong absorption at around 3400 cm⁻¹, corresponding to O–H stretching vibration of polysaccharide hydroxyl groups;  A moderate absorption at 1630 cm⁻¹, due to bending vibration of water (H–O–H);  A strong peak at 1070 cm⁻¹, characteristic of the C–O–C pyranose ring;  A fingerprint peak at 890 cm⁻¹, characteristic of β-glycosidic bonds, which should be present (distinguishing from α-glucan). | | |
| Xihuang Capsules | Bovine Gallstone Thin Layer Chromatography (TLC) Identification  Test solution: Take 1 g of the sample, add 20 ml methanol, extract by ultrasonication, evaporate the filtrate to dryness, dissolve the residue in 10 ml of 10% NaOH solution, extract with ether (discard the ether layer), acidify the aqueous layer to pH 2, then extract with ethyl acetate. Concentrate the ethyl acetate layer to 1 ml.  Reference solution: Mixed reference standard of cholic acid and deoxycholic acid, each at 0.5 mg/ml in methanol.  TLC conditions:  Plate: Silica gel G plate  Developing solvent: Isooctane–ethyl acetate–glacial acetic acid (15:7:5)  Visualization: Spray with 10% sulfuric acid in ethanol, heat at 105°C for 5 minutes.  Identification: The test chromatogram should show two purple-red spots corresponding to the reference standards, with Rf values of approximately 0.35 for cholic acid and 0.50 for deoxycholic acid.  Musk Microscopy and TLC Dual Identification  Microscopic Identification: Prepare a specimen of the sample. Under the microscope, observe muscone crystals (colorless, semi-transparent blocky crystals with the characteristic musk odor) and fragments of epidermal tissue (nearly round hair follicle structures, diameter 40–60 μm).  TLC Identification: Test solution: 2 g of sample, ultrasonically extracted with 10 ml cyclohexane, concentrate filtrate to 1 ml.  Reference standard: Muscone in cyclohexane, 0.1 mg/ml.  Developing solvent: Toluene  Visualization: Spray with 2,4-dinitrophenylhydrazine in ethanol.  Identification: Observe a yellow spot with the same Rf value as the reference (Rf ≈ 0.65).  Combined Identification of Toad Venom and Frankincense  Toad Venom (Chansu) TLC:  Test solution: 1 g sample, extracted with chloroform–methanol (1:1), concentrate filtrate.  Reference standard: Bufotoxin (1 mg/ml in methanol).  Developing solvent: Cyclohexane–acetone (4:1)  Visualization: Spray with 0.065% malachite green solution, observe under daylight.  Identification: The test chromatogram should show a blue-green spot (Rf ≈ 0.55) matching the reference standard.  Frankincense Color Reaction: Take 0.5 g of sample, boil with 5 ml water, filter. Add 0.5% ferric chloride in ethanol to the filtrate. The solution develops a dark green color, characteristic of frankincense resin acids. | | |
| Astragalus preparations | / | | |
| Qizhen Capsule | 1. Microscopic Identification:  Take the sample and observe under a microscope: irregular fragments, semi-transparent, with a granular surface. The fragments consist of several to dozens of thin layers overlapping tightly, exhibiting a sheet-like structure. Compact stratified lines or very fine wavy textures can be observed.  2. Calcium Salt Reaction:  Take 0.5 g of the sample contents, add dilute hydrochloric acid; a large amount of bubbles forms immediately. Filter, and the filtrate shows identification reactions for calcium salts (according to Appendix IV of the Chinese Pharmacopoeia, 2000 Edition, Volume I).  3. Thin Layer Chromatography for Indigo Carmine:  Take 1 g of the sample contents, add 25 ml of chloroform, heat under reflux for 1 hour, and filter. Evaporate the filtrate to dryness, dissolve the residue in 1 ml of chloroform to obtain the test solution.  Separately, prepare a reference solution of Indigo Carmine in chloroform at a concentration of 0.1 mg/ml.  According to Thin Layer Chromatography (Appendix VI B of the Chinese Pharmacopoeia, 2000 Edition, Volume I), spot 5 μl each of the test and reference solutions onto the same silica gel G TLC plate. Use benzene–chloroform–acetone (5:4:1) as the developing solvent, develop, remove, and dry. In the chromatogram of the test solution, a spot with the same color appears at the same position as the reference solution.  4. Thin Layer Chromatography for Ginsenoside Rg1 and Notoginsenoside R1:  Take 0.5 g of the sample contents, add 20 ml of water-saturated n-butanol, sonicate for 30 minutes, and filter. Wash the filtrate with 40 ml n-butanol-saturated water and discard the water layer. Wash the n-butanol layer with 40 ml ammonia solution and discard the ammonia layer. Evaporate the n-butanol layer to dryness, dissolve the residue in 1 ml of anhydrous ethanol to obtain the test solution.  Separately, prepare reference solutions of ginsenoside Rg1 and notoginsenoside R1 in anhydrous ethanol at a concentration of 1 mg/ml each.  According to Thin Layer Chromatography (Appendix VI B of the Chinese Pharmacopoeia, 2000 Edition, Volume I), spot 4 μl each of the three solutions onto the same silica gel G TLC plate. Use chloroform–ethyl acetate–methanol–water (15:40:22:10, lower phase kept below 10°C) as the developing solvent, develop, remove, and dry. Spray with 10% ethanol-sulfuric acid solution and heat at 110°C for several minutes. The chromatogram of the test solution reveals spots with the same color at the corresponding positions as the reference substances.  5. Thin Layer Chromatography for Paris Polyphylla:  Take 0.5 g of the sample contents, add 10 ml of anhydrous ethanol, sonicate for 30 minutes, and filter. Evaporate the filtrate to dryness, add 5 ml of 2 mol/L hydrochloric acid solution, and heat for 2 hours for hydrolysis. After cooling, extract three times with 5 ml petroleum ether (60–90°C) each time, combine the extracts, wash with water to neutral, and evaporate the petroleum ether layer to dryness. Dissolve the residue in 1 ml of chloroform to obtain the test solution.  Separately, take 0.2 g of Paris Polyphylla reference medicinal material and prepare the reference solution in the same way.  According to Thin Layer Chromatography (Appendix VI B of the Chinese Pharmacopoeia, 2000 Edition, Volume I), spot 10 μl of the test solution and 4 μl of the reference solution onto the same silica gel G TLC plate. Use cyclohexane–ethyl acetate (4:1) as the developing solvent, develop, remove, and dry. Spray with 10% ethanol-sulfuric acid solution and heat at 110°C for several minutes. In the chromatogram of the test solution, spots with the same color appear at the same positions as those in the reference chromatogram. | | |
| Ginseng Polysaccharide Injection | Dissolve the sample in water to obtain a solution containing 0.5 mg per 1 ml. Take 0.2 ml of this solution, add 3 ml of 0.25 mol/L borax–sulfuric acid solution, mix well, and heat in a water bath for 15 minutes. Remove and cool in ice water for 5 minutes. Add 2 drops of 0.125% carbazole anhydrous ethanol solution, mix, and let stand at room temperature for 1–2 minutes. The solution will turn purplish red. | | |
| Shengxue Granules | 1. Astragalus (Astragaloside IV) TLC Identification  Test solution: Weigh 5 g granules, add 50 ml methanol, reflux for 1 hour. Evaporate the filtrate to dryness, dissolve residue in 20 ml water. Defat the aqueous solution with ether. Extract the aqueous layer with n-butanol, evaporate the n-butanol layer to dryness, and dissolve the residue in 1 ml methanol.  Reference solution: Astragaloside IV reference standard, dissolved in methanol at 1 mg/ml.  TLC conditions:  Plate: Silica gel G plate  Developing solvent: Chloroform–methanol–water (13:7:2), lower layer  Visualization: Spray with 10% sulfuric acid in ethanol, heat at 105°C for 5 minutes.  Identification: The test chromatogram should display a brown spot at the same Rf value as the reference standard (Rf = 0.35 ± 0.03).  2. Angelica sinensis (Ferulic Acid) TLC Identification  Test solution: Weigh 3 g granules, add 20 ml of 70% ethanol, extract by ultrasonication. Evaporate the filtrate to dryness, dissolve residue in 10 ml of 1% sodium bicarbonate solution. Extract with ether (discard the ether layer), acidify the aqueous layer to pH 2, then extract with ether again. Concentrate the ether layer to 1 ml.  Reference solution: Ferulic acid reference standard, dissolved in methanol at 0.5 mg/ml.  TLC conditions:  Plate: Silica gel GF254 plate  Developing solvent: Cyclohexane–ethyl acetate–glacial acetic acid (8:2:0.5)  Observation: Under 365 nm UV lamp  Identification: The test chromatogram should display a blue fluorescent spot at the same Rf value as the reference standard (Rf = 0.40 ± 0.05).  3. Rubia cordifolia (Rubiadin) TLC Identification  Test solution: Weigh 2 g granules, add 20 ml ether, extract by ultrasonication, and concentrate the filtrate to 1 ml.  Reference solution: Rubiadin reference standard, dissolved in ether at 0.2 mg/ml.  TLC conditions:  Plate: Silica gel G plate  Developing solvent: Petroleum ether–acetone (8:2)  Observation: Under 365 nm UV lamp  Identification: The test chromatogram should display a sky blue fluorescent spot at the same Rf value as the reference standard (Rf = 0.55 ± 0.05). | | |
| Diyu Shengbai Tablets | 1. Microscopic Identification (Characteristic Features of Sanguisorba officinalis)  Slide preparation: Take a small amount of inner stem powder, treat with chloral hydrate for clearing, and prepare a slide.  Characteristic features: Sanguisorba bast fibers: Solitary and scattered, thick-walled, lumen linear, 10–25 μm in diameter. Calcium oxalate cluster crystals: 20–65 μm in diameter, with sharp angles.  Cork cells: Reddish-brown, polygonal, thin-walled.  2. Sanguisorba Saponin TLC Identification  Test solution: Weigh 2 g inner stem powder, add 20 ml 70% ethanol, extract by ultrasonication. Evaporate the filtrate to dryness, add 10 ml 1 mol/L HCl, hydrolyze for 30 minutes, neutralize with NaOH, extract with ethyl acetate. Concentrate the ethyl acetate layer to 1 ml.  Reference solution: Sanguisorba Saponin I reference standard, dissolved in methanol at 0.5 mg/ml.  TLC conditions:  Plate: Silica gel G plate  Developing solvent: Chloroform–methanol–water (7:3:1), lower layer  Visualization: Spray with 10% vanillin–sulfuric acid solution, heat at 105 °C for 5 minutes.  Identification: The test chromatogram should display a purple-red spot at the same Rf value as the reference standard (Rf = 0.35 ± 0.03).  3. Tannin Color Reaction  Test solution: Take 0.5 g inner stem powder, add 10 ml water, boil, and filter after cooling.  Test and identification: Add 1% FeCl₃ solution: A blue-black color appears, indicating a positive reaction typical for tannins. Add gelatin–sodium chloride solution: Formation of white precipitate, positive result, confirming the presence of true tannins (excludes pseudo-tannin interference). | | |
| Shenlian Capsule | 1. Sophora flavescens (Matrine/Oxymatrine) TLC Identification  Test solution: Take 2 g of sample, moisten with 0.5 ml concentrated ammonia solution. Add 30 ml chloroform, extract by ultrasonication. Evaporate the filtrate to dryness, and dissolve the residue in 1 ml ethanol.  Reference solution: Mixed reference solution of matrine and oxymatrine, each at 1 mg/ml in ethanol.  TLC conditions:  Plate: Silica gel G plate modified with 0.4% NaOH (to enhance separation)  Developing solvent: Toluene–acetone–methanol (8:3:1.5)  Visualization: Spray with modified bismuth potassium iodide reagent  Identification: The test chromatogram should reveal two orange-red spots with the same Rf values as the references (matrine Rf ≈ 0.35, oxymatrine Rf ≈ 0.50).  2. Sophora tonkinensis (Sophoranone) TLC Identification  Test solution: Take 3 g sample, add 50 ml ethanol, reflux. Acidify the filtrate with hydrochloric acid to pH 2. Extract with ether (discard ether layer). Alkalinize the aqueous layer to pH 10, then extract with chloroform. Concentrate the chloroform layer to 1 ml.  Reference solution: Sophoranone reference, dissolved in chloroform at 0.5 mg/ml.  TLC conditions:  Plate: Silica gel GF254 plate  Developing solvent: Cyclohexane–chloroform–methanol (7:3:1)  Observation: 365 nm UV light  Identification: The test chromatogram should show a bright green fluorescent spot with the same Rf value as the reference (Rf ≈ 0.45).  3. Psoralea corylifolia Microscopic Identification  Slide preparation:  Prepare a slide of the sample and mount using glycerin-acetic acid solution.  Diagnostic features: Seed coat palisade cells: In side view, arranged in a single row, long columnar, thick-walled, with a distinct bright band.  Glandular hairs: Nearly spherical heads, composed of 4–8 cells, with a single-celled stalk. | | |
| Compound Tianxian Capsules | 1. Microscopic Identification:  Take the sample contents and observe under a microscope: cork cells are square or polygonal, thin-walled, and light yellow-brown; reticulate and scalariform vessels have diameters of 25–45 μm; calcium oxalate cluster crystals have diameters of 25–70 μm; muscle tissue fragments with fine, wavy transverse striations can be seen.  2. Thin Layer Chromatography Identification:  Take 1 g of the sample contents, add 40 ml chloroform, heat under reflux in a water bath for 1 hour, filter, and concentrate the filtrate in a water bath to about 1 ml; this is test solution A. Dry the residue to remove solvent, moisten by mixing with 0.5 ml water, add 10 ml water-saturated n-butanol, ultrasonicate for 1 hour, collect the supernatant, add 30 ml ammonia solution, shake well, allow to separate layers, take the upper layer, evaporate to dryness, and dissolve the residue in 1 ml methanol; this is test solution B.  (1) Prepare a ginseng reference material solution by processing ginseng medicinal material as for test solution B, at a concentration of 1 g per 1 ml. Separately, prepare a mixed solution of ginsenoside Rb1, Re, and Rg1 reference substances, 2 mg of each per 1 ml methanol.  According to thin layer chromatography (Chinese Pharmacopoeia 1990 Edition, Volume I, Appendix p.57), spot 2 μl each of test solution B, ginseng reference material solution, and reference substance mixture onto the same silica gel G TLC plate. Develop with chloroform–ethyl acetate–methanol–water (15:40:22:10), using the lower layer stored below 10°C. Remove, dry, spray with 10% sulfuric acid–ethanol solution, heat at 105°C for several minutes, and inspect in daylight. On the chromatogram of the test solution, spots of the same color appear at the corresponding positions of the reference material; at the reference substance positions, three identical purple spots appear.  (2) Prepare a solution of cholic acid reference substance at 1 mg/ml in methanol.  According to thin layer chromatography (Chinese Pharmacopoeia 1990 Edition, Volume I, Appendix p.57), spot 2 μl each of test solution A and reference substance solution onto the same silica gel G TLC plate. Develop with isooctane–ethyl acetate–glacial acetic acid (15:7:5). Remove, dry, and spray with 10% sulfuric acid–ethanol solution. Heat at 105°C until spots are clearly colored, then observe under UV lamp at 365 nm. On the chromatogram of the test solution, blue-violet fluorescent spots are visible at corresponding positions as the reference substance.  (3) Prepare a mixed solution of indigo and indigotin reference substances, each at 1 mg/ml in chloroform.  According to thin layer chromatography (Chinese Pharmacopoeia 1990 Edition, Volume I, Appendix p.57), spot 2 μl each of test solution A and reference substance mixture onto the same silica gel G TLC plate. Develop with benzene–chloroform–acetone (5:4:1). Remove, dry, and blow with hot air until spots are clearly colored. On the chromatogram of the test solution, blue-purplish spots appear at the corresponding positions to the reference substance. | | |
| Zhenqi Fuzheng Granules | 1. Astragalus (Astragaloside IV) TLC Identification  Test solution:Weigh 10 g of granules, add 50 ml methanol, and reflux. Evaporate the filtrate to dryness, dissolve the residue in 30 ml water. Defat the solution with ether, then extract the aqueous layer with n-butanol. Evaporate the n-butanol layer to dryness, and dissolve the residue in 1 ml methanol.  Reference solution: Dissolve astragaloside IV reference standard in methanol to make a 1 mg/ml solution.  TLC conditions: Use a silica gel G plate. The developing solvent is chloroform–methanol–water (13:7:2), using the lower layer. For visualization, spray with 10% sulfuric acid in ethanol and heat at 105 °C for 5 minutes.  Judgement: The test sample should show a brown spot with the same Rf value as the reference (Rf = 0.35 ± 0.03).  2. Ligustrum lucidum (Oleanolic Acid) TLC Identification  Test solution: Weigh 5 g granules, add 30 ml ethanol, and ultrasonicate. Evaporate the filtrate to dryness, add 10% KOH ethanol solution (10 ml) for hydrolysis. Adjust to neutral with hydrochloric acid, extract with ether, and concentrate the ether layer to 1 ml.  Reference solution: Dissolve oleanolic acid reference standard in ether at 0.5 mg/ml.  TLC conditions: Use a silica gel G plate. The developing solvent is cyclohexane–acetone–ethyl acetate (5:2:1). For visualization, spray with 10% phosphomolybdic acid ethanol solution and heat at 105 °C.  Judgement: The test sample should show a blue-gray spot with the same Rf value as the reference (Rf = 0.50 ± 0.05).  3. Microscopic Identification (Astragalus & Ligustrum lucidum Features)  Slide preparation: Make a slide from the inner content of the granules, clear with chloral hydrate.  Identification features:  For Astragalus: Fiber bundles are observed with thick cell walls, and the broken ends of the fibers appear broom-like. These fibers are 15 to 25 μm in diameter. The thin-walled cells surrounding the bundle contain calcium oxalate prisms.  For Ligustrum lucidum: In surface view, the pericarp epidermal cells are polygonal. Stomata are anomocytic type, each with 4 to 5 subsidiary cells. | | |
| Shenfu Injection | 1. Identification Reaction:  Take 1 ml of the sample, place it in an evaporating dish, and evaporate to dryness in a water bath. Dissolve the residue with 0.5 ml acetic anhydride, transfer to a test tube, and carefully add 0.5 ml sulfuric acid along the tube wall. A brownish-red ring appears at the interface of the two liquids.  2. Thin Layer Chromatography (TLC) Identification:  Take 30 ml of the sample, place it in a separatory funnel, add 30 ml chloroform, shake well, and allow to stand. Collect the upper layer, evaporate to dryness, dissolve the residue in 2 ml water, add 10 ml water-saturated n-butanol, and sonicate for 30 minutes. Collect the supernatant, add 3 times its volume of ammonia solution, shake well, and allow to separate layers. Collect the upper layer and evaporate to dryness; dissolve the residue in 1 ml methanol as the test solution.  Separately, prepare a reference herbal material solution: Take 1 g of red ginseng as reference material, add 30 ml ethanol, reflux in a water bath for 30 minutes, filter, evaporate the filtrate to dryness, dissolve the residue in 30 ml water, filter, and process the filtrate in the same manner as above to obtain the reference herbal material solution.  Prepare a mixed reference solution of ginsenosides Rb1, Re, and Rg1 in methanol, each at 2 mg/ml.  According to the method for Thin Layer Chromatography (Appendix VI B), spot 10 μl of the test solution, 20 μl of the reference herbal solution, and 2 μl of the mixed reference solution onto the same silica gel G TLC plate. Develop with chloroform–ethyl acetate–methanol–water (15:40:22:10, use the lower layer stored at below 10°C) as the developing solvent. Remove and dry the plate, spray with 10% ethanolic sulfuric acid solution, and heat at 105°C until spots are clearly visible.  In the chromatogram of the test solution, spots of identical color appear at the corresponding positions as those in the chromatogram of the reference herbal material and the reference substance. | | |
| **Assay** | | |  |
| Kangai Injection | pH Value: Should be 4.0–7.0 (Chinese Pharmacopoeia 2000 Edition, Volume I, Appendix VII G).  Ignited Residue: Take 10 ml of the sample and test according to the method (Chinese Pharmacopoeia 2000 Edition, Volume I, Appendix IX J); should not exceed 1.5% (g/ml).  Hemolysis Test: Preparation of 2% Red Blood Cell Suspension:  Collect blood from the heart of a rabbit, place it in a container with glass beads, shake for 10 minutes to remove fibrinogen and obtain defibrinated blood. Add normal saline, mix well, centrifuge, and decant the supernatant. Wash the red blood cell precipitate 3–4 times with normal saline until the supernatant is colorless upon centrifugation. Dilute the obtained red blood cells with normal saline to make a 2% suspension, to be used on the same day; shake well before use.  Test Method: Prepare five test tubes and label them. Add 0.3 ml of the sample and 2.2 ml normal saline to tubes 1–3; add 2.5 ml normal saline to tube 4 (negative control); add 2.5 ml distilled water to tube 5 (positive control). Then, add 2.5 ml of 2% red blood cell suspension to each tube. Place the tubes in a thermostatic incubator at 36.5 ± 0.5°C for 3 hours. No hemolysis should be observed.  Pyrogen Test: Test according to the method (Chinese Pharmacopoeia 2000 Edition, Volume I, Appendix XIII A); the dose should be 2 ml per kg rabbit body weight by injection, and results should meet the specified requirements.  Others: Should comply with all relevant regulations for injection preparations and related substances as specified under the Chinese Pharmacopoeia 2000 Edition, Volume I, Appendix IU and IX S. | | |
| Shenqi Fuzheng Injection | pH Value: Should be 4.5–6.5 (Chinese Pharmacopoeia 2000 Edition, Volume I, Appendix VIII G).  Protein: Take 1 ml of the sample, add 1–3 drops of tannic acid test solution; no turbidity should form.  Tannins: Take 1 ml of the sample, add 1 drop of dilute acetic acid, then 4–5 drops of sodium chloride-gelatin test solution; no turbidity or precipitation should occur.  Oxalate: Take 2 ml of the sample, add 2–3 drops of 3% calcium chloride test solution, let stand for 10 minutes; no turbidity or precipitation should occur.  Potassium Ion: Test according to the method for substances related to injection preparations (Chinese Pharmacopoeia 2000 Edition, Volume I, Appendix IX S); should comply with requirements.  Resins: Take 5 ml of the sample, add 1 drop of hydrochloric acid, let stand for 30 minutes; no flocculent precipitate should form.  Ignited Residue: Precisely measure 10 ml of the sample, evaporate to dryness, test according to the method (Chinese Pharmacopoeia 2000 Edition, Volume I, Appendix IX J); residue should not exceed 1.5% (g/ml).  Heavy Metals: Use the residue obtained from the ignited residue test, and test according to the second method in the appendix (Chinese Pharmacopoeia 2000 Edition, Volume I, Appendix IX E); heavy metals should not exceed 0.0005 (five parts per million).  Arsenic Salts: Take 2 ml of the sample, place in a crucible, add 0.5 ml nitric acid and 0.25 ml perchloric acid; heat gently until fumes dissipate, then continue heating more strongly for several minutes. Cool, add about 1 ml water, add 5–10 drops hydrochloric acid, evaporate to dryness in water bath, add 23 ml water and heat to dissolve, then add 5 ml hydrochloric acid. Separately treat 2 ml standard arsenic solution in the same fashion, then test according to the first method (Chinese Pharmacopoeia 2000 Edition, Volume I, Appendix IX F); arsenic content should not exceed 0.0001 (one part per million).  Pyrogen Test: Test as prescribed (Chinese Pharmacopoeia 2000 Edition, Volume I, Appendix XIII A); dose: slowly inject 10 ml per kg rabbit body weight, and should meet requirements.  Others: Should comply with all relevant regulations for injection preparations (Chinese Pharmacopoeia 2000 Edition, Volume I, Appendix IU). | | |
| Compound Mylabris preparations | pH Value  Method: Potentiometric method (General Rule 0631)  Sample preparation:  Injections: Measure directly.  Capsules: Weigh 1 g of contents, add 20 ml water, shake well, centrifuge and use the supernatant.  Standards:  Injection: pH 6.8–7.2. Values outside this range may cause vascular irritation or precipitation.  Capsule contents: pH 4.5–6.0. Excessive acidity may accelerate hydrolysis and inactivation of cantharidin.  Ignition Residue (Control of Inorganic Impurities)  Method: High-temperature ashing (General Rule 0841)  Steps: Weigh 1.0 g of sample and char in a crucible (electric furnace). Transfer to a muffle furnace at 700–800°C and ignite for 4 hours. Cool and weigh the residue.  Limits:  Capsules: ≤ 3.0% (limit for excipient carbonization residue)  Injection: ≤ 0.5% (strict control of inorganic salts)  Hemolysis and Agglutination Test (Required for Injections)  Method: In vitro rabbit erythrocyte test (General Rule 1122)  Procedure: Prepare a 2% rabbit erythrocyte saline suspension. Mix 0.1 ml test solution with 0.9 ml erythrocyte suspension. Positive control: 0.1 ml distilled water + 0.9 ml erythrocyte suspension.  Negative control: 0.1 ml saline + 0.9 ml erythrocyte suspension. Incubate at 37°C for 3 hours.  Judgement: Hemolysis: Test tube solution becomes red and transparent (hemolysis rate ≥ 5% is unqualified).  Agglutination: Erythrocytes aggregate into clumps under the microscope (aggregation of ≥ 5 cells per clump is considered positive).  Pyrogen Test (For Injections and Tumor Injection Agents)  Method: Rabbit Pyrogen Test (General Rule 1142) or Limulus Amebocyte Lysate Test (General Rule 1143)  Rabbit Method:  Qualified rabbits: Body weight 1.5–2.5 kg; normal temperature range 38.0–39.8°C.  Injection dose: 1.0 ml/kg (slow intravenous injection via ear vein).  Measurement: Record temperature before injection, then at 0.5, 1, 2, and 3 hours post-injection.  Standard: The sum of temperature increases in three rabbits should be ≤ 1.4°C, and in any single rabbit, ≤ 0.6°C.  Abnormal Toxicity Test (For Cantharidin Risk Control)  Method: Mouse test (General Rule 1141)  Procedure: Weigh 0.5 g sample, add 10 ml saline. Extract ultrasonically, centrifuge, and use supernatant as test solution.  Select 5 mice (18–20 g), inject 0.5 ml/20 g via tail vein.  Observe for 48 hours.  Standard: No more than one mouse should die (if two or more die, retesting is required; if one or more die on retest, sample fails). | | |
| Aidi Injection | pH Value: Should be 3.8–5.0 (Appendix VII G).  Heavy Metals: Accurately measure 2 ml of the sample, place in a crucible, and evaporate to dryness in a water bath. Test according to the second method in Appendix IX E; heavy metals content should not exceed 0.0005 (five parts per million).  Pyrogen Test: Test as prescribed (Appendix VIII A); dilute the sample injection 10 times to the clinical concentration with corresponding pyrogen-free injection solution, and inject 3 ml per kg rabbit body weight. The result should meet requirements.  Others: Should comply with all relevant requirements for injection preparations (Appendix IU). | | |
| Ya Dan Zi Oil Emulsion Injection | pH Value: Should be 4.0–6.0 (Appendix VII G).  Particle Size: Take 1 drop of the sample and 1 drop of 50% glycerin solution, and observe under a microscope (400× magnification); the maximum particle size should not exceed 15 μm.  Pyrogen Test: Test as prescribed (Appendix VIII A); administer 1 ml per kg rabbit body weight. The result should meet the requirements.  Sterility: Test as prescribed (Appendix VIII B); the result should meet the requirements.  Others: Should comply with all relevant requirements for injection preparations (Appendix IU). | | |
| Huachansu preparations | 1. Content Determination (HPLC Method)  Target Components: Cinobufagin and Resibufogenin  Chromatographic Conditions: Column: C18 reversed-phase column (250 mm × 4.6 mm, 5 μm)  Mobile phase: Acetonitrile–0.5% potassium dihydrogen phosphate solution (50:50)  Detection wavelength: 296 nm  Limit Requirement: Each capsule should contain a total amount of bufadienolides, calculated as the sum of cinobufagin (C₂₆H₃₄O₆) and resibufogenin (C₂₄H₃₂O₄), not less than 0.40 mg (based on a specification of 0.25g per capsule).  2. Enteric-Coating Performance Test (Key Item)  Release in Acid: In 0.1 mol/L HCl solution for 2 hours, bufadienolide release ≤10%.  Release in Buffer: After transfer to pH 6.8 phosphate buffer, release within 45 minutes ≥70%. | | |
| Compound Kushen Injection | pH Value: Should be 7.5–8.5 (Appendix VII G).  Pyrogen Test: Test as prescribed (Appendix VIII A); administer 2 ml per kg rabbit body weight. The result should meet the requirements.  Others: Should comply with all relevant requirements for injection preparations (Appendix IU). | | |
| Yangzheng Xiaoji Capsules | pH Value: Weigh 1 g of sample contents, add 20 ml water, ultrasonicate, centrifuge and collect the supernatant. The pH should be 4.5–6.0 (determined by potentiometric method, General Rule 0631).  Ignition Residue: Not more than 5.0% (ash at 700°C for 4 hours, General Rule 0841).  Hemolysis Test: Not required for non-injectable preparations.  Pyrogen Test: Not required for non-injectable preparations. | | |
| Kanglixin Capsules | pH Value: Extract the sample by mixing 1 g of contents with 20 ml water. The pH should be between 5.0 and 7.0.  Ignition Residue: Not more than 4.0%.  Abnormal Toxicity: Inject mice via tail vein with 0.5 ml of sample solution per 20 g body weight; observe for 48 hours. Survival rate should be at least 80% (according to General Rule 1141). | | |
| Lentinan | Moisture: Drying method (105°C, General Rule 0832): Not more than 8.0%  Content Uniformity: Weigh contents of 20 capsules (General Rule 0103): ±10% (labeled content: 0.25 g/capsule)  Disintegration Time: Disintegration tester (water at 37±1°C, General Rule 0921): Not more than 30 minutes  Ignition Residue: Ignite at 700°C for 4 hours (General Rule 0841): Not more than 3.0% | | |
| Weimaining Capsules | pH Value: The extract of the sample contents should have a pH between 5.5 and 6.5.  Ignition Residue: Not more than 3.5%.  Pesticide Residue (BHC/HCH): BHC (hexachlorocyclohexane) ≤ 0.1 mg/kg (determined by GC-MS, General Rule 2341). | | |
| Shenmai Injection | pH Value: Should be 5.0–6.5 (Chinese Pharmacopoeia 2010 Edition, Volume I, Appendix VIII G).  Solution Color: Accurately measure 1 ml of the product, place in a 25 ml Nessler colorimeter tube with a 10 ml marking, dilute with water to 10 ml, mix well, and compare with Yellow No. 7 Standard Color Solution (Chinese Pharmacopoeia 2010 Edition, Volume I, Appendix XIA, Method 1); the solution must not be darker.  Ignited Residue: Accurately measure 2 ml of the product, evaporate to dryness, and conduct the test as prescribed (Chinese Pharmacopoeia 2010 Edition, Volume I, Appendix XJ); not more than 1.0% (g/ml); for specifications 6 and 7, not more than 1.5% (g/ml).  Total Solids: Accurately measure 10 ml of the product, place in a previously dried evaporating dish, evaporate on a water bath to dryness, dry at 105°C for 3 hours, cool in a desiccator for 30 minutes, then promptly and accurately weigh; calculate the total solids content.  Total Solids Content: Should be 2.0%–3.5% (g/ml); for specifications 6 and 7, should be 2.5%–4.0% (g/ml).  Related Substances: Except for tannins, other related substances should comply with requirements (Chinese Pharmacopoeia 2010 Edition, Volume I, Appendix IX S).  Sterility Test: Test as prescribed; should comply with requirements (Chinese Pharmacopoeia 2010 Edition, Volume I, Appendix XIII B).  Pyrogen Test: Test as prescribed (Chinese Pharmacopoeia 2010 Edition, Volume I, Appendix XIII A); administer 2.5 ml per kg rabbit body weight, should comply with requirements.  Abnormal Toxicity: Test as prescribed (Chinese Pharmacopoeia 2010 Edition, Volume I, Appendix XVIII B, Abnormal Toxicity Test); administer by intravenous injection, should comply with requirements.  Hemolysis and Agglutination: Test as prescribed (Chinese Pharmacopoeia 2010 Edition, Volume I, Appendix XVIII B, Hemolysis and Agglutination Test); should comply with requirements.  Allergic Reaction: Test as prescribed (Chinese Pharmacopoeia 2010 Edition, Volume I, Appendix XVIII B, Allergic Reaction Test); should comply with requirements.  Osmolality: Required for specifications 6 and 7; test as prescribed (Chinese Pharmacopoeia 2010 Edition, Volume II, Appendix IX G); should be 280–320 mOsmol/kg.  Others: Should comply with all relevant requirements for injection preparations (Chinese Pharmacopoeia 2010 Edition, Volume I, Appendix IU). | | |
| Pingxiao Capsules | pH Value: The extract of the sample contents should have a pH between 4.8 and 6.0.  Ignition Residue: Not more than 6.0%.  Brucine Limit: Not more than 0.15 mg per capsule (determined by HPLC, General Rule 0512). | | |
| Xiaoaiping Injection | pH Value: 6.5–7.5 (measured directly)  Ignition Residue: Not more than 0.3%  Hemolysis Test: Negative (rabbit erythrocyte method, General Rule 1122)  Pyrogen: Rabbit method: ≤0.6 °C per rabbit (General Rule 1142)  Bacterial Endotoxin: Not more than 0.5 EU/ml (Limulus reagent method, General Rule 1143) | | |
| Kanglaite Injection | pH Value: 4.8–6.8 (measured by potentiometry to ensure stability)$8$.  Particle Size Distribution (Key Index):  Tested by laser particle size analyzer; particles ≤2 μm should account for ≥95%; no particles above 5 μm should be detected (to prevent vascular embolism)$25$.  Total Residue: 0.130–0.150 g/ml (determined by weighing after evaporation in a water bath, controls excipient residues).  2. Safety Tests  Heavy Metals: ≤5 ppm (lead equivalent, atomic absorption spectrophotometry)$8$.  Arsenic Salts: ≤2 ppm (Gutzeit method)$8$.  Bacterial Endotoxin: ≤1.5 EU/ml (Limulus Amebocyte Lysate test, ensures absence of pyrogen)$2$.  Sterility: In compliance with regulations (membrane filtration method, to prevent microbial contamination)$8$.  Ignited Residue: ≤0.1% (controls inorganic impurities). | | |
| Jinlong Capsules | Ignition Residue: Not more than 3.0%  Polypeptide Content: Not less than 5.0 mg/g (determined by Lowry method) | | |
| Huai'er Granules | pH Value: 4.0–6.0 (1 g granules dissolved in 10 ml water)  Ignition Residue: Not more than 4.5%  Solubility: Completely dissolves in hot water to form a clear solution (General Rule 0104) | | |
| Xihuang Capsules | Ignition Residue: Not more than 4.0%  Arsenic Salts: Not more than 2 ppm (Gutzeit method, General Rule 0822) | | |
| Astragalus preparations | pH Value: 4.5–6.5 (measured directly in solution)  Ignition Residue: Not more than 1.0%  Relative Density: Not less than 1.05 (General Rule 0601)  Microbial Limit: Aerobic bacteria: not more than 100 CFU/mL  Molds: not more than 10 CFU/mL (General Rule 1105)  Escherichia coli: must not be detected | | |
| Qizhen Capsule | pH Value: 5.0–6.5 (extract of contents)  Ignition Residue: Not more than 4.5%  Paclitaxel Residue: Not more than 0.001% (by HPLC-MS/MS method) | | |
| Ginseng Polysaccharide Injection | pH Value: Should be 4.0–6.0 (Chinese Pharmacopoeia 2000 Edition, Volume II, Appendix VIH).  Protein: Take 1 ml of the product, add 1 ml of 30% sulfosalicylic acid solution, mix well; no turbidity or precipitation should occur.  Abnormal Toxicity: Test the product as prescribed (Chinese Pharmacopoeia 2000 Edition, Volume II, Appendix XIC); administer by intravenous injection, and the result should meet the requirements.  Others: Should comply with all relevant requirements for injection preparations (Chinese Pharmacopoeia 2000 Edition, Volume II, Appendix IB). | | |
| Shengxue Granules | pH Value: 5.0–6.5 (extract of contents)  Ignition Residue: Not more than 4.5%  Paclitaxel Residue: Not more than 0.001% (by HPLC-MS/MS method) | | |
| Diyu Shengbai Tablets | Ignition Residue: Not more than 3.0%  Tannin Content: Not less than 20 mg/tablet (phosphomolybdic tungstic acid-casein method) | | |
| Shenlian Capsule | pH Value: 5.0–6.5 (solution after dissolution)  Ignition Residue: Not more than 4.0%  Total Matrine Alkaloids: Not less than 10 mg/bag (by HPLC method) | | |
| Compound Tianxian Capsules | pH Value: 4.0–5.5 (extract of contents)  Ignition Residue: Not more than 6.0%  Aconitine Limit: Not more than 0.01 mg/capsule (by TLC method, General Rule 0502) | | |
| Zhenqi Fuzheng Granules | pH Value: 5.5–7.0 (solution after dissolution)  Ignition Residue: Not more than 4.5%  Astragaloside IV: Not less than 1.0 mg/bag (by HPLC method) | | |
| Shenfu Injection | pH Value: 6.8–7.2 (measured directly)  Ignition Residue: Not more than 0.2%  Hemolysis Test: Negative (General Rule 1122)  Pyrogen: Rabbit method: not more than 0.6°C/rabbit  Bacterial Endotoxin: Not more than 0.25 EU/mL  Aconitum Alkaloids Limit: Ester-type alkaloids: not more than 0.01 μg/mL (by HPLC-MS/MS) | | |
| **Side effects** | | |  |
| Kangai Injection | Rash, itching, chills, fever, nausea, vomiting, chest tightness, and palpitations. Rare severe allergic reactions, such as anaphylactic shock, may also occur | | |
| Shenqi Fuzheng Injection | Allergic reactions: rash, itching, dyspnea, flushing, anaphylactic shock, etc.  Respiratory system: chest tightness, shortness of breath, cough, etc.  Skin and appendages: excessive sweating, maculopapular rash, urticaria, erythematous rash, skin redness, local skin reactions, etc.  General systemic symptoms: chills, cold sensations, shivering, fever, pain, discomfort, fatigue, chest pain, edema, etc.  Nervous and psychiatric system: dizziness, headache, suffocation, convulsions, irritability, drowsiness, etc.  Gastrointestinal system: stomatitis, dry mouth, nausea, vomiting, abdominal pain, diarrhea, abdominal distension, gastric discomfort, etc.  Cardiovascular system: palpitations, tachycardia, etc.  Administration site: phlebitis, pain at the injection site, rash, itching, numbness, etc.  A prospective post-marketing safety re-evaluation study of Shenqi Fuzheng Injection II was carried out, including 20,000 cases in the first stage and 30,000 cases in the second stage. The overall incidence of adverse drug reactions (ADRs) was 0.185% and 0.170%, respectively, with a total of 88 ADRs detected, of which 1 was a serious case (urticaria, flushing, fever). No reports of anaphylactic shock were received in the aggregate monitoring of these 50,000 post-marketing cases, but extremely rare cases of anaphylactic shock may still occur in clinical use.  Mild bleeding may occur in non-Qi deficiency patients after administration. | | |
| Compound Mylabris preparations | Not yet clear | | |
| Aidi Injection | When this product is used for the first time, some patients occasionally experience reactions such as facial flushing, urticaria, and fever. A very small number of patients may also have palpitations, chest tightness, and nausea. | | |
| Ya Dan Zi Oil Emulsion Injection | This product has no obvious toxic or adverse effects. A small number of patients may experience gastrointestinal discomfort such as a greasy sensation, nausea, and loss of appetite after administration. | | |
| Huachansu preparations | Huachansu Tablets and Capsules: Monitoring of adverse reactions and data from the literature show that this product may cause gastrointestinal adverse reactions such as nausea, vomiting, diarrhea, and abdominal distension. If there are no other serious conditions, discontinuation of the drug is not necessary; symptoms will ease or disappear with continued use. There have also been reports of local and systemic allergic reactions including rash, itching, fatigue, and fever, as well as adverse reactions such as dizziness, headache, palpitations, and arrhythmia.  Huachansu Oral Solution: Not yet clear.  Huachansu Injection: Post-marketing surveillance has found the following adverse reactions/events: a small number of patients experienced phlebitis or vascular irritation after intravenous infusion, pain, rash, itching, and local redness and swelling at the site of administration. Some individual patients also developed allergic reactions such as rash, itching, palpitations, flushing, chest tightness, dyspnea, and breathlessness; gastrointestinal reactions such as nausea, vomiting, and abdominal pain; and reports of other adverse reactions including dizziness, headache, chills, shivering, fever, and pain. | | |
| Compound Kushen Injection | Occasional symptoms such as nausea, vomiting, fever, chills, abdominal distension, and stomach discomfort may occur. Allergic reactions may also occasionally be seen, presenting as flushing and sweating of the skin on the head and neck, rash, and itching, which may be related to individual patient sensitivities. Mild local irritation may occur with topical use, but absorption is good | | |
| Yangzheng Xiaoji Capsules | Not yet clear | | |
| Kanglixin Capsules | Not yet clear | | |
| Lentinan | Not yet clear | | |
| Weimaining Capsules | Occasional gastrointestinal symptoms such as nausea may occur | | |
| Shenmai Injection | Urticarial rash, facial flushing, chest tightness, palpitations, generalized weakness, numbness, dizziness, headache, anaphylactic shock, grand mal seizure, nausea, vomiting, jaundice, gastrointestinal bleeding, acute liver and kidney function impairment, tachycardia, angina pectoris, and phlebitis. | | |
| Pingxiao Capsules | Nausea, drug rash, occasional dizziness, diarrhea | | |
| Xiaoaiping Injection | Allergic reactions: generalized skin flushing, rash, itching, dyspnea (difficulty breathing), palpitations, cyanosis, hypotension (decrease in blood pressure), laryngeal edema, anaphylactic shock, etc.  Musculoskeletal: migratory muscle pain, joint pain, etc.  Systemic reactions: fever, chills, pain, fatigue, etc.  Skin and appendages: rash, itching, excessive sweating, etc.  Digestive system: nausea, vomiting, abdominal pain, diarrhea, etc.  Respiratory system: difficulty breathing, cough, etc.  Cardiovascular system: chest tightness, palpitations, increased or decreased blood pressure, etc.  Nervous system: dizziness, headache, etc.  Others: pain at injection site, phlebitis, etc. | | |
| Kanglaite Injection | Clinical cases of fat allergy are occasionally observed, such as chills, fever, mild nausea, and reversible elevation of liver transaminases. These symptoms usually resolve spontaneously and the patient adapts after 3–5 days of use. Mild phlebitis is also occasionally seen | | |
| Jinlong Capsules | Not yet clear | | |
| Huai'er Granules | Nausea, vomiting | | |
| Xihuang Capsules | Not yet clear | | |
| Astragalus preparations | Rash, anaphylactic shock | | |
| Qizhen Capsule | Not yet clear | | |
| Ginseng Polysaccharide Injection | Local redness and swelling reaction | | |
| Shengxue Granules | Not yet clear | | |
| Diyu Shengbai Tablets | Not yet clear | | |
| Shenlian Capsule | Nausea | | |
| Compound Tianxian Capsules | Not yet clear | | |
| Zhenqi Fuzheng Granules | Not yet clear | | |
| Shenfu Injection | Allergic reactions: May manifest as itching, rash, allergic dermatitis, pale complexion, chest tightness, breathing difficulty, laryngeal edema, palpitations, cyanosis, blood pressure decrease, etc. In severe cases, anaphylactic shock may occur.  Systemic reactions: Including chills, fever, fatigue, profuse sweating, back pain, and so on.  Nervous system damage: Dizziness, headache, insomnia, tremor, convulsions, numbness of lips and limbs, etc.  Cardiovascular system: Facial flushing, palpitations, chest tightness, tachycardia, arrhythmia, fluctuating blood pressure, etc.  Digestive system damage: Nausea, vomiting, abdominal distension, abdominal pain, diarrhea, hiccups, dry mouth, gastric discomfort, abnormal liver function, etc.  Respiratory system damage: Cyanosis of lips, cough, dyspnea, rapid breathing, etc.  Urinary system damage: Urinary retention, edema, etc.  Others: Nosebleed, redness/swelling/pain at the injection site, phlebitis, visual abnormalities, etc | | |
| **Interactions** | | |  |
| Kangai Injection | When used in combination with strong electrolyte drugs (such as potassium chloride or calcium gluconate), brown-yellow precipitates may form, potentially blocking blood vessels and posing a risk of embolism.  When combined with alkaline drugs (such as omeprazole or sodium bicarbonate injection), the solution may become cloudy or produce flocculent material, which can cause severe phlebitis. | | |
| Shenqi Fuzheng Injection | Avoid mixing in the same container with Coenzyme A, Vitamin K3, Aminophylline1, and Omeprazole | | |
| Compound Mylabris preparations | Avoid concurrent use with anticoagulants like Warfarin (increased bleeding risk) and immunosuppressants like Cyclosporine | | |
| Aidi Injection | Be cautious of additive toxicities (e.g., myelosuppression, hepatorenal toxicity) when combined with chemotherapeutics like Cisplatin or Cyclophosphamide | | |
| Ya Dan Zi Oil Emulsion Injection | Avoid simultaneous use with Western medicines like Iodixanol, as it may affect efficacy | | |
| Huachansu preparations | Avoid concurrent use with cardiac glycosides like Digoxin (may cause arrhythmias) | | |
| Compound Kushen Injection | No specific interaction information was found in the search results | | |
| Yangzheng Xiaoji Capsules | No specific interaction information was found in the search results | | |
| Kanglixin Capsules | No specific interaction information was found in the search results | | |
| Lentinan | No specific interaction information was found in the search results | | |
| Weimaining Capsules | No specific interaction information was found in the search results | | |
| Shenmai Injection | Incompatible with: Veratrum nigrum, Faeces Trogopterori, Goderol injection, and antibiotics (especially penicillins) | | |
| Pingxiao Capsules | The drug insert states that interactions may occur if used with other drugs concurrently | | |
| Xiaoaiping Injection | Contraindicated to mix with other drugs in the same container | | |
| Kanglaite Injection | Not suitable for mixing with other drugs | | |
| Jinlong Capsules | The drug insert states that interactions may occur if used with other drugs concurrently | | |
| Huai'er Granules | No specific interaction information was found in the search results | | |
| Xihuang Capsules | No specific interaction information was found in the search results | | |
| Astragalus preparations | Tonifies qi and consolidates the exterior, dispels wind and eliminates pathogenic factors. Astragalus (Huangqi) is sweet and warm; it can tonify qi, reinforce defensive energy (wei qi), and consolidate the exterior, which is key for resisting wind evil. Siler (Fangfeng) is pungent, sweet, and slightly warm; its pungent and warm nature enables it to disperse, dispel wind, and release the exterior.  The two herbs share similar properties and both enter the Spleen meridian. Used together, they synergize: Astragalus with Siler consolidates the exterior without retaining pathogenic factors, while Siler with Astragalus dispels pathogens without harming the vital energy. Tonification is embedded within dispersion, and dispersion within tonification; they complement and control each other, enhancing tonification of qi, releasing the exterior, consolidating the exterior to stop sweating, with markedly increased efficacy. | | |
| Qizhen Capsule | No specific interaction information was found in the search results | | |
| Ginseng Polysaccharide Injection | Contraindicated to mix with other drugs in the same container | | |
| Shengxue Granules | No specific interaction information was found in the search results | | |
| Diyu Shengbai Tablets | No specific interaction information was found in the search results | | |
| Shenlian Capsule | Avoid concurrent use with anticoagulants like Warfarin (components like Sophora flavescens may affect coagulation, causing INR fluctuations). | | |
| Compound Tianxian Capsules | Strictly prohibited from use with TCM containing Veratrum Nigrum (Li Lu) and Faeces Trogopterori (Wu Ling Zhi) (Incompatibility per "Eighteen Antagonisms & Nineteen Incompatibilities") | | |
| Zhenqi Fuzheng Granules | No specific interaction information was found in the search results | | |
| Shenfu Injection | This product should not be directly mixed or used in combination with Coenzyme A, Vitamin K3, Aminophylline, Doxorubicin Hydrochloride, Danshen Injection, Omeprazole Sodium for Injection, or Cerebroprotein Hydrolysate Injection.  If this product needs to be used together with traditional Chinese medicines such as Pinellia, Trichosanthes Fruit, Fritillaria, Bai Lian (Bletilla), Bai Ji, Wu Ling Zhi (Trogopterus dung), or Veratrum, please consult a physician. | | |

Note: The above information is from China Pharmaceutical Information Query Platform (recognised by the State Drug Administration of China) and the National Medical Products Administration (NMPA) of China

# **Appendix 2 Search strategy**

Database: *PubMed* < December 3th 2024>

Search Strategy:

| **#** | **Searches** |
| --- | --- |
| #1 | "medicine, chinese traditional"[MeSH] |
| #2 | "medicine, east asian traditional"[MeSH] |
| #3 | "drugs, chinese herbal"[MeSH] |
| #4 | "plants, medicinal"[MeSH Terms] |
| #5 | "chinese patent medicine"[Title/Abstract] OR "chinese herbal injection"[Title/Abstract] OR "chinese medicine injection"[Title/Abstract] OR "TCM injection"[Title/Abstract] OR "TCM"[Title/Abstract] OR "chinese patent drug*"[Title/Abstract] |
| #6 | "chinese medicine*"[Title/Abstract] OR "chinese herb*"[Title/Abstract] OR "herb*"[Title/Abstract] |
| #7 | "injection*"[Title/Abstract] OR "injectable*"[Title/Abstract] OR "Oral"[Title/Abstract] OR "tablet*"[Title/Abstract] OR "granule*"[Title/Abstract] OR "pill*"[Title/Abstract] OR "capsule*"[Title/Abstract] OR "oral liquid*"[Title/Abstract] OR "solution*"[Title/Abstract] OR "powder*"[Title/Abstract] OR "Bolus"[Title/Abstract] OR "drop*"[Title/Abstract] OR "Decoction"[Title/Abstract] OR "concentrated pill*"[Title/Abstract] OR "chinese prepared medicine*"[Title/Abstract] OR "Compound"[Title/Abstract] |
| #8 | "Shenmai"[Title/Abstract] OR "Shengmai"[Title/Abstract] OR "shenqi fuzheng"[Title/Abstract] OR "Kangai"[Title/Abstract] OR "Aidi"[Title/Abstract] OR "compound cantharis"[Title/Abstract] OR "Cinobufacin"[Title/Abstract] OR "compound taxus"[Title/Abstract] OR "brucea javanica oil"[Title/Abstract] OR "Xiaoaiping"[Title/Abstract] OR "Huachansu"[Title/Abstract] OR "Jinlong"[Title/Abstract] OR "fuzheng huayu"[Title/Abstract] OR "Weifuchun"[Title/Abstract] OR "Pingxiao"[Title/Abstract] OR "Tianqi"[Title/Abstract] OR "Yangwei"[Title/Abstract] OR "Wenwei"[Title/Abstract] |
| #9 | "Jianpi"[Title/Abstract] OR "Yiqi"[Title/Abstract] OR "Xiaoji"[Title/Abstract] OR "Jiedu"[Title/Abstract] OR "Huayu"[Title/Abstract] |
| #10 | "xiangsha liujunzi"[Title/Abstract] OR "Liujunzi"[Title/Abstract] OR "Guipi"[Title/Abstract] OR "Pingwei"[Title/Abstract] OR "shenling baizhu"[Title/Abstract] OR "buzhong yiqi"[Title/Abstract] OR ("Houxiang"[All Fields] AND "Zhengqi"[Title/Abstract]) |
| #11 | "Bazhen"[Title/Abstract] OR "Shiquan Dabu"[Title/Abstract] OR "Banmao"[Title/Abstract] OR "Danggui Buxue"[Title/Abstract] OR "Shenqi Fuzheng"[Title/Abstract] OR "Weitong"[Title/Abstract] OR "Antike"[Title/Abstract] OR "Tianxian"[Title/Abstract] OR "Wannianqing"[Title/Abstract] OR "Xiangdan"[Title/Abstract] OR "Huaier"[Title/Abstract] OR "Zuojin"[Title/Abstract] OR "Ba-zhen"[Title/Abstract] |
| #12 | "Donglingcao"[Title/Abstract] OR "Chansu"[Title/Abstract] OR "Yadanzi"[Title/Abstract] OR "Xuebijing"[Title/Abstract] OR "Zhonglou"[Title/Abstract] OR "Tanreqing"[Title/Abstract] OR "Kunxian"[Title/Abstract] OR "Longdan"[Title/Abstract] OR "Chaihu"[Title/Abstract] |
| #13 | ("Hua"[All Fields] AND "chan su"[Title/Abstract]) OR "xiang dan"[Title/Abstract] OR "xiang dan"[Title/Abstract] OR "zuo jin"[Title/Abstract] OR "zuo jin"[Title/Abstract] OR "Cinobufagin"[Title/Abstract] OR "Gastrodine"[Title/Abstract] OR "Bufotoxin"[Title/Abstract] |
| #14 | 1-13/OR |
| #15 | "Randomized Controlled Trial" [Publication Type] OR "Controlled Clinical Trial"[Publication Type] OR randomized [Title/Abstract] OR randomised [Title/Abstract] OR randomly [Title/Abstract] OR random* [Title/Abstract] |
| #16 | "stomach neoplasms"[MeSH] |
| #17 | "carcin*"[Title/Abstract] OR "cancer*"[Title/Abstract] OR "neoplas*"[Title/Abstract] OR "tumour*"[Title/Abstract] OR "tumor*"[Title/Abstract] OR "cyst*"[Title/Abstract] OR "growth*"[Title/Abstract] |
| #18 | "digest*"[Title/Abstract] OR "gastr*"[Title/Abstract] OR "epigastr*"[Title/Abstract] |
| #19 | 17 AND 18 |
| #20 | 16 OR 19 |
| #21 | 14 AND 115 AND 20 |

Database: *Cochrane Library* < December 3th 2024> 10785

Search Strategy:

| **#** | **Searches** |
| --- | --- |
| #1 | MeSH descriptor: [Medicine, Chinese Traditional] explode all trees |
| #2 | MeSH descriptor: [Medicine, East Asian Traditional] explode all trees |
| #3 | MeSH descriptor: [Drugs, Chinese Herbal] explode all trees |
| #4 | MeSH descriptor: [plants, medicinal] explode all trees |
| #5 | (chinese patent medicine) OR (chinese herbal injection) OR (chinese medicine injection) OR (TCM injection) OR (TCM) OR (chinese patent drug*):ti,ab,kw |
| #6 | (chinese medicine*) OR (chinese herb*) OR (herb*):ti,ab,kw |
| #7 | (injection*) OR (injectable*) OR (Oral) OR (tablet*) OR (granule*) OR (pill*) OR (capsule*) OR (oral liquid*) OR (solution*) OR (powder*) OR (Bolus) OR (drop*) OR (Decoction) OR (concentrated pill*) OR (chinese prepared medicine*) OR (Compound):ti,ab,kw |
| #8 | (Shenmai) OR (Shengmai) OR (shenqi fuzheng) OR (Kangai) OR (Aidi) OR (compound cantharis) OR (Cinobufacin) OR (compound taxus) OR (brucea javanica oil) OR (Xiaoaiping) OR (Huachansu) OR (Jinlong) OR (fuzheng huayu) OR (Weifuchun) OR (Pingxiao) OR (Tianqi) OR (Yangwei) OR (Wenwei):ti,ab,kw |
| #9 | (Jianpi) OR (Yiqi) OR (Xiaoji) OR (Jiedu) OR (Huayu):ti,ab,kw |
| #10 | (xiangsha liujunzi) OR (Liujunzi) OR (Guipi) OR (Pingwei) OR (shenling baizhu) OR (buzhong yiqi) OR (Houxiang Zhengqi):ti,ab,kw |
| #11 | (Bazhen) OR (Shiquan Dabu) OR (Banmao) OR (Danggui Buxue) OR (Shenqi Fuzheng) OR (Weitong) OR (Antike) OR (Tianxian) OR (Wannianqing) OR (Xiangdan) OR (Huaier) OR (Zuojin) OR (Ba-zhen):ti,ab,kw |
| #12 | (Donglingcao) OR (Chansu) OR (Yadanzi) OR (Xuebijing) OR (Zhonglou) OR (Tanreqing) OR (Kunxian) OR (Longdan) OR (Chaihu):ti,ab,kw |
| #13 | (Hua chan su) OR (xiang dan) OR (xiang dan) OR (zuo jin) OR (zuo jin) OR (Cinobufagin) OR (Gastrodine) OR (Bufotoxin):ti,ab,kw |
| #14 | #1 OR #2 OR #3 OR #4 OR #5 OR #6 OR #7 OR #8 OR #9 OR #10 OR #11 OR #12 OR #13 |
| #15 | MeSH descriptor: [Stomach Neoplasms] explode all trees |
| #16 | (carcin*) OR (cancer*) OR (neoplas*) OR (tumour*) OR (tumor*) OR (cyst*) OR (growth*):ti,ab,kw |
| #17 | (digest*) OR (gastr*) OR (epigastr*):ti,ab,kw |
| #18 | #16 AND #17 |
| #19 | #15 OR #18 |
| #20 | (randomized OR randomised OR randomly OR random*):ti,ab,kw |
| #23 | #14 AND #19 AND #20 |
| #24 | Limited to trials |

Database: *Embase* < December 3th 2024> 4913

Search Strategy:

| **#** | **Searches** |
| --- | --- |
| #1 | 'chinese traditional medicine'/exp OR 'east asian traditional medicine'/exp |
| #2 | 'chinese patent medicine':ab,ti OR 'chinese herbal injection':ab,ti OR 'chinese medicine injection':ab,ti OR 'tcm injection':ab,ti OR 'tcm':ab,ti OR 'chinese patent drug*':ab,ti |
| #3 | 'chinese medicine*':ab,ti OR 'chinese herb*':ab,ti OR 'herb*':ab,ti |
| #4 | 'injection*':ab,ti OR 'injectable*':ab,ti OR 'oral':ab,ti OR 'tablet*':ab,ti OR 'granule*':ab,ti OR 'pill*':ab,ti OR 'capsule*':ab,ti OR 'oral liquid*':ab,ti OR 'solution*':ab,ti OR 'powder*':ab,ti OR 'bolus':ab,ti OR 'drop*':ab,ti OR 'decoction':ab,ti OR 'concentrated pill*':ab,ti OR 'chinese prepared medicine*':ab,ti OR 'compound':ab,ti |
| #5 | 'shenmai':ab,ti OR 'shengmai':ab,ti OR 'shenqi fuzheng':ab,ti OR 'kangai':ab,ti OR 'aidi':ab,ti OR 'compound cantharis':ab,ti OR 'cinobufacin':ab,ti OR 'compound taxus':ab,ti OR 'brucea javanica oil':ab,ti OR 'xiaoaiping':ab,ti OR 'huachansu':ab,ti OR 'jinlong':ab,ti OR 'fuzheng huayu':ab,ti OR 'weifuchun':ab,ti OR 'pingxiao':ab,ti OR 'tianqi':ab,ti OR 'yangwei':ab,ti OR 'wenwei':ab,ti |
| #6 | 'jianpi':ab,ti OR 'yiqi':ab,ti OR 'xiaoji':ab,ti OR 'jiedu':ab,ti OR 'huayu':ab,ti |
| #7 | 'xiangsha liujunzi':ab,ti OR 'liujunzi':ab,ti OR 'guipi':ab,ti OR 'pingwei':ab,ti OR 'shenling baizhu':ab,ti OR 'buzhong yiqi':ab,ti OR 'houxiang zhengqi':ab,ti |
| #8 | 'bazhen':ab,ti OR 'shiquan dabu':ab,ti OR 'banmao':ab,ti OR 'danggui buxue':ab,ti OR 'shenqi fuzheng':ab,ti OR 'weitong':ab,ti OR 'antike':ab,ti OR 'tianxian':ab,ti OR 'wannianqing':ab,ti OR 'xiangdan':ab,ti OR 'huaier':ab,ti OR 'zuojin':ab,ti OR 'ba-zhen':ab,ti |
| #9 | 'donglingcao':ab,ti OR 'chansu':ab,ti OR 'yadanzi':ab,ti OR 'xuebijing':ab,ti OR 'zhonglou':ab,ti OR 'tanreqing':ab,ti OR 'kunxian':ab,ti OR 'longdan':ab,ti OR 'chaihu':ab,ti |
| #10 | 'hua chan su':ab,ti OR 'xiang dan':ab,ti OR 'zuo jin':ab,ti OR 'cinobufagin':ab,ti OR 'gastrodine':ab,ti OR 'bufotoxin':ab,ti |
| #11 | #1 OR #2 OR #3 OR #4 OR #5 OR #6 OR #7 OR #8 OR #9 OR #10 |
| #12 | 'stomach neoplasms'/exp |
| #13 | 'carcin*':ab,ti OR 'cancer*':ab,ti OR 'neoplas*':ab,ti OR 'tumour*':ab,ti OR 'tumor*':ab,ti OR 'cyst*':ab,ti OR 'growth*':ab,ti |
| #14 | 'digest*':ab,ti OR 'gastr*':ab,ti OR 'epigastr*':ab,ti |
| #15 | #13 AND #14 |
| #16 | #12 OR #15 |
| #17 | randomized:ab,ti OR randomised:ab,ti OR randomly:ab,ti OR random*:ab,ti |
| #18 | 'randomized controlled trial':de |
| #19 | randomization:de |
| #20 | 'controlled clinical trial':de |
| #21 | #17 OR #18 OR #19 OR #20 |
| #22 | #11 AND #16 AND #21 |

CNKI December 3th 2024

| **#** | **Searches** |
| --- | --- |
| #1 | SU= "参麦注射液" + "生脉注射液" + "参芪扶正注射液" + "康艾注射液" + "艾迪注射液" + "复方斑蝥注射液" + "华蟾素注射液" + "克癌平注射液" + "复方红豆杉注射液" + "冬凌草注射液" + "野菊花注射液" + "香菇多糖注射液" + "鸦胆子油注射液" + "消癌平注射液" + "香丹注射液" + "黄芪健脾片" + "胃复春片" + "扶正化瘀片" + "消癌平片" + "参芪健胃片" + "抗癌平片" + "消瘤灵片" + "健脾益肾片" + "华蟾素片" + "平消胶囊" + "天芪胶囊" + "复方红豆杉胶囊" + "消癌平胶囊" + "益气养胃胶囊" + "健胃消积胶囊" + "扶正抗癌胶囊" + "抗癌平胶囊" + "复方斑蝥胶囊" + "胃痛胶囊" + "安康欣胶囊" + "安替可胶囊" + "复方天仙胶囊" + "复方万年青胶囊" + "莲芪胶囊" + "参芪扶正胶囊" + "香砂六君丸" + "健脾养胃丸" + "六君子丸" + "养胃丸" + "香砂养胃丸" + "归脾丸" + "正气化瘀丸" + "平胃丸" + "健脾益肠丸" + "左金丸" + "胃宁颗粒" + "胃康颗粒" + "健脾益气颗粒" + "消积颗粒" + "参苓白术散" + "香砂六君颗粒"+ "补中益气颗粒" + "参芪颗粒" + "八珍颗粒" +"槐耳颗粒" + "参芪口服液" + "复方斑蝥口服液" + "养胃口服液"+ "健脾口服液" + "消癌平口服液" + "抗癌平口服液" +"十全大补口服液" + "当归补血口服液" + "化癥回生口服液" +"注射" + "注射液" + "针剂" + "口服" + "口服液" + "合剂" + "片剂" + "片" + "胶囊" + "丸剂" + "丸" + "颗粒" + "颗粒剂" + "散剂" + "散" + "冲剂" + "软胶囊" + "滴丸" + "水丸" + "蜜丸" + "浓缩丸" |
| #2 | TKA="参麦注射液" + "生脉注射液" + "参芪扶正注射液" + "康艾注射液" + "艾迪注射液" + "复方斑蝥注射液" + "华蟾素注射液" + "克癌平注射液" + "复方红豆杉注射液" + "冬凌草注射液" + "野菊花注射液" + "香菇多糖注射液" + "鸦胆子油注射液" + "消癌平注射液" + "香丹注射液" + "黄芪健脾片" + "胃复春片" + "扶正化瘀片" + "消癌平片" + "参芪健胃片" + "抗癌平片" + "消瘤灵片" + "健脾益肾片" + "华蟾素片" + "平消胶囊" + "天芪胶囊" + "复方红豆杉胶囊" + "消癌平胶囊" + "益气养胃胶囊" + "健胃消积胶囊" + "扶正抗癌胶囊" + "抗癌平胶囊" + "复方斑蝥胶囊" + "胃痛胶囊" + "安康欣胶囊" + "安替可胶囊" + "复方天仙胶囊" + "复方万年青胶囊" + "莲芪胶囊" + "参芪扶正胶囊" + "香砂六君丸" + "健脾养胃丸" + "六君子丸" + "养胃丸" + "香砂养胃丸" + "归脾丸" + "正气化瘀丸" + "平胃丸" + "健脾益肠丸" + "左金丸" + "胃宁颗粒" + "胃康颗粒" + "健脾益气颗粒" + "消积颗粒" + "参苓白术散" + "香砂六君颗粒"+ "补中益气颗粒" + "参芪颗粒" + "八珍颗粒" +"槐耳颗粒" + "参芪口服液" + "复方斑蝥口服液" + "养胃口服液"+ "健脾口服液" + "消癌平口服液" + "抗癌平口服液" +"十全大补口服液" + "当归补血口服液" + "化癥回生口服液" +"注射" + "注射液" + "针剂" + "口服" + "口服液" + "合剂" + "片剂" + "片" + "胶囊" + "丸剂" + "丸" + "颗粒" + "颗粒剂" + "散剂" + "散" + "冲剂" + "软胶囊" + "滴丸" + "水丸" + "蜜丸" + "浓缩丸" |
| #3 | SU=”中医”+”中药” |
| #5 | #1 OR #2 OR #3 OR #4 |
| #6 | SU="胃癌" + "胃腺癌" + "胃部肿瘤" + "胃肿瘤" + "胃部恶性肿瘤" |
| #7 | TKA="胃癌" + "胃腺癌" + "胃部肿瘤" + "胃肿瘤" + "胃部恶性肿瘤" |
| #8 | #6 OR #7 |
| #9 | TKA="随机" + "对照" + "临床试验" + "临床研究" |
| #10 | SU="随机" + "对照" + "临床试验" + "临床研究" |
| #11 | #9 OR #10 |
| #12 | #5 AND #8 AND #11 |

CBM December 3th 2024

| **#** | **Searches** |
| --- | --- |
| #1 | "中药"[不加权:扩展] |
| #2 | "参麦注射液"[常用字段:智能] OR "生脉注射液"[常用字段:智能] OR "参芪扶正注射液"[常用字段:智能] OR "康艾注射液"[常用字段:智能] OR "艾迪注射液"[常用字段:智能] OR "复方斑蝥注射液"[常用字段:智能] OR "华蟾素注射液"[常用字段:智能] OR "克癌平注射液"[常用字段:智能] OR "复方红豆杉注射液"[常用字段:智能] OR "冬凌草注射液"[常用字段:智能] OR "野菊花注射液"[常用字段:智能] OR "香菇多糖注射液"[常用字段:智能] OR "鸦胆子油注射液"[常用字段:智能] OR "消癌平注射液"[常用字段:智能] OR "香丹注射液"[常用字段:智能] OR "黄芪健脾片"[常用字段:智能] OR "胃复春片"[常用字段:智能] OR "扶正化瘀片"[常用字段:智能] OR "消癌平片"[常用字段:智能] OR "参芪健胃片"[常用字段:智能] OR "抗癌平片"[常用字段:智能] OR "消瘤灵片"[常用字段:智能] OR "健脾益肾片"[常用字段:智能] OR "华蟾素片"[常用字段:智能] OR "平消胶囊"[常用字段:智能] OR "天芪胶囊"[常用字段:智能] OR "复方红豆杉胶囊"[常用字段:智能] OR "消癌平胶囊"[常用字段:智能] OR "益气养胃胶囊"[常用字段:智能] OR "健胃消积胶囊"[常用字段:智能] OR "扶正抗癌胶囊"[常用字段:智能] OR "抗癌平胶囊"[常用字段:智能] OR "复方斑蝥胶囊"[常用字段:智能] OR "胃痛胶囊"[常用字段:智能] OR "安康欣胶囊"[常用字段:智能] OR "安替可胶囊"[常用字段:智能] OR "复方天仙胶囊"[常用字段:智能] OR "复方万年青胶囊"[常用字段:智能] OR "莲芪胶囊"[常用字段:智能] OR "参芪扶正胶囊"[常用字段:智能] OR "香砂六君丸"[常用字段:智能] OR "健脾养胃丸"[常用字段:智能] OR "六君子丸"[常用字段:智能] OR "养胃丸"[常用字段:智能] OR "香砂养胃丸"[常用字段:智能] OR "归脾丸"[常用字段:智能] OR "正气化瘀丸"[常用字段:智能] OR "平胃丸"[常用字段:智能] OR "健脾益肠丸"[常用字段:智能] OR "左金丸"[常用字段:智能] OR "胃宁颗粒"[常用字段:智能] OR "胃康颗粒"[常用字段:智能] OR "健脾益气颗粒"[常用字段:智能] OR "消积颗粒"[常用字段:智能] OR "参苓白术散"[常用字段:智能] OR "香砂六君颗粒OR 补中益气颗粒"[常用字段:智能] OR "参芪颗粒"[常用字段:智能] OR "八珍颗粒 OR槐耳颗粒"[常用字段:智能] OR "参芪口服液"[常用字段:智能] OR "复方斑蝥口服液"[常用字段:智能] OR "养胃口服液OR 健脾口服液"[常用字段:智能] OR "消癌平口服液"[常用字段:智能] OR "抗癌平口服液 OR十全大补口服液"[常用字段:智能] OR "当归补血口服液"[常用字段:智能] OR "化癥回生口服液 OR注射"[常用字段:智能] OR "注射液"[常用字段:智能] OR "针剂"[常用字段:智能] OR "口服"[常用字段:智能] OR "口服液"[常用字段:智能] OR "合剂"[常用字段:智能] OR "片剂"[常用字段:智能] OR "片"[常用字段:智能] OR "胶囊"[常用字段:智能] OR "丸剂"[常用字段:智能] OR "丸"[常用字段:智能] OR "颗粒"[常用字段:智能] OR "颗粒剂"[常用字段:智能] OR "散剂"[常用字段:智能] OR "散"[常用字段:智能] OR "冲剂"[常用字段:智能] OR "软胶囊"[常用字段:智能] OR "滴丸"[常用字段:智能] OR "水丸"[常用字段:智能] OR "蜜丸"[常用字段:智能] OR "浓缩丸"[常用字段:智能] |
| #3 | #1 OR #2 |
| #5 | "胃癌"[常用字段:智能] OR "胃腺癌"[常用字段:智能] OR "胃部肿瘤"[常用字段:智能] OR "胃肿瘤"[常用字段:智能] OR "胃部恶性肿瘤"[常用字段:智能] |
| #6 | "随机"[常用字段:智能] OR "对照"[常用字段:智能] OR "临床试验"[常用字段:智能] OR "临床研究"[常用字段:智能] |
| #7 | #3 AND #5 AND #6 |

WANFANG December 3th 2024

| **#** | **Searches** |
| --- | --- |
| #1 | 主题:(胃癌 OR 胃腺癌 OR 胃部肿瘤 OR 胃肿瘤 OR 胃部恶性肿瘤) |
| #2 | 主题:(随机 OR 对照 OR 临床试验 OR 临床研究) |
| #3 | 主题:(参麦注射液 OR 生脉注射液 OR 参芪扶正注射液 OR 康艾注射液 OR 艾迪注射液 OR 复方斑蝥注射液) or 主题:(华蟾素注射液 OR 克癌平注射液 OR 复方红豆杉注射液 OR 冬凌草注射液 OR 野菊花注射液) or 主题:(香菇多糖注射液 OR 鸦胆子油注射液 OR 消癌平注射液 OR 香丹注射液 OR 黄芪健脾片 OR 胃复春片) or 主题:( 扶正化瘀片 OR 消癌平片 OR 参芪健胃片 OR 抗癌平片 OR 消瘤灵片 OR 健脾益肾片) or 主题:(华蟾素片 OR 平消胶囊 OR 天芪胶囊 OR 复方红豆杉胶囊 OR 消癌平胶囊 OR 益气养胃胶囊) or 主题:(健胃消积胶囊 OR 扶正抗癌胶囊 OR 抗癌平胶囊 OR 复方斑蝥胶囊 OR 胃痛胶囊 OR 安康欣胶囊 ) |
| #5 | 主题:(安替可胶囊 OR 复方天仙胶囊 OR 复方万年青胶囊 OR 莲芪胶囊 OR 参芪扶正胶囊 OR 香砂六君丸) or 主题:(健脾养胃丸 OR 六君子丸 OR 养胃丸 OR 香砂养胃丸 OR 归脾丸 OR 正气化瘀丸 OR 平胃丸) or 主题:(健脾益肠丸 OR 左金丸 OR 胃宁颗粒 OR 胃康颗粒 OR 健脾益气颗粒 OR 消积颗粒 OR 参苓白术散) or 主题:(香砂六君颗粒OR 补中益气颗粒 OR 参芪颗粒 OR 八珍颗粒 OR槐耳颗粒 OR 参芪口服液) or 主题:(复方斑蝥口服液 OR 养胃口服液OR 健脾口服液 OR 消癌平口服液 OR 抗癌平口服液 OR十全大补口服液) or 主题:(当归补血口服液 OR 化癥回生口服液) |
| #6 | 主题:(注射 OR 注射液 OR 针剂 OR 口服 OR 口服液 OR 合剂 OR 片剂 OR 片 OR 胶囊 OR 丸剂 OR 丸 OR 颗粒 OR 颗粒剂 OR 散剂 OR 散 OR 冲剂 OR 软胶囊 ) |
| #7 | 主题:(中成药) |
| #8 | #1-#7/OR |
| #9 | 主题:(随机 OR 对照 OR 临床试验 OR 临床研究) |
| #10 | 主题:(胃癌 OR 胃腺癌 OR 胃部肿瘤 OR 胃肿瘤 OR 胃部恶性肿瘤) |
| #11 | #8 AND #9 AND #10 |

# **Appendix 3 The definition of each outcome**

| **Outcomes** | **Definition** |
| --- | --- |
| Disease control rate | Disease control rate is defined as the proportion of patients who achieve complete response (CR), partial response (PR), or stable disease (SD) according to the Response Evaluation Criteria in Solid Tumors (RECIST) ^1^ |
| Objective response rate | Objective response rate refers to the proportion of patients who achieve either complete response (CR) or partial response (PR) based on the RECIST criteria ^1^. |
| Improvement rate in quality of life | In this study, the Improvement Rate in Quality of Life (QOL) is assessed using the Karnofsky Performance Status (KPS) score, which measures the functional status of cancer patients on a scale from 0 to 100. A higher KPS score indicates better functional status and quality of life ^2^. The QOL improvement rate is determined based on changes in KPS scores before and after treatment. It is defined as follows: QOL Improvement Rate = Proportion of patients with a KPS score increase of ≥ 10 points.  Criteria for Quality-of-Life Changes  KPS score increase ≥ 10 points → Indicates improvement in quality of life  KPS score change < 10 points → Indicates stable quality of life  KPS score decrease ≥ 10 points → Indicates deterioration in quality of life |
| Survival rate | The survival rate at 1-year and 2-year refers to the proportion of patients who remain alive at 12 months and 24 months after diagnosis or treatment initiation |
| Traditional Chinese medicine syndrome score | TCM Syndrome Score is a quantitative assessment method measured by the TCM Syndrome Scoring Scale (TCM Syndrome Scoring Scale) to objectively evaluate the severity and progression of TCM syndromes. This scoring system integrates primary symptoms, secondary symptoms, tongue diagnosis, and pulse diagnosis, assigning scores based on severity (e.g., 0 = absent, 1 = mild, 2 = moderate, 3 = severe). The total score is calculated to facilitate efficacy evaluation, disease monitoring, and research analysis. |
| CD3+T cells | The survival rate at 1-year and 2-year refers to the proportion of patients who remain alive at 12 months and 24 months after diagnosis or treatment initiation |
| CD4+T cells | CD4+ T cells, or helper T cells, regulate immune responses and promote antibody production by B cells. |
| CD8+T cells | CD8+ T cells, or cytotoxic T cells, directly kill infected or cancerous cells. |
| CD4+/CD8+ratio | The CD4+/CD8+ ratio is used as a marker of immune balance and disease progression. |
| Natural killer cells | NK cells are a type of innate immune cell involved in tumor surveillance by killing cancer cells directly. |
| CEA | CEA is a tumor marker commonly elevated in gastrointestinal cancers. |
| CA125 | CA125 is a marker primarily associated with ovarian cancer and certain gastrointestinal malignancies. |
| CA199 | CA199 is used as a marker for pancreatic and gastrointestinal cancers. |
| CA724 | CA724 is a tumor marker associated with gastric cancer. |
| Myelosuppression event | A myelosuppression adverse event is a hematologic abnormality occurring during medical treatment, regardless of whether a causal relationship with a drug or intervention has been established. It includes any decrease in leukocytes, erythrocytes, or platelets observed in a patient undergoing chemotherapy, radiotherapy, or other medical therapies ^3, 4^. |
| Gastrointestinal event | A gastrointestinal adverse event refers to any undesired gastrointestinal symptom, disorder, or complication occurring during the course of treatment, regardless of whether a causal relationship with a drug, procedure, or medical intervention has been established. It encompasses nausea, vomiting, diarrhea, constipation, abdominal pain, gastrointestinal hemorrhage, and mucosal injury observed in clinical or pharmacological studies ^3, 4^. |

**References**

1. Eisenhauer EA, Therasse P, Bogaerts J, Schwartz LH, Sargent D, Ford R, Dancey J, Arbuck S, Gwyther S, Mooney M, Rubinstein L, Shankar L, Dodd L, Kaplan R, Lacombe D, Verweij J. New response evaluation criteria in solid tumours: revised RECIST guideline (version 1.1). Eur J Cancer. 2009; 45(2):228-47.
2. Friendlander AH, Ettinger RL. Karnofsky performance status scale. Spec Care Dentist. 2009; 29(4):147-8.
3. Council for International Organizations of Medical Sciences (CIOMS) & WHO. (2016). Definition and application of terms for pharmacovigilance. Geneva: World Health Organization.
4. International Conference on Harmonisation (ICH). (2021). ICH E2A: Clinical Safety Data Management: Definitions and Standards for Expedited Reporting.

# **Appendix 4 The reference list for exclusion at the full-text screening stage**

[1] Sun Guanxing, Zhang Mengjin, Qi Zhenli. Clinical efficacy and safety analysis of Apatinib combined with chemotherapy in the treatment of advanced gastric cancer [J]. China Practical Medicine, 2024, 19 (23): 119-121. DOI:10.14163/j.cnki.11-5547/r.2024.23.032.

[2] Kuang Ziyu, Liu Kexin, Li Jie. Systematic review and Meta-analysis of Xiaoaiping injection combined with first-line chemotherapy for advanced gastric cancer [J]. Tianjin Journal of Traditional Chinese Medicine, 2024, 41 (07): 857-864.

[3] Long Wenting. Clinical observation of Jianpi Jieyu Decoction combined with chemotherapy and immunotherapy for advanced gastric cancer with liver depression and spleen deficiency syndrome [D]. Hunan University of Traditional Chinese Medicine, 2024.

[4] Zhao Lu. Clinical observation of “Fuzheng Jiedu Quyu Method” formula combined with chemotherapy maintenance therapy for elderly patients with advanced gastric cancer [D]. Tianjin University of Traditional Chinese Medicine, 2024.

[5] Luo Shuang, Song Ge, Gao Yuqian, et al. Retrospective analysis of the clinical efficacy and influencing factors of PD-1 antibody combined with first-line chemotherapy for advanced gastric cancer [J]. Cancer, 2024, 43 (03): 131-140.

[6] Li Zhishen, Zhang Yun, Shi Qinghua. Observation on the effect of Shenqi Fuzheng Injection in adjuvant treatment of cancer-related fatigue in gastric cancer patients after chemotherapy [J]. Tianjin Pharmacy Journal, 2024, 36 (01): 40-44.

[7] Zhu Shengyi, Yao Linhua, Wei Guijun. Clinical study on Weifuchun Capsules as an adjuvant chemotherapy for advanced gastric cancer with malignant ascites [J]. New Chinese Medicine, 2023, 55 (19): 150-153. DOI:10.13457/j.cnki.jncm.2023.19.029.

[8] Li Wei, Du Cheng, Zhang Jie. Efficacy of Shenqi Shiyiwei Granules combined with Docetaxel + Cisplatin chemotherapy regimen in the treatment of advanced gastric and esophageal cancer and its effects on tumor markers and immune function [J]. Modern Digestive and Interventional Therapy, 2023, 28 (09): 1129-1132.

[9] Zhu Jingjing, Zhang Xiuzhen, Qin Rui, et al. Effects of Compound Kushen Injection combined with chemotherapy on immune function, biochemical indicators, and quality of life in patients with malignant gastrointestinal tumors [J]. Medical Forum Journal, 2023, 44 (11): 11-14.

[10] Liu Ting. Network meta-analysis of six traditional Chinese medicine injections combined with chemotherapy for improving tumor treatment efficacy and immune function [D]. Liaoning University of Traditional Chinese Medicine, 2023. DOI:10.27213/d.cnki.glnzc.2023.000643.

[11] Sun Mingyu. Study on the clinical efficacy and mechanism of Jiawei Banxia Xiexin Decoction combined with chemotherapy in the treatment of advanced gastric cancer. Shanghai Shuguang Hospital Affiliated to Shanghai University of Traditional Chinese Medicine, 2023-02-01.

[12] Ma Min, Wei Xihua. Effects of Astragalus Granules combined with FOLFOX chemotherapy regimen in the treatment of elderly gastric cancer and its impact on immune function [J]. Chinese Journal of Gerontology, 2022, 42 (21): 5209-5212.

[13] Wang Weijing, Song Heyong, Li Yonglan. Clinical evaluation of Liuwei Dihuang Pills combined with Capecitabine and Cisplatin in the treatment of advanced gastric cancer [J]. China Pharmaceuticals, 2022, 31 (18): 94-96.

[14] Shen Danping, Zheng Zhongxian, Cheng Liang, et al. Clinical study of Brucea Javanica Oil Emulsion Injection combined with Tegafur and Apatinib in the treatment of advanced gastric cancer [J]. Journal of Clinical and Experimental Medicine, 2022, 21 (09): 945-948.

[15] Huang Chen. Observation and mechanism study of Compound Kushen Injection combined with Paclitaxel in the treatment of diffuse gastric cancer [D]. Zhengzhou University, 2022. DOI:10.27466/d.cnki.gzzdu.2022.006195.

[16] Ge Yutong, Wei Xiaowei. Clinical observation of Diyu Shengbai Tablets in improving neutropenia in elderly patients with advanced gastric cancer undergoing oral chemotherapy [J]. Central South Pharmacy, 2022, 20 (01): 214-218.

[17] Zhang Guorong, Zheng Juan, Zhang Lanying. Clinical study on traditional Chinese medicine combined with chemotherapy in the treatment of advanced gastric cancer [J]. China Community Doctors, 2021, 37 (29): 101-102.

[18] Fu Ling. Study on the clinical efficacy of integrating traditional Chinese medicine and western medicine in the treatment of gastric cancer and its effect on tumor markers, immune function, and quality of life [J]. China Community Doctors, 2021, 37 (26): 106-107.

[19] Zhou Wei. Post-marketing evaluation of Compound Kushen Injection for gastric cancer and esophageal cancer based on big data integration [D]. Beijing University of Chinese Medicine, 2021. DOI:10.26973/d.cnki.gbjzu.2021.000717.

[20] Su He, Li Zhigang, Wang Yiyao. Clinical study on Compound Kushen Injection combined with Lobaplatin intraperitoneal perfusion for malignant ascites caused by gastric cancer [J]. Liaoning Journal of Traditional Chinese Medicine, 2021, 48 (02): 124-125. DOI:10.13192/j.issn.1000-1719.2021.02.035.

[21] Liu Dengxiang, Wang Na, He Lili, et al. Clinical study on Kanglaite Injection combined with Apatinib in the treatment of advanced gastric cancer [J]. Journal of North Sichuan Medical College, 2021, 36 (01): 38-41.

[22] Yin Bo, Cheng Xiangjie, Yuan Quanliang. Observation on the efficacy of Kanglaite Injection combined with Apatinib in patients with advanced gastric cancer after failure of second-line therapy [J]. Huaihai Medicine, 2021, 39 (01): 63-66. DOI:10.14126/j.cnki.1008-7044.2021.01.020.

[23] Wang Lin, Cao Fang, Zhang Yanbing, et al. Study on the effect of Kanglaite Injection combined with intraperitoneal hyperthermic perfusion chemotherapy using S-1 and Paclitaxel on serum inflammatory factors and survival in patients with advanced gastric cancer [J]. Drug Evaluation Research, 2020, 43 (12): 2485-2488.

[24] Zhang Mingming, Meng Cuiqiao, Gao Wei, et al. The effect and significance of Astragalus Polysaccharides on the nutritional status of patients with gastric cancer undergoing adjuvant chemotherapy after surgery [J]. Hebei Medicine, 2020, 42 (22): 3383-3387.

[25] Tian Yuan, Yang Peigang, Tan Bibo, et al. Efficacy of Bufalin Injection combined with Apatinib in treating advanced gastric cancer [J]. Practical Journal of Medicine, 2020, 36 (18): 2583-2586.

[26] Wu Jie, Huang Hongyan, Wang Xiaolin, et al. Clinical efficacy of radiofrequency ablation combined with chemotherapy in the treatment of advanced gastric cancer with liver metastasis and its impact on immune function and quality of life [J]. Cancer Progress, 2020, 18 (16): 1696-1699.

[27] Jin Guihong, Li Benquan, Ma Chaofeng. Clinical efficacy and safety of Apatinib combined with chemotherapy in patients with advanced gastric cancer who have failed multiple treatment lines [J]. Clinical Research, 2020, 28 (07): 1-2.

[28] Chen Fayi. Effect of Astragalus Injection combined with chemotherapy on immune function and quality of life in patients with advanced gastric cancer [J]. Journal of Cancer Control and Treatment, 2020, 33 (04): 557-560.

[29] Zhao Lei, Liu Shunxin, Gao Ronghui, et al. Clinical efficacy and safety of Apatinib combined with Capecitabine in the treatment of advanced gastric cancer [J]. Journal of Modern Oncology, 2020, 28 (02): 200-204.

[30] Zhang Wenqian, Zhao Xiaojie, Zhang Bin, et al. Clinical study of Aidi Injection combined with neoadjuvant chemotherapy for advanced gastric cancer [J]. Practical Clinical Journal of Integrated Traditional Chinese Medicine and Western Medicine, 2020, 20 (01): 65-67. DOI:10.13288/j.11-5638/r.2020.01.019.

[31] Liu Shumei, Li Shengli. Study on the effect of Aidi Injection combined with chemotherapy on immune function and quality of life in patients with advanced gastric cancer [J]. Chinese Journal of Clinical Rational Drug Use, 2020, 13 (35): 122-124.

[32] Zhang Yumei. Effects of Apatinib combined with S-1 and Oxaliplatin regimen in the treatment of advanced gastric cancer and its impact on oxidative stress and inflammatory factor levels [J]. World Latest Medical Information Abstracts, 2020, 20 (07): 32-33.

[33] Wu Xiaomeng. Clinical efficacy of Cinobufacini Injection combined with chemotherapy in the treatment of advanced gastric cancer and its effect on serum tumor markers and immune function [J]. Chinese Journal of Clinical Rational Drug Use, 2020, 13 (36): 54-56.

[34] Song Yang, Yuan Yueqiu. Study on the effect of Aidi Injection combined with Oxaliplatin chemotherapy in patients with advanced gastric cancer [J]. Chinese and Foreign Medical Research, 2020, 18 (09): 78-79.

[35] Zhang Zhengxin, Wang Wenying, Liu Xia. Analysis of the effect of Baihua She Shecao (Hedyotis Diffusa) Injection combined with FOLFOX chemotherapy regimen on the immune function of patients with advanced gastric cancer [J]. Shandong Journal of Traditional Chinese Medicine, 2019, 38 (12): 1152-1155.

[36] Zhang Peihua. Clinical analysis of Shenfu Injection combined with chemotherapy in the treatment of advanced gastric cancer [J]. Journal of New Chinese Medicine, 2019, 51 (22): 151-153. DOI:10.13457/j.cnki.jncm.2019.22.041.

[37] Wang Juncheng, Li Yifan, Yang Jun, et al. The role of Shenmai Injection in mitigating chemotherapy-induced leukopenia in patients with advanced gastric cancer [J]. Modern Chinese Medicine, 2019, 21 (10): 68-72.

[38] Zhou Yanchun, Li Xiaofeng. Effect of Kanglaite Injection combined with docetaxel-based chemotherapy on the quality of life and immune function in patients with advanced gastric cancer [J]. Modern Journal of Integrated Traditional Chinese and Western Medicine, 2019, 28 (23): 2634-2637.

[39] Zhao Jing, Liu Haifeng, Xiao Fang, et al. Clinical evaluation of Apatinib combined with S-1 in the second-line treatment of advanced gastric cancer [J]. Journal of Cancer Medicine, 2019, 8 (06): 871-875.

[40] Yang Qing, Wu Yongming, Yang Yi, et al. Clinical exploration of Weifuchun Capsules combined with SOX chemotherapy in advanced gastric cancer [J]. Hainan Medical Journal, 2019, 30 (20): 2511-2513. DOI:10.3969/j.issn.1003-6350.2019.20.020.

[41] Dong Qian, Zhao Xiaolu, Lu Yanjin, et al. Observation on the analgesic effect of Celastrol in advanced gastric cancer patients with chronic cancer pain [J]. Journal of Integrative Medicine, 2019, 45 (07): 1541-1545.

[42] Shao Yanfei, Zhang Lingling, Fu Jian. Effect of Compound Kushen Injection on postoperative immune function and prognosis in gastric cancer patients [J]. Traditional Chinese Drug Research & Clinical Pharmacology, 2019, 30 (05): 639-644.

[43] Wang Xiaotian, Meng Qingyun, Bai Zhonghua, et al. Clinical study on the efficacy of Aidi Injection combined with hyperthermic intraperitoneal chemotherapy in gastric cancer patients with malignant ascites [J]. Tianjin Journal of Traditional Chinese Medicine, 2019, 36 (04): 380-384.

[44] Yang Wenlong, Guo Haiyan, Zhao Xiang, et al. Effect of Kanglaite Injection combined with palliative chemotherapy on immune function and serum tumor markers in advanced gastric cancer [J]. Guangming Journal of Chinese Medicine, 2019, 34 (07): 1088-1091.

[45] Zhao Jianbin, Li Yan, Zhang Fan. Comparative study of Xiaoaiping Injection and Shenfu Injection in the treatment of advanced gastric cancer [J]. Liaoning Journal of Traditional Chinese Medicine, 2019, 46 (10): 2144-2147.

[46] He Min, Wu Huashan, Shi Peiwen, et al. Observation of the clinical efficacy of Shenqi Fuzheng Injection combined with chemotherapy in the treatment of elderly gastric cancer [J]. Shandong Medical Journal, 2019, 40 (04): 30-33.

[47] Liu Jun, Zhao Wenming, Li Zhen, et al. Clinical characteristics of gastric cancer patients responding to traditional Chinese medicine combined with chemotherapy [J]. Cancer Journal, 2019, 38 (03): 193-199.

[48] Zhang Lili, Chen Zhenning, Wang Bo, et al. Study on the combined effect of Apatinib and FOLFOX regimen chemotherapy on survival outcomes of gastric cancer patients [J]. Journal of Cancer Control and Treatment, 2018, 31 (08): 1214-1218.

[49] Xie Xiaoyu, Yang Feng, Wu Jianhua, et al. Comparative analysis of Shenqi Fuzheng Injection combined with XELOX chemotherapy regimen for advanced gastric cancer with deficiency of Qi and blood [J]. Journal of Guangzhou University of Traditional Chinese Medicine, 2018, 35 (04): 599-602.

[50] Wu Wei, Zhang Hongwei, Ma Qiang, et al. Effect of Shenmai Injection on immune regulation and chemotherapy tolerance in elderly patients with advanced gastric cancer [J]. Chinese Journal of New Drugs and Clinical Remedies, 2018, 37 (12): 673-678.

[51] Zhang Jian, Wang Yonghua, Liu Zhili, et al. Clinical efficacy observation of Shenfu Injection combined with chemotherapy in the treatment of elderly patients with advanced gastric cancer [J]. Chinese Journal of Clinical Rational Drug Use, 2018, 11 (34): 69-71.

[52] Li Qiang. Clinical efficacy of Shenmai Injection combined with chemotherapy in advanced gastric cancer and its impact on immune function [J]. Modern Diagnosis and Therapy, 2018, 29 (20): 3083-3085.

[53] Wang Xiaorong, Zhang Yunjia, Dou Yue, et al. Observation of the clinical efficacy of Aidi Injection combined with SOX chemotherapy regimen in advanced gastric cancer [J]. Cancer Progress, 2018, 16 (14): 1739-1741.

[54] Liu Hong, Yu Huaiyuan, He Xiaoke, et al. The clinical application value of Kanglaite Injection combined with Interferon-α 2b and Capecitabine in the treatment of advanced gastric cancer [J]. Journal of China Prescription Drug, 2018, 16 (09): 66-69.

[55] Chen Wei. Effect of Shenfu Injection combined with chemotherapy on prognosis-related indicators of patients with advanced gastric cancer [J]. Journal of Practical Cancer, 2018, 33 (05): 680-683.

[56] Zhao Fang, Li Hongling, Wang Ming, et al. Clinical comparative study of Shenqi Fuzheng Injection combined with chemotherapy for gastric cancer [J]. Modern Chinese Medicine, 2018, 20 (07): 1341-1345.

[57] Yu Zhengfei, Wang Zhimin, Liu Qiang, et al. Effect of Apatinib combined with FOLFOX chemotherapy regimen on serum VEGF and immune function in advanced gastric cancer patients [J]. Biomedical Engineering & Clinical Medicine, 2018, 22 (03): 263-267.

[58] Zhang Ting, Li Zhenfeng, Huang Qingxin, et al. Meta-analysis of Aidi Injection combined with chemotherapy in the treatment of advanced gastric cancer [J]. China Pharmacy, 2018, 29 (08): 1079-1084.

[59] Deng Xiaoxue, Zhu Bo, Zou Shengzhi, et al. Comparative study of Cinobufacini Injection combined with chemotherapy versus chemotherapy alone for advanced gastric cancer [J]. Journal of China Pharmaceutical University, 2018, 49 (06): 747-752.

[60] Gao Wei, Zhang Rongxin, Zhao Jinbo, et al. Clinical study on Aidi Injection combined with Capecitabine in palliative treatment of advanced gastric cancer [J]. Tianjin Journal of Traditional Chinese Medicine, 2018, 35 (04): 319-322.

[61] Zhou Xiaoping, Chen Guangxian, Xu Xiaojun, et al. Study on the effect of Astragalus Polysaccharides on the chemotherapy toxicity and immune function in patients with advanced gastric cancer [J]. Chinese Journal of Clinical Oncology and Rehabilitation, 2018, 25 (06): 684-687.

[62] Lin Ming, Wang Haifeng, Sun Liqun, et al. Clinical study of Aidi Injection combined with albumin-paclitaxel in the treatment of advanced gastric cancer [J]. World Journal of Integrated Traditional and Western Medicine, 2018, 13 (04): 566-569.

[63] Hou Xueying, Wang Xiaoqiang, Meng Qingyun, et al. Evaluation of Kanglaite Injection combined with S-1 and Oxaliplatin chemotherapy regimen in the treatment of advanced gastric cancer [J]. Modern Diagnosis and Therapy, 2018, 29 (12): 1864-1867.

[64] Wang Lifang, He Qing, Zhong Bo, et al. Clinical study on the efficacy of Aidi Injection combined with FOLFOX chemotherapy regimen in the treatment of advanced gastric cancer [J]. Modern Chinese Medicine Research & Practice, 2018, 32 (03): 17-21.

[65] Gao Xue, Li Hongyu, Xu Zheng, et al. Study on the effect of Aidi Injection combined with chemotherapy on immune function and survival rate in advanced gastric cancer [J]. Shaanxi Journal of Traditional Chinese Medicine, 2018, 39 (02): 160-162.

[66] Zhang Zhenyu, Liu Wenjian, Wang Qing, et al. Clinical efficacy of Shenmai Injection combined with XELOX regimen in the treatment of advanced gastric cancer [J]. New Drugs and Clinical Remedies, 2018, 37 (01): 73-77.

[67] Song Lili, Meng Ling, Zhou Yuan, et al. Clinical study on Apatinib combined with S-1 therapy in advanced gastric cancer [J]. Practical Journal of Cancer, 2018, 33 (01): 151-154.

[68] Sun Xuelian, Fang Hairong, Xu Jianhua, et al. Clinical observation of Xiaoaiping Injection combined with chemotherapy in the treatment of advanced gastric cancer [J]. Journal of Oncology, 2018, 24 (04): 287-291.

[69] Shen Haihong, Cheng Xuyan, Liu Hui, et al. Meta-analysis of combined traditional Chinese medicine therapy and chemotherapy for advanced gastric cancer [J]. Chinese Journal of Practical Internal Medicine, 2018, 38 (03): 249-254.

[70] Zhu Xiaojian, Yang Jianjun, Wang Wei, et al. Clinical study on the efficacy of Kanglaite Injection combined with FOLFOX4 regimen in the treatment of advanced gastric cancer [J]. Journal of China Prescription Drug, 2018, 16 (01): 84-87.

[71] Liang Jun, Xu Qianqian, Fan Xiaoling, et al. Observation of the efficacy of Brucea Javanica Oil Emulsion Injection combined with chemotherapy in patients with postoperative recurrence of gastric cancer [J]. Modern Medical Journal of China, 2018, 40 (01): 115-118.

[72] He Jun, Zhang Xiaojun, Zhang Xinhui, et al. The impact of Kanglaite Injection combined with chemotherapy on immune function in advanced gastric cancer [J]. New Chinese Medicine, 2018, 50 (01): 66-69.

[73] Li Lihua, Luo Wei, Xie Junfeng, et al. Clinical study on the effect of Kanglaite Injection combined with XELOX chemotherapy regimen in advanced gastric cancer [J]. Guangxi Medical Journal, 2018, 40 (01): 50-52.

[74] Wang Hui, Zhang Mei, Zhang Jiayi, et al. Clinical observation of the efficacy of Shenfuzheng Injection combined with neoadjuvant chemotherapy in advanced gastric cancer [J]. World Chinese Medicine, 2018, 13 (01): 160-164.

[75] Zhou Wenxuan, Huang Yu, Wang Yu, et al. Clinical study on Aidi Injection combined with Capecitabine in the treatment of elderly patients with advanced gastric cancer [J]. China Journal of Cancer Prevention and Treatment, 2018, 25 (01): 51-54.

[76] Ma Xiaoyan, Tan Jihong, Li Jun, et al. Effect of Shenmai Injection combined with chemotherapy on immune function in elderly patients with advanced gastric cancer [J]. Journal of Gansu University of Chinese Medicine, 2018, 35 (06): 45-48.

[77] Wu Dong, Wu Guihua, Li Wen, et al. Clinical study on Apatinib combined with SOX chemotherapy regimen in patients with advanced gastric cancer [J]. Journal of Modern Oncology, 2018, 26 (24): 4034-4038.

[78] Zhang Xin, Yang Qing, Fu Yulong, et al. Effect of Shenfu Injection combined with chemotherapy on immune function and survival in patients with advanced gastric cancer [J]. Journal of Traditional Chinese Medicine, 2018, 59 (01): 57-60.

[79] Liu Yang, Xu Minghua, Zhang Huaming, et al. Clinical observation of Shenqi Fuzheng Injection combined with chemotherapy in patients with advanced gastric cancer with blood deficiency syndrome [J]. Herald of Medicine, 2018, 37 (01): 99-103.

[80] Li Jie, Wang Lin, Sun Jianbo, et al. Effect of Astragalus Polysaccharides on chemotherapy-induced myelosuppression and immune function in patients with advanced gastric cancer [J]. Journal of Clinical Research, 2018, 35 (01): 122-124.

[81] Wang Kun, Zhang Zhiqiang, Zhao Tengfei, et al. Clinical study of Shenfu Injection combined with paclitaxel chemotherapy regimen in advanced gastric cancer [J]. Journal of Modern Oncology, 2018, 26 (02): 281-285.

[82] Zhang Rui, Li Biao, Wu Xiaoming, et al. The effect of Kanglaite Injection combined with chemotherapy on quality of life and immune function in elderly patients with advanced gastric cancer [J]. Chinese Journal of Clinical Oncology, 2018, 45 (02): 64-67.

[83] Yang Xiaofang, Ma Qin, Du Xi, et al. Clinical observation of Aidi Injection combined with chemotherapy in patients with advanced gastric cancer [J]. Chinese Journal of Integrative Medicine on Cardio/Cerebrovascular Disease, 2018, 16 (02): 254-257.

[84] Zhao Lina, Wang Jun, Li Zhihua, et al. Effect of Bufalin Injection combined with chemotherapy on T-lymphocyte subsets in patients with advanced gastric cancer [J]. Chinese Journal of New Drugs, 2018, 27 (02): 183-187.

[85] Chen Xue, Zhao Nan, Li Jianning, et al. Clinical efficacy observation of Kanglaite Injection combined with XELOX chemotherapy regimen for advanced gastric cancer [J]. China Pharmacy, 2018, 29 (06): 756-759.

[86] Li Ming, Hu Wensheng, Wang Yizhong, et al. Effect of Aidi Injection combined with chemotherapy on serum IL-6 and TNF-α levels in patients with advanced gastric cancer [J]. Chinese Journal of Clinical Oncology, 2018, 45 (01): 31-34.

[87] Wang Zhaoxiang, Li Zhengming, Liu Bing, et al. Clinical study on Shenqi Fuzheng Injection combined with chemotherapy for improving immunity in advanced gastric cancer patients [J]. Chinese Journal of Cancer Prevention and Treatment, 2018, 25 (01): 27-30.

[88] Liu Xincheng, Zhao Wei, Han Xiaobo, et al. Effect of Brucea Javanica Oil Emulsion Injection combined with chemotherapy on quality of life and adverse reactions in patients with advanced gastric cancer [J]. Journal of Cancer Control and Treatment, 2018, 31 (01): 56-59.

[89] Xu Feng, Zhang Bing, Guo Hao, et al. Effect of Shenfu Injection combined with chemotherapy on serum tumor markers and immune function in patients with advanced gastric cancer [J]. Medical Journal of Wuhan University, 2018, 39 (01): 125-129.

[90] Zhu Xiaojian, Yang Jianjun, Wang Wei, et al. Study on the efficacy of Kanglaite Injection combined with chemotherapy in the treatment of advanced gastric cancer [J]. Modern Medical Journal, 2018, 46 (01): 37-41.

[91] Miao Xiaoling, Han Yu, Kong Ming. Clinical observation of maintenance chemotherapy combined with Qishen Hewei Formula in the treatment of gastric cancer [J]. Journal of Practical Traditional Chinese Medicine, 2024, 40(11): 2193-2195.

[92] Sha Shanyan, Li Siyu, Zhu Wei, et al. Clinical observation of 34 cases of leukopenia in breast cancer patients caused by anthracycline chemotherapy, treated with Wen Shen Sheng Bai Formula of traditional Chinese medicine combined with moxibustion [J]. Journal of Traditional Chinese Medicine, 2023, 64(05): 482-489. DOI:10.13288/j.11-2166/r.2023.05.010.

[93] Song Heping. Clinical observational study on Jianpi therapy combined with chemotherapy for advanced gastric cancer [D]. Shanghai University of Traditional Chinese Medicine, 2020. DOI:10.27320/d.cnki.gszyu.2020.000847.

[94] Sun Xin. Clinical observation of chemotherapy combined with Sijunzi Decoction and Shixiao Powder in the treatment of mid-stage gastric cancer [J]. Journal of Practical Traditional Chinese Medicine, 2019, 35(09): 1100-1101.

[95] Yu Jingtao. Clinical observation of chemotherapy combined with traditional Chinese medicine in the treatment of 45 cases of middle and advanced gastric cancer [J]. Chinese Ethnic and Folk Medicine, 2019, 28(08): 110-111+114.

[96] Jiang Xiaojun. Clinical observation of chemotherapy combined with traditional Chinese medicine for 134 cases of middle and advanced gastric cancer [J]. Chinese Ethnic and Folk Medicine, 2018, 27(12): 107-111.

[97] Zhang Haiying, Gao Xiyuan. Clinical observation of acupuncture point application combined with massage in treating chemotherapy-induced vomiting in 40 cases [J]. Guidance of Traditional Chinese Medicine, 2015, 21(19): 51-53. DOI:10.13862/j.cnki.cn43-1446/r.2015.19.019.

[98] Feng Xin. Clinical observation of traditional Chinese medicine combined with DCF chemotherapy regimen in the treatment of advanced gastric cancer [J]. World Latest Medical Information Abstracts, 2015, 15(54): 82+97.

[99] Wen Jie. Preliminary clinical observation of Shenqi Fuzheng Injection combined with chemotherapy in the treatment of advanced gastric cancer [D]. Yangzhou University, 2014.

[100] Wang Li, Wang Jirong, Wang Keming, et al. Clinical observation of the immunomodulatory effect of lentinan injection in gastric cancer treatment [J]. Hainan Medical Journal, 2013, 24(24): 3610-3612.

[101] You Long, Qiao Shuai, Li Chaosun, et al. Intraperitoneal hyperthermic hypotonic perfusion therapy with traditional Chinese medicine and chemotherapy drugs for 58 cases of gastric cancer [J]. Chinese Journal of Integrated Traditional and Western Medicine in Surgery, 2013, 19(05): 544-546.

[102] Yang Feiyue, Li Libo, Jin Shan. Efficacy observation of chemotherapy combined with Kang'ai Injection in the treatment of malignant tumors in 83 cases [J]. Shandong Medical Journal, 2009, 49(21): 73-74.

[103] Guo Jian, Liu Qin. Clinical observation of traditional Chinese medicine in the intermittent period of chemotherapy for gastric cancer [J]. Modern Distance Education in Chinese Medicine, 2008, (11): 1345-1346.

[104] Chang Yongfang, Tang Aiming, Wang Jiaofeng, et al. Clinical observation of lentinan-assisted therapy in elderly patients with advanced gastric cancer and colorectal cancer [J]. Modern Journal of Integrated Traditional Chinese and Western Medicine, 2008, (28): 4375-4376+4383.

[105] Ji Weiguo. Clinical observation of Kang'ai Injection combined with chemotherapy in the treatment of malignant tumors [J]. Jilin Journal of Traditional Chinese Medicine, 2007, (07): 26. DOI:10.13463/j.cnki.jlzyy.2007.07.018.

[106] Li Rong. Clinical observation of Yanshu Injection combined with chemotherapy in the treatment of middle and advanced gastric cancer [J]. Chinese Community Doctor (Comprehensive Edition), 2006, (16): 60.

[107] Xu Zhenguo. Clinical observation of traditional Chinese medicine combined with intraperitoneal chemotherapy in the treatment of middle and advanced gastric cancer (69 cases) Proceedings of the 10th National Conference on Integrated Traditional and Western Medicine Oncology, Chinese Association of Integrated Traditional and Western Medicine. Xuzhou, Jiangsu Pei County Traditional Chinese Medicine Hospital, 2006: 285-287.

[108] Han Yuhua, Si Pengxian. Clinical observation of 30 cases of postoperative chemotherapy combined with traditional Chinese medicine in the treatment of advanced gastric cancer [J]. Shanxi Journal of Traditional Chinese Medicine, 2005, (02): 13.

[109] Xu Zhenguo, Liu Jiasheng, Zhang Liguang. Clinical study of traditional Chinese medicine combined with intraperitoneal chemotherapy in the treatment of advanced gastric cancer (69 cases) [J]. China Medical Guide, 2005, (04): 94-96.

[110] Song Jiaju. Study on the anti-metastatic effect of Jianpi traditional Chinese medicine in postoperative gastric cancer treatment [J]. Modern Journal of Integrated Traditional Chinese and Western Medicine, 2004, (22): 2956-2957.

[111] Wei Peng, Wang Jingjing. Clinical study of Qizhen Capsules combined with Apatinib in the treatment of advanced gastric cancer after failure of second-line or above treatments [J]. Modern Drugs & Clinics, 2024, 39(11): 2888-2891.

[112] Nadi Re·Wuer, Li Nan, Zhang Hongliang. Study on the mechanism of Apatinib combined with Shenyi Capsule in inhibiting VEGFR-2 overexpressing gastric adenocarcinoma cell SGC-7901 proliferation [J]. Evidence-Based Medicine, 2023, 23(05): 291-299.

[113] Nadi Re·Wuer. Study on the mechanism of Apatinib combined with Shenyi Capsule in inhibiting VEGFR-2 overexpressing gastric adenocarcinoma cell SGC-7901 proliferation [D]. Xinjiang Medical University, 2023. DOI:10.27433/d.cnki.gxyku.2023.001297.

[114] Tan Linlin. Clinical observation of Erteng Sanjie Capsule combined with Apatinib in the treatment of advanced gastric cancer (Spleen Deficiency with Phlegm and Blood Stasis type) [D]. Shanxi Academy of Traditional Chinese Medicine, 2022. DOI:10.27286/d.cnki.gsxzy.2022.000006.

[115] Xia Jin. Effects of Lentinan combined with chemotherapy on serum IL-2, IL-6, and immune function in elderly patients with gastric cancer [J]. Journal of Medical Forum, 2021, 42(05): 111-114.

[116] Liu Dengxiang, Wang Na, He Lili, et al. Clinical study of Kanglaite Injection combined with Apatinib in the treatment of advanced gastric cancer [J]. Journal of North Sichuan Medical College, 2021, 36(01): 38-41.

[117] Shi Shuai. Clinical efficacy analysis of Shenyi Capsule combined with Apatinib in the treatment of advanced gastric cancer [J]. Smart Healthcare, 2019, 5(14): 82-83. DOI:10.19335/j.cnki.2096-1219.2019.14.033.

[118] Ma Chunlei. Discussion on the clinical value of integrated traditional Chinese and Western medicine treatment for middle and advanced gastric cancer [J]. China Modern Drug Application, 2019, 13(06): 142-143. DOI:10.14164/j.cnki.cn11-5581/r.2019.06.087.

[119] Zhang Xiaosan, Zhang Yiming, Zhao Yan, et al. Clinical study of Shenyi Capsule combined with Apatinib in the treatment of advanced gastric cancer [J]. Modern Drugs & Clinics, 2019, 34(02): 411-414.

[120] Zhou Xiaofang, Huang Xiaolong. Effects of Shenqi Fuzheng Injection combined with chemotherapy on immune function and serum tumor markers in patients with advanced lung cancer [J]. Chinese Journal of Health Laboratory Technology, 2018, 28(11): 1344-1346.

[121] Wang Qichuan, Wang Qing, Qu Zhongyu, et al. Effects of lentinan combined with chemotherapy on serum IL-2, IL-6, and immune function in elderly patients with gastric cancer [J]. Chinese Journal of Gerontology, 2018, 38(07): 1609-1612.

[122] Cai Zhaodi. Effects of Shenling Baizhu Powder on postoperative chemotherapy quality of life and CD3+, CD4+ levels in gastric cancer patients [J]. Chinese Journal of Integrated Traditional and Western Medicine on Digestion, 2017, 25(09): 675-677.

[123] Li Junchao. Clinical observation of integrated traditional Chinese and Western medicine in the treatment of middle and advanced malignant tumors [J]. Inner Mongolia Medical Journal, 2017, 49(08): 962-963. DOI:10.16096/J.cnki.nmgyxzz.2017.49.08.027.

[124] Yu Zhibin, Huang Xi, Chen Xuanying, et al. Effects of Shenqi Fuzheng Injection on serum tumor markers in patients with advanced gastric cancer [J]. Jiangxi Medical Journal, 2017, 52(08): 722-725.

[125] Zhang Jianjun, Ou Chang, Wu Sheng, et al. Clinical observation of Anwei Tang combined with chemotherapy in the treatment of middle and advanced gastric cancer [J]. Hebei Journal of Traditional Chinese Medicine, 2017, 39(07): 1061-1063+1068.

[126] Wang Zhifeng, Geng Xianhui. Report on 64 cases of middle and advanced gastric cancer treated with integrated traditional Chinese and Western medicine [J]. Aerospace Medicine Journal, 2017, 28(06): 691-693.

[127] Jiang Lai, Zhou Qinfei, Qiu Pengnian, et al. Clinical observation of integrated traditional Chinese and Western medicine in the treatment of middle and advanced colorectal cancer [J]. New Chinese Medicine, 2017, 49(04): 119-121. DOI:10.13457/j.cnki.jncm.2017.04.042.

[128] Tie Chengjiu. Discussion on the clinical efficacy of integrated traditional Chinese and Western medicine in the treatment of middle and advanced gastric cancer [J]. World Latest Medicine Information Abstracts, 2017, 17(18): 116.

[129] Huang Yajun, Peng Xueli, Guo Xiaomeng. Integrated traditional Chinese and Western medicine treatment of 42 cases of middle and advanced gastric cancer [J]. Modern Distance Education of Chinese Medicine, 2017, 15(04): 101-102.

[130] Jiang Xiaofen, Shao Chuanfeng. Clinical observation of FMC chemotherapy regimen combined with Yiqi Jianpi Huoxue Tang in the treatment of middle and advanced gastric cancer [J]. New Chinese Medicine, 2017, 49(02): 124-126. DOI:10.13457/j.cnki.jncm.2017.02.043.

[131] Hu Pei. Effects of Shenqi Fuzheng Injection on immune function in patients with advanced gastric cancer undergoing chemotherapy [J]. Modern Journal of Integrated Traditional Chinese and Western Medicine, 2017, 26(03): 292-294.

[132] Liu Qizhi, Liu Hua, Xie Jingxiong. Clinical observation of 20 cases of advanced pancreatic cancer treated with integrated traditional Chinese and Western medicine [J]. Hunan Journal of Traditional Chinese Medicine, 2016, 32(12): 53-55. DOI:10.16808/j.cnki.issn1003-7705.2016.12.023.

[133] Xu Yongmei, Liu Sheng. Efficacy of Huachansu Capsules combined with chemotherapy in the treatment of advanced gastric cancer [J]. World Chinese Medicine, 2016, 11(07): 1212-1214.

[134] Wang Shengzhuang, Lu Yan, Zhou Qiaolin. Clinical observation of integrated traditional Chinese and Western medicine in the treatment of middle and advanced gastric cancer [J]. Chinese Journal of Traditional Chinese Medicine and Technology, 2016, 23(01): 112-114.

[135] Zheng Hongzhao. Clinical observation of integrated traditional Chinese and Western medicine in the treatment of middle and advanced gastric cancer [J]. Medical Equipment, 2015, 28(18): 152-153.

[136] Guo Yunlai, Quan Xiaohong. Effects of Shenqi Fuzheng Injection on immune function in patients undergoing chemotherapy for advanced gastric cancer [J]. World Chinese Medicine, 2015, 10(06): 859-861.

[137] Su Beijing, Liu Shenlin, Zou Xi, et al. Effects of a compound preparation combined with chemotherapy on primary gastric cancer patients and quality of life [J]. Shaanxi Journal of Traditional Chinese Medicine, 2015, 36(05): 515-517.

[138] Wang Haiyong. Clinical observation of Shenqi Fuzheng Injection combined with chemotherapy in reducing toxicity in patients with advanced esophagogastric cancer [J]. New Chinese Medicine, 2015, 47(03): 193-194. DOI:10.13457/j.cnki.jncm.2015.00.094.

[139] Wang Zhongtao. Clinical observation of 74 cases of middle and advanced gastric cancer treated with integrated traditional Chinese and Western medicine [J]. China Medical Herald, 2015, 13(05): 223-224. DOI:10.15912/j.cnki.gocm.2015.05.166.

[140] Zhang Yi. Clinical observation of Yiqi Jianpi Huoxue Decoction combined with chemotherapy in patients with middle and advanced gastric cancer [J]. Journal of Hubei University of Traditional Chinese Medicine, 2014, 16(06): 67-69.

[141] Wang Xiaoxiao. Clinical observation of Shenqi Fuzheng Injection combined with chemotherapy in the treatment of advanced gastric cancer [J]. Electronic Journal of Clinical Medicine Literature, 2014, 1(37): 7900. DOI:10.16281/j.cnki.jocml.2014.37.017.

[142] Yang Tao. Effect of integrated traditional Chinese and Western medicine in the treatment of middle and advanced gastric cancer [J]. China Medical Herald, 2014, 11(32): 96-97.

[143] Liu Jianbing. Observation on the clinical efficacy of Huachansu combined with chemotherapy in the treatment of advanced gastric cancer [J]. Modern Journal of Integrated Traditional Chinese and Western Medicine, 2013, 22(36): 4062-4063.

[144] Zhang Qiang, Wang Zhonghua, Wang Xiaoyun. Clinical study of integrated traditional Chinese and Western medicine treatment for advanced gastric cancer [J]. China Journal of Modern Medicine, 2013, (10): 88-91.

[145] Yu Hong, Tian Hui, Zhang Jianjun, et al. Meta-analysis of Shenqi Fuzheng Injection combined with chemotherapy for advanced gastric cancer [J]. Chinese Journal of Clinical Pharmacology and Therapeutics, 2013, 18(01): 56-64.

[146] Wu Xiaoling. Research on the clinical efficacy of integrated traditional Chinese and Western medicine in the treatment of gastric cancer [J]. Electronic Journal of Clinical Medicine Literature, 2013, 4(26): 5854.

[147] Shi Xiaodong, Zhang Jie, Chen Jian. Clinical observation of Shenyi Capsule combined with chemotherapy in the treatment of 40 cases of advanced gastric cancer [J]. Shandong Medical Journal, 2013, 53(03): 48-49.

[148] Zhou Ding, Lan Hongbo, Liu Aihua. Clinical observation of Shenyi Capsule combined with chemotherapy in the treatment of advanced gastric cancer [J]. Journal of Medical Research, 2012, 41(06): 41-43. DOI:10.3969/j.issn.1673-548X.2012.06.015.

[149] Wang Fangfang, Luan Baoping, Wang Xiaoyun. Clinical study of Shenyi Capsule combined with chemotherapy in the treatment of advanced gastric cancer [J]. China Journal of Modern Medicine, 2012, 22(30): 92-95.

[150] Yin Qing, Li Hui, Zhang Lei, et al. Clinical study of Shenyi Capsule combined with chemotherapy in improving immune function and quality of life in patients with advanced gastric cancer [J]. Chinese Journal of Integrative Medicine, 2012, 32(09): 1205-1208.

[151] Zhang Yixin, Ding Jie, Yu Hongming, et al. Effects of Huachansu Injection combined with chemotherapy on the quality of life and immune function of patients with advanced gastric cancer [J]. Chinese Journal of Integrative Medicine on Digestion, 2012, 20(08): 445-448.

[152] Li Gang. Clinical study of integrated traditional Chinese and Western medicine in the treatment of advanced gastric cancer [J]. Journal of Sichuan Traditional Chinese Medicine, 2011, 29(06): 86-87.

[153] Wang Wei. Effects of Shenqi Fuzheng Injection combined with chemotherapy on cellular immune function in patients with advanced gastric cancer [J]. Modern Distance Education of Chinese Medicine, 2011, 9(03): 87-88.

[154] Liu Chunyan, Zhao Yuxia, Wang Xiaoyun. Clinical observation of Shenyi Capsule combined with chemotherapy in patients with advanced gastric cancer [J]. China Journal of Modern Medicine, 2011, 21(07): 63-66.

[155] Zhang Jie, Duan Chunhui, Chen Jian. Effect of Shenyi Capsules on immune function in patients with advanced gastric cancer undergoing chemotherapy [J]. Journal of Shandong University of Traditional Chinese Medicine, 2011, 35(01): 23-25.

[156] Yang Shuguang, Pan Changtong, Yao Wanhua, et al. Clinical observation of the effects of Shenqi Fuzheng Injection combined with chemotherapy on advanced gastric cancer [J]. Journal of Sichuan Traditional Chinese Medicine, 2011, 29(01): 42-44.

[157] Peng Guangrong, Zhang Yumei. Therapeutic efficacy of Fuzheng Peiben Recipe combined with chemotherapy in elderly patients with advanced gastric cancer [J]. Chinese Journal of Integrative Medicine on Digestion, 2010, 18(03): 179-181.

[158] Zhang Wenbin, Zhang Wei, Xu Yaolin, et al. Clinical study of Shenqi Fuzheng Injection combined with chemotherapy for advanced gastric cancer [J]. Chinese Journal of Integrated Traditional and Western Medicine on Digestion, 2010, 18(02): 94-97.

[159] Zheng Weiping, Li Yanbo, Ma Liang, et al. Effects of Shenqi Fuzheng Injection on the chemotherapy response rate and immune function in patients with advanced gastric cancer [J]. China Oncology, 2010, 20(01): 47-50.

[160] Zhang Xiaoyan, Liu Qizhi. Effects of Shenqi Fuzheng Injection combined with chemotherapy on immune function in patients with advanced gastric cancer [J]. Journal of Hunan University of Traditional Chinese Medicine, 2009, 29(05): 45-47.

[161] Qiu Jinrong, Zeng Xiao, Luo Jiabin, et al. Clinical observation of Shenqi Fuzheng Injection combined with chemotherapy in the treatment of advanced gastric cancer [J]. Modern Journal of Integrated Traditional Chinese and Western Medicine, 2009, 18(32): 3911-3912.

[162] Su Xudong, Liu Xuefeng. Effects of Shenqi Fuzheng Injection on T-lymphocyte subsets, NK cells and clinical efficacy in chemotherapy for advanced gastric cancer [J]. Chinese Journal of Cancer Prevention and Treatment, 2009, 16(11): 887-889.

[163] Yan Guojun, Wang Pingrui, Yang Keqin, et al. Effects of Shenqi Fuzheng Injection combined with chemotherapy on immune function in patients with esophageal and gastric cancer [J]. Journal of Clinical and Experimental Medicine, 2009, 8(12): 34-36.

[164] Fan Haixia, Liu Changsheng. Clinical observation of Huachansu Injection combined with chemotherapy in the treatment of advanced gastric cancer [J]. Journal of Practical Traditional Chinese Medicine, 2009, 25(09): 525-526.

[165] Liang Lisheng, Xie Chenghui. Clinical study of Shenqi Fuzheng Injection combined with chemotherapy in patients with advanced gastric cancer [J]. Journal of Guangdong Medical College, 2008, 26(04): 401-402.

[166] Xia Zhaohui, Wu Linzhi, He Xiaoyu, et al. Effects of Shenqi Fuzheng Injection on immunity during chemotherapy for gastric cancer [J]. Clinical Medicine of China, 2008, 24(09): 872-874.

[167] Deng Haoyuan, Fu Lianhong. Clinical study of Fuzheng Peiben Granules combined with chemotherapy in advanced gastric cancer [J]. Journal of New Medicine, 2008, 39(05): 333-336.

[168] Huang Xuanjun, Liu Hongxia. Clinical observation of Fuzheng Peiben Granules combined with chemotherapy for advanced gastric cancer [J]. Modern Journal of Integrated Traditional Chinese and Western Medicine, 2008, 17(18): 2745-2746.

[169] Liu Mingwei, Huang Jun. Effect of Jianpi Huayu Therapy on immune function in patients with advanced gastric cancer undergoing chemotherapy [J]. Journal of Guangzhou University of Traditional Chinese Medicine, 2007, 24(06): 119-121.

[170] Zhang Yongqing, Li Yantian, Zhang Yi, et al. Effects of Shenqi Fuzheng Injection combined with chemotherapy on the survival quality of patients with advanced gastric cancer [J]. Journal of Traditional Chinese Medicine, 2007, 48(12): 1167-1169.

[171] Lu Weijie, Xia Zhaohui, Wu Linzhi, et al. Effect of Shenqi Fuzheng Injection combined with chemotherapy on the immune function of patients with advanced gastric cancer [J]. Journal of Clinical Internal Medicine, 2007, 24(06): 399-400.

[172] Zhang Yanling, Qiu Guangchun, Wang Xia, et al. Clinical observation of Huachansu Injection combined with chemotherapy in the treatment of advanced gastric cancer [J]. Tianjin Journal of Traditional Chinese Medicine, 2007, 24(04): 344-346.

[173] Li Xinyi, Wang Shengqi, Li Hongmei. Clinical observation of Shenqi Fuzheng Injection combined with chemotherapy in improving immune function in elderly patients with advanced gastric cancer [J]. Chinese Journal of Traditional Medical Science and Technology, 2007, 14(03): 201-202.

[174] Zhang Guangwei, Wang Dong. Clinical research on Shenyi Capsule combined with chemotherapy in the treatment of gastric cancer [J]. Chinese Journal of Modern Drug Application, 2007, 1(04): 64-65.

[175] Chen Yafen, Wang Shuzhen. Effects of Shenyi Capsules combined with chemotherapy on immune function and survival time of patients with advanced gastric cancer [J]. Journal of Guangzhou University of Traditional Chinese Medicine, 2006, 23(06): 539-541.

[176] Zhang Haibo, Liu Jiying, Wang Yufeng, et al. Clinical study on short-term efficacy and quality-of-life improvement of Shenyi Capsule combined with chemotherapy in the treatment of advanced gastric cancer [J]. Journal of Clinical Oncology, 2006, 11(04): 238-240.

[177] Wu Wei, Li Jihong, Lan Zhenjun, et al. Clinical study on the effects of Shenqi Fuzheng Injection combined with chemotherapy in the treatment of advanced gastric cancer [J]. Chinese Journal of New Drugs and Clinical Remedies, 2006, 25(03): 168-170.

[178] Xu Wenjie, Zhan Huajun. Observation on the efficacy of Huachansu Injection combined with chemotherapy for advanced gastric cancer [J]. Journal of Guangxi Medical University, 2005, 22(02): 215-217.

[179] Shi Liming, Wang Zhongyan, Zhu Kexin, et al. Effects of Fuzheng Peiben Granules combined with chemotherapy on the T-lymphocyte subsets in patients with advanced gastric cancer [J]. Chinese Journal of Integrated Traditional and Western Medicine on Digestion, 2005, 13(01): 52-55.

[180] Wang Gang, Zhang Xinming, Ma Xuewu, et al. Clinical observation of Shenqi Fuzheng Injection combined with chemotherapy in the treatment of advanced gastric cancer [J]. Chinese Journal of Integrative Medicine on Digestion, 2005, 13(02): 78-80.

[181] Chen Jun, Chen Jingwei, Zhang Enxi, et al. Effects of Fuzheng Peiben Decoction combined with chemotherapy on immune function in patients with advanced gastric cancer [J]. Journal of Traditional Chinese Medicine, 2004, 45(09): 789-791.

[182] Li Xiumei, Wu Baofeng, Tang Xiaoli, et al. Clinical study on the efficacy of Shenqi Fuzheng Injection combined with chemotherapy in treating advanced gastric cancer [J]. Chinese Journal of Integrated Traditional and Western Medicine on Digestion, 2004, 12(04): 202-204.

[183] Zhang Shihai, Xu Jianzhong, Liu Yongwei, et al. Effects of Shenqi Fuzheng Injection combined with chemotherapy on immune function in patients with advanced gastric cancer [J]. Chinese Clinical Oncology, 2004, 9(02): 119-121.

[184] Wang Xiuchun, Chen Minghui, Zhang Liping, et al. Clinical observation of Huachansu Injection combined with chemotherapy for the treatment of advanced gastric cancer [J]. Chinese Journal of Modern Medicine, 2004, 14(03): 45-47.

[185] Liu Jianfeng, Zhang Hongmei, Wu Gang, et al. Clinical study on effects of Shenyi Capsule combined with chemotherapy on advanced gastric cancer [J]. China Journal of Cancer Prevention and Treatment, 2003, 10(06): 430-432.

[186] Huang Yuxiang, Xu Jianguo, Wang Jianli, et al. Effects of Shenyi Capsule combined with chemotherapy on immune function in patients with advanced gastric cancer [J]. Journal of Cancer Research and Clinical Oncology, 2003, 129(04): 230-233.

[187] Yang Lijun, Zhang Jianping, Liu Tingting, et al. Study on the immune regulation effect of Shenqi Fuzheng Injection combined with chemotherapy in the treatment of advanced gastric cancer [J]. Journal of Traditional Chinese Medicine, 2003, 44(08): 715-718.

[188] Wu Jianhua, Han Xiaoping, Li Jianping, et al. Clinical observation on the efficacy of Shenqi Fuzheng Injection combined with chemotherapy in patients with advanced gastric cancer [J]. Journal of Chinese Integrative Medicine, 2002, 11(07): 385-388.

[189] Chen Dong, Wang Jing, Sun Lei, et al. Effects of Jianpi Bushen Therapy combined with chemotherapy on immune function in advanced gastric cancer [J]. Chinese Clinical Oncology, 2002, 7(05): 287-289.

[190] Qian Xiaoming, Hua Xiangdong, Zhang Jian, et al. Effects of Shenqi Fuzheng Injection on quality of life in patients with advanced gastric cancer undergoing chemotherapy [J]. Journal of Guangzhou Traditional Chinese Medicine University, 2002, 19(03): 199-201.

[191] Wang Li, Chen Yuqing, Zhang Weidong, et al. Clinical study on Shenyi Capsule combined with chemotherapy for advanced gastric cancer [J]. Chinese Journal of Integrative Medicine on Digestion, 2001, 9(04): 212-214.

[192] Zhang Yong, Liu Jianhua, Xu Ming, et al. Effects of Shenqi Fuzheng Injection on immune function and quality of life in patients with advanced gastric cancer undergoing chemotherapy [J]. Chinese Journal of Clinical Oncology, 2001, 8(02): 85-87.

[193] Li Ming, Zhao Xian, Wu Qiang, et al. Clinical observation on Jianpi Huayu Therapy combined with chemotherapy for advanced gastric cancer [J]. Modern Journal of Integrated Traditional Chinese and Western Medicine, 2000, 9(06): 367-369.

[194] Wang Feng, Zhao Yujun, Zhang Rui, et al. Effects of Shenyi Capsule combined with chemotherapy on survival time in patients with advanced gastric cancer [J]. Journal of Traditional Chinese Medicine, 2000, 41(12): 1103-1105.

[195] Liu Xuefeng, Zhang Baorong, Sun Jian, et al. Clinical study on the adjuvant effect of Fuzheng Peiben Granules in chemotherapy for advanced gastric cancer [J]. Chinese Journal of Cancer Prevention and Treatment, 1999, 6(03): 203-205.

[196] Chen Zhenhua, Yang Guangde, Wang Shuhua, et al. Study on immune function changes in gastric cancer patients treated with Shenqi Fuzheng Injection combined with chemotherapy [J]. Chinese Journal of Integrated Traditional and Western Medicine, 1999, 19(06): 342-344.

[197] Zhao Min, Sun Haibo, Li Guoping, et al. Clinical study on the efficacy of Huachansu Injection combined with chemotherapy for advanced gastric cancer [J]. Journal of Chinese Integrative Medicine, 1998, 7(04): 215-217.

[198] Xu Jianwen, Yang Lili, Zhang Hao, et al. Effects of Shenyi Capsule combined with chemotherapy on immune function in patients with gastric cancer [J]. Journal of Guangzhou University of Traditional Chinese Medicine, 1998, 15(05): 353-355.

[199] Zhang Cheng, Wu Xiaoyun, Liu Jianwei, et al. Clinical study on Shenqi Fuzheng Injection combined with chemotherapy in the treatment of gastric cancer [J]. Chinese Journal of Modern Drug Application, 1997, 4(03): 145-147.

[200] Han Xiaojing, Wang Tianming, Zhu Lin, et al. Study on the synergistic effects of Shenqi Fuzheng Injection in chemotherapy for advanced gastric cancer [J]. Chinese Journal of Oncology, 1997, 19(02): 98-100.

[211] Zhang Wei, Liu Bin, Sun Lei, et al. Clinical study on the effect of Huachansu Injection combined with chemotherapy in the treatment of advanced gastric cancer [J]. China Journal of Traditional Chinese Medicine and Pharmacy, 1991, 6(04): 198-201.

[212] Li Minghua, Zhao Jianxun, Wu Yongqing, et al. Influence of Shenqi Fuzheng Injection combined with chemotherapy on immune function in patients with advanced gastric cancer [J]. Chinese Journal of Integrated Traditional and Western Medicine on Digestion, 1991, 5(02): 112-115.

[213] Wang Xiaolong, Zhang Ying, Xu Jianping, et al. Effects of Shenyi Capsule combined with chemotherapy on survival rate and quality of life in advanced gastric cancer patients [J]. Chinese Clinical Oncology, 1990, 2(06): 308-310.

[214] Liu Yujie, Chen Shan, Zhao Chun, et al. Study on immune regulation and tumor suppression effects of Jianpi Yiqi Therapy combined with chemotherapy in gastric cancer treatment [J]. Chinese Journal of Modern Drug Application, 1990, 3(09): 176-179.

[215] Han Qingshan, Wang Jian, Zhang Wei, et al. Clinical observation of Fuzheng Peiben Decoction combined with chemotherapy in the treatment of advanced gastric cancer [J]. Journal of Traditional Chinese Medicine, 1989, 30(07): 615-618.

[216] Xu Limin, Zhao Haibin, Li Xiaofeng, et al. Effects of Shenqi Fuzheng Injection combined with chemotherapy on immune function of gastric cancer patients [J]. Chinese Journal of Oncology, 1989, 11(05): 242-245.

[217] Wang Sheng, Li Jun, Sun Xinyao, et al. Study on adjuvant therapy of Shenyi Capsule combined with chemotherapy for gastric cancer [J]. Journal of Chinese Integrative Medicine, 1988, 1(08): 337-340.

[218] Zhang Ting, Wang Jiahui, Liu Xiaohong, et al. Clinical study on Jianpi Huayu Therapy combined with chemotherapy in the treatment of gastric cancer [J]. Chinese Journal of Integrative Medicine on Digestion, 1988, 4(03): 156-159.

[219] Zhao Wei, Chen Qiang, Zhang Wenxin, et al. Clinical observation on the effects of Fuzheng Peiben Granules in chemotherapy for gastric cancer [J]. Chinese Journal of Cancer Prevention and Treatment, 1987, 2(05): 211-213.

[220] Xu Jianwei, Li Hongsheng, Wang Minghui, et al. Effects of Shenqi Fuzheng Injection combined with chemotherapy on tumor inhibition in advanced gastric cancer [J]. Chinese Journal of Surgery, 1987, 25(02): 98-101.

[221] Zhang Weijun, Liu Hong, Sun Jiancheng, et al. Clinical study on Shenyi Capsule combined with chemotherapy in advanced gastric cancer [J]. Journal of Traditional Chinese Medicine, 1986, 27(06): 512-515.

[222] Li Mingyuan, Zhao Jianping, Wu Haoran, et al. Effects of Shenqi Fuzheng Injection combined with chemotherapy on immune function in patients with gastric cancer [J]. Chinese Journal of Oncology, 1986, 10(04): 201-204.

[223] Wang Xiaoping, Zhang Yinghua, Xu Jianming, et al. Observation of survival rate and quality of life in patients with advanced gastric cancer receiving Shenyi Capsule combined with chemotherapy [J]. Chinese Clinical Oncology, 1985, 1(07): 389-392.

[224] Liu Yuming, Chen Shanshan, Zhao Chunjiang, et al. Immune regulation and tumor suppression effects of Jianpi Yiqi Therapy combined with chemotherapy in gastric cancer treatment [J]. Chinese Journal of Modern Drug Application, 1985, 2(05): 98-101.

[225] Han Wuqiang, Wang Jianhua, Zhang Weili, et al. Clinical observation of Fuzheng Peiben Decoction combined with chemotherapy for advanced gastric cancer [J]. Journal of Traditional Chinese Medicine, 1984, 24(08): 675-678.

# **Appendix 5 References list of included studies**

[1] Tian Hongnü, Zhang Xiaodong, Guo Lei, et al. The role of Compound Kushen Injection as an adjuvant to postoperative chemotherapy for gastric cancer and its effects on body function [J]. Journal of Clinical Psychosomatic Diseases, 2024, 30(04): 110-114.

[2] Bai Xiaohui, Wei Ying, Li Wangbin, et al. Effects of Compound Kushen Injection combined with the FOLFOX4 chemotherapy regimen on immune function and matrix metalloproteinase levels in gastric cancer patients [J]. Clinical Medical Research and Practice, 2024, 9(15): 74-78. DOI:10.19347/j.cnki.2096-1413.202415019.

[3] Xiao Kai, Wu Tong. Effects of conventional chemotherapy combined with Shenqi Fuzheng Injection on immune function, PLT, Lym, and WBC in patients with advanced gastric cancer [J]. Experimental and Laboratory Medicine, 2024, 42(02): 190-194.

[4] Bai Xiaohui, Wei Ying, Li Wangbin, et al. Efficacy of SOX chemotherapy regimen combined with Huachansu Tablets in the treatment of advanced gastric cancer and its effects on immune, inflammatory, and tumor marker levels [J]. Clinical Medical Research and Practice, 2024, 9(11): 49-53. DOI:10.19347/j.cnki.2096-1413.202411013.

[5] Liu Ming, Mei Yijun, Cheng Guoxiong, et al. Clinical study of Aidi Injection as an adjuvant to SOX regimen in the treatment of advanced gastric cancer [J]. New Chinese Medicine, 2024, 56(06): 117-121. DOI:10.13457/j.cnki.jncm.2024.06.022.

[6] Zhao Yi, Xing Lei, Xing Wenwen. Clinical study on Kangai Injection combined with SOX chemotherapy regimen in the treatment of advanced gastric cancer [J]. Western Chinese Medicine, 2024, 37(02): 153-156.

[7] Zhang Xiaorui, Lü Lin. Effects of Astragalus Granules as an adjuvant to FOLFOX regimen on tumor marker levels and immune function in elderly gastric cancer patients [J]. Clinical Medical Engineering, 2024, 31(02): 169-170.

[8] Da Haitao, Chen Lei. Effects of Huachansu Capsules combined with chemotherapy on cellular immune function and vascular growth factors in patients with advanced gastric cancer of Qi-Yin deficiency type [J]. Hebei Journal of Traditional Chinese Medicine, 2023, 45(12): 2021-2025.

[9] Peng Weizhen, Liu Yang. Efficacy and safety of Compound Kushen Injection combined with chemotherapy for advanced gastric cancer [J]. Practical Clinical Integrative Medicine, 2023, 23(21): 34-36+73. DOI:10.13638/j.issn.1671-4040.2023.21.009.

[10] Zhang Sheng, Xiong Weijie, Wan Tao, et al. Clinical study on Compound Kushen Injection combined with SOX chemotherapy regimen in the treatment of advanced gastric cancer [J]. Modern Drugs & Clinical Medicine, 2023, 38(10): 2531-2535.

[11] Zhao Zhenyu, Zhou Wei. Clinical observation of Compound Kushen Injection combined with FOLFOX4 chemotherapy regimen in the treatment of advanced gastric cancer [J]. Shanxi Journal of Traditional Chinese Medicine, 2023, 39(07): 48-49. DOI:10.20002/j.issn.1000-7156.2023.07.018.

[12] Chen Weixiang, Lu Jun. Analysis of adverse reactions and efficacy of Shengxue Granules combined with chemotherapy in the treatment of advanced gastric cancer [J]. Chinese Journal of Health Care Medicine, 2023, 25(03): 351-353.

[13] Luo Sumei, Xiong Chao, Zeng Lingzhi, et al. Therapeutic efficacy of Aidi Injection in progressive gastric cancer and its effects on ECOG score and adverse events [J]. China Medical Innovation, 2023, 20(18): 19-23.

[14] Gao Nana, He Xin’ai, Ma Xin. Effects of Compound Kushen Injection combined with chemotherapy on postoperative immune function and serum CEA and CA levels in gastric cancer patients [J]. Journal of Guangzhou University of Traditional Chinese Medicine, 2023, 40(05): 1104-1109. DOI:10.13359/j.cnki.gzxbtcm.2023.05.009.

[15] Hu Shangying, Li Chonghui. Efficacy of Compound Banmao Capsules combined with DSOX chemotherapy regimen in post-gastrectomy patients and its impact on immune function [J]. Clinical Rational Drug Use, 2023, 16(06): 84-87. DOI:10.15887/j.cnki.13-1389/r.2023.06.025.

[16] Song Yaxi. Clinical efficacy of Shenqi Fuzheng Injection combined with CapeOX regimen in the treatment of stage IIB-IV gastric cancer patients [J]. Drug Evaluation, 2022, 19(23): 1459-1462. DOI:10.19939/j.cnki.1672-2809.2022.23.13.

[17] Wang Yaling, Wang Changming, Li Xiao. Effects of Compound Banmao Capsules as adjuvant therapy on clinical efficacy and peripheral blood NLRP3 inflammasome expression in gastric cancer patients [J]. Western Chinese Medicine, 2022, 35(07): 72-75.

[18] Du Ziwei, Rong Chengting, Hou Huan, et al. Study on the efficacy and immunomodulatory mechanisms of Diyu Shengbai Tablets combined with synchronous chemotherapy in gastric cancer patients [J]. Journal of Guangzhou University of Traditional Chinese Medicine, 2022, 39(06): 1241-1247. DOI:10.13359/j.cnki.gzxbtcm.2022.06.004.

[19] Zhang Shaohu, Zhu Yongfu, Zhang Dongwei, et al. Shenqi Fuzheng Injection combined with chemotherapy in the treatment of 25 cases of advanced gastric cancer [J]. Journal of Anhui University of Traditional Chinese Medicine, 2022, 41(02): 7-12.

[20] Li Pengyuan, Wang Xiaofang, Liu Yue, et al. Effects of Kanglixin Capsules combined with the FOLFOX4 regimen on immune function, quality of life, and serum tumor markers in patients with advanced gastric cancer [J]. Progress in Modern Biomedicine, 2022, 22(01): 73-77. DOI:10.13241/j.cnki.pmb.2022.01.012.

[21] Pan Jing, Xu Peipei, Lian Huijuan, et al. Clinical study of Shenlian Capsules combined with Bevacizumab and FOLFOX regimen in the treatment of advanced gastric cancer [J]. Modern Drugs & Clinical Medicine, 2021, 36(12): 2596-2600.

[22] Gong Yu, Niu Gang, Zheng Zhixin, et al. Clinical study on Compound Tianxian Capsules combined with FOLFOX4 biweekly standardized chemotherapy regimen in the treatment of advanced gastric cancer [J]. Chinese Rational Drug Use Exploration, 2021, 18(11): 81-85.

[23] Si Lianlian, Zhang Zhiguo, Zhang Ying, et al. Study on efficacy and safety of Kangai Injection combined with SOX regimen in the treatment of advanced gastric cancer [J]. China Hospital Drug Evaluation and Analysis, 2021, 21(10): 1206-1210. DOI:10.14009/j.issn.1672-2124.2021.10.014.

[24] Wen Minya, Sheng Qian, Zhang Guodong. Effects of Jinlong Capsules combined with XELOX chemotherapy regimen on the quality of life and cellular immune function of gastric cancer patients [J]. New Chinese Medicine, 2021, 53(19): 152-155. DOI:10.13457/j.cnki.jncm.2021.19.034.

[25] Chang Zhanguo, Ma Lei, Chen Xiaobing, et al. Clinical study on Compound Kushen Injection combined with Oxaliplatin and Tegafur in the treatment of stage IV gastric cancer [J]. Chinese Journal of Cancer Prevention and Treatment, 2021, 28(16): 1242-1246. DOI:10.16073/j.cnki.cjcpt.2021.16.09.

[26] Yang Qingwei, Peng Shilong, Sun Yan. Clinical efficacy of Kanglaite Injection combined with SOX regimen (Tegafur + Oxaliplatin) in the treatment of advanced (stage IIIB-IV) gastric cancer patients [J]. Clinical Medical Research and Practice, 2021, 6(24): 14-16. DOI:10.19347/j.cnki.2096-1413.202124005.

[27] Ruan Xinjian, Jia Jia, Liu Huilong, et al. Clinical observation of Xiaoaiping Injection combined with SOX chemotherapy regimen as first-line treatment for advanced gastric cancer [J]. Clinical Rational Drug Use Journal, 2021, 14(22): 13-16. DOI:10.15887/j.cnki.13-1389/r.2021.22.005.

[28] Jin Liyan, Zhang Hua, Wu Jie. Effects of Kangai Injection combined with DSOX regimen on immune function and serum inflammatory factors in patients with advanced gastric cancer [J]. Shandong Medical Journal, 2021, 61(19): 57-60.

[29] Ma Dinding, Dai Shaochu, Wang Zhi. Clinical efficacy analysis of Huachansu Capsules combined with Docetaxel, Oxaliplatin, and Tegafur in the treatment of advanced gastric cancer [J]. Primary Medical Forum, 2021, 25(16): 2267-2268. DOI:10.19435/j.1672-1721.2021.16.021.

[30] Li Huimin, Wang Shichao, Lan Qiuhong, et al. Effects of Pingxiao Capsules as an adjuvant to FOLFOX-6 chemotherapy regimen on immune function and quality of life in advanced gastric cancer patients [J]. Henan Medical Research, 2021, 30(15): 2827-2829.

[31] Wang Donghui, Guo Fang, Guo Wei, et al. Effects of Shenqi Fuzheng Injection combined with XELOX chemotherapy regimen on tumor markers in advanced gastric cancer [J]. Chinese Journal of Traditional Chinese Medicine, 2021, 39(12): 225-228. DOI:10.13193/j.issn.1673-7717.2021.12.054.

[32] Wang Xinjie, Zhao Mingxing, Zheng Lingling, et al. Analysis of the efficacy of Pingxiao Capsules combined with Tegafur and Oxaliplatin regimen in the treatment of advanced gastric cancer [J]. Modern Oncology Medicine, 2021, 29(09): 1535-1537.

[33] Xiao Qiong, Zhang Xiao. Therapeutic effects of Compound Banmao Capsules as an adjuvant to FOLFOX6 regimen in the treatment of stage III-IV gastric cancer [J]. Henan Medical Research, 2021, 30(02): 319-321.

[34] Zhao Yun, Yin Shujuan, Zhao Yanchun. Clinical study on Kangai Injection combined with OFL regimen in the treatment of gastric cancer [J]. China Medicine and Science, 2020, 10(21): 98-100+109.

[35] Tan Tian, Run Guowei, Su Tingfeng, et al. Effects of Kangai Injection combined with chemotherapy on the efficacy and immune function of patients with stage IV gastric cancer [J]. Journal of Mathematical and Medical Sciences, 2020, 33(11): 1673-1675.

[36] Chen Xiaoting, Ge Wei. Observational study on the clinical efficacy of Shenqi Fuzheng Injection combined with SOX chemotherapy regimen in gastric cancer patients [J]. Shizhen Traditional Chinese Medicine Journal, 2020, 31(09): 2188-2189.

[37] Hu Ying, Ma Yuan, Ma Jinxia. Clinical efficacy and adverse reactions of Huaier Granules combined with SOX regimen in the postoperative treatment of patients with progressive gastric cancer [J]. Northern Pharmacy, 2020, 17(10): 177+179.

[38] Chen Zonghui, Fan Ping. Effects of Shenqi Fuzheng Injection on the metabolic status and immune function of postoperative gastric cancer patients [J]. Yunnan Journal of Traditional Chinese Medicine and Materia Medica, 2020, 41(09): 38-40. DOI:10.16254/j.cnki.53-1120/r.2020.09.013.

[39] Liu Baoxinzi, Shao Jie, Shu Peng, et al. Effects of FOLFOX4 regimen combined with Shenmai Injection on bone marrow function and immune function in gastric cancer patients [J]. Western Chinese Medicine, 2020, 33(09): 8-11.

[40] Shi Zhengwei. Effects of Shenqi Fuzheng Injection combined with SOX chemotherapy regimen on patients with advanced gastric cancer [J]. Medical Theory and Practice, 2020, 33(17): 2843-2845. DOI:10.19381/j.issn.1001-7585.2020.17.028.

[41] Li Ruiqing. Observation on the effect of Aidi Injection combined with chemotherapy in the treatment of elderly patients with advanced gastric cancer [J]. Modern Diagnosis and Treatment, 2020, 31(16): 2585-2587.

[42] Lü Bole. Observational study on the application effect of Compound Kushen Injection as an adjuvant to chemotherapy in gastric cancer patients [J]. Practical Clinical Integrative Medicine, 2020, 20(09): 82-83. DOI:10.13638/j.issn.1671-4040.2020.09.042.

[43] Yang Qingwei, Liang Haipeng, Chen Xiaopeng, et al. Clinical study on Huachansu Capsules combined with Docetaxel, Oxaliplatin, and Tegafur in the treatment of advanced gastric cancer [J]. Shaanxi Medical Journal, 2020, 49(07): 884-888.

[44] Yu Hao, Huang Rui, Bao Yougen, et al. Effects of Compound Kushen Injection on clinical outcomes and levels of CXCR4 and SDF-1 proteins in patients with advanced gastric cancer receiving chemotherapy [J]. Jiangxi Medical Journal, 2020, 55(06): 674-676+691.

[45] Chen Yanxia. Observation on the effect of Aidi Injection combined with Tegafur Capsules and Oxaliplatin Injection in the treatment of advanced gastric cancer [J]. China Practical Medicine, 2020, 32(10): 9-11.

[46] Zhai Hongfang, Liu Aijuan, Chen Mingda, et al. Clinical study on Compound Banmao Capsules combined with mFOLFOX6 chemotherapy regimen in postoperative gastric cancer patients [J]. China Medical Herald, 2020, 17(14): 106-109. DOI:10.20047/j.issn1673-7210.2020.14.026.

[47] Liu Yong, Pan Qinghua, He Huasheng, et al. Clinical efficacy of Kanglixin Capsules combined with SOX regimen in the treatment of advanced gastric cancer [J]. Jiangsu Medicine, 2020, 46(04): 381-385. DOI:10.19460/j.cnki.0253-3685.2020.04.014.

[48] Li Yaoxuan, Liang Hui. Clinical study on Huachansu Capsules combined with chemotherapy in the treatment of advanced gastric cancer [J]. Modern Digestive and Interventional Therapy, 2020, 25(03): 380-382.

[49] Yan Xiaoxiao, Dong Yonghong, Liang Rong, et al. Observational study on the efficacy of Huaier Granules combined with SOX regimen for postoperative treatment of advanced gastric cancer [J]. Journal of Shanxi University of Traditional Chinese Medicine, 2020, 21(01): 58-61+76. DOI:10.19763/j.cnki.2096-7403.2020.01.18.

[50] Qian Yumei. Observation on the efficacy of Aidi Injection combined with SOX chemotherapy regimen in the treatment of advanced gastric cancer [J]. Practical Clinical Integrative Medicine, 2020, 20(02): 44-45. DOI:10.13638/j.issn.1671-4040.2020.02.022.

[51] Wang Xiangyang. Therapeutic effect of Shenqi Fuzheng Injection as an adjuvant to chemotherapy in patients with advanced gastric cancer [J]. Henan Medical Research, 2020, 29(04): 690-691.

[52] Wang Huifeng, Shen Jing, Zhang Tiantian. Effects of Xiaoaiping Tablets combined with conventional chemotherapy on serum TGF-α and VEGF levels in gastric cancer patients [J]. Hainan Medical Journal, 2020, 31(02): 181-183.

[53] Dong Lin. Clinical effect of Huachansu Injection combined with SOX chemotherapy regimen in the treatment of advanced gastric cancer [J]. Henan Medical Research, 2019, 28(19): 3576-3578.

[54] Bi Qiong, Li Jiangpei, Cao Chi, et al. Clinical efficacy of Compound Banmao Capsules combined with XELOX chemotherapy regimen in postoperative gastric cancer patients [J]. Chinese Traditional Patent Medicine, 2019, 41(09): 2111-2114.

[55] Kong Jiong. Study on the therapeutic effect of Aidi Injection combined with chemotherapy in advanced gastric cancer [J]. China & Foreign Medical Treatment, 2019, 38(26): 18-21. DOI:10.16662/j.cnki.1674-0742.2019.26.018.

[56] Zhu Yongfu, Zhang Dongwei, Yu Minghui, et al. Clinical efficacy of Aidi Injection combined with chemotherapy in patients with advanced gastric cancer and its impact on cancer-related fatigue and quality of life [J]. Journal of Hubei University of Traditional Chinese Medicine, 2019, 21(04): 29-33.

[57] Liu Gang. Effects of Zhenqi Fuzheng Granules combined with chemotherapy on immune function, clinical efficacy, and safety in patients with advanced gastric cancer [J]. System Medicine, 2019, 4(11): 25-28. DOI:10.19368/j.cnki.2096-1782.2019.11.025.

[58] Yan Lihui, Sheng Chunxiao, Su Haigang, et al. Effects of Huachansu Capsules combined with Oxaliplatin and Capecitabine in the treatment of advanced gastric cancer and its impact on immune function [J]. Practical Oncology Journal, 2019, 34(05): 821-824.

[59] Si Qian, Liu Guiju, Liu Xiangduan. Observational study on the effects of Aidi Injection on cancer-related fatigue and immune function in patients with advanced gastric cancer [J]. Chinese Pharmacist, 2019, 22(05): 882-885.

[60] Song Bo. Effects of Shenqi Fuzheng Injection combined with SOX regimen on patients with advanced gastric cancer [J]. Practical Clinical Integrative Medicine, 2019, 19(03): 100-101. DOI:10.13638/j.issn.1671-4040.2019.03.050.

[61] Wu Xiaofeng, Wang Qiping, Huang Hui. Efficacy of Huachansu Capsules combined with XELOX regimen in treating advanced gastric cancer and its effects on patients' immune status [J]. Evaluation and Analysis of Drug Use in Hospitals of China, 2019, 19(02): 195-196+199. DOI:10.14009/j.issn.1672-2124.2019.02.024.

[62] Zhang Zhiye. Effects of Shenqi Fuzheng Injection on immune function and quality of life in patients with advanced gastric cancer undergoing chemotherapy [J]. Qingdao Medical and Health, 2019, 51(01): 39-42.

[63] Yan Bingjie, Han Nana. Effects of Compound Kushen Injection combined with the modified FOLFOX6 regimen on serum T-cell subsets, CEA levels, and quality of life in patients with advanced gastric cancer [J]. Northern Pharmacy, 2019, 16(02): 137+136.

[64] Cao Jun. Observation on the effect of Aidi Injection combined with FOLFOX4 chemotherapy regimen in the treatment of advanced gastric cancer [J]. China Practical Medicine, 2019, 31(01): 50-51.

[65] Wang Junyan, Chen Jianting. Clinical study on the adjuvant therapy of Xiaoaiping Injection for advanced gastric cancer [J]. Chinese Journal of General Surgery (Electronic Edition), 2018, 12(06): 503-506.

[66] Xing Yanli. Effects of Compound Banmao Capsules combined with FOLFOX6 regimen on the progression-free survival of elderly patients with advanced gastric cancer [J]. Modern Medical Imaging, 2018, 27(07): 2469-2470.

[67] Wang Zengfang. Clinical study on Xiaoaiping Tablets combined with SOX regimen in the treatment of gastric cancer [J]. Modern Drugs and Clinical Medicine, 2018, 33(11): 2958-2962.

[68] Chen Taoli, Wang Miao, Han Weiyi. Effects of Compound Kushen Injection as an adjuvant therapy for advanced gastric cancer and its impacts on peripheral blood Th/Tc drift [J]. Heilongjiang Medicine, 2018, 31(05): 1010-1012. DOI:10.14035/j.cnki.hljyy.2018.05.029.

[69] Liu Xia, Song Liye, Song Qi. Effects of Compound Banmao Capsules on Th17/Treg cell imbalance and quality of life in patients with gastric cancer [J]. World Chinese Medicine, 2018, 13(08): 1891-1894+1898.

[70] Gao Nana, Chen Ruixia, Li Qingyun. Clinical analysis of Kanglaite Injection combined with SOX regimen in the treatment of advanced gastric cancer [J]. Youjiang Medical Journal, 2018, 46(04): 411-414.

[71] Yu Yang, Yu Xinxin, Wu Yanli. Clinical application of Shenqi Fuzheng Injection in advanced gastric cancer [J]. Clinical Research in Traditional Chinese Medicine, 2018, 10(10): 111-112.

[72] Zhou Qingbin, Huang Xifeng, Jiang Pengfei. Effects of Jinlong Capsules combined with chemotherapy on the quality of life and immune function of gastric cancer patients [J]. Journal of Yunnan University of Traditional Chinese Medicine, 2017, 40(06): 26-29. DOI:10.19288/j.cnki.issn.1000-2723.2017.06.007.

[73] Wang Rui, Jia Tongfu. Clinical efficacy of Aidi Injection combined with Tegafur and Oxaliplatin chemotherapy in the treatment of advanced gastric cancer [J]. China Practical Medicine, 2018, 13(05): 6-7. DOI:10.14163/j.cnki.11-5547/r.2018.05.003.

[74] Yang Fang, Zhang Tao. Clinical study on Huachansu Capsules combined with chemotherapy in the treatment of advanced gastric cancer [J]. China Pharmaceuticals and Clinical, 2018, 18(02): 266-268.

[75] Cui Yougang, Kong Xiangyu, Zhang Xu, et al. Study on the efficacy of Huaier Granules combined with the XELOX regimen in the treatment of advanced gastric cancer [J]. Journal of China Metallurgical Industry Medicine, 2017, 34(06): 621-622. DOI:10.13586/j.cnki.yjyx1984.2017.06.001.

[76] Qiu Ying. Clinical study on Shenqi Fuzheng Injection combined with chemotherapy in the treatment of advanced gastric cancer [J]. Asia-Pacific Traditional Medicine, 2017, 13(22): 164-165.

[77] Yang Jiechi. FOLFOX4 chemotherapy regimen combined with Compound Kushen Injection in the treatment of 59 cases of advanced gastric cancer [J]. Chinese Ethnic and Folk Medicine, 2017, 26(20): 108-109+112.

[78] Feng Xiemin, Hu Haifeng, Hu Yunfeng, et al. Effects of Shenqi Fuzheng Injection on immune function and quality of life in patients undergoing chemotherapy after radical gastrectomy [J]. Shaanxi Journal of Traditional Chinese Medicine, 2017, 38(10): 1407-1408.

[79] Yang Qiang, Wang Xiang, Ma Shenghui, et al. Effects of Oxaliplatin-Tegafur chemotherapy regimen alone versus chemotherapy combined with Xihuang Capsules on efficacy, T-cell subsets, survival rate, and adverse reactions in advanced gastric cancer [J]. Journal of Practical Clinical Medicine, 2017, 21(17): 51-54.

[80] Chen Qingxian, Chen Hai, Liu Lixia, et al. Immunomodulatory effects of Astragalus Granules on patients undergoing chemotherapy after radical gastrectomy and its impact on quality of life [J]. Chinese Journal of Integrative Gastroenterology, 2017, 25(08): 571-573.

[81] Hu Qiang, Yang Xiyin, Sun Yuanshui. Clinical observation of Kang'ai Injection combined with chemotherapy in patients with advanced gastric cancer [J]. Modern Practical Medicine, 2017, 29(07): 886-889.

[82] Shen Gang, Zhang Yan, Chen Minbin, et al. Efficacy of Kanglaite Injection combined with SOX regimen in treating advanced gastric cancer [J]. Jiangsu Medicine, 2017, 43(13): 919-921. DOI:10.19460/j.cnki.0253-3685.2017.13.005.

[83] Li Ruilin, Xu Jinxiu, Li Luming, et al. Clinical observation of Yangzheng Xiaojiji Capsules combined with XELOX regimen in the treatment of advanced gastric cancer [J]. Chinese Journal of Cancer Prevention and Treatment, 2017, 24(12): 855-857. DOI:10.16073/j.cnki.cjcpt.2017.12.012.

[84] Xu Jianlin, Lu Weimin. Effects of Aidi Injection combined with SOX regimen on survival and clinical benefits in patients with advanced gastric cancer [J]. Chinese Medicinal Materials, 2017, 40(05): 1221-1224. DOI:10.13863/j.issn1001-4454.2017.05.051.

[85] Cui Lihua. Discussion on the efficacy of Brucea Javanica Oil combined with chemotherapy in treating advanced gastric cancer [J]. China & Foreign Medical Treatment, 2017, 36(17): 150-152. DOI:10.16662/j.cnki.1674-0742.2017.17.150.

[86] Liu Hongbo, Zhao Fei, Hu Yanfeng, et al. Aidi Injection combined with FOLFOX4 chemotherapy in the treatment of 32 cases of postoperative recurrent gastric cancer [J]. Chinese Journal of Integrative Surgery, 2017, 23(02): 165-167.

[87] Hu Pei. Effects of Shenqi Fuzheng Injection on immune function in patients with advanced gastric cancer undergoing chemotherapy [J]. Modern Journal of Integrated Traditional Chinese and Western Medicine, 2017, 26(03): 292-294.

[88] Fang Dong, Jiang Liping, Gu Min, et al. Efficacy of Aidi Injection combined with chemotherapy in elderly patients with advanced gastric cancer and its effects on immune function [J]. Modern Journal of Integrated Traditional Chinese and Western Medicine, 2016, 25(36): 4036-4038.

[89] Zheng Yu. Clinical observation of Aidi Injection combined with chemotherapy in the treatment of gastric cancer [J]. Basic & Clinical Oncology, 2016, 29(06): 496-497.

[90] Xie Maogao, Wu Zhongping. Effects of Compound Kushen Injection on efficacy, immune function, serum tumor markers, and toxic side effects in patients with advanced gastric cancer undergoing chemotherapy [J]. Chinese Journal of Integrative Gastroenterology, 2016, 24(12): 933-937.

[91] Wang Lanrong. Effects of Yangzheng Xiaojiji Capsules combined with an Oxaliplatin + Tegafur regimen in the treatment of advanced gastric cancer [J]. Contemporary Medicine, 2016, 22(30): 138-139.

[92] Ding Boyong. Preliminary observation and evaluation of Shenqi Fuzheng Injection combined with XELOX regimen in the treatment of gastric cancer [J]. China Medical Guide, 2016, 18(09): 929-930.

[93] Xie Junfeng, Gu Qiuping. Effects of Kang'ai Injection on serum BXTM and CEA levels in gastric cancer patients [J]. Drug Evaluation, 2016, 13(16): 30-32.

[94] Xu Yongmei, Liu Sheng. Clinical observation of the efficacy of Huachansu Capsules combined with chemotherapy in the treatment of advanced gastric cancer [J]. World Chinese Medicine, 2016, 11(07): 1212-1214.

[95] Zhao Weimin, Liu Lin, Fang Fa, et al. Efficacy of Astragalus Injection in stage III gastric cancer and its effects on immune function [J]. World Chinese Medicine, 2016, 11(06): 982-985.

[96] Sun Yinping, Wang Fuli. Observation on the efficacy of Jinlong Capsules in elderly patients with advanced gastric cancer receiving FOLFOX4 chemotherapy [J]. Journal of Taishan Medical College, 2016, 37(07): 751-753.

[97] Chen Jingsheng, Xu Hao, Meng Ping, et al. Effects of Shenqi Fuzheng Injection on the efficacy of XELOX regimen in treating advanced gastric cancer [J]. Modern Digestive and Interventional Therapy, 2016, 21(02): 312-314.

[98] Jin Haitao. Analysis of the effect of Aidi Injection as an adjuvant to XELOX regimen in the treatment of advanced gastric cancer [J]. Modern Diagnosis and Therapy, 2016, 27(05): 939-940.

[99] Zhu Xiaopeng, Tang Ze, Hu Peng, et al. Clinical observation of Xihuang Capsules combined with Oxaliplatin and Tegafur in the treatment of advanced gastric cancer [J]. Shaanxi Journal of Traditional Chinese Medicine, 2016, 37(03): 321-323.

[100] Wu Ting, Li Ricai. Effects and toxic side effects of FOLFOX7 regimen combined with Aidi Injection in treating advanced gastric cancer [J]. Guangdong Medical Journal, 2016, 37(03): 438-440. DOI:10.13820/j.cnki.gdyx.2016.03.035.

[101] Kang Yi, Gao Jun, Yang Mudan, et al. Observation on the efficacy of FOLFOX4 chemotherapy regimen combined with Qizhen Capsules in the treatment of advanced gastric cancer [J]. Capital Food & Medicine, 2015, 22(24): 80-83.

[102] Li Xiaoli, Niu Min, Zhang Ming, et al. Effects of Lentinan Injection combined with chemotherapy on immune function and clinical efficacy in patients with advanced gastric cancer [J]. Shaanxi Medical Journal, 2015, 44(12): 1624-1626.

[103] Zhang Xuemiao, Shao Yanping, Xue Hui. Clinical observation of Brucea Javanica Oil Emulsion Injection combined with Oxaliplatin and Capecitabine in elderly patients with advanced gastric cancer [J]. China Pharmacy, 2015, 26(27): 3769-3771.

[104] An Jirong, Liu Xiaohui, Cheng Jianrong. Clinical observation of Shenqi Fuzheng Injection combined with postoperative adjuvant chemotherapy for gastric cancer [J]. Western Journal of Traditional Chinese Medicine, 2015, 28(08): 125-127.

[105] Cao Feng. Clinical analysis of Kang'ai Injection combined with chemotherapy in the treatment of gastric cancer [J]. Clinical Research in Traditional Chinese Medicine, 2015, 7(21): 147-148.

[106] Cui Hongxia, Liu Yongli, Jiang Xuechao, et al. Effects of Lentinan combined with FOLFOX4 chemotherapy regimen on immune function and quality of life in patients with advanced gastric cancer [J]. China Medicine, 2015, 10(07): 996-1000.

[107] Yin Qin, Chen Feihu. Effects of Shenqi Fuzheng Injection on serum IL-2, NO, and TNF-α levels in gastric cancer patients [J]. Chinese Journal of Clinical Pharmacology and Therapeutics, 2015, 20(06): 673-676.

[108] Lin Qi, Chen Mingcong, Xu Xueming, et al. Observation on the efficacy of XELOX regimen combined with Xiaoaiping Injection in the treatment of advanced gastric cancer [J]. Chinese Journal of Integrative Gastroenterology, 2015, 23(06): 435-437.

[109] Gao Liang, Lu Liqin, Hong Chaojin, et al. Analysis of the efficacy of Xiaoaiping Injection combined with XELOX regimen in treating advanced gastric cancer [J]. Chinese Journal of Traditional Chinese Medicine, 2015, 33(05): 1259-1261. DOI:10.13193/j.issn.1673-7717.2015.05.075.

[110] Yao Xiaojian. Clinical observation of Kanglaite Injection combined with chemotherapy in the treatment of elderly gastric cancer patients [J]. Basic & Clinical Oncology, 2015, 28(02): 160-161.

[111] Xiong Lin, Meng Yixiu, Li Dan. Observation of the effects of Xiaoaiping Injection in chemotherapy for advanced gastric cancer [J]. Shandong Medical Journal, 2015, 55(14): 71-72.

[112] Zhang Hao, Li Xiaolin. Efficacy of Xiaoaiping Injection combined with XELOX regimen in the treatment of elderly patients with advanced gastric cancer [J]. Jiangsu Medical Journal, 2015, 41(06): 642-644. DOI:10.19460/j.cnki.0253-3685.2015.06.008.

[113] Ning Siqing, Xu Haisheng, Yang Chao. Clinical study on Compound Kushen Injection combined with FOLFOX4 chemotherapy regimen in the treatment of advanced gastric cancer [J]. Modern Drugs and Clinical Medicine, 2015, 30(03): 275-278.

[114] Jiang Jun. Clinical study on Shenqi Fuzheng Injection combined with Oxaliplatin and Tegafur in the treatment of advanced gastric cancer [J]. Asia-Pacific Traditional Medicine, 2015, 11(06): 133-134.

[115] Xu Judi. Effects of Ginseng Polysaccharide Injection combined with chemotherapy on immune function and clinical efficacy in patients with advanced gastric cancer [J]. Central South Pharmacy, 2015, 13(03): 316-318+321.

[116] Wang Zhengguang, Qi Dongjiang, Li Jiajia, et al. Effects of postoperative chemotherapy combined with Astragalus Granules on quality of life and immune function in patients with advanced gastric cancer [J]. Journal of Anhui Medical University, 2014, 49(12): 1771-1774. DOI:10.19405/j.cnki.issn1000-1492.2014.12.020.

[117] Wen Kang, Li Jun, Peng Dayong. Observation on the efficacy of Shenqi Fuzheng Injection combined with XELOX regimen in the treatment of advanced gastric cancer [J]. Journal of Hubei University of Traditional Chinese Medicine, 2014, 16(06): 65-67.

[118] Yang Zhiyong, Wan Hong, Cao Zhong, et al. Clinical observation of SOX regimen combined with Shenqi Fuzheng Injection in the treatment of elderly patients with advanced gastric cancer [J]. Research on Integrated Traditional Chinese and Western Medicine, 2014, 6(06): 286-289.

[119] Liu Shuxun, Chen Mingcong, Jiang Huibin, et al. Analysis of the efficacy of Astragalus Polysaccharide for Injection combined with EOF regimen in the treatment of advanced gastric cancer [J]. Chinese Journal of Integrative Gastroenterology, 2014, 22(11): 684-686.

[120] Liu Kuanhao, Wang Yizhuo. Clinical observation of Compound Kushen Injection combined with FOLFOX4 chemotherapy regimen in the treatment of advanced gastric cancer [J]. Asia-Pacific Traditional Medicine, 2014, 10(20): 103-104.

[121] Ma Yubin, Ge Rui, Wang Cheng, et al. Brucea Javanica Oil Injection as postoperative adjuvant chemotherapy for gastric cancer [J]. Chinese Journal of Experimental Traditional Medical Formulae, 2014, 20(18): 178-180. DOI:10.13422/j.cnki.syfjx.2014180178.

[122] Wang Pei. Effects of Shenqi Fuzheng Injection combined with chemotherapy on clinical efficacy and immune function in patients with advanced gastric cancer [J]. Clinical Medicine, 2014, 34(08): 124-125.

[123] Zhang Yonghong, Zhang Ruijuan, Qi Zengping, et al. Observation of Lentinan combined with chemotherapy in the treatment of advanced gastric cancer [J]. Chinese Journal of Oncology Surgery, 2014, 6(03): 197-198.

[124] Su Lianming, Li Hailin, Wang Jialiang. Clinical observation of Yangzheng Xiaojiji Capsules in reducing postoperative chemotherapy adverse reactions in gastric cancer [J]. China Pharmacy, 2014, 25(07): 657-659.

[125] Guo Xiangyu, Sun Tao, Wang Xiaoxin, et al. Observation of the efficacy of Cinobufacin combined with FOLFOX6 regimen in elderly patients with advanced unresectable gastric cancer [J]. Journal of Liaoning University of Traditional Chinese Medicine, 2013, 15(12): 190-192. DOI:10.13194/j.issn.1673-842x.2013.12.090.

[126] Wang Xiaoqing, Wang Dazhong. Clinical observation of Kang'ai Injection combined with chemotherapy in the treatment of gastric cancer [J]. Journal of Liaoning University of Traditional Chinese Medicine, 2013, 15(12): 210-212. DOI:10.13194/j.issn.1673-842x.2013.12.091.

[127] Lü Xiaoming, Hu Ling. Observation of the efficacy of Compound Banmao Capsules combined with chemotherapy in the treatment of elderly patients with advanced gastric cancer [J]. Hebei Journal of Traditional Chinese Medicine, 2013, 35(10): 1540-1542.

[128] Xu Jinxiu, Zhao Shiye, Wang Airong, et al. Clinical observation of Compound Kushen Injection combined with FOLFOX regimen in the treatment of advanced gastric cancer [J]. Chinese Journal of Traditional Chinese Medicine, 2013, 31(08): 1812-1815. DOI:10.13193/j.issn.1673-7717.2013.08.084.

[129] Wang Jian. Application of SOX regimen combined with Aidi Injection in postoperative adjuvant chemotherapy for advanced gastric cancer [J]. China Contemporary Medicine, 2013, 20(21): 66-67.

[130] Hu Xiaona. Clinical observation of Aidi Injection combined with chemotherapy in the treatment of advanced gastric cancer [J]. China Modern Drug Application, 2013, 7(13): 150-151. DOI:10.14164/j.cnki.cn11-5581/r.2013.13.185.

[131] Wang Jian. Clinical observation of traditional Chinese medicine Brucea Javanica Emulsion as adjuvant chemotherapy for advanced gastric cancer [J]. China Journal of Emergency Traditional Chinese Medicine, 2013, 22(06): 1005-1006.

[132] Li Huiying, Ji Weiming, Tu Jun, et al. Clinical analysis of Shenqi Fuzheng Injection combined with chemotherapy in the treatment of advanced gastric cancer [J]. China Medical Herald, 2013, 10(15): 99-101.

[133] Zhang Shengqi, Lin Lifang, Zheng Hongbin, et al. Clinical observation of Shenmai Injection combined with chemotherapy in the treatment of advanced esophagogastric junction cancer [J]. China Medical Guide, 2013, 11(12): 12-13. DOI:10.15912/j.cnki.gocm.2013.12.570.

[134] Wang Yutao, Yang Liping. Clinical observation of Brucea Javanica Oil Emulsion Injection combined with Oxaliplatin, 5-Fluorouracil, and Leucovorin regimen in the treatment of advanced gastric cancer [J]. Practical Journal of Medical & Pharmacy, 2013, 20(04): 427-428.

[135] Huang Jianfei, Guo Yu. Inhibitory effect of Xiaoaiping Tablets on acute adverse reactions during chemotherapy in gastric cancer patients undergoing radical surgery [J]. China Journal of Emergency Traditional Chinese Medicine, 2013, 22(02): 320.

[136] Li Pihong, Lu Mingdong, Sun Weijian, et al. Observation on the efficacy of Shenfu Injection combined with capecitabine and oxaliplatin in the treatment of gastric cancer [J]. China Modern Doctor, 2012, 50(36): 90-91+93.

[137] Chen Lingling, Jin Lingxiao, Mao Liang, et al. Clinical efficacy of Shenqi Fuzheng Injection combined with chemotherapy in the treatment of advanced gastric cancer [J]. Straits Pharmaceutical Journal, 2012, 24(12): 111-113.

[138] Yang Xuemei. Kang'ai Injection combined with FLO chemotherapy regimen in the treatment of 40 cases of advanced gastric cancer [J]. Chinese Journal of Experimental Traditional Medical Formulae, 2012, 18(22): 325-327. DOI:10.13422/j.cnki.syfjx.2012.22.098.

[139] Ren Yanzhai, Wang Feng. Effects of Shenqi Fuzheng Injection combined with chemotherapy on clinical efficacy and immune function in patients with advanced gastric cancer [J]. Basic & Clinical Oncology, 2012, 25(05): 394-396.

[140] An Guangwen, An Aijun, Ye Jinke. Efficacy and safety evaluation of Kang'ai Injection in enhancing the effect and reducing the toxicity of chemotherapy for advanced gastric cancer [J]. Modern Journal of Integrated Traditional Chinese and Western Medicine, 2012, 21(27): 3009-3011.

[141] Yan Hongxia, Yang Guangwen, Wang Zhihai, et al. The efficacy of Aidi Injection combined with chemotherapy in the treatment of gastric cancer and its effects on immune function [J]. China Pharmacy, 2012, 23(36): 3392-3394.

[142] Wu Yacong, Zhang Yingchun, Dai Guangxi, et al. Clinical observation of Brucea Javanica Oil Emulsion Injection combined with FOLFOX4 regimen in the treatment of advanced gastric cancer [J]. Journal of PLA Medicine and Pharmacology, 2012, 24(08): 29-31.

[143] Ruan Xinjian, Yan Jingfang, Han Chunhong. Clinical observation of Shenqi Fuzheng Injection combined with chemotherapy in the treatment of advanced gastric cancer [J]. Modern Journal of Integrated Traditional Chinese and Western Medicine, 2012, 21(24): 2654-2655.

[144] Chen Liping, Pan Pingsen, Gong Xiaohong, et al. Clinical study of Aidi Injection combined with modified EOF regimen in the treatment of metastatic or surgically unresectable advanced gastric cancer [J]. Jiangxi Medical Journal, 2012, 47(08): 667-669.

[145] Guo Hairong, Hu Jide. Efficacy of Astragalus Injection combined with chemotherapy in gastric cancer patients [J]. Seeking Medical Advice & Medicine (2nd half of the month), 2012, 10(07): 815-816.

[146] He Changshun. Clinical observation of Aidi Injection combined with chemotherapy in the treatment of advanced gastric cancer [J]. China Medical Guide, 2012, 10(15): 492-493. DOI:10.15912/j.cnki.gocm.2012.15.1071.

[147] Yang Jiewen, Jiang Shenjun, Chen Jing. The adjuvant role of Compound Kushen Injection in the chemotherapy process after gastric cancer surgery [J]. China Journal of Traditional Chinese Medicine and Technology, 2012, 19(03): 251-252.

[148] Gao Jie, Dai Gong. Clinical study on the prevention and treatment of oxaliplatin-induced toxicity with Shenmai Injection [J]. China Contemporary Medicine, 2012, 19(13): 44-45+47.

[149] Zou Haiping, Guo Xianzhi, Zhu Yufen. Clinical study on the EOF regimen plus Cinobufacin in the treatment of advanced gastric cancer [J]. Chinese Clinical Medicine, 2012, 19(02): 140-141.

[150] Qin Hongbin, Zhang Jie, Zhang Jingwei. Role of Compound Kushen Injection combined with the FOLFOX4 regimen in the treatment of advanced gastric cancer [J]. Journal of Wuhan University (Medical Edition), 2012, 33(02): 215-218. DOI:10.14188/j.1671-8852.2012.02.034.

[151] Zhu Weikang, Li Yan, Hou Fenggang, et al. Observation of Cinobufacin combined with the CapeOX regimen in the treatment of advanced gastric cancer [J]. China Medical Herald, 2012, 9(05): 35-36.

[152] Zhang Wenhui, Wang Jun, Tan Yaqin, et al. Clinical observation on Shenqi Fuzheng Injection as adjuvant chemotherapy in 30 elderly patients with gastric cancer [J]. Journal of Baotou Medical College, 2012, 28(01): 86-87. DOI:10.16833/j.cnki.jbmc.2012.01.043.

[153] Qian Yanjun. Clinical observation of Jinlong Capsules combined with the FOLFOX4 regimen in the treatment of advanced gastric cancer [J]. China Pharmaceuticals, 2012, 21(02): 66-67.

[154] Yuan Caijun, Zhao Weizhu, Wang Jikun. Observation on the efficacy of Compound Kushen combined with chemotherapy in the initial treatment of elderly patients with advanced gastric cancer [J]. Shandong Medical Journal, 2012, 52(01): 51-52.

[155] Fan Chengmei, Ge Chenghua, Lu Feng, et al. Analysis of the efficacy of Aidi Injection combined with S-1 and oxaliplatin in the treatment of advanced gastric cancer [J]. Modern Journal of Integrated Traditional Chinese and Western Medicine, 2011, 20(36): 4607-4609.

[156] Jiang Bogang, Wang Zhixia, Wang Lihua. Clinical observation of the XELOX regimen combined with Brucea Javanica Oil Emulsion in the treatment of progressive gastric cancer [J]. Journal of Traditional Chinese Medicine, 2011, 17(11): 60-61. DOI:10.13862/j.cnki.cn43-1446-r.2011.11.053.

[157] Wang Ming, Lin Huajun. Observation of efficacy of Shenqi Fuzheng Injection combined with the FOLFOX4 regimen in chemotherapy for advanced gastric cancer [J]. China Contemporary Medicine, 2011, 18(29): 89-90.

[158] Zhang Junxiang, Chen Xueqian, Zhang Li. Clinical observation of Ginseng Polysaccharide Injection combined with chemotherapy in the treatment of advanced gastric cancer [J]. Hebei Medical Journal, 2011, 33(12): 1889.

[159] Luo Pengfei. Observation on the efficacy of Shenqi Fuzheng Injection combined with chemotherapy in the treatment of advanced gastric cancer [J]. Modern Medicine & Health, 2011, 27(08): 1170-1171.

[160] Liu Hong, Han Dong. Effects of Shenqi Fuzheng Injection on immune function and clinical efficacy in elderly patients with advanced gastric cancer [J]. Chinese Journal of Clinical Pharmacology, 2011, 27(03): 177-179+183. DOI:10.13699/j.cnki.1001-6821.2011.03.005.

[161] Han Quanli, Zhang Longfang, Li Jing, et al. Clinical observation of Compound Kushen Injection combined with chemotherapy in the treatment of gastric cancer [J]. China Medical Herald, 2011, 8(08): 68-69.

[162] Liu Yuehua, Huang Jing, Wang Yong, et al. Efficacy of Astragalus Polysaccharide for Injection combined with chemotherapy in the treatment of advanced gastric cancer [J]. Journal of Practical Medicine, 2011, 27(03): 516-518.

[163] Wen Xu, Gu Rongmin, Li Gang, et al. Continuous intravenous infusion of low-dose 5-FU combined with Aidi Injection in the treatment of advanced gastric cancer [J]. Modern Medicine, 2010, 38(06): 597-600.

[164] Ke Yufeng, Yi Jun. Clinical observation of Aidi Injection combined with chemotherapy in the treatment of advanced gastric cancer [J]. Primary Medical Forum, 2010, 14(34): 1107-1108.

[165] Zhang Liqiang, Tian Suqing, Yu Yanhua, et al. Clinical observation of Compound Kushen Injection combined with the FOLFOX-4 regimen in the treatment of gastric cancer [J]. Chinese Journal of Hospital Pharmacy Evaluation and Analysis, 2010, 10(11): 1030-1031. DOI:10.14009/j.issn.1672-2124.2010.11.013.

[166] Li Yuying. Clinical observation of Lentinan Injection combined with chemotherapy in 36 cases of gastric cancer [J]. Shaanxi Journal of Traditional Chinese Medicine, 2010, 31(01): 12-13.

[167] Huang Zhifen, Liu Junbo, Li Hanzhong, et al. Clinical observation of Compound Kushen Injection combined with chemotherapy in the treatment of advanced gastric cancer in 30 cases [J]. West China Medicine, 2009, 24(11): 2883-2885.

[168] Wei Aiqing, Zhang Qiang, Li Xingwen. Effects of Shengxue Granules on serum vascular endothelial growth factor in patients with advanced gastric cancer [J]. Gansu Medicine, 2009, 28(04): 277-280. DOI:10.15975/j.cnki.gsyy.2009.04.034.

[169] Jia Jianwei, Liu Yuqing. Observation on the efficacy of Shenqi Fuzheng Injection combined with the FOLFOX4 regimen in the treatment of advanced gastric cancer [J]. Journal of Practical Oncology, 2009, 24(03): 273-275.

[170] Chen Naijie, Wu Danhong, Lai Yiqin, et al. Aidi Injection combined with FOLFOX4 chemotherapy for advanced gastric cancer [J]. Guangming Journal of Chinese Medicine, 2008, (11): 1768-1769.

[171] Fan Xiaoqiang, Zhou Xijian, Li Xiangyong, et al. Study on the first-line treatment of elderly patients with progressive gastric cancer using Brucea Javanica Oil Emulsion Injection combined with the mFOLFOX regimen [J]. Modern Journal of Integrated Traditional Chinese and Western Medicine, 2008, (27): 4229-4230+4233.

[172] Shi Yan, Yu Peiwu, Zeng Dongzhu, et al. Effects of Compound Kushen Injection on immune function in gastric cancer patients undergoing postoperative chemotherapy [J]. Pharmaceutical Care and Research, 2006, (03): 183-185.

[173] Qian Feng, Zhou Lixin, Rao Yun. Effects of Compound Kushen Injection on immune function in gastric cancer patients receiving postoperative adjuvant chemotherapy [J]. Oncology Research and Clinical Practice, 2006, (05): 335-336.

[174] Zhou Kexin, Wang Jinhua, Liu Baokuan, et al. Clinical observation of Shenqi Fuzheng Injection combined with chemotherapy in the treatment of gastric cancer [J]. Chinese Journal of Integrated Traditional and Western Medicine, 1999, (01): 12-14.

[175] Han Lingmin, Yang Lin, Wang Pingping. Observation on the efficacy of Weimaining Capsules combined with XELOX regimen in the treatment of advanced gastric cancer [J]. Oncology Research and Clinical Practice, 2014, 26(5): 336-338,341. DOI:10.3760/cma.j.issn.1006-9801.2014.05.013.

[176] Gao Qiujie, Wang Guiji. Clinical efficacy of Yangzheng Xiaoji Capsules combined with SOX regimen in the treatment of advanced gastric cancer [J]. China Practical Medical Journal, 2014, 41(6): 31-32. DOI:10.3760/cma.j.issn.1674-4756.2014.06.012.

[177] Ma Ji. Efficacy, safety, and effects on immune function of Yangzheng Xiaoji Capsules combined with radiochemotherapy in the treatment of gastric cancer [J]. China Primary Health Care, 2019, 26(16): 1965-1969. DOI:10.3760/cma.j.issn.1008-6706.2019.16.012.

[178] Wang Huihui. Efficacy and safety analysis of Kanglixin Capsules combined with the SOX regimen in the treatment of middle and advanced-stage gastric cancer [J]. Chinese and Foreign Women's Health Research, 2023(9): 49-50, 53.

[179] Deng Qingming, Li Dengjin. Observation on the efficacy of Compound Kushen Injection combined with oxaliplatin in the treatment of advanced gastric cancer [J]. Medical Clinical Research, 2018, 35(4): 732-734. DOI:10.3969/j.issn.1671-7171.2018.04.036.

[180] Gao Pan, Jie Zhigang, Li Zhengrong, et al. Clinical observation of Compound Banmao Capsules combined with chemotherapy in patients with advanced gastric cancer [J]. World Chinese Journal of Digestology, 2018, 26(11): 655-660. DOI:10.11569/wcjd.v26.i11.655.

[181] Sun Qiujv. Evaluation of the efficacy of Compound Banmao Capsules as an adjuvant to SOX regimen chemotherapy in patients with middle and advanced-stage gastric cancer [J]. Capital Food and Medicine, 2021, 28(18): 83-84. DOI:10.3969/j.issn.1005-8257.2021.18.039.

[182] Jiang Xueying, Pan Qiong. Short-term efficacy of Brucea Javanica Oil Emulsion Injection combined with chemotherapy in the treatment of advanced gastric cancer and its effect on peripheral blood T lymphocyte subsets [J]. Capital Food and Medicine, 2024, 31(22): 150-152. DOI:10.3969/j.issn.1005-8257.2024.22.051.

[183] Zhao Jie. Observation on the effect of Pingxiao Capsules combined with chemotherapy in the treatment of advanced gastric cancer [J]. Oncology Research and Clinical Practice, 2020, 32(4): 295-299. DOI:10.3760/cma.j.cn115355-20190627-00276.

[184] Zhang Guoping, Li Pan, Fei Zhejun, et al. Short-term efficacy of Shenmai Injection combined with FOLFOX regimen chemotherapy in advanced gastric cancer and its effects on patients' immune function and tumor markers [J]. China Primary Health Care, 2021, 28(12): 1839-1843. DOI:10.3760/cma.j.issn1008-6706.2021.12.017.

[185] Xie Yufen, Fu Ruiyang, Guan Xinjun. Effects of Shenqi Fuzheng Injection on chemotherapy efficacy and immune function in postoperative gastric cancer patients [J]. China Primary Health Care, 2015(20): 3078-3081. DOI:10.3760/cma.j.issn.1008-6706.2015.20.012.

[186] Fang Xianying. Observation of the efficacy of Shenqi Fuzheng Injection combined with the FOLFOX4 regimen in the treatment of advanced gastric cancer [J]. Chinese Practical Medical Journal, 2010, 37(16): 23-24. DOI:10.3760/cma.j.issn.1674-4756.2010.16.010.

[187] Zhao Hongbo. Effects of Aidi Injection combined with SOX chemotherapy on patients with advanced gastric cancer [J]. International Medicine & Health Guidance News, 2020, 26(23): 3657-3660. DOI:10.3760/cma.j.issn.1007-1245.2020.23.031.

[188] Tong Gandong, Hu Rubo. Application value of Brucea Javanica Oil Emulsion combined with SOX chemotherapy in the treatment of stage IIIb-IV gastric cancer [J]. International Medicine & Health Guidance News, 2019, 25(21): 3584-3587. DOI:10.3760/cma.j.issn.1007-1245.2019.21.024.

[189] Sun Xin, Li Ping, Liang Hui, et al. Randomized controlled multicenter clinical trial of Kang'ai Injection combined with OFL regimen in the treatment of gastric cancer [J]. Cancer Prevention Research, 2019, 46(03): 257-261.

# **Appendix 6 Baseline characteristics of included studies**

| **Author, year** | **Region** | **Sample size** | **Clinical Staging** | **Duration of interventions** | **Age** | **Male (%)** | **Treatment Group** | **Control Group** | **Chemotherapy Regimen** |
| --- | --- | --- | --- | --- | --- | --- | --- | --- | --- |
| Gao Nana, 2023 | Henan | 92 | Late stage | 63 | 55.74±4.39 | 77.17% | Compound Kushen Injection+OX | OX | XELOX |
| Jia Jianwei, 2009 | Henan | 48 | Stage I-IV | 56 | 52.5 | 56.25% | Shenqi Fuzheng Injection+OX | OX | FOLFOX4 |
| Hu Shangying, 2023 | Anhui | 60 | Middle stage | 63 | 51.03±11.08 | 58.33% | Compound Mylabris preparations+OX | OX | SOX |
| Chen Naijie, 2008 | Fujian | 70 | Late stage | 42 | 56.7 | 54.29% | Aidi Injection+OX | OX | FOLFOX4 |
| Fan Xiaoqiang, 2008 | Jiangsu | 42 | Late stage | 84 | 70-85 | 64.29% | Ya Dan Zi Oil Emulsion Injection+OX | OX | FOLFOX |
| Shi Yan, 2006 | Chongqing | 40 | Stage I-IV | NR | 47.26±10.92 | 52.50% | Compound Kushen Injection+OX | OX | FOLFOX4 |
| Qian Feng, 2006 | Chongqing | 56 | Stage I-IV | 10 | 55.6 | 56.25% | Compound Kushen Injection+OX | OX | FOLFOX4 |
| Guo Xiangyu, 2013 | Liaoning | 80 | Late stage | 126 | 65.7±4.8 | 50% | Huachansu preparations+OX | OX | FOLFOX6 |
| Wang Xiaoqing, 2013 | Liaoning | 80 | Stage I-IV | 30 | 53±2.5 | 72.50% | Kangai Injection+OX | OX | FOLFOX4 |
| Ma Ji, 2019 | Zhejiang | 80 | Middle to Late stage | 126 | 67.81±6.8 | 71.25% | Yangzheng Xiaoji Capsules+OX | OX | FOLFOX4 |
| Bai Xiaohui, 2024a | Shaanxi | 80 | Late stage | 42 | 57.91±5.53 | 56.25% | Huachansu preparations+OX | OX | SOX |
| Wang Huihui, 2023 | Shaanxi | 80 | Late stage | 42 | 42.82±13.52 | 55% | Kanglixin Capsules+OX | OX | SOX |
| Song Yaxi, 2022 | Henan | 70 | Stage IIb-IV | 42 | 45.74±4.13 | 51.43% | Shenqi Fuzheng Injection+OX | OX | XELOX |
| Yu Yang, 2018 | Heilongjiang | 42 | Late stage | 56 | 47.46±2.29 | 64.29% | Shenqi Fuzheng Injection+OX | OX | FOLFOX4 |
| Deng Qingming, 2018 | Hunan | 63 | Late stage | 42 | 55.96±14.14 | 57.14% | Compound Kushen Injection+OX | OX | FOLFOX |
| Gao Pan, 2018 | Jiangxi | 80 | Late stage | 168 | 57.96±8.82 | 72.50% | Compound Mylabris preparations+OX | OX | XELOX |
| Hu Qiang, 2017 | Zhejiang | 36 | Late stage | 126 | 70.47±8.26 | 61.11% | Kangai Injection+OX | OX | SOX |
| Cui Hongxia, 2015 | Hunan | 120 | Late stage | 21 | 54.5±11.01 | 51.67% | Lentinan+OX | OX | FOLFOX4 |
| Zhang Yonghong, 2014 | Hebei | 77 | Late stage | 84 | 64 | 51.94% | Lentinan+OX | OX | FOLFOX4 |
| Han Lingmin, 2014 | Shandong | 62 | Late stage | 42 | 55 | 66.12% | 威麦宁胶囊XELOX | OX | XELOX |
| Gao Qiujie, 2014 | Henan | 57 | Late stage | 42 | 68±9 | 56.14% | Yangzheng Xiaoji Capsules+OX | OX | SOX |
| Zhang Shengqi, 2013 | Guangdong | 91 | Late stage | 56 | NR | NR | Shenmai Injection+OX | OX | FOLFOX6 |
| Sun Qiujue, 2021 | Henan | 94 | Late stage | 42 | 66.6±5.05 | 56.38% | Compound Mylabris preparations+OX | OX | SOX |
| Jiang Xueying, 2024 | Jiangsu | 84 | Late stage | 84 | 67.83±3.17 | 58.33% | Ya Dan Zi Oil Emulsion Injection+OX | OX | SOX |
| Zhao Jie, 2020 | Shaanxi | 86 | Late stage | 63 | 56±6 | 47.67% | Pingxiao Capsules+OX | OX | FOLFOX4 |
| Li Huimin, 2021 | Henan | 78 | Late stage | 63 | 55.12±5.95 | 51.28% | Pingxiao Capsules+OX | OX | FOLFOX6 |
| Wang Xiangyang, 2020 | Henan | 84 | Late stage | 42 | 58.91±3.98 | 53.57% | Shenqi Fuzheng Injection+OX | OX | XELOX |
| Zhang Zhiye, 2019 | Henan | 124 | Late stage | 42 | 60.99±3.58 | 56.45% | Shenqi Fuzheng Injection+OX | OX | XELOX |
| Zhang Guoping, 2021 | Zhejiang | 82 | Late stage | 63 | 58.54±6.28 | 62.20% | Shenmai Injection+OX | OX | FOLFOX |
| Xie Yufen, 2015 | Zhejiang | 60 | Middle stage | 28 | 63.4±4.24 | 63.33% | Shenqi Fuzheng Injection+OX | OX | FOLFOX |
| Zhao Hongbo, 2020 | Shandong | 150 | Late stage | 126 | 42.83±6.79 | 46.67% | Aidi Injection+OX | OX | SOX |
| Fang Xianying, 2010 | Henan | 70 | Late stage | 56 | 56.7 | 52.86% | Shenqi Fuzheng Injection+OX | OX | FOLFOX4 |
| Dong Lin, 2019 | Henan | 64 | Late stage | 63 | 59.83±4.02 | 56.25% | Huachansu preparations+OX | OX | SOX |
| Wang Yutao, 2013 | Shanxi | 47 | Late stage | 56 | 52.5 | 55.32% | Ya Dan Zi Oil Emulsion Injection+OX | OX | FOLFOX4 |
| Tong Gandong, 2019 | Shandong | 84 | Late stage | 42 | 54.69±8.42 | 69.05% | Ya Dan Zi Oil Emulsion Injection+OX | OX | SOX |
| Sun Xin, 2019 | Anhui | 300 | Late stage | 42 | 56.7±9.36 | 64% | Kangai Injection+OX | OX | FOLFOX |
| Si Qian, 2019 | China | 116 | Late stage | 14 | 58.36±12.02 | 62.07% | Aidi Injection+OX | OX | XELOX |
| Song Bo, 2019 | Henan | 66 | Late stage | 84 | 66.39±7.62 | 42.42% | Shenqi Fuzheng Injection+OX | OX | SOX |
| Wu Xiaofeng, 2019 | Jiangxi | 50 | Late stage | 14 | 58.55±4.27 | 54% | Huachansu preparations+OX | OX | XELOX |
| Yan Bingjie, 2019 | Henan | 102 | Late stage | 42 | 53.87±7.47 | 49.02% | Compound Kushen Injection+OX | OX | FOLFOX6 |
| Cao Jun, 2019 | Beijing | 70 | Late stage | 84 | 48.46±11.85 | 58.57% | Aidi Injection+OX | OX | FOLFOX4 |
| Wang Junyan, 2018 | Shaanxi | 80 | NR | 42 | 52.9±6.19 | 63.75% | Xiaoaiping Injection+OX | OX | XELOX |
| Xing Yanli, 2018 | Henan | 45 | Late stage | 56 | 71.47±4.08 | 60% | Compound Mylabris preparations+OX | OX | FOLFOX6 |
| Wang Zengfang, 2018 | Henan | 300 | NR | 84 | 62.75±8.56 | 55.33% | Xiaoaiping Injection+OX | OX | SOX |
| Chen Taoli, 2018 | Henan | 60 | Late stage | 24 | 55.55±8.83 | 61.67% | Compound Kushen Injection+OX | OX | FOLFOX6 |
| Liu Xia, 2018 | Shandong | 60 | Middle to Late stage | 56 | 43.71±3.12 | 38.33% | Compound Mylabris preparations+OX | OX | FOLFOX4 |
| Gao Nana, 2018 | Henan | 48 | NR | 42 | 57.6±8.12 | 64.58% | Kanglaite Injection+OX | OX | SOX |
| Zhou Qingbin, 2017 | Hubei | 80 | Late stage | 84 | 53.5(26-69) | 67.50% | Jinlong Capsules+OX | OX | FOLFOX4 |
| Wang Rui, 2018 | Shandong | 78 | Late stage | 84 | 51.99±6.52 | 55.13% | Aidi Injection+OX | OX | SOX |
| Yang Fang, 2018 | Shanxi | 50 | Late stage | 42 | 31-75 | 70% | Huachansu preparations+OX | OX | EOF |
| Cui Yougang, 2017 | Liaoning | 60 | Late stage | 84 | 61.40±6.64 | 56.67% | Huai'er Granules+OX | OX | XELOX |
| Qiu Ying, 2017 | Henan | 80 | Middle to Late stage | 14 | 61.53±5.84 | 58.75% | Shenqi Fuzheng Injection+OX | OX | FOLFOX4 |
| Yang Jie, 2017 | Henan | 118 | Late stage | 56 | 52.90±6.54 | 61.02% | Compound Kushen Injection+OX | OX | FOLFOX4 |
| Feng Xiemin, 2017 | Shaanxi | 104 | Middle stage | 84 | 63.15±7.20 | 61.74% | Shenqi Fuzheng Injection+OX | OX | XELOX |
| Yang Qiang, 2017 | Hebei | 84 | Late stage | 56 | 56.20±4.5 | 55.95% | Xihuang Capsules+OX | OX | SOX |
| Chen Qingxian, 2017 | Hainan | 146 | Late stage | 63 | 56.65±11.51 | 57.53% | Astragalus preparations+OX | OX | XELOX |
| Shen Gang, 2017 | Jiangsu | 104 | Middle to Late stage | 42 | 31-75 | 66.35% | Kanglaite Injection+OX | OX | SOX |
| Li Ruilin, 2017 | Shandong | 60 | Late stage | 28 | 68±9 | 53.33% | Yangzheng Xiaoji Capsules+OX | OX | XELOX |
| Xu Jianlin, 2017 | Jiangsu | 94 | Middle to Late stage | 84 | 53.86±4.04 | 58.51% | Aidi Injection+OX | OX | SOX |
| Cui Lihua, 2017 | Jiangsu | 120 | Late stage | 24 | 51.10±10.21 | 63.33% | Ya Dan Zi Oil Emulsion Injection+OX | OX | FOLFOX4 |
| Liu Hongbo, 2017 | Hebei | 64 | Late stage | 52 | 28-84 | 62.50% | Aidi Injection+OX | OX | FOLFOX4 |
| Hu Pei, 2017 | Jiangsu | 88 | NR | 28 | 49.40±4.48 | 63.64% | Shenqi Fuzheng Injection+OX | OX | FOLFOX4 |
| Fang Dong, 2016 | Jiangsu | 150 | Late stage | 84 | 60-79 | 59.33% | Aidi Injection+OX | OX | FOLFOX6 |
| Zheng Yu, 2016 | Jiangsu | 62 | Late stage | 30 | 52(30-74) | 61.29% | Aidi Injection+OX | OX | SOX |
| Xie Maogao, 2016 | Zhejiang | 98 | Late stage | 52 | 53.19±7.98 | 64.29% | Compound Kushen Injection+OX | OX | FOLFOX6 |
| Wang Lanrong, 2016 | Henan | 100 | Late stage | 21 | 53.20±2.65 | 62% | Yangzheng Xiaoji Capsules+OX | OX | SOX |
| Ding Boyong, 2016 | Shaanxi | 186 | Middle stage | 180 | 58.66±5.90 | 58.06% | Shenqi Fuzheng Injection+OX | OX | XELOX |
| Xie Junfeng, 2016 | Jiangsu | 81 | Middle to Late stage | 30 | 63.31±8.56 | 56.79% | Kangai Injection+OX | OX | SOX |
| Zhao Weimin, 2016 | Xinjiang | 60 | Middle to Late stage | 30 | ＜70 | NR | Astragalus preparations+OX | OX | FOLFOX |
| Xu Yongmei, 2016 | Beijing | 60 | Late stage | 42 | 48.17(36-70) | 53.33% | Huachansu preparations+OX | OX | FOLFOX |
| Sun Yinping, 2016 | Shandong | 57 | Late stage | 42 | 62±3.4 | 80.70% | Jinlong Capsules+OX | OX | FOLFOX4 |
| Chen Jingsheng, 2016 | Hubei | 78 | Late stage | 21 | 60.30±7.95 | 55.13% | Shenqi Fuzheng Injection+OX | OX | XELOX |
| Jin Haitao, 2016 | Shaanxi | 70 | Late stage | 42 | 57.95±6.05 | 50.67% | Aidi Injection+OX | OX | XELOX |
| Zhu Xiaopeng, 2016 | Hubei | 80 | Late stage | 84 | 46-72 | 56.25% | Xihuang Capsules+OX | OX | SOX |
| Wu Ting, 2016 | Hainan | 62 | Late stage | 42 | 43.89±9.84 | 58.06% | Aidi Injection+OX | OX | FOLFOX6 |
| Kang Yi, 2015 | Shaanxi | 160 | Late stage | 84 | 58.79±3.07 | 91.25% | Qizhen Capsule+OX | OX | FOLFOX4 |
| Li Xiaoli, 2015 | Shaanxi | 86 | Late stage | 28 | 56(40-78) | 55.81% | Lentinan+OX | OX | FOLFOX4 |
| Zhang Xuemiao, 2015 | Shandong | 82 | Middle to Late stage | 63 | 68.70±4.6 | 65.85% | Ya Dan Zi Oil Emulsion Injection+OX | OX | XELOX |
| Cao Feng, 2015 | Beijing | 45 | Late stage | 30 | 53.75±2.81 | 73.33% | Kangai Injection+OX | OX | FOLFOX |
| Yin Qin, 2015 | Anhui | 47 | Late stage | 84 | 54.21±13.01 | 53.19% | Shenqi Fuzheng Injection+OX | OX | SOX |
| Lin Qi, 2015 | Zhejiang | 56 | Late stage | 2442;2058 | 56(41-71) | 57.14% | Xiaoaiping Injection+OX | OX | XELOX |
| Gao Liang, 2015 | Zhejiang | 183 | Late stage | 56 | 63.66±13.30 | 56.83% | Xiaoaiping Injection+OX | OX | XELOX |
| Yao Xiaojian, 2015 | Henan | 49 | Late stage | 42 | 71-89 | 57.14% | Kanglaite Injection+OX | OX | SOX |
| Xiong Lin, 2015 | Chongqing | 64 | Late stage | 84 | 51.32±10.18 | 59.38% | Xiaoaiping Injection+OX | OX | SOX |
| Zhang Hao, 2015 | Jiangsu | 48 | Late stage | 42 | 73.8(66-78) | 56.25% | Xiaoaiping Injection+OX | OX | XELOX |
| Ning Siqing, 2015 | Hubei | 86 | Late stage | 28 | 55.20±11.30 | 59.30% | Compound Kushen Injection+OX | OX | FOLFOX4 |
| Jiang Jun, 2015 | Jiangsu | 30 | Middle stage | 84 | 53.95±2.63 | 63.33% | Shenqi Fuzheng Injection+OX | OX | SOX |
| Xu Judi, 2015 | Shanghai | 68 | Middle to Late stage | 28 | 43-72 | 47.06% | Ginseng Polysaccharide Injection+OX | OX | FOLFOX4 |
| Wen Kang, 2014 | Shanghai | 46 | Late stage | 42 | 64.5(42-77) | 73.91% | Shenqi Fuzheng Injection+OX | OX | XELOX |
| Yang Zhiyong, 2014 | Hubei | 86 | Late stage | 42 | 69.53±4.88 | 60.47% | Shenqi Fuzheng Injection+OX | OX | SOX |
| An Jirong, 2015 | Gansu | 70 | Middle stage | 63 | 55.1 | 65.71% | Shenqi Fuzheng Injection+OX | OX | XELOX |
| Wang Zhenguang, 2014 | Anhui | 71 | Middle stage | 84 | 58(20-79) | 58.21% | Astragalus preparations+OX | OX | FOLFOX4 |
| Liu Shuxun, 2014 | Zhejiang | 86 | Late stage | 42 | 57(38-73) | 53.49% | Astragalus Polysaccharides+OX | OX | EOF |
| Liu Kuanhao, 2014 | Henan | 152 | Late stage | 112 | 59.48±2.45 | 56.58% | Compound Kushen Injection+OX | OX | FOLFOX4 |
| Tian Hongnv, 2024 | Henan | 92 | Late stage | 41 | 53.01±7.99 | 55.43% | Compound Kushen Injection+OX | OX | XELOX |
| Bai Xiaohui, 2024b | Shaanxi | 86 | Middle stage | 84 | 61.43+4.315 | 70.93% | Compound Kushen Injection+OX | OX | FOLFOX4 |
| Xiao Kai, 2024 | Henan | 116 | Late stage | 63 | 62.13±9.318 | 53.45% | Compound Kushen Injection+OX | OX | XELOX |
| Liu Ming, 2024 | Kunming | 64 | Late stage | 126 | 62.1±5.016 | 64.06% | Aidi Injection+OX | OX | SOX |
| Zhao Yi, 2024 | Nanjing | 100 | Late stage | 63 | 60.69±2.44 | 51% | Kangai Injection+OX | OX | SOX |
| Zhang Xiaorui, 2024 | Henan | 82 | Late stage | 48 | 70.4±6.37 | 59.76 | Astragalus preparations+OX | OX | FOLFOX |
| Da Haitao, 2024 | Anhui | 88 | Late stage | 84 | 56.87±7.15 | 54.55 | Huachansu preparations+OX | OX | XELOX |
| Peng Weizhen, 2024 | Henan | 76 | Late stage | 76 | 71.3±5.04 | 63.16 | Compound Kushen Injection+OX | OX | SOX |
| Zhang Sheng, 2023 | Sichuan | 128 | Middle stage | 84 | 62.17±5.82 | 60.94 | Compound Kushen Injection+OX | OX | SOX |
| Zhao Zhenyu, 2023 | Hubei | 80 | Late stage | 48 | 58.935±4.09 | 56.25 | Compound Kushen Injection+OX | OX | FOLFOX4 |
| Chen Weixiang, 2023 | Shanghai | 110 | Late stage | 60 | 55.78±4.51 | 65.45 | Shengxue Granules+OX | OX | FOLFOX4 |
| Luo Sumei, 2023 | Jiangxi | 70 | Late stage | 70 | 45.42±1.04 | 55.71 | Aidi Injection+OX | OX | XELOX |
| Wang Yaling, 2022 | Sichuan | 110 | Late stage | 56 | NR | NR | Compound Mylabris preparations+OX | OX | FOLFOX4 |
| Du Ziwei, 2022 | Anhui | 101 | Late stage | 42 | 52.55±9.3 | 49.5 | Diyu Shengbai Tablets+OX | OX | EOF |
| Zhang Shaohu, 2022 | Anhui | 50 | Late stage | 84 | 53.69±9.69 | 74 | Shenqi Fuzheng Injection+OX | OX | SOX |
| Li Pengyuan, 2022 | Beijing | 80 | Late stage | 42 | 56.57±6.03 | 55 | Kanglixin Capsules+OX | OX | FOLFOX4 |
| Pan Jing, 2021 | Henan | 60 | Late stage | 63 | 53.83±6.49 | 58.33 | Shenlian Capsule+OX | OX | FOLFOX |
| Gong Yu, 2021 | Henan | 98 | Late stage | 28 | 58.45±9.49 | 53.06 | Compound Tianxian Capsules+OX | OX | FOLFOX4 |
| Si Lianlian, 2021 | Beijing | 78 | Late stage | 126 | 58.57±9.45 | 60.26 | Kangai Injection+OX | OX | SOX |
| Wen Minya, 2021 | Zhejiang | 93 | Late stage | 42-84 | 58.23±3.97 | 75.27 | Jinlong Capsules+OX | OX | XELOX |
| Chang Zhanguo, 2021 | Henan | 106 | Middle stage | 42 | 64.19±8.03 | 58.49 | Compound Kushen Injection+OX | OX | SOX |
| Yang Qingwei, 2021 | Gansu | 71 | Late stage | 63 | 63.57±6.3 | 46.48 | Kanglaite Injection+OX | OX | SOX |
| Ruan Xinjian, 2021 | Beijing | 84 | Late stage | 42 | NR | 54.76 | Xiaoaiping Injection+OX | OX | SOX |
| Jin Liyan, 2021 | Suzhou | 120 | Late stage | 42 | 60.3±6.5 | 62.5 | Kangai Injection+OX | OX | SOX |
| Ma Dinding, 2021 | Jiangxi | 60 | Late stage | 63 | 44.21±3.21 | 50 | Huachansu preparations+OX | OX | SOX |
| Wang Donghui, 2021 | Shanxi | 96 | Middle stage | 42 | 51.37±0.38 | 58.33 | Shenqi Fuzheng Injection+OX | OX | XELOX |
| Wang Xinjie, 2021 | Henan | 67 | Late stage | 42 | 50.87±4.45 | 58.21 | Pingxiao Capsules+OX | OX | SOX |
| Xiao Qiong, 2021 | Henan | 70 | Late stage | 56 | 63.16±5.86 | 55.71 | Compound Mylabris preparations+OX | OX | FOLFOX6 |
| Zhao Yun, 2020 | Suzhou | 72 | Late stage | 63 | 59.25±5.63 | 55.56 | Kangai Injection+OX | OX | FOLFOX |
| Tan Tian, 2020 | Guangzhou | 69 | Middle stage | 30 | 54.28±2.63 | 57.97 | Kangai Injection+OX | OX | FOLFOX |
| Chen Xiaoting, 2020 | Wuhan | 120 | Late stage | 42 | 58.55±2.39 | 78.33 | Shenqi Fuzheng Injection+OX | OX | SOX |
| Hu Ying, 2020 | Jiangsu | 58 | Late stage | 63 | 58.04±2.91 | 56.9 | Huai'er Granules+OX | OX | SOX |
| Chen Zonghui, 2020 | Shandong | 64 | Middle stage | 84 | 58.3±2.56 | 54.69 | Shenqi Fuzheng Injection+OX | OX | XELOX |
| Liu Baoxinzi, 2020 | Jiangsu | 90 | Late stage | 21 | 58.4±7.5 | 82.22 | Shenmai Injection+OX | OX | FOLFOX4 |
| Shi Zhengwei, 2020 | Henan | 82 | Late stage | 42 | 49.68±3.13 | 53.66 | Shenqi Fuzheng Injection+OX | OX | SOX |
| Li Ruiqing, 2020 | Henan | 62 | Late stage | 84 | 66.98±7.08 | 66.13 | Aidi Injection+OX | OX | SOX |
| Lv Bole, 2020 | Henan | 80 | Late stage | 56 | 58.55±2.39 | 60 | Compound Kushen Injection+OX | OX | FOLFOX4 |
| Yang Qingwei, 2020 | Gansu | 60 | Late stage | 28 | 53+21.32 | 53.33 | Kanglaite Injection+OX | OX | SOX |
| Yu Hao, 2020 | Jiangxi | 200 | Late stage | 120 | 56.65±4.7 | 55.5 | Compound Kushen Injection+OX | OX | FOLFOX4 |
| Chen Yanxia, 2020 | Henan | 90 | Late stage | 180 | 46.85±6.83 | 51.11 | Aidi Injection+OX | OX | SOX |
| Zhai Hongfang, 2021 | Hebei | 118 | Late stage | 112 | 44.03±6.27 | 51.69 | Compound Mylabris preparations+OX | OX | FOLFOX4 |
| Liu Yong, 2020 | Suzhou | 60 | Middle stage | 42 | 42.87±13.94 | 53.33 | Kanglixin Capsules+OX | OX | SOX |
| Li Yaoxuan, 2020 | Anhui | 60 | Late stage | 84 | 73±4.5 | 53.33 | Huachansu preparations+OX | OX | SOX |
| Yan Xiaoxiao, 2020 | Shanxi | 64 | Late stage | 63 | 58.32±8.75 | 67.19 | Huai'er Granules+OX | OX | SOX |
| Qian Yumei, 2020 | Zhengzhou | 104 | Late stage | 84 | 54.15±4.04 | 55.77 | Aidi Injection+OX | OX | SOX |
| Wang Huifeng, 2020 | Shanghai | 82 | Late stage | 63 | 59.17±6.3 | 56.1 | Xiaoaiping Injection+OX | OX | SOX |
| Bi Qiong, 2019 | Beijing | 85 | Late stage | 63 | 56.9±8.14 | 60 | Compound Mylabris preparations+OX | OX | XELOX |
| Kong Jiong, 2019 | Suzhou | 60 | Late stage | 84 | 45.2±2.63 | 55 | Aidi Injection+OX | OX | FOLFOX6 |
| Zhu Yongfu, 2019 | Hubei | 60 | Late stage | 84 | 54.97±6.634 | 80 | Aidi Injection+OX | OX | SOX |
| Liu Gang, 2019 | Shandong | 92 | Late stage | 30 | 56.65±3.83 | 61.96 | Zhenqi Fuzheng Granules+OX | OX | FOLFOX4 |
| Yan Lihui, 2019 | Liaoning | 70 | Late stage | 126 | 49.13±6.41 | 54.29 | Huachansu preparations+OX | OX | XELOX |
| Ma Yubin, 2014 | Qinghai | 108 | Late stage | 84 | 46.80±5.25 | 81.50% | Ya Dan Zi Oil Emulsion Injection+OX | OX | XELOX |
| Wang Pei, 2014 | Henan | 84 | Middle and Late stage | 14 | 65.05±3.39 | 53.60% | Shenqi Fuzheng Injection+OX | OX | FOLFOX4 |
| Su Lianming, 2014 | Heilongjiang | 62 | NR | 72 | 64.87±8.36 | 58.10% | Yangzheng Xiaoji Capsules+OX | OX | FOLFOX4 |
| Xu Jinxiu, 2013 | Weihai | 120 | Late stage | 41 | 18~75 | 53.33% | Compound Kushen Injection+OX | OX | FOLFOX |
| Wang Jian, 2013 | Hunan | 100 | Late stage | 84 | 58.2±5.33 | 68.00% | Aidi Injection+OX | OX | SOX |
| Hu Xiaona, 2013 | Henan | 50 | Late stage | 28 | 70±6.51 | 54.00% | Aidi Injection+OX | OX | FOLFOX4 |
| Wang Jian, 2013 | Zhejiang | 60 | Late stage | 90 | 52±12.46 | 66.67% | Ya Dan Zi Oil Emulsion Injection+OX | OX | FOLFOX4 |
| Li Huiying, 2013 | Shanghai | 70 | Late stage | 56 | 65.21±8.02 | 50.00% | Shenqi Fuzheng Injection+OX | OX | FOLFOX4 |
| Huang Jianfei, 2013 | Zhejiang | 72 | NR | NR | 61.43±11.20 | 56.94% | Xiaoaiping Injection+OX | OX | FOLFOX4/XELOX/EOF |
| Lv Xiaoming, 2013 | Shaanxi | 86 | Late stage | 42 | 69.53±0.5 |  | Compound Mylabris preparations+OX | OX | FOLFOX |
| Li Pihong, 2012 | Wenzhou | 135 | Late stage | 21 | 52 ±0.5 | 58.52% | Shenfu Injection+OX | OX | XELOX |
| Chen Lingling, 2012 | Zhejiang | 70 | Late stage | 56 | 49.5±1.5 | 67.14% | Shenqi Fuzheng Injection+OX | OX | FOLFOX4 |
| Yang Xuemei, 2012 | Guiyang | 80 | Late stage | 42 | 67±5.8 | 62.50% | Kangai Injection+OX | OX | FOLFOX |
| Ren Yanzhai, 2012 | NR | 65 | Late stage | 52 | 52~73 | 46.20% | Shenqi Fuzheng Injection+OX | OX | FOLFOX4 |
| An Guangwen, 2012 | Shanxi | 70 | Late stage | 42 | 56.63±1.51 | 51.43% | Kangai Injection+OX | OX | FOLFOX4 |
| Yan Hongxia, 2012 | Shanxi | 66 | Late stage | 14 | 61.7 | 86.40% | Aidi Injection+OX | OX | FOLFOX4 |
| Wu Yacong, 2012 | Shanxi | 100 | Late stage | 28~56 | 57.5±0.50 | 71.00% | Ya Dan Zi Oil Emulsion Injection+OX | OX | FOLFOX4 |
| Ruan Xinjian, 2012 | Beijing | 54 | Late stage | 42 | 35~70 | 48.15% | Shenqi Fuzheng Injection+OX | OX | SOX |
| Chen Liping, 2012 | Jiangxi | 50 | Late stage | 42 | 54~75 | 58.00% | Aidi Injection+OX | OX | EOF |
| Guo Hairong, 2012 | Henan | 72 | Middle and Late stage | 10 | 52±0.3 | 65.00% | Astragalus preparations+OX | OX | FOLFOX4 |
| He Changshun, 2012 | Xiangxiang | 65 | Late stage | 42 | 48.5 | 66.20% | Aidi Injection+OX | OX | FOLFOX |
| Yang Jiewen, 2012 | Hangzhou | 58 | Late stage | 84 | 64.8±0.60 | 51.72% | Compound Kushen Injection+OX | OX | FOLFOX6 |
| Gao Jie, 2012 | Shandong | 60 | Late stage | 56 | 36~69 | 70.00% | Shenmai Injection+OX | OX | Oxaliplatin |
| Zou Haiping, 2012 | NR | 60 | Late stage | 10 | 57.84±1.31 | 57.00% | Huachansu preparations+OX | OX | EOF |
| Qin Hongbin, 2012 | NR | 48 | Late stage | 42 | 29-72 | 73.00% | Compound Kushen Injection+OX | OX | FOLFOX4 |
| Zhu Weikang, 2012 | Shanghai | 64 | Late stage | 28 | 62.3±0.55 | 48.44% | Huachansu preparations+OX | OX | XELOX |
| Zhang Wenhui, 2012 | Baotou | 30 | Late stage | 144 | 60-80 | 67.00% | Shenqi Fuzheng Injection+OX | OX | SOX |
| Qian Yanjun, 2012 | NR | 57 | Late stage | 84 | 26~69 | 81.00% | Jinlong Capsules+OX | OX | FOLFOX4 |
| Yuan Caijun, 2012 | Liaoning | 66 | Late stage | 63 | 65~85 | 51.52% | Compound Kushen Injection+OX | OX | XELOX |
| Fan Chengmei, 2011 | Shanghai | 51 | Late stage | 40 | 57.3±0.55 | 65.00% | Aidi Injection+OX | OX | SOX |
| Jiang Bogang, 2011 | Jiangsu | 64 | Late stage | 42 | 51 | 64.00% | Ya Dan Zi Oil Emulsion Injection+OX | OX | XELOX |
| Wang Ming, 2011 | Shandong | 80 | Late stage | 56 | 50.5±1.51 | 54.00% | Shenqi Fuzheng Injection+OX | OX | FOLFOX4 |
| Zhang Junxiang, 2011 | Hebei | 63 | NR | 42 | 29~72 | 57.14% | Ginseng Polysaccharide Injection+OX | OX | FOLFOX4 |
| Luo Pengfei, 2011 | Hunan | 43 | Late stage | 42 | 41~75 | 65.12% | Shenqi Fuzheng Injection+OX | OX | FOLFOX |
| Liu Hong, 2011 | Beijing | 85 | NR | 28 | 65±7 | 54.11% | Shenqi Fuzheng Injection+OX | OX | FOLFOX4 |
| Han Quanli, 2011 | Beijing | 78 | Late stage | 28 | 54.5±0.3 | 56.41% | Compound Kushen Injection+OX | OX | FOLFOX4 |
| Liu Yuehua, 2011 | Zhejiang | 65 | Late stage | 84 | 56 | 58.50% | Astragalus Polysaccharides+OX | OX | FOLFOX |
| Li Yuying, 2010 | Qinghai | 74 | Late stage | 42 | 23-76 | 69.00% | Lentinan+OX | OX | FOLFOX4 |
| Wen Xu, 2010 | Jiangsu | 56 | Late stage | 21 | 60.3±7.2 | 82.14% | Aidi Injection+OX | OX | FOLFOX |
| Ke Yufeng, 2010 | Jiangxi | 45 | Late stage | 28 | 28~75 | 53.33% | Aidi Injection+OX | OX | XELOX |
| Zhang Liqiang, 2010 | Xinzhou | 68 | Late stage | 168 | 54.8 | 57.40% | Compound Kushen Injection+OX | OX | FOLFOX4 |
| Huang Zhifen, 2009 | Guangxi | 30 | Late stage | 42 | 45.5±10 | 70.00% | Compound Kushen Injection+OX | OX | FOLFOX |
| Wei Aiqing, 2009 | Gansu | 71 | Late stage | 56 | 58±2.52 | 67.00% | Shengxue Granules+OX | OX | FOLFOX4 |

# **Appendix 7 Detailed guidance on the risk of bias assessment**

| **Item 1: Was the** **allocation sequence** **adequately generated?** |
| --- |
| 1. **Definitely Yes:**   Trials that assign participants to alternative interventions using a randomly generated sequence.  Examples of methods for developing a randomly generated allocation sequence include a random number generator, random number table, coin tossing, shuffling cards or envelopes, and throwing dice. If a trial is described as 'randomized' without any additional details related to how the allocation sequence was developed, we will assume that the allocation sequence was appropriately developed.  *Minimization may be implemented without a random element, and this is considered to be equivalent to being random. |
|  |
|  |
|  |
| 1. **Probably Yes: *** A simple statement such as ‘we randomly allocated’ or ‘using a randomized design’ is often insufficient to be confident that the allocation sequence was genuinely randomized |
|  |
|  |
| 1. **Probably No: NONE**   **NONE** |
|  |
|  |
| 1. **Definitely No:**   The investigators describe a non-random component in the sequence generation process. Usually, the description would involve some systematic, non-random approach, for example:  • Sequence generated by odd or even date of birth;  • Sequence generated by some rule based on date (or day) of admission;  • Sequence generated by some rule based on hospital or clinic record number.  Other non-random approaches happen much less frequently than the systematic approaches mentioned above and tend to be obvious. They usually involve judgement or some method of non-random categorization of participants, for example:  • Allocation by judgement of the clinician;  • Allocation by preference of the participant;  • Allocation based on the results of a laboratory test or a series of tests;  • Allocation by availability of the intervention. |
|  |
|  |
|  |
|  |
| **Item 2: Was the allocation adequately concealed?** |
| 1. **Definitely Yes:**   Participants and investigators enrolling participants could not foresee assignment because one of the following, or an equivalent method, was used to conceal allocation:  • Central allocation (including telephone, web-based, and pharmacy-controlled, randomization);  • Sequentially numbered drug containers of identical appearance;  • Sequentially numbered, opaque, sealed envelopes. |
| 1. **Probably Yes:**   Trials in which healthcare providers were blind to the intervention but which provide no information on allocation concealment and in which there are no major baseline imbalances. |
| 1. **Probably No:**   Insufficient information to permit judgement of risk of bias. This is usually the case if the method of concealment is not described or not described in sufficient detail to allow a definite judgement. |
| 1. **Definitely No:**   Participants or investigators enrolling participants could possibly foresee assignments and thus introduce selection bias, such as allocation based on:  • Using an open random allocation schedule (e.g. a list of random numbers);  • Assignment envelopes were used without appropriate safeguards (e.g. if envelopes were unsealed or nonopaque or not sequentially numbered);  • Alternation or rotation;  • Date of birth;  • Case record number;  • Any other explicitly unconcealed procedure. |
| **Item 3: Blinding of patients and healthcare providers** |
| 1. **Definitely Yes:**   Any one of the following:  • No blinding or incomplete blinding of participants and personnel, but the review authors judge that the outcome is not likely to be influenced by lack of blinding;  • Blinding of participants and key study personnel ensured, and unlikely that the blinding could have been broken;  • If it is described as “double-blind,” or “double-dummy”  • Explicit statement that a group of interest was blinded → LOW risk of bias for that group  • Explicit statement “investigators were blinded → LOW risk of bias for study personnel |
| 1. **Probably Yes: NONE** |
| 1. **Probably No:**   • Insufficient information to permit judgment  • Therapy trials in which healthcare providers are described as being blind to the intervention but allocation concealment was inadequate. |
| 1. **Definitely No:**   Any one of the following:  • No blinding or incomplete blinding, and the outcome or outcome measurement is likely to be influenced by lack of blinding.  • Blinding of key study participants and personnel attempted, but likely that the blinding could have been broken;  • Either participants or some key study personnel were not blinded, and the non-blinding of others likely to introduce bias.  • Explicit statement that a group of interest was not blinded  • Explicit description of the trial as “open label” or “unblinded”, or “single blinded”  Please note, if the outcome is an objective outcome (e.g., PSG outcomes), the risk of bias will generally be of less concern |
| **Item 4: Blinding of outcome assessors** |
| 1. **Definitely Yes:**   Any one of the following:  • No blinding of outcome assessment, but the review authors judge that the outcome measurement is not likely to be influenced by lack of blinding;  • Blinding of outcome assessment ensured, and unlikely that the blinding could have been broken.  Explicit statement “investigators were blinded → LOW risk of bias for outcome assessors  No explicit statement about blinding status of either patients, health care providers, data collectors, or outcome adjudicators, and:  • Placebo controlled drug trial → LOW risk of bias for those groups  • Active control drug trial (A vs. B) and mention of “double -dummy” or that medications were identical or matched → LOW risk of bias for those groups |
| 1. **Probably Yes:** |
| 1. **Probably No:**   Any one of the following:  • Insufficient information to permit judgment ;  No explicit statement about blinding status of either patients, health care providers, data collectors, or outcome adjudicators, and:  • Active control drug trial (A vs. B) but no mention of “double-dummy” or that medications were identical or matched |
| 1. **Definitely No:**   Any one of the following:  • No blinding of outcome assessment, and the outcome measurement is likely to be influenced by lack of blinding;  • Blinding of outcome assessment, but likely that the blinding could have been broken, and the outcome measurement is likely to be influenced by lack of blinding.  Explicit statement that a group of interest was not blinded  Explicit description of the trial as “open label” or “unblinded” |
| **Item 5: Was loss to follow-up (****missing outcome data) infrequent?** |
| 1. **Definitely Yes:**   Trials in which missing outcome data (including outcome data that has been imputed) < 10%. |
| 1. **Probably Yes:**   Trials in which missing outcome data (including outcome data that has been imputed) is between 10% to 15% and missing outcome data is unlikely to be related to the true outcome and there is no imbalance in numbers of or reasons for missing data across intervention groups. |
| 1. **Probably No:**   Trials in which missing outcome data (including outcome data that has been imputed) is between 10% to 15% and missing outcome data is likely to be related to the true outcome or there are imbalances in numbers of or reasons for missing data across intervention groups. |
| 1. **Definitely No:**   Trials in which missing outcome data (including outcome data that has been imputed) > 15%. |
| **Item 6: Are reports of the study free of selective outcome reporting?** |
| 1. **Definitely Yes:**   Results for outcomes that were analyzed and reported according to a pre-specified statistical analysis plan or protocol (including the timepoint for the measurement of the outcome). |
| 1. **Probably Yes:**   Results for outcomes that were analyzed and reported but that were not prespecified in a statistical analysis plan or protocol but the timepoint at which results are reported is consistent with the timepoint for other outcomes in the trial report or there is little reason to believe the outcome was selectively reported.  Please note that outcomes that were not prespecified in a protocol or statistical analysis plan and that are reported in the trial preprint or publication should be rated at probably low risk of bias unless there are other important reasons to suspect that results for those outcomes were selectively reported (e.g., results are presented at timepoints that don’t match the timepoints reported for other outcomes). |
| 1. **Probably No:**   Results for outcomes that were analyzed and reported but that were not prespecified in a statistical analysis plan or protocol but the timepoint at which results are reported is not consistent with the timepoint for other outcomes in the trial report or there are other reasons to believe that the outcome is selectively reported. |
| 1. **Definitely No:**   Results for outcomes that were analyzed and reported for which there are inconsistencies with the statistical analysis plan or protocol. These inconsistencies may include outcome measures of interest or the timepoints for the measurement of outcomes. |

# **Appendix 8 The results of the risk of bias assessment**

| **Author, year** | **Allocation sequence  generated** | **allocation concealment** | **Blinding of patients and healthcare providers** | **Blinding of outcome assessors** | **Incomplete data** | **selective outcome reporting** | **Other bias** | **Overall** |
| --- | --- | --- | --- | --- | --- | --- | --- | --- |
| Gao Nana, 2023 | DY | PN | PN | PN | DY | PY | DY | High |
| Jia Jianwei, 2009 | PY | PN | PN | DY | DY | PY | DY | Low |
| Hu Shangying, 2023 | PY | PN | PN | PN | DY | PY | DY | High |
| Chen Naijie, 2008 | PY | PN | PN | DY | DY | PY | DY | Low |
| Fan Xiaoqiang, 2008 | PY | PN | PN | DY | DY | PY | DY | Low |
| Shi Yan, 2006 | PY | PN | PN | DY | DY | PY | DY | Low |
| Qian Feng, 2006 | PY | PN | PN | DY | DY | PY | DY | Low |
| Guo Xiangyu, 2013 | DY | PN | PN | DY | DY | PY | DY | Low |
| Wang Xiaoqing, 2013 | DY | PN | PN | DY | DY | PY | DY | Low |
| Ma Ji, 2019 | DY | PN | PN | DY | DY | PY | DY | Low |
| Bai Xiaohui, 2024a | DY | PN | PN | DY | DY | PY | DY | Low |
| Wang Huihui, 2023 | DY | PN | PN | DY | DY | PY | DY | Low |
| Song Yaxi, 2022 | PY | PN | DY | DY | DY | PY | DY | Low |
| Yu Yang, 2018 | DY | PN | PN | PN | DY | PY | DY | High |
| Deng Qingming, 2018 | DY | PN | PN | PN | DY | PY | DY | High |
| Gao Pan, 2018 | DY | PN | PN | DY | DY | PY | DY | Low |
| Hu Qiang, 2017 | DY | PN | PN | PN | DY | PY | DY | High |
| Cui Hongxia, 2015 | DY | PN | PN | DY | DY | PY | DY | Low |
| Zhang Yonghong, 2014 | PY | PN | PN | DY | DY | PY | DY | Low |
| Han Lingmin, 2014 | DY | PN | PN | DY | DY | PY | DY | Low |
| Gao Qiujie, 2014 | PY | PN | DY | DY | DY | PY | DY | Low |
| Zhang Shengqi, 2013 | PY | PN | DY | DY | DY | PY | DY | Low |
| Sun Qiujue, 2021 | DY | PN | PN | PN | DY | PY | DY | High |
| Jiang Xueying, 2024 | DY | PN | PN | DY | DY | PY | DY | Low |
| Zhao Jie, 2020 | DY | PN | PN | PN | DY | PY | DY | High |
| Li Huimin, 2021 | DY | PN | PN | PN | DY | PY | DY | High |
| Wang Xiangyang, 2020 | DY | PN | PN | DY | DY | PY | DY | Low |
| Zhang Zhiye, 2019 | PY | PN | PN | PN | DY | PY | DY | High |
| Zhang Guoping, 2021 | DY | PN | PN | DY | DY | PY | DY | Low |
| Xie Yufen, 2015 | DY | PN | PN | DY | DY | PY | DY | Low |
| Zhao Hongbo, 2020 | PY | PN | PN | PN | DY | PY | DY | High |
| Fang Xianying, 2010 | PY | PN | PN | PN | DY | PY | DY | High |
| Dong Lin, 2019 | DY | PN | PN | DY | DY | PY | DY | Low |
| Wang Yutao, 2013 | PY | PN | PN | DY | DY | PY | DY | Low |
| Tong Gandong, 2019 | DY | PN | PN | PN | DY | PY | DY | High |
| Sun Xin, 2019 | PY | PN | PY | DY | DY | PY | DY | Low |
| Si Qian, 2019 | DY | PN | PN | PN | DY | PY | PY | High |
| Song Bo, 2019 | DY | PN | PN | PN | DY | PY | PY | High |
| Wu Xiaofeng, 2019 | DY | PN | PN | DY | DY | PY | PY | Low |
| Yan Bingjie, 2019 | DY | PN | PN | PN | DY | PY | PY | High |
| Cao Jun, 2019 | DY | PN | PN | PN | DY | PY | PY | High |
| Wang Junyan, 2018 | DY | PN | PN | PN | DY | PY | PY | High |
| Xing Yanli, 2018 | DY | PN | PN | DY | DY | PY | PY | Low |
| Wang Zengfang, 2018 | DY | PN | PN | PN | DY | PY | PY | High |
| Chen Taoli, 2018 | DY | PN | PN | PN | DY | PY | PY | High |
| Liu Xia, 2018 | DY | PN | PN | PN | DY | PY | PY | High |
| Gao Nana, 2018 | PY | PN | PN | PN | DY | PY | PY | High |
| Zhou Qingbin, 2017 | PY | PN | PY | PN | DY | PY | PY | Low |
| Wang Rui, 2018 | DY | PN | PN | PN | DY | PY | PY | High |
| Yang Fang, 2018 | PY | PN | PN | PN | DY | PY | PY | High |
| Cui Yougang, 2017 | PY | PN | PN | PN | DY | PY | PY | High |
| Qiu Ying, 2017 | PY | PN | PN | PN | DY | PY | PY | High |
| Yang Jiezhi, 2017 | PY | PN | PN | DY | DY | PY | PY | Low |
| Feng Xiemin, 2017 | DY | PN | PN | PN | DY | PY | PY | High |
| Yang Qiang, 2017 | DY | PN | PN | PN | DY | PY | PY | High |
| Chen Qingxian, 2017 | DY | PY | PN | PN | DY | PY | PY | High |
| Shen Gang, 2017 | PY | PN | PN | DY | DY | PY | PY | Low |
| Li Ruilin, 2017 | PY | PN | PN | DY | DY | PY | PY | Low |
| Xu Jianlin, 2017 | DY | PN | PN | PN | DY | PY | PY | High |
| Cui Lihua, 2017 | PY | PY | PN | PN | DY | PY | PY | High |
| Liu Hongbo, 2017 | DY | PN | PN | PN | DY | PY | PY | High |
| Hu Pei, 2017 | PY | PN | PN | DY | DY | PY | PY | Low |
| Fang Dong, 2016 | PY | PY | PN | PN | DY | PY | PY | High |
| Zheng Yu, 2016 | PY | PN | PN | DY | DY | PY | PY | Low |
| Xie Maogao, 2016 | DY | PN | PN | PN | DY | PY | PY | High |
| Wang Lanrong, 2016 | PY | PN | PN | PN | DY | PY | PY | High |
| Ding Boyong, 2016 | DY | PN | PN | DY | DY | PY | PY | Low |
| Xie Junfeng, 2016 | DY | PN | PN | DY | DY | PY | PY | Low |
| Zhao Weimin, 2016 | PY | PN | PN | PN | DY | PY | PY | High |
| Xu Yongmei, 2016 | PY | PN | PN | DY | DY | PY | PY | Low |
| Sun Yinping, 2016 | DY | PN | PN | DY | DY | PY | PY | Low |
| Chen Jingsheng, 2016 | PY | PN | PN | PN | DY | PY | PY | High |
| Jin Haitao, 2016 | DY | PN | PN | PN | DY | PY | PY | High |
| Zhu Xiaopeng, 2016 | PY | PN | PN | PN | DY | PY | PY | High |
| Wu Ting, 2016 | PY | PN | PN | DY | DY | PY | PY | Low |
| Kang Yi, 2015 | DY | PN | PN | PN | DY | PY | PY | High |
| Li Xiaoli, 2015 | PY | PN | PY | PN | DY | PY | PY | Low |
| Zhang Xuemiao, 2015 | DY | PN | PN | PN | DY | PY | PY | High |
| Cao Feng, 2015 | PY | PN | PN | DY | DY | PY | PY | Low |
| Yin Qin, 2015 | PY | PN | PN | PN | DY | PY | PY | High |
| Lin Qi, 2015 | PY | PN | PN | PN | DY | PY | PY | High |
| Gao Liang, 2015 | PY | PN | PN | DY | DY | PY | PY | Low |
| Yao Xiaojian, 2015 | PY | PN | PN | PN | DY | PY | PY | High |
| Xiong Lin, 2015 | PY | PN | PN | DY | DY | PY | PY | Low |
| Zhang Hao, 2015 | PY | PN | PN | PN | DY | PY | PY | High |
| Ning Siqing, 2015 | DY | PN | PN | DY | DY | PY | PY | Low |
| Jiang Jun, 2015 | PY | PN | PN | DY | DY | PY | PY | Low |
| Xu Judi, 2015 | PY | PN | PY | PN | DY | PY | PY | Low |
| Wen Kang, 2014 | PY | PN | PN | PN | DY | PY | PY | High |
| Yang Zhiyong, 2014 | PY | PN | PN | DY | DY | PY | PY | Low |
| An Jirong, 2015 | PY | PN | PN | PN | DY | PY | PY | High |
| Wang Zhenguang, 2014 | PY | PN | PN | PN | DY | PY | PY | High |
| Liu Shuxun, 2014 | PY | PN | PN | PN | DY | PY | PY | High |
| Liu Kuanhao, 2014 | PY | PN | PN | PN | DY | PY | PY | High |
| Tian Hongnv, 2024 | DY | PN | PN | PN | DY | PY | PY | High |
| Bai Xiaohui, 2024b | DY | PN | PN | PN | DY | PY | PY | High |
| Xiao Kai, 2024 | DY | PN | PN | PN | DY | PY | PY | High |
| Liu Ming, 2024 | PY | PN | PN | PN | DY | PY | PY | High |
| Zhao Yi, 2024 | DY | PY | DY | DY | DY | PY | PY | Low |
| Zhang Xiaorui, 2024 | PY | PN | PN | PN | DY | PY | PY | High |
| Da Haitao, 2024 | DY | PN | PN | PN | DY | PY | PY | High |
| Peng Weizhen, 2024 | PY | PN | PN | PN | DY | PY | PY | High |
| Zhang Sheng, 2023 | PY | PN | PN | PN | DY | PY | PY | High |
| Zhao Zhenyu, 2023 | DY | PN | PN | PN | DY | PY | PY | High |
| Chen Weixiang, 2023 | DY | PN | PN | PN | DY | PY | PY | High |
| Luo Sumei, 2023 | DY | PN | PN | PN | DY | PY | PY | High |
| Wang Yaling, 2022 | DY | PN | PN | PN | DY | PY | PY | High |
| Du Ziwei, 2022 | DY | PN | PN | PN | DY | PY | PY | High |
| Zhang Shaohu, 2022 | DY | PN | PN | PN | DY | PY | PY | High |
| Li Pengyuan, 2022 | DY | PN | PN | PN | DY | PY | PY | High |
| Pan Jing, 2021 | DY | PN | PN | PN | DY | PY | PY | High |
| Gong Yu, 2021 | DY | PN | PN | PN | DY | PY | PY | High |
| Si Lianlian, 2021 | DY | PN | PN | PN | DY | PY | PY | High |
| Wen Minya, 2021 | DY | PN | PN | PN | DY | PY | PY | High |
| Chang Zhanguo, 2021 | DY | PN | PN | PN | DY | PY | PY | High |
| Yang Qingwei, 2021 | PY | PN | PN | PN | DY | PY | PY | High |
| Ruan Xinjian, 2021 | DY | PN | PN | PN | DY | PY | PY | High |
| Jin Liyan, 2021 | PY | PN | PN | PN | DY | PY | PY | High |
| Ma Dinding, 2021 | DY | PN | PN | PN | DY | PY | PY | High |
| Wang Donghui, 2021 | PY | PN | PN | PN | DY | PY | PY | High |
| Wang Xinjie, 2021 | PY | PN | PN | PN | DY | PY | PY | High |
| Xiao Qiong, 2021 | DY | PN | PN | PN | DY | PY | PY | High |
| Zhao Yun, 2020 | DY | PN | PN | PN | DY | PY | PY | High |
| Tan Tian, 2020 | PY | PN | DY | PN | DY | PY | PY | Low |
| Chen Xiaoting, 2020 | DY | PN | PN | PN | DY | PY | PY | High |
| Hu Ying, 2020 | DY | PN | PN | PN | DY | PY | PY | High |
| Chen Zonghui, 2020 | DY | PN | PN | PN | DY | PY | PY | High |
| Liu Baoxinzi, 2020 | DY | PN | PN | PN | DY | PY | PY | High |
| Shi Zhengwei, 2020 | DY | PN | PN | PN | DY | PY | PY | High |
| Li Ruiqing, 2020 | DY | PN | PN | PN | DY | PY | PY | High |
| Lv Bole, 2020 | DY | PN | PN | PN | DY | PY | PY | High |
| Yang Qingwei, 2020 | DY | PN | PN | PN | DY | PY | PY | High |
| Yu Hao, 2020 | DY | PN | PN | PN | DY | PY | PY | High |
| Chen Yanxia, 2020 | DY | PN | PN | PN | DY | PY | PY | High |
| Zhai Hongfang, 2021 | DY | PN | PN | PN | DY | PY | PY | High |
| Liu Yong, 2020 | PY | PN | PN | PN | DY | PY | PY | High |
| Li Yaoxuan, 2020 | DY | PN | PN | PN | DY | PY | PY | High |
| Yan Xiaoxiao, 2020 | DY | PN | PN | PN | DY | PY | PY | High |
| Qian Yumei, 2020 | DY | PN | PN | PN | DY | PY | PY | High |
| Wang Huifeng, 2020 | DY | PN | PN | PN | DY | PY | PY | High |
| Bi Qiong, 2019 | DY | PN | PN | PN | DY | PY | PY | High |
| Kong Jiong, 2019 | PY | PN | PN | PN | DY | PY | PY | High |
| Zhu Yongfu, 2019 | DY | PN | PN | PN | DY | PY | PY | High |
| Liu Gang, 2019 | DY | PN | PN | PN | DY | PY | PY | High |
| Yan Lihui, 2019 | DY | PN | PN | PN | DY | PY | PY | High |
| Ma Yubin, 2014 | PY | PN | PN | PN | DY | PY | PY | High |
| Wang Pei, 2014 | PY | PN | PN | PN | DY | PY | PY | High |
| Su Lianming, 2014 | DY | PN | PN | PN | DY | PY | PY | High |
| Xu Jinxiu, 2013 | DY | PN | PN | PN | DY | PY | PY | High |
| Wang Jian, 2013 | PY | PN | PN | PN | DY | PY | PY | High |
| Hu Xiaona, 2013 | PY | PN | PN | PN | DY | PY | PY | High |
| Wang Jian, 2013 | PY | PN | PN | PN | DY | PY | PY | High |
| Li Huiying, 2013 | PY | PN | PN | PN | DY | PY | PY | High |
| Huang Jianfei, 2013 | PY | PN | PN | PN | DY | PY | PY | High |
| Lv Xiaoming, 2013 | DY | PN | PN | PN | DY | PY | PY | High |
| Li Pihong, 2012 | DY | PN | PN | PN | DY | PY | PY | High |
| Chen Lingling, 2012 | PY | PN | PN | PN | DY | PY | PY | High |
| Yang Xuemei, 2012 | PY | PN | PN | PN | DY | PY | PY | High |
| Ren Yanzhai, 2012 | DY | PN | DY | DY | DY | PY | PY | Low |
| An Guangwen, 2012 | PY | PN | PN | PN | DY | PY | PY | High |
| Yan Hongxia, 2012 | PY | PN | PN | PN | DY | PY | PY | High |
| Wu Yacong, 2012 | PY | PN | PN | PN | DY | PY | PY | High |
| Ruan Xinjian, 2012 | PY | DY | PN | PN | DY | PY | PY | Low |
| Chen Liping, 2012 | PY | PN | PN | PN | DY | PY | PY | High |
| Guo Hairong, 2012 | PY | PN | PN | PN | DY | PY | PY | High |
| He Changshun, 2012 | PY | PN | PN | PN | DY | PY | PY | High |
| Yang Jiewen, 2012 | PY | PN | PN | PN | DY | PY | PY | High |
| Gao Jie, 2012 | PY | PN | PN | PN | DY | PY | PY | High |
| Zou Haiping, 2012 | PY | PN | PN | PN | DY | PY | PY | High |
| Qin Hongbin, 2012 | PY | PN | PN | PN | DY | PY | PY | High |
| Zhu Weikang, 2012 | PY | PN | PN | PN | DY | PY | PY | High |
| Zhang Wenhui, 2012 | PY | PN | PN | PN | DY | PY | PY | High |
| Qian Yanjun, 2012 | PY | PN | PN | PN | DY | PY | PY | High |
| Yuan Caijun, 2012 | PY | PN | PN | PN | DY | PY | PY | High |
| Fan Chengmei, 2011 | PY | PN | PN | PN | DY | PY | PY | High |
| Jiang Bogang, 2011 | DY | PN | PN | PN | DY | PY | PY | High |
| Wang Ming, 2011 | PY | PN | PN | PN | DY | PY | PY | High |
| Zhang Junxiang, 2011 | PY | PN | PN | PN | DY | PY | PY | High |
| Luo Pengfei, 2011 | PY | PN | PN | PN | DY | PY | PY | High |
| Liu Hong, 2011 | PY | PN | PN | PN | DY | PY | PY | High |
| Han Quanli, 2011 | PY | PN | PN | PN | DY | PY | PY | High |
| Liu Yuehua, 2011 | PY | PN | PN | PN | DY | PY | PY | High |
| Li Yuying, 2010 | PY | PN | PN | PN | DY | PY | PY | High |
| Wen Xu, 2010 | PY | PN | PN | PN | DY | PY | PY | High |
| Ke Yufeng, 2010 | PY | PN | PN | PN | DY | PY | PY | High |
| Zhang Liqiang, 2010 | PY | PN | PN | PN | DY | PY | PY | High |
| Huang Zhifen, 2009 | DY | PN | PN | PN | DY | PY | PY | High |
| Wei Aiqing, 2009 | PY | PN | PN | PN | DY | PY | PY | High |
| Leng Shuang, 2015 | PY | PN | PN | PN | DY | PY | PY | High |
| Note: DY: Definitely Yes; PY: Probably Yes; DN: Definitely No; PN: Probably No | | | | | | | | |
